# TOR Inhibitors Synergistically Suppress the Growth and Development of *Phytophthora infestans*, a Highly Destructive Pathogenic Oomycete

**DOI:** 10.3389/fmicb.2021.596874

**Published:** 2021-04-16

**Authors:** Shumin Zhang, A. Rehman Khalid, Dongmei Guo, Jingping Zhang, Fangjie Xiong, Maozhi Ren

**Affiliations:** ^1^School of Preclinical Medicine, North Sichuan Medical College, Nanchong, China; ^2^Institute of Urban Agriculture, Chinese Academy of Agricultural Sciences, Chengdu, China; ^3^School of Life Sciences, Chongqing University, Chongqing, China; ^4^Department of Plant Pathology, University of Poonch Rawalakot, Rawalkot, Pakistan

**Keywords:** TOR signaling pathway, rapamycin, AZD, pathogenic oomycetes, synergism, *P. infestans*

## Abstract

*Phytophthora infestans*, one of most famous pathogenic oomycetes, triggered the Great Irish Famine from 1845 to 1852. The target of rapamycin (TOR) is well known as a key gene in eukaryotes that controls cell growth, survival and development. However, it is unclear about its function in controlling the mycelial growth, sporulation capacity, spore germination and virulence of *Phytophthora infestans*. In this study, key components of the TOR signaling pathway are analyzed in detail. TOR inhibitors, including rapamycin (RAP), AZD8055 (AZD), KU-0063794 (KU), and Torin1, inhibit the mycelial growth, sporulation capacity, spore germination, and virulence of *Phytophthora infestans* with AZD showing the best inhibitory effects on *Phytophthora infestans.* Importantly, compared with a combination of RAP + KU or RAP + Torin1, the co-application of RAP and AZD show the best synergistic inhibitory effects on *P. infestans*, resulting in the reduced dosage and increased efficacy of drugs. Transcriptome analysis supports the synergistic effects of the combination of RAP and AZD on gene expression, functions and pathways related to the TOR signaling pathway. Thus, TOR is an important target for controlling *Phytophthora infestans*, and synergism based on the application of TOR inhibitors exhibit the potential for controlling the growth of *Phytophthora infestans.*

## Introduction

Many destructive pathogenic oomycetes infect various species including plants, aquatic animals, and mammals. These pathogens are responsible for the destruction of agriculture, forestry, husbandry, aquaculture, and human health (Kamoun, 2003; Phillips et al., 2008). Especially in agriculture, *Phytophthora infestans* (*P. infestans*) is regarded as one of the most harmful and widespread plant pathogens in the world. It causes potato late blight, triggering the Great Irish Famine of 1845–1852 which led to population displacement and changes in the global political pattern (Haas et al., 2009). Because of the tremendous influence of the Great Irish Famine on human history, *P. infestans* has attracted the attention of scientists, resulting in the birth of plant pathology. To date, it remains the most serious threat to potato production, which is the fourth largest food crop in the world.

Chemical oomyceticides are the main management method for controlling *P. infestans.* However, its large and complex genome sequence gives *P. infestans* highly evolutionary potential for rapid adaptability to chemical oomyceticides (Haas et al., 2009). For example, as a kind of single-target oomyceticide, mefenoxam was widely used to combat *P. infestans* in 1970s due to its high efficiency and oomycete specificity. Unfortunately, *P. infestans* rapidly became resistant to mefenoxam during the early 1980s (Richard et al., 2015). This resistance was due to the mutation of RNA polymerase 1 in *P. infestans*, which was the single target of this drug. Further research revealed that *P. infestans* can easily develop resistance to oomyceticides with a single target (Richard et al., 2015). Since single-target oomyceticides are widely overused in agriculture, a higher risk of resistance has developed following the increased dosage and reduced efficacy of such oomyceticides (Jia et al., 2009).

The search for new targets is an effective measure for delaying drug resistance. Target of rapamycin (TOR) is highly conserved Ser/Thr protein kinase in eukaryote cells, and its mutation can cause cell death. Previous studies have indicated that the TOR signaling pathway may be also conserved in *P. infestans* (Judelson and Ah-Fong, 2010; Van Dam et al., 2011; Tatebe and Shiozaki, 2017). Furthermore, TOR kinase protein controls vegetative development and virulence in *Fusarium graminearum* (Yu et al., 2014), indicating that TOR has the potential to be developed into a novel drug target. TOR kinase protein is sensitive to first-generation inhibitors such as rapamycin (RAP) and second-generation inhibitors such as AZD8055 (AZD), KU-0063794 (KU), and Torin1 (Garcia-Martinez et al., 2009; Chresta et al., 2010; Liu et al., 2010; Wang et al., 2013). Li et al. (2019) have pointed out that rapamycin can significantly inhibit the mycelial growth and conidial development of *Verticillium dahlia*. Chen et al. (2017) and Qiu et al. (2020) also found that *Phytophthora sojae* was sensitive to rapamycin. However, the effects of TOR inhibitors on *P. infestans* are still unexplored.

Oomyceticide synergy is a common method for delaying resistance. Because of multi-oomyceticides with different targets, the synergism of multi-oomyceticides delays resistance while decreasing the dosage and increasing the effect (Jia et al., 2009). Previous studies have shown synergistic effects between rapamycin and a series of drugs including JQ1, corticosteroids, CI-1040 and sunitinib (Roberge et al., 1995; Legrier et al., 2008; Dhong Hyun et al., 2014; Li et al., 2014). For example, synergistic antitumor action between rapamycin and CI-1040 was observed in human non-small cell lung cancer, thereby reducing the dosage and increasing the effect of CI-1040 (Legrier et al., 2008). Furthermore, rapamycin (first-generation TOR inhibitor) recruits FKBP12 orthologs to TORC1, sterically inhibiting some functions of TORC1. Meanwhile, AZD, KU, and Torin1 (second-generation TOR inhibitor) target the kinase domain (Sandra et al., 2008). Importantly, third-generation TOR inhibitors based on the combination of first-generation and second-generation TOR inhibitors effectively overcome TOR inhibitor-resistant mutations in tumors by targeting both the FRB and kinase domains (Rodrik-Outmezguine et al., 2016). However, it is unknown whether combining RAP with AZD/KU/Torin1 would inhibit *P. infestans* with a reduction dosage, increased effect, and delayed drug-resistance.

In this study, we confirmed the presence of the TOR signaling pathway in *P. infestans*, tested the synergistic effects of TOR inhibitors on this pathogen and analyzed the synergistic effects on the molecular level. The results and observations show that the TOR signaling pathway is conserved in *P. infestans.* TOR inhibitors, especially AZD, showed significant inhibitory effects on *P. infestans.* Importantly, the combination of RAP and AZD synergistically inhibited *P. infestans*, and this was confirmed by its synergistically regulatory effects on genes, functions and pathways. These findings have implications for the development of a synergistic agent based on TOR inhibitors for controlling *P. infestans*.

## Materials and Methods

### *P. infestans* Strains, Media and Culture Conditions

The internationally recognized standard strain T30-4 (A1 mating type) was provided by Dr. Suomeng Dong of Nanjing Agriculture University, China. T30-4 was retained in the laboratory and used for the whole genome sequencing of *P. infestans* (Haas et al., 2009). The field-collected strain 002 (A2 mating type) was provided by Dr. Weixing Shan of Northwest A&F University, China. Both were cultured in darkness at 18°C on Rye A agar.

### Effects of Drugs on Mycelial Growth, Sporulation Capacity, Spore Germination and Virulence of *P. infestans*

Mycelial disks of T30-4 or 002 with a diameter of 7 mm were cultured on a Rye A agar medium supplemented with the following concentrations of drugs: T30-4: RAP (0, 1, 3, 5, and 10 μM), Torin1 (0, 0.5, 2, 5, and 10 μM), KU (0, 2, 5, 8, and 10 μM), AZD (0, 0.3, 1, 2, and 5 μM); 002: RAP (0, 0.05, 0.1, 0.5, 1, and 3 μM), Torin1 (0, 0.5, 2, 5, and 10 μM), KU (0, 0.5, 1, 2, and 5 μM) and AZD (0, 0.05, 0.1, 0.2, 1, and 3 μM). DMSO was used as the control. On the 15th day of culture, the diameters of the drug-treated colonies were measured and the inhibition rates calculated. The inhibition rates (%) were calculated using the following formula: Inhibition rate = [(*D*−*M*)/(*D*−0.7)] × 100% [*D*: diameter of control colony; *M*: diameter of drug-treated colony]. Three biological repeats were performed for each experiment. Reagent information: Rapamycin (RAP) (Selleck, S1039), Torin1 (Selleck, S2827), KU-0063794 (KU) (Selleck, S1226), AZD8055 (AZD) (Selleck, S1555).

Sporangia of T30-4 and 002 were washed with water from plates of AZD-treated and DMSO-treated colonies, then counted. AZD concentration: T30-4: 0, 0.3, 1, and 2 μM; 002: 0, 0.1, 0.2, and 1 μM. Next, sporulation capacity (sporangia per unit of area) was calculated using the following formula: sporulation capacity = total sporangia number of drug-treated colony or DMSO-treated colony/area of drug-treated colony or DMSO-treated colony. Three biological repeats were performed for each experiment.

Mycelia were cultured in darkness at 18°C on Rye A agar for 14 days. After 14 days, sporangia were produced. Then, sporangia were collected by washing off 14-day-old T30-4 or 002 strains with water. The concentration of spores was then adjusted to 40 spores/μL. Next, the spore suspensions were supplemented with concentrations of AZD (0, 0.5, 5, and 10 μM for T30-4; 0, 0.2, 2, and 4 μM for 002), respectively. DMSO was used as the control. After being cultured at 18°C for 16 h, spore germination was counted. Three biological repeats were performed for each experiment.

The spore concentration of T30-4 and 002 strains was adjusted to 40 spores/μL. The spore suspensions were then supplemented with concentrations of AZD (0, 0.5, 5, and 10 μM for T30-4; 0, 0.2, 2, and 4 μM for 002), respectively. DMSO was used as the control. After mixing with AZD for 10 min, 20 μL of drug-treated spore suspensions were immediately dropped on ten leaves (3–4 weeks mature potato) or tubers (2 months old potato). The mixture suspensions generally evaporated 5–10 min after inoculation. The inoculated leaves or tubers were maintained at 18°C in a 12 h/12 h light/dark cycle under 90% humidity for 4 days. After 4 days, inoculated leaf and tuber of the control and drug-treated potato were collected and frozen in liquid nitrogen for q-PCR (Avrova et al., 2003; Jahan et al., 2015). Then the biomass of fresh mycelia on the leaf and tuber was quantitatively analyzed by q-PCR and calculated according to a previously published study (Avrova et al., 2003). Data was normalized to the *EF1* DNA levels of potato. Three biological repeats were performed for each experiment.

### Effects of the Combination of First-Generation TOR Inhibitors and Second-Generation TOR Inhibitors on the Mycelial Growth of *P. infestans*

The 7-mm-diameter mycelial disks of T30-4 and 002 were maintained on a Rye A agar medium and supplemented with concentrations of RAP (0, 0.05, 0.1, 0.5, 1, 3 μM, Supplementary Table 1B), AZD (0, 0.01, 0.05, 0.1, 0.2 μM, Supplementary Table 1B), KU (0, 0.5, 1, 2, 5 μM, Supplementary Table 1C), and Torin1 (0, 0.5, 2, 5, 10 μM, Supplementary Table 1D), and various combinations of RAP + AZD/KU/Torin1 (Supplementary Tables 1B–D). DMSO was used as the control. The basal concentrations of RAP, AZD, KU, and Torin1 were 0.01 M, 0.01 M, 0.01 M, and 0.01 M, respectively. On the 15th day of culture, the colony diameters were measured and the inhibition rates calculated. Three biological repeats were performed for each experiment.

The interactions between RAP and AZD/KU/Torin1 were quantitatively analyzed using the Combination Index (CI) value (Xiong et al., 2017). The relationships of drug interaction were defined according to a previously published study (Chou, 2006): antagonism (CI > 1), synergism (CI < 1) and additive effect (CI = 1). The growth value (%) was calculated by the following formula: [(*T*−0.7)/(*D*−0.7)] × 100 (*T*: diameters of 15-day-old drug-treated colony; *D*: diameters of 15-day-old DMSO-treated control colony). The combination index (CI) and half maximal inhibitory concentration (IC50) were measured by using CompuSyn software (Chou and Talalay, 1984). The affected value (Fa) was measured by the formula (100 – % growth value)/100, which evaluated the growth inhibition of the colony by the drug.

### Transcriptome Assay

*Phytophthora infestans* T30-4 was cultured in a liquid medium in darkness at 18°C for 14 days. After 14 days, the mycelia of T30-4 were transplanted to a liquid medium supplement with 0.5 μM AZD (IC50), 5 μM RAP (IC50), 0.5 μM AZD + 5 μM RAP, and the largest volumes of DMSO as the control were cultured in darkness at 18°C for 24 h. The mycelia were then collected and frozen in liquid nitrogen for RNA extraction. The subsequent steps are described in a previous study (Zhang et al., 2018). Genes with an adjusted *p*-value of <0.05 were considered differentially expressed genes (DEGs). GO terms (Gene ontology) and KEGG pathways (Kyoto encyclopedia of genes and genomes) with a *padj* < 0.05 were considered significant. Three biological repeats were performed for each experiment. The transcriptome datasets were submitted to NCBI and the accession number—PRJNA415528.

### Quantitative Real-Time PCR (qRT-PCR) Assay

Seven genes were selected for qRT-PCR. The mycelia of *P. infestans* T30-4 were prepared as described in the transcriptome. The total RNA was isolated using the RNAprep Pure Plant Kit (TianGen Biotech, Beijing, China). Next, 1 μg of total RNA was used for a reverse transcription reaction using the PrimeScript RT Kit (TAKARA Biotech). Thereafter, the qRT-PCR assays were conducted on a Bio-Rad CFX96 System using the TransStart TopGreen qPCR Super Mix (TransGen Biotech). The primers used for qRT-PCR assay are listed in Supplementary Table 1A. Three biological repeats were performed for each experiment.

### Statistical Analysis

The statistical software used was the GraphPad Prism Version 5.01 program. Each value represents the mean ± SD of three independent experiments. Two-tailed Student’s *t*-test analysis was used to calculate the *p*-values (^∗^*P* < 0.05, ^∗∗^*P* < 0.01, ^∗∗∗^*P* < 0.001) or (lower case letters indicate significant difference, *p* < 0.05).

## Results

### The Conserved TOR Signaling Pathway Exists in *P. infestans*

**FIGURE 1:**
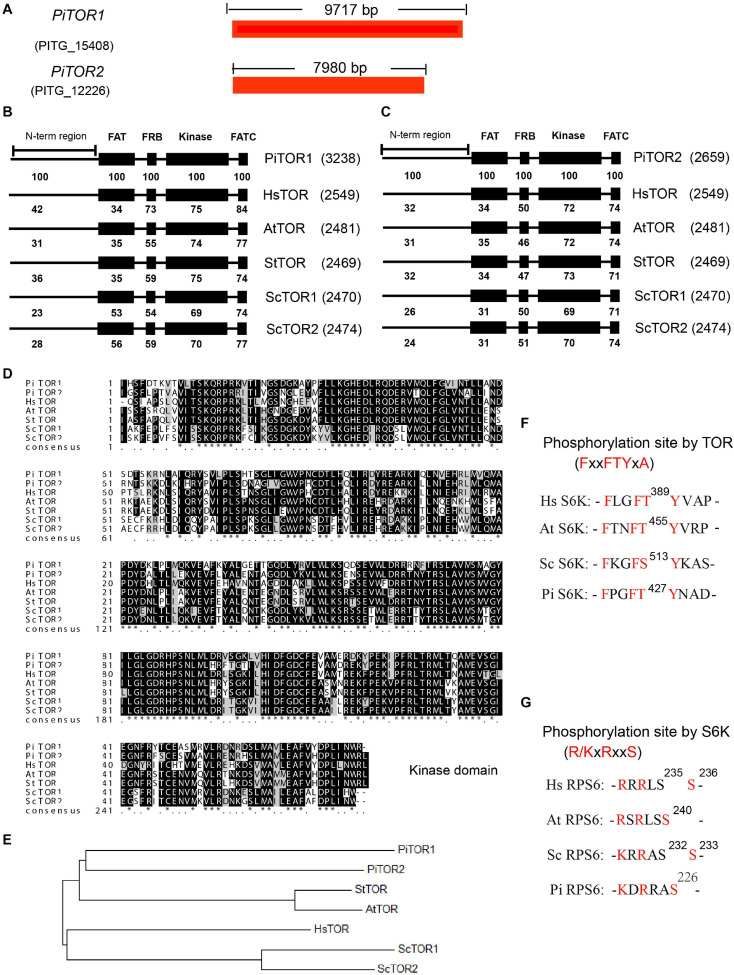
Sequence and structure analysis of the *PiTOR* gene in *P. infestans*. **(A)** Gene length of PiTOR1 and PiTOR2 in *P. infestans*. PiTOR1 and PiTOR2 are the homologous proteins of TORs in *P. infestans*. Red represents exons. No intron existed in PiTOR. **(B,C)** Comparison of four main TOR domains (FAT, FRB, kinase, and FATC) in Pi, Hs, At, St, and Sc. **(D)** Comparison of kinase domain in Pi, Hs, Sc, At, and St. **(E)** Evolutionary relationship of homologies of TORs in Pi, Sc, St, At, and Hs. The neighbor-joining method of MEGA5.0 was used to analyze the evolutionary relationship. **(F,G)** Comparison of phosphosite and conserved motif of S6Ks and RPS6s in Pi, Sc, At, and Hs (Pi, *Phytophthora infestans*; Hs, *Homo sapiens*; Sc, *Saccharomyces cerevisiae*; At, *Arabidopsis thaliana*; St, *Solanum tuberosum*).

The TOR signaling pathway is a highly conserved pathway in eukaryotes. Previous studies have analyzed this pathway in various species, especially *Phytophthora* (Judelson and Ah-Fong, 2010; Van Dam et al., 2011; Tatebe and Shiozaki, 2017). Here, based on previous studies, we further supplement the detailed analysis of this pathway in *P. infestans*. TOR kinase, a key component in this pathway, is a highly conserved Ser/Thr protein kinase in eukaryotes. In order to analyze the TOR kinase in *P. infestans*, the amino acid sequences of TOR kinases for *Homo sapiens (Hs), Saccharomyces cerevisiae* (*Sc*), *Arabidopsis thaliana* (*At*), and *Solanum tuberosum* (*St*) were used as bait to blast against the genome of *P. infestans*. PITG_15408 and PITG_12226 were the shared prey of these TOR kinase proteins (Figure 1A). *PITG_15408* and *PITG_12226* were therefore named *PiTOR1* and *PiTOR2*, respectively. *PiTOR1* contained 9,717 bp cds while *PiTOR2* contained 7,980 bp cds (Figure 1A). *PiTOR1* encoded 3,238 amino acid residues with 354 kDa molecular mass, while *PiTOR2* encoded 2,659 amino acid residues with 297 kDa molecular mass. Both the *PiTOR1* and *PiTOR2* gene sequences contained one exon and no introns. Interestingly, there is also no intron in the homology of TORs in *P. sojae* and *P. parasitica* (Supplementary Table 2A). *P. infestans, P. sojae*, and *P. parasitica* are the major phylogenetic clades of *Phytophthora*. This result indicates that no intron in TORs may be a feature of *Phytophthora.* Furthermore, compared with other species, the number of introns in various TORs were 59 in *Hs*, 0 in *Sc*, 55 in *At*, and 56 in *St* (Supplementary Table 2A). *P. infestans* and *S. cerevisiae* were highly similar in their number of introns. Moreover, the genome sequence of *P. infestans* is the largest and complex among the *chromalveolates*, and strikingly rich transposons exist in *P. infestans* (Haas et al., 2009). This complex genome and rich transposons may result in unusual processes such as exon repetition, scrambling, retroposition, and recombination, which may contribute to intron losses or gains (Xu et al., 2012). All of them may result in the absence or no presence of intron in TOR of *P. infestans.*

Target of rapamycin proteins consist of several conserved domains in diverse species, for example the FAT, FRB, kinase, and FATC domains (De Virgilio and Loewith, 2006). The kinase domain is the catalytic domain of TOR (Adami et al., 2007), and it is important for the functions of TORC1 and TORC2 (Zheng et al., 1995; Zhang et al., 2011). The alignment of PiTOR1 and PiTOR2 with TOR protein sequences from *Hs, Sc, At*, and *St* showed consistent domain arrays of FAT, FRB, kinase, and FATC domains ranging from N-terminal to C-terminal (Figures 1B,C). Furthermore, the alignment of PiTOR1 with various TORs showed higher identity in the FAT, FRB, kinase, and FATC domains than that in PiTOR2 (Figures 1B,C). Further analysis showed that the highest identical amino acid sequence (70% identity) was found in the kinase domain among various TORs (Figures 1B–D). Evolutionary relationship analysis indicated that PiTOR was phylogenetically related to plant TOR (Figure 1E). Kinase domain alignment and evolutionary relationship analysis show that PiTOR is evolutionarily conserved.

Target of rapamycin proteins interact with other components to assemble into two complexes: TORC1 and TORC2 (Sandra et al., 2008). Besides the homology of TOR, the homologous proteins of other components of TORC1, namely RAPTOR and LST8, exist in *P. infestans* (Supplementary Tables 2B,C). We also found other putative homologous components of TORC2 such as RICTOR, but SIN1 was not found (Supplementary Tables 2B,C). These results indicate that a conserved and functional TORC1 but not TORC2 exists in *P. infestans*, named PiTORC1.

The LKB-AMPK pathway is one of the crucial upstream signaling pathways of TORC1 (Shaw et al., 2004). The corresponding homologous proteins of LKB and AMPK were present in *P. infestans* (Supplementary Tables 2B,C). The PI3K-PDK-Akt signaling pathway is another key upstream regulatory system of TORC1 (Dobrenel et al., 2016). PI3K, PDK, and Akt, the crucial components of this regulatory system, were also identified; however, the homology of other components in this regulatory system were not found in *P. infestans*, such as IRS (Supplementary Tables 2B,C).

Next, the downstream components of TORC1 were analyzed, such as S6K, RPS6, E2F3. As well-known phosphorylated TORC1 substrates, the S6K proteins of *Hs*, *At*, and *Sc* were used as templates to search for the homologous protein in *P. infestans*. PITG_18420 in *P. infestans* was homologous to various S6Ks in the species as mentioned above. Interestingly, PITG_18420 also contains the minimally required FxxFT/SYxx, which is the TORC1 substrate recognition motif in S6Ks (Xiong and Sheen, 2012) (Figure 1F), implying that PITG_18420 is the homology of various S6Ks (named PiS6K). RPS6 is the key S6K substrate. Based on the same analysis method, there was high homology between PITG_00443 and various RPS6s. Interestingly, as the minimal S6K substrate recognition motif in RPS6s (Asier et al., 2015), R/KxRxxS was also conserved in PITG_00443 (Figure 1G), which further suggests that PITG_00443 is the homology of various RPS6s (named PiRPS6). In addition, some of the putative downstream homologous components of TORC1, such as E2F3, were also found in *P. infestans* (Supplementary Tables 2B,C). These analyses suggest that the TOR signaling pathway is conserved in *P. infestans* (named the PiTOR signaling pathway).

### TOR Inhibitors Significantly Inhibit *P. infestans*

**FIGURE 2:**
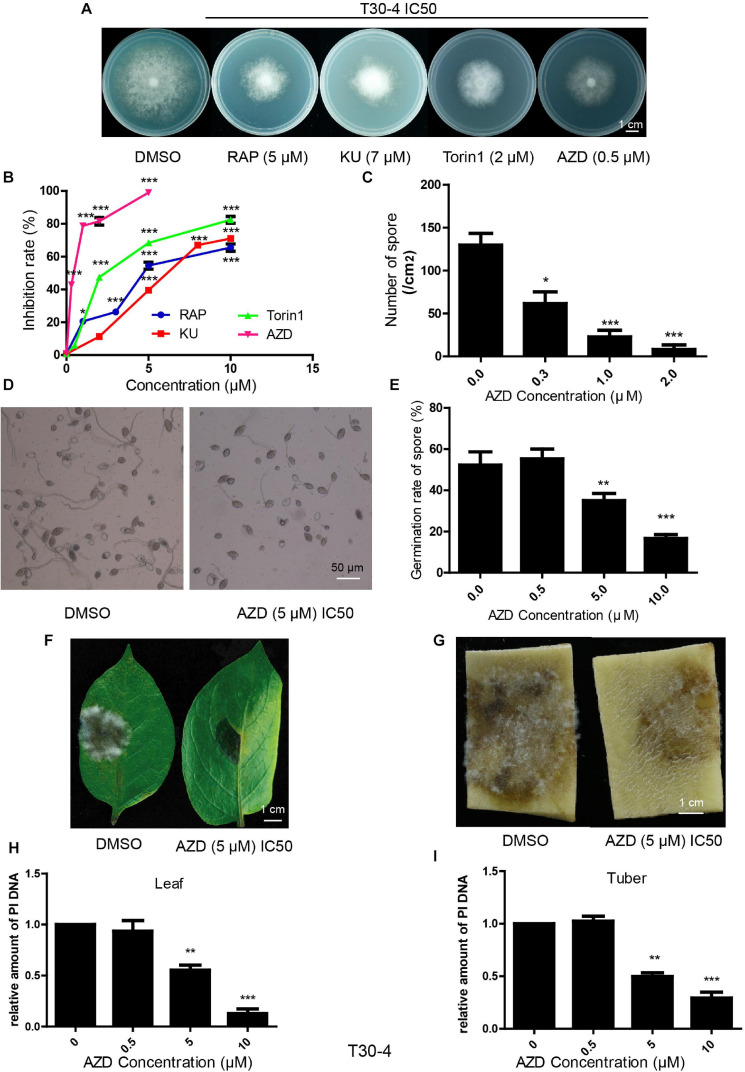
TOR inhibitors inhibited *P. infestans* (T30-4). **(A)** Mycelial phenotypes of T30-4 treated with IC50 of RAP, KU, Torin1, and AZD for 14 days. **(B)** Mycelia growth inhibition rate of T30-4 treated with different concentrations of four TOR inhibitors. **(C)** Inhibitory effects of sporulation capacity treated with different concentrations of AZD. **(D,E)** Spore germination phenotypes or rates of T30-4 treated with different concentrations of AZD. **(F,G)** Disease symptoms on leaves and tubers were captured after inoculation with the T30-4 strain for 4 days. The leaves and tubers were inoculated with spore solutions pretreated with different concentrations of AZD. **(H,I)** *P. infestans* DNA (T30-4) in tubers or leaves was quantified after being inoculated by spore solutions. Spore solutions were pretreated with different concentrations of AZD. Data was normalized to the *EF1* DNA levels of potato. (*P < 0.05, **P < 0.01, ***P < 0.001).

Bioinformatic analysis showed that the TOR signaling pathway was conserved in *P. infestans.* Here, we explored the inhibitory effects of TOR inhibitors on this pathogen. RAP, AZD, KU, and Torin1 are well-known TOR inhibitors. RAP is a first-generation TOR inhibitor and AZD, KU and Torin1 are regarded as second-generation TOR inhibitors. Assays of T30-4 and 002 *in vitro* showed that mycelial growth was significantly inhibited with an increasing concentration of RAP, KU, Torin1, and AZD (Figures 2A,B and Supplementary Figures 1A,B). Mycelial growth was sensitive to RAP, KU, Torin1, and AZD, with IC50 values for T30-4 of around 5, 7, 2, and 0.5 μM, respectively, and IC50 values for 002 of around 0.5, 4, 1, and 0.1 μM, respectively (Figures 2A,B and Supplementary Figures 1A,B). AZD displayed the strongest antiproliferative activity on mycelial growth due to it having the lowest IC50 among the four TOR inhibitors.

Besides mycelial growth, the inhibitory effects on sporulation capacity, spore germination and virulence were tested by the treatment of AZD (as the typical TOR inhibitor for its lowest IC50 on *P. infestans)*. These assays of T30-4 and 002 showed that sporulation capacity, spore germination and virulence were 50% inhibited (IC50) by AZD treatment with concentrations of roughly 0.3, 5, and 5 μM for T30-4 and 0.2, 2, and 2 μM for 002, respectively (Figures 2C–I and Supplementary Figures 1C–I). Thus, TOR inhibitors inhibit the mycelial growth, sporulation capacity, spore germination and virulence of *P. infestans.*

### Synergistic Effects of the Combination of First-Generation and Second-Generation TOR Inhibitors on *P. infestans*

#### Synergistic Effects of RAP + AZD, RAP + KU, and RAP + Torin1

**FIGURE 3:**
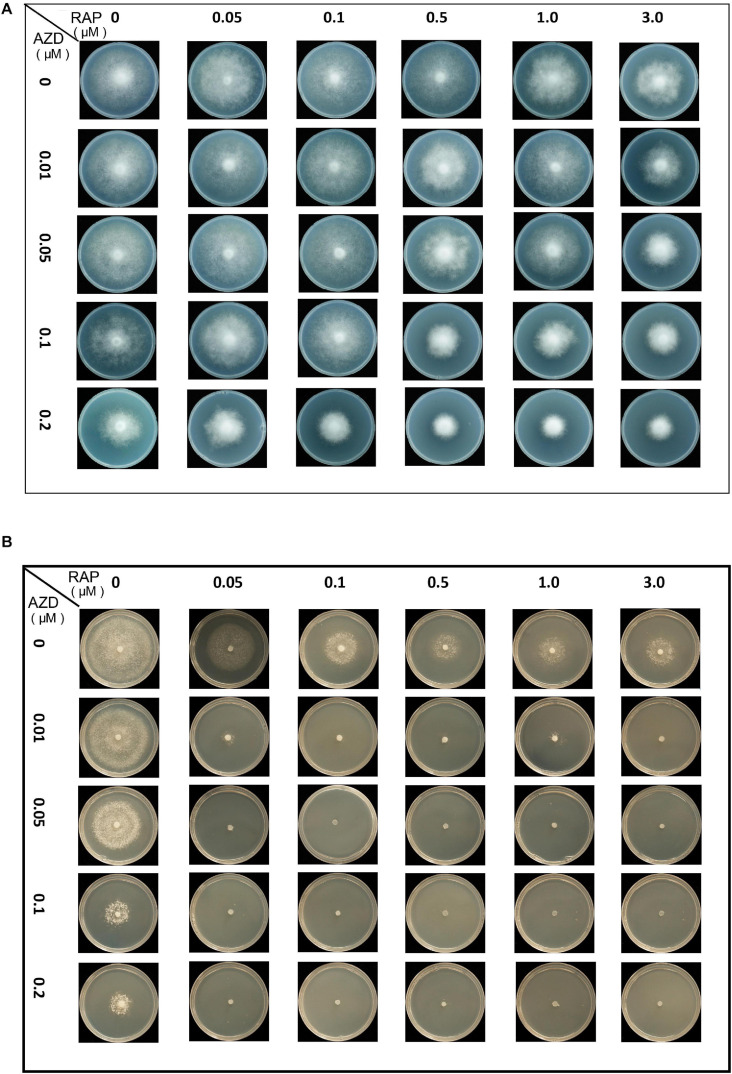
Synergistic growth inhibition of *P. infestans* treated by co-application with RAP and AZD. Colony morphology of **(A)** T30-4 and **(B)** 002 treated by co-application with RAP and AZD.

**FIGURE 4:**
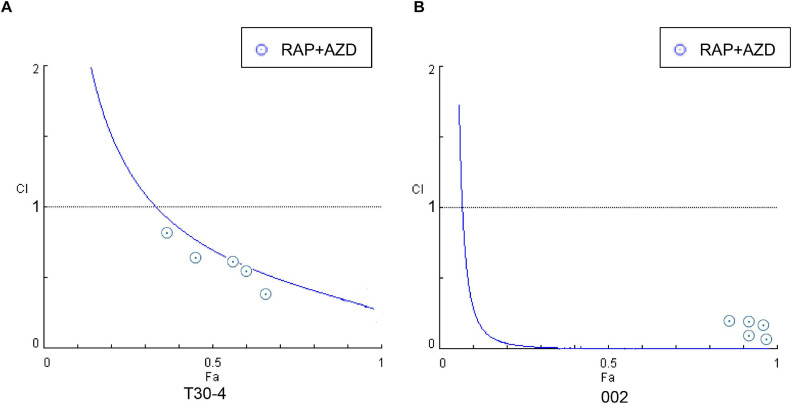
Fa-CI curves were generated by the treatment of *P. infestans* with RAP + AZD **(A)** T30-4 and **(B)** 002. CI < 1: Synergistic effect; CI = 1: Additive effect; CI > 1: Antagonistic effect. Fa (affected fraction): a function of the effect level.

First, the synergistic effects of RAP + AZD were tested. The mycelial growth assay of the two strains T30-4 and 002 showed that the combination of 0.5–3 μM RAP and 0.05–0.2 μM AZD on T30-4, and 0.05–3 μM RAP and 0.01–0.2 μM AZD on 002, resulted in smaller colony diameters than after single treatment (Figures 3A,B and Supplementary Figures 2A,B). For example, for T30-4, the inhibition rate after single treatment (0.5 μM RAP or 0.1 μM AZD alone) was around 10% or 25%, while the inhibition rate of combined treatment (0.5 μM RAP + 0.1 μM AZD) reached 40% (Figure 3A and Supplementary Figure 2A); for 002, the inhibition rate after single treatment (0.05 μM RAP or 0.01 μM AZD alone) was smaller than that after combined treatment (0.05 μM RAP + 0.01 μM AZD) (Figure 3B and Supplementary Figure 2B). These findings show that the decreased mycelial growth was more significant under combined treatment. Furthermore, the IC50 value of RAP + AZD (T30-4: 0.5 μM + 0.1 μM; 002: 0.05 μM + 0.005 μM) was much lower than that of single treatment with either RAP (T30-4: 5 μM; 002: 0.5 μM) or AZD (T30-4: 0.5 μM; 002: 0.1 μM) (Table 1), suggesting that the combination of RAP and AZD markedly reduced the effective dosages of both agents (T30-4: 10-fold reduction in RAP and 5-fold reduction in AZD; 002: 10-fold reduction in RAP and 20-fold reduction in AZD). This suggests that the combination of RAP and AZD results in a synergistic inhibition of mycelial growth in T30-4 and 002, which was confirmed by CI < 1 (synergism effect) (Figures 4A,B). Interestingly, the same synergistic inhibitory effects were also observed under RAP + KU or RAP + Torin1 (Supplementary Tables 3, 4).

**TABLE 1:** IC50 values of TOR inhibitors on *P. infestans* T30-4 and 002.

IC50 Strain	RAP (μ M)	AZD (μ M)	KU (μ M)	Torin1 (μ M)	RAP + AZD (μ M)	RAP + KU (μ M)	RAP + Torin1 (μ M)
T30-4	5	0.5	7	2	0.5 + 0.1	0.5 + 2	0.5 + 0.3
002	0.5	0.1	4	1	0.05 + 0.005	0.05 + 0.5	0.05 + 0.05

#### Comparison of RAP + AZD, RAP + KU, and RAP + Torin1

Collectively, our observations demonstrate that co-applying first-generation and second-generation TOR inhibitors synergistically inhibit *P. infestans.* Although the three drug combinations exhibited similar synergistic inhibition effects on *P. infestans*, it remained unknown which team was the most effective in controlling this pathogen. To this end, the synergistic inhibition effects among these three combinations were compared. For T30-4, the IC50 values for RAP + KU, RAP + Torin1 and RAP + AZD were 0.5 μM + 2 μM, 0.5 μM + 0.3 μM and 0.5 μM + 0.1 μM, respectively (Table 1). Under the same inhibition rate (50%) and the same RAP concentration (0.5 μM), the relationships of concentrations among the three other drugs were: AZD (0.1 μM) < Torin1 (0.3 μM) < KU (2 μM) (Table 1). For 002, the IC50 values for RAP + KU, RAP + Torin1 and RAP + AZD were 0.05 μM + 0.5 μM, 0.05 μM + 0.05 μM and 0.05 μM + 0.005 μM, respectively (Table 1). Under the conditions of a 50% inhibition rate and the same RAP concentration (0.05 μM), the relationships were still AZD (0.005 μM) < Torin1 (0.05 μM) < KU (0.5 μM) (Table 1). These results indicate that RAP + AZD is the best team due to its lowest concentration for the same inhibitory effects on *P. infestans.*

### Transcriptome Analysis of Synergistic Effects on *P. infestans*

Molecular-level analysis is regarded as an effective method for attaining a more comprehensive assessment of drug synergistic effects. Therefore, to explain the synergistic anti-oomycete actions of first-generation and second-generation TOR inhibitors on *P. infestans*, the transcriptome data was analyzed in detail. Because RAP + AZD showed the best inhibitory effects on *P. infestans*, this drug team was used as the representative for transcriptome analysis.

#### Synergistic Effects on Total DEGs

**FIGURE 5:**
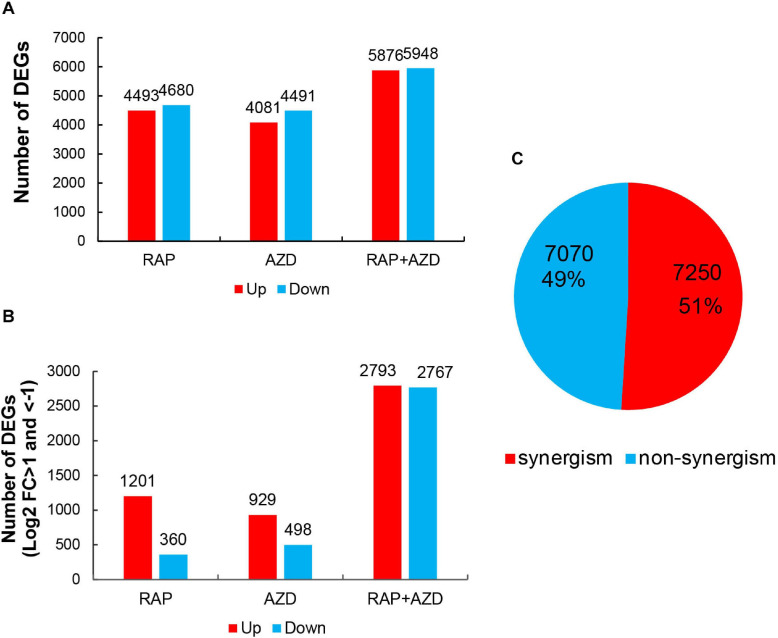
DEGs in RAP, AZD and RAP + AZD-treated samples. **(A)** DEGs with Log2 foldchange >0 or <0 in the three samples. Red: up-regulated DEGs (Log2 foldchange > 0). Blue: down-regulated DEGs (Log2 foldchange < 0). **(B)** DEGs with Log2 foldchange >1 or <–1. **(C)** Synergism and non-synergism of DEGs. [Synergistic DEGs: | gene expression of (RAP + AZD)| > | gene expression of (RAP/AZD alone)|].

The T30-4 strain was treated with DMSO, RAP, AZD, and RAP + AZD for transcriptome assay. The transcriptome data showed that when RAP and AZD were combined, there was a more significant increase in the amount of up-regulated or down-regulated DEGs (Figures 5A,B). For example, under the condition of DEGs with Log2 fold change >0 or <0, relationships among down-regulated DEGs: RAP + AZD (5,948 DEGs) > RAP (4,680 DEGs) > AZD (4,491 DEGs); relationships among up-regulated DEGs: RAP + AZD (5,876 DEGs) > RAP (4,493 DEGs) > AZD (4,081 DEGs) (Figure 5A and Supplementary Tables 5A–C). The same trends were also significantly observed in DEGs with Log2 fold change >1 or <−1 (Figure 5B and Supplementary Tables 5D–F). Importantly, in the RAP, AZD, and RAP + AZD-treated samples, 7,250 DEGs (51%) of a total of 14,320 DEGs showed synergistic effects on gene expression (Figure 5C and Supplementary Tables 6A,B). These results suggest that, compared with single treatment, combined treatment not only increases the number of up-regulated and down-regulated DEGs, but also induces synergistic effects on gene expression in DEGs.

#### Synergistic Effects on GO Terms

**FIGURE 6:**
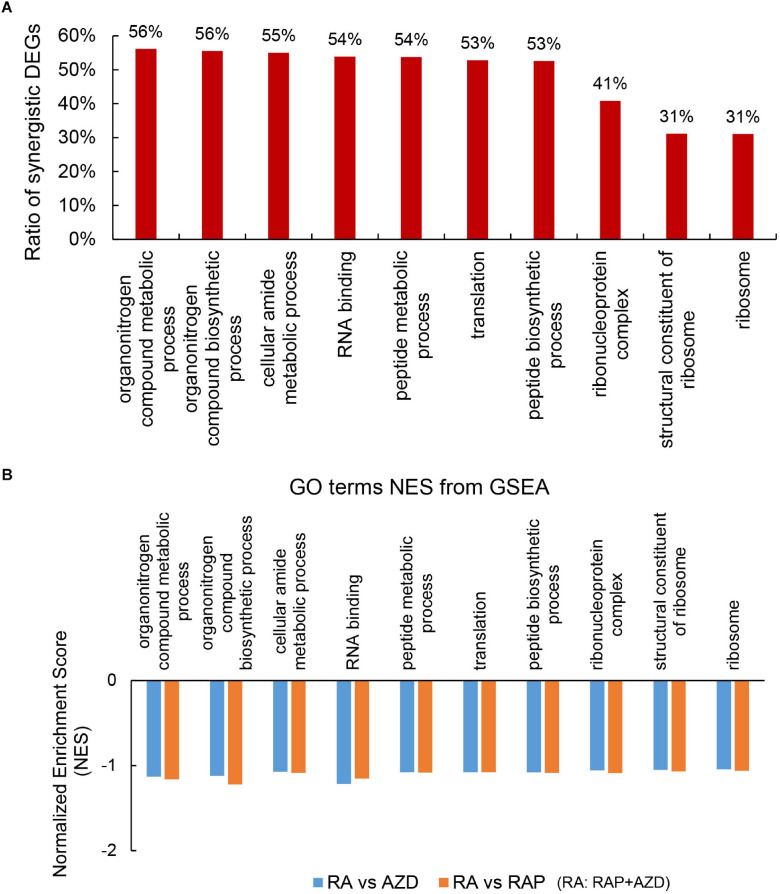
Synergistic DEGs and gene expression trends in 10 co-existing GO terms after treatment with RAP + AZD. **(A)** Ratio of synergistic DEGs in the 10 co-existing GO terms. Ratio of synergistic DEGs = synergistic DEGs/total DEGs. **(B)** GSEA analyses of genesets for 10 co-existing GO terms. NES, normalized enrichment score. FDR, false discovery rate. Negative (–) and Positive (+) NES indicate down-regulated and up-regulated expression in RAP + AZD, respectively. All the FDR of 10 co-existing GO terms < 0.25.

To explore the detailed synergistic effects of RAP, AZD, and RAP + AZD on *P. infestans*, the GO terms were evaluated. Among the top 30 GO terms, there were 22 co-existing GO terms in the three datasets of RAP, AZD, and RAP + AZD (Supplementary Tables 7A–D). 10 of the 22 co-existing GO terms play important role in various biological functions (Figure 6A and Supplementary Table 8A); especially, more than 50% of DEGs showed synergistic effects on gene expression in the 7 of 10 co-existing GO terms (Figure 6A and Supplementary Tables 8A–K). Therefore, the combination of RAP and AZD induces significantly synergistic effects on importantly biological functions.

Further GSEA analyses showed that RAP + AZD-treated sample exhibited lower gene expression than RAP or AZD-treated samples alone in 10 co-existing GO terms (Figure 6B and Supplementary Data Sheets 1, 2). For example, peptide metabolic process, peptide biosynthetic process and translation (Figure 6B and Supplementary Data Sheets 1, 2). This indicates that the co-application of RAP and AZD enhances the inhibitory effects on importantly biological functions of *P. infestans* compared to those of the RAP-treated or AZD-treated samples alone.

#### Synergistic Effects on KEGG Pathways

**FIGURE 7:**
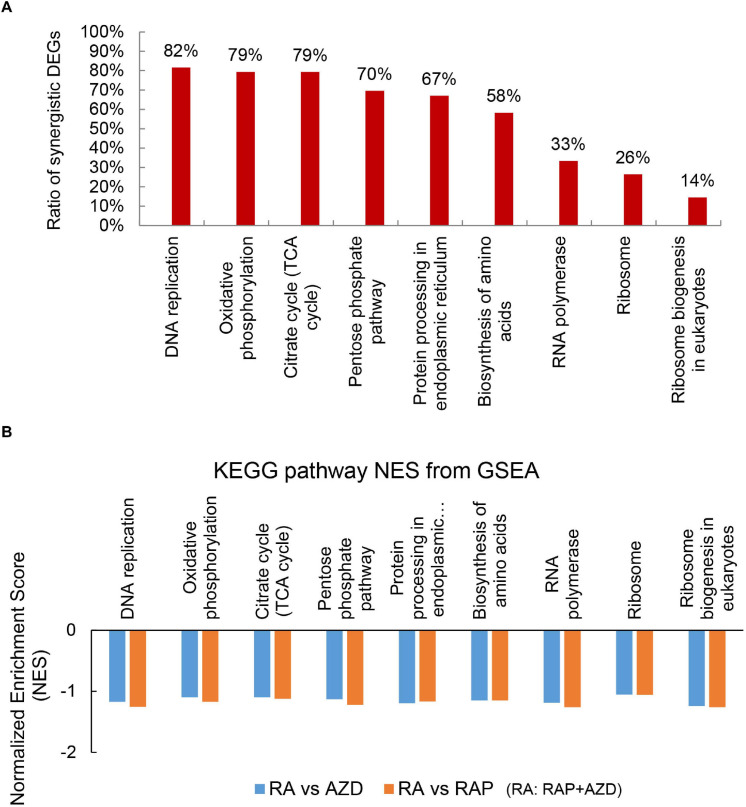
Synergistic DEGs and gene expression trends in nine co-existing KEGG pathways after treatment with RAP + AZD. **(A)** Ratio of synergistic DEGs in the nine co-existing KEGG pathways. Ratio of synergistic DEGs = synergistic DEGs/total DEGs. **(B)** GSEA analyses of genesets for nine co-existing KEGG pathways. NES, normalized enrichment score. FDR, false discovery rate. Negative (–) and Positive (+) NES indicate down-regulated and up-regulated expression in RAP + AZD, respectively. All the FDR of nine co-existing KEGG pathways < 0.25.

KEGG pathways were also analyzed to investigate the detailed synergistic effects of RAP + AZD on *P. infestans.* Among the top 30 KEGG pathways, 14 co-existed across the three datasets (RAP, AZD and RAP + AZD) (Supplementary Tables 9A–D). Nine of 14 co-existing pathways were related to importantly biological process (Figure 7A and Supplementary Table 10A). An analysis of the synergistic DEGs showed that more than 50% were synergistic DEGs in six of the nine co-existing KEGG pathways (Figure 7A and Supplementary Tables 10A–J). The top three synergistic DEGs at 82%, 79%, and 79% were observed in the KEGG pathways of DNA replication, citrate cycle and oxidative phosphorylation, respectively (Figure 7A and Supplementary Tables 10A,C–E). Thus, combined treatment showed significantly greater synergistic effects on the gene expression of DEGs in important processes compared with those of single treatment.

Through the further GSEA analyses, it was found that the obvious downregulation of nine co-existing KEGG pathways were generated after co-applying RAP and AZD, compared with RAP or AZD alone (Figure 7B and Supplementary Data Sheets 3, 4). For example, citrate cycle and oxidative phosphorylation (Figure 7B and Supplementary Data Sheets 3, 4). Thus, an analysis of the KEGG pathways revealed that the co-application of RAP and AZD enhances the inhibitory effects on important processes in *P. infestans.* These results are consistent with the analysis of GO terms.

#### Synergistic Effects on TOR Signaling Pathways

**FIGURE 8:**
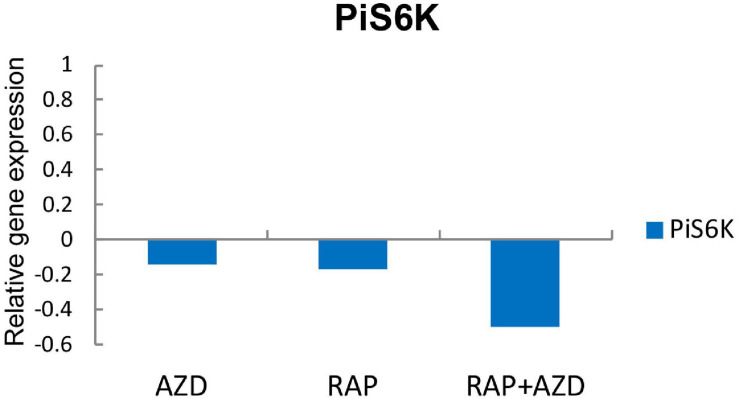
Gene expression of PiS6K in RAP, AZD and RAP + AZD.

Because TOR inhibitors target the TOR signaling pathway, we next explored the influence of RAP, AZD and RAP + AZD on the TOR signaling pathway of *P. infestans.* S6K is the direct substrate of TOR kinase protein (Nave et al., 1999). The expression of gene encoding PiS6K (homology of S6K) was more dramatically suppressed by drug combination than single treatment (Figure 8 and Supplementary Table 11), indicating that RAP + AZD enhances the inhibitory effects on TOR kinase protein.

**FIGURE 9:**
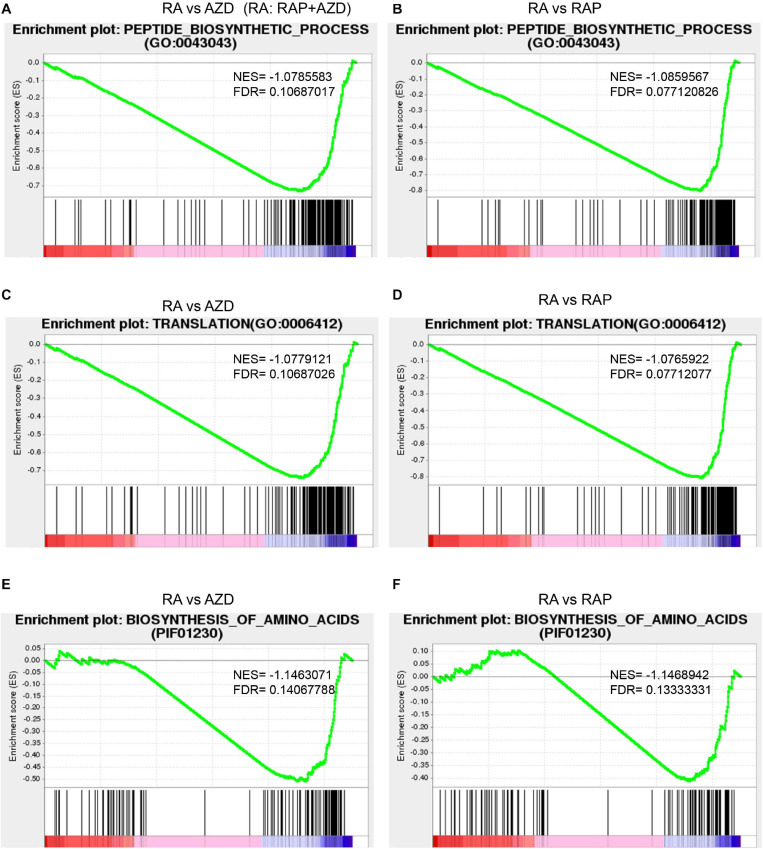
Synergistic effects of RAP + AZD on downstream functions and processes of TOR signaling pathways. The detailed GSEA analyses of genesets in RAP + AZD vs RAP, RAP + AZD vs AZD for the peptide biosynthetic process (**A** and **B**), translation (**C** and **D**), and the biosynthesis of amino acids (**E** and **F**). NES, normalized enrichment score. FDR, false discovery rate. Negative (–) and Positive (+) NES indicate down-regulated and up-regulated expression in RAP + AZD, respectively.

Target of rapamycin kinase protein controls cell growth by activating various downstream biological functions such as translation, ribosome biogenesis, protein biogenesis and so on (De Virgilio and Loewith, 2006; Wullschleger et al., 2006; Laplante and Sabatini, 2012). Next, the downstream functions of the TOR signaling pathway were analyzed. Among the top 30 GO terms, there were 5 related to translation, ribosome biogenesis and protein biogenesis: translation, peptide biosynthetic process, ribonucleoprotein complex, structural constituent of ribosome and ribosome (Figure 6A and Supplementary Table 8A). 53%, 53%, 41%, 31%, and 31% of DEGs were synergistic genes in translation, peptide biosynthetic process, ribonucleoprotein complex, structural constituent of ribosome and ribosome, respectively (Figure 6A and Supplementary Tables 8B,C,G,I,J). The top one ratio of synergistic DEGs existed in translation and the peptide biosynthetic process (Figure 6A and Supplementary Figures 8S,U), indicating that drug combination mainly synergistically targeted translation and the peptide biosynthetic process. Additionally, GSEA analysis indicated that the gene expression in translation and the peptide biosynthetic process were lower in the sample of RAP + AZD than that of RAP or AZD alone (Figures 9A–D and Supplementary Data Sheets 1, 2). Interestingly, 8 of the top 10 down-regulated synergistic DEGs in the translation and peptide biosynthetic process were elongation factors and translation initiation factors, indicating that combination of two drugs may mainly inhibit these factors (Supplementary Tables 8G,I). Collectively, these results suggest that the combination of RAP and AZD prominently synergistic inhibits translation and the peptide biosynthetic process compared with other downstream functions of the TOR signaling pathway. These results were consistent with the phenotypes of *Saccharomyces cerevisiae*: translation initiation and the protein biosynthesis of *Saccharomyces cerevisiae* were inhibited by rapamycin (Crespo and Hall, 2002; De Virgilio and Loewith, 2006).

As a further analysis of the KEGG pathways showed, among the top 30 KEGG pathways, 3 were involved in ribosome biogenesis and protein biogenesis: biosynthesis of amino acids, ribosome and ribosome biogenesis in eukaryotes (Figure 7A and Supplementary Table 10A). Among the three, the highest ratio of synergistic DEGs was in the biosynthesis of amino acids (58%) (Figure 7A and Supplementary Table 10B). Moreover, the GSEA indicated that RAP + AZD was more likely to inhibit gene expression of the biosynthesis of amino acids, compared with RAP or AZD alone (Figures 9E,F and Supplementary Data Sheets 3, 4). Particular, dihydroxy-acid dehydratase and ketol-acid reductoisomerase, the key enzymes of branched chain amino acid synthesis (Singh and Shaner, 1995), were the top 1 and 2 down-regulated synergistic DEGs, respectively, in the biosynthesis of amino acids (Supplementary Table 10B). These results suggest that, on the level of the KEGG pathways, drug combination mainly synergistically targets the biosynthesis of amino acids and tends to synergistically suppress the biosynthesis of amino acids. Interestingly, the previous transcriptome and phosphoproteomics also showed that rapamycin disrupted the amino acid metabolism of *Saccharomyces cerevisiae* (Oliveira et al., 2015; Dikicioglu et al., 2018), which were consistent with our analysis.

The top one ratio of synergistic DEGs existed in the GO terms of translation and the peptide biosynthetic process, and the KEGG pathway of the biosynthesis of amino acids. All of these are involved in protein biosynthesis. Thus, drug combination mainly synergistically targets protein biosynthesis. Collectively, the combination of RAP and AZD synergistically inhibits the substrate of TOR kinase protein, PiS6K, resulting in the synergistic suppression of the downstream protein biosynthesis function of the TOR signaling pathway.

In summary, an integration of the analysis of GO terms, KEGG pathways and the TOR signaling pathway indicates that the synergistic inhibitory effects on the various biological functions, pathways and TOR signaling pathway achieved through drug combination may contribute to enhanced inhibitory action on the growth and development of *P. infestans.*

#### Synergistic Effects on Pathogenicity-Related Genes

Several studies show that TOR signaling pathway controls pathogenicity of pathogen (Yu et al., 2014; Li et al., 2019). Secreted RXLR effector protein, the important pathogenicity-related protein, plays an important role in pathogenicity (Whisson et al., 2007; Boutemy et al., 2011). Thus, we employed transcriptomics to analyze the change of RXLR effector after treated with TOR inhibitors. The analyses of transcriptomics showed that RAP + AZD enhanced the gene expressions of 47 RXLR effector, compared with single drug (Supplementary Table 12); the expression of these effectors was modified, up-regulating 8 and down-regulating 39 of them. This result indicates that combination of two drugs enhance the inhibitory effect on gene expression of RXLR effectors (Supplementary Table 12). Therefore, co-application of AZD and rapamycin synergistic inhibited pathogenicity-related genes in *P. infestans*, which was consistent with the previous studies that TOR signaling pathway regulates pathogenicity.

#### qPCR

In order to confirm these transcriptome-based analyses of synergistic effects, 7 DEGs related to translation, peptide biosynthetic process and ribosome were selected for RT-qPCR, such as eukaryotic translation initiation factor 3, eukaryotic translation initiation factor 2, elongation factor 1-alpha, lysyl-tRNA synthetase, 50S ribosomal protein L4, selenocysteine-specific elongation factor, protein kinase (Supplementary Figure 3 and Supplementary Table 1). The results of RT-qPCR were highly consistent with the transcriptome data, indicating that the latter was reliable and valid (Supplementary Figure 3).

## Discussion

In this study, we explore a novel control method and strategy on *P. infestans.* The results show that the TOR signaling pathway may exist in *P. infestans*, and TOR inhibitors significantly inhibit *P. infestans.* Importantly, synergistic anti-oomycete effects on *P. infestans* by combining both RAP and AZD/KU/Torin1 treatments were significant, especially the combination of RAP and AZD. This was proven by the synergistic inhibition of genes, functions, KEGG pathways and TOR signaling pathway. To the best of our knowledge, this study is the first to reveal that RAP and AZD/KU/Torin1 jointly combat the pathogenic oomycete *P. infestans*. These findings have implications for designing a novel drug based on the co-application of TOR inhibitors to combat pathogenic oomycetes in plants, animals and humans.

Tatebe also analyzed the homologous genes of the TOR signaling pathway in various species (Tatebe and Shiozaki, 2017). The major difference between Supplementary Table 2C and Tatebe’s analysis is that the focused upstreams of the TOR signaling pathways are different. Our research focused on the LKB-AMPK and PI3K-PDK-Akt pathways (Wullschleger et al., 2006), while Tatebe paid attention to the GATOR1-RAG pathway, and this resulted in some homologous genes only existing in either Supplementary Table 2C or Tatebe’s Table; for example, some components of the GATOR1-RAG pathway, including RAG-A/B, RAG-C/D, DEPDC5, NPRL2, and NPRL3, were only shown in Tatebe’s research, whereas IRS, PI3K, PDK, AKT, and PTEN belonging to the PI3K-PDK-Akt pathway, were only shown in our research. Besides, some downstream homologs of the TOR signaling pathway were analyzed in our research, such as S6K and RPS6, that Tatebe did not focus on.

FKBP12 bridges the interaction between the FRB domain of TOR and rapamycin, leading to the inhibition of rapamycin on TORC1. However, due to the lack of FKBP12 in plants, various plants including *Vicia faba*, lotus, tobacco, rice, millet, and *Arabidopsis* (in solid medium with Suc) are not sensitive to rapamycin even at high concentrations (20 μM) (10 μM inhibited *Arabidopsis* in liquid medium with Glc) (Ren et al., 2012; Xiong and Sheen, 2012; Deng et al., 2017). Sometimes the concentration of rapamycin has to be as high as 100 μM to treat rice (Vleesschauwer et al., 2018), which is much higher than 5 or 0.5 μM in our research. Therefore, to explore the TOR pathway in plants, rapamycin hypersensitive plants were usually produced by introducing yeast FKBP12 into plants. According to the published data of our laboratory (Deng et al., 2017), potato plants also showed insignificant growth inhibition under rapamycin treatment at high concentrations (reaching 20 μM); this suggests that rapamycin used in our research could be harmless to potato plants. Furthermore, the IC50 value of AZD on the growth of potato explants reached 2 μM (Deng et al., 2017), which is much higher than 0.5 or 0.1 μM AZD (IC50) on mycelial growth of T30-4 and 002, respectively. In particular, the combination of AZD and rapamycin allowed a significant reduction in the dosage of AZD, leading to a further reduction in the harm caused to plants by AZD.

The TOR signaling pathway is a highly conserved pathway that regulates cell growth, development and metabolism. S6K is the direct substrate of the TOR kinase protein. The current study shows that, compared with a single agent, the combination of RAP and AZD enhances the inhibitory effects on the gene expression of S6K. It is consistent with previous study that rapamycin and pemetrexed synergistically inhibit S6K through the TOR signaling pathway in NSCLC cells (Kawabata et al., 2014). This result indicates that the combination of the TOR inhibitors may synergistically inhibit the TOR pathway and enhance control of the physiological functions of *P. infestans.*

The regulation of translation, protein biosynthesis and ribosome biogenesis are known to be the primary functions of the TOR (Wullschleger et al., 2006), and the suppression of the TOR pathway results in the inhibition of these downstream functions (Wullschleger et al., 2006). The analysis of GO terms indicates that the combination of RAP and AZD prominently synergistically inhibits translation and the peptide biosynthetic process; meanwhile, the KEGG pathways reveal that the co-application of RAP and AZD mainly has a cooperative inhibitory effect on the biosynthesis of amino acids, all of which participate in protein biosynthesis. These results suggest that drug combination mainly exhibits co-suppression of protein biosynthesis in *P. infestans*, which is one of the primary downstream functions of the TOR signaling pathway.

As found in the detailed analysis, in the GO terms of the translation and peptide biosynthetic process, elongation factors and translation initiation factors were prominently synergistically inhibited by two drugs, both of which play important roles in the two biological processes (Riis et al., 1990; Xia et al., 2010). This suggests that the co-application of these two drugs may mainly target elongation factors and translation initiation factors to synergistically inhibit the translation and peptide biosynthetic process. Meanwhile, in the KEGG pathway of the biosynthesis of amino acids, dihydroxy-acid dehydratase and ketol-acid reductoisomerase were the top 1 and 2 down-regulated synergistic DEGs, respectively, in this pathway. Both enzymes are necessary for branched chain amino acid synthesis (Singh and Shaner, 1995). This suggests that the inhibition of branched chain amino acid synthesis by these two drugs may play a key role in the regulation of amino acids biosynthesis. Thus, the elongation factors, translation initiation factors and key enzymes of branched chain amino acid synthesis may mainly contribute to the synergistically inhibitory effect on protein synthesis by the co-application of rapamycin and AZD.

The inhibition of protein synthesis can influence various biological functions of pathogens. Kasugamycin inhibits the protein synthesis of *Pyricularia oryzae* to prevent rice blast (Okuyama et al., 1971; Schuwirth et al., 2006); oxytetracycline exhibits antibacterial activities by suppressing the protein synthesis of various pathogens, including *Staphylococcus aureus*, *Streptococcus pneumoniae*, *Streptococcus pyogenes*, and *Neisseria gonorrhoeae* (Streltsov et al., 1975). Thus, the combination of rapamycin and AZD may enhance the inhibition of protein synthesis to control *P. infestans.* According to the top 30 GO terms and KEGG pathways, besides the direct downstream functions or processes of the TOR signaling pathway, other indirect functions or pathways were also synergistically inhibited by drug combination; for example, the GO terms of RNA binding, organonitrogen compound metabolic process etc., and the KEGG pathways of the citrate cycle, DNA replication, protein processing in the endoplasmic reticulum, etc. These results indicate that besides the direct downstream functions of the TOR signaling pathway, the indirect functions of the pathway are also synergistically targeted by drug combination, and they all contribute to the synergistically inhibitory effects of RAP and AZD on *P. infestans.*

Single-targeted agents usually exhibit limited efficacy and serious drug resistance, making them perform poorly in controlling pathogens. Since it is very difficult for organisms to compensate for the effects of multiple sites being targeted by multiple drugs simultaneously, multi-component therapies or multi-targeted agents based on synergism are regarded as effective methods; such treatment has increased efficacy and makes drug resistance less likely (Zimmermann et al., 2007). Drug synergy has been widely applied in clinical medicine and pesticides. For example, the co-application of penicillin and gentamicin exhibits synergistic effects on enterococcal endocarditis (Watanakunakorn, 1972). Based on the co-application of first and second-generation TOR inhibitors, third-generation TOR inhibitors effectively delay drug-resistance in tumors (Rodrik-Outmezguine et al., 2016). The principle of “third-generation TOR inhibitors” has been also applied in our research. The co-application of RAP and AZD targets different domains of TOR kinase protein in *P. infestans*, which causes the simultaneously enhanced suppression of TOR kinase protein and leads to enhanced inhibitory effects on downstream functions, especially protein biosynthesis; this results in the synergistic suppression of *P. infestans* with the possibility of delaying drug resistance.

This research clarifies that TOR kinase protein is an important target for controlling *P. infestans.* Importantly, we have proven the synergistic inhibitory effect of first-generation and second-generation TOR inhibitors on *P. infestans*, providing a new insight for taking corresponding prevention and control measures regarding oomycete diseases. However, the TOR inhibitors are not suitable for popularization and application due to their high costs. Thus, in the future, we will focus on three aspects. First, we need to demonstrate that the rapamycin and/or ATP-competitive inhibitors of TOR do inhibit TOR kinase activity in *P. infestans* (e.g., phosphorylation level of S6K and RPS6). Second, we will focus on searching for a cheap, efficient, and safe TOR-targeted drug instead of the TOR inhibitors. Finally, we will explore the synergistic effects of the TOR inhibitors with other chemical or biological oomyceticides to reduce the dosage and enhance the efficacy of oomyceticides.

## Data Availability Statement

The datasets presented in this study can be found in online repositories. The names of the repository/repositories and accession number(s) can be found in the article/Supplementary Material.

## Author Contributions

MR and SZ contributed to the conceptualization. JZ contributed to the methodology. AK contributed to the software. SZ and JZ contributed to the validation. FX contributed to the formal analysis. MR, DG, and SZ contributed to the data curation. SZ contributed to the writing—original draft preparation. MR and SZ contributed to the writing—review and editing. All authors have read and agreed to the published version of the manuscript.

## Conflict of Interest

The authors declare that the research was conducted in the absence of any commercial or financial relationships that could be construed as a potential conflict of interest.

## Supplementary Material

Supplementary Figure 1 TOR inhibitors inhibited *P. infestans* (002). **(A)** Mycelial phenotypes of 002 treated with IC50 of RAP, KU, Torin1, and AZD for 14 days. TOR inhibitors: Rapamycin (RAP), AZD8055 (AZD), Torin1, and KU. **(B)** Mycelia growth inhibition rate of 002 treated with different concentrations of four TOR inhibitors. **(C)** Inhibitory effects on the sporulation capacity of 002 treated with different concentrations of AZD. **(D,E)** Spore germination phenotypes or rates of 002 treated with different concentrations of AZD. **(F,G)** Disease symptoms on leaves and tubers were captured after inoculation with the 002 strain for 4 days. The leaves and tubers were inoculated with spore solutions pretreated with different concentrations of AZD. **(H)** and **(I)** Relative amounts of *P. infestans* DNA (002) in leaves and tubers after being inoculated by spore solutions. Spore solutions were pretreated with different concentrations of AZD. The data was normalized to the *EF1* DNA levels of potato. (^∗^*P* < 0.05, ^∗∗^*P* < 0.01, ^∗∗∗^*P* < 0.001).

Supplementary Figure 2 Synergistic growth inhibition rate of *P. infestans* treated by co-application with RAP and AZD. **(A)** T30-4. **(B)** 002. (Lower case letters indicate significant difference, *p* < 0.05).

Supplementary Figure 3 Relative expression levels of seven synergistic DEGs both in transcriptome and RT-qPCR. 1. PITG_12483, eukaryotic translation initiation factor 3, putative; 2. PITG_07885, eukaryotic translation initiation factor 2, subunit alpha; 3. PITG_22657, elongation factor 1-alpha; 4. PITG_08714, Lysyl-tRNA synthetase; 5. PITG_03768, 50S ribosomal protein L4; 6. PITG_17009, selenocysteine-specific elongation factor; 7. PITG_18420, protein kinase.

Supplementary Data Sheets 1, 2 The detailed GSEA analyses of GO terms (RAP + AZD vs. RAP, RAP + AZD vs. AZD). 1. Cellular amide metabolic process; 2. Organonitrogen compound biosynthetic process; 3. Organonitrogen compound metabolic process; 4. Peptide biosynthetic process; 5. Peptide metabolic process; 6. Ribonucleoprotein complex; 7. Ribosome; 8. RNA binding; 9. Structural constituent of ribosome; 10. Translation.

Supplementary Data Sheets 3, 4 The detailed GSEA analyses of KEGG pathways (RAP + AZD vs. RAP, RAP + AZD vs. AZD). 1. Biosynthesis of amino acids; 2. Citrate cycle (TCA cycle); 3. DNA replication; 4. Oxidative phosphorylation; 5. Pentose phosphate pathway; 6. Protein processing in endoplasmic reticulum; 7. Ribosome; 8. Ribosome biogenesis in eukaryotes; 9. RNA polymerase.

Supplementary Table 1 **(A)** Primer sequence of qPCR. **(B)** Combination of RAP and AZD. **(C)** Combination of RAP and KU. **(D)** Combination of RAP and Torin1.

Supplementary Table 2 **(A)** Intron in TORs of various species. **(B)** Putative homologous components of the TOR signaling pathway in *P. infestans* in detail. **(C)** Putative homologous components of the TOR signaling pathway in *P. infestans* (Concise edition).

Supplementary Table 3 IC50 of RAP, KU and RAP + KU for *P. infestans* T30-4 and 002.

Supplementary Table 4 IC50 of RAP, Torin1 and RAP + Torin1 for *P. infestans* T30-4 and 002.

Supplementary Table 5 DEGs in RAP, AZD and RAP + AZD samples, respectively. DEGs with Log2 fold change >0 or <0 in **(A)** RAP, **(B)** AZD and **(C)** RAP + AZD-treated samples. DEGs with Log2 fold change >1 or <−1 in **(D)** RAP, **(E)** AZD and **(F)** RAP + AZD-treated samples.

Supplementary Table 6 Total DEGs and synergistic DEGs. **(A)** Total DEGs in three samples. **(B)** Synergistic DEGs in RAP + AZD.

Supplementary Table 7 Top 30 GO terms in **(A)** RAP, **(B)** AZD and **(C)** RAP + AZD samples. **(D)** 22 co-existing GO terms in RAP, AZD and RAP + AZD samples.

Supplementary Table 8 Synergistic DEGs in 10 co-existing GO terms. **(A)** Overall synergistic DEGs in the 10 co-existing GO terms. Synergistic DEGs in the GO terms of **(B)** ribonucleoprotein complex, **(C)** ribosome, **(D)** cellular amide metabolic process, **(E)** organonitrogen compound biosynthetic process, **(F)** organonitrogen compound metabolic process, **(G)** peptide biosynthetic process, **(H)** peptide metabolic process, **(I)** translation, **(J)** structural constituent of ribosome and **(K)** RNA binding.

Supplementary Table 9 Top 30 KEGG pathways in **(A)** RAP, **(B)** AZD and **(C)** RAP + AZD samples. **(D)** 14 co-existing KEGG pathways in RAP, AZD and RAP + AZD samples.

Supplementary Table 10 Synergistic DEGs in nine co-existing KEGG pathways. **(A)** Overall synergistic DEGs in the nine co-existing KEGG pathways. Synergistic DEGs in the KEGG pathways of **(B)** Biosynthesis of amino acids, **(C)** Citrate cycle (TCA cycle), **(D)** DNA replication, **(E)** Oxidative phosphorylation, **(F)** Pentose phosphate pathway, **(G)** Protein processing in endoplasmic reticulum, **(H)** Ribosome, **(I)** Ribosome biogenesis in eukaryotes, **(J)** RNA polymerase.

Supplementary Table 11 Synergistic effects on gene expression of PiS6K and PiRPS6 after treatment with RAP, AZD, and RAP + AZD.

Supplementary Table 12 Synergistic effects on pathogenicity-related genes.
