# Supplementary figures and images for: TOR Inhibitors Synergistically Suppress the Growth and Development of Phytophthora infestans, a Highly Destructive Pathogenic Oomycete
Source: Front Microbiol. 2021 Apr 16;12:596874. doi: 10.3389/fmicb.2021.596874 (PMC8086431; doi:10.3389/fmicb.2021.596874)

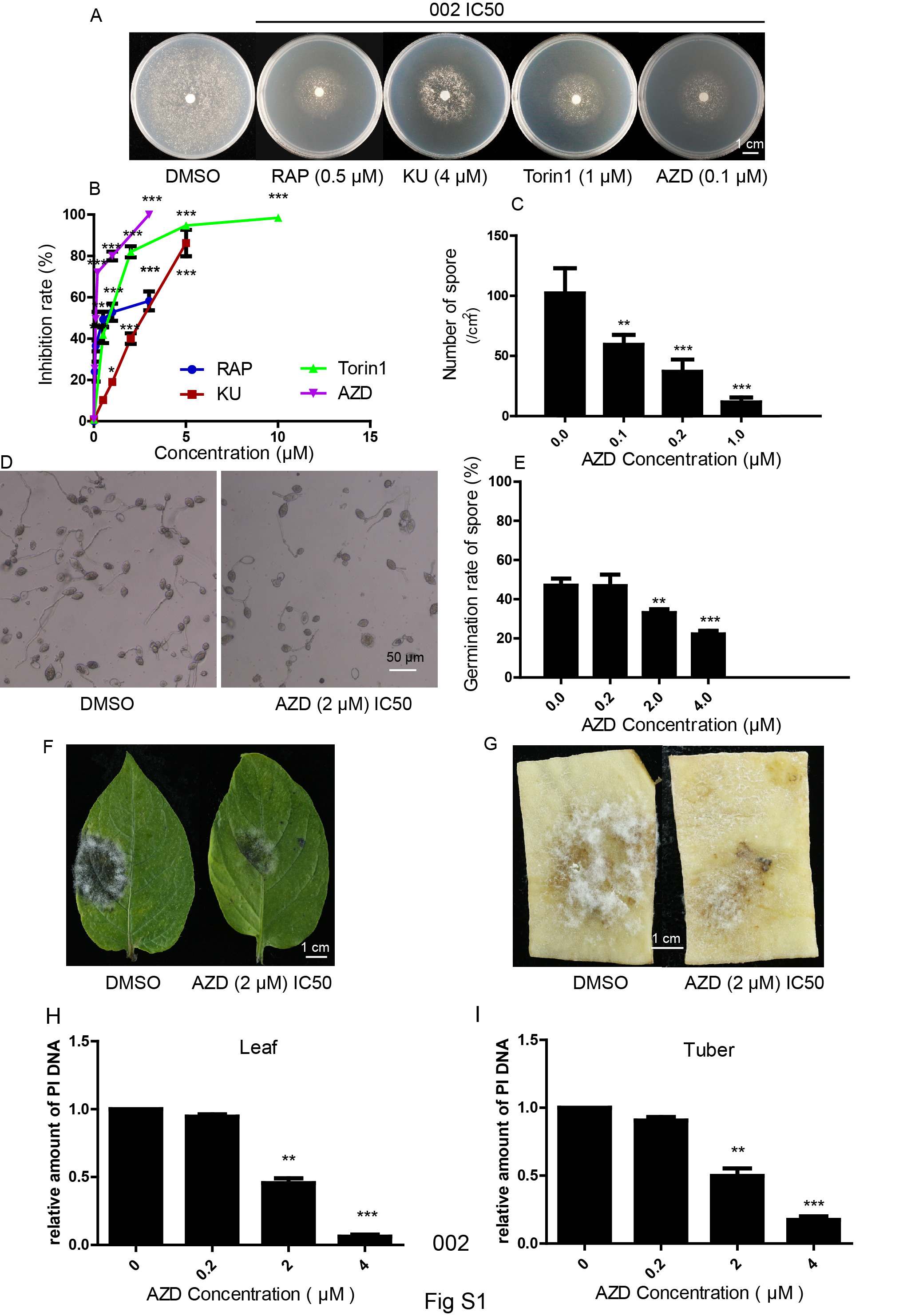

Supplement: Supplementary Figure 1 — TOR inhibitors inhibited P. infestans (002). (A) Mycelial phenotypes of 002 treated with IC50 of RAP, KU, Torin1, and AZD for 14 days. TOR inhibitors: Rapamycin (RAP), AZD8055 (AZD), Torin1, and KU. (B) Mycelia growth inhibition rate of 002 treated with different concentrations of four TOR inhibitors. (C) Inhibitory effects on the sporulation capacity of 002 treated with different concentrations of AZD. (D,E) Spore germination phenotypes or rates of 002 treated with different concentrations of AZD. (F,G) Disease symptoms on leaves and tubers were captured after inoculation with the 002 strain for 4 days. The leaves and tubers were inoculated with spore solutions pretreated with different concentrations of AZD. (H) and (I) Relative amounts of P. infestans DNA (002) in leaves and tubers after being inoculated by spore solutions. Spore solutions were pretreated with different concentrations of AZD. The data was normalized to the EF1 DNA levels of potato. (∗P < 0.05, ∗∗P < 0.01, ∗∗∗P < 0.001). [file Image_1.jpg]

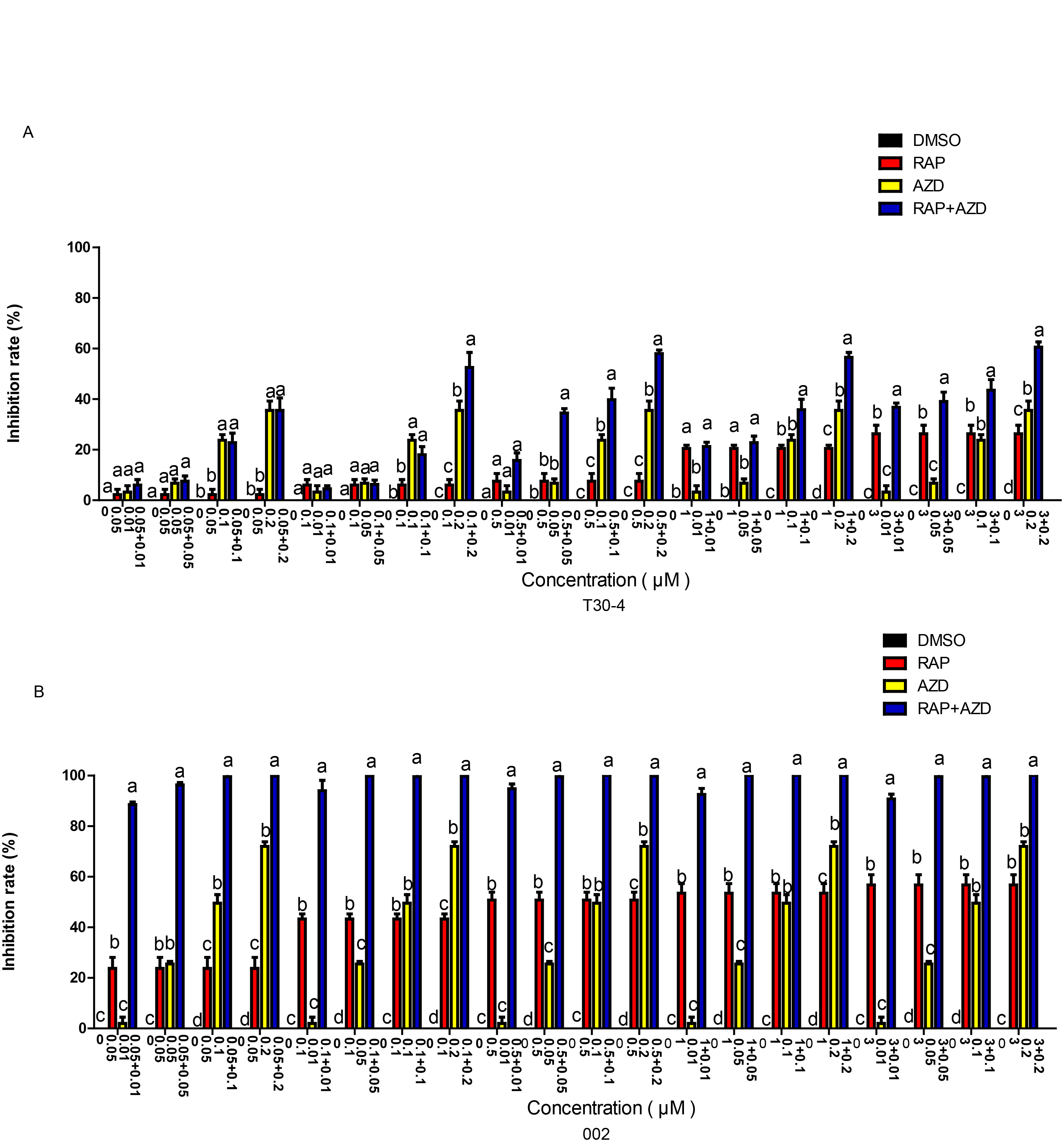

Supplement: Supplementary Figure 2 — Synergistic growth inhibition rate of P. infestans treated by co-application with RAP and AZD. (A) T30-4. (B) 002. (Lower case letters indicate significant difference, p < 0.05). [file Image_2.jpg]

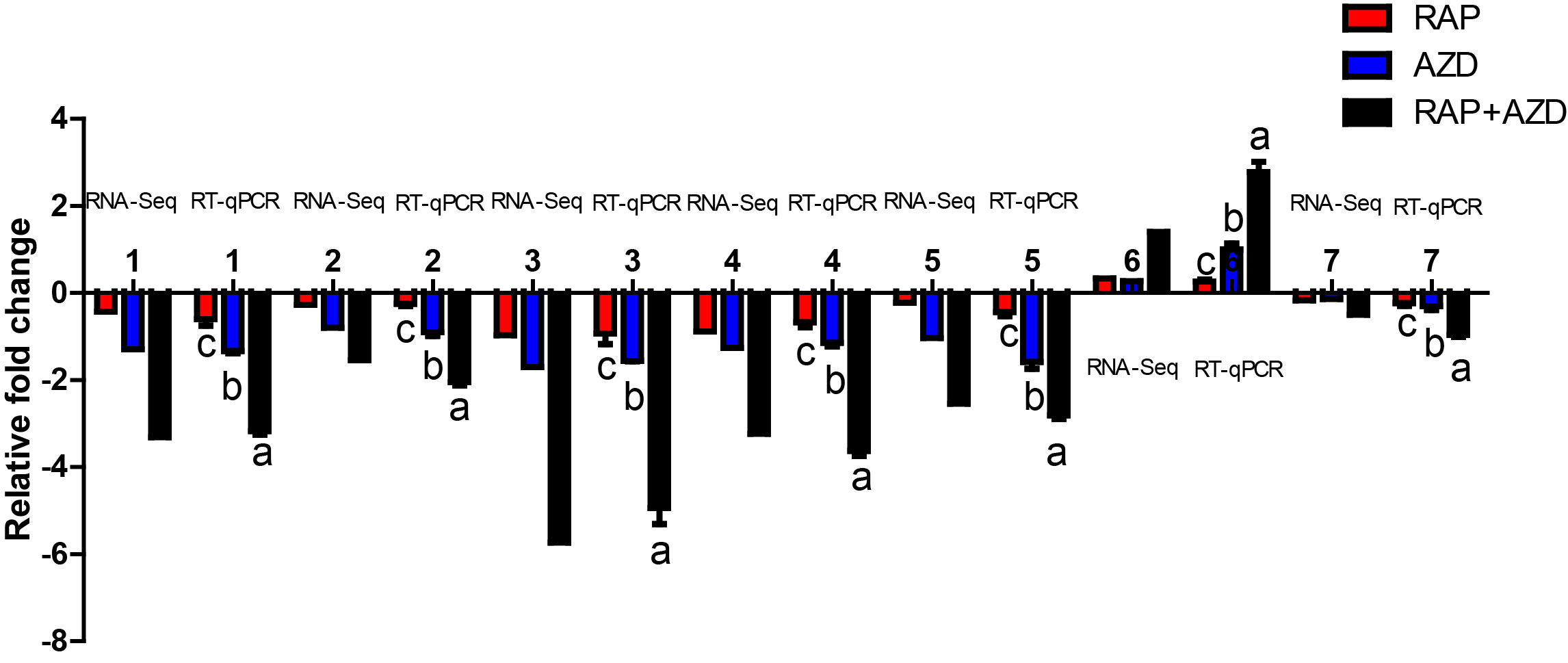

Supplement: Supplementary Figure 3 — Relative expression levels of seven synergistic DEGs both in transcriptome and RT-qPCR. 1. PITG_12483, eukaryotic translation initiation factor 3, putative; 2. PITG_07885, eukaryotic translation initiation factor 2, subunit alpha; 3. PITG_22657, elongation factor 1-alpha; 4. PITG_08714, Lysyl-tRNA synthetase; 5. PITG_03768, 50S ribosomal protein L4; 6. PITG_17009, selenocysteine-specific elongation factor; 7. PITG_18420, protein kinase. [file Image_3.jpg]
