# Supplementary material for: TOR Inhibitors Synergistically Suppress the Growth and Development of Phytophthora infestans, a Highly Destructive Pathogenic Oomycete
Source: Front Microbiol. 2021 Apr 16;12:596874. doi: 10.3389/fmicb.2021.596874 (PMC8086431; doi:10.3389/fmicb.2021.596874)
Supplement: Supplementary file 4 [file Data_Sheet_1.PDF]

1. cellular amide metabolic process

Table: GSEA Results Summary

Dataset	fpkm.sample
Phenotype	sample.cls
Upregulated in class	RAP
GeneSet	CELLULAR_AMIDE_METABOLIC_PROCESS(GO:0043603)
Enrichment Score (ES)	-0.7572587
Normalized Enrichment Score (NES)	-1.0858035
Nominal p-value	0.0
FDR q-value	0.077120826
FWER p-Value	0.06

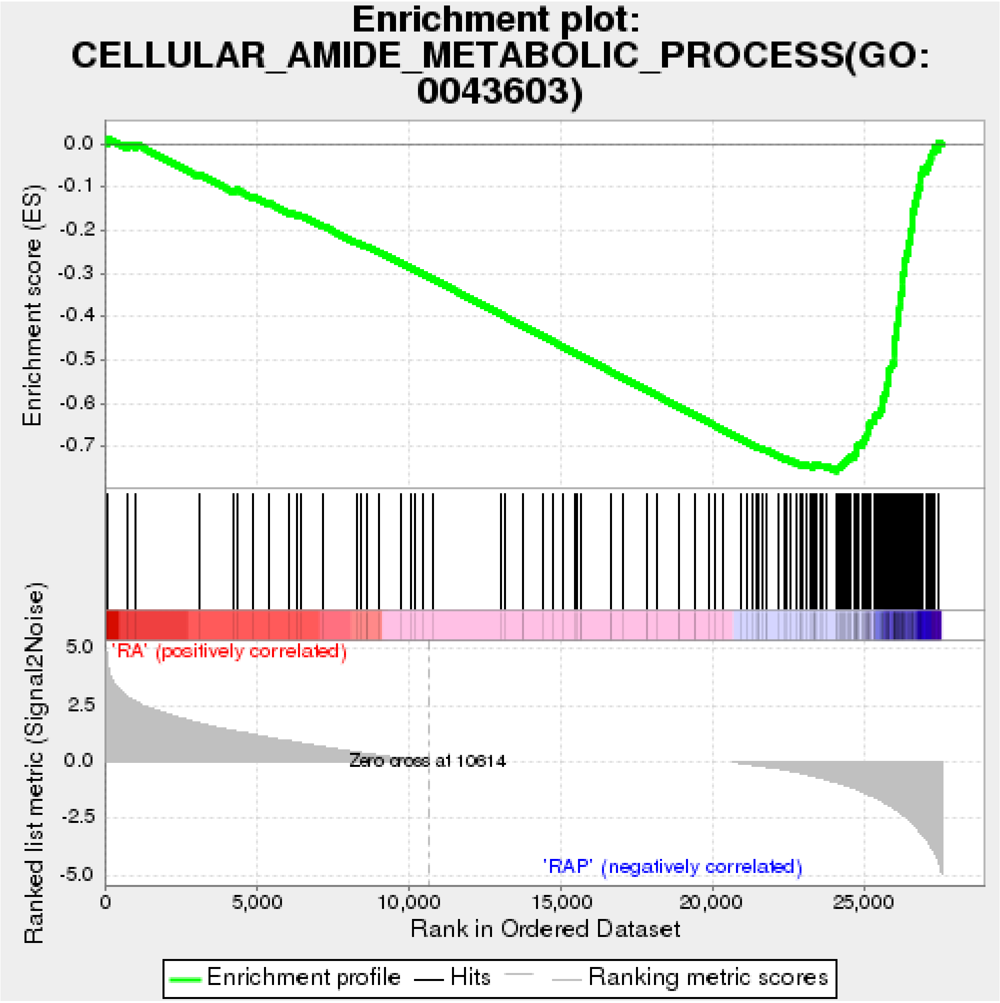

Fig 1: Enrichment plot: CELLULAR_AMIDE_METABOLIC_PROCESS(GO:0043603)
Profile of the Running ES Score & Positions of GeneSet Members on the Rank Ordered List

Table: GSEA details [plain text format]

	PROBE	DESCRIPTION	GENE	GENE_TITLE	RANK IN	RANK	RUNNING	CORE

		(from dataset)	SYMBOL		GENE LIST	METRIC SCORE	ES	ENRICHMENT
1	PITG_10138	PITG_10138			42	4.218	0.0137	No
2	PITG_04708	PITG_04708			676	2.910	0.0011	No
3	PITG_19121	PITG_19121			962	2.666	0.0004	No
4	PITG_00636	PITG_00636			3084	1.716	-0.0709	No
5	PITG_00177	PITG_00177			4178	1.386	-0.1058	No
6	PITG_16088	PITG_16088			4287	1.359	-0.1049	No
7	PITG_17651	PITG_17651			4826	1.224	-0.1201	No
8	Novel00922	Novel00922			5355	1.090	-0.1354	No
9	PITG_07234	PITG_07234			6061	0.926	-0.1579	No
10	PITG_05730	PITG_05730			6273	0.878	-0.1624	No
11	PITG_03660	PITG_03660			6456	0.832	-0.1660	No
12	PITG_15417	PITG_15417			7136	0.681	-0.1884	No
13	PITG_00543	PITG_00543			8271	0.430	-0.2282	No
14	PITG_08669	PITG_08669			8433	0.394	-0.2327	No
15	PITG_11630	PITG_11630			8572	0.362	-0.2364	No
16	PITG_10516	PITG_10516			8635	0.351	-0.2374	No
17	PITG_14557	PITG_14557			8993	0.287	-0.2494	No
18	PITG_18303	PITG_18303			9750	0.151	-0.2765	No
19	PITG_09791	PITG_09791			10045	0.103	-0.2869	No
20	PITG_02992	PITG_02992			10209	0.072	-0.2926	No
21	PITG_10139	PITG_10139			10456	0.026	-0.3015	No
22	PITG_20640	PITG_20640			10808	0.000	-0.3143	No
23	PITG_21582	PITG_21582			13034	0.000	-0.3956	No
24	PITG_20824	PITG_20824			13172	0.000	-0.4006	No
25	PITG_15722	PITG_15722			13740	0.000	-0.4213	No
26	PITG_22058	PITG_22058			13753	0.000	-0.4218	No
27	PITG_01091	PITG_01091			14376	0.000	-0.4445	No
28	PITG_05812	PITG_05812			14754	0.000	-0.4583	No
29	PITG_09431	PITG_09431			15054	0.000	-0.4692	No
30	PITG_17187	PITG_17187			15487	0.000	-0.4850	No
31	PITG_06873	PITG_06873			15537	0.000	-0.4868	No
32	PITG_19379	PITG_19379			15632	0.000	-0.4902	No
33	PITG_19374	PITG_19374			15633	0.000	-0.4902	No
34	PITG_14310	PITG_14310			16667	0.000	-0.5279	No
35	PITG_14315	PITG_14315			16668	0.000	-0.5279	No
36	PITG_16530	PITG_16530			17026	0.000	-0.5410	No
37	PITG_14346	PITG_14346			17808	0.000	-0.5695	No
38	PITG_14344	PITG_14344			17809	0.000	-0.5695	No

39	PITG_18225	PITG_18225			18167	0.000	-0.5826	No
40	PITG_14322	PITG_14322			18878	0.000	-0.6085	No
41	PITG_20240	PITG_20240			18905	0.000	-0.6095	No
42	PITG_03806	PITG_03806			19383	0.000	-0.6269	No
43	PITG_03807	PITG_03807			19384	0.000	-0.6269	No
44	PITG_18553	PITG_18553			19886	0.000	-0.6452	No
45	PITG_04594	PITG_04594			20065	0.000	-0.6517	No
46	PITG_14325	PITG_14325			20368	0.000	-0.6627	No
47	PITG_14312	PITG_14312			20936	-0.059	-0.6832	No
48	PITG_02493	PITG_02493			21136	-0.100	-0.6902	No
49	PITG_05009	PITG_05009			21324	-0.134	-0.6965	No
50	PITG_22310	PITG_22310			21487	-0.165	-0.7018	No
51	PITG_01580	PITG_01580			21509	-0.170	-0.7020	No
52	PITG_21979	PITG_21979			21528	-0.172	-0.7020	No
53	PITG_21349	PITG_21349			21623	-0.186	-0.7048	No
54	PITG_12151	PITG_12151			21769	-0.212	-0.7093	No
55	PITG_13735	PITG_13735			21784	-0.214	-0.7090	No
56	PITG_05007	PITG_05007			22208	-0.303	-0.7234	No
57	PITG_03093	PITG_03093			22409	-0.359	-0.7294	No
58	PITG_03681	PITG_03681			22436	-0.366	-0.7290	No
59	PITG_10193	PITG_10193			22606	-0.404	-0.7338	No
60	PITG_11734	PITG_11734			22780	-0.455	-0.7384	No
61	PITG_16328	PITG_16328			22919	-0.491	-0.7417	No
62	PITG_22249	PITG_22249			22933	-0.496	-0.7404	No
63	PITG_09846	PITG_09846			22986	-0.514	-0.7404	No
64	PITG_10519	PITG_10519			23111	-0.552	-0.7429	No
65	PITG_12961	PITG_12961			23230	-0.590	-0.7451	No
66	PITG_16741	PITG_16741			23328	-0.615	-0.7464	No
67	PITG_08369	PITG_08369			23360	-0.629	-0.7453	No
68	PITG_04918	PITG_04918			23385	-0.637	-0.7439	No
69	PITG_00757	PITG_00757			23392	-0.640	-0.7418	No
70	PITG_03799	PITG_03799			23448	-0.655	-0.7414	No
71	PITG_05354	PITG_05354			23584	-0.710	-0.7438	No
72	PITG_02580	PITG_02580			23651	-0.734	-0.7435	No
73	PITG_17748	PITG_17748			23795	-0.780	-0.7459	No
74	PITG_00754	PITG_00754			24106	-0.902	-0.7540	Yes
75	PITG_21071	PITG_21071			24126	-0.913	-0.7514	Yes
76	PITG_14456	PITG_14456			24139	-0.918	-0.7485	Yes
77	PITG_19669	PITG_19669			24217	-0.958	-0.7478	Yes

78	PITG_06222	PITG_06222			24228	-0.961	-0.7447	Yes
79	PITG_11111	PITG_11111			24301	-0.988	-0.7438	Yes
80	PITG_05733	PITG_05733			24305	-0.989	-0.7403	Yes
81	PITG_17153	PITG_17153			24348	-1.006	-0.7382	Yes
82	PITG_01762	PITG_01762			24386	-1.026	-0.7358	Yes
83	PITG_16757	PITG_16757			24413	-1.036	-0.7330	Yes
84	PITG_18251	PITG_18251			24457	-1.057	-0.7308	Yes
85	PITG_03322	PITG_03322			24462	-1.057	-0.7271	Yes
86	PITG_22572	PITG_22572			24528	-1.084	-0.7256	Yes
87	PITG_10979	PITG_10979			24567	-1.100	-0.7230	Yes
88	PITG_07797	PITG_07797			24662	-1.147	-0.7222	Yes
89	PITG_07888	PITG_07888			24666	-1.148	-0.7182	Yes
90	PITG_03274	PITG_03274			24715	-1.175	-0.7157	Yes
91	PITG_14609	PITG_14609			24732	-1.182	-0.7120	Yes
92	PITG_05405	PITG_05405			24742	-1.189	-0.7080	Yes
93	PITG_07841	PITG_07841			24743	-1.190	-0.7037	Yes
94	PITG_04703	PITG_04703			24756	-1.196	-0.6998	Yes
95	PITG_19999	PITG_19999			24792	-1.214	-0.6967	Yes
96	PITG_12864	PITG_12864			24801	-1.219	-0.6926	Yes
97	PITG_03480	PITG_03480			24930	-1.293	-0.6926	Yes
98	PITG_20189	PITG_20189			24966	-1.317	-0.6891	Yes
99	PITG_01922	PITG_01922			25002	-1.336	-0.6856	Yes
100	PITG_12839	PITG_12839			25031	-1.352	-0.6817	Yes
101	PITG_14850	PITG_14850			25066	-1.380	-0.6779	Yes
102	PITG_11923	PITG_11923			25096	-1.398	-0.6739	Yes
103	PITG_16008	PITG_16008			25114	-1.410	-0.6694	Yes
104	PITG_06771	PITG_06771			25118	-1.413	-0.6644	Yes
105	PITG_15723	PITG_15723			25159	-1.438	-0.6607	Yes
106	PITG_07141	PITG_07141			25165	-1.441	-0.6557	Yes
107	PITG_04992	PITG_04992			25172	-1.445	-0.6507	Yes
108	PITG_00443	PITG_00443			25193	-1.456	-0.6461	Yes
109	PITG_04843	PITG_04843			25217	-1.471	-0.6416	Yes
110	PITG_05171	PITG_05171			25340	-1.548	-0.6405	Yes
111	PITG_12745	PITG_12745			25347	-1.553	-0.6351	Yes
112	PITG_10974	PITG_10974			25362	-1.559	-0.6299	Yes
113	PITG_08206	PITG_08206			25382	-1.567	-0.6250	Yes
114	PITG_20188	PITG_20188			25502	-1.654	-0.6233	Yes
115	PITG_12697	PITG_12697			25529	-1.673	-0.6182	Yes
116	PITG_04729	PITG_04729			25577	-1.699	-0.6138	Yes

117	PITG_09234	PITG_09234			25595	-1.710	-0.6082	Yes
118	PITG_10887	PITG_10887			25597	-1.713	-0.6021	Yes
119	PITG_07300	PITG_07300			25611	-1.727	-0.5963	Yes
120	PITG_03221	PITG_03221			25613	-1.729	-0.5901	Yes
121	PITG_15090	PITG_15090			25650	-1.757	-0.5850	Yes
122	PITG_04774	PITG_04774			25653	-1.761	-0.5787	Yes
123	PITG_08703	PITG_08703			25716	-1.807	-0.5744	Yes
124	PITG_01255	PITG_01255			25748	-1.835	-0.5689	Yes
125	PITG_10110	PITG_10110			25769	-1.847	-0.5630	Yes
126	PITG_02694	PITG_02694			25772	-1.849	-0.5564	Yes
127	PITG_15069	PITG_15069			25785	-1.859	-0.5501	Yes
128	PITG_01943	PITG_01943			25791	-1.864	-0.5435	Yes
129	PITG_15407	PITG_15407			25795	-1.868	-0.5368	Yes
130	PITG_11766	PITG_11766			25814	-1.881	-0.5307	Yes
131	PITG_01833	PITG_01833			25831	-1.897	-0.5244	Yes
132	PITG_02921	PITG_02921			25864	-1.929	-0.5186	Yes
133	PITG_08579	PITG_08579			25907	-1.965	-0.5130	Yes
134	PITG_20264	PITG_20264			25950	-1.992	-0.5073	Yes
135	PITG_07173	PITG_07173			25980	-2.022	-0.5011	Yes
136	PITG_19157	PITG_19157			25981	-2.022	-0.4938	Yes
137	PITG_10863	PITG_10863			25982	-2.022	-0.4865	Yes
138	PITG_03460	PITG_03460			25987	-2.027	-0.4793	Yes
139	PITG_09540	PITG_09540			25997	-2.035	-0.4722	Yes
140	PITG_03661	PITG_03661			26015	-2.048	-0.4654	Yes
141	PITG_03353	PITG_03353			26026	-2.059	-0.4583	Yes
142	PITG_13831	PITG_13831			26029	-2.061	-0.4510	Yes
143	PITG_09506	PITG_09506			26047	-2.074	-0.4441	Yes
144	PITG_00941	PITG_00941			26052	-2.078	-0.4367	Yes
145	PITG_14913	PITG_14913			26073	-2.104	-0.4298	Yes
146	PITG_04487	PITG_04487			26098	-2.129	-0.4230	Yes
147	PITG_00302	PITG_00302			26103	-2.131	-0.4154	Yes
148	PITG_04747	PITG_04747			26111	-2.138	-0.4079	Yes
149	PITG_03235	PITG_03235			26130	-2.165	-0.4007	Yes
150	PITG_19531	PITG_19531			26134	-2.169	-0.3930	Yes
151	PITG_04382	PITG_04382			26147	-2.181	-0.3856	Yes
152	PITG_06995	PITG_06995			26169	-2.197	-0.3784	Yes
153	PITG_08959	PITG_08959			26174	-2.203	-0.3705	Yes
154	PITG_05174	PITG_05174			26178	-2.205	-0.3627	Yes
155	PITG_03239	PITG_03239			26180	-2.206	-0.3547	Yes

156	PITG_03294	PITG_03294			26205	-2.241	-0.3475	Yes
157	PITG_09555	PITG_09555			26249	-2.286	-0.3408	Yes
158	PITG_13399	PITG_13399			26268	-2.305	-0.3331	Yes
159	Novel00015	Novel00015			26276	-2.309	-0.3250	Yes
160	PITG_17785	PITG_17785			26278	-2.310	-0.3167	Yes
161	PITG_12947	PITG_12947			26294	-2.326	-0.3088	Yes
162	PITG_18052	PITG_18052			26295	-2.327	-0.3004	Yes
163	PITG_04683	PITG_04683			26329	-2.368	-0.2930	Yes
164	PITG_12077	PITG_12077			26335	-2.375	-0.2846	Yes
165	PITG_13371	PITG_13371			26336	-2.376	-0.2760	Yes
166	PITG_06636	PITG_06636			26348	-2.389	-0.2677	Yes
167	PITG_03178	PITG_03178			26392	-2.437	-0.2605	Yes
168	PITG_00523	PITG_00523			26405	-2.460	-0.2520	Yes
169	PITG_09631	PITG_09631			26454	-2.515	-0.2447	Yes
170	PITG_13681	PITG_13681			26460	-2.519	-0.2357	Yes
171	PITG_08129	PITG_08129			26476	-2.540	-0.2271	Yes
172	PITG_10146	PITG_10146			26517	-2.576	-0.2192	Yes
173	PITG_02578	PITG_02578			26546	-2.608	-0.2108	Yes
174	PITG_06237	PITG_06237			26557	-2.626	-0.2017	Yes
175	PITG_00910	PITG_00910			26570	-2.639	-0.1926	Yes
176	PITG_17607	PITG_17607			26591	-2.668	-0.1836	Yes
177	PITG_03420	PITG_03420			26604	-2.687	-0.1744	Yes
178	PITG_01042	PITG_01042			26613	-2.704	-0.1649	Yes
179	PITG_03999	PITG_03999			26623	-2.720	-0.1553	Yes
180	PITG_09521	PITG_09521			26652	-2.752	-0.1464	Yes
181	PITG_02039	PITG_02039			26671	-2.781	-0.1370	Yes
182	PITG_09552	PITG_09552			26705	-2.829	-0.1280	Yes
183	PITG_14729	PITG_14729			26712	-2.839	-0.1179	Yes
184	PITG_07991	PITG_07991			26768	-2.940	-0.1093	Yes
185	PITG_00397	PITG_00397			26815	-3.015	-0.1000	Yes
186	PITG_03768	PITG_03768			26845	-3.062	-0.0900	Yes
187	PITG_06821	PITG_06821			26849	-3.067	-0.0790	Yes
188	PITG_08714	PITG_08714			26862	-3.088	-0.0683	Yes
189	PITG_18054	PITG_18054			26907	-3.165	-0.0584	Yes
190	PITG_06596	PITG_06596			27055	-3.399	-0.0515	Yes
191	PITG_21661	PITG_21661			27155	-3.586	-0.0421	Yes
192	PITG_22020	PITG_22020			27176	-3.631	-0.0297	Yes
193	PITG_19399	PITG_19399			27227	-3.743	-0.0180	Yes
194	PITG_18545	PITG_18545			27314	-3.923	-0.0069	Yes

195	PITG_14352	PITG_14352			27476	-4.401	0.0031	Yes

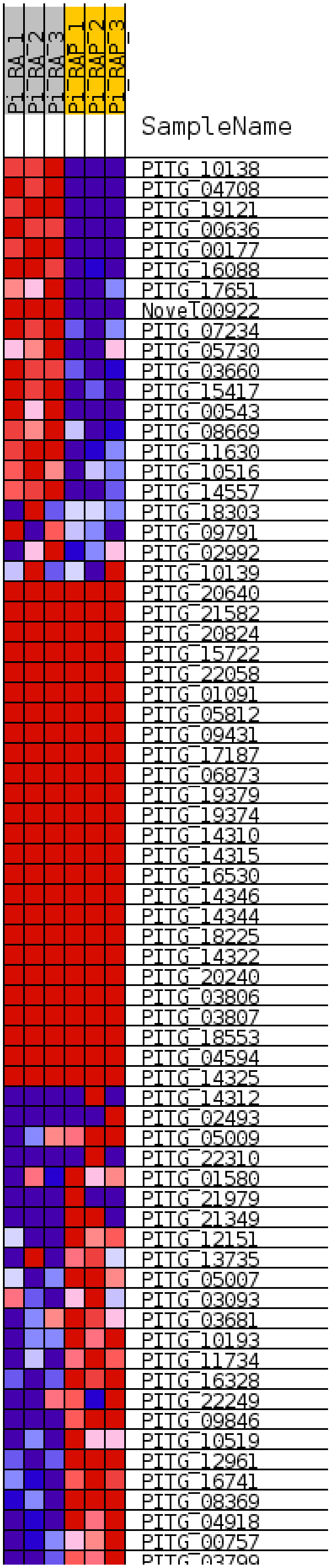

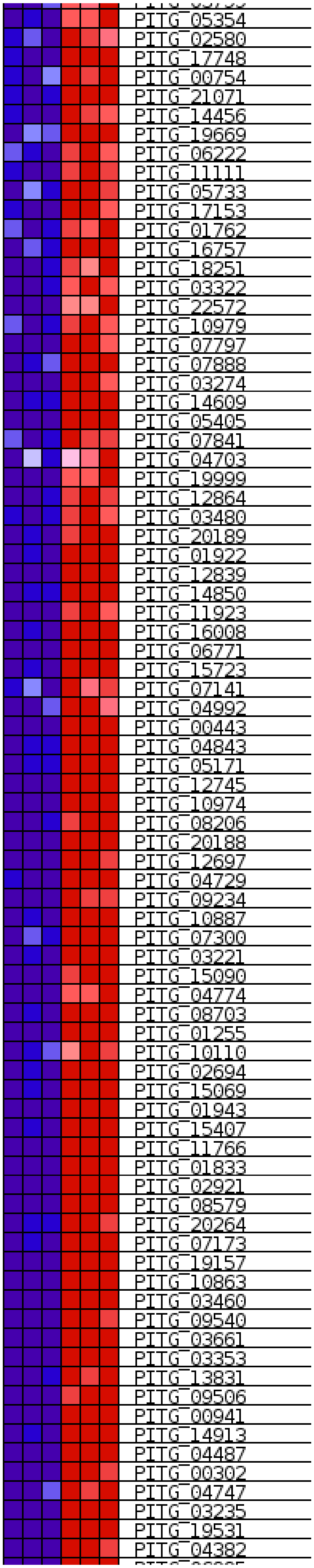
PITG_05354
PITG_02580
PITG_17748
PITG_00754
PITG_21071
PITG_14456
PITG_19669
PITG_06222
PITG_11111
PITG_05733
PITG_17153
PITG_01762
PITG_16757
PITG_18251
PITG_03322
PITG_22572
PITG_10979
PITG_07797
PITG_07888
PITG_03274
PITG_14609
PITG_05405
PITG_07841
PITG_04703
PITG_19999
PITG_12864
PITG_03480
PITG_20189
PITG_01922
PITG_12839
PITG_14850
PITG_11923
PITG_16008
PITG_06771
PITG_15723
PITG_07141
PITG_04992
PITG_00443
PITG_04843
PITG_05171
PITG_12745
PITG_10974
PITG_08206
PITG_20188
PITG_12697
PITG_04729
PITG_09234
PITG_10887
PITG_07300
PITG_03221
PITG_15090
PITG_04774
PITG_08703
PITG_01255
PITG_10110
PITG_02694
PITG_15069
PITG_01943
PITG_15407
PITG_11766
PITG_01833
PITG_02921
PITG_08579
PITG_20264
PITG_07173
PITG_19157
PITG_10863
PITG_03460
PITG_09540
PITG_03661
PITG_03353
PITG_13831
PITG_09506
PITG_00941
PITG_14913
PITG_04487
PITG_00302
PITG_04747
PITG_03235
PITG_19531
PITG_04382

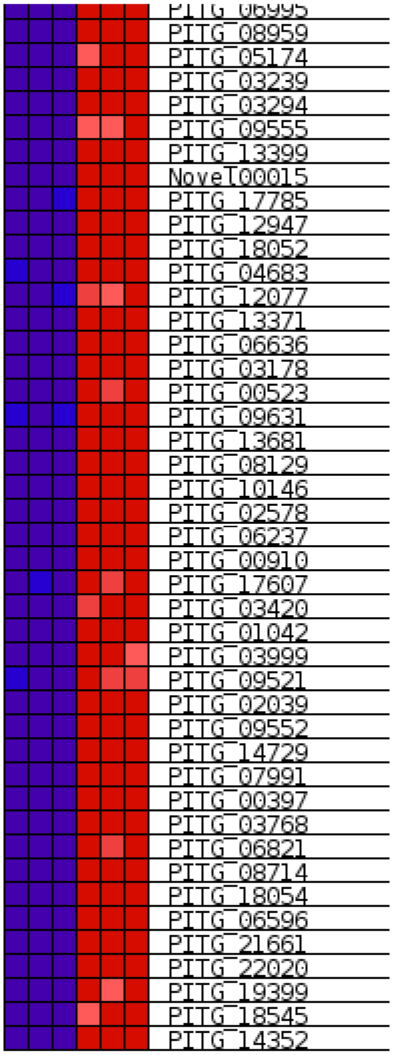

Fig 2: CELLULAR_AMIDE_METABOLIC_PROCESS(GO:0043603)
Blue-Pink O' Gram in the Space of the Analyzed GeneSet

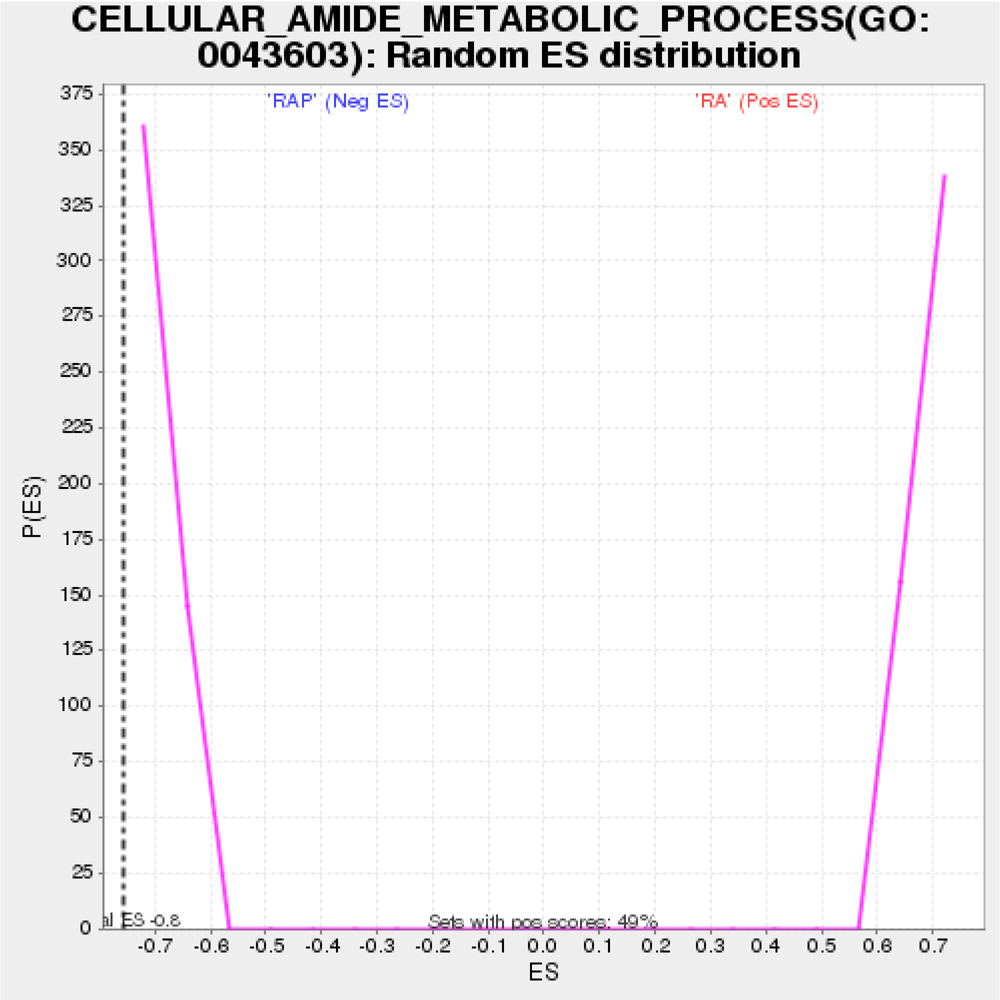

Fig 3: CELLULAR_AMIDE_METABOLIC_PROCESS(GO:0043603): Random ES distribution
Gene set null distribution of ES for CELLULAR_AMIDE_METABOLIC_PROCESS(GO:0043603)

2. organonitrogen compound biosynthetic process

Table: GSEA Results Summary

Dataset	fpkm.sample
Phenotype	sample.cls
Upregulated in class	RAP
GeneSet	ORGANONITROGEN_COMPOUND_BIOSYNTHETIC_PROCESS(GO:1901566)
Enrichment Score (ES)	-0.5964128
Normalized Enrichment Score (NES)	-1.2219783
Nominal p-value	0.0
FDR q-value	0.07712077
FWER p-Value	0.0

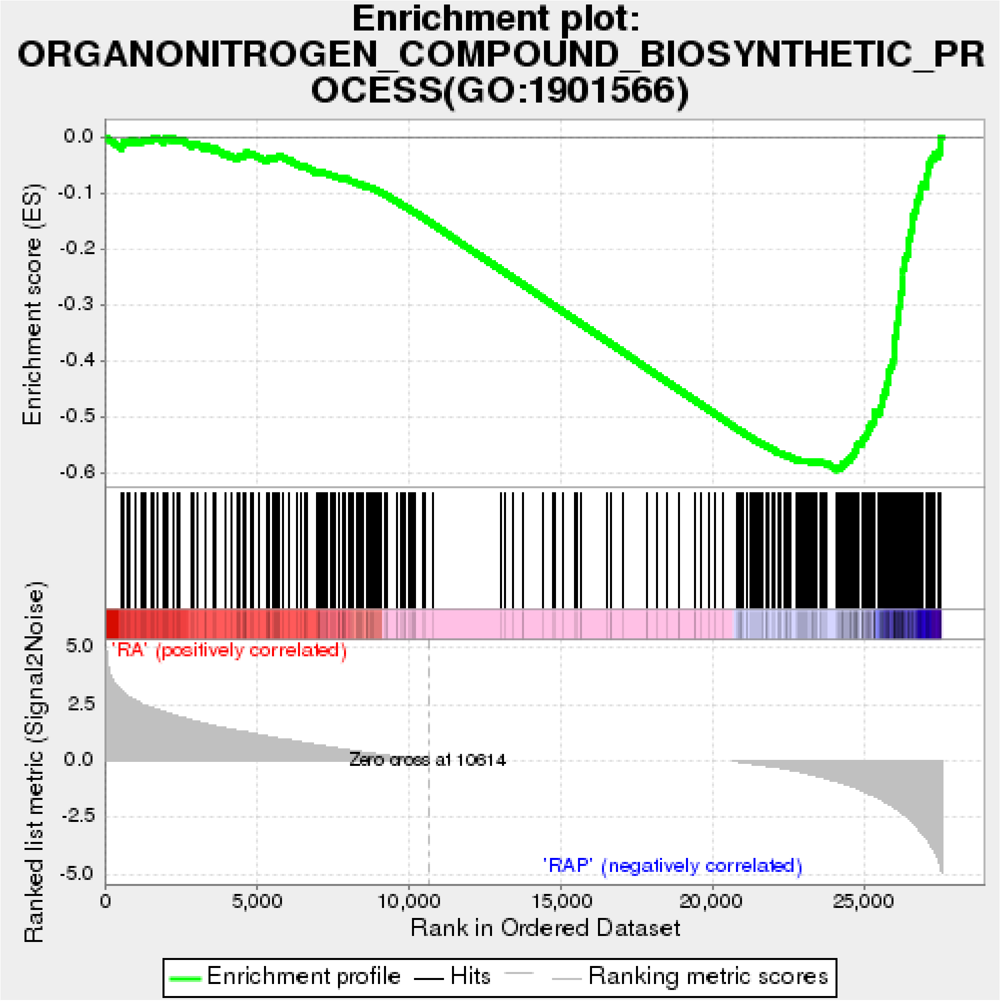

Fig 1: Enrichment plot: ORGANONITROGEN_COMPOUND_BIOSYNTHETIC_PROCESS(GO:1901566) Profile of the Running ES Score & Positions of GeneSet Members on the Rank Ordered List

Table: GSEA details ***[plain text format]***

	PROBE	DESCRIPTION (from dataset)	GENE SYMBOL	GENE_TITLE	RANK IN GENE LIST	RANK METRIC SCORE	RUNNING ES	CORE ENRICHMENT
1	PITG_06273	PITG_06273			527	3.082	-0.0129	No
2	PITG_06684	PITG_06684			546	3.068	-0.0071	No
3	PITG_04708	PITG_04708			676	2.910	-0.0057	No
4	PITG_02050	PITG_02050			791	2.809	-0.0040	No
5	PITG_19121	PITG_19121			962	2.666	-0.0047	No
6	PITG_02757	PITG_02757			1125	2.546	-0.0053	No
7	PITG_05953	PITG_05953			1190	2.503	-0.0024	No
8	PITG_18262	PITG_18262			1313	2.433	-0.0017	No
9	PITG_17661	PITG_17661			1472	2.355	-0.0026	No
10	PITG_15001	PITG_15001			1525	2.330	0.0004	No
11	PITG_14634	PITG_14634			1656	2.276	0.0004	No
12	PITG_09400	PITG_09400			1906	2.172	-0.0042	No
13	PITG_06022	PITG_06022			1925	2.161	-0.0003	No
14	PITG_21313	PITG_21313			2003	2.123	0.0013	No
15	PITG_02384	PITG_02384			2180	2.053	-0.0009	No
16	PITG_06280	PITG_06280			2319	1.997	-0.0017	No
17	PITG_18256	PITG_18256			2432	1.956	-0.0017	No
18	PITG_19310	PITG_19310			2829	1.796	-0.0125	No
19	PITG_21941	PITG_21941			2865	1.784	-0.0101	No
20	PITG_17126	PITG_17126			3003	1.737	-0.0115	No
21	PITG_07866	PITG_07866			3250	1.662	-0.0170	No
22	PITG_13024	PITG_13024			3262	1.657	-0.0139	No
23	PITG_16016	PITG_16016			3545	1.565	-0.0210	No
24	PITG_06279	PITG_06279			3573	1.557	-0.0187	No
25	PITG_03060	PITG_03060			3914	1.456	-0.0282	No
26	PITG_00430	PITG_00430			4119	1.406	-0.0327	No
27	PITG_16088	PITG_16088			4287	1.359	-0.0360	No
28	PITG_01188	PITG_01188			4341	1.345	-0.0351	No
29	PITG_09402	PITG_09402			4368	1.339	-0.0333	No
30	PITG_15003	PITG_15003			4408	1.329	-0.0319	No
31	PITG_04715	PITG_04715			4505	1.306	-0.0327	No
32	PITG_10448	PITG_10448			4532	1.302	-0.0309	No
33	PITG_14808	PITG_14808			4552	1.297	-0.0289	No
34	PITG_08348	PITG_08348			4611	1.282	-0.0283	No
35	PITG_20211	PITG_20211			4613	1.281	-0.0257	No
36	PITG_02489	PITG_02489			4763	1.242	-0.0285	No

37	PITG_17651	PITG_17651			4826	1.224	-0.0282	No
38	PITG_05374	PITG_05374			5055	1.167	-0.0342	No
39	PITG_13832	PITG_13832			5312	1.100	-0.0413	No
40	PITG_10601	PITG_10601			5327	1.097	-0.0395	No
41	Novel00922	Novel00922			5355	1.090	-0.0382	No
42	PITG_20634	PITG_20634			5357	1.090	-0.0359	No
43	PITG_13347	PITG_13347			5358	1.090	-0.0336	No
44	PITG_08810	PITG_08810			5519	1.046	-0.0373	No
45	PITG_18255	PITG_18255			5566	1.034	-0.0368	No
46	PITG_09393	PITG_09393			5588	1.031	-0.0355	No
47	PITG_18266	PITG_18266			5621	1.022	-0.0345	No
48	PITG_18279	PITG_18279			5653	1.012	-0.0335	No
49	PITG_00570	PITG_00570			5672	1.008	-0.0320	No
50	PITG_18271	PITG_18271			5685	1.005	-0.0304	No
51	PITG_15629	PITG_15629			5866	0.969	-0.0349	No
52	PITG_07234	PITG_07234			6061	0.926	-0.0401	No
53	PITG_05730	PITG_05730			6273	0.878	-0.0461	No
54	PITG_03660	PITG_03660			6456	0.832	-0.0510	No
55	PITG_18277	PITG_18277			6539	0.812	-0.0523	No
56	PITG_13043	PITG_13043			6540	0.812	-0.0506	No
57	PITG_18265	PITG_18265			6610	0.798	-0.0515	No
58	PITG_17295	PITG_17295			6973	0.713	-0.0633	No
59	PITG_00257	PITG_00257			6992	0.708	-0.0624	No
60	PITG_03634	PITG_03634			7030	0.702	-0.0623	No
61	PITG_01653	PITG_01653			7045	0.699	-0.0614	No
62	PITG_16057	PITG_16057			7082	0.692	-0.0612	No
63	PITG_15417	PITG_15417			7136	0.681	-0.0618	No
64	PITG_19429	PITG_19429			7229	0.661	-0.0638	No
65	PITG_14699	PITG_14699			7272	0.652	-0.0639	No
66	PITG_02393	PITG_02393			7297	0.645	-0.0635	No
67	PITG_18275	PITG_18275			7421	0.617	-0.0667	No
68	PITG_09092	PITG_09092			7493	0.600	-0.0680	No
69	PITG_06019	PITG_06019			7537	0.591	-0.0684	No
70	PITG_18276	PITG_18276			7651	0.567	-0.0713	No
71	PITG_09550	PITG_09550			7673	0.562	-0.0709	No
72	PITG_18272	PITG_18272			7695	0.557	-0.0705	No
73	PITG_17663	PITG_17663			7799	0.536	-0.0732	No
74	PITG_06724	PITG_06724			7835	0.527	-0.0734	No
75	PITG_18258	PITG_18258			7838	0.526	-0.0723	No

76	PITG_20746	PITG_20746			7846	0.525	-0.0715	No
77	PITG_19932	PITG_19932			7852	0.524	-0.0706	No
78	PITG_10077	PITG_10077			8008	0.487	-0.0753	No
79	PITG_10080	PITG_10080			8090	0.468	-0.0772	No
80	PITG_08761	PITG_08761			8111	0.464	-0.0770	No
81	PITG_10652	PITG_10652			8265	0.433	-0.0817	No
82	PITG_00543	PITG_00543			8271	0.430	-0.0810	No
83	PITG_02291	PITG_02291			8341	0.418	-0.0827	No
84	PITG_18257	PITG_18257			8371	0.409	-0.0829	No
85	PITG_12540	PITG_12540			8430	0.395	-0.0842	No
86	PITG_08669	PITG_08669			8433	0.394	-0.0834	No
87	PITG_18298	PITG_18298			8466	0.386	-0.0838	No
88	PITG_11630	PITG_11630			8572	0.362	-0.0869	No
89	PITG_06685	PITG_06685			8614	0.354	-0.0876	No
90	PITG_10516	PITG_10516			8635	0.351	-0.0876	No
91	PITG_13172	PITG_13172			8693	0.339	-0.0890	No
92	PITG_06738	PITG_06738			8723	0.334	-0.0894	No
93	PITG_01072	PITG_01072			8755	0.328	-0.0898	No
94	PITG_00471	PITG_00471			8794	0.321	-0.0906	No
95	PITG_12181	PITG_12181			8823	0.317	-0.0909	No
96	PITG_02394	PITG_02394			8866	0.309	-0.0918	No
97	PITG_06749	PITG_06749			8954	0.294	-0.0944	No
98	PITG_14557	PITG_14557			8993	0.287	-0.0952	No
99	PITG_18278	PITG_18278			9070	0.273	-0.0974	No
100	PITG_02854	PITG_02854			9178	0.250	-0.1008	No
101	PITG_06015	PITG_06015			9260	0.235	-0.1033	No
102	PITG_13991	PITG_13991			9286	0.230	-0.1038	No
103	PITG_02750	PITG_02750			9577	0.183	-0.1140	No
104	PITG_03456	PITG_03456			9585	0.180	-0.1139	No
105	PITG_18303	PITG_18303			9750	0.151	-0.1196	No
106	PITG_18296	PITG_18296			9785	0.145	-0.1206	No
107	PITG_17133	PITG_17133			9824	0.138	-0.1217	No
108	PITG_09394	PITG_09394			9840	0.133	-0.1220	No
109	PITG_18261	PITG_18261			9998	0.109	-0.1275	No
110	PITG_09791	PITG_09791			10045	0.103	-0.1290	No
111	PITG_14920	PITG_14920			10087	0.097	-0.1303	No
112	PITG_02136	PITG_02136			10102	0.094	-0.1306	No
113	PITG_02992	PITG_02992			10209	0.072	-0.1343	No
114	PITG_15000	PITG_15000			10212	0.072	-0.1343	No

115	PITG_11178	PITG_11178			10422	0.034	-0.1419	No
116	PITG_01193	PITG_01193			10499	0.023	-0.1446	No
117	PITG_20640	PITG_20640			10808	0.000	-0.1560	No
118	PITG_21582	PITG_21582			13034	0.000	-0.2378	No
119	PITG_20824	PITG_20824			13172	0.000	-0.2428	No
120	PITG_10610	PITG_10610			13398	0.000	-0.2511	No
121	PITG_15722	PITG_15722			13740	0.000	-0.2637	No
122	PITG_22058	PITG_22058			13753	0.000	-0.2641	No
123	PITG_01091	PITG_01091			14376	0.000	-0.2870	No
124	PITG_05812	PITG_05812			14754	0.000	-0.3008	No
125	PITG_20131	PITG_20131			14790	0.000	-0.3021	No
126	PITG_09431	PITG_09431			15054	0.000	-0.3118	No
127	PITG_17187	PITG_17187			15487	0.000	-0.3277	No
128	PITG_06873	PITG_06873			15537	0.000	-0.3295	No
129	PITG_19379	PITG_19379			15632	0.000	-0.3329	No
130	PITG_19374	PITG_19374			15633	0.000	-0.3329	No
131	PITG_21202	PITG_21202			16504	0.000	-0.3649	No
132	PITG_14310	PITG_14310			16667	0.000	-0.3709	No
133	PITG_14315	PITG_14315			16668	0.000	-0.3709	No
134	PITG_16530	PITG_16530			17026	0.000	-0.3840	No
135	PITG_14346	PITG_14346			17808	0.000	-0.4128	No
136	PITG_14344	PITG_14344			17809	0.000	-0.4128	No
137	PITG_18225	PITG_18225			18167	0.000	-0.4259	No
138	PITG_22629	PITG_22629			18497	0.000	-0.4380	No
139	PITG_14322	PITG_14322			18878	0.000	-0.4520	No
140	PITG_20240	PITG_20240			18905	0.000	-0.4529	No
141	PITG_03806	PITG_03806			19383	0.000	-0.4705	No
142	PITG_03807	PITG_03807			19384	0.000	-0.4705	No
143	PITG_05850	PITG_05850			19624	0.000	-0.4793	No
144	PITG_18553	PITG_18553			19886	0.000	-0.4889	No
145	PITG_04594	PITG_04594			20065	0.000	-0.4954	No
146	PITG_14325	PITG_14325			20368	0.000	-0.5065	No
147	PITG_06016	PITG_06016			20809	-0.027	-0.5226	No
148	PITG_03738	PITG_03738			20839	-0.035	-0.5236	No
149	PITG_17664	PITG_17664			20849	-0.037	-0.5239	No
150	PITG_11733	PITG_11733			20907	-0.054	-0.5259	No
151	PITG_12692	PITG_12692			20932	-0.059	-0.5266	No
152	PITG_14312	PITG_14312			20936	-0.059	-0.5266	No
153	PITG_07201	PITG_07201			20983	-0.069	-0.5282	No

154	PITG_06783	PITG_06783			21112	-0.095	-0.5327	No
155	PITG_02493	PITG_02493			21136	-0.100	-0.5333	No
156	PITG_03700	PITG_03700			21245	-0.122	-0.5370	No
157	PITG_05009	PITG_05009			21324	-0.134	-0.5396	No
158	PITG_13042	PITG_13042			21416	-0.151	-0.5426	No
159	PITG_22310	PITG_22310			21487	-0.165	-0.5449	No
160	PITG_21979	PITG_21979			21528	-0.172	-0.5460	No
161	PITG_05853	PITG_05853			21589	-0.178	-0.5478	No
162	PITG_21349	PITG_21349			21623	-0.186	-0.5486	No
163	PITG_01235	PITG_01235			21655	-0.190	-0.5494	No
164	PITG_14918	PITG_14918			21759	-0.210	-0.5527	No
165	PITG_12151	PITG_12151			21769	-0.212	-0.5526	No
166	PITG_13735	PITG_13735			21784	-0.214	-0.5527	No
167	PITG_00688	PITG_00688			21852	-0.227	-0.5547	No
168	PITG_03598	PITG_03598			21994	-0.254	-0.5593	No
169	PITG_06688	PITG_06688			22079	-0.271	-0.5618	No
170	PITG_18259	PITG_18259			22162	-0.292	-0.5642	No
171	PITG_05007	PITG_05007			22208	-0.303	-0.5652	No
172	PITG_01195	PITG_01195			22270	-0.321	-0.5668	No
173	PITG_06845	PITG_06845			22273	-0.322	-0.5662	No
174	PITG_03093	PITG_03093			22409	-0.359	-0.5704	No
175	PITG_03681	PITG_03681			22436	-0.366	-0.5706	No
176	PITG_05245	PITG_05245			22442	-0.368	-0.5700	No
177	PITG_12037	PITG_12037			22444	-0.368	-0.5693	No
178	PITG_16280	PITG_16280			22523	-0.387	-0.5713	No
179	PITG_13148	PITG_13148			22602	-0.404	-0.5734	No
180	PITG_10193	PITG_10193			22606	-0.404	-0.5726	No
181	PITG_11734	PITG_11734			22780	-0.455	-0.5780	No
182	PITG_01862	PITG_01862			22814	-0.464	-0.5783	No
183	PITG_03698	PITG_03698			22833	-0.469	-0.5779	No
184	PITG_16328	PITG_16328			22919	-0.491	-0.5800	No
185	PITG_22249	PITG_22249			22933	-0.496	-0.5795	No
186	PITG_09846	PITG_09846			22986	-0.514	-0.5803	No
187	PITG_09666	PITG_09666			22987	-0.514	-0.5792	No
188	PITG_09596	PITG_09596			23025	-0.527	-0.5795	No
189	PITG_02294	PITG_02294			23057	-0.537	-0.5795	No
190	PITG_19364	PITG_19364			23093	-0.548	-0.5796	No
191	PITG_10519	PITG_10519			23111	-0.552	-0.5791	No
192	PITG_12050	PITG_12050			23188	-0.576	-0.5807	No

193	PITG_12961	PITG_12961			23230	-0.590	-0.5809	No
194	PITG_00132	PITG_00132			23312	-0.612	-0.5826	No
195	PITG_04610	PITG_04610			23321	-0.613	-0.5816	No
196	PITG_16741	PITG_16741			23328	-0.615	-0.5806	No
197	PITG_08369	PITG_08369			23360	-0.629	-0.5804	No
198	PITG_04918	PITG_04918			23385	-0.637	-0.5799	No
199	PITG_02925	PITG_02925			23409	-0.645	-0.5794	No
200	PITG_03799	PITG_03799			23448	-0.655	-0.5794	No
201	PITG_12588	PITG_12588			23577	-0.708	-0.5827	No
202	PITG_05354	PITG_05354			23584	-0.710	-0.5814	No
203	PITG_02580	PITG_02580			23651	-0.734	-0.5823	No
204	PITG_08808	PITG_08808			23702	-0.751	-0.5825	No
205	PITG_17748	PITG_17748			23795	-0.780	-0.5843	No
206	PITG_21071	PITG_21071			24126	-0.913	-0.5945	Yes
207	PITG_19557	PITG_19557			24128	-0.914	-0.5926	Yes
208	PITG_14456	PITG_14456			24139	-0.918	-0.5910	Yes
209	PITG_19669	PITG_19669			24217	-0.958	-0.5919	Yes
210	PITG_06222	PITG_06222			24228	-0.961	-0.5902	Yes
211	PITG_01804	PITG_01804			24285	-0.982	-0.5902	Yes
212	PITG_08157	PITG_08157			24299	-0.988	-0.5886	Yes
213	PITG_11111	PITG_11111			24301	-0.988	-0.5866	Yes
214	PITG_05733	PITG_05733			24305	-0.989	-0.5846	Yes
215	PITG_17153	PITG_17153			24348	-1.006	-0.5840	Yes
216	PITG_01762	PITG_01762			24386	-1.026	-0.5832	Yes
217	PITG_15294	PITG_15294			24402	-1.032	-0.5816	Yes
218	PITG_16757	PITG_16757			24413	-1.036	-0.5798	Yes
219	PITG_18251	PITG_18251			24457	-1.057	-0.5792	Yes
220	PITG_03322	PITG_03322			24462	-1.057	-0.5771	Yes
221	PITG_22572	PITG_22572			24528	-1.084	-0.5772	Yes
222	PITG_15015	PITG_15015			24533	-1.085	-0.5751	Yes
223	PITG_09698	PITG_09698			24544	-1.090	-0.5731	Yes
224	PITG_10979	PITG_10979			24567	-1.100	-0.5716	Yes
225	PITG_09635	PITG_09635			24639	-1.133	-0.5719	Yes
226	PITG_04698	PITG_04698			24645	-1.135	-0.5697	Yes
227	PITG_10777	PITG_10777			24660	-1.145	-0.5678	Yes
228	PITG_07797	PITG_07797			24662	-1.147	-0.5654	Yes
229	PITG_07888	PITG_07888			24666	-1.148	-0.5631	Yes
230	PITG_03274	PITG_03274			24715	-1.175	-0.5624	Yes
231	PITG_14609	PITG_14609			24732	-1.182	-0.5605	Yes

232	PITG_05405	PITG_05405			24742	-1.189	-0.5583	Yes
233	PITG_07841	PITG_07841			24743	-1.190	-0.5558	Yes
234	PITG_04703	PITG_04703			24756	-1.196	-0.5537	Yes
235	PITG_14765	PITG_14765			24768	-1.201	-0.5516	Yes
236	PITG_19999	PITG_19999			24792	-1.214	-0.5499	Yes
237	PITG_12864	PITG_12864			24801	-1.219	-0.5477	Yes
238	PITG_02397	PITG_02397			24921	-1.290	-0.5493	Yes
239	PITG_03480	PITG_03480			24930	-1.293	-0.5469	Yes
240	PITG_16366	PITG_16366			24945	-1.308	-0.5447	Yes
241	PITG_20189	PITG_20189			24966	-1.317	-0.5426	Yes
242	PITG_00566	PITG_00566			24968	-1.317	-0.5399	Yes
243	PITG_01922	PITG_01922			25002	-1.336	-0.5383	Yes
244	PITG_12839	PITG_12839			25031	-1.352	-0.5365	Yes
245	PITG_14850	PITG_14850			25066	-1.380	-0.5348	Yes
246	PITG_11923	PITG_11923			25096	-1.398	-0.5330	Yes
247	PITG_16008	PITG_16008			25114	-1.410	-0.5306	Yes
248	PITG_06771	PITG_06771			25118	-1.413	-0.5278	Yes
249	PITG_15723	PITG_15723			25159	-1.438	-0.5262	Yes
250	PITG_07141	PITG_07141			25165	-1.441	-0.5234	Yes
251	PITG_04992	PITG_04992			25172	-1.445	-0.5205	Yes
252	PITG_00443	PITG_00443			25193	-1.456	-0.5182	Yes
253	PITG_04843	PITG_04843			25217	-1.471	-0.5160	Yes
254	PITG_18053	PITG_18053			25243	-1.483	-0.5138	Yes
255	PITG_09582	PITG_09582			25294	-1.519	-0.5124	Yes
256	PITG_06021	PITG_06021			25318	-1.533	-0.5100	Yes
257	PITG_20965	PITG_20965			25323	-1.536	-0.5070	Yes
258	PITG_05171	PITG_05171			25340	-1.548	-0.5043	Yes
259	PITG_12745	PITG_12745			25347	-1.553	-0.5013	Yes
260	PITG_10974	PITG_10974			25362	-1.559	-0.4985	Yes
261	PITG_09553	PITG_09553			25363	-1.561	-0.4952	Yes
262	PITG_07792	PITG_07792			25364	-1.561	-0.4919	Yes
263	PITG_20188	PITG_20188			25502	-1.654	-0.4935	Yes
264	PITG_12697	PITG_12697			25529	-1.673	-0.4909	Yes
265	PITG_12930	PITG_12930			25557	-1.688	-0.4884	Yes
266	PITG_15526	PITG_15526			25559	-1.690	-0.4849	Yes
267	PITG_04729	PITG_04729			25577	-1.699	-0.4819	Yes
268	PITG_05851	PITG_05851			25578	-1.700	-0.4783	Yes
269	PITG_09234	PITG_09234			25595	-1.710	-0.4753	Yes
270	PITG_10887	PITG_10887			25597	-1.713	-0.4718	Yes

271	PITG_07300	PITG_07300			25611	-1.727	-0.4686	Yes
272	PITG_03221	PITG_03221			25613	-1.729	-0.4650	Yes
273	PITG_15090	PITG_15090			25650	-1.757	-0.4626	Yes
274	PITG_04774	PITG_04774			25653	-1.761	-0.4590	Yes
275	PITG_19428	PITG_19428			25674	-1.776	-0.4560	Yes
276	PITG_08703	PITG_08703			25716	-1.807	-0.4537	Yes
277	PITG_01245	PITG_01245			25744	-1.834	-0.4509	Yes
278	PITG_01255	PITG_01255			25748	-1.835	-0.4471	Yes
279	PITG_10110	PITG_10110			25769	-1.847	-0.4440	Yes
280	PITG_02694	PITG_02694			25772	-1.849	-0.4402	Yes
281	PITG_15069	PITG_15069			25785	-1.859	-0.4367	Yes
282	PITG_01943	PITG_01943			25791	-1.864	-0.4330	Yes
283	PITG_15407	PITG_15407			25795	-1.868	-0.4291	Yes
284	PITG_10089	PITG_10089			25810	-1.878	-0.4257	Yes
285	PITG_11766	PITG_11766			25814	-1.881	-0.4219	Yes
286	PITG_01833	PITG_01833			25831	-1.897	-0.4185	Yes
287	PITG_02921	PITG_02921			25864	-1.929	-0.4156	Yes
288	PITG_13586	PITG_13586			25869	-1.931	-0.4117	Yes
289	PITG_08579	PITG_08579			25907	-1.965	-0.4089	Yes
290	PITG_20264	PITG_20264			25950	-1.992	-0.4063	Yes
291	PITG_09576	PITG_09576			25964	-2.006	-0.4025	Yes
292	PITG_00632	PITG_00632			25974	-2.018	-0.3986	Yes
293	PITG_18270	PITG_18270			25976	-2.021	-0.3944	Yes
294	PITG_07173	PITG_07173			25980	-2.022	-0.3903	Yes
295	PITG_19157	PITG_19157			25981	-2.022	-0.3860	Yes
296	PITG_10863	PITG_10863			25982	-2.022	-0.3818	Yes
297	PITG_03460	PITG_03460			25987	-2.027	-0.3776	Yes
298	PITG_09540	PITG_09540			25997	-2.035	-0.3737	Yes
299	PITG_03661	PITG_03661			26015	-2.048	-0.3700	Yes
300	PITG_14936	PITG_14936			26019	-2.053	-0.3658	Yes
301	PITG_03353	PITG_03353			26026	-2.059	-0.3617	Yes
302	PITG_13831	PITG_13831			26029	-2.061	-0.3574	Yes
303	PITG_09640	PITG_09640			26046	-2.074	-0.3537	Yes
304	PITG_09506	PITG_09506			26047	-2.074	-0.3493	Yes
305	PITG_00941	PITG_00941			26052	-2.078	-0.3451	Yes
306	PITG_14913	PITG_14913			26073	-2.104	-0.3414	Yes
307	PITG_04487	PITG_04487			26098	-2.129	-0.3378	Yes
308	PITG_00302	PITG_00302			26103	-2.131	-0.3335	Yes
309	PITG_04747	PITG_04747			26111	-2.138	-0.3292	Yes

310	PITG_00074	PITG_00074			26129	-2.164	-0.3253	Yes	
311	PITG_03235	PITG_03235			26130	-2.165	-0.3208	Yes	
312	PITG_19531	PITG_19531			26134	-2.169	-0.3163	Yes	
313	PITG_04382	PITG_04382			26147	-2.181	-0.3122	Yes	
314	PITG_06518	PITG_06518			26151	-2.183	-0.3077	Yes	
315	PITG_06995	PITG_06995			26169	-2.197	-0.3037	Yes	
316	PITG_08959	PITG_08959			26174	-2.203	-0.2992	Yes	
317	PITG_05174	PITG_05174			26178	-2.205	-0.2947	Yes	
318	PITG_03239	PITG_03239			26180	-2.206	-0.2901	Yes	
319	PITG_01769	PITG_01769			26199	-2.231	-0.2860	Yes	
320	PITG_03294	PITG_03294			26205	-2.241	-0.2815	Yes	
321	PITG_07405	PITG_07405			26228	-2.258	-0.2776	Yes	
322	PITG_09555	PITG_09555			26249	-2.286	-0.2735	Yes	
323	PITG_13399	PITG_13399			26268	-2.305	-0.2693	Yes	
324	Novel00015	Novel00015			26276	-2.309	-0.2647	Yes	
325	PITG_17785	PITG_17785			26278	-2.310	-0.2599	Yes	
326	PITG_09547	PITG_09547			26285	-2.315	-0.2553	Yes	
327	PITG_04418	PITG_04418			26293	-2.325	-0.2506	Yes	
328	PITG_12947	PITG_12947			26294	-2.326	-0.2457	Yes	
329	PITG_18052	PITG_18052			26295	-2.327	-0.2409	Yes	
330	PITG_22112	PITG_22112			26302	-2.335	-0.2362	Yes	
331	PITG_04683	PITG_04683			26329	-2.368	-0.2321	Yes	
332	PITG_12077	PITG_12077			26335	-2.375	-0.2273	Yes	
333	PITG_13371	PITG_13371			26336	-2.376	-0.2223	Yes	
334	PITG_06636	PITG_06636			26348	-2.389	-0.2177	Yes	
335	PITG_03178	PITG_03178			26392	-2.437	-0.2142	Yes	
336	PITG_00523	PITG_00523			26405	-2.460	-0.2095	Yes	
337	PITG_09726	PITG_09726			26447	-2.506	-0.2057	Yes	
338	PITG_09631	PITG_09631			26454	-2.515	-0.2006	Yes	
339	PITG_14195	PITG_14195			26456	-2.517	-0.1954	Yes	
340	PITG_13681	PITG_13681			26460	-2.519	-0.1902	Yes	
341	PITG_08129	PITG_08129			26476	-2.540	-0.1854	Yes	
342	PITG_04419	PITG_04419			26491	-2.550	-0.1805	Yes	
343	PITG_10146	PITG_10146			26517	-2.576	-0.1761	Yes	
344	PITG_02578	PITG_02578			26546	-2.608	-0.1716	Yes	
345	PITG_06237	PITG_06237			26557	-2.626	-0.1664	Yes	
346	PITG_00910	PITG_00910			26570	-2.639	-0.1613	Yes	
347	PITG_17607	PITG_17607			26591	-2.668	-0.1565	Yes	
348	PITG_03420	PITG_03420			26604	-2.687	-0.1513	Yes	

349	PITG_01042	PITG_01042			26613	-2.704	-0.1459	Yes
350	PITG_03999	PITG_03999			26623	-2.720	-0.1405	Yes
351	PITG_09521	PITG_09521			26652	-2.752	-0.1357	Yes
352	PITG_02039	PITG_02039			26671	-2.781	-0.1305	Yes
353	PITG_09552	PITG_09552			26705	-2.829	-0.1258	Yes
354	PITG_14729	PITG_14729			26712	-2.839	-0.1201	Yes
355	PITG_16736	PITG_16736			26755	-2.913	-0.1155	Yes
356	PITG_07991	PITG_07991			26768	-2.940	-0.1097	Yes
357	PITG_00397	PITG_00397			26815	-3.015	-0.1051	Yes
358	PITG_03768	PITG_03768			26845	-3.062	-0.0997	Yes
359	PITG_06821	PITG_06821			26849	-3.067	-0.0934	Yes
360	PITG_08714	PITG_08714			26862	-3.088	-0.0873	Yes
361	PITG_18054	PITG_18054			26907	-3.165	-0.0823	Yes
362	PITG_12300	PITG_12300			27048	-3.394	-0.0803	Yes
363	PITG_06596	PITG_06596			27055	-3.399	-0.0734	Yes
364	PITG_02392	PITG_02392			27080	-3.440	-0.0670	Yes
365	PITG_00571	PITG_00571			27095	-3.470	-0.0603	Yes
366	PITG_14639	PITG_14639			27127	-3.538	-0.0540	Yes
367	PITG_21661	PITG_21661			27155	-3.586	-0.0474	Yes
368	PITG_22020	PITG_22020			27176	-3.631	-0.0405	Yes
369	PITG_19399	PITG_19399			27227	-3.743	-0.0345	Yes
370	PITG_18545	PITG_18545			27314	-3.923	-0.0294	Yes
371	PITG_14352	PITG_14352			27476	-4.401	-0.0261	Yes
372	PITG_01062	PITG_01062			27511	-4.554	-0.0177	Yes
373	PITG_19993	PITG_19993			27522	-4.614	-0.0084	Yes
374	PITG_02621	PITG_02621			27528	-4.667	0.0012	Yes

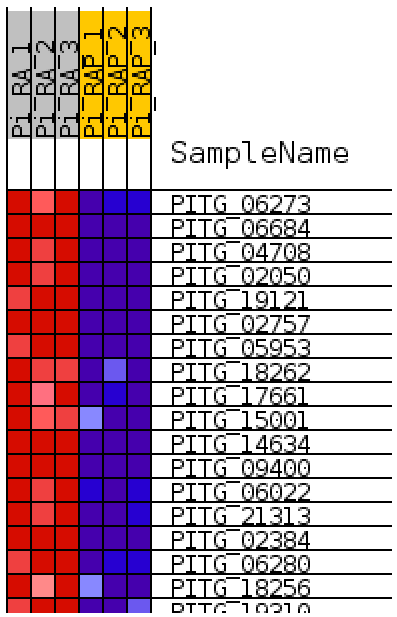

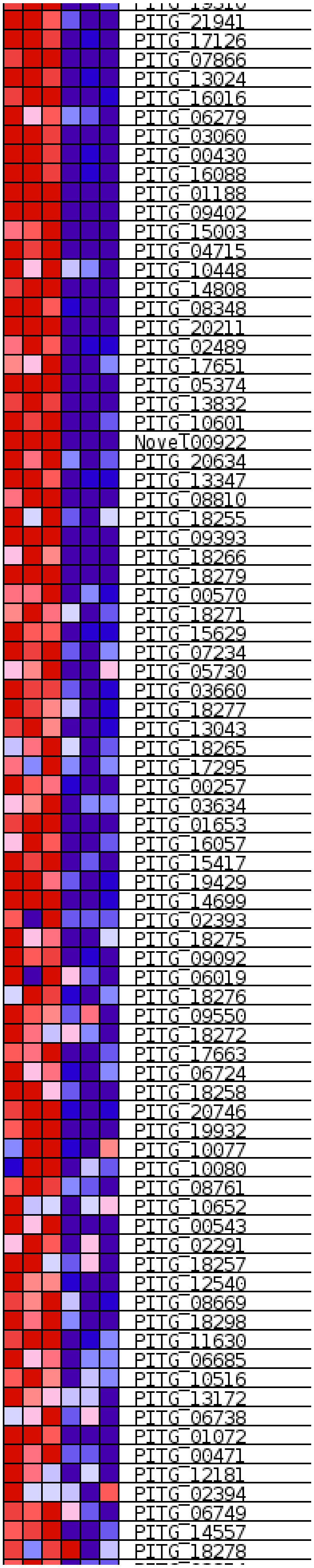
PITG_21941
PITG_17126
PITG_07866
PITG_13024
PITG_16016
PITG_06279
PITG_03060
PITG_00430
PITG_16088
PITG_01188
PITG_09402
PITG_15003
PITG_04715
PITG_10448
PITG_14808
PITG_08348
PITG_20211
PITG_02489
PITG_17651
PITG_05374
PITG_13832
PITG_10601
Novel00922
PITG_20634
PITG_13347
PITG_08810
PITG_18255
PITG_09393
PITG_18266
PITG_18279
PITG_00570
PITG_18271
PITG_15629
PITG_07234
PITG_05730
PITG_03660
PITG_18277
PITG_13043
PITG_18265
PITG_17295
PITG_00257
PITG_03634
PITG_01653
PITG_16057
PITG_15417
PITG_19429
PITG_14699
PITG_02393
PITG_18275
PITG_09092
PITG_06019
PITG_18276
PITG_09550
PITG_18272
PITG_17663
PITG_06724
PITG_18258
PITG_20746
PITG_19932
PITG_10077
PITG_10080
PITG_08761
PITG_10652
PITG_00543
PITG_02291
PITG_18257
PITG_12540
PITG_08669
PITG_18298
PITG_11630
PITG_06685
PITG_10516
PITG_13172
PITG_06738
PITG_01072
PITG_00471
PITG_12181
PITG_02394
PITG_06749
PITG_14557
PITG_18278

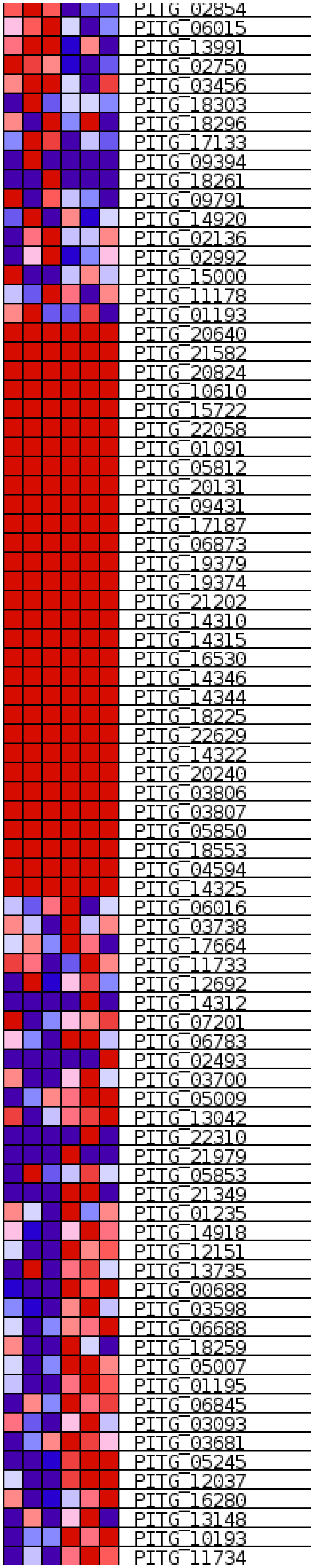
PITG_02854
PITG_06015
PITG_13991
PITG_02750
PITG_03456
PITG_18303
PITG_18296
PITG_17133
PITG_09394
PITG_18261
PITG_09791
PITG_14920
PITG_02136
PITG_02992
PITG_15000
PITG_11178
PITG_01193
PITG_20640
PITG_21582
PITG_20824
PITG_10610
PITG_15722
PITG_22058
PITG_01091
PITG_05812
PITG_20131
PITG_09431
PITG_17187
PITG_06873
PITG_19379
PITG_19374
PITG_21202
PITG_14310
PITG_14315
PITG_16530
PITG_14346
PITG_14344
PITG_18225
PITG_22629
PITG_14322
PITG_20240
PITG_03806
PITG_03807
PITG_05850
PITG_18553
PITG_04594
PITG_14325
PITG_06016
PITG_03738
PITG_17664
PITG_11733
PITG_12692
PITG_14312
PITG_07201
PITG_06783
PITG_02493
PITG_03700
PITG_05009
PITG_13042
PITG_22310
PITG_21979
PITG_05853
PITG_21349
PITG_01235
PITG_14918
PITG_12151
PITG_13735
PITG_00688
PITG_03598
PITG_06688
PITG_18259
PITG_05007
PITG_01195
PITG_06845
PITG_03093
PITG_03681
PITG_05245
PITG_12037
PITG_16280
PITG_13148
PITG_10193
PITG_11734

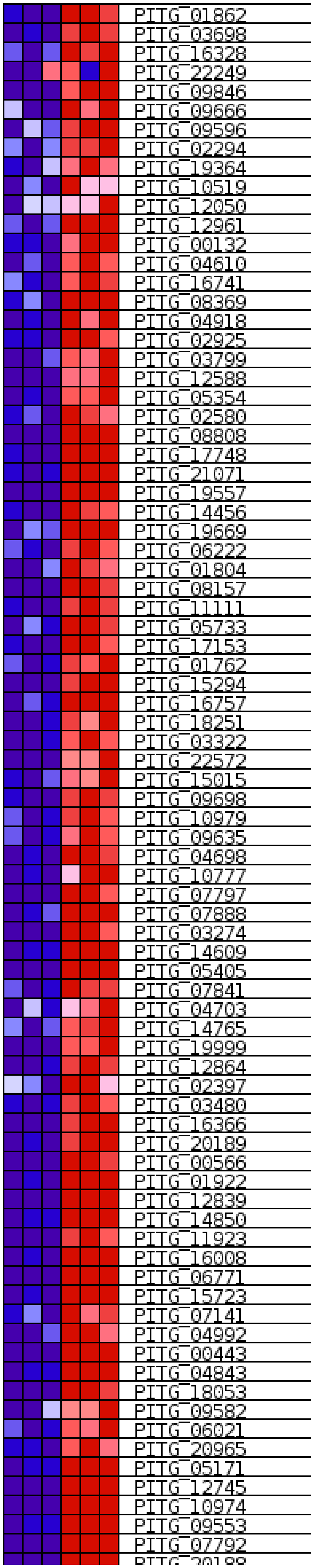
PITG_01862
PITG_03698
PITG_16328
PITG_22249
PITG_09846
PITG_09666
PITG_09596
PITG_02294
PITG_19364
PITG_10519
PITG_12050
PITG_12961
PITG_00132
PITG_04610
PITG_16741
PITG_08369
PITG_04918
PITG_02925
PITG_03799
PITG_12588
PITG_05354
PITG_02580
PITG_08808
PITG_17748
PITG_21071
PITG_19557
PITG_14456
PITG_19669
PITG_06222
PITG_01804
PITG_08157
PITG_11111
PITG_05733
PITG_17153
PITG_01762
PITG_15294
PITG_16757
PITG_18251
PITG_03322
PITG_22572
PITG_15015
PITG_09698
PITG_10979
PITG_09635
PITG_04698
PITG_10777
PITG_07797
PITG_07888
PITG_03274
PITG_14609
PITG_05405
PITG_07841
PITG_04703
PITG_14765
PITG_19999
PITG_12864
PITG_02397
PITG_03480
PITG_16366
PITG_20189
PITG_00566
PITG_01922
PITG_12839
PITG_14850
PITG_11923
PITG_16008
PITG_06771
PITG_15723
PITG_07141
PITG_04992
PITG_00443
PITG_04843
PITG_18053
PITG_09582
PITG_06021
PITG_20965
PITG_05171
PITG_12745
PITG_10974
PITG_09553
PITG_07792

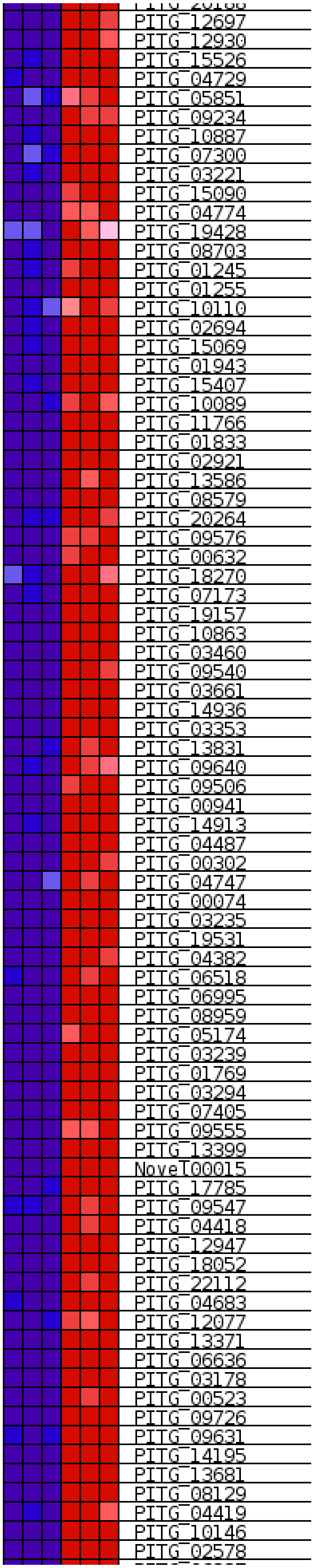
PITG_12697
PITG_12930
PITG_15526
PITG_04729
PITG_05851
PITG_09234
PITG_10887
PITG_07300
PITG_03221
PITG_15090
PITG_04774
PITG_19428
PITG_08703
PITG_01245
PITG_01255
PITG_10110
PITG_02694
PITG_15069
PITG_01943
PITG_15407
PITG_10089
PITG_11766
PITG_01833
PITG_02921
PITG_13586
PITG_08579
PITG_20264
PITG_09576
PITG_00632
PITG_18270
PITG_07173
PITG_19157
PITG_10863
PITG_03460
PITG_09540
PITG_03661
PITG_14936
PITG_03353
PITG_13831
PITG_09640
PITG_09506
PITG_00941
PITG_14913
PITG_04487
PITG_00302
PITG_04747
PITG_00074
PITG_03235
PITG_19531
PITG_04382
PITG_06518
PITG_06995
PITG_08959
PITG_05174
PITG_03239
PITG_01769
PITG_03294
PITG_07405
PITG_09555
PITG_13399
Novel00015
PITG_17785
PITG_09547
PITG_04418
PITG_12947
PITG_18052
PITG_22112
PITG_04683
PITG_12077
PITG_13371
PITG_06636
PITG_03178
PITG_00523
PITG_09726
PITG_09631
PITG_14195
PITG_13681
PITG_08129
PITG_04419
PITG_10146
PITG_02578

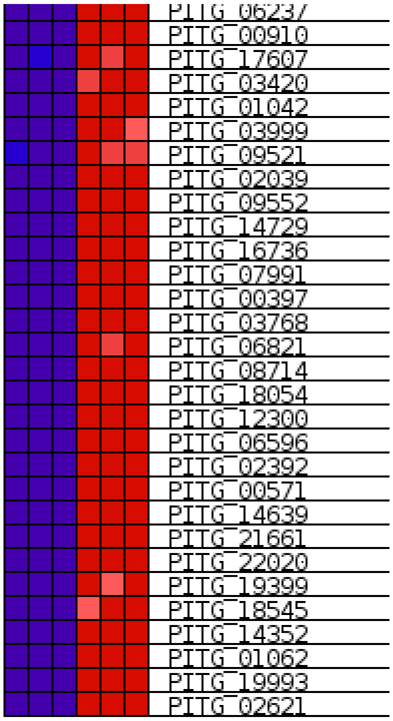

Fig 2: ORGANONITROGEN_COMPOUND_BIOSYNTHETIC_PROCESS(GO:1901566) Blue-Pink O' Gram in the Space of the Analyzed GeneSet

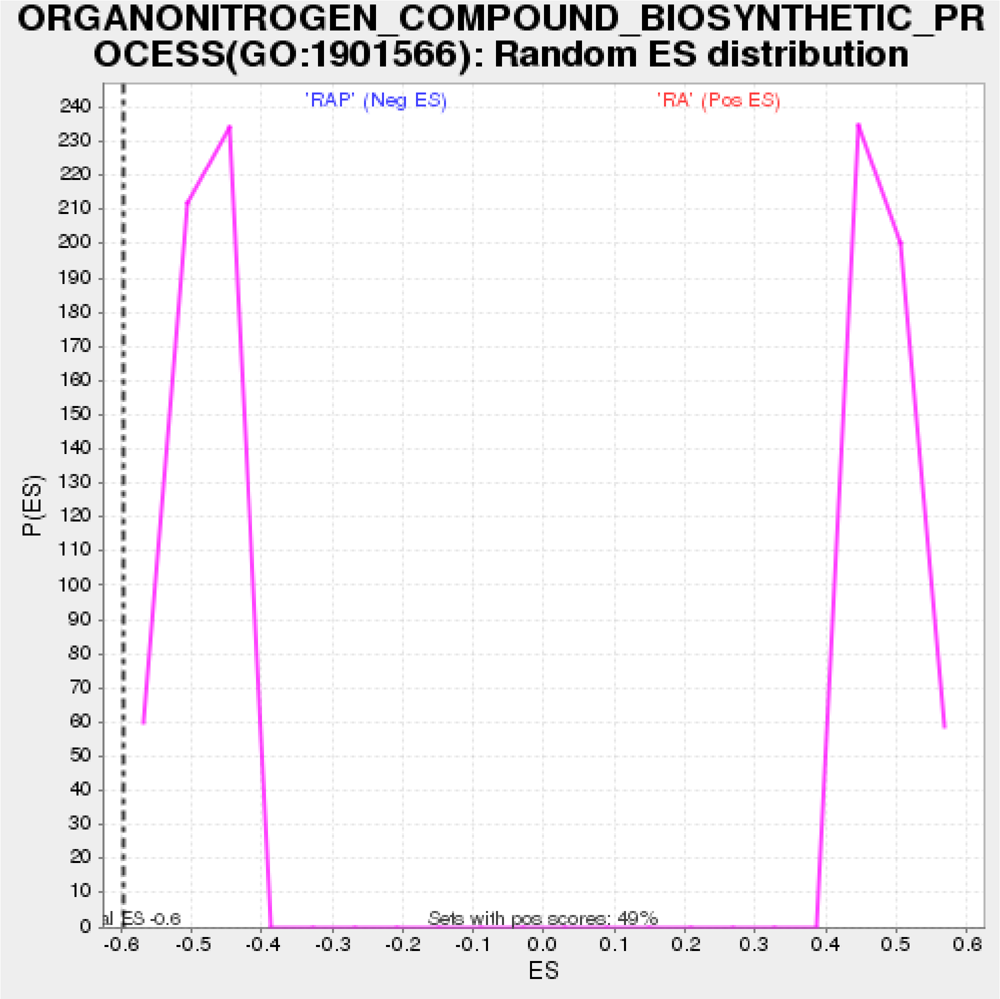

Fig 3: ORGANONITROGEN_COMPOUND_BIOSYNTHETIC_PROCESS(GO:1901566): Random ES distribution

Gene set null distribution of ES for ORGANONITROGEN_COMPOUND_BIOSYNTHETIC_PROCESS(GO:1901566)

3. organonitrogen compound metabolic process

Table: GSEA Results Summary

Dataset	fpkm.sample
Phenotype	sample.cls
Upregulated in class	RAP
GeneSet	ORGANONITROGEN_COMPOUND_METABOLIC_PROCESS(GO:1901564)
Enrichment Score (ES)	-0.28257424
Normalized Enrichment Score (NES)	-1.162863
Nominal p-value	0.0
FDR q-value	0.07712077
FWER p-Value	0.06

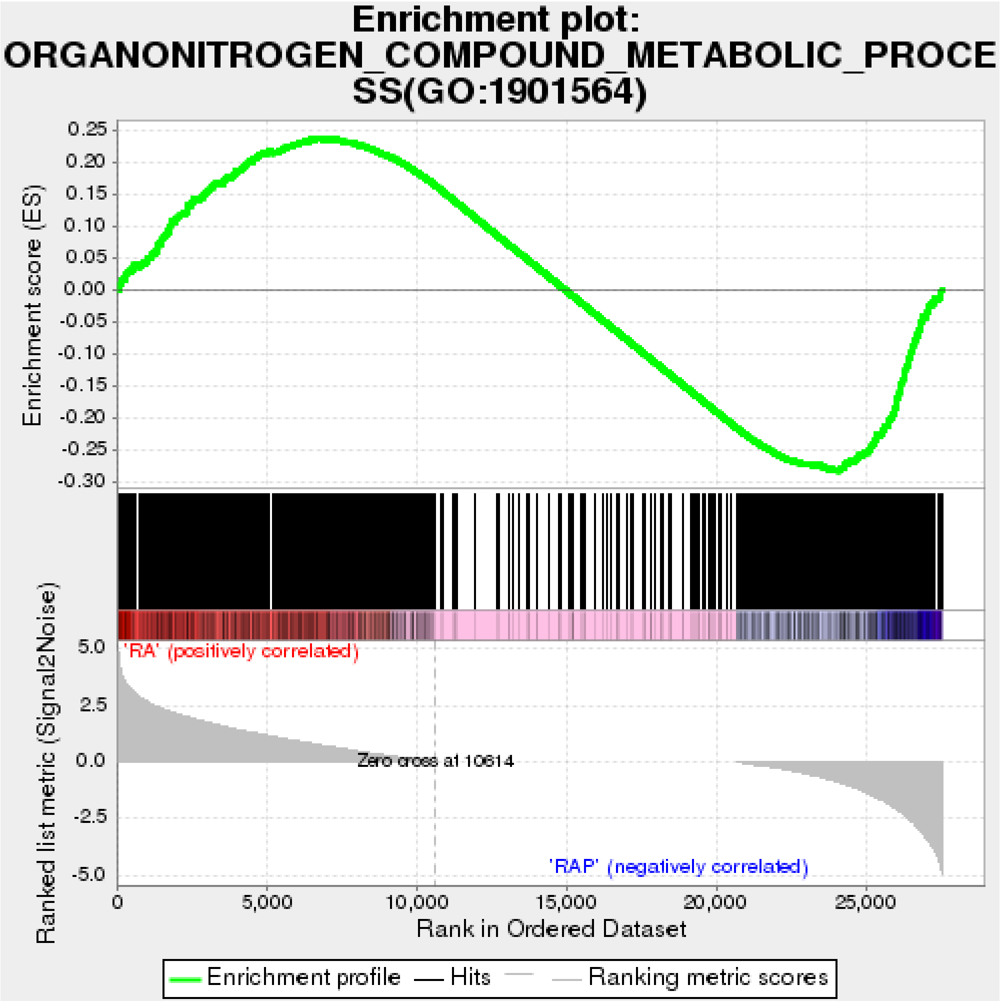

Fig 1: Enrichment plot: ORGANONITROGEN_COMPOUND_METABOLIC_PROCESS(GO:1901564)
Profile of the Running ES Score & Positions of GeneSet Members on the Rank Ordered List

Table: GSEA details ***[plain text format]***

	PROBE	DESCRIPTION (from dataset)	GENE SYMBOL	GENE_TITLE	RANK IN GENE LIST	RANK METRIC SCORE	RUNNING ES	CORE ENRICHMENT
1	PITG_02767	PITG_02767			21	4.420	0.0023	No
2	PITG_10138	PITG_10138			42	4.218	0.0046	No
3	PITG_13914	PITG_13914			46	4.202	0.0074	No
4	PITG_14993	PITG_14993			62	4.114	0.0097	No
5	PITG_05365	PITG_05365			92	3.970	0.0115	No
6	PITG_08784	PITG_08784			93	3.968	0.0143	No
7	PITG_00230	PITG_00230			100	3.937	0.0168	No
8	PITG_15917	PITG_15917			155	3.761	0.0174	No
9	PITG_08549	PITG_08549			216	3.578	0.0177	No
10	PITG_08725	PITG_08725			221	3.570	0.0200	No
11	PITG_05855	PITG_05855			222	3.567	0.0226	No
12	PITG_02821	PITG_02821			262	3.481	0.0236	No
13	PITG_03020	PITG_03020			267	3.466	0.0259	No
14	PITG_16203	PITG_16203			269	3.462	0.0283	No
15	PITG_02116	PITG_02116			319	3.377	0.0288	No
16	PITG_21116	PITG_21116			363	3.311	0.0295	No
17	PITG_01809	PITG_01809			397	3.257	0.0305	No
18	PITG_00245	PITG_00245			453	3.186	0.0307	No
19	PITG_07298	PITG_07298			455	3.183	0.0329	No
20	PITG_23349	PITG_23349			491	3.125	0.0338	No
21	PITG_06236	PITG_06236			495	3.120	0.0359	No
22	PITG_16904	PITG_16904			502	3.110	0.0379	No
23	PITG_06273	PITG_06273			527	3.082	0.0391	No
24	PITG_06684	PITG_06684			546	3.068	0.0406	No
25	PITG_04708	PITG_04708			676	2.910	0.0378	No
26	PITG_03941	PITG_03941			684	2.897	0.0396	No
27	PITG_03305	PITG_03305			742	2.845	0.0394	No
28	PITG_02050	PITG_02050			791	2.809	0.0396	No
29	PITG_10192	PITG_10192			806	2.795	0.0410	No
30	PITG_17359	PITG_17359			849	2.749	0.0414	No
31	PITG_13398	PITG_13398			862	2.738	0.0428	No
32	PITG_06928	PITG_06928			907	2.702	0.0431	No
33	PITG_15644	PITG_15644			936	2.687	0.0439	No
34	PITG_07248	PITG_07248			941	2.683	0.0457	No
35	PITG_00887	PITG_00887			955	2.675	0.0471	No
36	PITG_19121	PITG_19121			962	2.666	0.0487	No
37	PITG_00063	PITG_00063			971	2.658	0.0503	No

38	PITG_06607	PITG_06607			1010	2.632	0.0507	No
39	PITG_07157	PITG_07157			1045	2.609	0.0513	No
40	PITG_00614	PITG_00614			1082	2.580	0.0517	No
41	PITG_11470	PITG_11470			1102	2.567	0.0528	No
42	PITG_02757	PITG_02757			1125	2.546	0.0538	No
43	PITG_14396	PITG_14396			1130	2.544	0.0554	No
44	PITG_07210	PITG_07210			1152	2.526	0.0564	No
45	PITG_12186	PITG_12186			1174	2.511	0.0574	No
46	PITG_05953	PITG_05953			1190	2.503	0.0586	No
47	PITG_16204	PITG_16204			1233	2.475	0.0588	No
48	PITG_15981	PITG_15981			1246	2.466	0.0600	No
49	PITG_02710	PITG_02710			1264	2.460	0.0611	No
50	PITG_00416	PITG_00416			1278	2.453	0.0624	No
51	PITG_10623	PITG_10623			1285	2.448	0.0639	No
52	PITG_02224	PITG_02224			1296	2.441	0.0652	No
53	PITG_19589	PITG_19589			1304	2.436	0.0667	No
54	PITG_18262	PITG_18262			1313	2.433	0.0681	No
55	PITG_06288	PITG_06288			1317	2.430	0.0697	No
56	PITG_07215	PITG_07215			1326	2.427	0.0711	No
57	PITG_14626	PITG_14626			1350	2.418	0.0720	No
58	PITG_08008	PITG_08008			1359	2.411	0.0734	No
59	PITG_06963	PITG_06963			1390	2.393	0.0739	No
60	PITG_00588	PITG_00588			1428	2.377	0.0742	No
61	PITG_03110	PITG_03110			1431	2.376	0.0758	No
62	PITG_02997	PITG_02997			1447	2.370	0.0769	No
63	PITG_20767	PITG_20767			1452	2.368	0.0784	No
64	PITG_09671	PITG_09671			1465	2.362	0.0796	No
65	PITG_13752	PITG_13752			1470	2.358	0.0811	No
66	PITG_17661	PITG_17661			1472	2.355	0.0828	No
67	PITG_14393	PITG_14393			1488	2.348	0.0839	No
68	PITG_06937	PITG_06937			1513	2.334	0.0846	No
69	PITG_15001	PITG_15001			1525	2.330	0.0858	No
70	PITG_04421	PITG_04421			1584	2.308	0.0853	No
71	PITG_08774	PITG_08774			1588	2.306	0.0868	No
72	PITG_16603	PITG_16603			1593	2.303	0.0883	No
73	PITG_15216	PITG_15216			1613	2.295	0.0892	No
74	PITG_07724	PITG_07724			1629	2.288	0.0902	No
75	PITG_00124	PITG_00124			1630	2.288	0.0918	No
76	PITG_14634	PITG_14634			1656	2.276	0.0925	No

77	PITG_07828	PITG_07828			1690	2.257	0.0928	No
78	PITG_00081	PITG_00081			1702	2.253	0.0940	No
79	PITG_16476	PITG_16476			1703	2.253	0.0956	No
80	PITG_16636	PITG_16636			1706	2.252	0.0971	No
81	PITG_01936	PITG_01936			1712	2.250	0.0985	No
82	PITG_04478	PITG_04478			1728	2.246	0.0995	No
83	PITG_02177	PITG_02177			1729	2.246	0.1011	No
84	PITG_11204	PITG_11204			1741	2.243	0.1023	No
85	PITG_02960	PITG_02960			1747	2.241	0.1037	No
86	PITG_10270	PITG_10270			1748	2.241	0.1053	No
87	PITG_11607	PITG_11607			1757	2.236	0.1065	No
88	PITG_17577	PITG_17577			1772	2.231	0.1076	No
89	PITG_05548	PITG_05548			1799	2.219	0.1082	No
90	PITG_10119	PITG_10119			1864	2.190	0.1073	No
91	PITG_02565	PITG_02565			1894	2.177	0.1077	No
92	PITG_00761	PITG_00761			1898	2.175	0.1092	No
93	PITG_09400	PITG_09400			1906	2.172	0.1104	No
94	PITG_06022	PITG_06022			1925	2.161	0.1113	No
95	PITG_17361	PITG_17361			1929	2.159	0.1127	No
96	PITG_11016	PITG_11016			1939	2.152	0.1139	No
97	PITG_20163	PITG_20163			1992	2.129	0.1134	No
98	PITG_21313	PITG_21313			2003	2.123	0.1145	No
99	PITG_11807	PITG_11807			2017	2.118	0.1155	No
100	PITG_05798	PITG_05798			2038	2.108	0.1163	No
101	PITG_16537	PITG_16537			2054	2.100	0.1172	No
102	PITG_15089	PITG_15089			2073	2.092	0.1180	No
103	PITG_04133	PITG_04133			2094	2.084	0.1187	No
104	PITG_20204	PITG_20204			2109	2.079	0.1196	No
105	PITG_16069	PITG_16069			2157	2.060	0.1193	No
106	PITG_02384	PITG_02384			2180	2.053	0.1199	No
107	PITG_17012	PITG_17012			2198	2.047	0.1207	No
108	PITG_21606	PITG_21606			2199	2.046	0.1222	No
109	PITG_00211	PITG_00211			2260	2.025	0.1213	No
110	PITG_06481	PITG_06481			2265	2.024	0.1226	No
111	PITG_14703	PITG_14703			2294	2.010	0.1229	No
112	PITG_15611	PITG_15611			2300	2.008	0.1242	No
113	PITG_17314	PITG_17314			2309	2.003	0.1253	No
114	PITG_12090	PITG_12090			2311	2.003	0.1267	No
115	PITG_06280	PITG_06280			2319	1.997	0.1278	No

116	PITG_22671	PITG_22671			2322	1.995	0.1291	No
117	PITG_20560	PITG_20560			2332	1.990	0.1302	No
118	PITG_05358	PITG_05358			2334	1.989	0.1316	No
119	PITG_18578	PITG_18578			2335	1.989	0.1330	No
120	PITG_06480	PITG_06480			2344	1.986	0.1341	No
121	PITG_07251	PITG_07251			2385	1.971	0.1340	No
122	PITG_11919	PITG_11919			2424	1.958	0.1339	No
123	PITG_18256	PITG_18256			2432	1.956	0.1350	No
124	PITG_20589	PITG_20589			2458	1.941	0.1354	No
125	PITG_08042	PITG_08042			2459	1.940	0.1368	No
126	PITG_04619	PITG_04619			2472	1.933	0.1377	No
127	PITG_06889	PITG_06889			2489	1.927	0.1385	No
128	PITG_17897	PITG_17897			2509	1.918	0.1391	No
129	PITG_23143	PITG_23143			2510	1.917	0.1405	No
130	PITG_03934	PITG_03934			2517	1.916	0.1416	No
131	PITG_17585	PITG_17585			2530	1.910	0.1425	No
132	PITG_07302	PITG_07302			2561	1.898	0.1427	No
133	PITG_19445	PITG_19445			2636	1.870	0.1412	No
134	PITG_12646	PITG_12646			2647	1.865	0.1421	No
135	PITG_17584	PITG_17584			2687	1.847	0.1420	No
136	PITG_15256	PITG_15256			2725	1.834	0.1419	No
137	PITG_05261	PITG_05261			2728	1.832	0.1431	No
138	PITG_15301	PITG_15301			2751	1.825	0.1435	No
139	PITG_23090	PITG_23090			2754	1.823	0.1448	No
140	PITG_01453	PITG_01453			2775	1.816	0.1453	No
141	PITG_15619	PITG_15619			2806	1.806	0.1454	No
142	PITG_16916	PITG_16916			2817	1.802	0.1463	No
143	PITG_19310	PITG_19310			2829	1.796	0.1472	No
144	PITG_19878	PITG_19878			2831	1.796	0.1484	No
145	PITG_14392	PITG_14392			2855	1.788	0.1488	No
146	PITG_06355	PITG_06355			2861	1.785	0.1499	No
147	PITG_21941	PITG_21941			2865	1.784	0.1510	No
148	PITG_17579	PITG_17579			2914	1.767	0.1504	No
149	PITG_15892	PITG_15892			2930	1.761	0.1511	No
150	PITG_04477	PITG_04477			2931	1.759	0.1524	No
151	PITG_03585	PITG_03585			2955	1.754	0.1527	No
152	PITG_04458	PITG_04458			2977	1.746	0.1532	No
153	PITG_11728	PITG_11728			2986	1.744	0.1541	No
154	PITG_07154	PITG_07154			3002	1.739	0.1547	No

155	PITG_17126	PITG_17126			3003	1.737	0.1560	No
156	PITG_17506	PITG_17506			3026	1.730	0.1564	No
157	PITG_02423	PITG_02423			3061	1.721	0.1563	No
158	PITG_19472	PITG_19472			3063	1.720	0.1575	No
159	PITG_03584	PITG_03584			3072	1.718	0.1584	No
160	PITG_14707	PITG_14707			3076	1.717	0.1595	No
161	PITG_00636	PITG_00636			3084	1.716	0.1604	No
162	PITG_20766	PITG_20766			3100	1.711	0.1611	No
163	PITG_18359	PITG_18359			3110	1.709	0.1619	No
164	PITG_01409	PITG_01409			3123	1.703	0.1627	No
165	PITG_21806	PITG_21806			3175	1.686	0.1619	No
166	PITG_14994	PITG_14994			3180	1.685	0.1630	No
167	PITG_02110	PITG_02110			3182	1.685	0.1641	No
168	PITG_16214	PITG_16214			3184	1.684	0.1653	No
169	PITG_20772	PITG_20772			3205	1.675	0.1657	No
170	PITG_17508	PITG_17508			3235	1.667	0.1658	No
171	PITG_07866	PITG_07866			3250	1.662	0.1664	No
172	PITG_13024	PITG_13024			3262	1.657	0.1672	No
173	PITG_17879	PITG_17879			3293	1.648	0.1672	No
174	PITG_06639	PITG_06639			3343	1.633	0.1665	No
175	PITG_12002	PITG_12002			3344	1.633	0.1677	No
176	PITG_13814	PITG_13814			3426	1.607	0.1657	No
177	PITG_11926	PITG_11926			3471	1.592	0.1652	No
178	PITG_05817	PITG_05817			3489	1.586	0.1656	No
179	PITG_16585	PITG_16585			3494	1.584	0.1666	No
180	PITG_04682	PITG_04682			3499	1.582	0.1676	No
181	PITG_16074	PITG_16074			3507	1.580	0.1684	No
182	PITG_04971	PITG_04971			3511	1.578	0.1694	No
183	PITG_10830	PITG_10830			3520	1.575	0.1702	No
184	PITG_01450	PITG_01450			3527	1.572	0.1711	No
185	PITG_17583	PITG_17583			3530	1.571	0.1722	No
186	PITG_19939	PITG_19939			3538	1.567	0.1730	No
187	PITG_16016	PITG_16016			3545	1.565	0.1739	No
188	PITG_06279	PITG_06279			3573	1.557	0.1740	No
189	PITG_07235	PITG_07235			3584	1.553	0.1747	No
190	PITG_08411	PITG_08411			3613	1.543	0.1747	No
191	PITG_06796	PITG_06796			3624	1.539	0.1754	No
192	PITG_08375	PITG_08375			3638	1.533	0.1760	No
193	PITG_07978	PITG_07978			3641	1.533	0.1770	No

194	PITG_13488	PITG_13488			3647	1.531	0.1779	No
195	PITG_22022	PITG_22022			3666	1.525	0.1783	No
196	PITG_05762	PITG_05762			3733	1.505	0.1769	No
197	PITG_19572	PITG_19572			3770	1.495	0.1765	No
198	PITG_08587	PITG_08587			3772	1.495	0.1776	No
199	PITG_12140	PITG_12140			3794	1.490	0.1778	No
200	PITG_07349	PITG_07349			3799	1.488	0.1787	No
201	PITG_03055	PITG_03055			3818	1.485	0.1791	No
202	PITG_08358	PITG_08358			3842	1.477	0.1793	No
203	PITG_04393	PITG_04393			3843	1.477	0.1803	No
204	PITG_10831	PITG_10831			3875	1.468	0.1802	No
205	PITG_17576	PITG_17576			3880	1.467	0.1810	No
206	PITG_04255	PITG_04255			3907	1.458	0.1811	No
207	PITG_02288	PITG_02288			3912	1.457	0.1820	No
208	PITG_03060	PITG_03060			3914	1.456	0.1830	No
209	PITG_23166	PITG_23166			3918	1.456	0.1839	No
210	PITG_00178	PITG_00178			3920	1.455	0.1849	No
211	PITG_17292	PITG_17292			3922	1.455	0.1859	No
212	PITG_02264	PITG_02264			3925	1.454	0.1868	No
213	PITG_03497	PITG_03497			3978	1.439	0.1859	No
214	PITG_20491	PITG_20491			3983	1.439	0.1867	No
215	PITG_03409	PITG_03409			3987	1.438	0.1876	No
216	PITG_06925	PITG_06925			4021	1.428	0.1874	No
217	PITG_16473	PITG_16473			4033	1.426	0.1880	No
218	PITG_02071	PITG_02071			4065	1.415	0.1878	No
219	PITG_22487	PITG_22487			4081	1.413	0.1882	No
220	PITG_17333	PITG_17333			4092	1.410	0.1888	No
221	PITG_18274	PITG_18274			4095	1.410	0.1898	No
222	PITG_08572	PITG_08572			4117	1.407	0.1900	No
223	PITG_00430	PITG_00430			4119	1.406	0.1909	No
224	PITG_14968	PITG_14968			4148	1.395	0.1908	No
225	PITG_04405	PITG_04405			4161	1.391	0.1914	No
226	PITG_00177	PITG_00177			4178	1.386	0.1917	No
227	PITG_02666	PITG_02666			4180	1.385	0.1927	No
228	PITG_20156	PITG_20156			4190	1.381	0.1933	No
229	PITG_12322	PITG_12322			4195	1.380	0.1941	No
230	PITG_09119	PITG_09119			4230	1.373	0.1938	No
231	PITG_05781	PITG_05781			4239	1.372	0.1945	No
232	PITG_05340	PITG_05340			4245	1.371	0.1953	No

233	PITG_02457	PITG_02457			4249	1.370	0.1961	No
234	PITG_17705	PITG_17705			4276	1.362	0.1961	No
235	PITG_16088	PITG_16088			4287	1.359	0.1967	No
236	PITG_01262	PITG_01262			4293	1.357	0.1974	No
237	PITG_13415	PITG_13415			4322	1.349	0.1973	No
238	PITG_17343	PITG_17343			4326	1.348	0.1982	No
239	PITG_01188	PITG_01188			4341	1.345	0.1986	No
240	PITG_13074	PITG_13074			4351	1.343	0.1992	No
241	PITG_09402	PITG_09402			4368	1.339	0.1995	No
242	PITG_22381	PITG_22381			4369	1.338	0.2005	No
243	PITG_08304	PITG_08304			4381	1.335	0.2010	No
244	PITG_04568	PITG_04568			4388	1.334	0.2017	No
245	PITG_15003	PITG_15003			4408	1.329	0.2019	No
246	PITG_00654	PITG_00654			4410	1.329	0.2029	No
247	PITG_12475	PITG_12475			4443	1.322	0.2026	No
248	PITG_08899	PITG_08899			4487	1.310	0.2019	No
249	PITG_08753	PITG_08753			4499	1.308	0.2024	No
250	PITG_01526	PITG_01526			4501	1.307	0.2033	No
251	PITG_04715	PITG_04715			4505	1.306	0.2041	No
252	PITG_17840	PITG_17840			4509	1.306	0.2049	No
253	PITG_10488	PITG_10488			4514	1.305	0.2056	No
254	PITG_10448	PITG_10448			4532	1.302	0.2059	No
255	PITG_09938	PITG_09938			4546	1.298	0.2063	No
256	PITG_14808	PITG_14808			4552	1.297	0.2071	No
257	PITG_02114	PITG_02114			4601	1.283	0.2062	No
258	PITG_08348	PITG_08348			4611	1.282	0.2067	No
259	PITG_20211	PITG_20211			4613	1.281	0.2076	No
260	PITG_14156	PITG_14156			4621	1.277	0.2082	No
261	PITG_17575	PITG_17575			4645	1.272	0.2083	No
262	PITG_01006	PITG_01006			4655	1.270	0.2088	No
263	PITG_10239	PITG_10239			4664	1.268	0.2094	No
264	PITG_20007	PITG_20007			4686	1.263	0.2095	No
265	PITG_16360	PITG_16360			4689	1.262	0.2103	No
266	PITG_00298	PITG_00298			4696	1.261	0.2110	No
267	PITG_04938	PITG_04938			4697	1.260	0.2119	No
268	PITG_15596	PITG_15596			4712	1.256	0.2122	No
269	PITG_17711	PITG_17711			4741	1.249	0.2120	No
270	PITG_23141	PITG_23141			4747	1.247	0.2127	No
271	PITG_02489	PITG_02489			4763	1.242	0.2130	No

272	PITG_06195	PITG_06195			4788	1.235	0.2130	No
273	PITG_00858	PITG_00858			4824	1.224	0.2125	No
274	PITG_17651	PITG_17651			4826	1.224	0.2134	No
275	PITG_02546	PITG_02546			4833	1.222	0.2140	No
276	PITG_05862	PITG_05862			4841	1.220	0.2146	No
277	PITG_16644	PITG_16644			4904	1.204	0.2131	No
278	PITG_12259	PITG_12259			4905	1.204	0.2140	No
279	PITG_10857	PITG_10857			4907	1.203	0.2148	No
280	PITG_17942	PITG_17942			4908	1.203	0.2156	No
281	PITG_10780	PITG_10780			4962	1.191	0.2144	No
282	PITG_16276	PITG_16276			4963	1.191	0.2153	No
283	PITG_21189	PITG_21189			4993	1.184	0.2150	No
284	PITG_02857	PITG_02857			5002	1.181	0.2156	No
285	PITG_00004	PITG_00004			5010	1.179	0.2161	No
286	PITG_19773	PITG_19773			5023	1.176	0.2165	No
287	PITG_21504	PITG_21504			5024	1.176	0.2173	No
288	PITG_19450	PITG_19450			5045	1.170	0.2174	No
289	PITG_05374	PITG_05374			5055	1.167	0.2179	No
290	PITG_18027	PITG_18027			5065	1.164	0.2184	No
291	PITG_06708	PITG_06708			5161	1.133	0.2156	No
292	PITG_08439	PITG_08439			5208	1.123	0.2146	No
293	PITG_00218	PITG_00218			5210	1.122	0.2154	No
294	PITG_08570	PITG_08570			5212	1.122	0.2161	No
295	PITG_14598	PITG_14598			5228	1.119	0.2163	No
296	PITG_00525	PITG_00525			5267	1.109	0.2157	No
297	PITG_13832	PITG_13832			5312	1.100	0.2148	No
298	PITG_10601	PITG_10601			5327	1.097	0.2150	No
299	PITG_01012	PITG_01012			5338	1.094	0.2154	No
300	Novel00922	Novel00922			5355	1.090	0.2156	No
301	PITG_20634	PITG_20634			5357	1.090	0.2163	No
302	PITG_13347	PITG_13347			5358	1.090	0.2171	No
303	PITG_12516	PITG_12516			5360	1.090	0.2178	No
304	PITG_11431	PITG_11431			5374	1.086	0.2181	No
305	PITG_16618	PITG_16618			5375	1.085	0.2189	No
306	PITG_18687	PITG_18687			5401	1.079	0.2187	No
307	PITG_09375	PITG_09375			5421	1.074	0.2187	No
308	PITG_02119	PITG_02119			5447	1.067	0.2185	No
309	PITG_06885	PITG_06885			5449	1.066	0.2192	No
310	PITG_20272	PITG_20272			5472	1.061	0.2191	No

311	PITG_12194	PITG_12194			5481	1.058	0.2196	No
312	PITG_11304	PITG_11304			5503	1.051	0.2195	No
313	PITG_09010	PITG_09010			5506	1.050	0.2202	No
314	PITG_07916	PITG_07916			5517	1.047	0.2206	No
315	PITG_08967	PITG_08967			5518	1.046	0.2213	No
316	PITG_08810	PITG_08810			5519	1.046	0.2220	No
317	PITG_09407	PITG_09407			5552	1.039	0.2216	No
318	PITG_18255	PITG_18255			5566	1.034	0.2218	No
319	PITG_18649	PITG_18649			5569	1.034	0.2225	No
320	PITG_16616	PITG_16616			5586	1.032	0.2226	No
321	PITG_09393	PITG_09393			5588	1.031	0.2233	No
322	PITG_07652	PITG_07652			5612	1.024	0.2231	No
323	PITG_18266	PITG_18266			5621	1.022	0.2235	No
324	PITG_13164	PITG_13164			5635	1.018	0.2238	No
325	PITG_18279	PITG_18279			5653	1.012	0.2238	No
326	PITG_01851	PITG_01851			5663	1.009	0.2242	No
327	PITG_00570	PITG_00570			5672	1.008	0.2246	No
328	PITG_04457	PITG_04457			5679	1.007	0.2251	No
329	PITG_18271	PITG_18271			5685	1.005	0.2256	No
330	PITG_19872	PITG_19872			5700	1.002	0.2258	No
331	PITG_08425	PITG_08425			5753	0.993	0.2245	No
332	PITG_13934	PITG_13934			5768	0.991	0.2247	No
333	PITG_15776	PITG_15776			5781	0.987	0.2249	No
334	PITG_10821	PITG_10821			5785	0.986	0.2255	No
335	PITG_12293	PITG_12293			5803	0.981	0.2256	No
336	PITG_02762	PITG_02762			5804	0.981	0.2263	No
337	PITG_03015	PITG_03015			5820	0.978	0.2264	No
338	PITG_03522	PITG_03522			5830	0.977	0.2267	No
339	PITG_15774	PITG_15774			5847	0.973	0.2268	No
340	PITG_15629	PITG_15629			5866	0.969	0.2268	No
341	PITG_17791	PITG_17791			5876	0.967	0.2272	No
342	PITG_01043	PITG_01043			5891	0.964	0.2273	No
343	PITG_15298	PITG_15298			5902	0.962	0.2276	No
344	PITG_09251	PITG_09251			5906	0.962	0.2282	No
345	PITG_07656	PITG_07656			5945	0.952	0.2274	No
346	PITG_10032	PITG_10032			5953	0.951	0.2278	No
347	PITG_07191	PITG_07191			5960	0.951	0.2283	No
348	PITG_12155	PITG_12155			5999	0.940	0.2275	No
349	PITG_02400	PITG_02400			6007	0.938	0.2279	No

350	PITG_08004	PITG_08004			6032	0.932	0.2276	No
351	PITG_08471	PITG_08471			6049	0.929	0.2277	No
352	PITG_12916	PITG_12916			6050	0.928	0.2283	No
353	PITG_15735	PITG_15735			6059	0.926	0.2287	No
354	PITG_07234	PITG_07234			6061	0.926	0.2293	No
355	PITG_04226	PITG_04226			6063	0.926	0.2299	No
356	PITG_08901	PITG_08901			6070	0.925	0.2303	No
357	PITG_05865	PITG_05865			6084	0.920	0.2305	No
358	PITG_07164	PITG_07164			6086	0.920	0.2311	No
359	PITG_09039	PITG_09039			6087	0.920	0.2318	No
360	PITG_03513	PITG_03513			6108	0.915	0.2316	No
361	PITG_04474	PITG_04474			6115	0.914	0.2321	No
362	PITG_03006	PITG_03006			6134	0.911	0.2320	No
363	PITG_03860	PITG_03860			6144	0.908	0.2323	No
364	PITG_18473	PITG_18473			6194	0.897	0.2311	No
365	PITG_09665	PITG_09665			6199	0.896	0.2316	No
366	PITG_05240	PITG_05240			6207	0.894	0.2319	No
367	PITG_02442	PITG_02442			6227	0.890	0.2319	No
368	PITG_03945	PITG_03945			6232	0.888	0.2323	No
369	PITG_08900	PITG_08900			6247	0.885	0.2324	No
370	PITG_02529	PITG_02529			6254	0.883	0.2328	No
371	PITG_05730	PITG_05730			6273	0.878	0.2328	No
372	PITG_15970	PITG_15970			6300	0.872	0.2324	No
373	PITG_19875	PITG_19875			6301	0.872	0.2330	No
374	PITG_10998	PITG_10998			6313	0.868	0.2332	No
375	PITG_22686	PITG_22686			6319	0.864	0.2336	No
376	PITG_06832	PITG_06832			6329	0.862	0.2339	No
377	PITG_23089	PITG_23089			6371	0.852	0.2329	No
378	PITG_16056	PITG_16056			6373	0.852	0.2335	No
379	PITG_00176	PITG_00176			6375	0.852	0.2341	No
380	PITG_23044	PITG_23044			6377	0.851	0.2346	No
381	PITG_15307	PITG_15307			6389	0.849	0.2348	No
382	PITG_13680	PITG_13680			6406	0.844	0.2348	No
383	PITG_04254	PITG_04254			6442	0.836	0.2341	No
384	PITG_06223	PITG_06223			6445	0.835	0.2346	No
385	PITG_21223	PITG_21223			6447	0.834	0.2351	No
386	PITG_03660	PITG_03660			6456	0.832	0.2354	No
387	PITG_13301	PITG_13301			6465	0.830	0.2357	No
388	PITG_03049	PITG_03049			6472	0.828	0.2361	No

389	PITG_17990	PITG_17990			6490	0.825	0.2360	No
390	PITG_13997	PITG_13997			6498	0.824	0.2363	No
391	PITG_03703	PITG_03703			6521	0.815	0.2360	No
392	PITG_18277	PITG_18277			6539	0.812	0.2360	No
393	PITG_13043	PITG_13043			6540	0.812	0.2366	No
394	PITG_03415	PITG_03415			6541	0.811	0.2371	No
395	PITG_02465	PITG_02465			6604	0.799	0.2353	No
396	PITG_18265	PITG_18265			6610	0.798	0.2357	No
397	PITG_05487	PITG_05487			6613	0.797	0.2362	No
398	PITG_04225	PITG_04225			6625	0.795	0.2363	No
399	PITG_11875	PITG_11875			6633	0.794	0.2366	No
400	PITG_12099	PITG_12099			6636	0.794	0.2371	No
401	PITG_11102	PITG_11102			6647	0.791	0.2373	No
402	PITG_04678	PITG_04678			6663	0.787	0.2373	No
403	PITG_12903	PITG_12903			6664	0.787	0.2378	No
404	PITG_02724	PITG_02724			6679	0.782	0.2379	No
405	PITG_02211	PITG_02211			6700	0.777	0.2377	No
406	PITG_21400	PITG_21400			6735	0.767	0.2369	No
407	PITG_03075	PITG_03075			6750	0.764	0.2369	No
408	PITG_04589	PITG_04589			6780	0.757	0.2364	No
409	PITG_07055	PITG_07055			6787	0.756	0.2367	No
410	PITG_09260	PITG_09260			6796	0.753	0.2369	No
411	PITG_08414	PITG_08414			6858	0.738	0.2351	No
412	PITG_19456	PITG_19456			6860	0.737	0.2356	No
413	PITG_12041	PITG_12041			6876	0.733	0.2355	No
414	PITG_16210	PITG_16210			6886	0.730	0.2357	No
415	PITG_16734	PITG_16734			6944	0.719	0.2340	No
416	PITG_12105	PITG_12105			6948	0.718	0.2344	No
417	PITG_18045	PITG_18045			6958	0.716	0.2346	No
418	PITG_17295	PITG_17295			6973	0.713	0.2346	No
419	PITG_05653	PITG_05653			6984	0.710	0.2347	No
420	PITG_13298	PITG_13298			6989	0.709	0.2350	No
421	PITG_00257	PITG_00257			6992	0.708	0.2355	No
422	PITG_00279	PITG_00279			7003	0.707	0.2356	No
423	PITG_16446	PITG_16446			7024	0.703	0.2353	No
424	PITG_03634	PITG_03634			7030	0.702	0.2356	No
425	PITG_07995	PITG_07995			7040	0.700	0.2358	No
426	PITG_01653	PITG_01653			7045	0.699	0.2361	No
427	PITG_05338	PITG_05338			7061	0.696	0.2360	No

428	PITG_01480	PITG_01480			7068	0.695	0.2363	No
429	PITG_12094	PITG_12094			7071	0.695	0.2367	No
430	PITG_00187	PITG_00187			7074	0.694	0.2371	No
431	PITG_16057	PITG_16057			7082	0.692	0.2374	No
432	PITG_01920	PITG_01920			7120	0.685	0.2364	No
433	PITG_13421	PITG_13421			7130	0.683	0.2366	No
434	PITG_15417	PITG_15417			7136	0.681	0.2369	No
435	PITG_06282	PITG_06282			7182	0.673	0.2356	No
436	PITG_02446	PITG_02446			7188	0.672	0.2359	No
437	PITG_22427	PITG_22427			7202	0.668	0.2359	No
438	PITG_06231	PITG_06231			7203	0.668	0.2364	No
439	PITG_19429	PITG_19429			7229	0.661	0.2359	No
440	PITG_01832	PITG_01832			7265	0.653	0.2350	No
441	PITG_14699	PITG_14699			7272	0.652	0.2353	No
442	PITG_07317	PITG_07317			7296	0.645	0.2348	No
443	PITG_02393	PITG_02393			7297	0.645	0.2353	No
444	PITG_21395	PITG_21395			7298	0.645	0.2358	No
445	PITG_02401	PITG_02401			7305	0.643	0.2360	No
446	PITG_10899	PITG_10899			7307	0.642	0.2364	No
447	PITG_08736	PITG_08736			7341	0.633	0.2356	No
448	PITG_08760	PITG_08760			7366	0.629	0.2351	No
449	PITG_07160	PITG_07160			7401	0.622	0.2343	No
450	PITG_23142	PITG_23142			7412	0.620	0.2343	No
451	PITG_18275	PITG_18275			7421	0.617	0.2345	No
452	PITG_03709	PITG_03709			7471	0.605	0.2330	No
453	PITG_21617	PITG_21617			7481	0.603	0.2331	No
454	PITG_09092	PITG_09092			7493	0.600	0.2331	No
455	PITG_06379	PITG_06379			7505	0.598	0.2331	No
456	PITG_18999	PITG_18999			7507	0.598	0.2335	No
457	PITG_06019	PITG_06019			7537	0.591	0.2328	No
458	PITG_08312	PITG_08312			7564	0.587	0.2323	No
459	PITG_02212	PITG_02212			7566	0.587	0.2326	No
460	PITG_11566	PITG_11566			7603	0.579	0.2317	No
461	PITG_11126	PITG_11126			7609	0.577	0.2319	No
462	PITG_02561	PITG_02561			7633	0.570	0.2314	No
463	PITG_18276	PITG_18276			7651	0.567	0.2312	No
464	PITG_09550	PITG_09550			7673	0.562	0.2308	No
465	PITG_18272	PITG_18272			7695	0.557	0.2304	No
466	PITG_15603	PITG_15603			7701	0.556	0.2306	No

467	PITG_10645	PITG_10645			7705	0.554	0.2309	No
468	PITG_10847	PITG_10847			7711	0.553	0.2311	No
469	PITG_09508	PITG_09508			7726	0.551	0.2309	No
470	PITG_09706	PITG_09706			7731	0.550	0.2312	No
471	PITG_22479	PITG_22479			7741	0.548	0.2312	No
472	PITG_11236	PITG_11236			7770	0.543	0.2305	No
473	PITG_06942	PITG_06942			7794	0.537	0.2300	No
474	PITG_17663	PITG_17663			7799	0.536	0.2303	No
475	PITG_05587	PITG_05587			7800	0.535	0.2306	No
476	PITG_01447	PITG_01447			7833	0.527	0.2298	No
477	PITG_06724	PITG_06724			7835	0.527	0.2301	No
478	PITG_18258	PITG_18258			7838	0.526	0.2304	No
479	PITG_10513	PITG_10513			7844	0.525	0.2306	No
480	PITG_20746	PITG_20746			7846	0.525	0.2309	No
481	PITG_19932	PITG_19932			7852	0.524	0.2311	No
482	PITG_20103	PITG_20103			7856	0.523	0.2314	No
483	PITG_00077	PITG_00077			7911	0.509	0.2297	No
484	PITG_12160	PITG_12160			7915	0.507	0.2299	No
485	PITG_18064	PITG_18064			7932	0.505	0.2297	No
486	PITG_10077	PITG_10077			8008	0.487	0.2272	No
487	PITG_22989	PITG_22989			8038	0.480	0.2264	No
488	PITG_01343	PITG_01343			8046	0.477	0.2265	No
489	PITG_09620	PITG_09620			8057	0.474	0.2264	No
490	PITG_04506	PITG_04506			8063	0.473	0.2266	No
491	PITG_07031	PITG_07031			8080	0.470	0.2263	No
492	PITG_19318	PITG_19318			8087	0.469	0.2264	No
493	PITG_10080	PITG_10080			8090	0.468	0.2267	No
494	PITG_11525	PITG_11525			8098	0.466	0.2267	No
495	PITG_01856	PITG_01856			8109	0.464	0.2267	No
496	PITG_08761	PITG_08761			8111	0.464	0.2270	No
497	PITG_16137	PITG_16137			8118	0.462	0.2271	No
498	PITG_07910	PITG_07910			8133	0.460	0.2269	No
499	PITG_19869	PITG_19869			8155	0.454	0.2264	No
500	PITG_15569	PITG_15569			8171	0.451	0.2261	No
501	PITG_16461	PITG_16461			8203	0.447	0.2253	No
502	PITG_17926	PITG_17926			8224	0.443	0.2248	No
503	PITG_22892	PITG_22892			8237	0.440	0.2247	No
504	PITG_02711	PITG_02711			8239	0.439	0.2250	No
505	PITG_07156	PITG_07156			8246	0.437	0.2250	No

506	PITG_10652	PITG_10652			8265	0.433	0.2247	No
507	PITG_06199	PITG_06199			8267	0.432	0.2249	No
508	PITG_00543	PITG_00543			8271	0.430	0.2251	No
509	PITG_02291	PITG_02291			8341	0.418	0.2228	No
510	PITG_08599	PITG_08599			8350	0.416	0.2228	No
511	PITG_18257	PITG_18257			8371	0.409	0.2223	No
512	PITG_07153	PITG_07153			8390	0.403	0.2219	No
513	PITG_11524	PITG_11524			8404	0.400	0.2217	No
514	PITG_12540	PITG_12540			8430	0.395	0.2210	No
515	PITG_08669	PITG_08669			8433	0.394	0.2212	No
516	PITG_05483	PITG_05483			8436	0.394	0.2214	No
517	PITG_08756	PITG_08756			8440	0.393	0.2216	No
518	PITG_18298	PITG_18298			8466	0.386	0.2209	No
519	PITG_17832	PITG_17832			8489	0.380	0.2204	No
520	PITG_01314	PITG_01314			8512	0.375	0.2198	No
521	PITG_06880	PITG_06880			8530	0.371	0.2194	No
522	PITG_22662	PITG_22662			8561	0.364	0.2185	No
523	PITG_11630	PITG_11630			8572	0.362	0.2184	No
524	PITG_16055	PITG_16055			8579	0.360	0.2184	No
525	PITG_20964	PITG_20964			8581	0.359	0.2186	No
526	PITG_00783	PITG_00783			8593	0.356	0.2185	No
527	PITG_06685	PITG_06685			8614	0.354	0.2180	No
528	PITG_10516	PITG_10516			8635	0.351	0.2175	No
529	PITG_13130	PITG_13130			8649	0.348	0.2172	No
530	PITG_09870	PITG_09870			8666	0.345	0.2168	No
531	PITG_02226	PITG_02226			8678	0.342	0.2167	No
532	PITG_13172	PITG_13172			8693	0.339	0.2164	No
533	PITG_06738	PITG_06738			8723	0.334	0.2155	No
534	PITG_07851	PITG_07851			8733	0.331	0.2154	No
535	PITG_07809	PITG_07809			8735	0.331	0.2156	No
536	PITG_01072	PITG_01072			8755	0.328	0.2151	No
537	PITG_05112	PITG_05112			8790	0.321	0.2140	No
538	PITG_08890	PITG_08890			8792	0.321	0.2142	No
539	PITG_00471	PITG_00471			8794	0.321	0.2144	No
540	PITG_10045	PITG_10045			8821	0.318	0.2137	No
541	PITG_12181	PITG_12181			8823	0.317	0.2138	No
542	PITG_04677	PITG_04677			8848	0.312	0.2132	No
543	PITG_17703	PITG_17703			8849	0.312	0.2134	No
544	PITG_08898	PITG_08898			8855	0.311	0.2134	No

545	PITG_02394	PITG_02394			8866	0.309	0.2132	No
546	PITG_07354	PITG_07354			8869	0.309	0.2134	No
547	PITG_06926	PITG_06926			8909	0.301	0.2121	No
548	PITG_03150	PITG_03150			8939	0.297	0.2112	No
549	PITG_06749	PITG_06749			8954	0.294	0.2109	No
550	PITG_00194	PITG_00194			8957	0.294	0.2110	No
551	PITG_07731	PITG_07731			8960	0.293	0.2112	No
552	PITG_18347	PITG_18347			8984	0.289	0.2105	No
553	PITG_14557	PITG_14557			8993	0.287	0.2104	No
554	PITG_16977	PITG_16977			8995	0.287	0.2106	No
555	PITG_04724	PITG_04724			9015	0.282	0.2100	No
556	PITG_10675	PITG_10675			9023	0.281	0.2100	No
557	PITG_22264	PITG_22264			9030	0.280	0.2099	No
558	PITG_18863	PITG_18863			9032	0.280	0.2101	No
559	PITG_20808	PITG_20808			9047	0.278	0.2098	No
560	PITG_02474	PITG_02474			9051	0.277	0.2098	No
561	PITG_06107	PITG_06107			9055	0.276	0.2099	No
562	PITG_18278	PITG_18278			9070	0.273	0.2096	No
563	PITG_10049	PITG_10049			9085	0.268	0.2092	No
564	PITG_11486	PITG_11486			9105	0.264	0.2087	No
565	PITG_00640	PITG_00640			9114	0.261	0.2086	No
566	PITG_01950	PITG_01950			9124	0.261	0.2084	No
567	PITG_00633	PITG_00633			9157	0.254	0.2074	No
568	PITG_01389	PITG_01389			9158	0.254	0.2076	No
569	PITG_01445	PITG_01445			9161	0.253	0.2077	No
570	PITG_02854	PITG_02854			9178	0.250	0.2073	No
571	PITG_06118	PITG_06118			9187	0.249	0.2071	No
572	PITG_19535	PITG_19535			9205	0.246	0.2067	No
573	PITG_19235	PITG_19235			9220	0.244	0.2063	No
574	PITG_10008	PITG_10008			9222	0.243	0.2064	No
575	PITG_07242	PITG_07242			9237	0.241	0.2061	No
576	PITG_06015	PITG_06015			9260	0.235	0.2054	No
577	PITG_13991	PITG_13991			9286	0.230	0.2046	No
578	PITG_01777	PITG_01777			9292	0.229	0.2046	No
579	PITG_08806	PITG_08806			9306	0.227	0.2042	No
580	PITG_01002	PITG_01002			9313	0.226	0.2042	No
581	PITG_19849	PITG_19849			9344	0.219	0.2032	No
582	PITG_05886	PITG_05886			9352	0.218	0.2031	No
583	PITG_11752	PITG_11752			9353	0.218	0.2032	No

584	PITG_19041	PITG_19041			9359	0.217	0.2032	No
585	PITG_14835	PITG_14835			9384	0.213	0.2024	No
586	PITG_03901	PITG_03901			9415	0.207	0.2014	No
587	PITG_11798	PITG_11798			9425	0.207	0.2013	No
588	PITG_00952	PITG_00952			9444	0.205	0.2007	No
589	PITG_01087	PITG_01087			9454	0.203	0.2005	No
590	PITG_06774	PITG_06774			9458	0.202	0.2005	No
591	PITG_18799	PITG_18799			9510	0.194	0.1987	No
592	PITG_10760	PITG_10760			9553	0.187	0.1973	No
593	PITG_06259	PITG_06259			9554	0.186	0.1974	No
594	PITG_20600	PITG_20600			9568	0.184	0.1971	No
595	PITG_02750	PITG_02750			9577	0.183	0.1969	No
596	PITG_03456	PITG_03456			9585	0.180	0.1967	No
597	PITG_00296	PITG_00296			9594	0.179	0.1966	No
598	PITG_08876	PITG_08876			9598	0.179	0.1966	No
599	PITG_14137	PITG_14137			9623	0.176	0.1958	No
600	PITG_17502	PITG_17502			9624	0.176	0.1959	No
601	PITG_10829	PITG_10829			9640	0.173	0.1955	No
602	PITG_18303	PITG_18303			9750	0.151	0.1914	No
603	PITG_22582	PITG_22582			9771	0.146	0.1908	No
604	PITG_18296	PITG_18296			9785	0.145	0.1904	No
605	PITG_19148	PITG_19148			9803	0.142	0.1898	No
606	PITG_17133	PITG_17133			9824	0.138	0.1892	No
607	PITG_06776	PITG_06776			9828	0.136	0.1892	No
608	PITG_20584	PITG_20584			9839	0.133	0.1889	No
609	PITG_09394	PITG_09394			9840	0.133	0.1890	No
610	PITG_12517	PITG_12517			9843	0.133	0.1890	No
611	PITG_06505	PITG_06505			9858	0.131	0.1885	No
612	PITG_08210	PITG_08210			9867	0.130	0.1883	No
613	PITG_17550	PITG_17550			9876	0.128	0.1881	No
614	PITG_19488	PITG_19488			9886	0.126	0.1879	No
615	PITG_14380	PITG_14380			9888	0.126	0.1879	No
616	PITG_00319	PITG_00319			9949	0.117	0.1857	No
617	PITG_15982	PITG_15982			9960	0.115	0.1854	No
618	PITG_12122	PITG_12122			9962	0.114	0.1855	No
619	PITG_06964	PITG_06964			9965	0.114	0.1855	No
620	PITG_16184	PITG_16184			9975	0.113	0.1852	No
621	PITG_19294	PITG_19294			9991	0.110	0.1847	No
622	PITG_18261	PITG_18261			9998	0.109	0.1846	No

623	PITG_09791	PITG_09791			10045	0.103	0.1829	No
624	PITG_22959	PITG_22959			10054	0.102	0.1827	No
625	PITG_15890	PITG_15890			10070	0.100	0.1822	No
626	PITG_14920	PITG_14920			10087	0.097	0.1816	No
627	PITG_02136	PITG_02136			10102	0.094	0.1812	No
628	PITG_04611	PITG_04611			10105	0.093	0.1811	No
629	PITG_03220	PITG_03220			10124	0.090	0.1805	No
630	PITG_13564	PITG_13564			10128	0.089	0.1805	No
631	PITG_10003	PITG_10003			10164	0.082	0.1792	No
632	PITG_13458	PITG_13458			10199	0.074	0.1780	No
633	PITG_02992	PITG_02992			10209	0.072	0.1777	No
634	PITG_15000	PITG_15000			10212	0.072	0.1777	No
635	PITG_13648	PITG_13648			10232	0.068	0.1770	No
636	PITG_00254	PITG_00254			10255	0.067	0.1762	No
637	PITG_11626	PITG_11626			10256	0.067	0.1762	No
638	PITG_17945	PITG_17945			10277	0.064	0.1755	No
639	PITG_06817	PITG_06817			10278	0.064	0.1756	No
640	PITG_01203	PITG_01203			10285	0.063	0.1754	No
641	PITG_15382	PITG_15382			10300	0.061	0.1749	No
642	PITG_13913	PITG_13913			10307	0.059	0.1747	No
643	PITG_14228	PITG_14228			10328	0.053	0.1740	No
644	PITG_07830	PITG_07830			10356	0.049	0.1730	No
645	PITG_08452	PITG_08452			10395	0.040	0.1716	No
646	PITG_11569	PITG_11569			10405	0.039	0.1713	No
647	PITG_08014	PITG_08014			10406	0.039	0.1713	No
648	PITG_11178	PITG_11178			10422	0.034	0.1707	No
649	PITG_11999	PITG_11999			10434	0.031	0.1704	No
650	PITG_10139	PITG_10139			10456	0.026	0.1696	No
651	PITG_20562	PITG_20562			10475	0.024	0.1689	No
652	PITG_01193	PITG_01193			10499	0.023	0.1680	No
653	PITG_19905	PITG_19905			10534	0.016	0.1668	No
654	PITG_12482	PITG_12482			10538	0.016	0.1667	No
655	PITG_06775	PITG_06775			10567	0.012	0.1656	No
656	PITG_20640	PITG_20640			10808	0.000	0.1565	No
657	PITG_12509	PITG_12509			10849	0.000	0.1550	No
658	PITG_18980	PITG_18980			11172	0.000	0.1427	No
659	PITG_07481	PITG_07481			11257	0.000	0.1396	No
660	PITG_21243	PITG_21243			11281	0.000	0.1387	No
661	PITG_12629	PITG_12629			11341	0.000	0.1364	No

662	PITG_19346	PITG_19346			11914	0.000	0.1147	No
663	PITG_23338	PITG_23338			12635	0.000	0.0874	No
664	PITG_07182	PITG_07182			12702	0.000	0.0849	No
665	PITG_21582	PITG_21582			13034	0.000	0.0723	No
666	PITG_21586	PITG_21586			13035	0.000	0.0723	No
667	PITG_20824	PITG_20824			13172	0.000	0.0671	No
668	PITG_10610	PITG_10610			13398	0.000	0.0586	No
669	PITG_20161	PITG_20161			13646	0.000	0.0492	No
670	PITG_15722	PITG_15722			13740	0.000	0.0457	No
671	PITG_22058	PITG_22058			13753	0.000	0.0452	No
672	PITG_10666	PITG_10666			14000	0.000	0.0359	No
673	PITG_10100	PITG_10100			14003	0.000	0.0358	No
674	PITG_01091	PITG_01091			14376	0.000	0.0217	No
675	PITG_05812	PITG_05812			14754	0.000	0.0074	No
676	PITG_01017	PITG_01017			14766	0.000	0.0069	No
677	PITG_20131	PITG_20131			14790	0.000	0.0061	No
678	PITG_09431	PITG_09431			15054	0.000	-0.0039	No
679	PITG_18701	PITG_18701			15148	0.000	-0.0075	No
680	PITG_08191	PITG_08191			15194	0.000	-0.0092	No
681	PITG_07214	PITG_07214			15226	0.000	-0.0103	No
682	PITG_01013	PITG_01013			15237	0.000	-0.0107	No
683	PITG_01016	PITG_01016			15238	0.000	-0.0107	No
684	PITG_17187	PITG_17187			15487	0.000	-0.0201	No
685	PITG_06873	PITG_06873			15537	0.000	-0.0220	No
686	PITG_19379	PITG_19379			15632	0.000	-0.0256	No
687	PITG_19374	PITG_19374			15633	0.000	-0.0256	No
688	Novel00393	Novel00393			15971	0.000	-0.0384	No
689	PITG_22488	PITG_22488			16185	0.000	-0.0465	No
690	PITG_05162	PITG_05162			16359	0.000	-0.0530	No
691	PITG_21202	PITG_21202			16504	0.000	-0.0585	No
692	PITG_14310	PITG_14310			16667	0.000	-0.0647	No
693	PITG_14315	PITG_14315			16668	0.000	-0.0647	No
694	PITG_22801	PITG_22801			16709	0.000	-0.0662	No
695	PITG_16530	PITG_16530			17026	0.000	-0.0782	No
696	PITG_20587	PITG_20587			17149	0.000	-0.0828	No
697	PITG_02702	PITG_02702			17212	0.000	-0.0852	No
698	PITG_01007	PITG_01007			17512	0.000	-0.0965	No
699	PITG_16604	PITG_16604			17600	0.000	-0.0998	No
700	PITG_16601	PITG_16601			17601	0.000	-0.0998	No

701	PITG_14346	PITG_14346			17808	0.000	-0.1077	No
702	PITG_14344	PITG_14344			17809	0.000	-0.1077	No
703	PITG_10111	PITG_10111			17812	0.000	-0.1077	No
704	PITG_20943	PITG_20943			17928	0.000	-0.1121	No
705	PITG_18225	PITG_18225			18167	0.000	-0.1211	No
706	PITG_18226	PITG_18226			18168	0.000	-0.1211	No
707	PITG_21673	PITG_21673			18243	0.000	-0.1239	No
708	PITG_19256	PITG_19256			18405	0.000	-0.1301	No
709	PITG_05803	PITG_05803			18478	0.000	-0.1328	No
710	PITG_22629	PITG_22629			18497	0.000	-0.1335	No
711	PITG_14322	PITG_14322			18878	0.000	-0.1479	No
712	PITG_20240	PITG_20240			18905	0.000	-0.1489	No
713	PITG_21148	PITG_21148			19144	0.000	-0.1579	No
714	PITG_15474	PITG_15474			19220	0.000	-0.1608	No
715	PITG_12459	PITG_12459			19283	0.000	-0.1631	No
716	Novel01184	Novel01184			19348	0.000	-0.1656	No
717	PITG_03806	PITG_03806			19383	0.000	-0.1669	No
718	PITG_03807	PITG_03807			19384	0.000	-0.1669	No
719	PITG_07548	PITG_07548			19582	0.000	-0.1743	No
720	PITG_05850	PITG_05850			19624	0.000	-0.1759	No
721	PITG_03730	PITG_03730			19724	0.000	-0.1797	No
722	PITG_03731	PITG_03731			19725	0.000	-0.1797	No
723	PITG_19463	PITG_19463			19748	0.000	-0.1805	No
724	PITG_04498	PITG_04498			19789	0.000	-0.1820	No
725	PITG_18553	PITG_18553			19886	0.000	-0.1857	No
726	PITG_20405	PITG_20405			19919	0.000	-0.1869	No
727	PITG_04594	PITG_04594			20065	0.000	-0.1924	No
728	PITG_11927	PITG_11927			20106	0.000	-0.1939	No
729	PITG_21079	PITG_21079			20135	0.000	-0.1950	No
730	PITG_15977	PITG_15977			20158	0.000	-0.1958	No
731	PITG_07643	PITG_07643			20346	0.000	-0.2029	No
732	PITG_14325	PITG_14325			20368	0.000	-0.2037	No
733	PITG_21615	PITG_21615			20488	0.000	-0.2082	No
734	PITG_05086	PITG_05086			20667	0.000	-0.2150	No
735	PITG_03617	PITG_03617			20688	-0.000	-0.2157	No
736	PITG_07278	PITG_07278			20701	-0.004	-0.2162	No
737	PITG_00248	PITG_00248			20715	-0.008	-0.2167	No
738	PITG_21372	PITG_21372			20721	-0.009	-0.2169	No
739	PITG_22715	PITG_22715			20744	-0.014	-0.2177	No

740	PITG_05521	PITG_05521			20750	-0.015	-0.2179	No
741	PITG_16440	PITG_16440			20777	-0.020	-0.2188	No
742	PITG_14497	PITG_14497			20806	-0.026	-0.2199	No
743	PITG_06016	PITG_06016			20809	-0.027	-0.2199	No
744	PITG_21607	PITG_21607			20821	-0.031	-0.2203	No
745	PITG_03738	PITG_03738			20839	-0.035	-0.2210	No
746	PITG_12699	PITG_12699			20843	-0.036	-0.2210	No
747	PITG_17664	PITG_17664			20849	-0.037	-0.2212	No
748	PITG_17165	PITG_17165			20885	-0.048	-0.2225	No
749	PITG_11733	PITG_11733			20907	-0.054	-0.2233	No
750	PITG_19158	PITG_19158			20911	-0.055	-0.2233	No
751	PITG_12692	PITG_12692			20932	-0.059	-0.2241	No
752	PITG_14312	PITG_14312			20936	-0.059	-0.2241	No
753	PITG_11900	PITG_11900			20962	-0.064	-0.2250	No
754	PITG_09118	PITG_09118			20963	-0.064	-0.2250	No
755	PITG_11793	PITG_11793			20978	-0.068	-0.2255	No
756	PITG_07201	PITG_07201			20983	-0.069	-0.2256	No
757	PITG_01290	PITG_01290			20989	-0.070	-0.2257	No
758	PITG_05251	PITG_05251			20991	-0.071	-0.2257	No
759	PITG_06927	PITG_06927			21000	-0.072	-0.2260	No
760	PITG_21989	PITG_21989			21018	-0.077	-0.2266	No
761	PITG_02182	PITG_02182			21093	-0.091	-0.2293	No
762	PITG_06783	PITG_06783			21112	-0.095	-0.2299	No
763	PITG_02493	PITG_02493			21136	-0.100	-0.2307	No
764	PITG_13638	PITG_13638			21142	-0.100	-0.2308	No
765	PITG_13641	PITG_13641			21191	-0.111	-0.2326	No
766	PITG_06850	PITG_06850			21194	-0.111	-0.2326	No
767	PITG_07165	PITG_07165			21208	-0.114	-0.2330	No
768	PITG_18420	PITG_18420			21229	-0.118	-0.2337	No
769	PITG_03700	PITG_03700			21245	-0.122	-0.2341	No
770	PITG_10317	PITG_10317			21250	-0.123	-0.2342	No
771	Novel01760	Novel01760			21259	-0.125	-0.2344	No
772	PITG_08197	PITG_08197			21300	-0.130	-0.2359	No
773	PITG_04365	PITG_04365			21304	-0.131	-0.2359	No
774	PITG_01848	PITG_01848			21307	-0.131	-0.2359	No
775	PITG_05632	PITG_05632			21311	-0.132	-0.2359	No
776	PITG_05009	PITG_05009			21324	-0.134	-0.2362	No
777	PITG_17390	PITG_17390			21354	-0.139	-0.2372	No
778	PITG_10929	PITG_10929			21375	-0.142	-0.2379	No

779	PITG_00756	PITG_00756			21401	-0.147	-0.2388	No
780	PITG_04665	PITG_04665			21414	-0.151	-0.2391	No
781	PITG_13042	PITG_13042			21416	-0.151	-0.2390	No
782	PITG_00172	PITG_00172			21418	-0.151	-0.2390	No
783	PITG_17356	PITG_17356			21478	-0.164	-0.2411	No
784	PITG_22310	PITG_22310			21487	-0.165	-0.2413	No
785	PITG_13724	PITG_13724			21502	-0.169	-0.2417	No
786	PITG_01580	PITG_01580			21509	-0.170	-0.2418	No
787	PITG_17495	PITG_17495			21518	-0.171	-0.2420	No
788	PITG_21979	PITG_21979			21528	-0.172	-0.2422	No
789	PITG_05853	PITG_05853			21589	-0.178	-0.2444	No
790	PITG_21349	PITG_21349			21623	-0.186	-0.2455	No
791	PITG_18292	PITG_18292			21626	-0.186	-0.2454	No
792	PITG_00331	PITG_00331			21629	-0.187	-0.2454	No
793	PITG_01235	PITG_01235			21655	-0.190	-0.2462	No
794	PITG_10778	PITG_10778			21673	-0.192	-0.2467	No
795	PITG_06326	PITG_06326			21686	-0.195	-0.2470	No
796	PITG_00999	PITG_00999			21687	-0.195	-0.2469	No
797	PITG_14971	PITG_14971			21710	-0.201	-0.2476	No
798	PITG_08368	PITG_08368			21719	-0.202	-0.2477	No
799	PITG_11603	PITG_11603			21723	-0.203	-0.2477	No
800	PITG_10334	PITG_10334			21748	-0.208	-0.2485	No
801	PITG_14918	PITG_14918			21759	-0.210	-0.2487	No
802	PITG_11253	PITG_11253			21765	-0.211	-0.2487	No
803	PITG_12151	PITG_12151			21769	-0.212	-0.2487	No
804	PITG_02077	PITG_02077			21774	-0.213	-0.2487	No
805	PITG_13735	PITG_13735			21784	-0.214	-0.2489	No
806	PITG_17778	PITG_17778			21816	-0.219	-0.2499	No
807	PITG_00688	PITG_00688			21852	-0.227	-0.2511	No
808	PITG_18129	PITG_18129			21918	-0.242	-0.2534	No
809	PITG_10877	PITG_10877			21933	-0.245	-0.2537	No
810	PITG_02407	PITG_02407			21969	-0.252	-0.2549	No
811	PITG_03598	PITG_03598			21994	-0.254	-0.2556	No
812	PITG_11273	PITG_11273			22000	-0.255	-0.2556	No
813	PITG_16856	PITG_16856			22006	-0.258	-0.2556	No
814	PITG_21185	PITG_21185			22027	-0.261	-0.2562	No
815	PITG_22374	PITG_22374			22029	-0.262	-0.2561	No
816	PITG_08968	PITG_08968			22066	-0.269	-0.2572	No
817	PITG_23274	PITG_23274			22075	-0.271	-0.2573	No

818	PITG_06688	PITG_06688			22079	-0.271	-0.2573	No
819	PITG_17251	PITG_17251			22086	-0.273	-0.2573	No
820	PITG_03293	PITG_03293			22100	-0.276	-0.2576	No
821	PITG_10092	PITG_10092			22114	-0.278	-0.2579	No
822	PITG_17586	PITG_17586			22121	-0.280	-0.2579	No
823	PITG_10932	PITG_10932			22140	-0.287	-0.2584	No
824	PITG_18259	PITG_18259			22162	-0.292	-0.2590	No
825	PITG_00238	PITG_00238			22167	-0.294	-0.2589	No
826	PITG_05007	PITG_05007			22208	-0.303	-0.2603	No
827	PITG_10147	PITG_10147			22223	-0.306	-0.2606	No
828	PITG_08888	PITG_08888			22233	-0.309	-0.2607	No
829	PITG_23319	PITG_23319			22246	-0.312	-0.2609	No
830	PITG_01195	PITG_01195			22270	-0.321	-0.2616	No
831	PITG_06845	PITG_06845			22273	-0.322	-0.2614	No
832	PITG_20960	PITG_20960			22304	-0.331	-0.2623	No
833	PITG_17574	PITG_17574			22328	-0.336	-0.2630	No
834	PITG_01871	PITG_01871			22353	-0.342	-0.2636	No
835	PITG_14001	PITG_14001			22355	-0.342	-0.2634	No
836	PITG_01260	PITG_01260			22362	-0.344	-0.2634	No
837	PITG_03093	PITG_03093			22409	-0.359	-0.2649	No
838	PITG_07022	PITG_07022			22411	-0.359	-0.2647	No
839	PITG_03364	PITG_03364			22428	-0.364	-0.2650	No
840	PITG_02191	PITG_02191			22432	-0.365	-0.2649	No
841	PITG_03681	PITG_03681			22436	-0.366	-0.2647	No
842	PITG_02867	PITG_02867			22441	-0.367	-0.2646	No
843	PITG_05245	PITG_05245			22442	-0.368	-0.2644	No
844	PITG_12037	PITG_12037			22444	-0.368	-0.2642	No
845	PITG_18067	PITG_18067			22485	-0.377	-0.2654	No
846	PITG_17509	PITG_17509			22487	-0.378	-0.2652	No
847	PITG_02904	PITG_02904			22488	-0.378	-0.2649	No
848	PITG_13315	PITG_13315			22506	-0.381	-0.2653	No
849	PITG_16280	PITG_16280			22523	-0.387	-0.2656	No
850	PITG_14992	PITG_14992			22530	-0.389	-0.2656	No
851	PITG_14463	PITG_14463			22590	-0.403	-0.2675	No
852	PITG_07549	PITG_07549			22592	-0.403	-0.2673	No
853	PITG_04034	PITG_04034			22601	-0.404	-0.2673	No
854	PITG_13148	PITG_13148			22602	-0.404	-0.2670	No
855	PITG_10193	PITG_10193			22606	-0.404	-0.2668	No
856	PITG_13669	PITG_13669			22617	-0.407	-0.2669	No

857	PITG_07670	PITG_07670			22619	-0.407	-0.2667	No
858	PITG_19459	PITG_19459			22635	-0.410	-0.2670	No
859	PITG_13671	PITG_13671			22636	-0.410	-0.2667	No
860	PITG_08802	PITG_08802			22709	-0.436	-0.2691	No
861	PITG_16671	PITG_16671			22727	-0.443	-0.2694	No
862	PITG_12993	PITG_12993			22733	-0.445	-0.2693	No
863	PITG_17599	PITG_17599			22748	-0.448	-0.2695	No
864	PITG_00208	PITG_00208			22758	-0.451	-0.2696	No
865	PITG_11615	PITG_11615			22762	-0.452	-0.2693	No
866	PITG_11734	PITG_11734			22780	-0.455	-0.2697	No
867	PITG_11142	PITG_11142			22802	-0.459	-0.2701	No
868	PITG_01862	PITG_01862			22814	-0.464	-0.2702	No
869	PITG_10953	PITG_10953			22821	-0.465	-0.2701	No
870	PITG_20769	PITG_20769			22823	-0.465	-0.2698	No
871	PITG_03856	PITG_03856			22830	-0.469	-0.2697	No
872	PITG_03698	PITG_03698			22833	-0.469	-0.2695	No
873	PITG_00477	PITG_00477			22834	-0.470	-0.2691	No
874	PITG_09824	PITG_09824			22898	-0.487	-0.2712	No
875	PITG_16328	PITG_16328			22919	-0.491	-0.2716	No
876	PITG_22249	PITG_22249			22933	-0.496	-0.2718	No
877	PITG_23114	PITG_23114			22941	-0.498	-0.2717	No
878	PITG_07967	PITG_07967			22981	-0.512	-0.2728	No
879	PITG_15777	PITG_15777			22984	-0.513	-0.2725	No
880	PITG_09846	PITG_09846			22986	-0.514	-0.2722	No
881	PITG_09666	PITG_09666			22987	-0.514	-0.2718	No
882	PITG_17512	PITG_17512			22988	-0.514	-0.2714	No
883	PITG_07968	PITG_07968			23007	-0.521	-0.2718	No
884	PITG_11100	PITG_11100			23011	-0.522	-0.2715	No
885	PITG_09596	PITG_09596			23025	-0.527	-0.2716	No
886	PITG_06286	PITG_06286			23048	-0.534	-0.2721	No
887	PITG_02294	PITG_02294			23057	-0.537	-0.2720	No
888	PITG_10447	PITG_10447			23064	-0.539	-0.2719	No
889	PITG_07737	PITG_07737			23084	-0.545	-0.2722	No
890	PITG_17580	PITG_17580			23088	-0.546	-0.2719	No
891	PITG_19364	PITG_19364			23093	-0.548	-0.2717	No
892	PITG_16807	PITG_16807			23094	-0.548	-0.2713	No
893	PITG_10519	PITG_10519			23111	-0.552	-0.2715	No
894	PITG_03416	PITG_03416			23120	-0.554	-0.2714	No
895	PITG_00407	PITG_00407			23140	-0.560	-0.2718	No

896	PITG_15817	PITG_15817			23143	-0.561	-0.2714	No
897	PITG_12050	PITG_12050			23188	-0.576	-0.2727	No
898	PITG_05318	PITG_05318			23201	-0.581	-0.2727	No
899	PITG_00005	PITG_00005			23216	-0.586	-0.2729	No
900	PITG_12961	PITG_12961			23230	-0.590	-0.2729	No
901	PITG_07161	PITG_07161			23233	-0.590	-0.2726	No
902	PITG_00395	PITG_00395			23238	-0.592	-0.2723	No
903	PITG_14970	PITG_14970			23256	-0.595	-0.2725	No
904	PITG_13079	PITG_13079			23296	-0.605	-0.2736	No
905	PITG_03754	PITG_03754			23303	-0.608	-0.2734	No
906	PITG_00132	PITG_00132			23312	-0.612	-0.2733	No
907	PITG_04610	PITG_04610			23321	-0.613	-0.2731	No
908	PITG_16741	PITG_16741			23328	-0.615	-0.2729	No
909	PITG_08369	PITG_08369			23360	-0.629	-0.2737	No
910	PITG_05920	PITG_05920			23375	-0.634	-0.2738	No
911	PITG_04918	PITG_04918			23385	-0.637	-0.2736	No
912	PITG_07149	PITG_07149			23389	-0.638	-0.2733	No
913	PITG_00757	PITG_00757			23392	-0.640	-0.2729	No
914	PITG_02925	PITG_02925			23409	-0.645	-0.2731	No
915	PITG_17572	PITG_17572			23411	-0.645	-0.2727	No
916	PITG_20760	PITG_20760			23412	-0.645	-0.2722	No
917	PITG_02213	PITG_02213			23435	-0.652	-0.2726	No
918	PITG_03799	PITG_03799			23448	-0.655	-0.2726	No
919	PITG_00464	PITG_00464			23463	-0.659	-0.2726	No
920	PITG_02707	PITG_02707			23536	-0.686	-0.2749	No
921	PITG_12588	PITG_12588			23577	-0.708	-0.2759	No
922	PITG_05354	PITG_05354			23584	-0.710	-0.2756	No
923	PITG_07539	PITG_07539			23620	-0.722	-0.2765	No
924	PITG_02580	PITG_02580			23651	-0.734	-0.2771	No
925	PITG_08001	PITG_08001			23695	-0.749	-0.2782	No
926	PITG_08808	PITG_08808			23702	-0.751	-0.2779	No
927	PITG_02082	PITG_02082			23763	-0.769	-0.2796	No
928	PITG_01142	PITG_01142			23784	-0.778	-0.2798	No
929	PITG_16213	PITG_16213			23790	-0.779	-0.2795	No
930	PITG_23109	PITG_23109			23792	-0.779	-0.2789	No
931	PITG_17748	PITG_17748			23795	-0.780	-0.2785	No
932	PITG_06174	PITG_06174			23808	-0.786	-0.2784	No
933	PITG_22684	PITG_22684			23824	-0.793	-0.2784	No
934	PITG_11910	PITG_11910			23845	-0.800	-0.2786	No

935	PITG_17578	PITG_17578			23895	-0.819	-0.2799	No
936	PITG_20084	PITG_20084			23920	-0.830	-0.2802	No
937	PITG_19213	PITG_19213			23937	-0.838	-0.2802	No
938	PITG_21501	PITG_21501			23960	-0.846	-0.2804	No
939	PITG_00115	PITG_00115			23979	-0.853	-0.2805	No
940	PITG_17573	PITG_17573			24014	-0.863	-0.2812	No
941	PITG_12489	PITG_12489			24017	-0.864	-0.2807	No
942	PITG_03276	PITG_03276			24046	-0.871	-0.2811	No
943	PITG_21621	PITG_21621			24051	-0.873	-0.2806	No
944	PITG_08553	PITG_08553			24103	-0.901	-0.2819	Yes
945	PITG_00754	PITG_00754			24106	-0.902	-0.2814	Yes
946	PITG_21071	PITG_21071			24126	-0.913	-0.2815	Yes
947	PITG_19557	PITG_19557			24128	-0.914	-0.2808	Yes
948	PITG_06274	PITG_06274			24136	-0.916	-0.2805	Yes
949	PITG_14456	PITG_14456			24139	-0.918	-0.2799	Yes
950	PITG_02721	PITG_02721			24165	-0.930	-0.2802	Yes
951	PITG_09101	PITG_09101			24168	-0.931	-0.2796	Yes
952	PITG_03077	PITG_03077			24171	-0.933	-0.2790	Yes
953	PITG_13437	PITG_13437			24180	-0.938	-0.2787	Yes
954	PITG_04344	PITG_04344			24184	-0.940	-0.2781	Yes
955	PITG_15850	PITG_15850			24209	-0.953	-0.2783	Yes
956	PITG_19669	PITG_19669			24217	-0.958	-0.2779	Yes
957	PITG_06222	PITG_06222			24228	-0.961	-0.2776	Yes
958	PITG_06701	PITG_06701			24242	-0.966	-0.2774	Yes
959	PITG_07217	PITG_07217			24245	-0.966	-0.2768	Yes
960	PITG_03056	PITG_03056			24260	-0.973	-0.2767	Yes
961	PITG_20747	PITG_20747			24270	-0.976	-0.2763	Yes
962	PITG_05238	PITG_05238			24278	-0.979	-0.2759	Yes
963	PITG_01804	PITG_01804			24285	-0.982	-0.2754	Yes
964	PITG_01296	PITG_01296			24296	-0.987	-0.2751	Yes
965	PITG_08157	PITG_08157			24299	-0.988	-0.2745	Yes
966	PITG_17410	PITG_17410			24300	-0.988	-0.2738	Yes
967	PITG_11111	PITG_11111			24301	-0.988	-0.2731	Yes
968	PITG_05733	PITG_05733			24305	-0.989	-0.2725	Yes
969	PITG_02124	PITG_02124			24309	-0.991	-0.2719	Yes
970	PITG_13069	PITG_13069			24318	-0.995	-0.2715	Yes
971	PITG_01036	PITG_01036			24321	-0.997	-0.2709	Yes
972	Novel01790	Novel01790			24331	-1.001	-0.2705	Yes
973	PITG_06979	PITG_06979			24340	-1.004	-0.2701	Yes

974	PITG_17153	PITG_17153			24348	-1.006	-0.2697	Yes
975	PITG_01762	PITG_01762			24386	-1.026	-0.2704	Yes
976	PITG_15294	PITG_15294			24402	-1.032	-0.2702	Yes
977	PITG_16757	PITG_16757			24413	-1.036	-0.2698	Yes
978	PITG_03813	PITG_03813			24420	-1.039	-0.2693	Yes
979	PITG_03643	PITG_03643			24432	-1.043	-0.2690	Yes
980	PITG_08000	PITG_08000			24442	-1.049	-0.2686	Yes
981	PITG_15457	PITG_15457			24452	-1.054	-0.2682	Yes
982	PITG_18251	PITG_18251			24457	-1.057	-0.2676	Yes
983	PITG_03322	PITG_03322			24462	-1.057	-0.2670	Yes
984	PITG_11909	PITG_11909			24479	-1.063	-0.2669	Yes
985	PITG_00997	PITG_00997			24484	-1.064	-0.2663	Yes
986	PITG_22572	PITG_22572			24528	-1.084	-0.2671	Yes
987	PITG_15015	PITG_15015			24533	-1.085	-0.2665	Yes
988	PITG_09698	PITG_09698			24544	-1.090	-0.2661	Yes
989	PITG_10979	PITG_10979			24567	-1.100	-0.2662	Yes
990	PITG_09635	PITG_09635			24639	-1.133	-0.2681	Yes
991	PITG_04698	PITG_04698			24645	-1.135	-0.2675	Yes
992	PITG_10777	PITG_10777			24660	-1.145	-0.2672	Yes
993	PITG_07797	PITG_07797			24662	-1.147	-0.2664	Yes
994	PITG_07888	PITG_07888			24666	-1.148	-0.2657	Yes
995	PITG_12514	PITG_12514			24671	-1.152	-0.2651	Yes
996	PITG_05649	PITG_05649			24675	-1.154	-0.2644	Yes
997	PITG_00221	PITG_00221			24677	-1.155	-0.2636	Yes
998	PITG_08984	PITG_08984			24689	-1.162	-0.2632	Yes
999	PITG_07274	PITG_07274			24703	-1.168	-0.2628	Yes
1000	PITG_03274	PITG_03274			24715	-1.175	-0.2624	Yes
1001	PITG_01576	PITG_01576			24723	-1.176	-0.2619	Yes
1002	PITG_13512	PITG_13512			24729	-1.180	-0.2612	Yes
1003	PITG_14609	PITG_14609			24732	-1.182	-0.2605	Yes
1004	PITG_05405	PITG_05405			24742	-1.189	-0.2600	Yes
1005	PITG_07841	PITG_07841			24743	-1.190	-0.2591	Yes
1006	PITG_04703	PITG_04703			24756	-1.196	-0.2587	Yes
1007	PITG_14765	PITG_14765			24768	-1.201	-0.2583	Yes
1008	PITG_08155	PITG_08155			24778	-1.206	-0.2578	Yes
1009	PITG_19999	PITG_19999			24792	-1.214	-0.2574	Yes
1010	PITG_12864	PITG_12864			24801	-1.219	-0.2569	Yes
1011	PITG_10450	PITG_10450			24832	-1.237	-0.2571	Yes
1012	PITG_04207	PITG_04207			24854	-1.247	-0.2570	Yes

1013	PITG_20771	PITG_20771			24882	-1.270	-0.2572	Yes
1014	PITG_02397	PITG_02397			24921	-1.290	-0.2577	Yes
1015	PITG_03480	PITG_03480			24930	-1.293	-0.2571	Yes
1016	PITG_07535	PITG_07535			24931	-1.295	-0.2562	Yes
1017	PITG_16366	PITG_16366			24945	-1.308	-0.2558	Yes
1018	PITG_20189	PITG_20189			24966	-1.317	-0.2556	Yes
1019	PITG_00566	PITG_00566			24968	-1.317	-0.2547	Yes
1020	PITG_17500	PITG_17500			24974	-1.319	-0.2539	Yes
1021	PITG_02429	PITG_02429			24988	-1.327	-0.2535	Yes
1022	PITG_01922	PITG_01922			25002	-1.336	-0.2530	Yes
1023	PITG_12839	PITG_12839			25031	-1.352	-0.2532	Yes
1024	PITG_14850	PITG_14850			25066	-1.380	-0.2535	Yes
1025	PITG_06191	PITG_06191			25082	-1.391	-0.2531	Yes
1026	PITG_11923	PITG_11923			25096	-1.398	-0.2526	Yes
1027	PITG_02672	PITG_02672			25104	-1.401	-0.2518	Yes
1028	PITG_16008	PITG_16008			25114	-1.410	-0.2512	Yes
1029	PITG_06771	PITG_06771			25118	-1.413	-0.2503	Yes
1030	PITG_13655	PITG_13655			25132	-1.424	-0.2498	Yes
1031	PITG_04838	PITG_04838			25152	-1.435	-0.2495	Yes
1032	PITG_15723	PITG_15723			25159	-1.438	-0.2487	Yes
1033	PITG_13420	PITG_13420			25161	-1.439	-0.2477	Yes
1034	PITG_07141	PITG_07141			25165	-1.441	-0.2468	Yes
1035	PITG_04992	PITG_04992			25172	-1.445	-0.2460	Yes
1036	PITG_06595	PITG_06595			25183	-1.453	-0.2454	Yes
1037	PITG_00443	PITG_00443			25193	-1.456	-0.2447	Yes
1038	PITG_07671	PITG_07671			25198	-1.458	-0.2438	Yes
1039	PITG_21854	PITG_21854			25207	-1.466	-0.2431	Yes
1040	PITG_04843	PITG_04843			25217	-1.471	-0.2424	Yes
1041	PITG_18053	PITG_18053			25243	-1.483	-0.2423	Yes
1042	PITG_17947	PITG_17947			25248	-1.485	-0.2414	Yes
1043	PITG_21299	PITG_21299			25256	-1.496	-0.2406	Yes
1044	PITG_03498	PITG_03498			25263	-1.500	-0.2398	Yes
1045	PITG_09699	PITG_09699			25275	-1.508	-0.2391	Yes
1046	PITG_07028	PITG_07028			25277	-1.509	-0.2381	Yes
1047	PITG_05523	PITG_05523			25293	-1.519	-0.2376	Yes
1048	PITG_09582	PITG_09582			25294	-1.519	-0.2365	Yes
1049	PITG_06021	PITG_06021			25318	-1.533	-0.2363	Yes
1050	PITG_20965	PITG_20965			25323	-1.536	-0.2354	Yes
1051	PITG_02700	PITG_02700			25326	-1.537	-0.2343	Yes

1052	PITG_07725	PITG_07725			25339	-1.548	-0.2337	Yes
1053	PITG_05171	PITG_05171			25340	-1.548	-0.2326	Yes
1054	PITG_12745	PITG_12745			25347	-1.553	-0.2317	Yes
1055	PITG_11116	PITG_11116			25350	-1.554	-0.2307	Yes
1056	PITG_10974	PITG_10974			25362	-1.559	-0.2300	Yes
1057	PITG_09553	PITG_09553			25363	-1.561	-0.2289	Yes
1058	PITG_07792	PITG_07792			25364	-1.561	-0.2278	Yes
1059	PITG_13860	PITG_13860			25367	-1.563	-0.2268	Yes
1060	PITG_08206	PITG_08206			25382	-1.567	-0.2262	Yes
1061	PITG_09664	PITG_09664			25391	-1.577	-0.2254	Yes
1062	PITG_02858	PITG_02858			25402	-1.582	-0.2247	Yes
1063	PITG_18073	PITG_18073			25456	-1.622	-0.2255	Yes
1064	PITG_16646	PITG_16646			25484	-1.642	-0.2254	Yes
1065	PITG_20188	PITG_20188			25502	-1.654	-0.2249	Yes
1066	PITG_12697	PITG_12697			25529	-1.673	-0.2247	Yes
1067	PITG_12930	PITG_12930			25557	-1.688	-0.2245	Yes
1068	PITG_15526	PITG_15526			25559	-1.690	-0.2234	Yes
1069	PITG_04522	PITG_04522			25562	-1.692	-0.2222	Yes
1070	PITG_10062	PITG_10062			25575	-1.699	-0.2215	Yes
1071	PITG_04729	PITG_04729			25577	-1.699	-0.2203	Yes
1072	PITG_05851	PITG_05851			25578	-1.700	-0.2191	Yes
1073	PITG_09234	PITG_09234			25595	-1.710	-0.2185	Yes
1074	PITG_10887	PITG_10887			25597	-1.713	-0.2173	Yes
1075	PITG_07300	PITG_07300			25611	-1.727	-0.2166	Yes
1076	PITG_03221	PITG_03221			25613	-1.729	-0.2154	Yes
1077	PITG_00073	PITG_00073			25631	-1.738	-0.2148	Yes
1078	PITG_04922	PITG_04922			25636	-1.741	-0.2138	Yes
1079	PITG_15090	PITG_15090			25650	-1.757	-0.2130	Yes
1080	PITG_04774	PITG_04774			25653	-1.761	-0.2119	Yes
1081	PITG_19428	PITG_19428			25674	-1.776	-0.2114	Yes
1082	PITG_08703	PITG_08703			25716	-1.807	-0.2116	Yes
1083	PITG_02080	PITG_02080			25743	-1.834	-0.2113	Yes
1084	PITG_01245	PITG_01245			25744	-1.834	-0.2100	Yes
1085	PITG_01255	PITG_01255			25748	-1.835	-0.2088	Yes
1086	PITG_13513	PITG_13513			25757	-1.841	-0.2078	Yes
1087	PITG_10110	PITG_10110			25769	-1.847	-0.2070	Yes
1088	PITG_02694	PITG_02694			25772	-1.849	-0.2057	Yes
1089	PITG_15069	PITG_15069			25785	-1.859	-0.2049	Yes
1090	PITG_01943	PITG_01943			25791	-1.864	-0.2037	Yes

1091	PITG_15407	PITG_15407			25795	-1.868	-0.2025	Yes
1092	PITG_10089	PITG_10089			25810	-1.878	-0.2017	Yes
1093	PITG_11766	PITG_11766			25814	-1.881	-0.2005	Yes
1094	PITG_01833	PITG_01833			25831	-1.897	-0.1998	Yes
1095	PITG_02921	PITG_02921			25864	-1.929	-0.1996	Yes
1096	PITG_13586	PITG_13586			25869	-1.931	-0.1984	Yes
1097	PITG_03775	PITG_03775			25881	-1.944	-0.1975	Yes
1098	PITG_17024	PITG_17024			25894	-1.952	-0.1965	Yes
1099	PITG_08579	PITG_08579			25907	-1.965	-0.1956	Yes
1100	PITG_14972	PITG_14972			25919	-1.970	-0.1946	Yes
1101	PITG_17501	PITG_17501			25921	-1.970	-0.1933	Yes
1102	PITG_20264	PITG_20264			25950	-1.992	-0.1929	Yes
1103	PITG_09576	PITG_09576			25964	-2.006	-0.1920	Yes
1104	PITG_00632	PITG_00632			25974	-2.018	-0.1909	Yes
1105	PITG_18270	PITG_18270			25976	-2.021	-0.1895	Yes
1106	PITG_07173	PITG_07173			25980	-2.022	-0.1882	Yes
1107	PITG_19157	PITG_19157			25981	-2.022	-0.1868	Yes
1108	PITG_10863	PITG_10863			25982	-2.022	-0.1854	Yes
1109	PITG_03460	PITG_03460			25987	-2.027	-0.1841	Yes
1110	PITG_09540	PITG_09540			25997	-2.035	-0.1830	Yes
1111	PITG_20559	PITG_20559			26014	-2.048	-0.1821	Yes
1112	PITG_03661	PITG_03661			26015	-2.048	-0.1807	Yes
1113	PITG_14936	PITG_14936			26019	-2.053	-0.1794	Yes
1114	PITG_03353	PITG_03353			26026	-2.059	-0.1781	Yes
1115	PITG_19676	PITG_19676			26028	-2.059	-0.1767	Yes
1116	PITG_13831	PITG_13831			26029	-2.061	-0.1752	Yes
1117	PITG_09619	PITG_09619			26034	-2.064	-0.1739	Yes
1118	PITG_09640	PITG_09640			26046	-2.074	-0.1729	Yes
1119	PITG_09506	PITG_09506			26047	-2.074	-0.1714	Yes
1120	PITG_00941	PITG_00941			26052	-2.078	-0.1701	Yes
1121	PITG_13397	PITG_13397			26053	-2.079	-0.1686	Yes
1122	PITG_14913	PITG_14913			26073	-2.104	-0.1679	Yes
1123	PITG_04487	PITG_04487			26098	-2.129	-0.1673	Yes
1124	PITG_00302	PITG_00302			26103	-2.131	-0.1659	Yes
1125	PITG_04747	PITG_04747			26111	-2.138	-0.1647	Yes
1126	PITG_03414	PITG_03414			26114	-2.141	-0.1632	Yes
1127	PITG_00074	PITG_00074			26129	-2.164	-0.1622	Yes
1128	PITG_03235	PITG_03235			26130	-2.165	-0.1607	Yes
1129	PITG_19531	PITG_19531			26134	-2.169	-0.1593	Yes

1130	PITG_04382	PITG_04382			26147	-2.181	-0.1582	Yes
1131	PITG_06518	PITG_06518			26151	-2.183	-0.1568	Yes
1132	PITG_14969	PITG_14969			26161	-2.192	-0.1556	Yes
1133	PITG_06995	PITG_06995			26169	-2.197	-0.1543	Yes
1134	PITG_08959	PITG_08959			26174	-2.203	-0.1529	Yes
1135	PITG_05174	PITG_05174			26178	-2.205	-0.1514	Yes
1136	PITG_03239	PITG_03239			26180	-2.206	-0.1499	Yes
1137	PITG_17582	PITG_17582			26182	-2.207	-0.1484	Yes
1138	PITG_04910	PITG_04910			26183	-2.209	-0.1468	Yes
1139	PITG_01769	PITG_01769			26199	-2.231	-0.1458	Yes
1140	PITG_00646	PITG_00646			26202	-2.237	-0.1443	Yes
1141	PITG_03294	PITG_03294			26205	-2.241	-0.1428	Yes
1142	PITG_07405	PITG_07405			26228	-2.258	-0.1420	Yes
1143	PITG_06175	PITG_06175			26229	-2.258	-0.1404	Yes
1144	PITG_09555	PITG_09555			26249	-2.286	-0.1395	Yes
1145	PITG_13399	PITG_13399			26268	-2.305	-0.1386	Yes
1146	Novel00015	Novel00015			26276	-2.309	-0.1372	Yes
1147	PITG_17785	PITG_17785			26278	-2.310	-0.1356	Yes
1148	PITG_09547	PITG_09547			26285	-2.315	-0.1342	Yes
1149	PITG_04418	PITG_04418			26293	-2.325	-0.1328	Yes
1150	PITG_12947	PITG_12947			26294	-2.326	-0.1312	Yes
1151	PITG_18052	PITG_18052			26295	-2.327	-0.1296	Yes
1152	PITG_22112	PITG_22112			26302	-2.335	-0.1281	Yes
1153	PITG_16339	PITG_16339			26327	-2.364	-0.1274	Yes
1154	PITG_04683	PITG_04683			26329	-2.368	-0.1257	Yes
1155	PITG_12077	PITG_12077			26335	-2.375	-0.1242	Yes
1156	PITG_13371	PITG_13371			26336	-2.376	-0.1226	Yes
1157	PITG_06636	PITG_06636			26348	-2.389	-0.1213	Yes
1158	PITG_17252	PITG_17252			26351	-2.394	-0.1197	Yes
1159	PITG_01694	PITG_01694			26388	-2.432	-0.1193	Yes
1160	PITG_03178	PITG_03178			26392	-2.437	-0.1177	Yes
1161	PITG_00523	PITG_00523			26405	-2.460	-0.1164	Yes
1162	PITG_08002	PITG_08002			26423	-2.484	-0.1153	Yes
1163	PITG_01824	PITG_01824			26440	-2.501	-0.1142	Yes
1164	PITG_09726	PITG_09726			26447	-2.506	-0.1126	Yes
1165	PITG_09631	PITG_09631			26454	-2.515	-0.1111	Yes
1166	PITG_14195	PITG_14195			26456	-2.517	-0.1093	Yes
1167	PITG_13681	PITG_13681			26460	-2.519	-0.1077	Yes
1168	PITG_13732	PITG_13732			26464	-2.528	-0.1060	Yes

1169	PITG_08129	PITG_08129			26476	-2.540	-0.1046	Yes
1170	PITG_04419	PITG_04419			26491	-2.550	-0.1033	Yes
1171	PITG_03672	PITG_03672			26510	-2.566	-0.1022	Yes
1172	PITG_10146	PITG_10146			26517	-2.576	-0.1006	Yes
1173	PITG_17300	PITG_17300			26519	-2.576	-0.0988	Yes
1174	PITG_00643	PITG_00643			26529	-2.591	-0.0973	Yes
1175	PITG_17571	PITG_17571			26537	-2.595	-0.0958	Yes
1176	PITG_17357	PITG_17357			26540	-2.598	-0.0940	Yes
1177	PITG_02578	PITG_02578			26546	-2.608	-0.0923	Yes
1178	PITG_06237	PITG_06237			26557	-2.626	-0.0909	Yes
1179	PITG_00910	PITG_00910			26570	-2.639	-0.0895	Yes
1180	PITG_17607	PITG_17607			26591	-2.668	-0.0883	Yes
1181	PITG_09851	PITG_09851			26595	-2.670	-0.0866	Yes
1182	PITG_03420	PITG_03420			26604	-2.687	-0.0850	Yes
1183	PITG_01042	PITG_01042			26613	-2.704	-0.0834	Yes
1184	PITG_17289	PITG_17289			26618	-2.713	-0.0816	Yes
1185	PITG_03999	PITG_03999			26623	-2.720	-0.0798	Yes
1186	PITG_09521	PITG_09521			26652	-2.752	-0.0789	Yes
1187	PITG_02039	PITG_02039			26671	-2.781	-0.0777	Yes
1188	PITG_12764	PITG_12764			26699	-2.823	-0.0767	Yes
1189	PITG_09552	PITG_09552			26705	-2.829	-0.0749	Yes
1190	PITG_14729	PITG_14729			26712	-2.839	-0.0731	Yes
1191	PITG_21456	PITG_21456			26719	-2.845	-0.0713	Yes
1192	PITG_04348	PITG_04348			26729	-2.869	-0.0696	Yes
1193	PITG_16736	PITG_16736			26755	-2.913	-0.0685	Yes
1194	PITG_17507	PITG_17507			26758	-2.915	-0.0665	Yes
1195	PITG_07991	PITG_07991			26768	-2.940	-0.0648	Yes
1196	PITG_00276	PITG_00276			26776	-2.957	-0.0630	Yes
1197	PITG_03773	PITG_03773			26795	-2.981	-0.0615	Yes
1198	PITG_00397	PITG_00397			26815	-3.015	-0.0601	Yes
1199	PITG_01849	PITG_01849			26831	-3.040	-0.0585	Yes
1200	PITG_03768	PITG_03768			26845	-3.062	-0.0569	Yes
1201	PITG_06821	PITG_06821			26849	-3.067	-0.0548	Yes
1202	PITG_05377	PITG_05377			26850	-3.068	-0.0526	Yes
1203	PITG_15616	PITG_15616			26858	-3.085	-0.0507	Yes
1204	PITG_08714	PITG_08714			26862	-3.088	-0.0487	Yes
1205	PITG_18054	PITG_18054			26907	-3.165	-0.0481	Yes
1206	PITG_14413	PITG_14413			26910	-3.165	-0.0459	Yes
1207	PITG_02708	PITG_02708			26914	-3.169	-0.0438	Yes

1208	PITG_13636	PITG_13636			26921	-3.184	-0.0418	Yes
1209	PITG_08957	PITG_08957			26986	-3.291	-0.0419	Yes
1210	PITG_13924	PITG_13924			27007	-3.330	-0.0403	Yes
1211	PITG_12300	PITG_12300			27048	-3.394	-0.0394	Yes
1212	PITG_06596	PITG_06596			27055	-3.399	-0.0372	Yes
1213	PITG_02704	PITG_02704			27062	-3.410	-0.0351	Yes
1214	PITG_02392	PITG_02392			27080	-3.440	-0.0333	Yes
1215	PITG_01271	PITG_01271			27088	-3.463	-0.0311	Yes
1216	PITG_00571	PITG_00571			27095	-3.470	-0.0289	Yes
1217	PITG_22529	PITG_22529			27119	-3.526	-0.0272	Yes
1218	PITG_14639	PITG_14639			27127	-3.538	-0.0250	Yes
1219	PITG_21661	PITG_21661			27155	-3.586	-0.0235	Yes
1220	PITG_22020	PITG_22020			27176	-3.631	-0.0217	Yes
1221	PITG_19399	PITG_19399			27227	-3.743	-0.0209	Yes
1222	PITG_21623	PITG_21623			27259	-3.807	-0.0194	Yes
1223	PITG_21127	PITG_21127			27286	-3.872	-0.0177	Yes
1224	PITG_17831	PITG_17831			27306	-3.910	-0.0156	Yes
1225	PITG_18545	PITG_18545			27314	-3.923	-0.0131	Yes
1226	PITG_03493	PITG_03493			27416	-4.186	-0.0140	Yes
1227	PITG_00661	PITG_00661			27446	-4.295	-0.0121	Yes
1228	PITG_14352	PITG_14352			27476	-4.401	-0.0100	Yes
1229	PITG_01062	PITG_01062			27511	-4.554	-0.0081	Yes
1230	PITG_19993	PITG_19993			27522	-4.614	-0.0052	Yes
1231	PITG_02621	PITG_02621			27528	-4.667	-0.0021	Yes
1232	PITG_00691	PITG_00691			27536	-4.719	0.0009	Yes

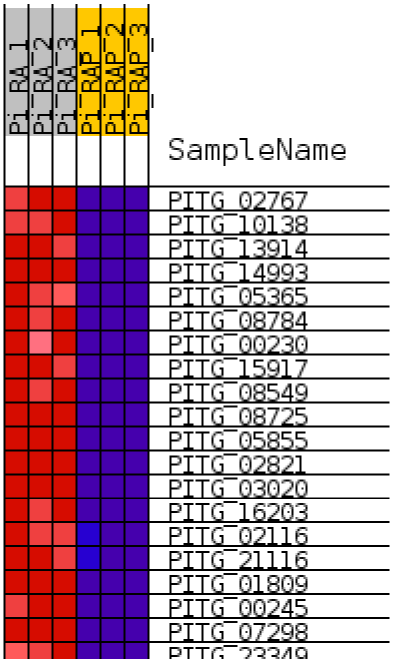

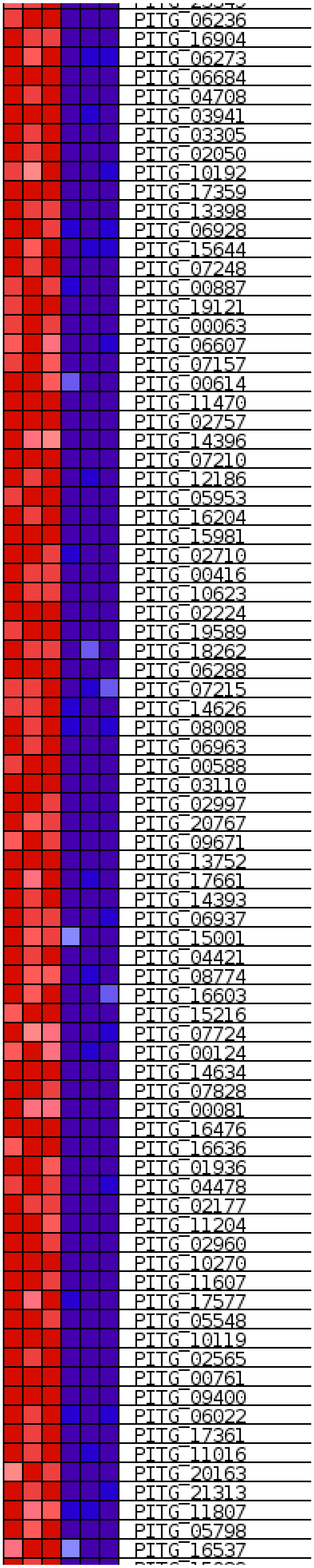
PITG_06236
PITG_16904
PITG_06273
PITG_06684
PITG_04708
PITG_03941
PITG_03305
PITG_02050
PITG_10192
PITG_17359
PITG_13398
PITG_06928
PITG_15644
PITG_07248
PITG_00887
PITG_19121
PITG_00063
PITG_06607
PITG_07157
PITG_00614
PITG_11470
PITG_02757
PITG_14396
PITG_07210
PITG_12186
PITG_05953
PITG_16204
PITG_15981
PITG_02710
PITG_00416
PITG_10623
PITG_02224
PITG_19589
PITG_18262
PITG_06288
PITG_07215
PITG_14626
PITG_08008
PITG_06963
PITG_00588
PITG_03110
PITG_02997
PITG_20767
PITG_09671
PITG_13752
PITG_17661
PITG_14393
PITG_06937
PITG_15001
PITG_04421
PITG_08774
PITG_16603
PITG_15216
PITG_07724
PITG_00124
PITG_14634
PITG_07828
PITG_00081
PITG_16476
PITG_16636
PITG_01936
PITG_04478
PITG_02177
PITG_11204
PITG_02960
PITG_10270
PITG_11607
PITG_17577
PITG_05548
PITG_10119
PITG_02565
PITG_00761
PITG_09400
PITG_06022
PITG_17361
PITG_11016
PITG_20163
PITG_21313
PITG_11807
PITG_05798
PITG_16537

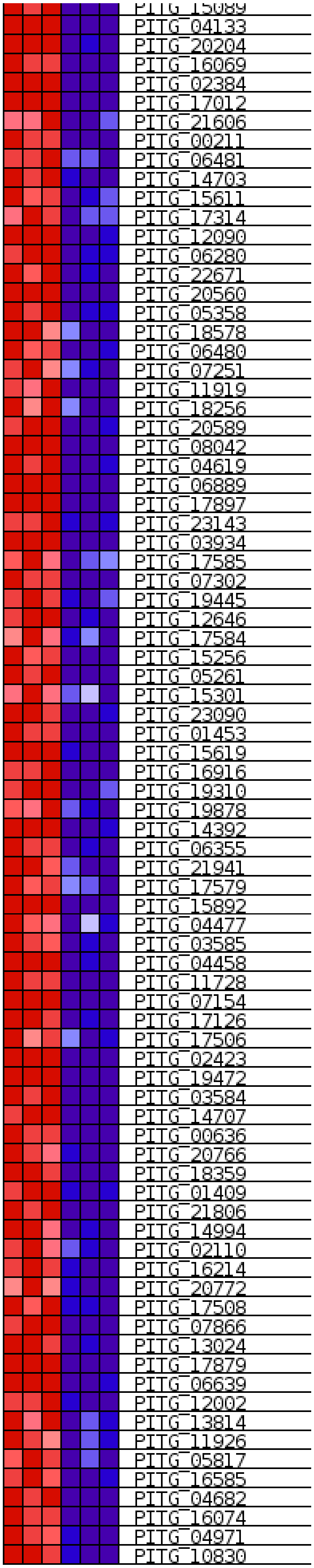
PITG_04133
PITG_20204
PITG_16069
PITG_02384
PITG_17012
PITG_21606
PITG_00211
PITG_06481
PITG_14703
PITG_15611
PITG_17314
PITG_12090
PITG_06280
PITG_22671
PITG_20560
PITG_05358
PITG_18578
PITG_06480
PITG_07251
PITG_11919
PITG_18256
PITG_20589
PITG_08042
PITG_04619
PITG_06889
PITG_17897
PITG_23143
PITG_03934
PITG_17585
PITG_07302
PITG_19445
PITG_12646
PITG_17584
PITG_15256
PITG_05261
PITG_15301
PITG_23090
PITG_01453
PITG_15619
PITG_16916
PITG_19310
PITG_19878
PITG_14392
PITG_06355
PITG_21941
PITG_17579
PITG_15892
PITG_04477
PITG_03585
PITG_04458
PITG_11728
PITG_07154
PITG_17126
PITG_17506
PITG_02423
PITG_19472
PITG_03584
PITG_14707
PITG_00636
PITG_20766
PITG_18359
PITG_01409
PITG_21806
PITG_14994
PITG_02110
PITG_16214
PITG_20772
PITG_17508
PITG_07866
PITG_13024
PITG_17879
PITG_06639
PITG_12002
PITG_13814
PITG_11926
PITG_05817
PITG_16585
PITG_04682
PITG_16074
PITG_04971
PITG_10830

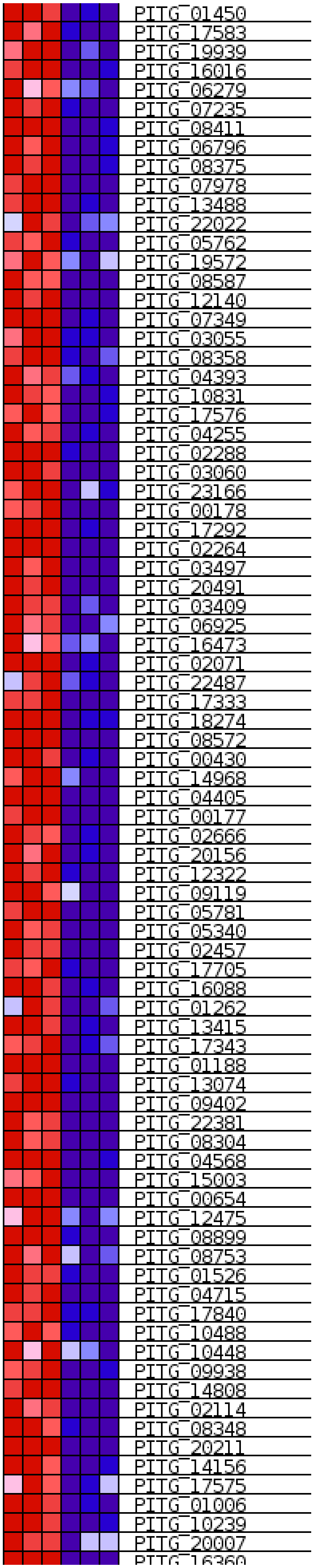
PITG_01450
PITG_17583
PITG_19939
PITG_16016
PITG_06279
PITG_07235
PITG_08411
PITG_06796
PITG_08375
PITG_07978
PITG_13488
PITG_22022
PITG_05762
PITG_19572
PITG_08587
PITG_12140
PITG_07349
PITG_03055
PITG_08358
PITG_04393
PITG_10831
PITG_17576
PITG_04255
PITG_02288
PITG_03060
PITG_23166
PITG_00178
PITG_17292
PITG_02264
PITG_03497
PITG_20491
PITG_03409
PITG_06925
PITG_16473
PITG_02071
PITG_22487
PITG_17333
PITG_18274
PITG_08572
PITG_00430
PITG_14968
PITG_04405
PITG_00177
PITG_02666
PITG_20156
PITG_12322
PITG_09119
PITG_05781
PITG_05340
PITG_02457
PITG_17705
PITG_16088
PITG_01262
PITG_13415
PITG_17343
PITG_01188
PITG_13074
PITG_09402
PITG_22381
PITG_08304
PITG_04568
PITG_15003
PITG_00654
PITG_12475
PITG_08899
PITG_08753
PITG_01526
PITG_04715
PITG_17840
PITG_10488
PITG_10448
PITG_09938
PITG_14808
PITG_02114
PITG_08348
PITG_20211
PITG_14156
PITG_17575
PITG_01006
PITG_10239
PITG_20007
PITG_16360

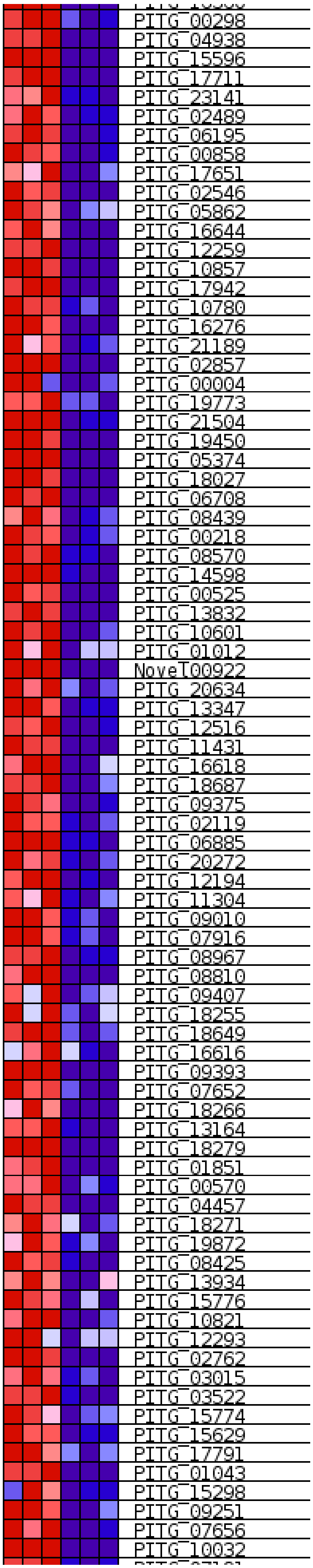
PITG_00298
PITG_04938
PITG_15596
PITG_17711
PITG_23141
PITG_02489
PITG_06195
PITG_00858
PITG_17651
PITG_02546
PITG_05862
PITG_16644
PITG_12259
PITG_10857
PITG_17942
PITG_10780
PITG_16276
PITG_21189
PITG_02857
PITG_00004
PITG_19773
PITG_21504
PITG_19450
PITG_05374
PITG_18027
PITG_06708
PITG_08439
PITG_00218
PITG_08570
PITG_14598
PITG_00525
PITG_13832
PITG_10601
PITG_01012
Novel00922
PITG_20634
PITG_13347
PITG_12516
PITG_11431
PITG_16618
PITG_18687
PITG_09375
PITG_02119
PITG_06885
PITG_20272
PITG_12194
PITG_11304
PITG_09010
PITG_07916
PITG_08967
PITG_08810
PITG_09407
PITG_18255
PITG_18649
PITG_16616
PITG_09393
PITG_07652
PITG_18266
PITG_13164
PITG_18279
PITG_01851
PITG_00570
PITG_04457
PITG_18271
PITG_19872
PITG_08425
PITG_13934
PITG_15776
PITG_10821
PITG_12293
PITG_02762
PITG_03015
PITG_03522
PITG_15774
PITG_15629
PITG_17791
PITG_01043
PITG_15298
PITG_09251
PITG_07656
PITG_10032

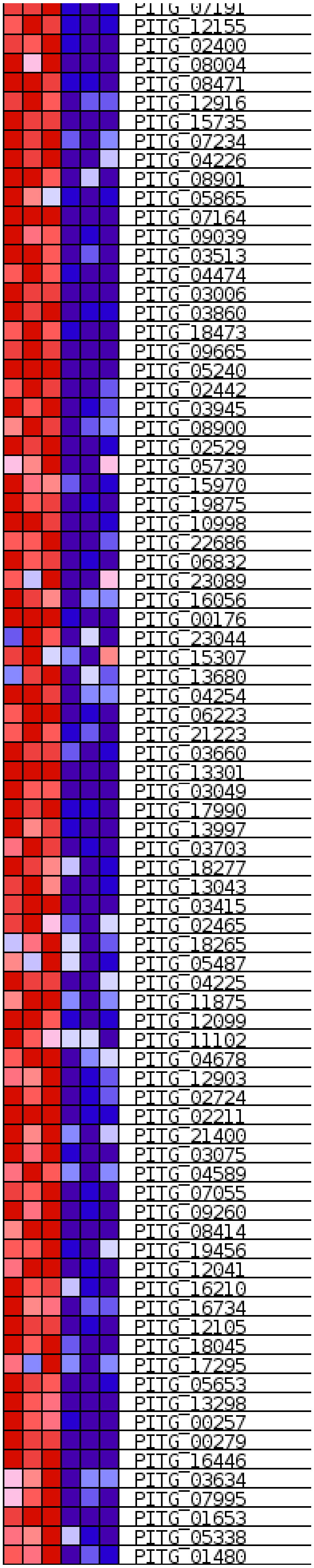

PITG_07191
PITG_12155
PITG_02400
PITG_08004
PITG_08471
PITG_12916
PITG_15735
PITG_07234
PITG_04226
PITG_08901
PITG_05865
PITG_07164
PITG_09039
PITG_03513
PITG_04474
PITG_03006
PITG_03860
PITG_18473
PITG_09665
PITG_05240
PITG_02442
PITG_03945
PITG_08900
PITG_02529
PITG_05730
PITG_15970
PITG_19875
PITG_10998
PITG_22686
PITG_06832
PITG_23089
PITG_16056
PITG_00176
PITG_23044
PITG_15307
PITG_13680
PITG_04254
PITG_06223
PITG_21223
PITG_03660
PITG_13301
PITG_03049
PITG_17990
PITG_13997
PITG_03703
PITG_18277
PITG_13043
PITG_03415
PITG_02465
PITG_18265
PITG_05487
PITG_04225
PITG_11875
PITG_12099
PITG_11102
PITG_04678
PITG_12903
PITG_02724
PITG_02211
PITG_21400
PITG_03075
PITG_04589
PITG_07055
PITG_09260
PITG_08414
PITG_19456
PITG_12041
PITG_16210
PITG_16734
PITG_12105
PITG_18045
PITG_17295
PITG_05653
PITG_13298
PITG_00257
PITG_00279
PITG_16446
PITG_03634
PITG_07995
PITG_01653
PITG_05338
PITG_01480

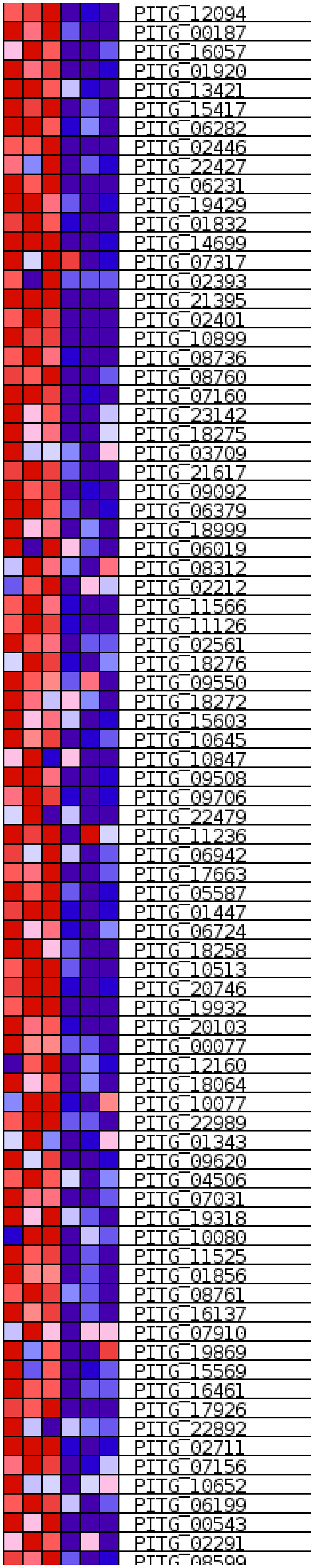
PITG_12094
PITG_00187
PITG_16057
PITG_01920
PITG_13421
PITG_15417
PITG_06282
PITG_02446
PITG_22427
PITG_06231
PITG_19429
PITG_01832
PITG_14699
PITG_07317
PITG_02393
PITG_21395
PITG_02401
PITG_10899
PITG_08736
PITG_08760
PITG_07160
PITG_23142
PITG_18275
PITG_03709
PITG_21617
PITG_09092
PITG_06379
PITG_18999
PITG_06019
PITG_08312
PITG_02212
PITG_11566
PITG_11126
PITG_02561
PITG_18276
PITG_09550
PITG_18272
PITG_15603
PITG_10645
PITG_10847
PITG_09508
PITG_09706
PITG_22479
PITG_11236
PITG_06942
PITG_17663
PITG_05587
PITG_01447
PITG_06724
PITG_18258
PITG_10513
PITG_20746
PITG_19932
PITG_20103
PITG_00077
PITG_12160
PITG_18064
PITG_10077
PITG_22989
PITG_01343
PITG_09620
PITG_04506
PITG_07031
PITG_19318
PITG_10080
PITG_11525
PITG_01856
PITG_08761
PITG_16137
PITG_07910
PITG_19869
PITG_15569
PITG_16461
PITG_17926
PITG_22892
PITG_02711
PITG_07156
PITG_10652
PITG_06199
PITG_00543
PITG_02291
PITG_08599

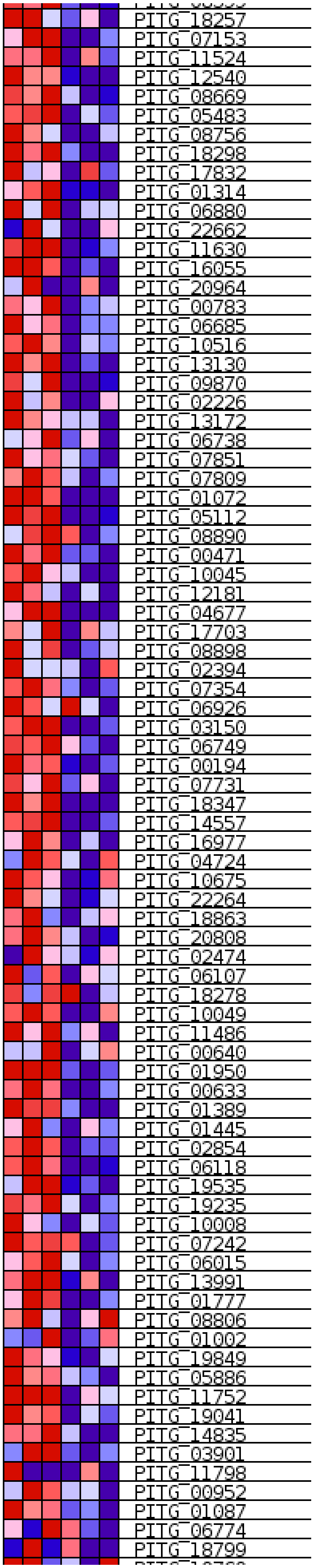
PITG_18257
PITG_07153
PITG_11524
PITG_12540
PITG_08669
PITG_05483
PITG_08756
PITG_18298
PITG_17832
PITG_01314
PITG_06880
PITG_22662
PITG_11630
PITG_16055
PITG_20964
PITG_00783
PITG_06685
PITG_10516
PITG_13130
PITG_09870
PITG_02226
PITG_13172
PITG_06738
PITG_07851
PITG_07809
PITG_01072
PITG_05112
PITG_08890
PITG_00471
PITG_10045
PITG_12181
PITG_04677
PITG_17703
PITG_08898
PITG_02394
PITG_07354
PITG_06926
PITG_03150
PITG_06749
PITG_00194
PITG_07731
PITG_18347
PITG_14557
PITG_16977
PITG_04724
PITG_10675
PITG_22264
PITG_18863
PITG_20808
PITG_02474
PITG_06107
PITG_18278
PITG_10049
PITG_11486
PITG_00640
PITG_01950
PITG_00633
PITG_01389
PITG_01445
PITG_02854
PITG_06118
PITG_19535
PITG_19235
PITG_10008
PITG_07242
PITG_06015
PITG_13991
PITG_01777
PITG_08806
PITG_01002
PITG_19849
PITG_05886
PITG_11752
PITG_19041
PITG_14835
PITG_03901
PITG_11798
PITG_00952
PITG_01087
PITG_06774
PITG_18799

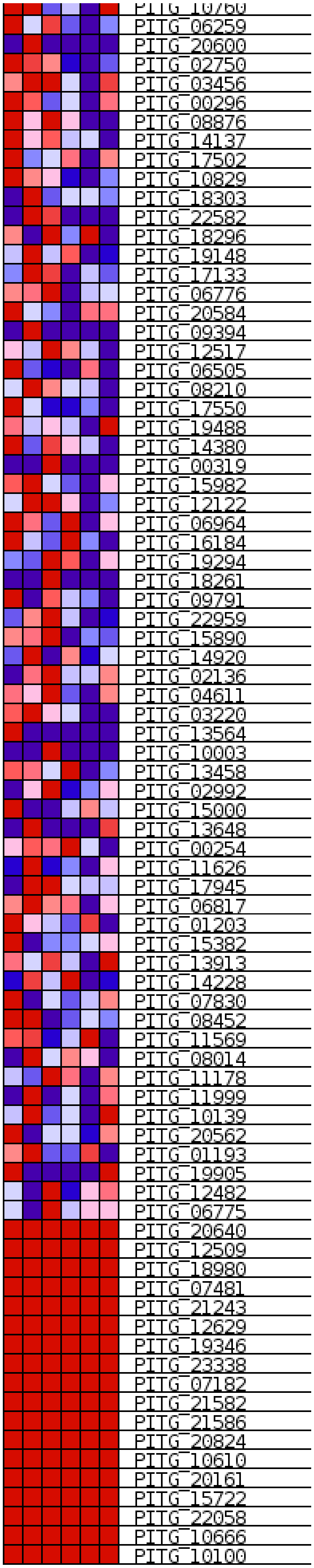

PITG_10760
PITG_06259
PITG_20600
PITG_02750
PITG_03456
PITG_00296
PITG_08876
PITG_14137
PITG_17502
PITG_10829
PITG_18303
PITG_22582
PITG_18296
PITG_19148
PITG_17133
PITG_06776
PITG_20584
PITG_09394
PITG_12517
PITG_06505
PITG_08210
PITG_17550
PITG_19488
PITG_14380
PITG_00319
PITG_15982
PITG_12122
PITG_06964
PITG_16184
PITG_19294
PITG_18261
PITG_09791
PITG_22959
PITG_15890
PITG_14920
PITG_02136
PITG_04611
PITG_03220
PITG_13564
PITG_10003
PITG_13458
PITG_02992
PITG_15000
PITG_13648
PITG_00254
PITG_11626
PITG_17945
PITG_06817
PITG_01203
PITG_15382
PITG_13913
PITG_14228
PITG_07830
PITG_08452
PITG_11569
PITG_08014
PITG_11178
PITG_11999
PITG_10139
PITG_20562
PITG_01193
PITG_19905
PITG_12482
PITG_06775
PITG_20640
PITG_12509
PITG_18980
PITG_07481
PITG_21243
PITG_12629
PITG_19346
PITG_23338
PITG_07182
PITG_21582
PITG_21586
PITG_20824
PITG_10610
PITG_20161
PITG_15722
PITG_22058
PITG_10666
PITG_10100

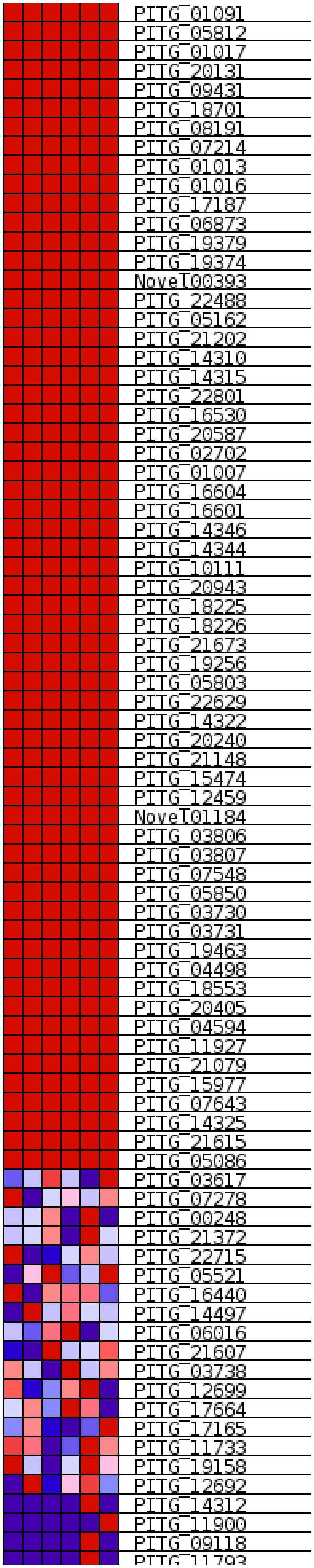
PITG_01091
PITG_05812
PITG_01017
PITG_20131
PITG_09431
PITG_18701
PITG_08191
PITG_07214
PITG_01013
PITG_01016
PITG_17187
PITG_06873
PITG_19379
PITG_19374
Novel00393
PITG_22488
PITG_05162
PITG_21202
PITG_14310
PITG_14315
PITG_22801
PITG_16530
PITG_20587
PITG_02702
PITG_01007
PITG_16604
PITG_16601
PITG_14346
PITG_14344
PITG_10111
PITG_20943
PITG_18225
PITG_18226
PITG_21673
PITG_19256
PITG_05803
PITG_22629
PITG_14322
PITG_20240
PITG_21148
PITG_15474
PITG_12459
Novel01184
PITG_03806
PITG_03807
PITG_07548
PITG_05850
PITG_03730
PITG_03731
PITG_19463
PITG_04498
PITG_18553
PITG_20405
PITG_04594
PITG_11927
PITG_21079
PITG_15977
PITG_07643
PITG_14325
PITG_21615
PITG_05086
PITG_03617
PITG_07278
PITG_00248
PITG_21372
PITG_22715
PITG_05521
PITG_16440
PITG_14497
PITG_06016
PITG_21607
PITG_03738
PITG_12699
PITG_17664
PITG_17165
PITG_11733
PITG_19158
PITG_12692
PITG_14312
PITG_11900
PITG_09118
PITG_11793

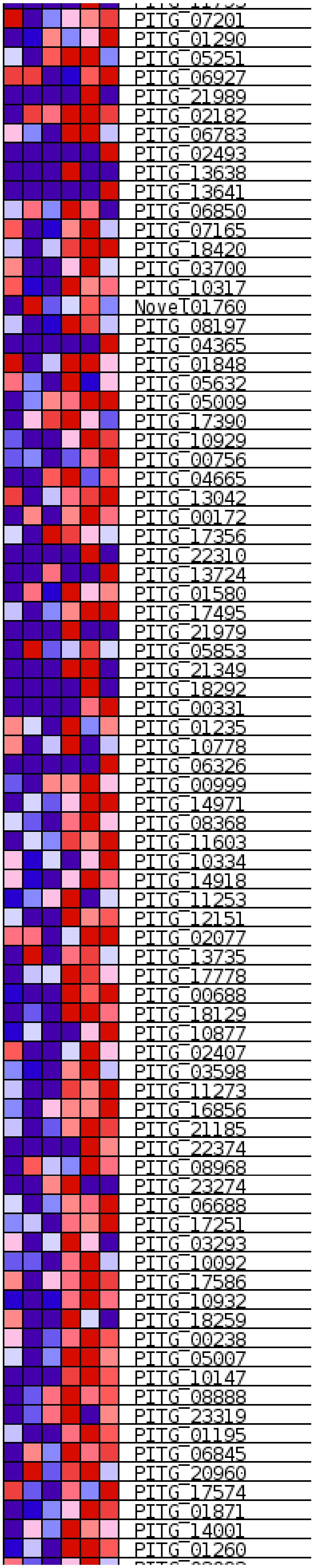
PITG_07201
PITG_01290
PITG_05251
PITG_06927
PITG_21989
PITG_02182
PITG_06783
PITG_02493
PITG_13638
PITG_13641
PITG_06850
PITG_07165
PITG_18420
PITG_03700
PITG_10317
Novel01760
PITG_08197
PITG_04365
PITG_01848
PITG_05632
PITG_05009
PITG_17390
PITG_10929
PITG_00756
PITG_04665
PITG_13042
PITG_00172
PITG_17356
PITG_22310
PITG_13724
PITG_01580
PITG_17495
PITG_21979
PITG_05853
PITG_21349
PITG_18292
PITG_00331
PITG_01235
PITG_10778
PITG_06326
PITG_00999
PITG_14971
PITG_08368
PITG_11603
PITG_10334
PITG_14918
PITG_11253
PITG_12151
PITG_02077
PITG_13735
PITG_17778
PITG_00688
PITG_18129
PITG_10877
PITG_02407
PITG_03598
PITG_11273
PITG_16856
PITG_21185
PITG_22374
PITG_08968
PITG_23274
PITG_06688
PITG_17251
PITG_03293
PITG_10092
PITG_17586
PITG_10932
PITG_18259
PITG_00238
PITG_05007
PITG_10147
PITG_08888
PITG_23319
PITG_01195
PITG_06845
PITG_20960
PITG_17574
PITG_01871
PITG_14001
PITG_01260

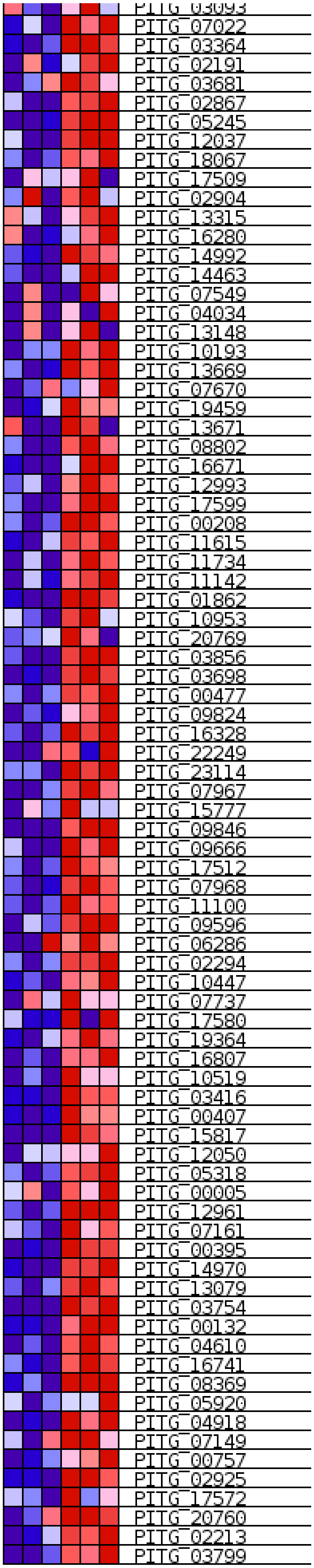
PITG_03093
PITG_07022
PITG_03364
PITG_02191
PITG_03681
PITG_02867
PITG_05245
PITG_12037
PITG_18067
PITG_17509
PITG_02904
PITG_13315
PITG_16280
PITG_14992
PITG_14463
PITG_07549
PITG_04034
PITG_13148
PITG_10193
PITG_13669
PITG_07670
PITG_19459
PITG_13671
PITG_08802
PITG_16671
PITG_12993
PITG_17599
PITG_00208
PITG_11615
PITG_11734
PITG_11142
PITG_01862
PITG_10953
PITG_20769
PITG_03856
PITG_03698
PITG_00477
PITG_09824
PITG_16328
PITG_22249
PITG_23114
PITG_07967
PITG_15777
PITG_09846
PITG_09666
PITG_17512
PITG_07968
PITG_11100
PITG_09596
PITG_06286
PITG_02294
PITG_10447
PITG_07737
PITG_17580
PITG_19364
PITG_16807
PITG_10519
PITG_03416
PITG_00407
PITG_15817
PITG_12050
PITG_05318
PITG_00005
PITG_12961
PITG_07161
PITG_00395
PITG_14970
PITG_13079
PITG_03754
PITG_00132
PITG_04610
PITG_16741
PITG_08369
PITG_05920
PITG_04918
PITG_07149
PITG_00757
PITG_02925
PITG_17572
PITG_20760
PITG_02213
PITG_03799

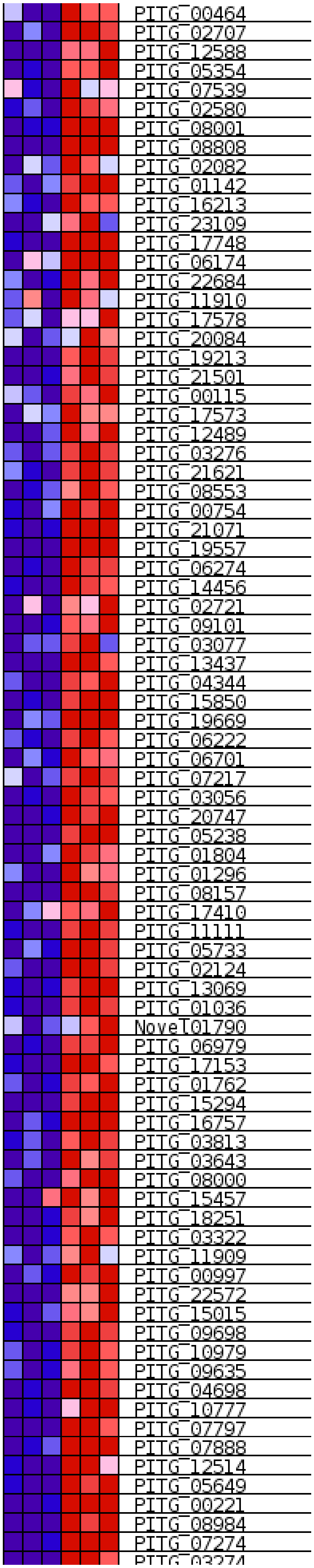
PITG_00464
PITG_02707
PITG_12588
PITG_05354
PITG_07539
PITG_02580
PITG_08001
PITG_08808
PITG_02082
PITG_01142
PITG_16213
PITG_23109
PITG_17748
PITG_06174
PITG_22684
PITG_11910
PITG_17578
PITG_20084
PITG_19213
PITG_21501
PITG_00115
PITG_17573
PITG_12489
PITG_03276
PITG_21621
PITG_08553
PITG_00754
PITG_21071
PITG_19557
PITG_06274
PITG_14456
PITG_02721
PITG_09101
PITG_03077
PITG_13437
PITG_04344
PITG_15850
PITG_19669
PITG_06222
PITG_06701
PITG_07217
PITG_03056
PITG_20747
PITG_05238
PITG_01804
PITG_01296
PITG_08157
PITG_17410
PITG_11111
PITG_05733
PITG_02124
PITG_13069
PITG_01036
Novel01790
PITG_06979
PITG_17153
PITG_01762
PITG_15294
PITG_16757
PITG_03813
PITG_03643
PITG_08000
PITG_15457
PITG_18251
PITG_03322
PITG_11909
PITG_00997
PITG_22572
PITG_15015
PITG_09698
PITG_10979
PITG_09635
PITG_04698
PITG_10777
PITG_07797
PITG_07888
PITG_12514
PITG_05649
PITG_00221
PITG_08984
PITG_07274
PITG_03274

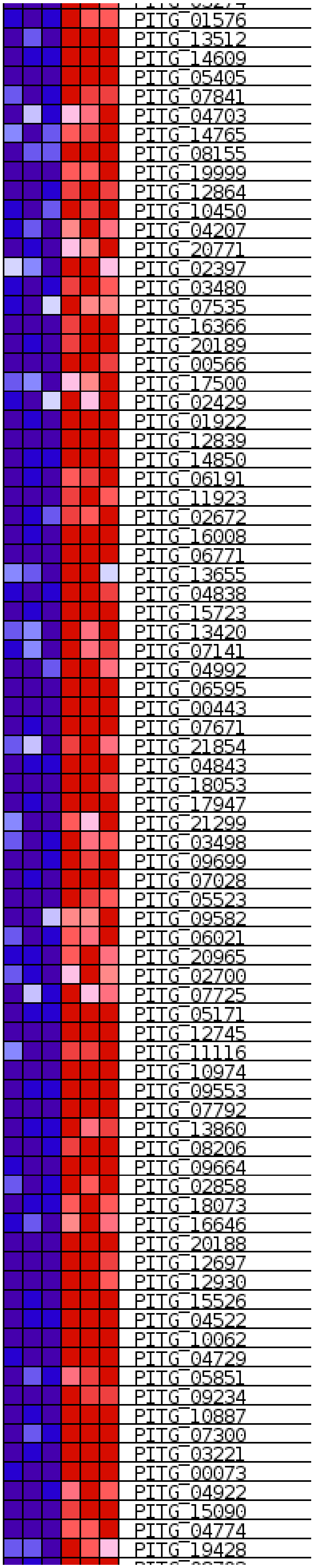
PITG_01576
PITG_13512
PITG_14609
PITG_05405
PITG_07841
PITG_04703
PITG_14765
PITG_08155
PITG_19999
PITG_12864
PITG_10450
PITG_04207
PITG_20771
PITG_02397
PITG_03480
PITG_07535
PITG_16366
PITG_20189
PITG_00566
PITG_17500
PITG_02429
PITG_01922
PITG_12839
PITG_14850
PITG_06191
PITG_11923
PITG_02672
PITG_16008
PITG_06771
PITG_13655
PITG_04838
PITG_15723
PITG_13420
PITG_07141
PITG_04992
PITG_06595
PITG_00443
PITG_07671
PITG_21854
PITG_04843
PITG_18053
PITG_17947
PITG_21299
PITG_03498
PITG_09699
PITG_07028
PITG_05523
PITG_09582
PITG_06021
PITG_20965
PITG_02700
PITG_07725
PITG_05171
PITG_12745
PITG_11116
PITG_10974
PITG_09553
PITG_07792
PITG_13860
PITG_08206
PITG_09664
PITG_02858
PITG_18073
PITG_16646
PITG_20188
PITG_12697
PITG_12930
PITG_15526
PITG_04522
PITG_10062
PITG_04729
PITG_05851
PITG_09234
PITG_10887
PITG_07300
PITG_03221
PITG_00073
PITG_04922
PITG_15090
PITG_04774
PITG_19428

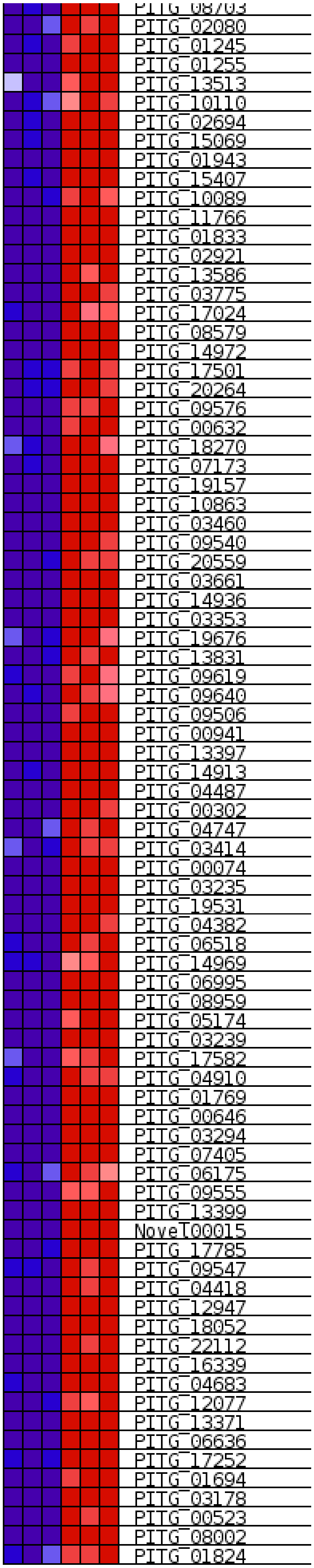
PITG_08703
PITG_02080
PITG_01245
PITG_01255
PITG_13513
PITG_10110
PITG_02694
PITG_15069
PITG_01943
PITG_15407
PITG_10089
PITG_11766
PITG_01833
PITG_02921
PITG_13586
PITG_03775
PITG_17024
PITG_08579
PITG_14972
PITG_17501
PITG_20264
PITG_09576
PITG_00632
PITG_18270
PITG_07173
PITG_19157
PITG_10863
PITG_03460
PITG_09540
PITG_20559
PITG_03661
PITG_14936
PITG_03353
PITG_19676
PITG_13831
PITG_09619
PITG_09640
PITG_09506
PITG_00941
PITG_13397
PITG_14913
PITG_04487
PITG_00302
PITG_04747
PITG_03414
PITG_00074
PITG_03235
PITG_19531
PITG_04382
PITG_06518
PITG_14969
PITG_06995
PITG_08959
PITG_05174
PITG_03239
PITG_17582
PITG_04910
PITG_01769
PITG_00646
PITG_03294
PITG_07405
PITG_06175
PITG_09555
PITG_13399
Novel00015
PITG_17785
PITG_09547
PITG_04418
PITG_12947
PITG_18052
PITG_22112
PITG_16339
PITG_04683
PITG_12077
PITG_13371
PITG_06636
PITG_17252
PITG_01694
PITG_03178
PITG_00523
PITG_08002
PITG_01824

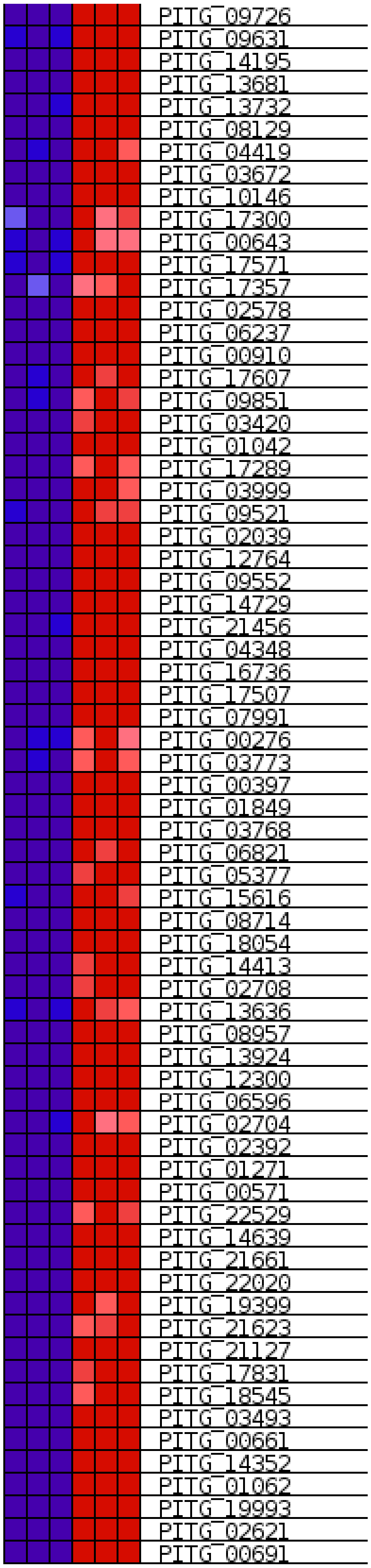

Fig 2: ORGANONITROGEN_COMPOUND_METABOLIC_PROCESS(GO:1901564)
Blue-Pink O' Gram in the Space of the Analyzed GeneSet

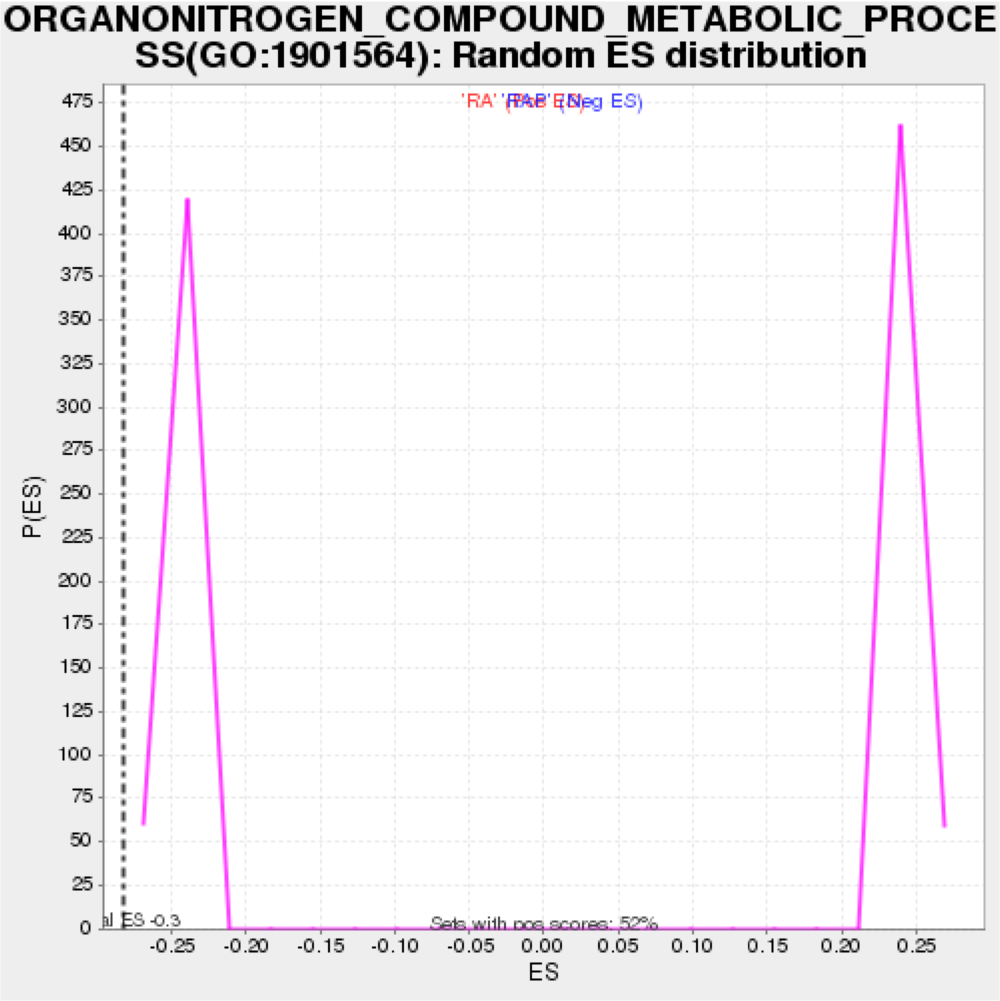

Fig 3: ORGANONITROGEN_COMPOUND_METABOLIC_PROCESS(GO:1901564): Random ES distribution
Gene set null distribution of ES for
ORGANONITROGEN_COMPOUND_METABOLIC_PROCESS(GO:1901564)

4. peptide biosynthetic process

Table: GSEA Results Summary

Dataset	fpkm.sample
Phenotype	sample.cls
Upregulated in class	RAP
GeneSet	PEPTIDE_BIOSYNTHETIC_PROCESS(GO:0043043)
Enrichment Score (ES)	-0.80185455
Normalized Enrichment Score (NES)	-1.0859567
Nominal p-value	0.0
FDR q-value	0.077120826
FWER p-Value	0.06

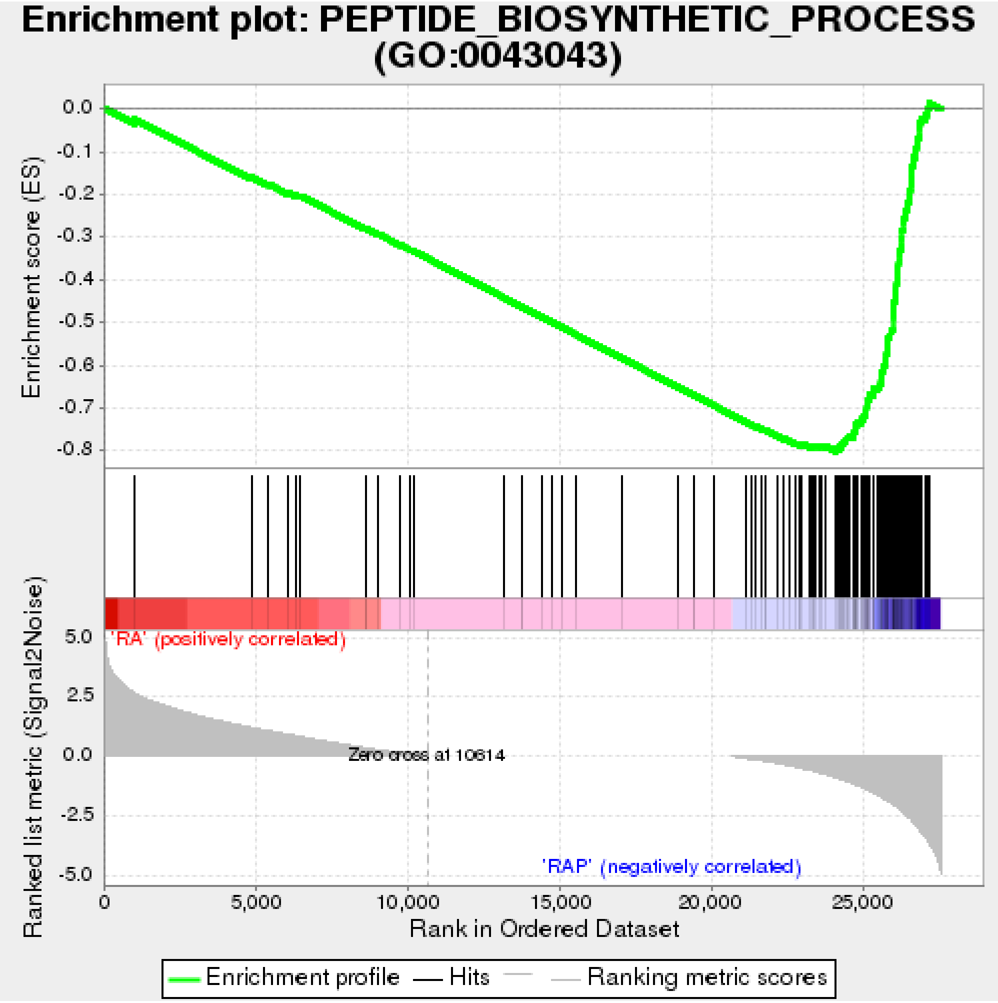

Fig 1: Enrichment plot: PEPTIDE_BIOSYNTHETIC_PROCESS(GO:0043043)
Profile of the Running ES Score & Positions of GeneSet Members on the Rank Ordered List

Table: GSEA details [plain text format]

	PROBE	DESCRIPTION	GENE	GENE_TITLE	RANK IN	RANK	RUNNING	CORE

		(from dataset)	SYMBOL		GENE LIST	METRIC SCORE	ES	ENRICHMENT
1	PITG_19121	PITG_19121			962	2.666	-0.0239	No
2	PITG_17651	PITG_17651			4826	1.224	-0.1597	No
3	Novel00922	Novel00922			5355	1.090	-0.1744	No
4	PITG_07234	PITG_07234			6061	0.926	-0.1963	No
5	PITG_05730	PITG_05730			6273	0.878	-0.2003	No
6	PITG_03660	PITG_03660			6456	0.832	-0.2034	No
7	PITG_11630	PITG_11630			8572	0.362	-0.2791	No
8	PITG_10516	PITG_10516			8635	0.351	-0.2799	No
9	PITG_14557	PITG_14557			8993	0.287	-0.2917	No
10	PITG_18303	PITG_18303			9750	0.151	-0.3187	No
11	PITG_09791	PITG_09791			10045	0.103	-0.3290	No
12	PITG_02992	PITG_02992			10209	0.072	-0.3346	No
13	PITG_20824	PITG_20824			13172	0.000	-0.4427	No
14	PITG_15722	PITG_15722			13740	0.000	-0.4634	No
15	PITG_22058	PITG_22058			13753	0.000	-0.4638	No
16	PITG_01091	PITG_01091			14376	0.000	-0.4865	No
17	PITG_05812	PITG_05812			14754	0.000	-0.5003	No
18	PITG_09431	PITG_09431			15054	0.000	-0.5112	No
19	PITG_06873	PITG_06873			15537	0.000	-0.5288	No
20	PITG_16530	PITG_16530			17026	0.000	-0.5831	No
21	PITG_20240	PITG_20240			18905	0.000	-0.6516	No
22	PITG_03806	PITG_03806			19383	0.000	-0.6690	No
23	PITG_03807	PITG_03807			19384	0.000	-0.6690	No
24	PITG_04594	PITG_04594			20065	0.000	-0.6938	No
25	PITG_02493	PITG_02493			21136	-0.100	-0.7325	No
26	PITG_05009	PITG_05009			21324	-0.134	-0.7387	No
27	PITG_22310	PITG_22310			21487	-0.165	-0.7439	No
28	PITG_21349	PITG_21349			21623	-0.186	-0.7481	No
29	PITG_12151	PITG_12151			21769	-0.212	-0.7525	No
30	PITG_13735	PITG_13735			21784	-0.214	-0.7521	No
31	PITG_05007	PITG_05007			22208	-0.303	-0.7663	No
32	PITG_03093	PITG_03093			22409	-0.359	-0.7721	No
33	PITG_10193	PITG_10193			22606	-0.404	-0.7775	No
34	PITG_11734	PITG_11734			22780	-0.455	-0.7819	No
35	PITG_16328	PITG_16328			22919	-0.491	-0.7849	No
36	PITG_22249	PITG_22249			22933	-0.496	-0.7833	No
37	PITG_09846	PITG_09846			22986	-0.514	-0.7830	No
38	PITG_12961	PITG_12961			23230	-0.590	-0.7894	No

39	PITG_16741	PITG_16741			23328	-0.615	-0.7904	No
40	PITG_08369	PITG_08369			23360	-0.629	-0.7889	No
41	PITG_04918	PITG_04918			23385	-0.637	-0.7871	No
42	PITG_03799	PITG_03799			23448	-0.655	-0.7866	No
43	PITG_05354	PITG_05354			23584	-0.710	-0.7885	No
44	PITG_02580	PITG_02580			23651	-0.734	-0.7879	No
45	PITG_17748	PITG_17748			23795	-0.780	-0.7898	No
46	PITG_21071	PITG_21071			24126	-0.913	-0.7980	Yes
47	PITG_14456	PITG_14456			24139	-0.918	-0.7946	Yes
48	PITG_19669	PITG_19669			24217	-0.958	-0.7934	Yes
49	PITG_06222	PITG_06222			24228	-0.961	-0.7897	Yes
50	PITG_11111	PITG_11111			24301	-0.988	-0.7882	Yes
51	PITG_05733	PITG_05733			24305	-0.989	-0.7842	Yes
52	PITG_17153	PITG_17153			24348	-1.006	-0.7815	Yes
53	PITG_01762	PITG_01762			24386	-1.026	-0.7785	Yes
54	PITG_16757	PITG_16757			24413	-1.036	-0.7751	Yes
55	PITG_18251	PITG_18251			24457	-1.057	-0.7723	Yes
56	PITG_03322	PITG_03322			24462	-1.057	-0.7680	Yes
57	PITG_10979	PITG_10979			24567	-1.100	-0.7672	Yes
58	PITG_07797	PITG_07797			24662	-1.147	-0.7658	Yes
59	PITG_07888	PITG_07888			24666	-1.148	-0.7611	Yes
60	PITG_03274	PITG_03274			24715	-1.175	-0.7579	Yes
61	PITG_14609	PITG_14609			24732	-1.182	-0.7535	Yes
62	PITG_05405	PITG_05405			24742	-1.189	-0.7489	Yes
63	PITG_07841	PITG_07841			24743	-1.190	-0.7439	Yes
64	PITG_04703	PITG_04703			24756	-1.196	-0.7393	Yes
65	PITG_19999	PITG_19999			24792	-1.214	-0.7355	Yes
66	PITG_12864	PITG_12864			24801	-1.219	-0.7306	Yes
67	PITG_03480	PITG_03480			24930	-1.293	-0.7299	Yes
68	PITG_20189	PITG_20189			24966	-1.317	-0.7256	Yes
69	PITG_01922	PITG_01922			25002	-1.336	-0.7213	Yes
70	PITG_12839	PITG_12839			25031	-1.352	-0.7167	Yes
71	PITG_14850	PITG_14850			25066	-1.380	-0.7121	Yes
72	PITG_11923	PITG_11923			25096	-1.398	-0.7073	Yes
73	PITG_16008	PITG_16008			25114	-1.410	-0.7020	Yes
74	PITG_06771	PITG_06771			25118	-1.413	-0.6962	Yes
75	PITG_15723	PITG_15723			25159	-1.438	-0.6916	Yes
76	PITG_07141	PITG_07141			25165	-1.441	-0.6858	Yes
77	PITG_04992	PITG_04992			25172	-1.445	-0.6799	Yes

78	PITG_00443	PITG_00443			25193	-1.456	-0.6745	Yes
79	PITG_04843	PITG_04843			25217	-1.471	-0.6692	Yes
80	PITG_05171	PITG_05171			25340	-1.548	-0.6672	Yes
81	PITG_12745	PITG_12745			25347	-1.553	-0.6609	Yes
82	PITG_10974	PITG_10974			25362	-1.559	-0.6548	Yes
83	PITG_20188	PITG_20188			25502	-1.654	-0.6530	Yes
84	PITG_12697	PITG_12697			25529	-1.673	-0.6469	Yes
85	PITG_04729	PITG_04729			25577	-1.699	-0.6415	Yes
86	PITG_09234	PITG_09234			25595	-1.710	-0.6349	Yes
87	PITG_10887	PITG_10887			25597	-1.713	-0.6278	Yes
88	PITG_07300	PITG_07300			25611	-1.727	-0.6210	Yes
89	PITG_03221	PITG_03221			25613	-1.729	-0.6138	Yes
90	PITG_15090	PITG_15090			25650	-1.757	-0.6077	Yes
91	PITG_04774	PITG_04774			25653	-1.761	-0.6004	Yes
92	PITG_08703	PITG_08703			25716	-1.807	-0.5951	Yes
93	PITG_01255	PITG_01255			25748	-1.835	-0.5885	Yes
94	PITG_10110	PITG_10110			25769	-1.847	-0.5815	Yes
95	PITG_02694	PITG_02694			25772	-1.849	-0.5738	Yes
96	PITG_15069	PITG_15069			25785	-1.859	-0.5665	Yes
97	PITG_01943	PITG_01943			25791	-1.864	-0.5588	Yes
98	PITG_15407	PITG_15407			25795	-1.868	-0.5511	Yes
99	PITG_11766	PITG_11766			25814	-1.881	-0.5439	Yes
100	PITG_01833	PITG_01833			25831	-1.897	-0.5365	Yes
101	PITG_02921	PITG_02921			25864	-1.929	-0.5296	Yes
102	PITG_08579	PITG_08579			25907	-1.965	-0.5228	Yes
103	PITG_20264	PITG_20264			25950	-1.992	-0.5160	Yes
104	PITG_07173	PITG_07173			25980	-2.022	-0.5086	Yes
105	PITG_19157	PITG_19157			25981	-2.022	-0.5001	Yes
106	PITG_10863	PITG_10863			25982	-2.022	-0.4916	Yes
107	PITG_03460	PITG_03460			25987	-2.027	-0.4833	Yes
108	PITG_09540	PITG_09540			25997	-2.035	-0.4751	Yes
109	PITG_03661	PITG_03661			26015	-2.048	-0.4671	Yes
110	PITG_03353	PITG_03353			26026	-2.059	-0.4588	Yes
111	PITG_13831	PITG_13831			26029	-2.061	-0.4502	Yes
112	PITG_09506	PITG_09506			26047	-2.074	-0.4422	Yes
113	PITG_00941	PITG_00941			26052	-2.078	-0.4336	Yes
114	PITG_14913	PITG_14913			26073	-2.104	-0.4255	Yes
115	PITG_04487	PITG_04487			26098	-2.129	-0.4174	Yes
116	PITG_00302	PITG_00302			26103	-2.131	-0.4086	Yes

117	PITG_04747	PITG_04747			26111	-2.138	-0.3999	Yes
118	PITG_03235	PITG_03235			26130	-2.165	-0.3915	Yes
119	PITG_19531	PITG_19531			26134	-2.169	-0.3825	Yes
120	PITG_04382	PITG_04382			26147	-2.181	-0.3738	Yes
121	PITG_06995	PITG_06995			26169	-2.197	-0.3653	Yes
122	PITG_08959	PITG_08959			26174	-2.203	-0.3562	Yes
123	PITG_05174	PITG_05174			26178	-2.205	-0.3471	Yes
124	PITG_03239	PITG_03239			26180	-2.206	-0.3379	Yes
125	PITG_03294	PITG_03294			26205	-2.241	-0.3294	Yes
126	PITG_09555	PITG_09555			26249	-2.286	-0.3213	Yes
127	Novel00015	Novel00015			26276	-2.309	-0.3126	Yes
128	PITG_17785	PITG_17785			26278	-2.310	-0.3029	Yes
129	PITG_12947	PITG_12947			26294	-2.326	-0.2937	Yes
130	PITG_18052	PITG_18052			26295	-2.327	-0.2840	Yes
131	PITG_12077	PITG_12077			26335	-2.375	-0.2754	Yes
132	PITG_13371	PITG_13371			26336	-2.376	-0.2655	Yes
133	PITG_06636	PITG_06636			26348	-2.389	-0.2558	Yes
134	PITG_03178	PITG_03178			26392	-2.437	-0.2472	Yes
135	PITG_00523	PITG_00523			26405	-2.460	-0.2373	Yes
136	PITG_09631	PITG_09631			26454	-2.515	-0.2285	Yes
137	PITG_13681	PITG_13681			26460	-2.519	-0.2181	Yes
138	PITG_10146	PITG_10146			26517	-2.576	-0.2094	Yes
139	PITG_02578	PITG_02578			26546	-2.608	-0.1994	Yes
140	PITG_06237	PITG_06237			26557	-2.626	-0.1888	Yes
141	PITG_00910	PITG_00910			26570	-2.639	-0.1781	Yes
142	PITG_17607	PITG_17607			26591	-2.668	-0.1677	Yes
143	PITG_03420	PITG_03420			26604	-2.687	-0.1569	Yes
144	PITG_01042	PITG_01042			26613	-2.704	-0.1458	Yes
145	PITG_03999	PITG_03999			26623	-2.720	-0.1347	Yes
146	PITG_09521	PITG_09521			26652	-2.752	-0.1242	Yes
147	PITG_02039	PITG_02039			26671	-2.781	-0.1132	Yes
148	PITG_09552	PITG_09552			26705	-2.829	-0.1025	Yes
149	PITG_14729	PITG_14729			26712	-2.839	-0.0908	Yes
150	PITG_07991	PITG_07991			26768	-2.940	-0.0805	Yes
151	PITG_00397	PITG_00397			26815	-3.015	-0.0695	Yes
152	PITG_03768	PITG_03768			26845	-3.062	-0.0577	Yes
153	PITG_06821	PITG_06821			26849	-3.067	-0.0450	Yes
154	PITG_08714	PITG_08714			26862	-3.088	-0.0325	Yes
155	PITG_18054	PITG_18054			26907	-3.165	-0.0208	Yes

156	PITG_06596	PITG_06596			27055	-3.399	-0.0119	Yes
157	PITG_21661	PITG_21661			27155	-3.586	-0.0005	Yes
158	PITG_22020	PITG_22020			27176	-3.631	0.0140	Yes

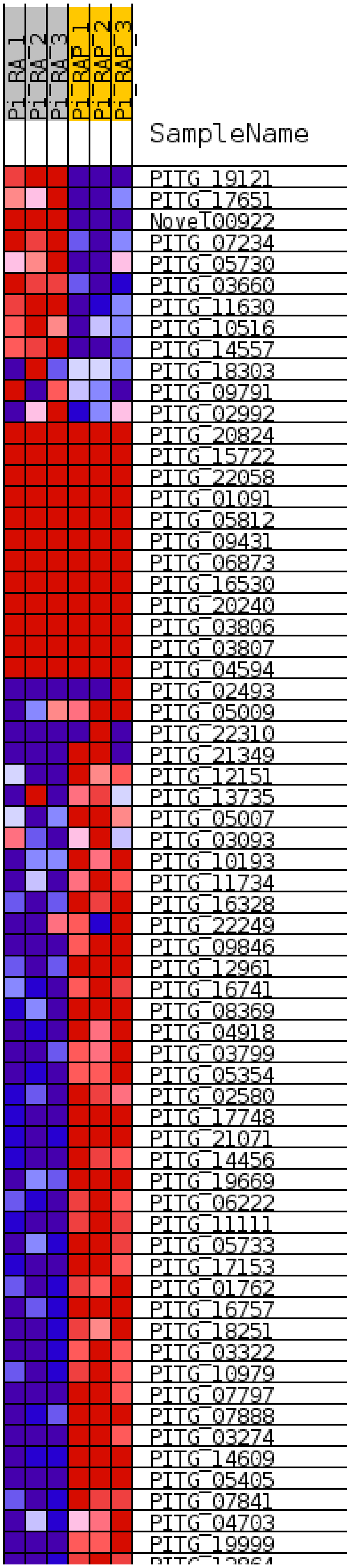

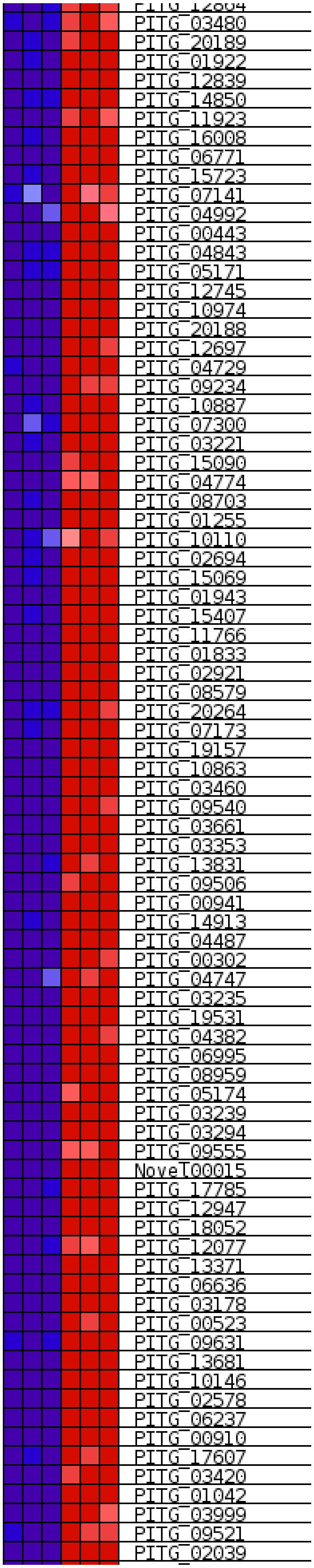
PITG_03480
PITG_20189
PITG_01922
PITG_12839
PITG_14850
PITG_11923
PITG_16008
PITG_06771
PITG_15723
PITG_07141
PITG_04992
PITG_00443
PITG_04843
PITG_05171
PITG_12745
PITG_10974
PITG_20188
PITG_12697
PITG_04729
PITG_09234
PITG_10887
PITG_07300
PITG_03221
PITG_15090
PITG_04774
PITG_08703
PITG_01255
PITG_10110
PITG_02694
PITG_15069
PITG_01943
PITG_15407
PITG_11766
PITG_01833
PITG_02921
PITG_08579
PITG_20264
PITG_07173
PITG_19157
PITG_10863
PITG_03460
PITG_09540
PITG_03661
PITG_03353
PITG_13831
PITG_09506
PITG_00941
PITG_14913
PITG_04487
PITG_00302
PITG_04747
PITG_03235
PITG_19531
PITG_04382
PITG_06995
PITG_08959
PITG_05174
PITG_03239
PITG_03294
PITG_09555
Novel00015
PITG_17785
PITG_12947
PITG_18052
PITG_12077
PITG_13371
PITG_06636
PITG_03178
PITG_00523
PITG_09631
PITG_13681
PITG_10146
PITG_02578
PITG_06237
PITG_00910
PITG_17607
PITG_03420
PITG_01042
PITG_03999
PITG_09521
PITG_02039

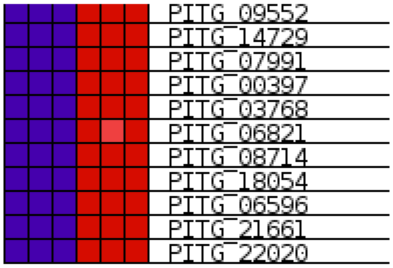

Fig 2: PEPTIDE_BIOSYNTHETIC_PROCESS(GO:0043043)
Blue-Pink O' Gram in the Space of the Analyzed GeneSet

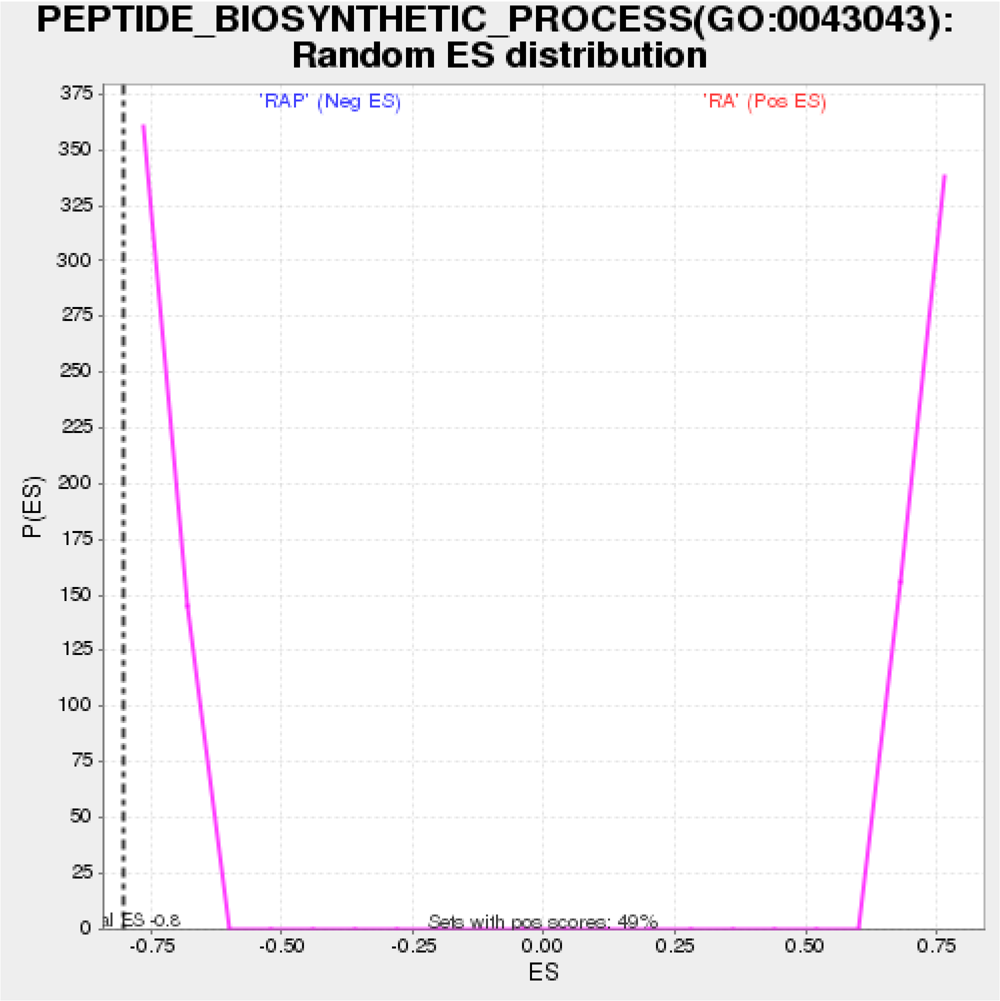

Fig 3: PEPTIDE_BIOSYNTHETIC_PROCESS(GO:0043043): Random ES distribution
Gene set null distribution of ES for PEPTIDE_BIOSYNTHETIC_PROCESS(GO:0043043)

5. peptide metabolic process

Table: GSEA Results Summary

Dataset	fpkm.sample
Phenotype	sample.cls
Upregulated in class	RAP
GeneSet	PEPTIDE_METABOLIC_PROCESS(GO:0006518)
Enrichment Score (ES)	-0.7988726
Normalized Enrichment Score (NES)	-1.0822283
Nominal p-value	0.0
FDR q-value	0.07712086
FWER p-Value	0.06

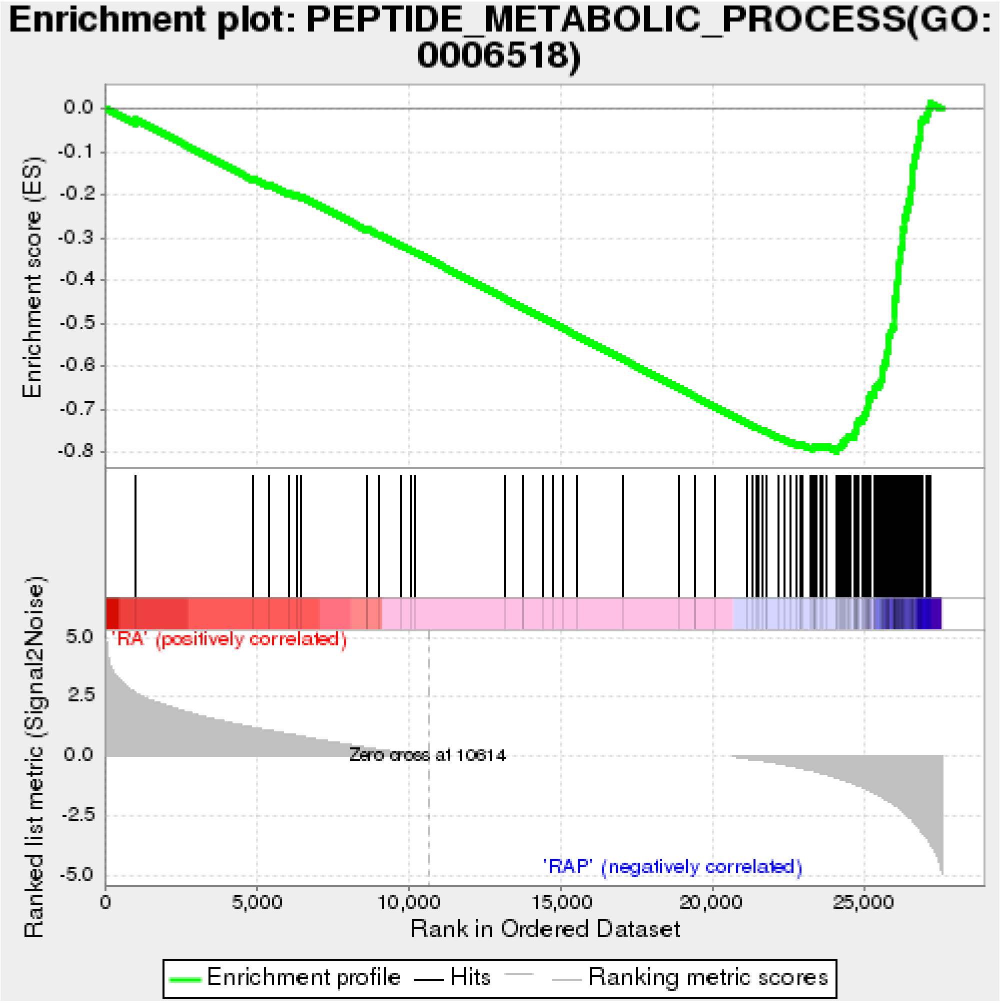

Fig 1: Enrichment plot: PEPTIDE_METABOLIC_PROCESS(GO:0006518)
Profile of the Running ES Score & Positions of GeneSet Members on the Rank Ordered List

Table: GSEA details [plain text format]

	PROBE	DESCRIPTION	GENE	GENE_TITLE	RANK IN	RANK	RUNNING	CORE

		(from dataset)	SYMBOL		GENE LIST	METRIC SCORE	ES	ENRICHMENT
1	PITG_19121	PITG_19121			962	2.666	-0.0241	No
2	PITG_17651	PITG_17651			4826	1.224	-0.1600	No
3	Novel00922	Novel00922			5355	1.090	-0.1748	No
4	PITG_07234	PITG_07234			6061	0.926	-0.1967	No
5	PITG_05730	PITG_05730			6273	0.878	-0.2007	No
6	PITG_03660	PITG_03660			6456	0.832	-0.2039	No
7	PITG_11630	PITG_11630			8572	0.362	-0.2796	No
8	PITG_10516	PITG_10516			8635	0.351	-0.2804	No
9	PITG_14557	PITG_14557			8993	0.287	-0.2923	No
10	PITG_18303	PITG_18303			9750	0.151	-0.3192	No
11	PITG_09791	PITG_09791			10045	0.103	-0.3295	No
12	PITG_02992	PITG_02992			10209	0.072	-0.3352	No
13	PITG_20824	PITG_20824			13172	0.000	-0.4433	No
14	PITG_15722	PITG_15722			13740	0.000	-0.4640	No
15	PITG_22058	PITG_22058			13753	0.000	-0.4644	No
16	PITG_01091	PITG_01091			14376	0.000	-0.4871	No
17	PITG_05812	PITG_05812			14754	0.000	-0.5009	No
18	PITG_09431	PITG_09431			15054	0.000	-0.5118	No
19	PITG_06873	PITG_06873			15537	0.000	-0.5294	No
20	PITG_16530	PITG_16530			17026	0.000	-0.5837	No
21	PITG_20240	PITG_20240			18905	0.000	-0.6522	No
22	PITG_03806	PITG_03806			19383	0.000	-0.6696	No
23	PITG_03807	PITG_03807			19384	0.000	-0.6696	No
24	PITG_04594	PITG_04594			20065	0.000	-0.6944	No
25	PITG_02493	PITG_02493			21136	-0.100	-0.7331	No
26	PITG_05009	PITG_05009			21324	-0.134	-0.7394	No
27	PITG_22310	PITG_22310			21487	-0.165	-0.7446	No
28	PITG_01580	PITG_01580			21509	-0.170	-0.7446	No
29	PITG_21349	PITG_21349			21623	-0.186	-0.7480	No
30	PITG_12151	PITG_12151			21769	-0.212	-0.7524	No
31	PITG_13735	PITG_13735			21784	-0.214	-0.7520	No
32	PITG_05007	PITG_05007			22208	-0.303	-0.7662	No
33	PITG_03093	PITG_03093			22409	-0.359	-0.7720	No
34	PITG_10193	PITG_10193			22606	-0.404	-0.7775	No
35	PITG_11734	PITG_11734			22780	-0.455	-0.7819	No
36	PITG_16328	PITG_16328			22919	-0.491	-0.7850	No
37	PITG_22249	PITG_22249			22933	-0.496	-0.7834	No
38	PITG_09846	PITG_09846			22986	-0.514	-0.7831	No

39	PITG_12961	PITG_12961			23230	-0.590	-0.7896	No
40	PITG_16741	PITG_16741			23328	-0.615	-0.7906	No
41	PITG_08369	PITG_08369			23360	-0.629	-0.7891	No
42	PITG_04918	PITG_04918			23385	-0.637	-0.7873	No
43	PITG_00757	PITG_00757			23392	-0.640	-0.7849	No
44	PITG_03799	PITG_03799			23448	-0.655	-0.7842	No
45	PITG_05354	PITG_05354			23584	-0.710	-0.7862	No
46	PITG_02580	PITG_02580			23651	-0.734	-0.7856	No
47	PITG_17748	PITG_17748			23795	-0.780	-0.7876	No
48	PITG_00754	PITG_00754			24106	-0.902	-0.7951	Yes
49	PITG_21071	PITG_21071			24126	-0.913	-0.7921	Yes
50	PITG_14456	PITG_14456			24139	-0.918	-0.7887	Yes
51	PITG_19669	PITG_19669			24217	-0.958	-0.7875	Yes
52	PITG_06222	PITG_06222			24228	-0.961	-0.7839	Yes
53	PITG_11111	PITG_11111			24301	-0.988	-0.7825	Yes
54	PITG_05733	PITG_05733			24305	-0.989	-0.7785	Yes
55	PITG_17153	PITG_17153			24348	-1.006	-0.7758	Yes
56	PITG_01762	PITG_01762			24386	-1.026	-0.7730	Yes
57	PITG_16757	PITG_16757			24413	-1.036	-0.7696	Yes
58	PITG_18251	PITG_18251			24457	-1.057	-0.7668	Yes
59	PITG_03322	PITG_03322			24462	-1.057	-0.7626	Yes
60	PITG_10979	PITG_10979			24567	-1.100	-0.7618	Yes
61	PITG_07797	PITG_07797			24662	-1.147	-0.7605	Yes
62	PITG_07888	PITG_07888			24666	-1.148	-0.7559	Yes
63	PITG_03274	PITG_03274			24715	-1.175	-0.7528	Yes
64	PITG_14609	PITG_14609			24732	-1.182	-0.7484	Yes
65	PITG_05405	PITG_05405			24742	-1.189	-0.7439	Yes
66	PITG_07841	PITG_07841			24743	-1.190	-0.7389	Yes
67	PITG_04703	PITG_04703			24756	-1.196	-0.7344	Yes
68	PITG_19999	PITG_19999			24792	-1.214	-0.7307	Yes
69	PITG_12864	PITG_12864			24801	-1.219	-0.7259	Yes
70	PITG_03480	PITG_03480			24930	-1.293	-0.7252	Yes
71	PITG_20189	PITG_20189			24966	-1.317	-0.7211	Yes
72	PITG_01922	PITG_01922			25002	-1.336	-0.7168	Yes
73	PITG_12839	PITG_12839			25031	-1.352	-0.7122	Yes
74	PITG_14850	PITG_14850			25066	-1.380	-0.7078	Yes
75	PITG_11923	PITG_11923			25096	-1.398	-0.7030	Yes
76	PITG_16008	PITG_16008			25114	-1.410	-0.6978	Yes
77	PITG_06771	PITG_06771			25118	-1.413	-0.6921	Yes

78	PITG_15723	PITG_15723			25159	-1.438	-0.6876	Yes
79	PITG_07141	PITG_07141			25165	-1.441	-0.6818	Yes
80	PITG_04992	PITG_04992			25172	-1.445	-0.6761	Yes
81	PITG_00443	PITG_00443			25193	-1.456	-0.6708	Yes
82	PITG_04843	PITG_04843			25217	-1.471	-0.6655	Yes
83	PITG_05171	PITG_05171			25340	-1.548	-0.6636	Yes
84	PITG_12745	PITG_12745			25347	-1.553	-0.6573	Yes
85	PITG_10974	PITG_10974			25362	-1.559	-0.6514	Yes
86	PITG_08206	PITG_08206			25382	-1.567	-0.6456	Yes
87	PITG_20188	PITG_20188			25502	-1.654	-0.6431	Yes
88	PITG_12697	PITG_12697			25529	-1.673	-0.6371	Yes
89	PITG_04729	PITG_04729			25577	-1.699	-0.6318	Yes
90	PITG_09234	PITG_09234			25595	-1.710	-0.6254	Yes
91	PITG_10887	PITG_10887			25597	-1.713	-0.6183	Yes
92	PITG_07300	PITG_07300			25611	-1.727	-0.6116	Yes
93	PITG_03221	PITG_03221			25613	-1.729	-0.6045	Yes
94	PITG_15090	PITG_15090			25650	-1.757	-0.5986	Yes
95	PITG_04774	PITG_04774			25653	-1.761	-0.5913	Yes
96	PITG_08703	PITG_08703			25716	-1.807	-0.5861	Yes
97	PITG_01255	PITG_01255			25748	-1.835	-0.5797	Yes
98	PITG_10110	PITG_10110			25769	-1.847	-0.5728	Yes
99	PITG_02694	PITG_02694			25772	-1.849	-0.5652	Yes
100	PITG_15069	PITG_15069			25785	-1.859	-0.5579	Yes
101	PITG_01943	PITG_01943			25791	-1.864	-0.5504	Yes
102	PITG_15407	PITG_15407			25795	-1.868	-0.5428	Yes
103	PITG_11766	PITG_11766			25814	-1.881	-0.5356	Yes
104	PITG_01833	PITG_01833			25831	-1.897	-0.5284	Yes
105	PITG_02921	PITG_02921			25864	-1.929	-0.5216	Yes
106	PITG_08579	PITG_08579			25907	-1.965	-0.5150	Yes
107	PITG_20264	PITG_20264			25950	-1.992	-0.5082	Yes
108	PITG_07173	PITG_07173			25980	-2.022	-0.5009	Yes
109	PITG_19157	PITG_19157			25981	-2.022	-0.4926	Yes
110	PITG_10863	PITG_10863			25982	-2.022	-0.4842	Yes
111	PITG_03460	PITG_03460			25987	-2.027	-0.4760	Yes
112	PITG_09540	PITG_09540			25997	-2.035	-0.4679	Yes
113	PITG_03661	PITG_03661			26015	-2.048	-0.4600	Yes
114	PITG_03353	PITG_03353			26026	-2.059	-0.4518	Yes
115	PITG_13831	PITG_13831			26029	-2.061	-0.4434	Yes
116	PITG_09506	PITG_09506			26047	-2.074	-0.4354	Yes

117	PITG_00941	PITG_00941			26052	-2.078	-0.4270	Yes
118	PITG_14913	PITG_14913			26073	-2.104	-0.4190	Yes
119	PITG_04487	PITG_04487			26098	-2.129	-0.4111	Yes
120	PITG_00302	PITG_00302			26103	-2.131	-0.4024	Yes
121	PITG_04747	PITG_04747			26111	-2.138	-0.3938	Yes
122	PITG_03235	PITG_03235			26130	-2.165	-0.3855	Yes
123	PITG_19531	PITG_19531			26134	-2.169	-0.3766	Yes
124	PITG_04382	PITG_04382			26147	-2.181	-0.3680	Yes
125	PITG_06995	PITG_06995			26169	-2.197	-0.3597	Yes
126	PITG_08959	PITG_08959			26174	-2.203	-0.3507	Yes
127	PITG_05174	PITG_05174			26178	-2.205	-0.3417	Yes
128	PITG_03239	PITG_03239			26180	-2.206	-0.3326	Yes
129	PITG_03294	PITG_03294			26205	-2.241	-0.3242	Yes
130	PITG_09555	PITG_09555			26249	-2.286	-0.3163	Yes
131	Novel00015	Novel00015			26276	-2.309	-0.3077	Yes
132	PITG_17785	PITG_17785			26278	-2.310	-0.2982	Yes
133	PITG_12947	PITG_12947			26294	-2.326	-0.2891	Yes
134	PITG_18052	PITG_18052			26295	-2.327	-0.2795	Yes
135	PITG_12077	PITG_12077			26335	-2.375	-0.2711	Yes
136	PITG_13371	PITG_13371			26336	-2.376	-0.2613	Yes
137	PITG_06636	PITG_06636			26348	-2.389	-0.2518	Yes
138	PITG_03178	PITG_03178			26392	-2.437	-0.2433	Yes
139	PITG_00523	PITG_00523			26405	-2.460	-0.2335	Yes
140	PITG_09631	PITG_09631			26454	-2.515	-0.2249	Yes
141	PITG_13681	PITG_13681			26460	-2.519	-0.2146	Yes
142	PITG_10146	PITG_10146			26517	-2.576	-0.2060	Yes
143	PITG_02578	PITG_02578			26546	-2.608	-0.1962	Yes
144	PITG_06237	PITG_06237			26557	-2.626	-0.1857	Yes
145	PITG_00910	PITG_00910			26570	-2.639	-0.1752	Yes
146	PITG_17607	PITG_17607			26591	-2.668	-0.1649	Yes
147	PITG_03420	PITG_03420			26604	-2.687	-0.1543	Yes
148	PITG_01042	PITG_01042			26613	-2.704	-0.1433	Yes
149	PITG_03999	PITG_03999			26623	-2.720	-0.1324	Yes
150	PITG_09521	PITG_09521			26652	-2.752	-0.1221	Yes
151	PITG_02039	PITG_02039			26671	-2.781	-0.1112	Yes
152	PITG_09552	PITG_09552			26705	-2.829	-0.1007	Yes
153	PITG_14729	PITG_14729			26712	-2.839	-0.0892	Yes
154	PITG_07991	PITG_07991			26768	-2.940	-0.0790	Yes
155	PITG_00397	PITG_00397			26815	-3.015	-0.0682	Yes

156	PITG_03768	PITG_03768			26845	-3.062	-0.0566	Yes
157	PITG_06821	PITG_06821			26849	-3.067	-0.0440	Yes
158	PITG_08714	PITG_08714			26862	-3.088	-0.0317	Yes
159	PITG_18054	PITG_18054			26907	-3.165	-0.0202	Yes
160	PITG_06596	PITG_06596			27055	-3.399	-0.0115	Yes
161	PITG_21661	PITG_21661			27155	-3.586	-0.0002	Yes
162	PITG_22020	PITG_22020			27176	-3.631	0.0141	Yes

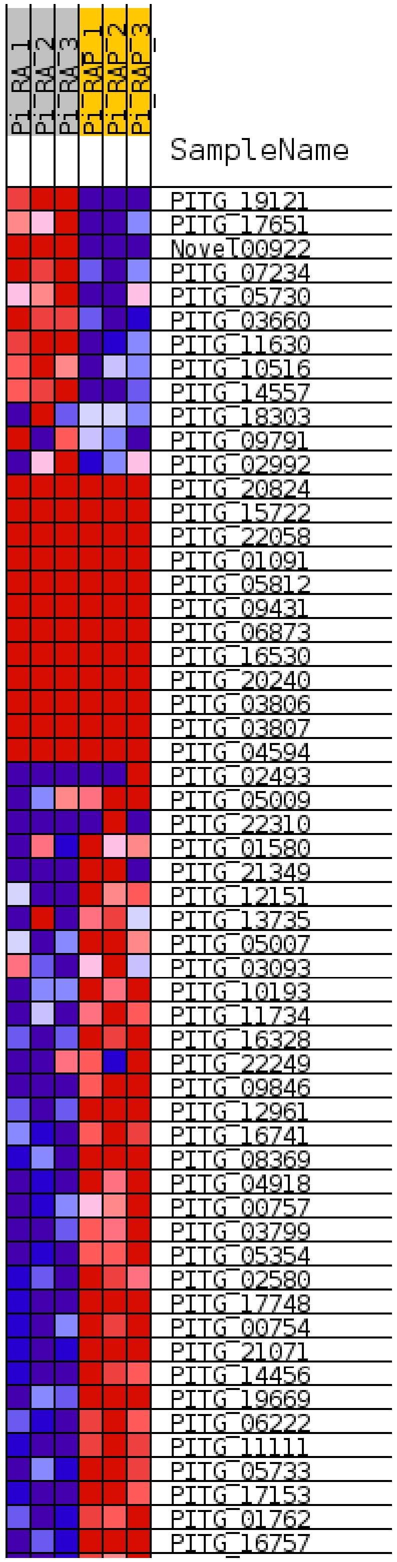

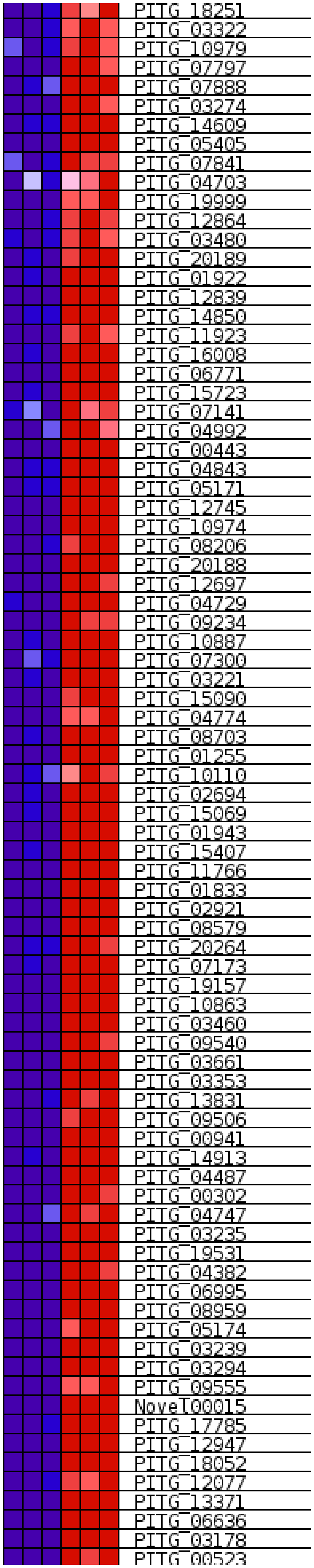
PITG_18251
PITG_03322
PITG_10979
PITG_07797
PITG_07888
PITG_03274
PITG_14609
PITG_05405
PITG_07841
PITG_04703
PITG_19999
PITG_12864
PITG_03480
PITG_20189
PITG_01922
PITG_12839
PITG_14850
PITG_11923
PITG_16008
PITG_06771
PITG_15723
PITG_07141
PITG_04992
PITG_00443
PITG_04843
PITG_05171
PITG_12745
PITG_10974
PITG_08206
PITG_20188
PITG_12697
PITG_04729
PITG_09234
PITG_10887
PITG_07300
PITG_03221
PITG_15090
PITG_04774
PITG_08703
PITG_01255
PITG_10110
PITG_02694
PITG_15069
PITG_01943
PITG_15407
PITG_11766
PITG_01833
PITG_02921
PITG_08579
PITG_20264
PITG_07173
PITG_19157
PITG_10863
PITG_03460
PITG_09540
PITG_03661
PITG_03353
PITG_13831
PITG_09506
PITG_00941
PITG_14913
PITG_04487
PITG_00302
PITG_04747
PITG_03235
PITG_19531
PITG_04382
PITG_06995
PITG_08959
PITG_05174
PITG_03239
PITG_03294
PITG_09555
Novel00015
PITG_17785
PITG_12947
PITG_18052
PITG_12077
PITG_13371
PITG_06636
PITG_03178
PITG_00523

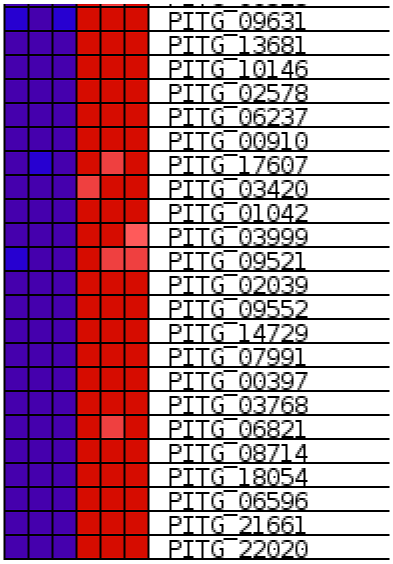

Fig 2: PEPTIDE_METABOLIC_PROCESS(GO:0006518)
Blue-Pink O' Gram in the Space of the Analyzed GeneSet

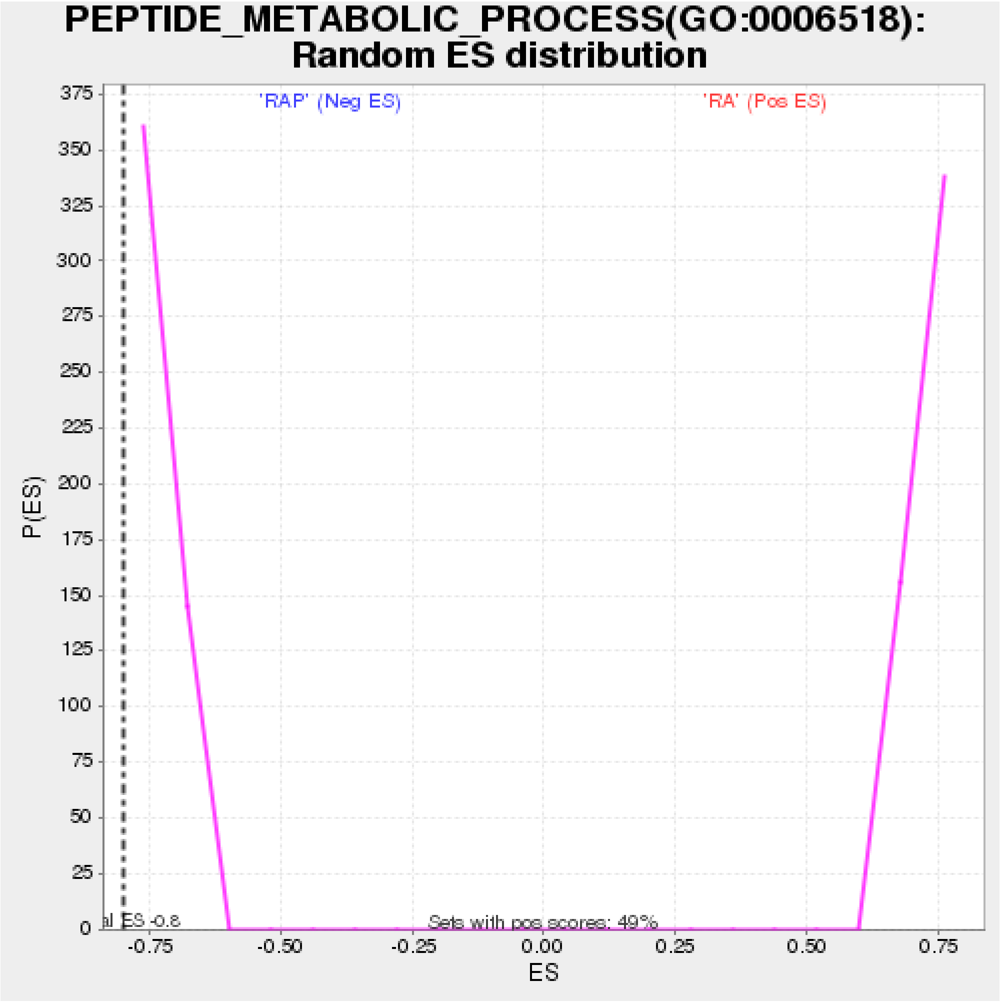

Fig 3: PEPTIDE_METABOLIC_PROCESS(GO:0006518): Random ES distribution
Gene set null distribution of ES for PEPTIDE_METABOLIC_PROCESS(GO:0006518)

6. ribonucleoprotein complex

Table: GSEA Results Summary

Dataset	fpkm.sample
Phenotype	sample.cls
Upregulated in class	RAP
GeneSet	RIBONUCLEOPROTEIN_COMPLEX(GO:1990904)
Enrichment Score (ES)	-0.8102501
Normalized Enrichment Score (NES)	-1.0889138
Nominal p-value	0.0
FDR q-value	0.07712077
FWER p-Value	0.06

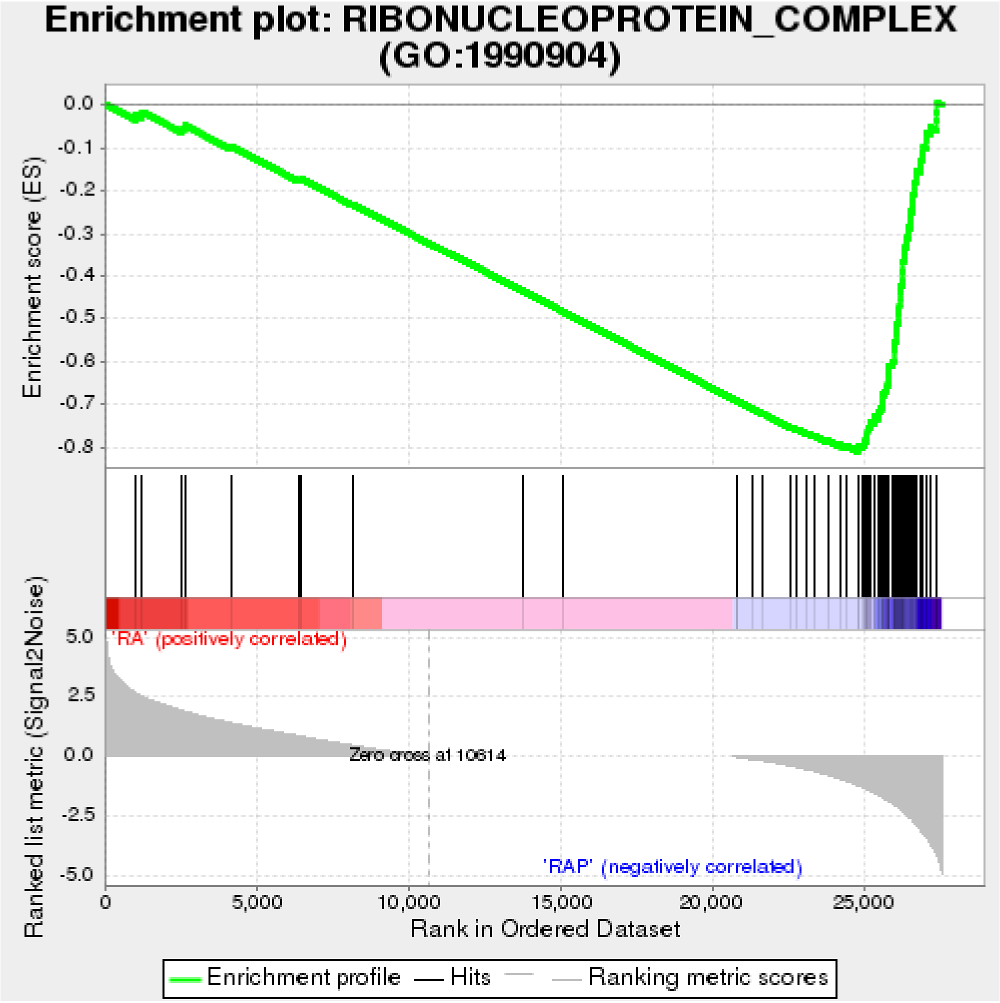

Fig 1: Enrichment plot: RIBONUCLEOPROTEIN_COMPLEX(GO:1990904)
Profile of the Running ES Score & Positions of GeneSet Members on the Rank Ordered List

Table: GSEA details [plain text format]

	PROBE	DESCRIPTION	GENE	GENE_TITLE	RANK IN	RANK	RUNNING	CORE

		(from dataset)	SYMBOL		GENE LIST	METRIC SCORE	ES	ENRICHMENT
1	PITG_19121	PITG_19121			962	2.666	-0.0212	No
2	PITG_03040	PITG_03040			1181	2.509	-0.0162	No
3	PITG_05680	PITG_05680			2502	1.920	-0.0543	No
4	PITG_12932	PITG_12932			2577	1.892	-0.0472	No
5	PITG_01925	PITG_01925			4127	1.404	-0.0964	No
6	PITG_10482	PITG_10482			6376	0.851	-0.1738	No
7	PITG_03660	PITG_03660			6456	0.832	-0.1724	No
8	PITG_04395	PITG_04395			8123	0.461	-0.2307	No
9	PITG_15722	PITG_15722			13740	0.000	-0.4352	No
10	PITG_09431	PITG_09431			15054	0.000	-0.4830	No
11	PITG_17166	PITG_17166			20773	-0.020	-0.6911	No
12	PITG_14854	PITG_14854			20790	-0.023	-0.6915	No
13	PITG_05009	PITG_05009			21324	-0.134	-0.7103	No
14	PITG_01171	PITG_01171			21663	-0.191	-0.7216	No
15	PITG_10193	PITG_10193			22606	-0.404	-0.7538	No
16	PITG_11734	PITG_11734			22780	-0.455	-0.7577	No
17	PITG_17727	PITG_17727			23133	-0.558	-0.7677	No
18	PITG_02996	PITG_02996			23344	-0.623	-0.7721	No
19	PITG_01246	PITG_01246			23821	-0.792	-0.7853	No
20	PITG_19669	PITG_19669			24217	-0.958	-0.7947	No
21	PITG_16757	PITG_16757			24413	-1.036	-0.7965	No
22	PITG_19999	PITG_19999			24792	-1.214	-0.8040	Yes
23	PITG_19007	PITG_19007			24804	-1.221	-0.7981	Yes
24	PITG_20189	PITG_20189			24966	-1.317	-0.7971	Yes
25	PITG_01922	PITG_01922			25002	-1.336	-0.7915	Yes
26	PITG_12839	PITG_12839			25031	-1.352	-0.7855	Yes
27	PITG_14850	PITG_14850			25066	-1.380	-0.7796	Yes
28	PITG_11923	PITG_11923			25096	-1.398	-0.7734	Yes
29	PITG_16008	PITG_16008			25114	-1.410	-0.7667	Yes
30	PITG_06771	PITG_06771			25118	-1.413	-0.7595	Yes
31	PITG_15723	PITG_15723			25159	-1.438	-0.7535	Yes
32	PITG_00443	PITG_00443			25193	-1.456	-0.7472	Yes
33	PITG_04843	PITG_04843			25217	-1.471	-0.7404	Yes
34	PITG_05171	PITG_05171			25340	-1.548	-0.7369	Yes
35	PITG_12745	PITG_12745			25347	-1.553	-0.7290	Yes
36	PITG_10725	PITG_10725			25496	-1.649	-0.7259	Yes
37	PITG_20188	PITG_20188			25502	-1.654	-0.7175	Yes
38	PITG_12697	PITG_12697			25529	-1.673	-0.7098	Yes

39	PITG_17733	PITG_17733			25582	-1.702	-0.7029	Yes
40	PITG_10887	PITG_10887			25597	-1.713	-0.6945	Yes
41	PITG_07300	PITG_07300			25611	-1.727	-0.6861	Yes
42	PITG_03221	PITG_03221			25613	-1.729	-0.6771	Yes
43	PITG_15090	PITG_15090			25650	-1.757	-0.6694	Yes
44	PITG_08703	PITG_08703			25716	-1.807	-0.6624	Yes
45	PITG_02694	PITG_02694			25772	-1.849	-0.6548	Yes
46	PITG_15069	PITG_15069			25785	-1.859	-0.6456	Yes
47	PITG_01943	PITG_01943			25791	-1.864	-0.6362	Yes
48	PITG_15407	PITG_15407			25795	-1.868	-0.6266	Yes
49	PITG_11766	PITG_11766			25814	-1.881	-0.6175	Yes
50	PITG_01833	PITG_01833			25831	-1.897	-0.6083	Yes
51	PITG_20264	PITG_20264			25950	-1.992	-0.6023	Yes
52	PITG_07173	PITG_07173			25980	-2.022	-0.5929	Yes
53	PITG_19157	PITG_19157			25981	-2.022	-0.5824	Yes
54	PITG_10863	PITG_10863			25982	-2.022	-0.5719	Yes
55	PITG_09540	PITG_09540			25997	-2.035	-0.5619	Yes
56	PITG_03353	PITG_03353			26026	-2.059	-0.5523	Yes
57	PITG_09506	PITG_09506			26047	-2.074	-0.5423	Yes
58	PITG_00941	PITG_00941			26052	-2.078	-0.5317	Yes
59	PITG_14913	PITG_14913			26073	-2.104	-0.5215	Yes
60	PITG_04487	PITG_04487			26098	-2.129	-0.5114	Yes
61	PITG_03235	PITG_03235			26130	-2.165	-0.5013	Yes
62	PITG_19531	PITG_19531			26134	-2.169	-0.4902	Yes
63	PITG_04382	PITG_04382			26147	-2.181	-0.4793	Yes
64	PITG_06995	PITG_06995			26169	-2.197	-0.4687	Yes
65	PITG_08959	PITG_08959			26174	-2.203	-0.4574	Yes
66	PITG_05174	PITG_05174			26178	-2.205	-0.4461	Yes
67	PITG_03239	PITG_03239			26180	-2.206	-0.4348	Yes
68	PITG_03294	PITG_03294			26205	-2.241	-0.4240	Yes
69	PITG_09555	PITG_09555			26249	-2.286	-0.4138	Yes
70	Novel00015	Novel00015			26276	-2.309	-0.4028	Yes
71	PITG_17785	PITG_17785			26278	-2.310	-0.3909	Yes
72	PITG_12947	PITG_12947			26294	-2.326	-0.3794	Yes
73	PITG_18052	PITG_18052			26295	-2.327	-0.3673	Yes
74	PITG_03818	PITG_03818			26310	-2.341	-0.3557	Yes
75	PITG_13371	PITG_13371			26336	-2.376	-0.3443	Yes
76	PITG_06636	PITG_06636			26348	-2.389	-0.3324	Yes
77	PITG_03178	PITG_03178			26392	-2.437	-0.3213	Yes

78	PITG_00523	PITG_00523			26405	-2.460	-0.3090	Yes
79	PITG_09631	PITG_09631			26454	-2.515	-0.2978	Yes
80	PITG_13681	PITG_13681			26460	-2.519	-0.2849	Yes
81	PITG_10146	PITG_10146			26517	-2.576	-0.2736	Yes
82	PITG_02578	PITG_02578			26546	-2.608	-0.2611	Yes
83	PITG_06237	PITG_06237			26557	-2.626	-0.2479	Yes
84	PITG_03420	PITG_03420			26604	-2.687	-0.2357	Yes
85	PITG_01042	PITG_01042			26613	-2.704	-0.2220	Yes
86	PITG_14730	PITG_14730			26625	-2.727	-0.2083	Yes
87	Novel01297	Novel01297			26646	-2.747	-0.1948	Yes
88	PITG_09521	PITG_09521			26652	-2.752	-0.1807	Yes
89	PITG_09552	PITG_09552			26705	-2.829	-0.1680	Yes
90	PITG_14729	PITG_14729			26712	-2.839	-0.1535	Yes
91	PITG_03768	PITG_03768			26845	-3.062	-0.1425	Yes
92	PITG_12305	PITG_12305			26887	-3.143	-0.1277	Yes
93	PITG_18054	PITG_18054			26907	-3.165	-0.1120	Yes
94	PITG_12292	PITG_12292			26958	-3.253	-0.0970	Yes
95	PITG_06596	PITG_06596			27055	-3.399	-0.0829	Yes
96	PITG_01019	PITG_01019			27065	-3.415	-0.0655	Yes
97	PITG_12931	PITG_12931			27200	-3.684	-0.0513	Yes
98	PITG_02593	PITG_02593			27396	-4.103	-0.0372	Yes
99	PITG_02592	PITG_02592			27405	-4.144	-0.0161	Yes
100	PITG_17365	PITG_17365			27420	-4.193	0.0051	Yes

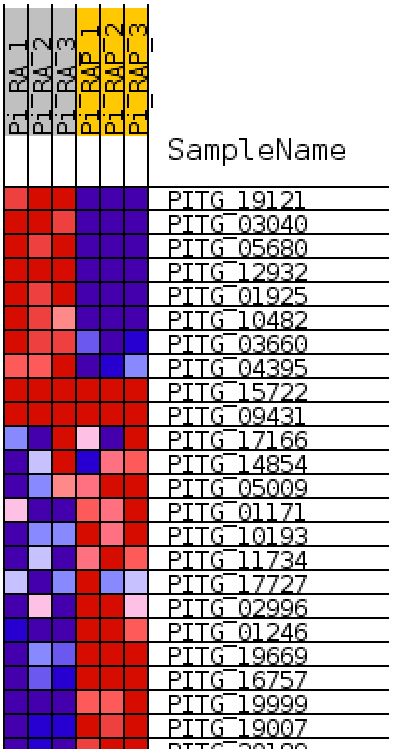

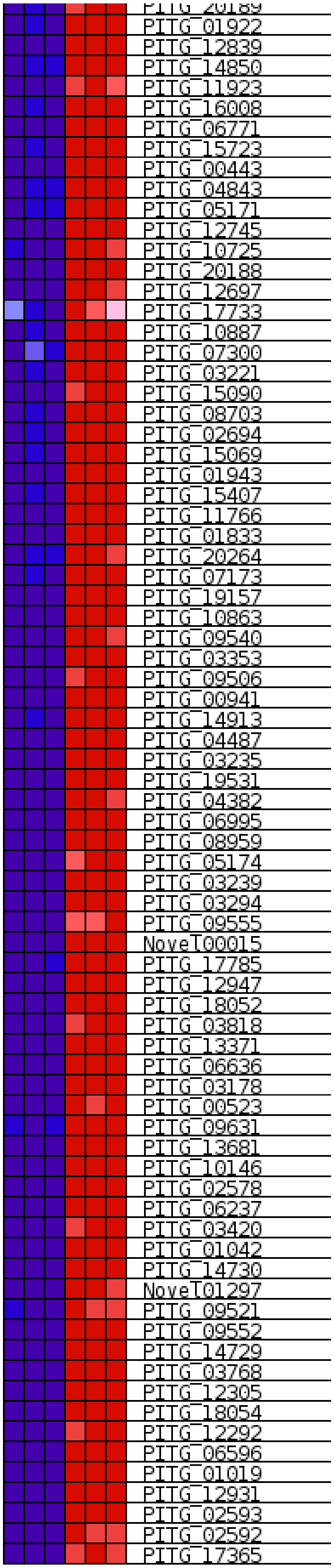

Fig 2: RIBONUCLEOPROTEIN_COMPLEX(GO:1990904)
Blue-Pink O' Gram in the Space of the Analyzed GeneSet

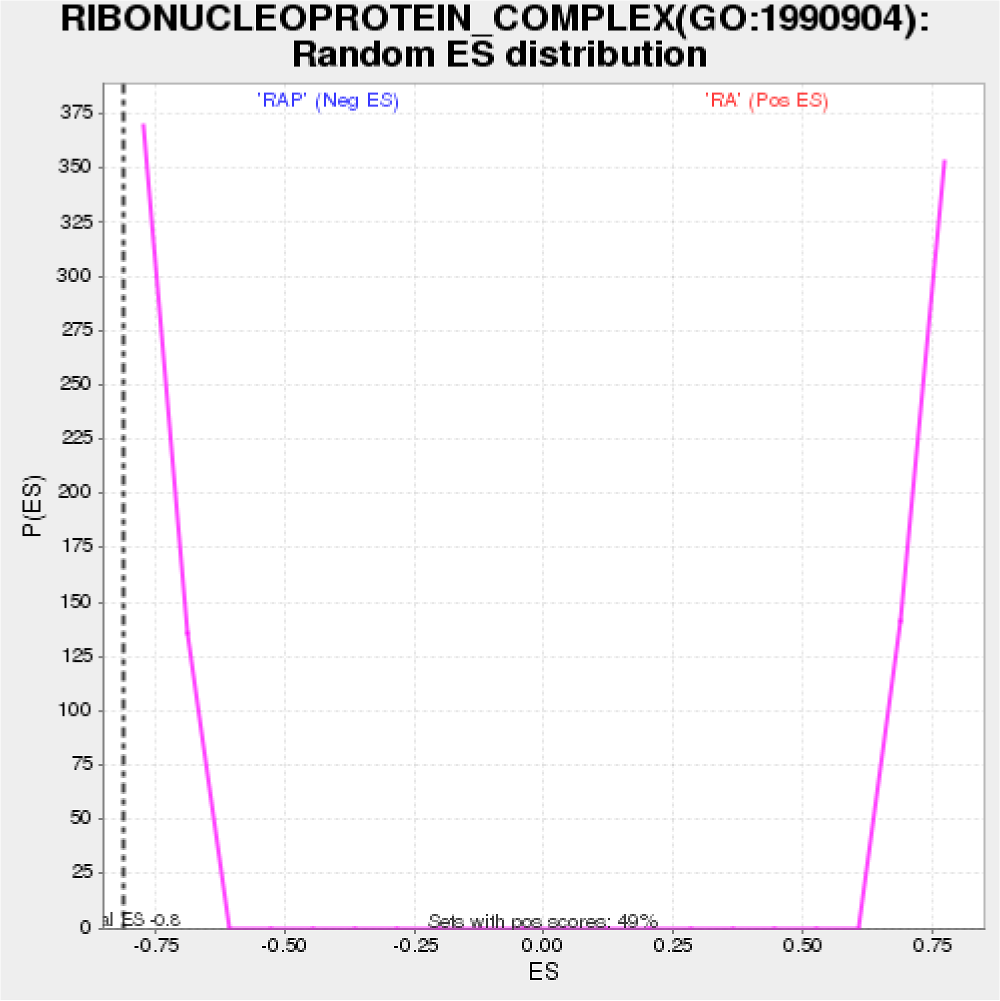

Fig 3: RIBONUCLEOPROTEIN_COMPLEX(GO:1990904): Random ES distribution
Gene set null distribution of ES for RIBONUCLEOPROTEIN_COMPLEX(GO:1990904)

7. Ribosome

Table: GSEA Results Summary

Dataset	fpkm.sample
Phenotype	sample.cls
Upregulated in class	RAP
GeneSet	RIBOSOME(GO:0005840)
Enrichment Score (ES)	-0.85688764
Normalized Enrichment Score (NES)	-1.0609467
Nominal p-value	0.0
FDR q-value	0.077120826
FWER p-Value	0.06

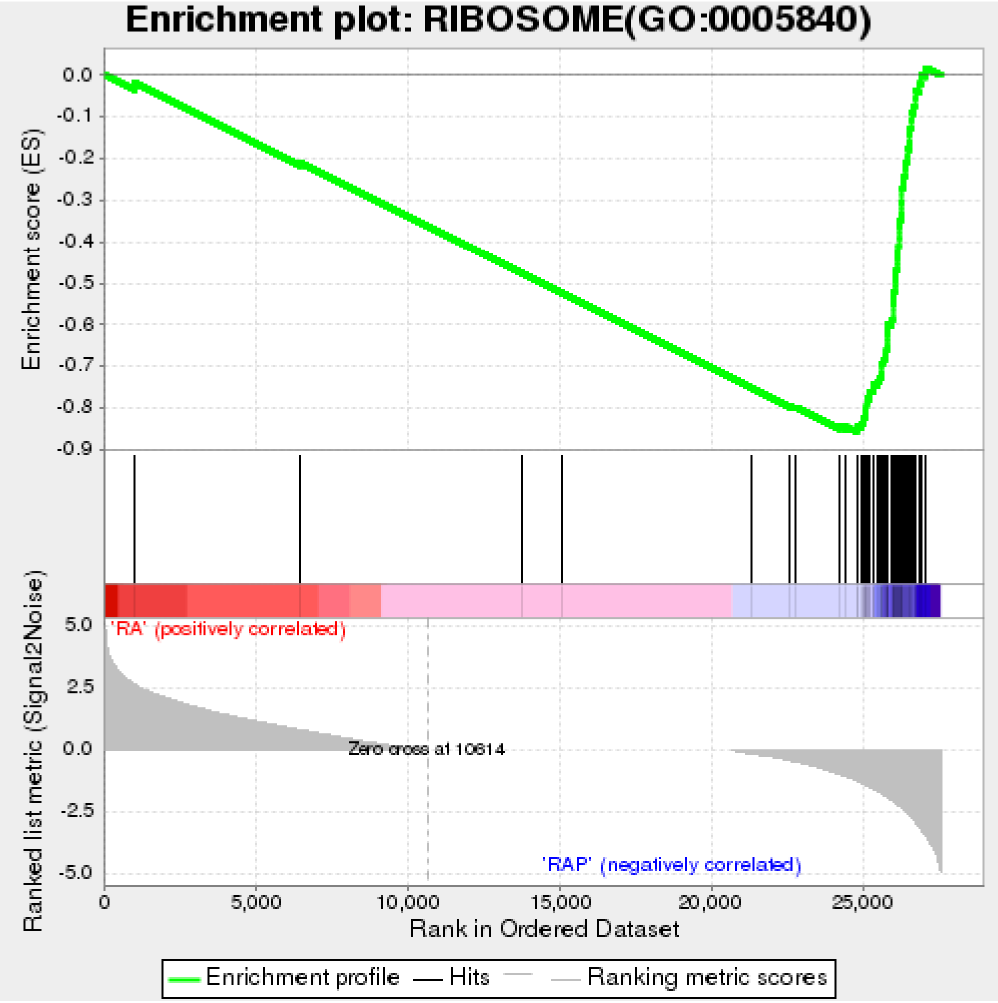

Fig 1: Enrichment plot: RIBOSOME(GO:0005840)
Profile of the Running ES Score & Positions of GeneSet Members on the Rank Ordered List

Table: GSEA details [plain text format]

	PROBE	DESCRIPTION	GENE	GENE_TITLE	RANK IN	RANK	RUNNING	CORE

		(from dataset)	SYMBOL		GENE LIST	METRIC SCORE	ES	ENRICHMENT
1	PITG_19121	PITG_19121			962	2.666	-0.0166	No
2	PITG_03660	PITG_03660			6456	0.832	-0.2107	No
3	PITG_15722	PITG_15722			13740	0.000	-0.4757	No
4	PITG_09431	PITG_09431			15054	0.000	-0.5234	No
5	PITG_05009	PITG_05009			21324	-0.134	-0.7506	No
6	PITG_10193	PITG_10193			22606	-0.404	-0.7944	No
7	PITG_11734	PITG_11734			22780	-0.455	-0.7976	No
8	PITG_19669	PITG_19669			24217	-0.958	-0.8432	No
9	PITG_16757	PITG_16757			24413	-1.036	-0.8431	No
10	PITG_19999	PITG_19999			24792	-1.214	-0.8485	Yes
11	PITG_19007	PITG_19007			24804	-1.221	-0.8405	Yes
12	PITG_20189	PITG_20189			24966	-1.317	-0.8373	Yes
13	PITG_01922	PITG_01922			25002	-1.336	-0.8293	Yes
14	PITG_12839	PITG_12839			25031	-1.352	-0.8210	Yes
15	PITG_14850	PITG_14850			25066	-1.380	-0.8127	Yes
16	PITG_11923	PITG_11923			25096	-1.398	-0.8041	Yes
17	PITG_16008	PITG_16008			25114	-1.410	-0.7950	Yes
18	PITG_06771	PITG_06771			25118	-1.413	-0.7854	Yes
19	PITG_15723	PITG_15723			25159	-1.438	-0.7769	Yes
20	PITG_00443	PITG_00443			25193	-1.456	-0.7680	Yes
21	PITG_04843	PITG_04843			25217	-1.471	-0.7587	Yes
22	PITG_05171	PITG_05171			25340	-1.548	-0.7525	Yes
23	PITG_12745	PITG_12745			25347	-1.553	-0.7420	Yes
24	PITG_20188	PITG_20188			25502	-1.654	-0.7362	Yes
25	PITG_12697	PITG_12697			25529	-1.673	-0.7255	Yes
26	PITG_10887	PITG_10887			25597	-1.713	-0.7162	Yes
27	PITG_07300	PITG_07300			25611	-1.727	-0.7047	Yes
28	PITG_03221	PITG_03221			25613	-1.729	-0.6928	Yes
29	PITG_15090	PITG_15090			25650	-1.757	-0.6820	Yes
30	PITG_08703	PITG_08703			25716	-1.807	-0.6719	Yes
31	PITG_02694	PITG_02694			25772	-1.849	-0.6611	Yes
32	PITG_15069	PITG_15069			25785	-1.859	-0.6487	Yes
33	PITG_01943	PITG_01943			25791	-1.864	-0.6360	Yes
34	PITG_15407	PITG_15407			25795	-1.868	-0.6233	Yes
35	PITG_11766	PITG_11766			25814	-1.881	-0.6109	Yes
36	PITG_01833	PITG_01833			25831	-1.897	-0.5984	Yes
37	PITG_20264	PITG_20264			25950	-1.992	-0.5890	Yes
38	PITG_07173	PITG_07173			25980	-2.022	-0.5761	Yes

39	PITG_19157	PITG_19157			25981	-2.022	-0.5621	Yes
40	PITG_10863	PITG_10863			25982	-2.022	-0.5482	Yes
41	PITG_09540	PITG_09540			25997	-2.035	-0.5346	Yes
42	PITG_03353	PITG_03353			26026	-2.059	-0.5214	Yes
43	PITG_09506	PITG_09506			26047	-2.074	-0.5078	Yes
44	PITG_00941	PITG_00941			26052	-2.078	-0.4936	Yes
45	PITG_14913	PITG_14913			26073	-2.104	-0.4798	Yes
46	PITG_04487	PITG_04487			26098	-2.129	-0.4660	Yes
47	PITG_03235	PITG_03235			26130	-2.165	-0.4522	Yes
48	PITG_19531	PITG_19531			26134	-2.169	-0.4373	Yes
49	PITG_04382	PITG_04382			26147	-2.181	-0.4227	Yes
50	PITG_06995	PITG_06995			26169	-2.197	-0.4083	Yes
51	PITG_08959	PITG_08959			26174	-2.203	-0.3933	Yes
52	PITG_05174	PITG_05174			26178	-2.205	-0.3781	Yes
53	PITG_03239	PITG_03239			26180	-2.206	-0.3630	Yes
54	PITG_03294	PITG_03294			26205	-2.241	-0.3484	Yes
55	PITG_09555	PITG_09555			26249	-2.286	-0.3341	Yes
56	Novel00015	Novel00015			26276	-2.309	-0.3192	Yes
57	PITG_17785	PITG_17785			26278	-2.310	-0.3032	Yes
58	PITG_12947	PITG_12947			26294	-2.326	-0.2877	Yes
59	PITG_18052	PITG_18052			26295	-2.327	-0.2717	Yes
60	PITG_13371	PITG_13371			26336	-2.376	-0.2567	Yes
61	PITG_06636	PITG_06636			26348	-2.389	-0.2406	Yes
62	PITG_03178	PITG_03178			26392	-2.437	-0.2254	Yes
63	PITG_00523	PITG_00523			26405	-2.460	-0.2088	Yes
64	PITG_09631	PITG_09631			26454	-2.515	-0.1932	Yes
65	PITG_13681	PITG_13681			26460	-2.519	-0.1760	Yes
66	PITG_10146	PITG_10146			26517	-2.576	-0.1603	Yes
67	PITG_02578	PITG_02578			26546	-2.608	-0.1433	Yes
68	PITG_06237	PITG_06237			26557	-2.626	-0.1255	Yes
69	PITG_03420	PITG_03420			26604	-2.687	-0.1087	Yes
70	PITG_01042	PITG_01042			26613	-2.704	-0.0903	Yes
71	PITG_09521	PITG_09521			26652	-2.752	-0.0727	Yes
72	PITG_09552	PITG_09552			26705	-2.829	-0.0550	Yes
73	PITG_14729	PITG_14729			26712	-2.839	-0.0357	Yes
74	PITG_03768	PITG_03768			26845	-3.062	-0.0193	Yes
75	PITG_18054	PITG_18054			26907	-3.165	0.0003	Yes
76	PITG_06596	PITG_06596			27055	-3.399	0.0184	Yes

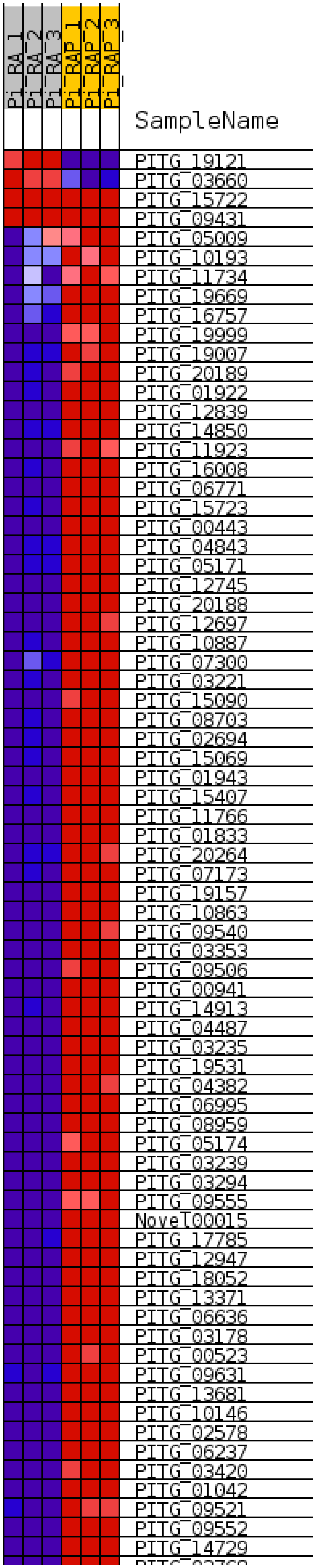

Pi_RA_1
Pi_RA_2
Pi_RA_3
Pi_RAP_1
Pi_RAP_2
Pi_RAP_3
SampleName
PITG_19121
PITG_03660
PITG_15722
PITG_09431
PITG_05009
PITG_10193
PITG_11734
PITG_19669
PITG_16757
PITG_19999
PITG_19007
PITG_20189
PITG_01922
PITG_12839
PITG_14850
PITG_11923
PITG_16008
PITG_06771
PITG_15723
PITG_00443
PITG_04843
PITG_05171
PITG_12745
PITG_20188
PITG_12697
PITG_10887
PITG_07300
PITG_03221
PITG_15090
PITG_08703
PITG_02694
PITG_15069
PITG_01943
PITG_15407
PITG_11766
PITG_01833
PITG_20264
PITG_07173
PITG_19157
PITG_10863
PITG_09540
PITG_03353
PITG_09506
PITG_00941
PITG_14913
PITG_04487
PITG_03235
PITG_19531
PITG_04382
PITG_06995
PITG_08959
PITG_05174
PITG_03239
PITG_03294
PITG_09555
Novel00015
PITG_17785
PITG_12947
PITG_18052
PITG_13371
PITG_06636
PITG_03178
PITG_00523
PITG_09631
PITG_13681
PITG_10146
PITG_02578
PITG_06237
PITG_03420
PITG_01042
PITG_09521
PITG_09552
PITG_14729

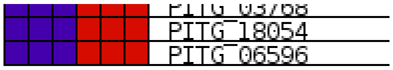

Fig 2: RIBOSOME(GO:0005840)
lue-Pink O' Gram in the Space of the Analyzed GeneSet

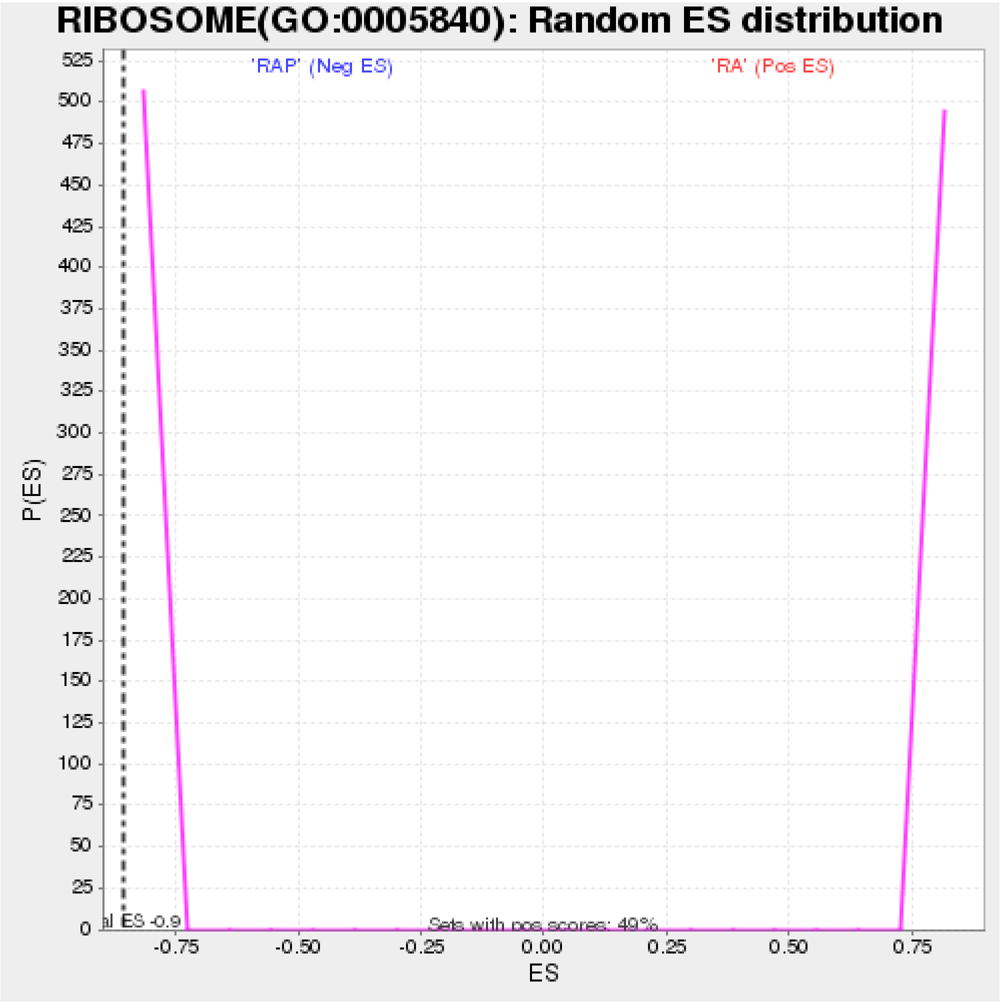

Fig 3: RIBOSOME(GO:0005840): Random ES distribution
Gene set null distribution of ES for RIBOSOME(GO:0005840)

8. RNA binding

Table: GSEA Results Summary

Dataset	fpkm.sample
Phenotype	sample.cls
Upregulated in class	RAP
GeneSet	RNA_BINDING(GO:0003723)
Enrichment Score (ES)	-0.5927679
Normalized Enrichment Score (NES)	-1.1506867
Nominal p-value	0.0
FDR q-value	0.077120826
FWER p-Value	0.06

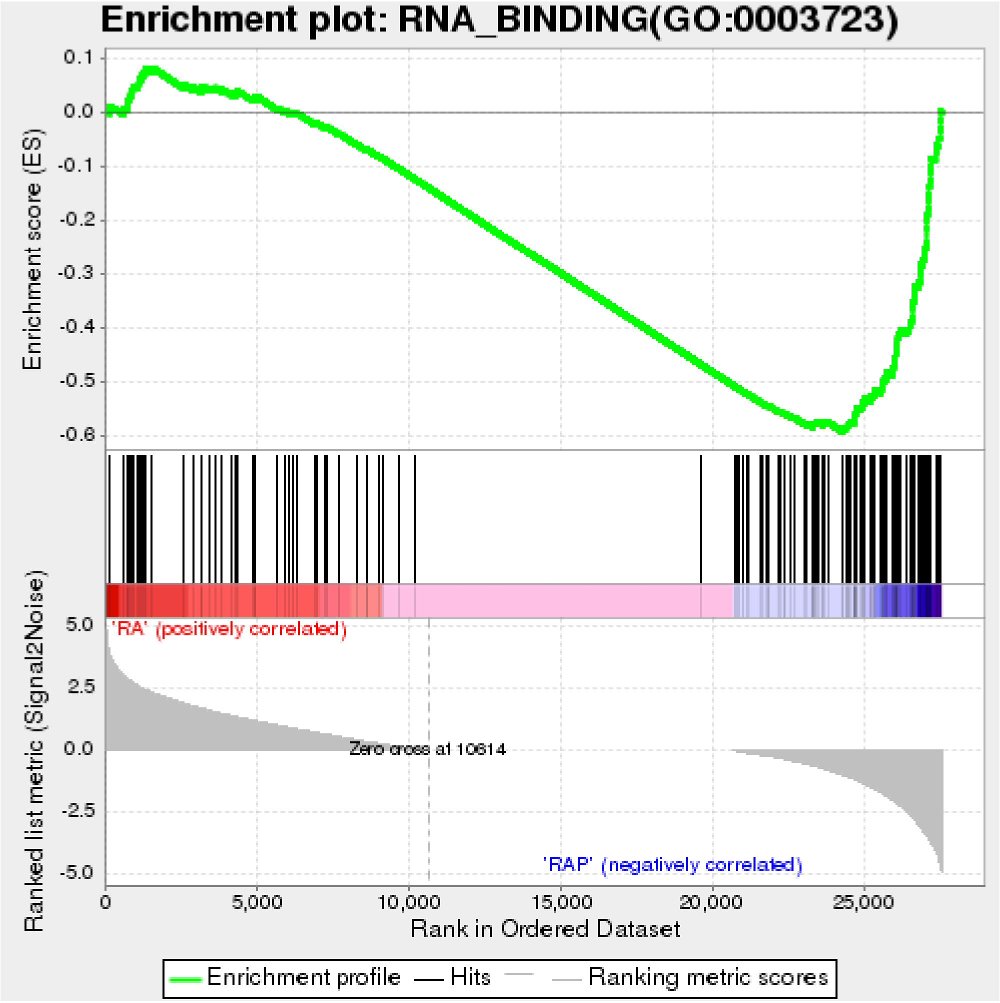

Fig 1: Enrichment plot: RNA_BINDING(GO:0003723)
Profile of the Running ES Score & Positions of GeneSet Members on the Rank Ordered List

Table: GSEA details [plain text format]

	PROBE	DESCRIPTION	GENE	GENE_TITLE	RANK IN	RANK	RUNNING	CORE

		(from dataset)	SYMBOL		GENE LIST	METRIC SCORE	ES	ENRICHMENT
1	PITG_08597	PITG_08597			97	3.943	0.0113	No
2	PITG_16523	PITG_16523			579	3.026	0.0051	No
3	PITG_12487	PITG_12487			679	2.907	0.0124	No
4	PITG_17224	PITG_17224			707	2.873	0.0222	No
5	PITG_12486	PITG_12486			787	2.812	0.0299	No
6	PITG_16517	PITG_16517			807	2.795	0.0397	No
7	PITG_16519	PITG_16519			860	2.741	0.0481	No
8	PITG_16524	PITG_16524			1053	2.600	0.0508	No
9	PITG_00483	PITG_00483			1060	2.596	0.0603	No
10	PITG_17222	PITG_17222			1139	2.535	0.0670	No
11	PITG_23106	PITG_23106			1236	2.472	0.0728	No
12	PITG_16522	PITG_16522			1302	2.437	0.0796	No
13	PITG_16521	PITG_16521			1500	2.340	0.0812	No
14	PITG_16548	PITG_16548			2543	1.906	0.0503	No
15	PITG_16516	PITG_16516			2849	1.791	0.0459	No
16	PITG_09292	PITG_09292			3141	1.696	0.0417	No
17	PITG_10574	PITG_10574			3156	1.691	0.0475	No
18	PITG_11433	PITG_11433			3422	1.607	0.0439	No
19	PITG_17407	PITG_17407			3562	1.561	0.0446	No
20	PITG_04376	PITG_04376			3807	1.487	0.0413	No
21	PITG_01925	PITG_01925			4127	1.404	0.0350	No
22	PITG_06886	PITG_06886			4277	1.361	0.0346	No
23	PITG_10167	PITG_10167			4305	1.352	0.0387	No
24	PITG_17651	PITG_17651			4826	1.224	0.0244	No
25	PITG_10857	PITG_10857			4907	1.203	0.0260	No
26	PITG_18633	PITG_18633			4925	1.199	0.0298	No
27	PITG_16378	PITG_16378			5664	1.009	0.0067	No
28	PITG_03118	PITG_03118			5880	0.967	0.0025	No
29	PITG_07234	PITG_07234			6061	0.926	-0.0006	No
30	PITG_08211	PITG_08211			6142	0.909	-0.0001	No
31	PITG_12355	PITG_12355			6318	0.864	-0.0033	No
32	PITG_06546	PITG_06546			6857	0.738	-0.0201	No
33	PITG_10481	PITG_10481			6955	0.717	-0.0210	No
34	PITG_17225	PITG_17225			7240	0.659	-0.0289	No
35	PITG_12797	PITG_12797			7304	0.643	-0.0287	No
36	PITG_07100	PITG_07100			7679	0.560	-0.0403	No
37	PITG_06610	PITG_06610			8287	0.427	-0.0608	No
38	PITG_11630	PITG_11630			8572	0.362	-0.0698	No

39	PITG_16369	PITG_16369			8595	0.356	-0.0693	No
40	PITG_14557	PITG_14557			8993	0.287	-0.0827	No
41	PITG_11369	PITG_11369			9123	0.261	-0.0864	No
42	PITG_10969	PITG_10969			9674	0.165	-0.1059	No
43	PITG_02992	PITG_02992			10209	0.072	-0.1251	No
44	PITG_17228	PITG_17228			19581	0.000	-0.4670	No
45	PITG_09243	PITG_09243			20743	-0.014	-0.5093	No
46	PITG_14854	PITG_14854			20790	-0.023	-0.5109	No
47	PITG_01846	PITG_01846			20894	-0.050	-0.5145	No
48	PITG_04538	PITG_04538			20990	-0.070	-0.5177	No
49	PITG_02493	PITG_02493			21136	-0.100	-0.5226	No
50	PITG_17930	PITG_17930			21185	-0.110	-0.5239	No
51	PITG_03778	PITG_03778			21565	-0.177	-0.5371	No
52	PITG_01171	PITG_01171			21663	-0.191	-0.5399	No
53	PITG_13735	PITG_13735			21784	-0.214	-0.5435	No
54	PITG_16012	PITG_16012			21869	-0.230	-0.5457	No
55	PITG_14278	PITG_14278			21882	-0.233	-0.5452	No
56	PITG_13806	PITG_13806			22211	-0.304	-0.5561	No
57	PITG_08831	PITG_08831			22238	-0.311	-0.5558	No
58	PITG_00967	PITG_00967			22354	-0.342	-0.5588	No
59	PITG_03093	PITG_03093			22409	-0.359	-0.5594	No
60	PITG_04079	PITG_04079			22581	-0.401	-0.5641	No
61	PITG_08684	PITG_08684			22704	-0.434	-0.5669	No
62	PITG_17964	PITG_17964			23040	-0.532	-0.5772	No
63	PITG_17727	PITG_17727			23133	-0.558	-0.5784	No
64	PITG_05536	PITG_05536			23288	-0.603	-0.5818	No
65	PITG_04567	PITG_04567			23300	-0.606	-0.5799	No
66	PITG_05826	PITG_05826			23331	-0.617	-0.5787	No
67	PITG_02996	PITG_02996			23344	-0.623	-0.5768	No
68	PITG_05366	PITG_05366			23395	-0.642	-0.5762	No
69	PITG_03799	PITG_03799			23448	-0.655	-0.5756	No
70	PITG_13146	PITG_13146			23511	-0.676	-0.5754	No
71	PITG_20903	PITG_20903			23649	-0.733	-0.5776	No
72	PITG_02580	PITG_02580			23651	-0.734	-0.5749	No
73	PITG_18998	PITG_18998			23706	-0.752	-0.5740	No
74	PITG_01246	PITG_01246			23821	-0.792	-0.5752	No
75	PITG_01045	PITG_01045			24303	-0.989	-0.5891	Yes
76	PITG_01036	PITG_01036			24321	-0.997	-0.5859	Yes
77	PITG_04666	PITG_04666			24393	-1.027	-0.5847	Yes

78	PITG_07890	PITG_07890			24455	-1.056	-0.5829	Yes
79	PITG_18251	PITG_18251			24457	-1.057	-0.5790	Yes
80	PITG_11653	PITG_11653			24491	-1.067	-0.5762	Yes
81	PITG_10979	PITG_10979			24567	-1.100	-0.5748	Yes
82	PITG_07797	PITG_07797			24662	-1.147	-0.5739	Yes
83	PITG_07888	PITG_07888			24666	-1.148	-0.5697	Yes
84	PITG_05649	PITG_05649			24675	-1.154	-0.5657	Yes
85	PITG_13500	PITG_13500			24681	-1.158	-0.5615	Yes
86	PITG_03274	PITG_03274			24715	-1.175	-0.5583	Yes
87	PITG_14609	PITG_14609			24732	-1.182	-0.5545	Yes
88	PITG_04703	PITG_04703			24756	-1.196	-0.5508	Yes
89	PITG_17779	PITG_17779			24773	-1.203	-0.5469	Yes
90	PITG_16525	PITG_16525			24903	-1.277	-0.5468	Yes
91	PITG_03480	PITG_03480			24930	-1.293	-0.5429	Yes
92	PITG_08724	PITG_08724			24936	-1.301	-0.5382	Yes
93	PITG_21447	PITG_21447			24996	-1.331	-0.5353	Yes
94	PITG_15623	PITG_15623			24997	-1.332	-0.5303	Yes
95	PITG_15217	PITG_15217			25214	-1.470	-0.5327	Yes
96	PITG_00462	PITG_00462			25220	-1.473	-0.5273	Yes
97	PITG_03201	PITG_03201			25312	-1.530	-0.5249	Yes
98	PITG_10974	PITG_10974			25362	-1.559	-0.5208	Yes
99	PITG_09563	PITG_09563			25371	-1.565	-0.5153	Yes
100	PITG_07885	PITG_07885			25526	-1.673	-0.5146	Yes
101	PITG_17733	PITG_17733			25582	-1.702	-0.5102	Yes
102	PITG_01795	PITG_01795			25584	-1.703	-0.5039	Yes
103	PITG_03221	PITG_03221			25613	-1.729	-0.4984	Yes
104	PITG_20377	PITG_20377			25669	-1.773	-0.4937	Yes
105	PITG_20401	PITG_20401			25736	-1.828	-0.4893	Yes
106	PITG_01255	PITG_01255			25748	-1.835	-0.4828	Yes
107	PITG_05603	PITG_05603			25914	-1.968	-0.4814	Yes
108	PITG_19551	PITG_19551			25930	-1.979	-0.4746	Yes
109	PITG_22434	PITG_22434			25978	-2.021	-0.4687	Yes
110	PITG_15393	PITG_15393			26008	-2.042	-0.4621	Yes
111	PITG_10201	PITG_10201			26016	-2.050	-0.4546	Yes
112	PITG_17240	PITG_17240			26039	-2.070	-0.4477	Yes
113	PITG_09506	PITG_09506			26047	-2.074	-0.4401	Yes
114	PITG_14747	PITG_14747			26074	-2.105	-0.4332	Yes
115	PITG_04487	PITG_04487			26098	-2.129	-0.4260	Yes
116	PITG_00302	PITG_00302			26103	-2.131	-0.4182	Yes

117	PITG_19531	PITG_19531			26134	-2.169	-0.4111	Yes
118	PITG_08959	PITG_08959			26174	-2.203	-0.4043	Yes
119	PITG_03178	PITG_03178			26392	-2.437	-0.4030	Yes
120	PITG_18184	PITG_18184			26500	-2.556	-0.3973	Yes
121	PITG_10146	PITG_10146			26517	-2.576	-0.3883	Yes
122	PITG_00910	PITG_00910			26570	-2.639	-0.3802	Yes
123	PITG_01248	PITG_01248			26590	-2.665	-0.3709	Yes
124	PITG_17607	PITG_17607			26591	-2.668	-0.3609	Yes
125	PITG_03999	PITG_03999			26623	-2.720	-0.3518	Yes
126	Novel01297	Novel01297			26646	-2.747	-0.3423	Yes
127	PITG_09987	PITG_09987			26655	-2.760	-0.3323	Yes
128	PITG_02039	PITG_02039			26671	-2.781	-0.3224	Yes
129	PITG_00397	PITG_00397			26815	-3.015	-0.3163	Yes
130	PITG_06821	PITG_06821			26849	-3.067	-0.3059	Yes
131	PITG_09728	PITG_09728			26854	-3.079	-0.2945	Yes
132	PITG_16015	PITG_16015			26883	-3.129	-0.2838	Yes
133	PITG_01035	PITG_01035			26931	-3.206	-0.2735	Yes
134	PITG_15740	PITG_15740			26976	-3.279	-0.2628	Yes
135	PITG_10166	PITG_10166			27016	-3.337	-0.2517	Yes
136	PITG_04428	PITG_04428			27050	-3.394	-0.2401	Yes
137	PITG_06981	PITG_06981			27063	-3.411	-0.2278	Yes
138	PITG_01215	PITG_01215			27064	-3.412	-0.2150	Yes
139	PITG_21196	PITG_21196			27072	-3.433	-0.2023	Yes
140	PITG_03764	PITG_03764			27087	-3.458	-0.1898	Yes
141	PITG_06262	PITG_06262			27099	-3.481	-0.1772	Yes
142	PITG_01495	PITG_01495			27113	-3.511	-0.1645	Yes
143	PITG_00558	PITG_00558			27144	-3.571	-0.1522	Yes
144	PITG_21661	PITG_21661			27155	-3.586	-0.1391	Yes
145	PITG_12483	PITG_12483			27164	-3.601	-0.1258	Yes
146	PITG_22020	PITG_22020			27176	-3.631	-0.1126	Yes
147	PITG_01680	PITG_01680			27195	-3.668	-0.0995	Yes
148	PITG_02450	PITG_02450			27206	-3.695	-0.0860	Yes
149	PITG_06827	PITG_06827			27358	-4.010	-0.0764	Yes
150	PITG_18688	PITG_18688			27378	-4.055	-0.0619	Yes
151	PITG_07651	PITG_07651			27481	-4.426	-0.0490	Yes
152	PITG_05512	PITG_05512			27495	-4.492	-0.0326	Yes
153	PITG_07308	PITG_07308			27517	-4.578	-0.0162	Yes
154	PITG_03308	PITG_03308			27541	-4.729	0.0007	Yes

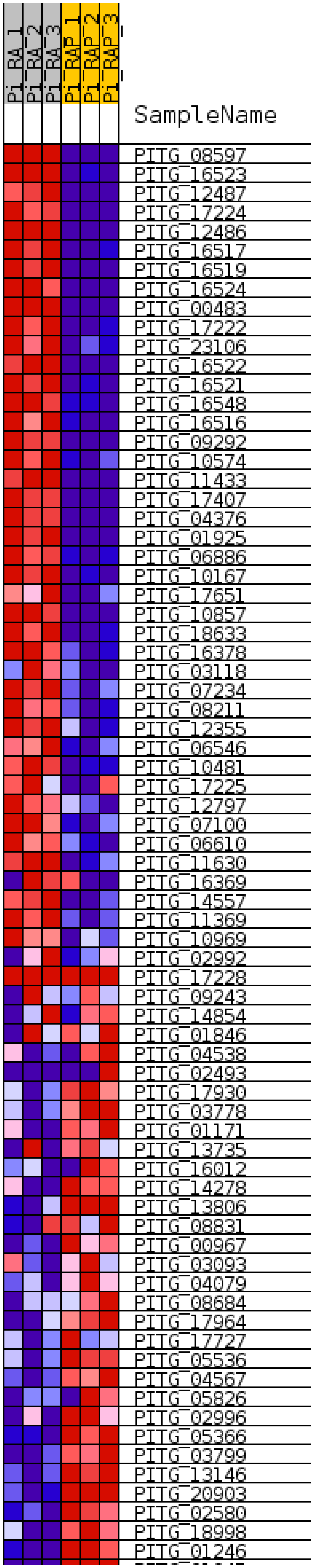
Pi_RA_1
Pi_RA_2
Pi_RA_3
Pi_RAP_1
Pi_RAP_2
Pi_RAP_3
SampleName
PITG_08597
PITG_16523
PITG_12487
PITG_17224
PITG_12486
PITG_16517
PITG_16519
PITG_16524
PITG_00483
PITG_17222
PITG_23106
PITG_16522
PITG_16521
PITG_16548
PITG_16516
PITG_09292
PITG_10574
PITG_11433
PITG_17407
PITG_04376
PITG_01925
PITG_06886
PITG_10167
PITG_17651
PITG_10857
PITG_18633
PITG_16378
PITG_03118
PITG_07234
PITG_08211
PITG_12355
PITG_06546
PITG_10481
PITG_17225
PITG_12797
PITG_07100
PITG_06610
PITG_11630
PITG_16369
PITG_14557
PITG_11369
PITG_10969
PITG_02992
PITG_17228
PITG_09243
PITG_14854
PITG_01846
PITG_04538
PITG_02493
PITG_17930
PITG_03778
PITG_01171
PITG_13735
PITG_16012
PITG_14278
PITG_13806
PITG_08831
PITG_00967
PITG_03093
PITG_04079
PITG_08684
PITG_17964
PITG_17727
PITG_05536
PITG_04567
PITG_05826
PITG_02996
PITG_05366
PITG_03799
PITG_13146
PITG_20903
PITG_02580
PITG_18998
PITG_01246

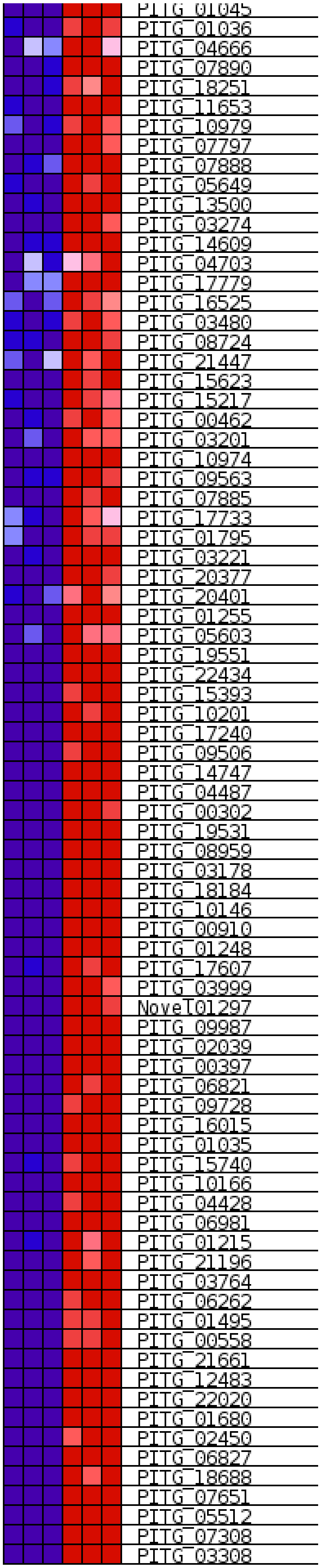
PITG_01045
PITG_01036
PITG_04666
PITG_07890
PITG_18251
PITG_11653
PITG_10979
PITG_07797
PITG_07888
PITG_05649
PITG_13500
PITG_03274
PITG_14609
PITG_04703
PITG_17779
PITG_16525
PITG_03480
PITG_08724
PITG_21447
PITG_15623
PITG_15217
PITG_00462
PITG_03201
PITG_10974
PITG_09563
PITG_07885
PITG_17733
PITG_01795
PITG_03221
PITG_20377
PITG_20401
PITG_01255
PITG_05603
PITG_19551
PITG_22434
PITG_15393
PITG_10201
PITG_17240
PITG_09506
PITG_14747
PITG_04487
PITG_00302
PITG_19531
PITG_08959
PITG_03178
PITG_18184
PITG_10146
PITG_00910
PITG_01248
PITG_17607
PITG_03999
Novel_01297
PITG_09987
PITG_02039
PITG_00397
PITG_06821
PITG_09728
PITG_16015
PITG_01035
PITG_15740
PITG_10166
PITG_04428
PITG_06981
PITG_01215
PITG_21196
PITG_03764
PITG_06262
PITG_01495
PITG_00558
PITG_21661
PITG_12483
PITG_22020
PITG_01680
PITG_02450
PITG_06827
PITG_18688
PITG_07651
PITG_05512
PITG_07308
PITG_03308

Fig 2: RNA_BINDING(GO:0003723)
Blue-Pink O' Gram in the Space of the Analyzed GeneSet

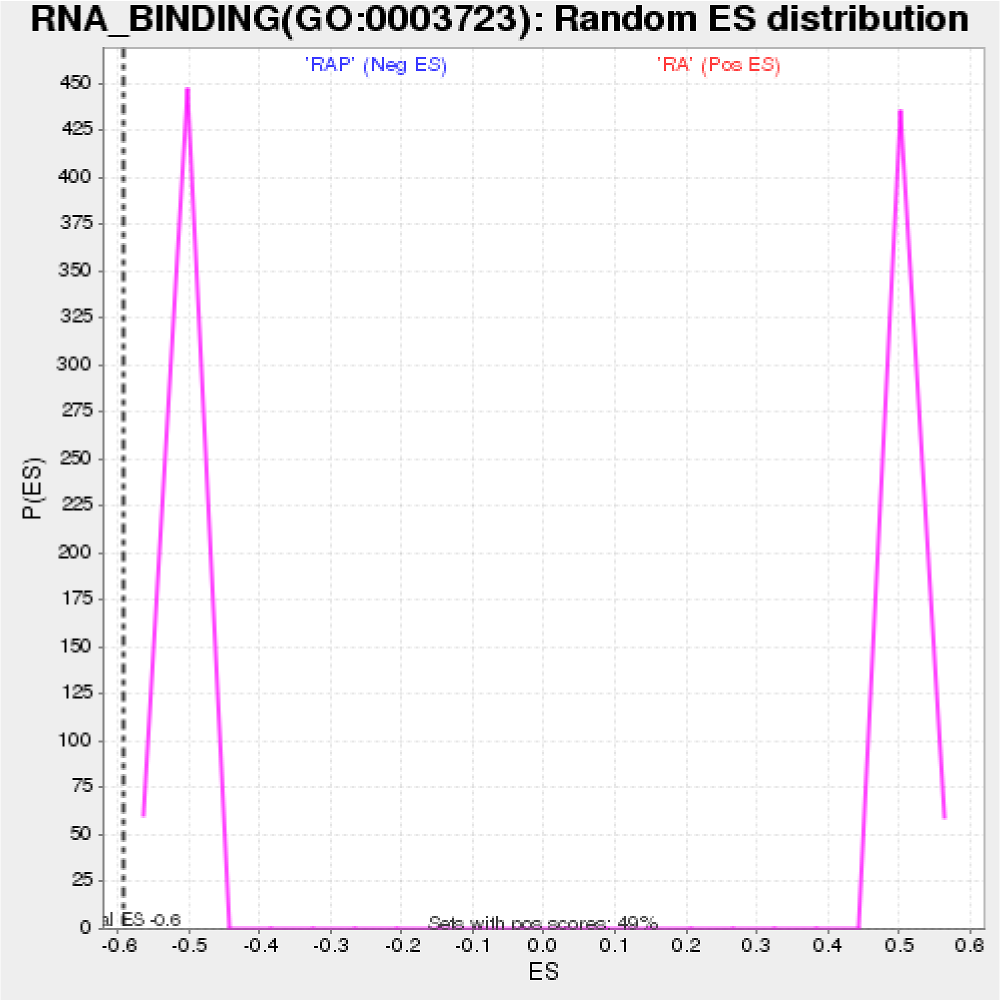

Fig 3: RNA_BINDING(GO:0003723): Random ES distribution
Gene set null distribution of ES for RNA_BINDING(GO:0003723)

9. structural constituent of ribosome

Table: GSEA Results Summary

Dataset	fpkm.sample
Phenotype	sample.cls
Upregulated in class	RAP
GeneSet	STRUCTURAL_CONSTITUENT_OF_RIBOSOME(GO:0003735)
Enrichment Score (ES)	-0.8530315
Normalized Enrichment Score (NES)	-1.067388
Nominal p-value	0.0
FDR q-value	0.07712086
FWER p-Value	0.06

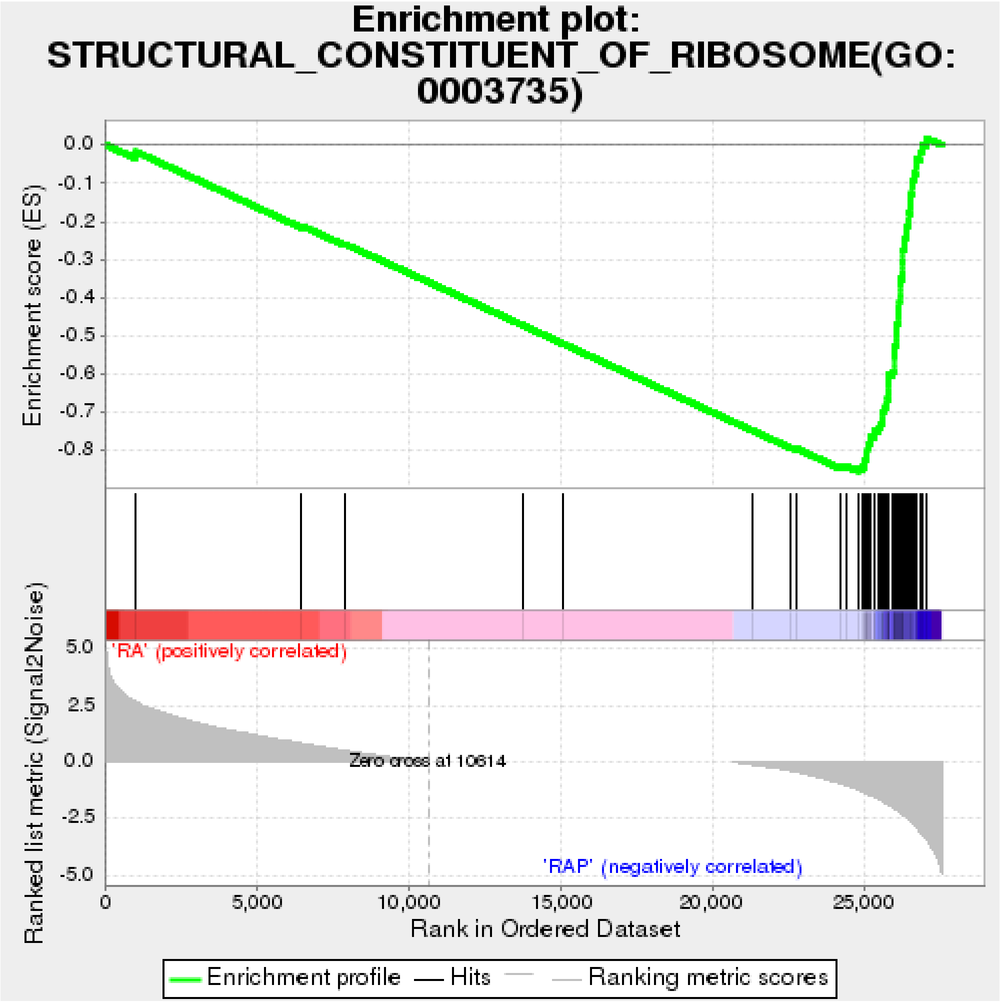

Fig 1: Enrichment plot: STRUCTURAL_CONSTITUENT_OF_RIBOSOME(GO:0003735)
Profile of the Running ES Score & Positions of GeneSet Members on the Rank Ordered List

Table: GSEA details [plain text format]

	PROBE	DESCRIPTION	GENE	GENE_TITLE	RANK IN	RANK	RUNNING	CORE

		(from dataset)	SYMBOL		GENE LIST	METRIC SCORE	ES	ENRICHMENT
1	PITG_19121	PITG_19121			962	2.666	-0.0165	No
2	PITG_03660	PITG_03660			6456	0.832	-0.2106	No
3	PITG_04337	PITG_04337			7866	0.519	-0.2582	No
4	PITG_15722	PITG_15722			13740	0.000	-0.4719	No
5	PITG_09431	PITG_09431			15054	0.000	-0.5197	No
6	PITG_05009	PITG_05009			21324	-0.134	-0.7468	No
7	PITG_10193	PITG_10193			22606	-0.404	-0.7906	No
8	PITG_11734	PITG_11734			22780	-0.455	-0.7938	No
9	PITG_19669	PITG_19669			24217	-0.958	-0.8394	No
10	PITG_16757	PITG_16757			24413	-1.036	-0.8393	No
11	PITG_19999	PITG_19999			24792	-1.214	-0.8446	Yes
12	PITG_20189	PITG_20189			24966	-1.317	-0.8418	Yes
13	PITG_01922	PITG_01922			25002	-1.336	-0.8338	Yes
14	PITG_12839	PITG_12839			25031	-1.352	-0.8254	Yes
15	PITG_14850	PITG_14850			25066	-1.380	-0.8171	Yes
16	PITG_11923	PITG_11923			25096	-1.398	-0.8084	Yes
17	PITG_16008	PITG_16008			25114	-1.410	-0.7993	Yes
18	PITG_06771	PITG_06771			25118	-1.413	-0.7896	Yes
19	PITG_15723	PITG_15723			25159	-1.438	-0.7811	Yes
20	PITG_00443	PITG_00443			25193	-1.456	-0.7722	Yes
21	PITG_04843	PITG_04843			25217	-1.471	-0.7628	Yes
22	PITG_05171	PITG_05171			25340	-1.548	-0.7565	Yes
23	PITG_12745	PITG_12745			25347	-1.553	-0.7460	Yes
24	PITG_20188	PITG_20188			25502	-1.654	-0.7401	Yes
25	PITG_12697	PITG_12697			25529	-1.673	-0.7294	Yes
26	PITG_10887	PITG_10887			25597	-1.713	-0.7200	Yes
27	PITG_07300	PITG_07300			25611	-1.727	-0.7085	Yes
28	PITG_03221	PITG_03221			25613	-1.729	-0.6965	Yes
29	PITG_15090	PITG_15090			25650	-1.757	-0.6856	Yes
30	PITG_08703	PITG_08703			25716	-1.807	-0.6755	Yes
31	PITG_02694	PITG_02694			25772	-1.849	-0.6647	Yes
32	PITG_15069	PITG_15069			25785	-1.859	-0.6522	Yes
33	PITG_01943	PITG_01943			25791	-1.864	-0.6394	Yes
34	PITG_15407	PITG_15407			25795	-1.868	-0.6266	Yes
35	PITG_11766	PITG_11766			25814	-1.881	-0.6142	Yes
36	PITG_01833	PITG_01833			25831	-1.897	-0.6016	Yes
37	PITG_20264	PITG_20264			25950	-1.992	-0.5921	Yes
38	PITG_07173	PITG_07173			25980	-2.022	-0.5791	Yes

39	PITG_19157	PITG_19157			25981	-2.022	-0.5651	Yes
40	PITG_10863	PITG_10863			25982	-2.022	-0.5511	Yes
41	PITG_09540	PITG_09540			25997	-2.035	-0.5375	Yes
42	PITG_03353	PITG_03353			26026	-2.059	-0.5242	Yes
43	PITG_09506	PITG_09506			26047	-2.074	-0.5106	Yes
44	PITG_00941	PITG_00941			26052	-2.078	-0.4963	Yes
45	PITG_14913	PITG_14913			26073	-2.104	-0.4824	Yes
46	PITG_04487	PITG_04487			26098	-2.129	-0.4685	Yes
47	PITG_03235	PITG_03235			26130	-2.165	-0.4546	Yes
48	PITG_19531	PITG_19531			26134	-2.169	-0.4397	Yes
49	PITG_04382	PITG_04382			26147	-2.181	-0.4250	Yes
50	PITG_06995	PITG_06995			26169	-2.197	-0.4105	Yes
51	PITG_08959	PITG_08959			26174	-2.203	-0.3954	Yes
52	PITG_05174	PITG_05174			26178	-2.205	-0.3802	Yes
53	PITG_03239	PITG_03239			26180	-2.206	-0.3650	Yes
54	PITG_03294	PITG_03294			26205	-2.241	-0.3503	Yes
55	PITG_09555	PITG_09555			26249	-2.286	-0.3360	Yes
56	Novel00015	Novel00015			26276	-2.309	-0.3209	Yes
57	PITG_17785	PITG_17785			26278	-2.310	-0.3049	Yes
58	PITG_12947	PITG_12947			26294	-2.326	-0.2894	Yes
59	PITG_18052	PITG_18052			26295	-2.327	-0.2732	Yes
60	PITG_13371	PITG_13371			26336	-2.376	-0.2582	Yes
61	PITG_06636	PITG_06636			26348	-2.389	-0.2420	Yes
62	PITG_03178	PITG_03178			26392	-2.437	-0.2267	Yes
63	PITG_00523	PITG_00523			26405	-2.460	-0.2101	Yes
64	PITG_09631	PITG_09631			26454	-2.515	-0.1944	Yes
65	PITG_13681	PITG_13681			26460	-2.519	-0.1771	Yes
66	PITG_10146	PITG_10146			26517	-2.576	-0.1612	Yes
67	PITG_02578	PITG_02578			26546	-2.608	-0.1442	Yes
68	PITG_06237	PITG_06237			26557	-2.626	-0.1263	Yes
69	PITG_03420	PITG_03420			26604	-2.687	-0.1094	Yes
70	PITG_01042	PITG_01042			26613	-2.704	-0.0909	Yes
71	PITG_09521	PITG_09521			26652	-2.752	-0.0732	Yes
72	PITG_09552	PITG_09552			26705	-2.829	-0.0555	Yes
73	PITG_14729	PITG_14729			26712	-2.839	-0.0360	Yes
74	PITG_03768	PITG_03768			26845	-3.062	-0.0195	Yes
75	PITG_18054	PITG_18054			26907	-3.165	0.0002	Yes
76	PITG_06596	PITG_06596			27055	-3.399	0.0184	Yes

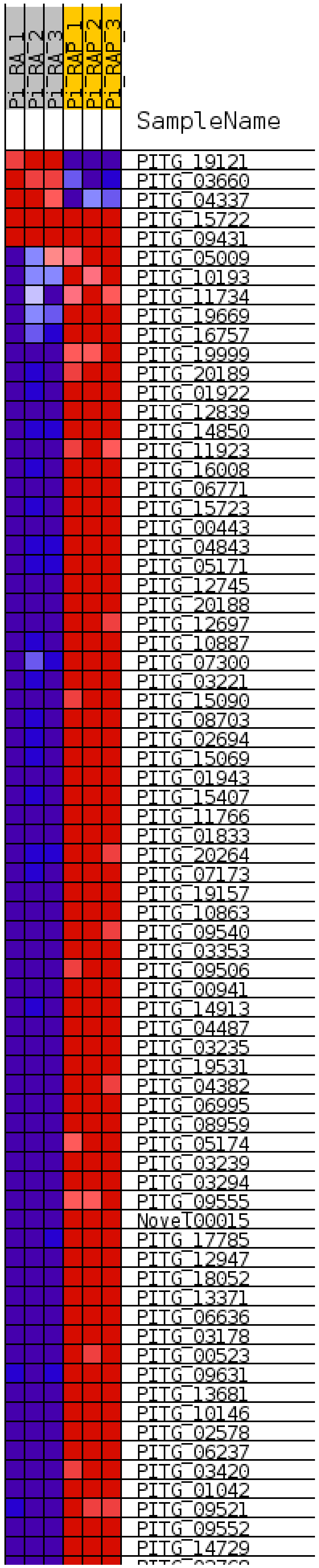
Pi_RA_1
Pi_RA_2
Pi_RA_3
Pi_RAP_1
Pi_RAP_2
Pi_RAP_3
SampleName
PITG_19121
PITG_03660
PITG_04337
PITG_15722
PITG_09431
PITG_05009
PITG_10193
PITG_11734
PITG_19669
PITG_16757
PITG_19999
PITG_20189
PITG_01922
PITG_12839
PITG_14850
PITG_11923
PITG_16008
PITG_06771
PITG_15723
PITG_00443
PITG_04843
PITG_05171
PITG_12745
PITG_20188
PITG_12697
PITG_10887
PITG_07300
PITG_03221
PITG_15090
PITG_08703
PITG_02694
PITG_15069
PITG_01943
PITG_15407
PITG_11766
PITG_01833
PITG_20264
PITG_07173
PITG_19157
PITG_10863
PITG_09540
PITG_03353
PITG_09506
PITG_00941
PITG_14913
PITG_04487
PITG_03235
PITG_19531
PITG_04382
PITG_06995
PITG_08959
PITG_05174
PITG_03239
PITG_03294
PITG_09555
Novel00015
PITG_17785
PITG_12947
PITG_18052
PITG_13371
PITG_06636
PITG_03178
PITG_00523
PITG_09631
PITG_13681
PITG_10146
PITG_02578
PITG_06237
PITG_03420
PITG_01042
PITG_09521
PITG_09552
PITG_14729

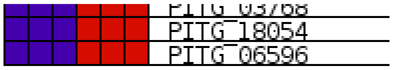

Fig 2: STRUCTURAL_CONSTITUENT_OF_RIBOSOME(GO:0003735)
Blue-Pink O' Gram in the Space of the Analyzed GeneSet

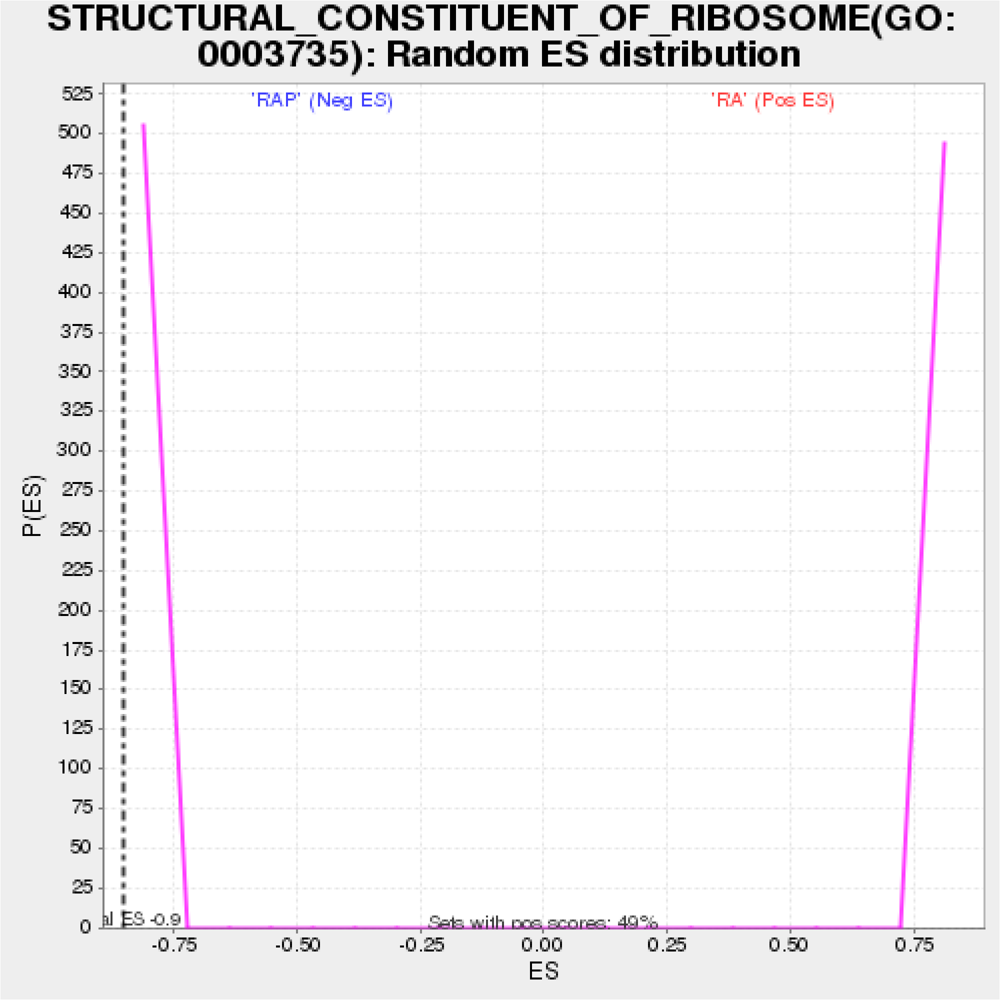

Fig 3: STRUCTURAL_CONSTITUENT_OF_RIBOSOME(GO:0003735): Random ES distribution
Gene set null distribution of ES for STRUCTURAL_CONSTITUENT_OF_RIBOSOME(GO:0003735)

10. Translation

Table: GSEA Results Summary

Dataset	fpkm.sample
Phenotype	sample.cls
Upregulated in class	RAP
GeneSet	TRANSLATION(GO:0006412)
Enrichment Score (ES)	-0.8083162
Normalized Enrichment Score (NES)	-1.0765922
Nominal p-value	0.0
FDR q-value	0.07712077
FWER p-Value	0.06

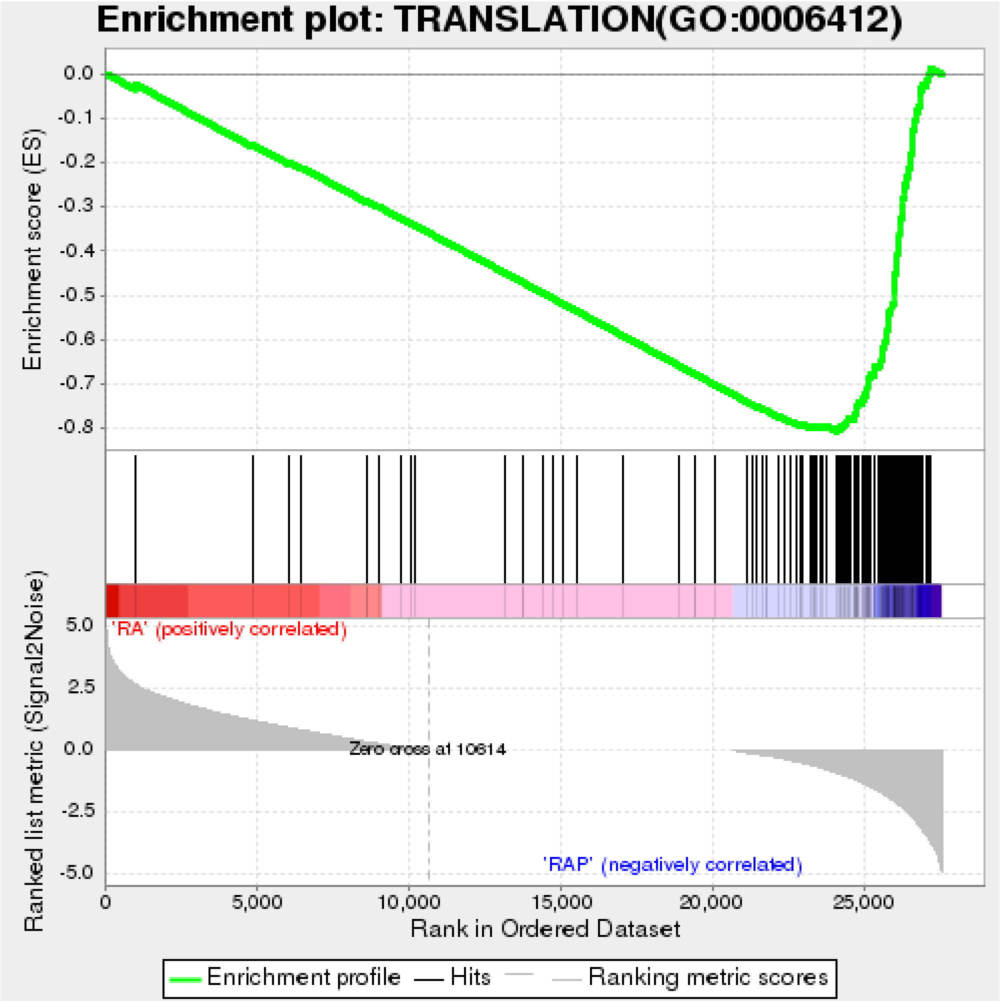

Fig 1: Enrichment plot: TRANSLATION(GO:0006412)
Profile of the Running ES Score & Positions of GeneSet Members on the Rank Ordered List

Table: GSEA details [plain text format]

	PROBE	DESCRIPTION	GENE	GENE_TITLE	RANK IN	RANK	RUNNING	CORE

		(from dataset)	SYMBOL		GENE LIST	METRIC SCORE	ES	ENRICHMENT
1	PITG_19121	PITG_19121			962	2.666	-0.0236	No
2	PITG_17651	PITG_17651			4826	1.224	-0.1593	No
3	PITG_07234	PITG_07234			6061	0.926	-0.2003	No
4	PITG_03660	PITG_03660			6456	0.832	-0.2111	No
5	PITG_11630	PITG_11630			8572	0.362	-0.2868	No
6	PITG_10516	PITG_10516			8635	0.351	-0.2875	No
7	PITG_14557	PITG_14557			8993	0.287	-0.2993	No
8	PITG_18303	PITG_18303			9750	0.151	-0.3262	No
9	PITG_09791	PITG_09791			10045	0.103	-0.3365	No
10	PITG_02992	PITG_02992			10209	0.072	-0.3421	No
11	PITG_20824	PITG_20824			13172	0.000	-0.4502	No
12	PITG_15722	PITG_15722			13740	0.000	-0.4709	No
13	PITG_22058	PITG_22058			13753	0.000	-0.4713	No
14	PITG_01091	PITG_01091			14376	0.000	-0.4940	No
15	PITG_05812	PITG_05812			14754	0.000	-0.5078	No
16	PITG_09431	PITG_09431			15054	0.000	-0.5187	No
17	PITG_06873	PITG_06873			15537	0.000	-0.5363	No
18	PITG_16530	PITG_16530			17026	0.000	-0.5906	No
19	PITG_20240	PITG_20240			18905	0.000	-0.6591	No
20	PITG_03806	PITG_03806			19383	0.000	-0.6765	No
21	PITG_03807	PITG_03807			19384	0.000	-0.6765	No
22	PITG_04594	PITG_04594			20065	0.000	-0.7013	No
23	PITG_02493	PITG_02493			21136	-0.100	-0.7399	No
24	PITG_05009	PITG_05009			21324	-0.134	-0.7462	No
25	PITG_22310	PITG_22310			21487	-0.165	-0.7514	No
26	PITG_21349	PITG_21349			21623	-0.186	-0.7555	No
27	PITG_12151	PITG_12151			21769	-0.212	-0.7599	No
28	PITG_13735	PITG_13735			21784	-0.214	-0.7595	No
29	PITG_05007	PITG_05007			22208	-0.303	-0.7736	No
30	PITG_03093	PITG_03093			22409	-0.359	-0.7793	No
31	PITG_10193	PITG_10193			22606	-0.404	-0.7848	No
32	PITG_11734	PITG_11734			22780	-0.455	-0.7891	No
33	PITG_16328	PITG_16328			22919	-0.491	-0.7920	No
34	PITG_22249	PITG_22249			22933	-0.496	-0.7904	No
35	PITG_09846	PITG_09846			22986	-0.514	-0.7901	No
36	PITG_12961	PITG_12961			23230	-0.590	-0.7964	No
37	PITG_16741	PITG_16741			23328	-0.615	-0.7973	No
38	PITG_08369	PITG_08369			23360	-0.629	-0.7957	No

39	PITG_04918	PITG_04918			23385	-0.637	-0.7938	No
40	PITG_03799	PITG_03799			23448	-0.655	-0.7933	No
41	PITG_05354	PITG_05354			23584	-0.710	-0.7952	No
42	PITG_02580	PITG_02580			23651	-0.734	-0.7944	No
43	PITG_17748	PITG_17748			23795	-0.780	-0.7963	No
44	PITG_21071	PITG_21071			24126	-0.913	-0.8044	Yes
45	PITG_14456	PITG_14456			24139	-0.918	-0.8009	Yes
46	PITG_19669	PITG_19669			24217	-0.958	-0.7996	Yes
47	PITG_06222	PITG_06222			24228	-0.961	-0.7958	Yes
48	PITG_11111	PITG_11111			24301	-0.988	-0.7942	Yes
49	PITG_17153	PITG_17153			24348	-1.006	-0.7915	Yes
50	PITG_01762	PITG_01762			24386	-1.026	-0.7885	Yes
51	PITG_16757	PITG_16757			24413	-1.036	-0.7850	Yes
52	PITG_18251	PITG_18251			24457	-1.057	-0.7820	Yes
53	PITG_03322	PITG_03322			24462	-1.057	-0.7776	Yes
54	PITG_10979	PITG_10979			24567	-1.100	-0.7766	Yes
55	PITG_07797	PITG_07797			24662	-1.147	-0.7751	Yes
56	PITG_07888	PITG_07888			24666	-1.148	-0.7703	Yes
57	PITG_03274	PITG_03274			24715	-1.175	-0.7670	Yes
58	PITG_14609	PITG_14609			24732	-1.182	-0.7625	Yes
59	PITG_05405	PITG_05405			24742	-1.189	-0.7577	Yes
60	PITG_07841	PITG_07841			24743	-1.190	-0.7526	Yes
61	PITG_04703	PITG_04703			24756	-1.196	-0.7479	Yes
62	PITG_19999	PITG_19999			24792	-1.214	-0.7439	Yes
63	PITG_12864	PITG_12864			24801	-1.219	-0.7390	Yes
64	PITG_03480	PITG_03480			24930	-1.293	-0.7381	Yes
65	PITG_20189	PITG_20189			24966	-1.317	-0.7337	Yes
66	PITG_01922	PITG_01922			25002	-1.336	-0.7292	Yes
67	PITG_12839	PITG_12839			25031	-1.352	-0.7244	Yes
68	PITG_14850	PITG_14850			25066	-1.380	-0.7197	Yes
69	PITG_11923	PITG_11923			25096	-1.398	-0.7148	Yes
70	PITG_16008	PITG_16008			25114	-1.410	-0.7093	Yes
71	PITG_06771	PITG_06771			25118	-1.413	-0.7034	Yes
72	PITG_15723	PITG_15723			25159	-1.438	-0.6986	Yes
73	PITG_07141	PITG_07141			25165	-1.441	-0.6926	Yes
74	PITG_04992	PITG_04992			25172	-1.445	-0.6866	Yes
75	PITG_00443	PITG_00443			25193	-1.456	-0.6811	Yes
76	PITG_04843	PITG_04843			25217	-1.471	-0.6756	Yes
77	PITG_05171	PITG_05171			25340	-1.548	-0.6734	Yes

78	PITG_12745	PITG_12745			25347	-1.553	-0.6669	Yes
79	PITG_10974	PITG_10974			25362	-1.559	-0.6607	Yes
80	PITG_20188	PITG_20188			25502	-1.654	-0.6587	Yes
81	PITG_12697	PITG_12697			25529	-1.673	-0.6524	Yes
82	PITG_04729	PITG_04729			25577	-1.699	-0.6468	Yes
83	PITG_09234	PITG_09234			25595	-1.710	-0.6401	Yes
84	PITG_10887	PITG_10887			25597	-1.713	-0.6328	Yes
85	PITG_07300	PITG_07300			25611	-1.727	-0.6258	Yes
86	PITG_03221	PITG_03221			25613	-1.729	-0.6184	Yes
87	PITG_15090	PITG_15090			25650	-1.757	-0.6122	Yes
88	PITG_04774	PITG_04774			25653	-1.761	-0.6047	Yes
89	PITG_08703	PITG_08703			25716	-1.807	-0.5992	Yes
90	PITG_01255	PITG_01255			25748	-1.835	-0.5924	Yes
91	PITG_10110	PITG_10110			25769	-1.847	-0.5852	Yes
92	PITG_02694	PITG_02694			25772	-1.849	-0.5773	Yes
93	PITG_15069	PITG_15069			25785	-1.859	-0.5697	Yes
94	PITG_01943	PITG_01943			25791	-1.864	-0.5619	Yes
95	PITG_15407	PITG_15407			25795	-1.868	-0.5540	Yes
96	PITG_11766	PITG_11766			25814	-1.881	-0.5465	Yes
97	PITG_01833	PITG_01833			25831	-1.897	-0.5390	Yes
98	PITG_02921	PITG_02921			25864	-1.929	-0.5318	Yes
99	PITG_08579	PITG_08579			25907	-1.965	-0.5249	Yes
100	PITG_20264	PITG_20264			25950	-1.992	-0.5179	Yes
101	PITG_07173	PITG_07173			25980	-2.022	-0.5102	Yes
102	PITG_19157	PITG_19157			25981	-2.022	-0.5015	Yes
103	PITG_10863	PITG_10863			25982	-2.022	-0.4928	Yes
104	PITG_03460	PITG_03460			25987	-2.027	-0.4843	Yes
105	PITG_09540	PITG_09540			25997	-2.035	-0.4758	Yes
106	PITG_03661	PITG_03661			26015	-2.048	-0.4677	Yes
107	PITG_03353	PITG_03353			26026	-2.059	-0.4592	Yes
108	PITG_13831	PITG_13831			26029	-2.061	-0.4504	Yes
109	PITG_09506	PITG_09506			26047	-2.074	-0.4421	Yes
110	PITG_00941	PITG_00941			26052	-2.078	-0.4333	Yes
111	PITG_14913	PITG_14913			26073	-2.104	-0.4249	Yes
112	PITG_04487	PITG_04487			26098	-2.129	-0.4167	Yes
113	PITG_00302	PITG_00302			26103	-2.131	-0.4076	Yes
114	PITG_04747	PITG_04747			26111	-2.138	-0.3987	Yes
115	PITG_03235	PITG_03235			26130	-2.165	-0.3900	Yes
116	PITG_19531	PITG_19531			26134	-2.169	-0.3808	Yes

117	PITG_04382	PITG_04382			26147	-2.181	-0.3719	Yes
118	PITG_06995	PITG_06995			26169	-2.197	-0.3632	Yes
119	PITG_08959	PITG_08959			26174	-2.203	-0.3539	Yes
120	PITG_05174	PITG_05174			26178	-2.205	-0.3445	Yes
121	PITG_03239	PITG_03239			26180	-2.206	-0.3350	Yes
122	PITG_03294	PITG_03294			26205	-2.241	-0.3263	Yes
123	PITG_09555	PITG_09555			26249	-2.286	-0.3180	Yes
124	Novel00015	Novel00015			26276	-2.309	-0.3090	Yes
125	PITG_17785	PITG_17785			26278	-2.310	-0.2991	Yes
126	PITG_12947	PITG_12947			26294	-2.326	-0.2897	Yes
127	PITG_18052	PITG_18052			26295	-2.327	-0.2796	Yes
128	PITG_12077	PITG_12077			26335	-2.375	-0.2709	Yes
129	PITG_13371	PITG_13371			26336	-2.376	-0.2606	Yes
130	PITG_06636	PITG_06636			26348	-2.389	-0.2508	Yes
131	PITG_03178	PITG_03178			26392	-2.437	-0.2418	Yes
132	PITG_00523	PITG_00523			26405	-2.460	-0.2317	Yes
133	PITG_09631	PITG_09631			26454	-2.515	-0.2226	Yes
134	PITG_13681	PITG_13681			26460	-2.519	-0.2120	Yes
135	PITG_10146	PITG_10146			26517	-2.576	-0.2029	Yes
136	PITG_02578	PITG_02578			26546	-2.608	-0.1927	Yes
137	PITG_06237	PITG_06237			26557	-2.626	-0.1818	Yes
138	PITG_00910	PITG_00910			26570	-2.639	-0.1709	Yes
139	PITG_17607	PITG_17607			26591	-2.668	-0.1601	Yes
140	PITG_03420	PITG_03420			26604	-2.687	-0.1490	Yes
141	PITG_01042	PITG_01042			26613	-2.704	-0.1377	Yes
142	PITG_03999	PITG_03999			26623	-2.720	-0.1263	Yes
143	PITG_09521	PITG_09521			26652	-2.752	-0.1155	Yes
144	PITG_02039	PITG_02039			26671	-2.781	-0.1042	Yes
145	PITG_09552	PITG_09552			26705	-2.829	-0.0932	Yes
146	PITG_14729	PITG_14729			26712	-2.839	-0.0812	Yes
147	PITG_00397	PITG_00397			26815	-3.015	-0.0720	Yes
148	PITG_03768	PITG_03768			26845	-3.062	-0.0599	Yes
149	PITG_06821	PITG_06821			26849	-3.067	-0.0468	Yes
150	PITG_08714	PITG_08714			26862	-3.088	-0.0339	Yes
151	PITG_18054	PITG_18054			26907	-3.165	-0.0219	Yes
152	PITG_06596	PITG_06596			27055	-3.399	-0.0127	Yes
153	PITG_21661	PITG_21661			27155	-3.586	-0.0008	Yes
154	PITG_22020	PITG_22020			27176	-3.631	0.0140	Yes

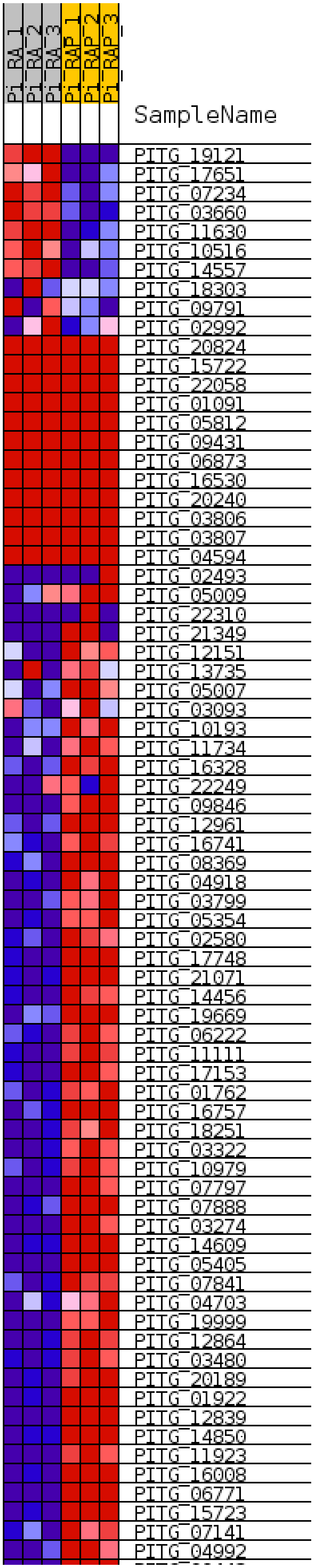
Pi_RA_1
Pi_RA_2
Pi_RA_3
Pi_RAP_1
Pi_RAP_2
Pi_RAP_3
SampleName
PITG_19121
PITG_17651
PITG_07234
PITG_03660
PITG_11630
PITG_10516
PITG_14557
PITG_18303
PITG_09791
PITG_02992
PITG_20824
PITG_15722
PITG_22058
PITG_01091
PITG_05812
PITG_09431
PITG_06873
PITG_16530
PITG_20240
PITG_03806
PITG_03807
PITG_04594
PITG_02493
PITG_05009
PITG_22310
PITG_21349
PITG_12151
PITG_13735
PITG_05007
PITG_03093
PITG_10193
PITG_11734
PITG_16328
PITG_22249
PITG_09846
PITG_12961
PITG_16741
PITG_08369
PITG_04918
PITG_03799
PITG_05354
PITG_02580
PITG_17748
PITG_21071
PITG_14456
PITG_19669
PITG_06222
PITG_11111
PITG_17153
PITG_01762
PITG_16757
PITG_18251
PITG_03322
PITG_10979
PITG_07797
PITG_07888
PITG_03274
PITG_14609
PITG_05405
PITG_07841
PITG_04703
PITG_19999
PITG_12864
PITG_03480
PITG_20189
PITG_01922
PITG_12839
PITG_14850
PITG_11923
PITG_16008
PITG_06771
PITG_15723
PITG_07141
PITG_04992

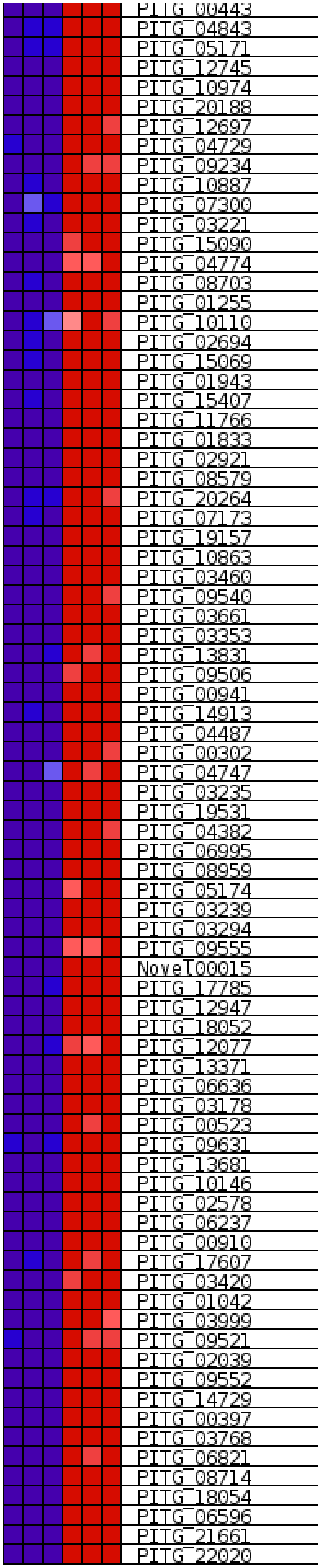
PITG_00443
PITG_04843
PITG_05171
PITG_12745
PITG_10974
PITG_20188
PITG_12697
PITG_04729
PITG_09234
PITG_10887
PITG_07300
PITG_03221
PITG_15090
PITG_04774
PITG_08703
PITG_01255
PITG_10110
PITG_02694
PITG_15069
PITG_01943
PITG_15407
PITG_11766
PITG_01833
PITG_02921
PITG_08579
PITG_20264
PITG_07173
PITG_19157
PITG_10863
PITG_03460
PITG_09540
PITG_03661
PITG_03353
PITG_13831
PITG_09506
PITG_00941
PITG_14913
PITG_04487
PITG_00302
PITG_04747
PITG_03235
PITG_19531
PITG_04382
PITG_06995
PITG_08959
PITG_05174
PITG_03239
PITG_03294
PITG_09555
Novel00015
PITG_17785
PITG_12947
PITG_18052
PITG_12077
PITG_13371
PITG_06636
PITG_03178
PITG_00523
PITG_09631
PITG_13681
PITG_10146
PITG_02578
PITG_06237
PITG_00910
PITG_17607
PITG_03420
PITG_01042
PITG_03999
PITG_09521
PITG_02039
PITG_09552
PITG_14729
PITG_00397
PITG_03768
PITG_06821
PITG_08714
PITG_18054
PITG_06596
PITG_21661
PITG_22020

Fig 2: TRANSLATION(GO:0006412)
Blue-Pink O' Gram in the Space of the Analyzed GeneSet

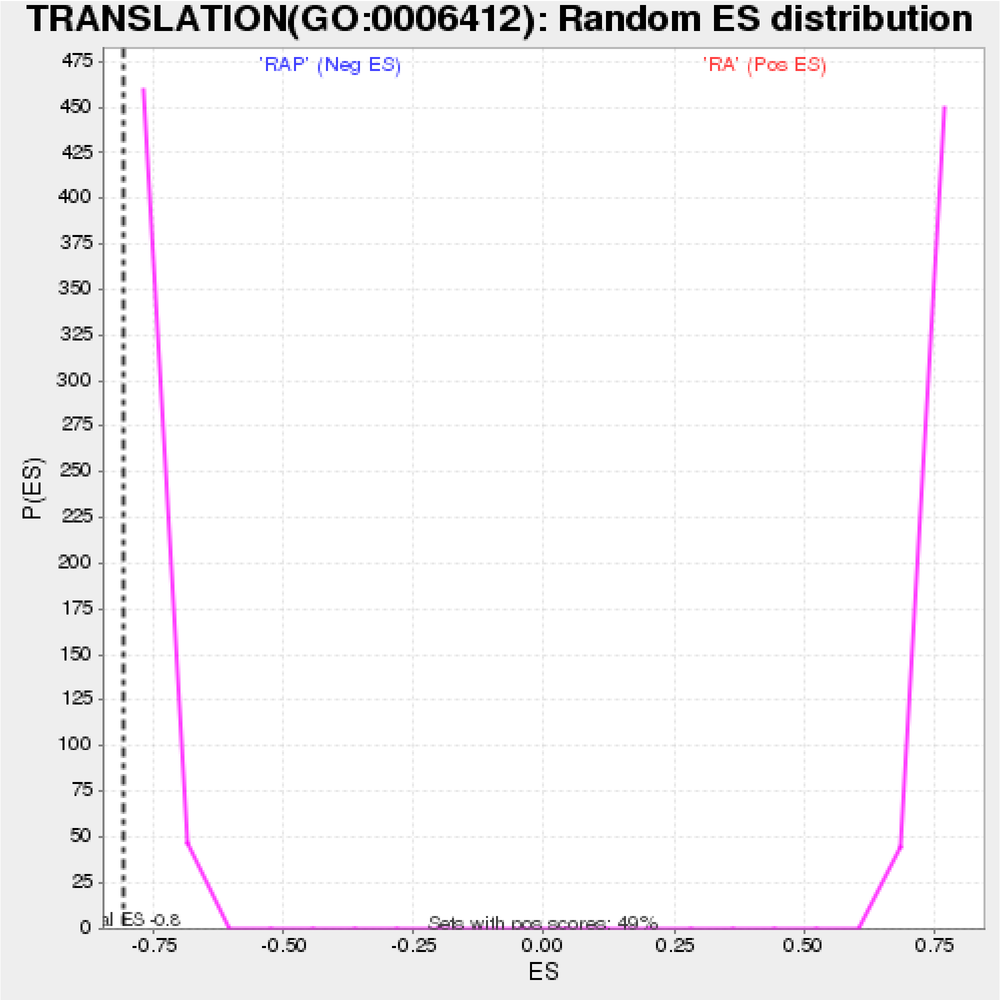

Fig 3: TRANSLATION(GO:0006412): Random ES distribution
Gene set null distribution of ES for TRANSLATION(GO:0006412)
