# Supplementary material for: TOR Inhibitors Synergistically Suppress the Growth and Development of Phytophthora infestans, a Highly Destructive Pathogenic Oomycete
Source: Front Microbiol. 2021 Apr 16;12:596874. doi: 10.3389/fmicb.2021.596874 (PMC8086431; doi:10.3389/fmicb.2021.596874)
Supplement: Supplementary Data Sheets 1, 2 — The detailed GSEA analyses of GO terms (RAP + AZD vs. RAP, RAP + AZD vs. AZD). 1. Cellular amide metabolic process; 2. Organonitrogen compound biosynthetic process; 3. Organonitrogen compound metabolic process; 4. Peptide biosynthetic process; 5. Peptide metabolic process; 6. Ribonucleoprotein complex; 7. Ribosome; 8. RNA binding; 9. Structural constituent of ribosome; 10. Translation. [file Data_Sheet_2.PDF]

1. cellular amide metabolic process

Table: GSEA Results Summary

Dataset	fpkm.sample
Phenotype	sample.cls
Upregulated in class	AZD
GeneSet	CELLULAR_AMIDE_METABOLIC_PROCESS(GO:0043603)
Enrichment Score (ES)	-0.6774449
Normalized Enrichment Score (NES)	-1.0728428
Nominal p-value	0.0
FDR q-value	0.12050168
FWER p-Value	0.106

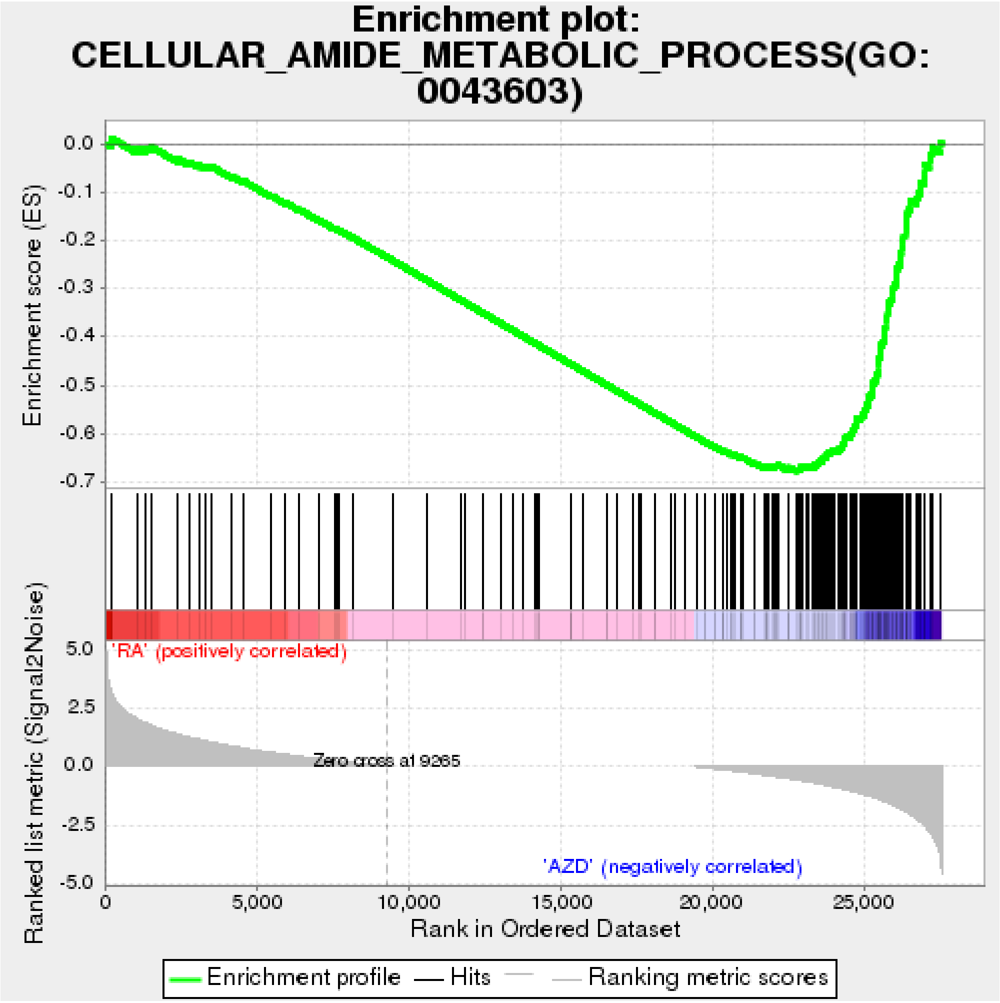

Fig 1: Enrichment plot: CELLULAR_AMIDE_METABOLIC_PROCESS(GO:0043603)
Profile of the Running ES Score & Positions of GeneSet Members on the Rank Ordered List

Table: GSEA details [plain text format]

	PROBE	DESCRIPTION	GENE	GENE_TITLE	RANK IN	RANK	RUNNING	CORE

		(from dataset)	SYMBOL		GENE LIST	METRIC SCORE	ES	ENRICHMENT
1	PITG_10138	PITG_10138			159	3.220	0.0112	No
2	PITG_19121	PITG_19121			1014	2.048	-0.0092	No
3	PITG_04708	PITG_04708			1272	1.888	-0.0086	No
4	PITG_00636	PITG_00636			1466	1.768	-0.0063	No
5	PITG_00543	PITG_00543			2335	1.398	-0.0306	No
6	PITG_11630	PITG_11630			2711	1.279	-0.0376	No
7	Novel00922	Novel00922			3038	1.181	-0.0432	No
8	PITG_03660	PITG_03660			3265	1.116	-0.0456	No
9	PITG_00177	PITG_00177			3445	1.064	-0.0465	No
10	PITG_15417	PITG_15417			4137	0.885	-0.0671	No
11	PITG_16088	PITG_16088			4491	0.802	-0.0758	No
12	PITG_07234	PITG_07234			5449	0.602	-0.1075	No
13	PITG_17651	PITG_17651			5906	0.516	-0.1215	No
14	PITG_22572	PITG_22572			6371	0.437	-0.1361	No
15	PITG_05009	PITG_05009			7048	0.319	-0.1591	No
16	PITG_02992	PITG_02992			7548	0.243	-0.1761	No
17	PITG_03093	PITG_03093			7622	0.232	-0.1775	No
18	PITG_10516	PITG_10516			7711	0.219	-0.1796	No
19	PITG_17153	PITG_17153			8119	0.169	-0.1936	No
20	PITG_20640	PITG_20640			9457	0.000	-0.2424	No
21	PITG_22310	PITG_22310			10562	0.000	-0.2828	No
22	PITG_21582	PITG_21582			11683	0.000	-0.3237	No
23	PITG_20824	PITG_20824			11824	0.000	-0.3288	No
24	PITG_15722	PITG_15722			12398	0.000	-0.3498	No
25	PITG_22058	PITG_22058			12412	0.000	-0.3502	No
26	PITG_01091	PITG_01091			13039	0.000	-0.3731	No
27	PITG_05812	PITG_05812			13417	0.000	-0.3869	No
28	PITG_09431	PITG_09431			13717	0.000	-0.3978	No
29	PITG_17187	PITG_17187			14156	0.000	-0.4138	No
30	PITG_06873	PITG_06873			14206	0.000	-0.4156	No
31	PITG_19379	PITG_19379			14298	0.000	-0.4189	No
32	PITG_19374	PITG_19374			14299	0.000	-0.4189	No
33	PITG_14310	PITG_14310			15340	0.000	-0.4569	No
34	PITG_14312	PITG_14312			15341	0.000	-0.4569	No
35	PITG_14315	PITG_14315			15343	0.000	-0.4570	No
36	PITG_16530	PITG_16530			15706	0.000	-0.4702	No
37	PITG_14346	PITG_14346			16494	0.000	-0.4989	No
38	PITG_14344	PITG_14344			16495	0.000	-0.4989	No

39	PITG_18225	PITG_18225			16859	0.000	-0.5122	No
40	PITG_21979	PITG_21979			17341	0.000	-0.5298	No
41	PITG_14322	PITG_14322			17578	0.000	-0.5384	No
42	PITG_20240	PITG_20240			17604	0.000	-0.5393	No
43	PITG_03806	PITG_03806			18089	0.000	-0.5570	No
44	PITG_03807	PITG_03807			18090	0.000	-0.5570	No
45	PITG_18553	PITG_18553			18597	0.000	-0.5755	No
46	PITG_04594	PITG_04594			18776	0.000	-0.5820	No
47	PITG_14325	PITG_14325			19076	0.000	-0.5929	No
48	PITG_10193	PITG_10193			19492	-0.015	-0.6080	No
49	PITG_18303	PITG_18303			19751	-0.048	-0.6172	No
50	PITG_09791	PITG_09791			19763	-0.050	-0.6173	No
51	PITG_03480	PITG_03480			20045	-0.095	-0.6271	No
52	PITG_21349	PITG_21349			20105	-0.104	-0.6287	No
53	PITG_09846	PITG_09846			20357	-0.142	-0.6371	No
54	PITG_01255	PITG_01255			20360	-0.142	-0.6364	No
55	PITG_14557	PITG_14557			20366	-0.143	-0.6359	No
56	PITG_04747	PITG_04747			20459	-0.156	-0.6384	No
57	PITG_16741	PITG_16741			20622	-0.179	-0.6434	No
58	PITG_03681	PITG_03681			20625	-0.180	-0.6425	No
59	PITG_00757	PITG_00757			20661	-0.186	-0.6428	No
60	PITG_04729	PITG_04729			20710	-0.194	-0.6435	No
61	PITG_02493	PITG_02493			20900	-0.221	-0.6493	No
62	PITG_01580	PITG_01580			20920	-0.225	-0.6488	No
63	PITG_04774	PITG_04774			20937	-0.229	-0.6481	No
64	PITG_05730	PITG_05730			21018	-0.243	-0.6498	No
65	PITG_12077	PITG_12077			21406	-0.311	-0.6623	No
66	PITG_11734	PITG_11734			21411	-0.312	-0.6608	No
67	PITG_12151	PITG_12151			21701	-0.361	-0.6694	No
68	PITG_16328	PITG_16328			21756	-0.372	-0.6694	No
69	PITG_10519	PITG_10519			21779	-0.377	-0.6683	No
70	PITG_14850	PITG_14850			21815	-0.384	-0.6675	No
71	PITG_16757	PITG_16757			21821	-0.385	-0.6657	No
72	PITG_07797	PITG_07797			21968	-0.412	-0.6688	No
73	PITG_03799	PITG_03799			22019	-0.421	-0.6684	No
74	PITG_05405	PITG_05405			22072	-0.429	-0.6680	No
75	PITG_05733	PITG_05733			22091	-0.432	-0.6664	No
76	PITG_08669	PITG_08669			22119	-0.437	-0.6651	No
77	PITG_20188	PITG_20188			22157	-0.445	-0.6641	No

78	PITG_05007	PITG_05007			22498	-0.509	-0.6738	No
79	PITG_19669	PITG_19669			22503	-0.510	-0.6713	No
80	PITG_12839	PITG_12839			22518	-0.514	-0.6691	No
81	PITG_06222	PITG_06222			22748	-0.566	-0.6745	Yes
82	PITG_10979	PITG_10979			22822	-0.583	-0.6740	Yes
83	PITG_19999	PITG_19999			22878	-0.594	-0.6729	Yes
84	PITG_04918	PITG_04918			22927	-0.602	-0.6715	Yes
85	PITG_00443	PITG_00443			22936	-0.604	-0.6686	Yes
86	PITG_05354	PITG_05354			22976	-0.613	-0.6668	Yes
87	PITG_02921	PITG_02921			23131	-0.650	-0.6690	Yes
88	PITG_12961	PITG_12961			23203	-0.663	-0.6681	Yes
89	PITG_12745	PITG_12745			23280	-0.681	-0.6672	Yes
90	PITG_14609	PITG_14609			23351	-0.695	-0.6661	Yes
91	PITG_02580	PITG_02580			23358	-0.695	-0.6627	Yes
92	PITG_11111	PITG_11111			23461	-0.720	-0.6626	Yes
93	PITG_15090	PITG_15090			23482	-0.725	-0.6595	Yes
94	PITG_04843	PITG_04843			23523	-0.735	-0.6571	Yes
95	PITG_16008	PITG_16008			23545	-0.740	-0.6539	Yes
96	PITG_17748	PITG_17748			23641	-0.765	-0.6534	Yes
97	PITG_13735	PITG_13735			23644	-0.766	-0.6494	Yes
98	PITG_06771	PITG_06771			23729	-0.789	-0.6483	Yes
99	PITG_08369	PITG_08369			23772	-0.802	-0.6456	Yes
100	PITG_10887	PITG_10887			23788	-0.807	-0.6419	Yes
101	PITG_20189	PITG_20189			23835	-0.820	-0.6392	Yes
102	PITG_03420	PITG_03420			23870	-0.829	-0.6361	Yes
103	PITG_15723	PITG_15723			23962	-0.858	-0.6349	Yes
104	PITG_01922	PITG_01922			24019	-0.874	-0.6323	Yes
105	PITG_10974	PITG_10974			24137	-0.910	-0.6318	Yes
106	PITG_12864	PITG_12864			24223	-0.936	-0.6299	Yes
107	PITG_04703	PITG_04703			24269	-0.949	-0.6265	Yes
108	PITG_21071	PITG_21071			24300	-0.958	-0.6226	Yes
109	PITG_02694	PITG_02694			24328	-0.970	-0.6184	Yes
110	PITG_04487	PITG_04487			24365	-0.980	-0.6146	Yes
111	PITG_01943	PITG_01943			24378	-0.982	-0.6098	Yes
112	PITG_11923	PITG_11923			24405	-0.990	-0.6056	Yes
113	PITG_03274	PITG_03274			24545	-1.036	-0.6052	Yes
114	PITG_03221	PITG_03221			24557	-1.040	-0.6001	Yes
115	PITG_10139	PITG_10139			24603	-1.053	-0.5962	Yes
116	PITG_11766	PITG_11766			24642	-1.064	-0.5919	Yes

117	PITG_07841	PITG_07841			24668	-1.073	-0.5872	Yes
118	PITG_00754	PITG_00754			24709	-1.085	-0.5829	Yes
119	PITG_03322	PITG_03322			24740	-1.096	-0.5782	Yes
120	PITG_06995	PITG_06995			24760	-1.104	-0.5731	Yes
121	PITG_01833	PITG_01833			24767	-1.106	-0.5674	Yes
122	PITG_01762	PITG_01762			24911	-1.153	-0.5666	Yes
123	PITG_05171	PITG_05171			24921	-1.158	-0.5608	Yes
124	PITG_22249	PITG_22249			25013	-1.195	-0.5578	Yes
125	PITG_15069	PITG_15069			25019	-1.198	-0.5516	Yes
126	PITG_00941	PITG_00941			25067	-1.214	-0.5469	Yes
127	PITG_07888	PITG_07888			25072	-1.216	-0.5407	Yes
128	PITG_18251	PITG_18251			25141	-1.242	-0.5366	Yes
129	PITG_12697	PITG_12697			25164	-1.249	-0.5308	Yes
130	PITG_13371	PITG_13371			25168	-1.249	-0.5243	Yes
131	PITG_15407	PITG_15407			25203	-1.263	-0.5189	Yes
132	PITG_03999	PITG_03999			25257	-1.283	-0.5140	Yes
133	PITG_05174	PITG_05174			25267	-1.288	-0.5075	Yes
134	PITG_03294	PITG_03294			25303	-1.306	-0.5019	Yes
135	PITG_08703	PITG_08703			25308	-1.308	-0.4952	Yes
136	PITG_07173	PITG_07173			25342	-1.325	-0.4894	Yes
137	PITG_14913	PITG_14913			25405	-1.356	-0.4845	Yes
138	PITG_18052	PITG_18052			25424	-1.365	-0.4779	Yes
139	PITG_08959	PITG_08959			25458	-1.380	-0.4718	Yes
140	PITG_03235	PITG_03235			25480	-1.389	-0.4652	Yes
141	PITG_08206	PITG_08206			25481	-1.390	-0.4579	Yes
142	PITG_03661	PITG_03661			25487	-1.391	-0.4507	Yes
143	PITG_04382	PITG_04382			25503	-1.398	-0.4439	Yes
144	PITG_14729	PITG_14729			25528	-1.410	-0.4373	Yes
145	PITG_07141	PITG_07141			25545	-1.420	-0.4304	Yes
146	PITG_09506	PITG_09506			25563	-1.431	-0.4235	Yes
147	PITG_08579	PITG_08579			25572	-1.438	-0.4161	Yes
148	PITG_19399	PITG_19399			25621	-1.462	-0.4102	Yes
149	PITG_04992	PITG_04992			25670	-1.484	-0.4041	Yes
150	PITG_10146	PITG_10146			25685	-1.491	-0.3967	Yes
151	PITG_09540	PITG_09540			25692	-1.493	-0.3891	Yes
152	PITG_00523	PITG_00523			25696	-1.497	-0.3813	Yes
153	PITG_09234	PITG_09234			25712	-1.502	-0.3739	Yes
154	PITG_10863	PITG_10863			25713	-1.502	-0.3659	Yes
155	PITG_06237	PITG_06237			25757	-1.523	-0.3595	Yes

156	PITG_17785	PITG_17785			25774	-1.532	-0.3519	Yes
157	PITG_03353	PITG_03353			25780	-1.535	-0.3440	Yes
158	PITG_07300	PITG_07300			25805	-1.550	-0.3367	Yes
159	PITG_20264	PITG_20264			25831	-1.561	-0.3294	Yes
160	PITG_00302	PITG_00302			25872	-1.579	-0.3225	Yes
161	Novel00015	Novel00015			25949	-1.627	-0.3167	Yes
162	PITG_14456	PITG_14456			25952	-1.628	-0.3081	Yes
163	PITG_04683	PITG_04683			25968	-1.637	-0.3000	Yes
164	PITG_12947	PITG_12947			26003	-1.658	-0.2925	Yes
165	PITG_03239	PITG_03239			26044	-1.683	-0.2851	Yes
166	PITG_02578	PITG_02578			26070	-1.705	-0.2770	Yes
167	PITG_19531	PITG_19531			26082	-1.713	-0.2683	Yes
168	PITG_03460	PITG_03460			26094	-1.719	-0.2597	Yes
169	PITG_03178	PITG_03178			26113	-1.732	-0.2512	Yes
170	PITG_13831	PITG_13831			26183	-1.780	-0.2443	Yes
171	PITG_08129	PITG_08129			26202	-1.791	-0.2355	Yes
172	PITG_00910	PITG_00910			26229	-1.807	-0.2269	Yes
173	PITG_13681	PITG_13681			26238	-1.816	-0.2176	Yes
174	PITG_06821	PITG_06821			26267	-1.833	-0.2089	Yes
175	PITG_09552	PITG_09552			26270	-1.835	-0.1993	Yes
176	PITG_19157	PITG_19157			26301	-1.850	-0.1906	Yes
177	PITG_06636	PITG_06636			26370	-1.898	-0.1831	Yes
178	PITG_09631	PITG_09631			26373	-1.899	-0.1731	Yes
179	PITG_01042	PITG_01042			26385	-1.915	-0.1634	Yes
180	PITG_00397	PITG_00397			26402	-1.929	-0.1538	Yes
181	PITG_09555	PITG_09555			26429	-1.950	-0.1444	Yes
182	PITG_13399	PITG_13399			26492	-2.007	-0.1361	Yes
183	PITG_09521	PITG_09521			26522	-2.027	-0.1264	Yes
184	PITG_07991	PITG_07991			26527	-2.030	-0.1159	Yes
185	PITG_02039	PITG_02039			26754	-2.231	-0.1123	Yes
186	PITG_03768	PITG_03768			26780	-2.261	-0.1013	Yes
187	PITG_06596	PITG_06596			26861	-2.351	-0.0918	Yes
188	PITG_18054	PITG_18054			26868	-2.362	-0.0795	Yes
189	PITG_17607	PITG_17607			26969	-2.477	-0.0701	Yes
190	PITG_18545	PITG_18545			27014	-2.541	-0.0583	Yes
191	PITG_22020	PITG_22020			27018	-2.545	-0.0449	Yes
192	PITG_21661	PITG_21661			27161	-2.778	-0.0354	Yes
193	PITG_08714	PITG_08714			27196	-2.846	-0.0216	Yes
194	PITG_10110	PITG_10110			27232	-2.902	-0.0076	Yes

195	PITG_14352	PITG_14352			27505	-3.705	0.0020	Yes

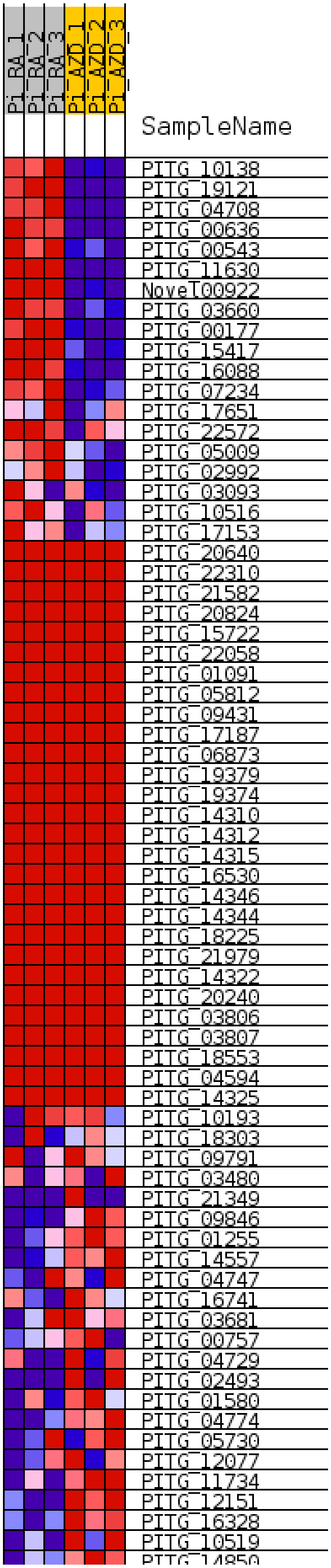

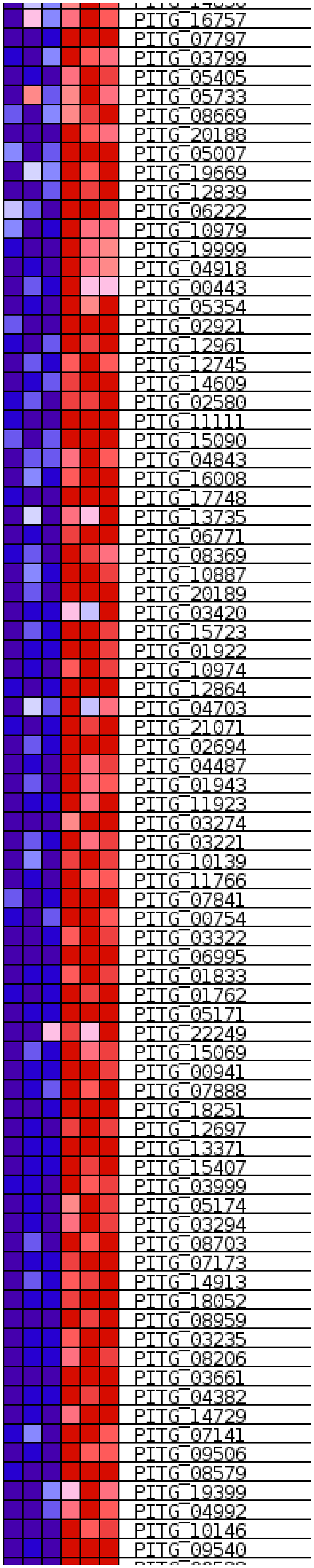
PITG_16757
PITG_07797
PITG_03799
PITG_05405
PITG_05733
PITG_08669
PITG_20188
PITG_05007
PITG_19669
PITG_12839
PITG_06222
PITG_10979
PITG_19999
PITG_04918
PITG_00443
PITG_05354
PITG_02921
PITG_12961
PITG_12745
PITG_14609
PITG_02580
PITG_11111
PITG_15090
PITG_04843
PITG_16008
PITG_17748
PITG_13735
PITG_06771
PITG_08369
PITG_10887
PITG_20189
PITG_03420
PITG_15723
PITG_01922
PITG_10974
PITG_12864
PITG_04703
PITG_21071
PITG_02694
PITG_04487
PITG_01943
PITG_11923
PITG_03274
PITG_03221
PITG_10139
PITG_11766
PITG_07841
PITG_00754
PITG_03322
PITG_06995
PITG_01833
PITG_01762
PITG_05171
PITG_22249
PITG_15069
PITG_00941
PITG_07888
PITG_18251
PITG_12697
PITG_13371
PITG_15407
PITG_03999
PITG_05174
PITG_03294
PITG_08703
PITG_07173
PITG_14913
PITG_18052
PITG_08959
PITG_03235
PITG_08206
PITG_03661
PITG_04382
PITG_14729
PITG_07141
PITG_09506
PITG_08579
PITG_19399
PITG_04992
PITG_10146
PITG_09540

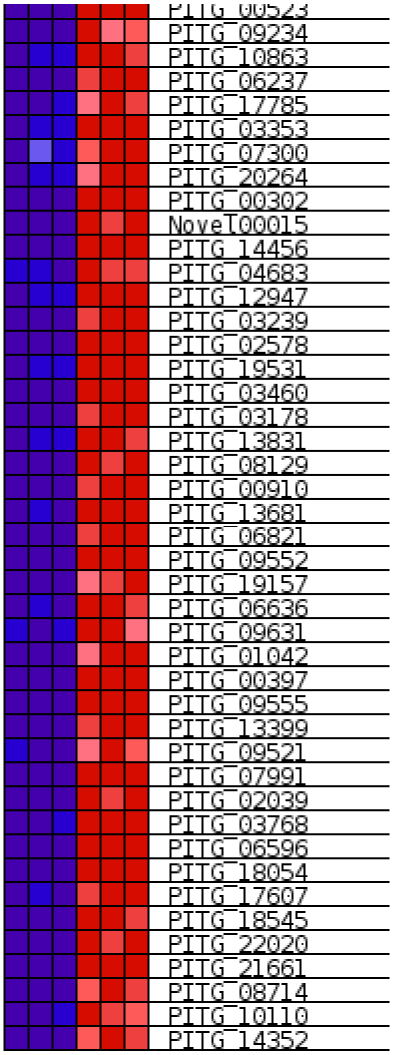

Fig 2: CELLULAR_AMIDE_METABOLIC_PROCESS(GO:0043603)
Blue-Pink O' Gram in the Space of the Analyzed GeneSet

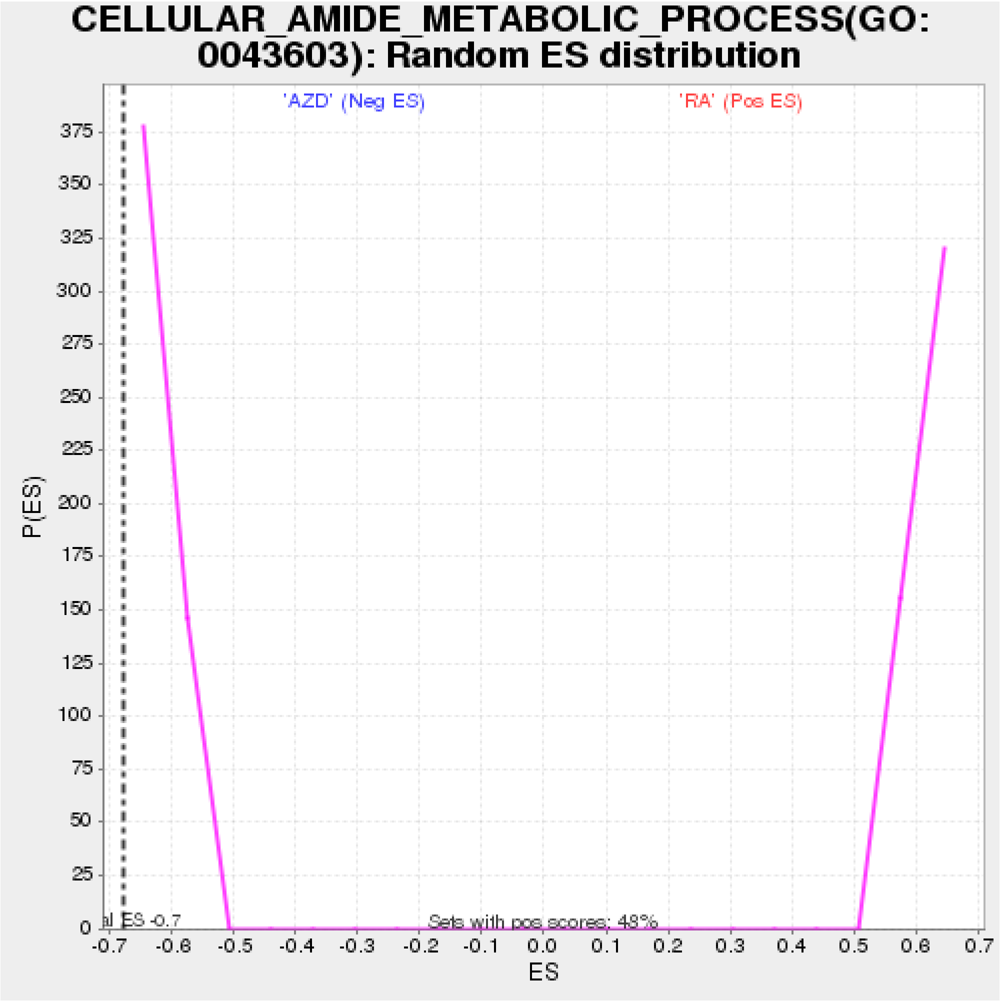

Fig 3: CELLULAR_AMIDE_METABOLIC_PROCESS(GO:0043603): Random ES distribution
Gene set null distribution of ES for CELLULAR_AMIDE_METABOLIC_PROCESS(GO:0043603)

2. organonitrogen compound biosynthetic process

Table: GSEA Results Summary

Dataset	fpkm.sample
Phenotype	sample.cls
Upregulated in class	AZD
GeneSet	ORGANONITROGEN_COMPOUND_BIOSYNTHETIC_PROCESS(GO:1901566)
Enrichment Score (ES)	-0.57017547
Normalized Enrichment Score (NES)	-1.120637
Nominal p-value	0.0
FDR q-value	0.10687026
FWER p-Value	0.056

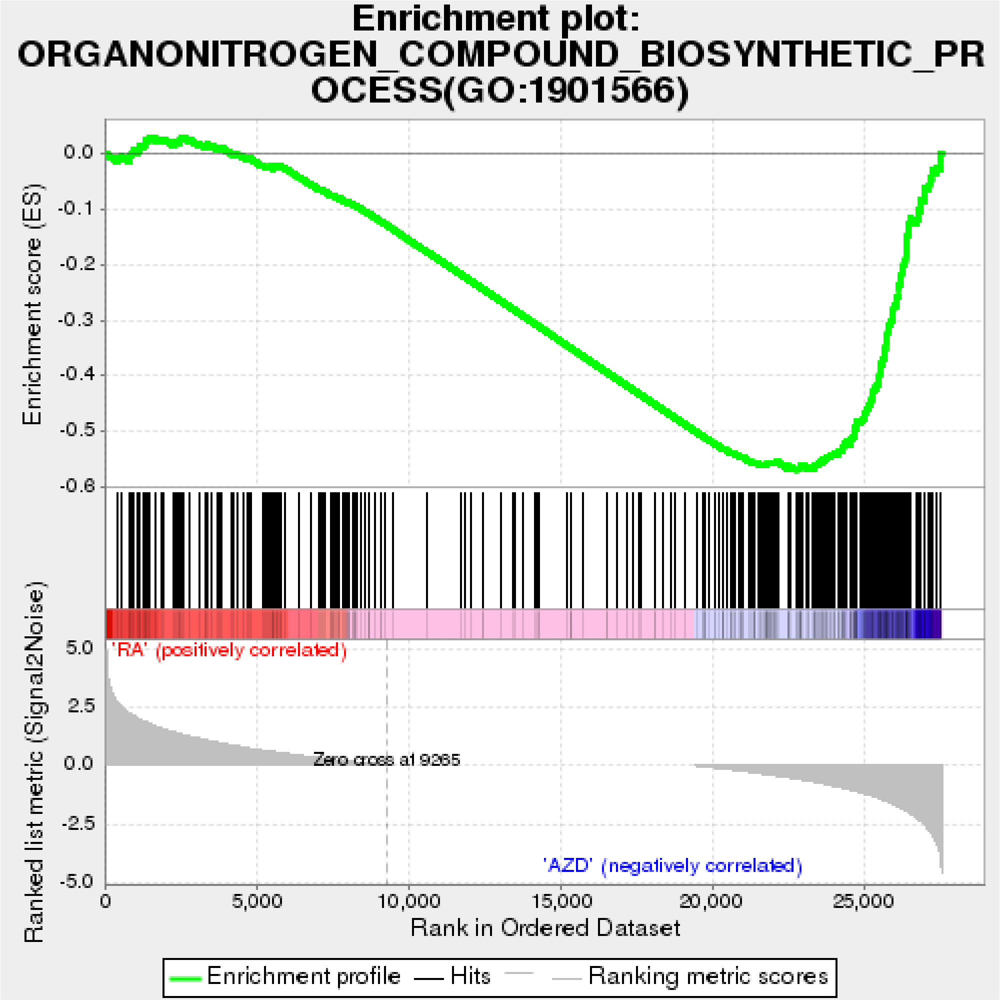

Fig 1: Enrichment plot: ORGANONITROGEN_COMPOUND_BIOSYNTHETIC_PROCESS(GO:1901566) Profile of the Running ES Score & Positions of GeneSet Members on the Rank Ordered List

Table: GSEA details [plain text format]

	PROBE	DESCRIPTION (from dataset)	GENE SYMBOL	GENE_TITLE	RANK IN GENE LIST	RANK METRIC SCORE	RUNNING ES	CORE ENRICHMENT
1	PITG_18256	PITG_18256			364	2.717	-0.0058	No
2	PITG_06273	PITG_06273			468	2.583	-0.0024	No
3	PITG_06684	PITG_06684			748	2.265	-0.0064	No
4	PITG_02050	PITG_02050			776	2.237	-0.0011	No
5	PITG_15001	PITG_15001			834	2.193	0.0029	No
6	PITG_14634	PITG_14634			891	2.148	0.0068	No
7	PITG_19121	PITG_19121			1014	2.048	0.0080	No
8	PITG_09400	PITG_09400			1065	2.019	0.0118	No
9	PITG_05953	PITG_05953			1081	2.007	0.0168	No
10	PITG_13024	PITG_13024			1235	1.911	0.0165	No
11	PITG_04708	PITG_04708			1272	1.888	0.0204	No
12	PITG_02384	PITG_02384			1299	1.868	0.0247	No
13	PITG_17661	PITG_17661			1349	1.838	0.0280	No
14	PITG_20634	PITG_20634			1436	1.785	0.0298	No
15	PITG_01072	PITG_01072			1606	1.693	0.0283	No
16	PITG_10448	PITG_10448			1785	1.624	0.0263	No
17	PITG_15629	PITG_15629			1886	1.577	0.0270	No
18	PITG_03060	PITG_03060			2186	1.456	0.0200	No
19	PITG_21941	PITG_21941			2263	1.424	0.0212	No
20	PITG_00543	PITG_00543			2335	1.398	0.0225	No
21	PITG_16016	PITG_16016			2415	1.374	0.0234	No
22	PITG_18265	PITG_18265			2434	1.369	0.0266	No
23	PITG_10652	PITG_10652			2479	1.355	0.0287	No
24	PITG_01653	PITG_01653			2530	1.335	0.0306	No
25	PITG_11630	PITG_11630			2711	1.279	0.0275	No
26	Novel00922	Novel00922			3038	1.181	0.0188	No
27	PITG_03660	PITG_03660			3265	1.116	0.0136	No
28	PITG_18266	PITG_18266			3288	1.110	0.0159	No
29	PITG_08348	PITG_08348			3318	1.099	0.0179	No
30	PITG_20211	PITG_20211			3478	1.056	0.0150	No
31	PITG_06280	PITG_06280			3639	1.012	0.0119	No
32	PITG_18262	PITG_18262			3739	0.987	0.0110	No
33	PITG_02854	PITG_02854			3807	0.968	0.0113	No
34	PITG_15417	PITG_15417			4137	0.885	0.0016	No
35	PITG_06685	PITG_06685			4180	0.877	0.0025	No
36	PITG_00430	PITG_00430			4330	0.843	-0.0006	No

37	PITG_16088	PITG_16088			4491	0.802	-0.0043	No
38	PITG_02393	PITG_02393			4631	0.772	-0.0072	No
39	PITG_14808	PITG_14808			4728	0.751	-0.0087	No
40	PITG_18257	PITG_18257			4747	0.748	-0.0073	No
41	PITG_01188	PITG_01188			5156	0.661	-0.0204	No
42	PITG_13043	PITG_13043			5270	0.635	-0.0228	No
43	PITG_17126	PITG_17126			5319	0.625	-0.0228	No
44	PITG_06783	PITG_06783			5351	0.620	-0.0222	No
45	PITG_07234	PITG_07234			5449	0.602	-0.0241	No
46	PITG_15000	PITG_15000			5509	0.592	-0.0247	No
47	PITG_02750	PITG_02750			5528	0.588	-0.0237	No
48	PITG_19310	PITG_19310			5533	0.588	-0.0222	No
49	PITG_10601	PITG_10601			5586	0.575	-0.0225	No
50	PITG_07866	PITG_07866			5655	0.562	-0.0234	No
51	PITG_02757	PITG_02757			5656	0.562	-0.0219	No
52	PITG_01195	PITG_01195			5728	0.549	-0.0230	No
53	PITG_06688	PITG_06688			5758	0.543	-0.0225	No
54	PITG_08761	PITG_08761			5775	0.539	-0.0216	No
55	PITG_17651	PITG_17651			5906	0.516	-0.0250	No
56	PITG_22572	PITG_22572			6371	0.437	-0.0408	No
57	PITG_06845	PITG_06845			6777	0.367	-0.0547	No
58	PITG_05009	PITG_05009			7048	0.319	-0.0637	No
59	PITG_18255	PITG_18255			7082	0.312	-0.0641	No
60	PITG_19932	PITG_19932			7130	0.307	-0.0649	No
61	PITG_17295	PITG_17295			7199	0.300	-0.0666	No
62	PITG_06279	PITG_06279			7424	0.261	-0.0741	No
63	PITG_18279	PITG_18279			7436	0.259	-0.0738	No
64	PITG_15003	PITG_15003			7471	0.254	-0.0743	No
65	PITG_06738	PITG_06738			7493	0.250	-0.0744	No
66	PITG_02992	PITG_02992			7548	0.243	-0.0757	No
67	PITG_03093	PITG_03093			7622	0.232	-0.0778	No
68	PITG_16280	PITG_16280			7708	0.220	-0.0803	No
69	PITG_10516	PITG_10516			7711	0.219	-0.0797	No
70	PITG_08810	PITG_08810			7804	0.204	-0.0826	No
71	PITG_12037	PITG_12037			7907	0.193	-0.0858	No
72	PITG_03456	PITG_03456			7938	0.188	-0.0864	No
73	PITG_18258	PITG_18258			7971	0.184	-0.0870	No
74	PITG_09402	PITG_09402			7984	0.183	-0.0870	No
75	PITG_06724	PITG_06724			7999	0.181	-0.0870	No

76	PITG_02489	PITG_02489			8030	0.177	-0.0876	No
77	PITG_17153	PITG_17153			8119	0.169	-0.0903	No
78	PITG_00570	PITG_00570			8143	0.166	-0.0907	No
79	PITG_20746	PITG_20746			8201	0.157	-0.0924	No
80	PITG_14920	PITG_14920			8217	0.154	-0.0925	No
81	PITG_04715	PITG_04715			8219	0.154	-0.0921	No
82	PITG_02291	PITG_02291			8306	0.141	-0.0949	No
83	PITG_03738	PITG_03738			8391	0.130	-0.0976	No
84	PITG_18261	PITG_18261			8533	0.109	-0.1025	No
85	PITG_18275	PITG_18275			8639	0.095	-0.1061	No
86	PITG_19429	PITG_19429			8646	0.094	-0.1061	No
87	PITG_17663	PITG_17663			8670	0.090	-0.1066	No
88	PITG_03700	PITG_03700			8871	0.057	-0.1138	No
89	PITG_09394	PITG_09394			9068	0.027	-0.1210	No
90	PITG_03634	PITG_03634			9084	0.026	-0.1215	No
91	PITG_14699	PITG_14699			9188	0.012	-0.1252	No
92	PITG_20640	PITG_20640			9457	0.000	-0.1351	No
93	PITG_22310	PITG_22310			10562	0.000	-0.1757	No
94	PITG_21582	PITG_21582			11683	0.000	-0.2169	No
95	PITG_20824	PITG_20824			11824	0.000	-0.2220	No
96	PITG_10610	PITG_10610			12049	0.000	-0.2303	No
97	PITG_15722	PITG_15722			12398	0.000	-0.2431	No
98	PITG_22058	PITG_22058			12412	0.000	-0.2435	No
99	PITG_01091	PITG_01091			13039	0.000	-0.2666	No
100	PITG_05812	PITG_05812			13417	0.000	-0.2804	No
101	PITG_20131	PITG_20131			13455	0.000	-0.2818	No
102	PITG_09431	PITG_09431			13717	0.000	-0.2914	No
103	PITG_17187	PITG_17187			14156	0.000	-0.3075	No
104	PITG_06873	PITG_06873			14206	0.000	-0.3093	No
105	PITG_19379	PITG_19379			14298	0.000	-0.3127	No
106	PITG_19374	PITG_19374			14299	0.000	-0.3127	No
107	PITG_21202	PITG_21202			15178	0.000	-0.3449	No
108	PITG_14310	PITG_14310			15340	0.000	-0.3509	No
109	PITG_14312	PITG_14312			15341	0.000	-0.3509	No
110	PITG_14315	PITG_14315			15343	0.000	-0.3509	No
111	PITG_16530	PITG_16530			15706	0.000	-0.3642	No
112	PITG_14346	PITG_14346			16494	0.000	-0.3932	No
113	PITG_14344	PITG_14344			16495	0.000	-0.3932	No
114	PITG_18225	PITG_18225			16859	0.000	-0.4065	No

115	PITG_22629	PITG_22629			17194	0.000	-0.4188	No
116	PITG_21979	PITG_21979			17341	0.000	-0.4242	No
117	PITG_14322	PITG_14322			17578	0.000	-0.4329	No
118	PITG_20240	PITG_20240			17604	0.000	-0.4338	No
119	PITG_03806	PITG_03806			18089	0.000	-0.4516	No
120	PITG_03807	PITG_03807			18090	0.000	-0.4516	No
121	PITG_05850	PITG_05850			18333	0.000	-0.4605	No
122	PITG_18553	PITG_18553			18597	0.000	-0.4701	No
123	PITG_04594	PITG_04594			18776	0.000	-0.4767	No
124	PITG_14325	PITG_14325			19076	0.000	-0.4877	No
125	PITG_10193	PITG_10193			19492	-0.015	-0.5029	No
126	PITG_13172	PITG_13172			19672	-0.037	-0.5094	No
127	PITG_09092	PITG_09092			19694	-0.040	-0.5101	No
128	PITG_13042	PITG_13042			19712	-0.043	-0.5106	No
129	PITG_18303	PITG_18303			19751	-0.048	-0.5118	No
130	PITG_09791	PITG_09791			19763	-0.050	-0.5121	No
131	PITG_06015	PITG_06015			19886	-0.071	-0.5164	No
132	PITG_03480	PITG_03480			20045	-0.095	-0.5219	No
133	PITG_21349	PITG_21349			20105	-0.104	-0.5238	No
134	PITG_12540	PITG_12540			20191	-0.117	-0.5266	No
135	PITG_09666	PITG_09666			20334	-0.138	-0.5315	No
136	PITG_09846	PITG_09846			20357	-0.142	-0.5319	No
137	PITG_01255	PITG_01255			20360	-0.142	-0.5315	No
138	PITG_14557	PITG_14557			20366	-0.143	-0.5313	No
139	PITG_04747	PITG_04747			20459	-0.156	-0.5343	No
140	PITG_09393	PITG_09393			20582	-0.174	-0.5383	No
141	PITG_16741	PITG_16741			20622	-0.179	-0.5392	No
142	PITG_03681	PITG_03681			20625	-0.180	-0.5388	No
143	PITG_13991	PITG_13991			20651	-0.186	-0.5392	No
144	PITG_04729	PITG_04729			20710	-0.194	-0.5408	No
145	PITG_18276	PITG_18276			20741	-0.197	-0.5413	No
146	PITG_06022	PITG_06022			20893	-0.220	-0.5463	No
147	PITG_02493	PITG_02493			20900	-0.221	-0.5459	No
148	PITG_18296	PITG_18296			20914	-0.224	-0.5457	No
149	PITG_04774	PITG_04774			20937	-0.229	-0.5459	No
150	PITG_10077	PITG_10077			20981	-0.236	-0.5468	No
151	PITG_05730	PITG_05730			21018	-0.243	-0.5475	No
152	PITG_06518	PITG_06518			21226	-0.277	-0.5543	No
153	PITG_01193	PITG_01193			21260	-0.285	-0.5547	No

154	PITG_16057	PITG_16057			21307	-0.293	-0.5556	No
155	PITG_17664	PITG_17664			21382	-0.306	-0.5575	No
156	PITG_05853	PITG_05853			21389	-0.307	-0.5569	No
157	PITG_12077	PITG_12077			21406	-0.311	-0.5566	No
158	PITG_11734	PITG_11734			21411	-0.312	-0.5559	No
159	PITG_11733	PITG_11733			21492	-0.325	-0.5579	No
160	PITG_17133	PITG_17133			21566	-0.336	-0.5597	No
161	PITG_13347	PITG_13347			21592	-0.341	-0.5596	No
162	PITG_18277	PITG_18277			21660	-0.353	-0.5611	No
163	PITG_12151	PITG_12151			21701	-0.361	-0.5616	No
164	PITG_01235	PITG_01235			21702	-0.361	-0.5606	No
165	PITG_16328	PITG_16328			21756	-0.372	-0.5615	No
166	PITG_12181	PITG_12181			21763	-0.373	-0.5607	No
167	PITG_06749	PITG_06749			21769	-0.375	-0.5598	No
168	PITG_10519	PITG_10519			21779	-0.377	-0.5591	No
169	PITG_14850	PITG_14850			21815	-0.384	-0.5593	No
170	PITG_13148	PITG_13148			21820	-0.385	-0.5584	No
171	PITG_16757	PITG_16757			21821	-0.385	-0.5573	No
172	PITG_05245	PITG_05245			21857	-0.392	-0.5575	No
173	PITG_14918	PITG_14918			21886	-0.397	-0.5574	No
174	PITG_19364	PITG_19364			21911	-0.402	-0.5572	No
175	PITG_07201	PITG_07201			21935	-0.406	-0.5569	No
176	PITG_09596	PITG_09596			21965	-0.412	-0.5568	No
177	PITG_07797	PITG_07797			21968	-0.412	-0.5558	No
178	PITG_03799	PITG_03799			22019	-0.421	-0.5564	No
179	PITG_04610	PITG_04610			22066	-0.429	-0.5569	No
180	PITG_05405	PITG_05405			22072	-0.429	-0.5559	No
181	PITG_05733	PITG_05733			22091	-0.432	-0.5554	No
182	PITG_08669	PITG_08669			22119	-0.437	-0.5551	No
183	PITG_20188	PITG_20188			22157	-0.445	-0.5553	No
184	PITG_15294	PITG_15294			22488	-0.507	-0.5660	No
185	PITG_05007	PITG_05007			22498	-0.509	-0.5649	No
186	PITG_19669	PITG_19669			22503	-0.510	-0.5636	No
187	PITG_12839	PITG_12839			22518	-0.514	-0.5627	No
188	PITG_02394	PITG_02394			22610	-0.535	-0.5646	No
189	PITG_06222	PITG_06222			22748	-0.566	-0.5680	No
190	PITG_12692	PITG_12692			22807	-0.578	-0.5686	Yes
191	PITG_10979	PITG_10979			22822	-0.583	-0.5675	Yes
192	PITG_02925	PITG_02925			22842	-0.586	-0.5665	Yes

193	PITG_19999	PITG_19999			22878	-0.594	-0.5662	Yes
194	PITG_13832	PITG_13832			22885	-0.595	-0.5647	Yes
195	PITG_00257	PITG_00257			22922	-0.600	-0.5644	Yes
196	PITG_04918	PITG_04918			22927	-0.602	-0.5628	Yes
197	PITG_00443	PITG_00443			22936	-0.604	-0.5615	Yes
198	PITG_05354	PITG_05354			22976	-0.613	-0.5612	Yes
199	PITG_02921	PITG_02921			23131	-0.650	-0.5650	Yes
200	PITG_09640	PITG_09640			23185	-0.660	-0.5652	Yes
201	PITG_12961	PITG_12961			23203	-0.663	-0.5639	Yes
202	PITG_12745	PITG_12745			23280	-0.681	-0.5648	Yes
203	PITG_01804	PITG_01804			23342	-0.693	-0.5651	Yes
204	PITG_14609	PITG_14609			23351	-0.695	-0.5635	Yes
205	PITG_02580	PITG_02580			23358	-0.695	-0.5618	Yes
206	PITG_01862	PITG_01862			23364	-0.696	-0.5600	Yes
207	PITG_02294	PITG_02294			23416	-0.708	-0.5599	Yes
208	PITG_11111	PITG_11111			23461	-0.720	-0.5596	Yes
209	PITG_15090	PITG_15090			23482	-0.725	-0.5583	Yes
210	PITG_04843	PITG_04843			23523	-0.735	-0.5577	Yes
211	PITG_02136	PITG_02136			23534	-0.738	-0.5560	Yes
212	PITG_16008	PITG_16008			23545	-0.740	-0.5543	Yes
213	PITG_09698	PITG_09698			23607	-0.757	-0.5545	Yes
214	PITG_17748	PITG_17748			23641	-0.765	-0.5535	Yes
215	PITG_13735	PITG_13735			23644	-0.766	-0.5515	Yes
216	PITG_18259	PITG_18259			23713	-0.785	-0.5518	Yes
217	PITG_06771	PITG_06771			23729	-0.789	-0.5502	Yes
218	PITG_12588	PITG_12588			23738	-0.792	-0.5482	Yes
219	PITG_08369	PITG_08369			23772	-0.802	-0.5472	Yes
220	PITG_10887	PITG_10887			23788	-0.807	-0.5455	Yes
221	PITG_20189	PITG_20189			23835	-0.820	-0.5449	Yes
222	PITG_03420	PITG_03420			23870	-0.829	-0.5439	Yes
223	PITG_05374	PITG_05374			23918	-0.844	-0.5433	Yes
224	PITG_15723	PITG_15723			23962	-0.858	-0.5425	Yes
225	PITG_01922	PITG_01922			24019	-0.874	-0.5421	Yes
226	PITG_21313	PITG_21313			24020	-0.874	-0.5396	Yes
227	PITG_09550	PITG_09550			24034	-0.880	-0.5377	Yes
228	PITG_10974	PITG_10974			24137	-0.910	-0.5389	Yes
229	PITG_10080	PITG_10080			24151	-0.913	-0.5368	Yes
230	PITG_09547	PITG_09547			24182	-0.924	-0.5354	Yes
231	PITG_12864	PITG_12864			24223	-0.936	-0.5342	Yes

232	PITG_04703	PITG_04703			24269	-0.949	-0.5332	Yes
233	PITG_21071	PITG_21071			24300	-0.958	-0.5317	Yes
234	PITG_18298	PITG_18298			24307	-0.960	-0.5292	Yes
235	PITG_18271	PITG_18271			24322	-0.966	-0.5270	Yes
236	PITG_02694	PITG_02694			24328	-0.970	-0.5245	Yes
237	PITG_04487	PITG_04487			24365	-0.980	-0.5231	Yes
238	PITG_01943	PITG_01943			24378	-0.982	-0.5208	Yes
239	PITG_11923	PITG_11923			24405	-0.990	-0.5190	Yes
240	PITG_03274	PITG_03274			24545	-1.036	-0.5213	Yes
241	PITG_03221	PITG_03221			24557	-1.040	-0.5188	Yes
242	PITG_09635	PITG_09635			24568	-1.043	-0.5162	Yes
243	PITG_12050	PITG_12050			24576	-1.046	-0.5136	Yes
244	PITG_10777	PITG_10777			24618	-1.057	-0.5122	Yes
245	PITG_11766	PITG_11766			24642	-1.064	-0.5100	Yes
246	PITG_07841	PITG_07841			24668	-1.073	-0.5080	Yes
247	PITG_15015	PITG_15015			24677	-1.075	-0.5053	Yes
248	PITG_18278	PITG_18278			24697	-1.082	-0.5030	Yes
249	PITG_04698	PITG_04698			24698	-1.082	-0.4999	Yes
250	PITG_00688	PITG_00688			24703	-1.083	-0.4971	Yes
251	PITG_13586	PITG_13586			24704	-1.083	-0.4941	Yes
252	PITG_03322	PITG_03322			24740	-1.096	-0.4923	Yes
253	PITG_08157	PITG_08157			24750	-1.102	-0.4896	Yes
254	PITG_09576	PITG_09576			24756	-1.103	-0.4867	Yes
255	PITG_06995	PITG_06995			24760	-1.104	-0.4837	Yes
256	PITG_01833	PITG_01833			24767	-1.106	-0.4809	Yes
257	PITG_01762	PITG_01762			24911	-1.153	-0.4829	Yes
258	PITG_05171	PITG_05171			24921	-1.158	-0.4800	Yes
259	PITG_00566	PITG_00566			24953	-1.171	-0.4779	Yes
260	PITG_06016	PITG_06016			25004	-1.191	-0.4764	Yes
261	PITG_22249	PITG_22249			25013	-1.195	-0.4734	Yes
262	PITG_15069	PITG_15069			25019	-1.198	-0.4702	Yes
263	PITG_11178	PITG_11178			25025	-1.201	-0.4671	Yes
264	PITG_16366	PITG_16366			25055	-1.210	-0.4648	Yes
265	PITG_00941	PITG_00941			25067	-1.214	-0.4618	Yes
266	PITG_07888	PITG_07888			25072	-1.216	-0.4586	Yes
267	PITG_18251	PITG_18251			25141	-1.242	-0.4576	Yes
268	PITG_12697	PITG_12697			25164	-1.249	-0.4549	Yes
269	PITG_13371	PITG_13371			25168	-1.249	-0.4516	Yes
270	PITG_15407	PITG_15407			25203	-1.263	-0.4493	Yes

271	PITG_03698	PITG_03698			25237	-1.274	-0.4470	Yes
272	PITG_19557	PITG_19557			25245	-1.278	-0.4437	Yes
273	PITG_03999	PITG_03999			25257	-1.283	-0.4405	Yes
274	PITG_14765	PITG_14765			25258	-1.283	-0.4369	Yes
275	PITG_05174	PITG_05174			25267	-1.288	-0.4336	Yes
276	PITG_03294	PITG_03294			25303	-1.306	-0.4313	Yes
277	PITG_08703	PITG_08703			25308	-1.308	-0.4278	Yes
278	PITG_07173	PITG_07173			25342	-1.325	-0.4253	Yes
279	PITG_09553	PITG_09553			25359	-1.334	-0.4222	Yes
280	PITG_14913	PITG_14913			25405	-1.356	-0.4201	Yes
281	PITG_18052	PITG_18052			25424	-1.365	-0.4169	Yes
282	PITG_08959	PITG_08959			25458	-1.380	-0.4143	Yes
283	PITG_03235	PITG_03235			25480	-1.389	-0.4112	Yes
284	PITG_03661	PITG_03661			25487	-1.391	-0.4076	Yes
285	PITG_04382	PITG_04382			25503	-1.398	-0.4042	Yes
286	PITG_02397	PITG_02397			25510	-1.400	-0.4005	Yes
287	PITG_09582	PITG_09582			25513	-1.402	-0.3967	Yes
288	PITG_14729	PITG_14729			25528	-1.410	-0.3933	Yes
289	PITG_07141	PITG_07141			25545	-1.420	-0.3899	Yes
290	PITG_09506	PITG_09506			25563	-1.431	-0.3866	Yes
291	PITG_04418	PITG_04418			25564	-1.431	-0.3826	Yes
292	PITG_08579	PITG_08579			25572	-1.438	-0.3788	Yes
293	PITG_19399	PITG_19399			25621	-1.462	-0.3765	Yes
294	PITG_14195	PITG_14195			25624	-1.463	-0.3725	Yes
295	PITG_05851	PITG_05851			25628	-1.465	-0.3686	Yes
296	PITG_04992	PITG_04992			25670	-1.484	-0.3660	Yes
297	PITG_18272	PITG_18272			25674	-1.486	-0.3619	Yes
298	PITG_10146	PITG_10146			25685	-1.491	-0.3581	Yes
299	PITG_09540	PITG_09540			25692	-1.493	-0.3542	Yes
300	PITG_00523	PITG_00523			25696	-1.497	-0.3502	Yes
301	PITG_09234	PITG_09234			25712	-1.502	-0.3465	Yes
302	PITG_10863	PITG_10863			25713	-1.502	-0.3423	Yes
303	PITG_07405	PITG_07405			25728	-1.511	-0.3387	Yes
304	PITG_20965	PITG_20965			25743	-1.516	-0.3349	Yes
305	PITG_06237	PITG_06237			25757	-1.523	-0.3312	Yes
306	PITG_08808	PITG_08808			25759	-1.524	-0.3270	Yes
307	PITG_17785	PITG_17785			25774	-1.532	-0.3232	Yes
308	PITG_03353	PITG_03353			25780	-1.535	-0.3191	Yes
309	PITG_12930	PITG_12930			25785	-1.538	-0.3150	Yes

310	PITG_07300	PITG_07300			25805	-1.550	-0.3114	Yes
311	PITG_20264	PITG_20264			25831	-1.561	-0.3080	Yes
312	PITG_00302	PITG_00302			25872	-1.579	-0.3050	Yes
313	PITG_01769	PITG_01769			25892	-1.589	-0.3013	Yes
314	PITG_06019	PITG_06019			25918	-1.604	-0.2978	Yes
315	PITG_22112	PITG_22112			25925	-1.611	-0.2935	Yes
316	Novel00015	Novel00015			25949	-1.627	-0.2898	Yes
317	PITG_14456	PITG_14456			25952	-1.628	-0.2854	Yes
318	PITG_04683	PITG_04683			25968	-1.637	-0.2814	Yes
319	PITG_04419	PITG_04419			25974	-1.640	-0.2770	Yes
320	PITG_12947	PITG_12947			26003	-1.658	-0.2734	Yes
321	PITG_03239	PITG_03239			26044	-1.683	-0.2702	Yes
322	PITG_02578	PITG_02578			26070	-1.705	-0.2663	Yes
323	PITG_19531	PITG_19531			26082	-1.713	-0.2620	Yes
324	PITG_03460	PITG_03460			26094	-1.719	-0.2576	Yes
325	PITG_03178	PITG_03178			26113	-1.732	-0.2534	Yes
326	PITG_19428	PITG_19428			26114	-1.733	-0.2486	Yes
327	PITG_01245	PITG_01245			26115	-1.733	-0.2438	Yes
328	PITG_18053	PITG_18053			26117	-1.734	-0.2390	Yes
329	PITG_14639	PITG_14639			26157	-1.764	-0.2355	Yes
330	PITG_13831	PITG_13831			26183	-1.780	-0.2315	Yes
331	PITG_08129	PITG_08129			26202	-1.791	-0.2272	Yes
332	PITG_07792	PITG_07792			26226	-1.805	-0.2230	Yes
333	PITG_00910	PITG_00910			26229	-1.807	-0.2180	Yes
334	PITG_13681	PITG_13681			26238	-1.816	-0.2133	Yes
335	PITG_06821	PITG_06821			26267	-1.833	-0.2092	Yes
336	PITG_09552	PITG_09552			26270	-1.835	-0.2042	Yes
337	PITG_19157	PITG_19157			26301	-1.850	-0.2001	Yes
338	PITG_02392	PITG_02392			26315	-1.857	-0.1954	Yes
339	PITG_12300	PITG_12300			26358	-1.891	-0.1917	Yes
340	PITG_06636	PITG_06636			26370	-1.898	-0.1868	Yes
341	PITG_09631	PITG_09631			26373	-1.899	-0.1816	Yes
342	PITG_01042	PITG_01042			26385	-1.915	-0.1767	Yes
343	PITG_00632	PITG_00632			26390	-1.921	-0.1715	Yes
344	PITG_09726	PITG_09726			26397	-1.925	-0.1663	Yes
345	PITG_00397	PITG_00397			26402	-1.929	-0.1611	Yes
346	PITG_00132	PITG_00132			26411	-1.939	-0.1560	Yes
347	PITG_10089	PITG_10089			26420	-1.946	-0.1509	Yes
348	PITG_09555	PITG_09555			26429	-1.950	-0.1458	Yes

349	PITG_03598	PITG_03598			26463	-1.987	-0.1414	Yes
350	PITG_15526	PITG_15526			26465	-1.988	-0.1359	Yes
351	PITG_00074	PITG_00074			26475	-1.996	-0.1307	Yes
352	PITG_13399	PITG_13399			26492	-2.007	-0.1257	Yes
353	PITG_09521	PITG_09521			26522	-2.027	-0.1211	Yes
354	PITG_07991	PITG_07991			26527	-2.030	-0.1156	Yes
355	PITG_02039	PITG_02039			26754	-2.231	-0.1177	Yes
356	PITG_03768	PITG_03768			26780	-2.261	-0.1124	Yes
357	PITG_14936	PITG_14936			26810	-2.306	-0.1070	Yes
358	PITG_00571	PITG_00571			26814	-2.307	-0.1007	Yes
359	PITG_06596	PITG_06596			26861	-2.351	-0.0959	Yes
360	PITG_16736	PITG_16736			26862	-2.351	-0.0893	Yes
361	PITG_18054	PITG_18054			26868	-2.362	-0.0829	Yes
362	PITG_17607	PITG_17607			26969	-2.477	-0.0797	Yes
363	PITG_06021	PITG_06021			26996	-2.511	-0.0737	Yes
364	PITG_18545	PITG_18545			27014	-2.541	-0.0672	Yes
365	PITG_22020	PITG_22020			27018	-2.545	-0.0602	Yes
366	PITG_18270	PITG_18270			27151	-2.759	-0.0574	Yes
367	PITG_21661	PITG_21661			27161	-2.778	-0.0500	Yes
368	PITG_08714	PITG_08714			27196	-2.846	-0.0433	Yes
369	PITG_00471	PITG_00471			27228	-2.898	-0.0364	Yes
370	PITG_10110	PITG_10110			27232	-2.902	-0.0284	Yes
371	PITG_01062	PITG_01062			27369	-3.233	-0.0244	Yes
372	PITG_14352	PITG_14352			27505	-3.705	-0.0191	Yes
373	PITG_19993	PITG_19993			27511	-3.727	-0.0089	Yes
374	PITG_02621	PITG_02621			27522	-3.845	0.0014	Yes

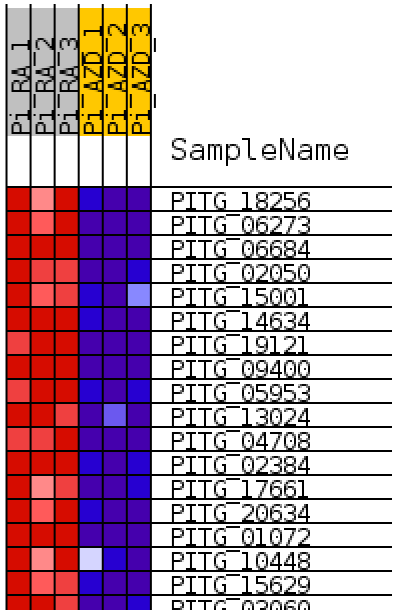

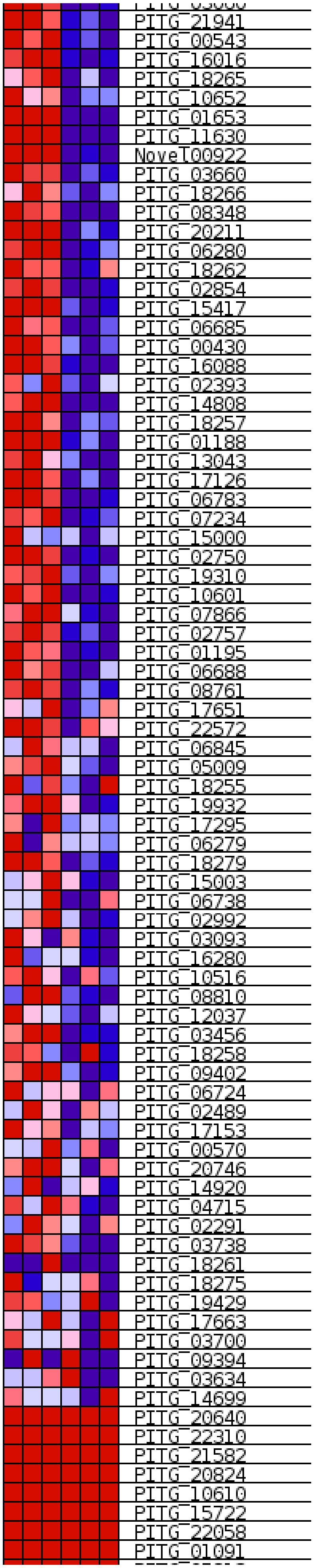
PITG_21941
PITG_00543
PITG_16016
PITG_18265
PITG_10652
PITG_01653
PITG_11630
Novel00922
PITG_03660
PITG_18266
PITG_08348
PITG_20211
PITG_06280
PITG_18262
PITG_02854
PITG_15417
PITG_06685
PITG_00430
PITG_16088
PITG_02393
PITG_14808
PITG_18257
PITG_01188
PITG_13043
PITG_17126
PITG_06783
PITG_07234
PITG_15000
PITG_02750
PITG_19310
PITG_10601
PITG_07866
PITG_02757
PITG_01195
PITG_06688
PITG_08761
PITG_17651
PITG_22572
PITG_06845
PITG_05009
PITG_18255
PITG_19932
PITG_17295
PITG_06279
PITG_18279
PITG_15003
PITG_06738
PITG_02992
PITG_03093
PITG_16280
PITG_10516
PITG_08810
PITG_12037
PITG_03456
PITG_18258
PITG_09402
PITG_06724
PITG_02489
PITG_17153
PITG_00570
PITG_20746
PITG_14920
PITG_04715
PITG_02291
PITG_03738
PITG_18261
PITG_18275
PITG_19429
PITG_17663
PITG_03700
PITG_09394
PITG_03634
PITG_14699
PITG_20640
PITG_22310
PITG_21582
PITG_20824
PITG_10610
PITG_15722
PITG_22058
PITG_01091

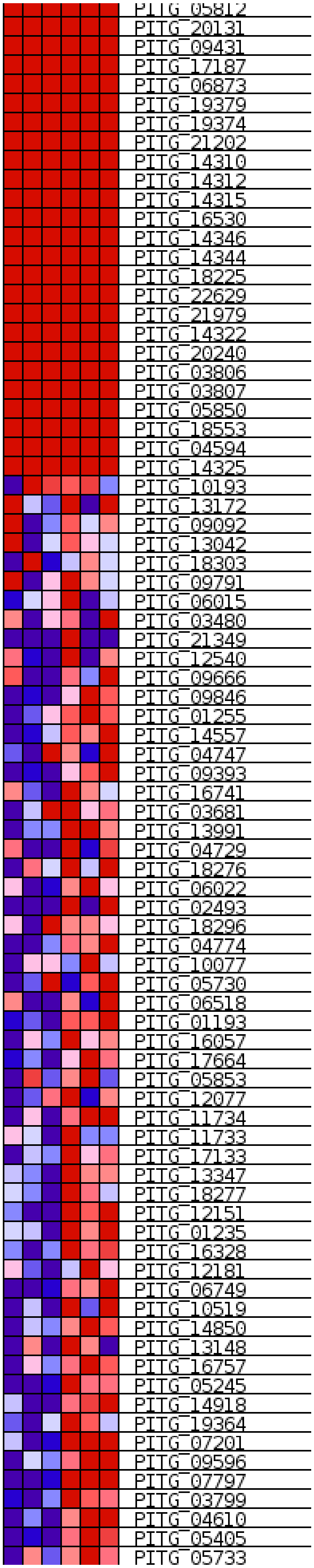
PITG_05812
PITG_20131
PITG_09431
PITG_17187
PITG_06873
PITG_19379
PITG_19374
PITG_21202
PITG_14310
PITG_14312
PITG_14315
PITG_16530
PITG_14346
PITG_14344
PITG_18225
PITG_22629
PITG_21979
PITG_14322
PITG_20240
PITG_03806
PITG_03807
PITG_05850
PITG_18553
PITG_04594
PITG_14325
PITG_10193
PITG_13172
PITG_09092
PITG_13042
PITG_18303
PITG_09791
PITG_06015
PITG_03480
PITG_21349
PITG_12540
PITG_09666
PITG_09846
PITG_01255
PITG_14557
PITG_04747
PITG_09393
PITG_16741
PITG_03681
PITG_13991
PITG_04729
PITG_18276
PITG_06022
PITG_02493
PITG_18296
PITG_04774
PITG_10077
PITG_05730
PITG_06518
PITG_01193
PITG_16057
PITG_17664
PITG_05853
PITG_12077
PITG_11734
PITG_11733
PITG_17133
PITG_13347
PITG_18277
PITG_12151
PITG_01235
PITG_16328
PITG_12181
PITG_06749
PITG_10519
PITG_14850
PITG_13148
PITG_16757
PITG_05245
PITG_14918
PITG_19364
PITG_07201
PITG_09596
PITG_07797
PITG_03799
PITG_04610
PITG_05405
PITG_05733

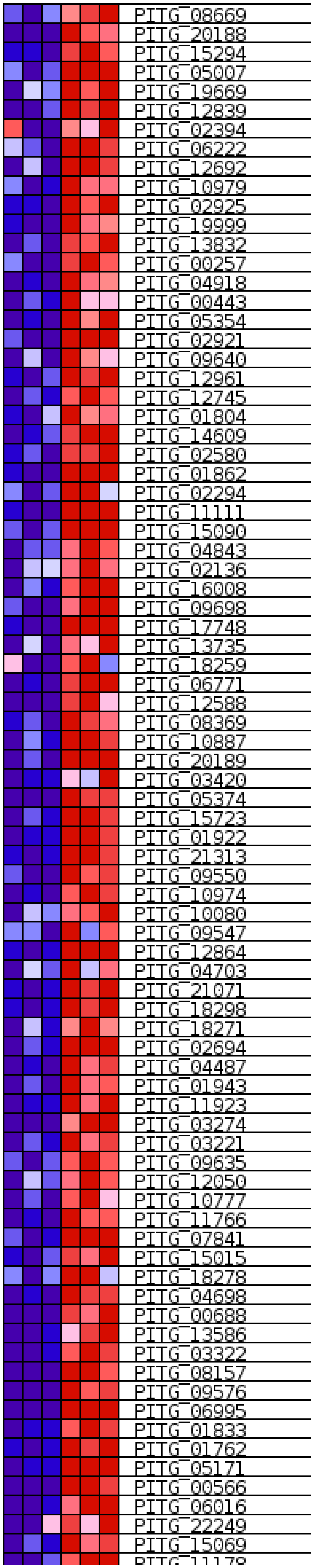
PITG_08669
PITG_20188
PITG_15294
PITG_05007
PITG_19669
PITG_12839
PITG_02394
PITG_06222
PITG_12692
PITG_10979
PITG_02925
PITG_19999
PITG_13832
PITG_00257
PITG_04918
PITG_00443
PITG_05354
PITG_02921
PITG_09640
PITG_12961
PITG_12745
PITG_01804
PITG_14609
PITG_02580
PITG_01862
PITG_02294
PITG_11111
PITG_15090
PITG_04843
PITG_02136
PITG_16008
PITG_09698
PITG_17748
PITG_13735
PITG_18259
PITG_06771
PITG_12588
PITG_08369
PITG_10887
PITG_20189
PITG_03420
PITG_05374
PITG_15723
PITG_01922
PITG_21313
PITG_09550
PITG_10974
PITG_10080
PITG_09547
PITG_12864
PITG_04703
PITG_21071
PITG_18298
PITG_18271
PITG_02694
PITG_04487
PITG_01943
PITG_11923
PITG_03274
PITG_03221
PITG_09635
PITG_12050
PITG_10777
PITG_11766
PITG_07841
PITG_15015
PITG_18278
PITG_04698
PITG_00688
PITG_13586
PITG_03322
PITG_08157
PITG_09576
PITG_06995
PITG_01833
PITG_01762
PITG_05171
PITG_00566
PITG_06016
PITG_22249
PITG_15069

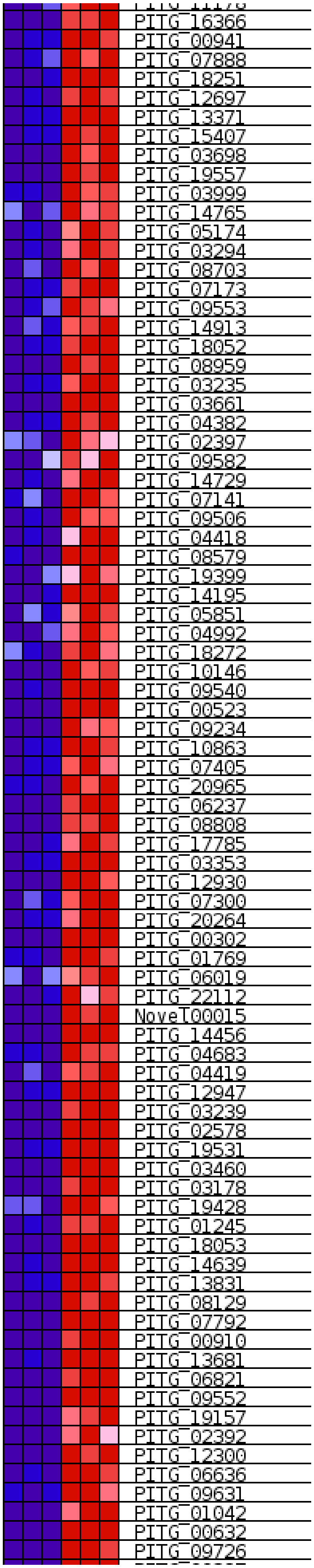

PITG_16366
PITG_00941
PITG_07888
PITG_18251
PITG_12697
PITG_13371
PITG_15407
PITG_03698
PITG_19557
PITG_03999
PITG_14765
PITG_05174
PITG_03294
PITG_08703
PITG_07173
PITG_09553
PITG_14913
PITG_18052
PITG_08959
PITG_03235
PITG_03661
PITG_04382
PITG_02397
PITG_09582
PITG_14729
PITG_07141
PITG_09506
PITG_04418
PITG_08579
PITG_19399
PITG_14195
PITG_05851
PITG_04992
PITG_18272
PITG_10146
PITG_09540
PITG_00523
PITG_09234
PITG_10863
PITG_07405
PITG_20965
PITG_06237
PITG_08808
PITG_17785
PITG_03353
PITG_12930
PITG_07300
PITG_20264
PITG_00302
PITG_01769
PITG_06019
PITG_22112
Novel00015
PITG_14456
PITG_04683
PITG_04419
PITG_12947
PITG_03239
PITG_02578
PITG_19531
PITG_03460
PITG_03178
PITG_19428
PITG_01245
PITG_18053
PITG_14639
PITG_13831
PITG_08129
PITG_07792
PITG_00910
PITG_13681
PITG_06821
PITG_09552
PITG_19157
PITG_02392
PITG_12300
PITG_06636
PITG_09631
PITG_01042
PITG_00632
PITG_09726

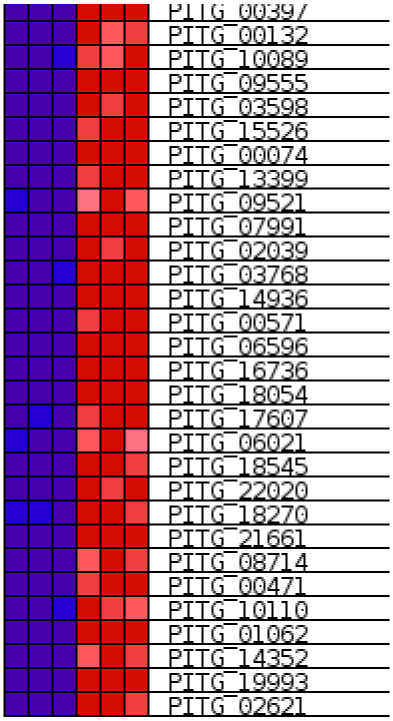

Fig 2: ORGANONITROGEN_COMPOUND_BIOSYNTHETIC_PROCESS(GO:1901566) Blue-Pink O' Gram in the Space of the Analyzed GeneSet

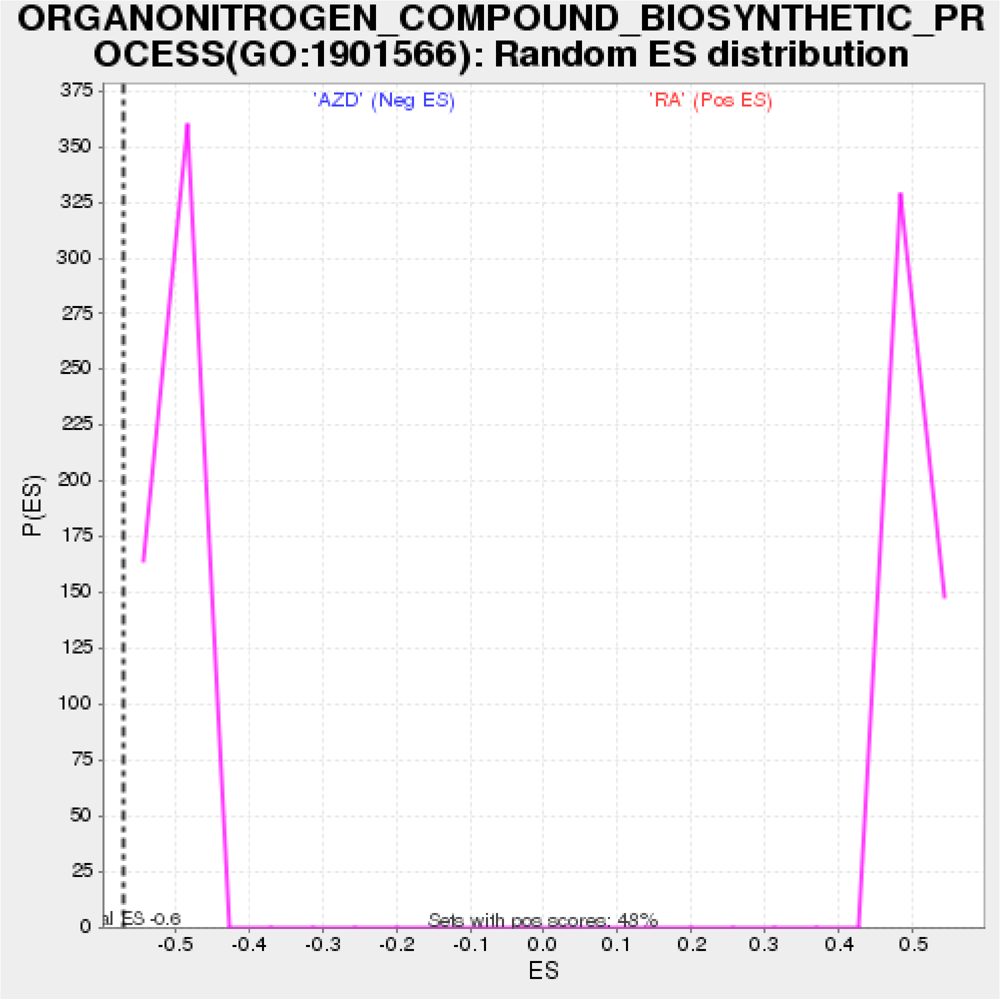

Fig 3: ORGANONITROGEN_COMPOUND_BIOSYNTHETIC_PROCESS(GO:1901566): Random ES distribution

Gene set null distribution of ES for ORGANONITROGEN_COMPOUND_BIOSYNTHETIC_PROCESS(GO:1901566)

3. organonitrogen compound metabolic process

Table: GSEA Results Summary

Dataset	fpkm.sample
Phenotype	sample.cls
Upregulated in class	AZD
GeneSet	ORGANONITROGEN_COMPOUND_METABOLIC_PROCESS(GO:1901564)
Enrichment Score (ES)	-0.33187124
Normalized Enrichment Score (NES)	-1.1317714
Nominal p-value	0.0
FDR q-value	0.10687017
FWER p-Value	0.056

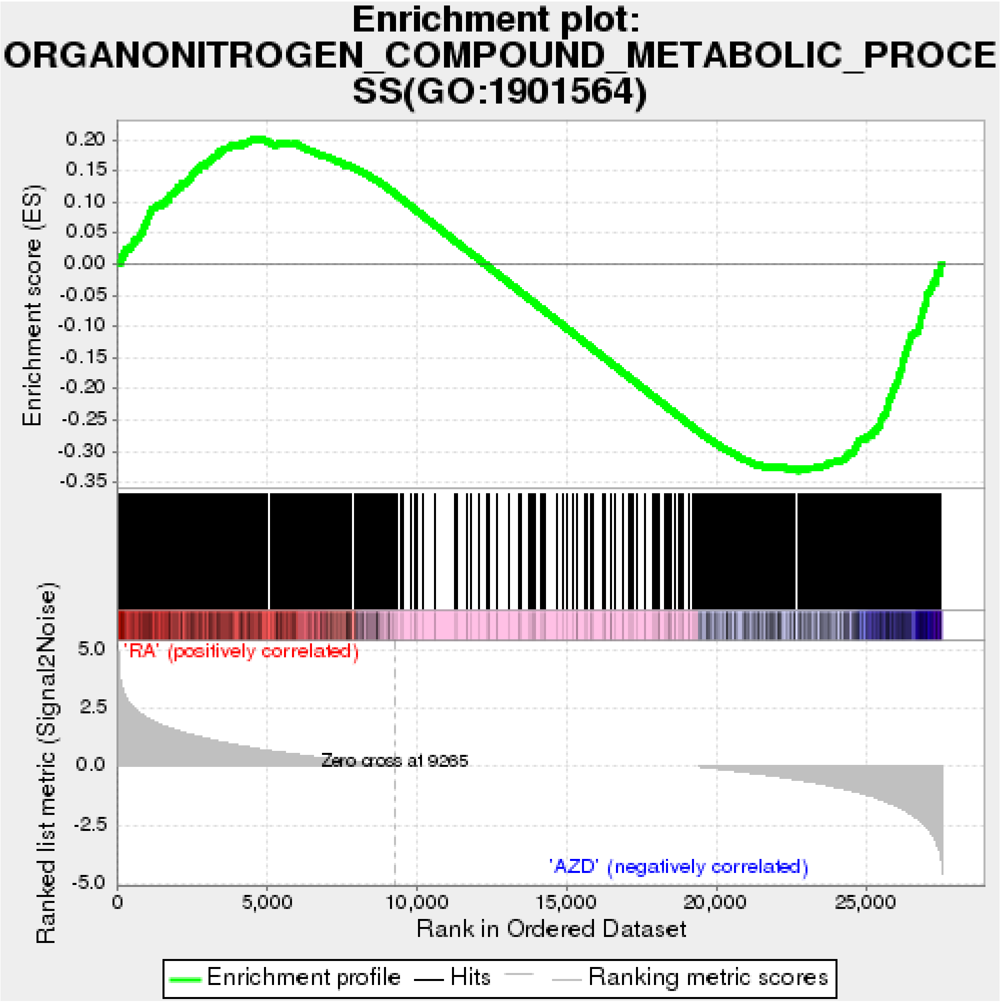

Fig 1: Enrichment plot: ORGANONITROGEN_COMPOUND_METABOLIC_PROCESS(GO:1901564) Profile of the Running ES Score & Positions of GeneSet Members on the Rank Ordered List

Table: GSEA details [plain text format]

	PROBE	DESCRIPTION (from dataset)	GENE SYMBOL	GENE_TITLE	RANK IN GENE LIST	RANK METRIC SCORE	RUNNING ES	CORE ENRICHMENT
1	PITG_13914	PITG_13914			32	4.051	0.0024	No
2	PITG_05365	PITG_05365			70	3.677	0.0043	No
3	PITG_03941	PITG_03941			71	3.672	0.0076	No
4	PITG_00230	PITG_00230			76	3.640	0.0108	No
5	PITG_02767	PITG_02767			86	3.585	0.0136	No
6	PITG_10138	PITG_10138			159	3.220	0.0138	No
7	PITG_18578	PITG_18578			165	3.201	0.0165	No
8	PITG_17577	PITG_17577			184	3.127	0.0186	No
9	PITG_15917	PITG_15917			218	3.020	0.0201	No
10	PITG_15644	PITG_15644			242	2.957	0.0219	No
11	PITG_08784	PITG_08784			248	2.948	0.0243	No
12	PITG_00640	PITG_00640			280	2.882	0.0257	No
13	PITG_18256	PITG_18256			364	2.717	0.0250	No
14	PITG_02821	PITG_02821			366	2.711	0.0274	No
15	PITG_11016	PITG_11016			444	2.604	0.0268	No
16	PITG_00614	PITG_00614			460	2.588	0.0286	No
17	PITG_06273	PITG_06273			468	2.583	0.0307	No
18	PITG_07298	PITG_07298			472	2.581	0.0329	No
19	PITG_20767	PITG_20767			479	2.571	0.0350	No
20	PITG_16204	PITG_16204			494	2.549	0.0367	No
21	PITG_13814	PITG_13814			513	2.535	0.0383	No
22	PITG_00245	PITG_00245			558	2.466	0.0389	No
23	PITG_08549	PITG_08549			577	2.447	0.0404	No
24	PITG_07215	PITG_07215			604	2.414	0.0416	No
25	PITG_05855	PITG_05855			637	2.376	0.0425	No
26	PITG_08725	PITG_08725			692	2.307	0.0425	No
27	PITG_22671	PITG_22671			702	2.292	0.0442	No
28	PITG_02224	PITG_02224			710	2.289	0.0460	No
29	PITG_23090	PITG_23090			733	2.270	0.0472	No
30	PITG_07251	PITG_07251			734	2.270	0.0493	No
31	PITG_06684	PITG_06684			748	2.265	0.0508	No
32	PITG_02050	PITG_02050			776	2.237	0.0518	No
33	PITG_02997	PITG_02997			799	2.218	0.0530	No
34	PITG_10192	PITG_10192			832	2.193	0.0537	No
35	PITG_15001	PITG_15001			834	2.193	0.0556	No
36	PITG_06963	PITG_06963			843	2.186	0.0573	No
37	PITG_13398	PITG_13398			846	2.185	0.0592	No

38	PITG_03020	PITG_03020			855	2.180	0.0609	No
39	PITG_03305	PITG_03305			872	2.168	0.0622	No
40	PITG_06236	PITG_06236			887	2.154	0.0636	No
41	PITG_14634	PITG_14634			891	2.148	0.0654	No
42	PITG_01809	PITG_01809			909	2.126	0.0667	No
43	PITG_21116	PITG_21116			911	2.124	0.0686	No
44	PITG_11607	PITG_11607			947	2.097	0.0691	No
45	PITG_02960	PITG_02960			977	2.074	0.0699	No
46	PITG_00063	PITG_00063			983	2.070	0.0715	No
47	PITG_13934	PITG_13934			995	2.067	0.0730	No
48	PITG_21606	PITG_21606			999	2.064	0.0747	No
49	PITG_08774	PITG_08774			1003	2.062	0.0765	No
50	PITG_19121	PITG_19121			1014	2.048	0.0779	No
51	PITG_06925	PITG_06925			1024	2.044	0.0794	No
52	PITG_23143	PITG_23143			1046	2.029	0.0805	No
53	PITG_00211	PITG_00211			1057	2.022	0.0819	No
54	PITG_09400	PITG_09400			1065	2.019	0.0834	No
55	PITG_05953	PITG_05953			1081	2.007	0.0847	No
56	PITG_02177	PITG_02177			1091	1.999	0.0861	No
57	PITG_20766	PITG_20766			1102	1.992	0.0875	No
58	PITG_07157	PITG_07157			1113	1.983	0.0890	No
59	PITG_17585	PITG_17585			1125	1.974	0.0903	No
60	PITG_14396	PITG_14396			1180	1.945	0.0900	No
61	PITG_16476	PITG_16476			1222	1.919	0.0902	No
62	PITG_11524	PITG_11524			1224	1.919	0.0919	No
63	PITG_13024	PITG_13024			1235	1.911	0.0932	No
64	PITG_04971	PITG_04971			1247	1.904	0.0945	No
65	PITG_04708	PITG_04708			1272	1.888	0.0953	No
66	PITG_02384	PITG_02384			1299	1.868	0.0960	No
67	PITG_02465	PITG_02465			1310	1.862	0.0973	No
68	PITG_17661	PITG_17661			1349	1.838	0.0975	No
69	PITG_20634	PITG_20634			1436	1.785	0.0958	No
70	PITG_16916	PITG_16916			1445	1.779	0.0971	No
71	PITG_00636	PITG_00636			1466	1.768	0.0979	No
72	PITG_14703	PITG_14703			1498	1.752	0.0983	No
73	PITG_15619	PITG_15619			1514	1.744	0.0993	No
74	PITG_16904	PITG_16904			1558	1.722	0.0993	No
75	PITG_16537	PITG_16537			1580	1.707	0.1000	No
76	PITG_17576	PITG_17576			1584	1.707	0.1014	No

77	PITG_01072	PITG_01072			1606	1.693	0.1021	No
78	PITG_04477	PITG_04477			1614	1.690	0.1034	No
79	PITG_23166	PITG_23166			1624	1.687	0.1046	No
80	PITG_02710	PITG_02710			1684	1.665	0.1038	No
81	PITG_23349	PITG_23349			1692	1.662	0.1051	No
82	PITG_09671	PITG_09671			1695	1.660	0.1065	No
83	PITG_12186	PITG_12186			1702	1.658	0.1077	No
84	PITG_17705	PITG_17705			1710	1.655	0.1090	No
85	PITG_20560	PITG_20560			1734	1.646	0.1096	No
86	PITG_15301	PITG_15301			1735	1.646	0.1110	No
87	PITG_20589	PITG_20589			1743	1.643	0.1123	No
88	PITG_10448	PITG_10448			1785	1.624	0.1122	No
89	PITG_11102	PITG_11102			1802	1.615	0.1130	No
90	PITG_03110	PITG_03110			1843	1.595	0.1129	No
91	PITG_07161	PITG_07161			1846	1.595	0.1143	No
92	PITG_05340	PITG_05340			1854	1.591	0.1154	No
93	PITG_22381	PITG_22381			1864	1.588	0.1165	No
94	PITG_15629	PITG_15629			1886	1.577	0.1172	No
95	PITG_01006	PITG_01006			1914	1.564	0.1175	No
96	PITG_06889	PITG_06889			1937	1.553	0.1181	No
97	PITG_08411	PITG_08411			1938	1.553	0.1195	No
98	PITG_17359	PITG_17359			1948	1.547	0.1205	No
99	PITG_12002	PITG_12002			1957	1.545	0.1216	No
100	PITG_17343	PITG_17343			1966	1.542	0.1227	No
101	PITG_05632	PITG_05632			1969	1.540	0.1240	No
102	PITG_08899	PITG_08899			2027	1.516	0.1232	No
103	PITG_03585	PITG_03585			2067	1.502	0.1231	No
104	PITG_07248	PITG_07248			2076	1.499	0.1241	No
105	PITG_20272	PITG_20272			2080	1.498	0.1254	No
106	PITG_08358	PITG_08358			2087	1.495	0.1265	No
107	PITG_10623	PITG_10623			2090	1.495	0.1277	No
108	PITG_07154	PITG_07154			2101	1.491	0.1287	No
109	PITG_20163	PITG_20163			2107	1.490	0.1299	No
110	PITG_05548	PITG_05548			2133	1.478	0.1302	No
111	PITG_03060	PITG_03060			2186	1.456	0.1296	No
112	PITG_15981	PITG_15981			2253	1.428	0.1284	No
113	PITG_21941	PITG_21941			2263	1.424	0.1293	No
114	PITG_16585	PITG_16585			2268	1.422	0.1304	No
115	PITG_09119	PITG_09119			2279	1.416	0.1313	No

116	PITG_17506	PITG_17506			2300	1.410	0.1318	No
117	PITG_03497	PITG_03497			2304	1.409	0.1330	No
118	PITG_15216	PITG_15216			2324	1.403	0.1335	No
119	PITG_00543	PITG_00543			2335	1.398	0.1344	No
120	PITG_09375	PITG_09375			2358	1.393	0.1348	No
121	PITG_06885	PITG_06885			2367	1.388	0.1358	No
122	PITG_08572	PITG_08572			2377	1.385	0.1367	No
123	PITG_21400	PITG_21400			2391	1.381	0.1374	No
124	PITG_16016	PITG_16016			2415	1.374	0.1378	No
125	PITG_21806	PITG_21806			2425	1.371	0.1387	No
126	PITG_10119	PITG_10119			2427	1.371	0.1399	No
127	PITG_02264	PITG_02264			2430	1.370	0.1410	No
128	PITG_18265	PITG_18265			2434	1.369	0.1421	No
129	PITG_17012	PITG_17012			2435	1.368	0.1434	No
130	PITG_00588	PITG_00588			2438	1.367	0.1445	No
131	PITG_08008	PITG_08008			2478	1.355	0.1443	No
132	PITG_10652	PITG_10652			2479	1.355	0.1455	No
133	PITG_11470	PITG_11470			2489	1.351	0.1463	No
134	PITG_03945	PITG_03945			2506	1.344	0.1469	No
135	PITG_01653	PITG_01653			2530	1.335	0.1473	No
136	PITG_02110	PITG_02110			2535	1.333	0.1483	No
137	PITG_12646	PITG_12646			2544	1.329	0.1492	No
138	PITG_14392	PITG_14392			2561	1.325	0.1498	No
139	PITG_05817	PITG_05817			2572	1.322	0.1506	No
140	PITG_10488	PITG_10488			2588	1.316	0.1512	No
141	PITG_12516	PITG_12516			2608	1.310	0.1517	No
142	PITG_10270	PITG_10270			2640	1.300	0.1517	No
143	PITG_14971	PITG_14971			2641	1.299	0.1528	No
144	PITG_14393	PITG_14393			2694	1.283	0.1520	No
145	PITG_00218	PITG_00218			2698	1.282	0.1531	No
146	PITG_05261	PITG_05261			2699	1.281	0.1542	No
147	PITG_11630	PITG_11630			2711	1.279	0.1549	No
148	PITG_18359	PITG_18359			2720	1.276	0.1558	No
149	PITG_04938	PITG_04938			2725	1.274	0.1568	No
150	PITG_05358	PITG_05358			2755	1.263	0.1568	No
151	PITG_08587	PITG_08587			2759	1.260	0.1578	No
152	PITG_19472	PITG_19472			2764	1.258	0.1588	No
153	PITG_06481	PITG_06481			2789	1.253	0.1590	No
154	PITG_03055	PITG_03055			2802	1.249	0.1597	No

155	PITG_04133	PITG_04133			2811	1.247	0.1605	No
156	PITG_15089	PITG_15089			2840	1.239	0.1606	No
157	PITG_21372	PITG_21372			2920	1.215	0.1587	No
158	PITG_09407	PITG_09407			2922	1.215	0.1597	No
159	PITG_19318	PITG_19318			2924	1.214	0.1608	No
160	PITG_03584	PITG_03584			2925	1.214	0.1619	No
161	PITG_03934	PITG_03934			2935	1.210	0.1626	No
162	PITG_04458	PITG_04458			2951	1.206	0.1631	No
163	PITG_22022	PITG_22022			2985	1.198	0.1629	No
164	PITG_06288	PITG_06288			3009	1.190	0.1631	No
165	PITG_07349	PITG_07349			3015	1.187	0.1640	No
166	Novel00922	Novel00922			3038	1.181	0.1642	No
167	PITG_19572	PITG_19572			3041	1.181	0.1652	No
168	PITG_05798	PITG_05798			3050	1.179	0.1660	No
169	PITG_17361	PITG_17361			3052	1.178	0.1670	No
170	PITG_11919	PITG_11919			3074	1.170	0.1673	No
171	PITG_16734	PITG_16734			3081	1.167	0.1681	No
172	PITG_05865	PITG_05865			3098	1.162	0.1685	No
173	PITG_15596	PITG_15596			3109	1.157	0.1692	No
174	PITG_19445	PITG_19445			3110	1.157	0.1702	No
175	PITG_23141	PITG_23141			3128	1.151	0.1706	No
176	PITG_18064	PITG_18064			3137	1.149	0.1713	No
177	PITG_02857	PITG_02857			3157	1.144	0.1716	No
178	PITG_19872	PITG_19872			3176	1.140	0.1720	No
179	PITG_19456	PITG_19456			3199	1.133	0.1722	No
180	PITG_04421	PITG_04421			3201	1.133	0.1731	No
181	PITG_08898	PITG_08898			3215	1.130	0.1737	No
182	PITG_20491	PITG_20491			3249	1.122	0.1734	No
183	PITG_03660	PITG_03660			3265	1.116	0.1739	No
184	PITG_18266	PITG_18266			3288	1.110	0.1740	No
185	PITG_10831	PITG_10831			3292	1.110	0.1749	No
186	PITG_01262	PITG_01262			3309	1.103	0.1753	No
187	PITG_00081	PITG_00081			3311	1.100	0.1762	No
188	PITG_02423	PITG_02423			3314	1.100	0.1772	No
189	PITG_08348	PITG_08348			3318	1.099	0.1780	No
190	PITG_11807	PITG_11807			3341	1.093	0.1782	No
191	PITG_07210	PITG_07210			3360	1.086	0.1785	No
192	PITG_17840	PITG_17840			3363	1.086	0.1794	No
193	PITG_08753	PITG_08753			3371	1.083	0.1801	No

194	PITG_06355	PITG_06355			3386	1.080	0.1805	No
195	PITG_11566	PITG_11566			3389	1.079	0.1814	No
196	PITG_06796	PITG_06796			3408	1.074	0.1817	No
197	PITG_00177	PITG_00177			3445	1.064	0.1813	No
198	PITG_01409	PITG_01409			3452	1.062	0.1820	No
199	PITG_06708	PITG_06708			3468	1.059	0.1824	No
200	PITG_12094	PITG_12094			3469	1.058	0.1833	No
201	PITG_20211	PITG_20211			3478	1.056	0.1840	No
202	PITG_09938	PITG_09938			3492	1.051	0.1844	No
203	PITG_04682	PITG_04682			3524	1.042	0.1842	No
204	PITG_02212	PITG_02212			3526	1.042	0.1851	No
205	PITG_08439	PITG_08439			3546	1.037	0.1853	No
206	PITG_04405	PITG_04405			3549	1.036	0.1862	No
207	PITG_13752	PITG_13752			3565	1.032	0.1865	No
208	PITG_07302	PITG_07302			3581	1.026	0.1869	No
209	PITG_06280	PITG_06280			3639	1.012	0.1856	No
210	PITG_13164	PITG_13164			3652	1.008	0.1861	No
211	PITG_02116	PITG_02116			3666	1.005	0.1865	No
212	PITG_02071	PITG_02071			3673	1.004	0.1872	No
213	PITG_04478	PITG_04478			3702	0.997	0.1870	No
214	PITG_07317	PITG_07317			3715	0.994	0.1874	No
215	PITG_04226	PITG_04226			3735	0.988	0.1876	No
216	PITG_07910	PITG_07910			3736	0.988	0.1885	No
217	PITG_18262	PITG_18262			3739	0.987	0.1893	No
218	PITG_18799	PITG_18799			3744	0.986	0.1900	No
219	PITG_17832	PITG_17832			3764	0.982	0.1902	No
220	PITG_02457	PITG_02457			3773	0.979	0.1908	No
221	PITG_12475	PITG_12475			3776	0.978	0.1916	No
222	PITG_17583	PITG_17583			3787	0.973	0.1921	No
223	PITG_02854	PITG_02854			3807	0.968	0.1922	No
224	PITG_11926	PITG_11926			3834	0.963	0.1921	No
225	PITG_12903	PITG_12903			3850	0.958	0.1924	No
226	PITG_07156	PITG_07156			3852	0.958	0.1932	No
227	PITG_04393	PITG_04393			3913	0.943	0.1918	No
228	PITG_04255	PITG_04255			3984	0.924	0.1900	No
229	PITG_15892	PITG_15892			4036	0.912	0.1888	No
230	PITG_00654	PITG_00654			4038	0.910	0.1896	No
231	PITG_06880	PITG_06880			4041	0.910	0.1904	No
232	PITG_07828	PITG_07828			4048	0.907	0.1910	No

233	PITG_09665	PITG_09665			4085	0.899	0.1904	No
234	PITG_23319	PITG_23319			4107	0.893	0.1904	No
235	PITG_09620	PITG_09620			4109	0.893	0.1912	No
236	PITG_10857	PITG_10857			4110	0.893	0.1920	No
237	PITG_07235	PITG_07235			4124	0.889	0.1923	No
238	PITG_12155	PITG_12155			4128	0.888	0.1930	No
239	PITG_15417	PITG_15417			4137	0.885	0.1934	No
240	PITG_11728	PITG_11728			4139	0.885	0.1942	No
241	PITG_21617	PITG_21617			4155	0.882	0.1944	No
242	PITG_00004	PITG_00004			4168	0.879	0.1948	No
243	PITG_06685	PITG_06685			4180	0.877	0.1951	No
244	PITG_00124	PITG_00124			4190	0.875	0.1956	No
245	PITG_20808	PITG_20808			4244	0.862	0.1943	No
246	PITG_12322	PITG_12322			4260	0.858	0.1945	No
247	PITG_14707	PITG_14707			4278	0.854	0.1947	No
248	PITG_03049	PITG_03049			4281	0.853	0.1954	No
249	PITG_04225	PITG_04225			4297	0.849	0.1956	No
250	PITG_00430	PITG_00430			4330	0.843	0.1951	No
251	PITG_01453	PITG_01453			4332	0.843	0.1958	No
252	PITG_00887	PITG_00887			4350	0.838	0.1959	No
253	PITG_11525	PITG_11525			4354	0.836	0.1966	No
254	PITG_04568	PITG_04568			4378	0.830	0.1964	No
255	PITG_06964	PITG_06964			4380	0.829	0.1971	No
256	PITG_07724	PITG_07724			4384	0.828	0.1978	No
257	PITG_17550	PITG_17550			4385	0.828	0.1985	No
258	PITG_01526	PITG_01526			4400	0.824	0.1987	No
259	PITG_01936	PITG_01936			4404	0.823	0.1994	No
260	PITG_10830	PITG_10830			4417	0.820	0.1996	No
261	PITG_17879	PITG_17879			4440	0.815	0.1995	No
262	PITG_10780	PITG_10780			4465	0.809	0.1993	No
263	PITG_03522	PITG_03522			4469	0.808	0.2000	No
264	PITG_16088	PITG_16088			4491	0.802	0.1999	No
265	PITG_02666	PITG_02666			4500	0.800	0.2003	No
266	PITG_03709	PITG_03709			4503	0.800	0.2009	No
267	PITG_22686	PITG_22686			4522	0.797	0.2010	No
268	PITG_11204	PITG_11204			4568	0.784	0.2000	No
269	PITG_04724	PITG_04724			4588	0.780	0.1999	No
270	PITG_18027	PITG_18027			4596	0.779	0.2004	No
271	PITG_23142	PITG_23142			4621	0.775	0.2002	No

272	PITG_02393	PITG_02393			4631	0.772	0.2005	No
273	PITG_17897	PITG_17897			4659	0.766	0.2002	No
274	PITG_05338	PITG_05338			4683	0.760	0.2000	No
275	PITG_02288	PITG_02288			4708	0.756	0.1998	No
276	PITG_22487	PITG_22487			4717	0.753	0.2001	No
277	PITG_14808	PITG_14808			4728	0.751	0.2004	No
278	PITG_03409	PITG_03409			4738	0.750	0.2008	No
279	PITG_10829	PITG_10829			4743	0.749	0.2013	No
280	PITG_18257	PITG_18257			4747	0.748	0.2018	No
281	PITG_18863	PITG_18863			4758	0.745	0.2021	No
282	PITG_12090	PITG_12090			4776	0.741	0.2022	No
283	PITG_17508	PITG_17508			4783	0.739	0.2026	No
284	PITG_21504	PITG_21504			4812	0.732	0.2022	No
285	PITG_07978	PITG_07978			4819	0.730	0.2026	No
286	PITG_22479	PITG_22479			4911	0.708	0.1998	No
287	PITG_04619	PITG_04619			4964	0.696	0.1985	No
288	PITG_18473	PITG_18473			5012	0.687	0.1973	No
289	PITG_00416	PITG_00416			5088	0.674	0.1950	No
290	PITG_16473	PITG_16473			5095	0.671	0.1954	No
291	PITG_01188	PITG_01188			5156	0.661	0.1937	No
292	PITG_13298	PITG_13298			5254	0.638	0.1906	No
293	PITG_17942	PITG_17942			5265	0.636	0.1908	No
294	PITG_13043	PITG_13043			5270	0.635	0.1912	No
295	PITG_20960	PITG_20960			5283	0.633	0.1913	No
296	PITG_01450	PITG_01450			5295	0.630	0.1915	No
297	PITG_08304	PITG_08304			5307	0.627	0.1916	No
298	PITG_10899	PITG_10899			5310	0.626	0.1921	No
299	PITG_07164	PITG_07164			5314	0.626	0.1926	No
300	PITG_05653	PITG_05653			5318	0.625	0.1930	No
301	PITG_17126	PITG_17126			5319	0.625	0.1936	No
302	PITG_00298	PITG_00298			5328	0.623	0.1938	No
303	PITG_18274	PITG_18274			5345	0.620	0.1938	No
304	PITG_06783	PITG_06783			5351	0.620	0.1942	No
305	PITG_02529	PITG_02529			5357	0.619	0.1945	No
306	PITG_08312	PITG_08312			5367	0.618	0.1947	No
307	PITG_17584	PITG_17584			5372	0.617	0.1951	No
308	PITG_06480	PITG_06480			5434	0.605	0.1934	No
309	PITG_10847	PITG_10847			5444	0.604	0.1936	No
310	PITG_07234	PITG_07234			5449	0.602	0.1940	No

311	PITG_19869	PITG_19869			5462	0.600	0.1940	No
312	PITG_08901	PITG_08901			5465	0.600	0.1945	No
313	PITG_01012	PITG_01012			5468	0.600	0.1950	No
314	PITG_01920	PITG_01920			5469	0.600	0.1955	No
315	PITG_15000	PITG_15000			5509	0.592	0.1946	No
316	PITG_00176	PITG_00176			5527	0.589	0.1944	No
317	PITG_02750	PITG_02750			5528	0.588	0.1950	No
318	PITG_19310	PITG_19310			5533	0.588	0.1954	No
319	PITG_18687	PITG_18687			5568	0.579	0.1946	No
320	PITG_14156	PITG_14156			5577	0.577	0.1948	No
321	PITG_10601	PITG_10601			5586	0.575	0.1950	No
322	PITG_12140	PITG_12140			5639	0.565	0.1935	No
323	PITG_00187	PITG_00187			5648	0.563	0.1937	No
324	PITG_07866	PITG_07866			5655	0.562	0.1940	No
325	PITG_02757	PITG_02757			5656	0.562	0.1945	No
326	PITG_06231	PITG_06231			5668	0.559	0.1946	No
327	PITG_01195	PITG_01195			5728	0.549	0.1929	No
328	PITG_05251	PITG_05251			5733	0.548	0.1932	No
329	PITG_08414	PITG_08414			5742	0.546	0.1934	No
330	PITG_06688	PITG_06688			5758	0.543	0.1933	No
331	PITG_08761	PITG_08761			5775	0.539	0.1932	No
332	PITG_10045	PITG_10045			5787	0.538	0.1933	No
333	PITG_16210	PITG_16210			5793	0.537	0.1936	No
334	PITG_08375	PITG_08375			5819	0.532	0.1931	No
335	PITG_19939	PITG_19939			5822	0.531	0.1935	No
336	PITG_16616	PITG_16616			5848	0.526	0.1930	No
337	PITG_08967	PITG_08967			5850	0.526	0.1934	No
338	PITG_12259	PITG_12259			5853	0.525	0.1938	No
339	PITG_03015	PITG_03015			5870	0.521	0.1937	No
340	PITG_01871	PITG_01871			5882	0.519	0.1937	No
341	PITG_20204	PITG_20204			5885	0.519	0.1941	No
342	PITG_15735	PITG_15735			5897	0.517	0.1942	No
343	PITG_08890	PITG_08890			5898	0.517	0.1946	No
344	PITG_17651	PITG_17651			5906	0.516	0.1948	No
345	PITG_17333	PITG_17333			5914	0.515	0.1950	No
346	PITG_20007	PITG_20007			5962	0.506	0.1937	No
347	PITG_11626	PITG_11626			5964	0.506	0.1941	No
348	PITG_02226	PITG_02226			6031	0.495	0.1921	No
349	PITG_19878	PITG_19878			6044	0.492	0.1921	No

350	PITG_19875	PITG_19875			6102	0.480	0.1903	No
351	PITG_01851	PITG_01851			6138	0.475	0.1894	No
352	PITG_07191	PITG_07191			6161	0.471	0.1890	No
353	PITG_06282	PITG_06282			6176	0.469	0.1889	No
354	PITG_16056	PITG_16056			6251	0.456	0.1865	No
355	PITG_00783	PITG_00783			6297	0.449	0.1852	No
356	PITG_09824	PITG_09824			6344	0.441	0.1838	No
357	PITG_16603	PITG_16603			6354	0.440	0.1839	No
358	PITG_22572	PITG_22572			6371	0.437	0.1837	No
359	PITG_12194	PITG_12194			6393	0.433	0.1833	No
360	PITG_16184	PITG_16184			6394	0.433	0.1837	No
361	PITG_07217	PITG_07217			6444	0.423	0.1822	No
362	PITG_15298	PITG_15298			6450	0.422	0.1824	No
363	PITG_12105	PITG_12105			6465	0.419	0.1822	No
364	PITG_15970	PITG_15970			6510	0.410	0.1809	No
365	PITG_16636	PITG_16636			6554	0.405	0.1796	No
366	PITG_03293	PITG_03293			6559	0.403	0.1799	No
367	PITG_01832	PITG_01832			6568	0.402	0.1799	No
368	PITG_11875	PITG_11875			6580	0.400	0.1799	No
369	PITG_10675	PITG_10675			6603	0.396	0.1794	No
370	PITG_03006	PITG_03006			6622	0.393	0.1791	No
371	PITG_00178	PITG_00178			6659	0.387	0.1780	No
372	PITG_22892	PITG_22892			6677	0.385	0.1777	No
373	PITG_05886	PITG_05886			6719	0.377	0.1765	No
374	PITG_18999	PITG_18999			6776	0.367	0.1747	No
375	PITG_06845	PITG_06845			6777	0.367	0.1750	No
376	PITG_17711	PITG_17711			6802	0.363	0.1745	No
377	PITG_05862	PITG_05862			6804	0.363	0.1748	No
378	PITG_03075	PITG_03075			6805	0.363	0.1751	No
379	PITG_07809	PITG_07809			6817	0.359	0.1750	No
380	PITG_19589	PITG_19589			6839	0.356	0.1745	No
381	PITG_05762	PITG_05762			6859	0.352	0.1741	No
382	PITG_18045	PITG_18045			6871	0.350	0.1740	No
383	PITG_03703	PITG_03703			6885	0.348	0.1738	No
384	PITG_06832	PITG_06832			6898	0.345	0.1737	No
385	PITG_00858	PITG_00858			6917	0.340	0.1733	No
386	PITG_07916	PITG_07916			6929	0.338	0.1732	No
387	PITG_10239	PITG_10239			6955	0.335	0.1725	No
388	PITG_14598	PITG_14598			6979	0.330	0.1720	No

389	PITG_23044	PITG_23044			6982	0.330	0.1722	No
390	PITG_20964	PITG_20964			6994	0.328	0.1721	No
391	PITG_13997	PITG_13997			7003	0.326	0.1720	No
392	PITG_23089	PITG_23089			7016	0.324	0.1719	No
393	PITG_02119	PITG_02119			7023	0.323	0.1719	No
394	PITG_05009	PITG_05009			7048	0.319	0.1713	No
395	PITG_18255	PITG_18255			7082	0.312	0.1703	No
396	PITG_15256	PITG_15256			7119	0.308	0.1693	No
397	PITG_06926	PITG_06926			7122	0.307	0.1695	No
398	PITG_03513	PITG_03513			7126	0.307	0.1696	No
399	PITG_19932	PITG_19932			7130	0.307	0.1698	No
400	PITG_18347	PITG_18347			7138	0.306	0.1698	No
401	PITG_02446	PITG_02446			7145	0.305	0.1698	No
402	PITG_06775	PITG_06775			7166	0.304	0.1693	No
403	PITG_21607	PITG_21607			7181	0.302	0.1691	No
404	PITG_21621	PITG_21621			7196	0.300	0.1688	No
405	PITG_17295	PITG_17295			7199	0.300	0.1690	No
406	PITG_06942	PITG_06942			7257	0.288	0.1671	No
407	PITG_22715	PITG_22715			7265	0.287	0.1671	No
408	PITG_01043	PITG_01043			7284	0.283	0.1667	No
409	PITG_15457	PITG_15457			7299	0.281	0.1664	No
410	PITG_07737	PITG_07737			7305	0.279	0.1665	No
411	PITG_17165	PITG_17165			7346	0.274	0.1652	No
412	PITG_05238	PITG_05238			7350	0.273	0.1653	No
413	PITG_05240	PITG_05240			7362	0.272	0.1651	No
414	PITG_20760	PITG_20760			7367	0.270	0.1652	No
415	PITG_17314	PITG_17314			7410	0.262	0.1639	No
416	PITG_06279	PITG_06279			7424	0.261	0.1636	No
417	PITG_00194	PITG_00194			7427	0.260	0.1638	No
418	PITG_18279	PITG_18279			7436	0.259	0.1637	No
419	PITG_15003	PITG_15003			7471	0.254	0.1626	No
420	PITG_06738	PITG_06738			7493	0.250	0.1621	No
421	PITG_22264	PITG_22264			7496	0.250	0.1622	No
422	PITG_02992	PITG_02992			7548	0.243	0.1605	No
423	PITG_08957	PITG_08957			7565	0.240	0.1601	No
424	PITG_14994	PITG_14994			7594	0.237	0.1593	No
425	PITG_16446	PITG_16446			7616	0.233	0.1587	No
426	PITG_05781	PITG_05781			7620	0.232	0.1588	No
427	PITG_03093	PITG_03093			7622	0.232	0.1589	No

428	PITG_06259	PITG_06259			7625	0.232	0.1591	No
429	PITG_11431	PITG_11431			7635	0.231	0.1589	No
430	PITG_17990	PITG_17990			7649	0.229	0.1586	No
431	PITG_06199	PITG_06199			7654	0.228	0.1587	No
432	PITG_16644	PITG_16644			7659	0.228	0.1588	No
433	PITG_11798	PITG_11798			7669	0.226	0.1586	No
434	PITG_00221	PITG_00221			7679	0.224	0.1585	No
435	PITG_17292	PITG_17292			7685	0.223	0.1585	No
436	PITG_02407	PITG_02407			7690	0.221	0.1585	No
437	PITG_09251	PITG_09251			7698	0.221	0.1585	No
438	PITG_13415	PITG_13415			7701	0.220	0.1586	No
439	PITG_10032	PITG_10032			7703	0.220	0.1587	No
440	PITG_16280	PITG_16280			7708	0.220	0.1588	No
441	PITG_00115	PITG_00115			7710	0.220	0.1589	No
442	PITG_10516	PITG_10516			7711	0.219	0.1591	No
443	PITG_17509	PITG_17509			7729	0.217	0.1587	No
444	PITG_07652	PITG_07652			7756	0.212	0.1579	No
445	PITG_16646	PITG_16646			7772	0.209	0.1575	No
446	PITG_13074	PITG_13074			7794	0.205	0.1569	No
447	PITG_13301	PITG_13301			7795	0.205	0.1571	No
448	PITG_08810	PITG_08810			7804	0.204	0.1570	No
449	PITG_02213	PITG_02213			7809	0.204	0.1570	No
450	PITG_02711	PITG_02711			7895	0.194	0.1539	No
451	PITG_16618	PITG_16618			7898	0.194	0.1540	No
452	PITG_12037	PITG_12037			7907	0.193	0.1539	No
453	PITG_01848	PITG_01848			7931	0.189	0.1532	No
454	PITG_03456	PITG_03456			7938	0.188	0.1532	No
455	PITG_02724	PITG_02724			7957	0.186	0.1526	No
456	PITG_02546	PITG_02546			7962	0.186	0.1527	No
457	PITG_18258	PITG_18258			7971	0.184	0.1525	No
458	PITG_20600	PITG_20600			7975	0.184	0.1526	No
459	PITG_09402	PITG_09402			7984	0.183	0.1524	No
460	PITG_06724	PITG_06724			7999	0.181	0.1521	No
461	PITG_07160	PITG_07160			8014	0.179	0.1517	No
462	PITG_02489	PITG_02489			8030	0.177	0.1513	No
463	PITG_07731	PITG_07731			8047	0.175	0.1508	No
464	PITG_11126	PITG_11126			8053	0.175	0.1508	No
465	PITG_01950	PITG_01950			8055	0.175	0.1509	No
466	PITG_19450	PITG_19450			8115	0.169	0.1488	No

467	PITG_02211	PITG_02211			8116	0.169	0.1490	No
468	PITG_17153	PITG_17153			8119	0.169	0.1491	No
469	PITG_00570	PITG_00570			8143	0.166	0.1483	No
470	PITG_16977	PITG_16977			8146	0.165	0.1484	No
471	PITG_06607	PITG_06607			8158	0.163	0.1481	No
472	PITG_07656	PITG_07656			8181	0.159	0.1474	No
473	PITG_08736	PITG_08736			8197	0.157	0.1470	No
474	PITG_20746	PITG_20746			8201	0.157	0.1470	No
475	PITG_21189	PITG_21189			8210	0.155	0.1469	No
476	PITG_14920	PITG_14920			8217	0.154	0.1468	No
477	PITG_04715	PITG_04715			8219	0.154	0.1469	No
478	PITG_11999	PITG_11999			8250	0.149	0.1459	No
479	PITG_22582	PITG_22582			8269	0.146	0.1453	No
480	PITG_02291	PITG_02291			8306	0.141	0.1441	No
481	PITG_16461	PITG_16461			8310	0.141	0.1441	No
482	PITG_09260	PITG_09260			8316	0.140	0.1440	No
483	PITG_02400	PITG_02400			8344	0.137	0.1431	No
484	PITG_00248	PITG_00248			8348	0.136	0.1431	No
485	PITG_16276	PITG_16276			8357	0.134	0.1430	No
486	PITG_13648	PITG_13648			8389	0.130	0.1419	No
487	PITG_03738	PITG_03738			8391	0.130	0.1420	No
488	PITG_01480	PITG_01480			8403	0.128	0.1417	No
489	PITG_09039	PITG_09039			8431	0.122	0.1408	No
490	PITG_12517	PITG_12517			8458	0.118	0.1399	No
491	PITG_00319	PITG_00319			8474	0.117	0.1394	No
492	PITG_08760	PITG_08760			8476	0.116	0.1395	No
493	PITG_08570	PITG_08570			8492	0.114	0.1390	No
494	PITG_06774	PITG_06774			8519	0.111	0.1381	No
495	PITG_08004	PITG_08004			8525	0.110	0.1380	No
496	PITG_18261	PITG_18261			8533	0.109	0.1379	No
497	PITG_15382	PITG_15382			8558	0.105	0.1370	No
498	PITG_19905	PITG_19905			8575	0.103	0.1365	No
499	PITG_16440	PITG_16440			8602	0.100	0.1356	No
500	PITG_02904	PITG_02904			8604	0.100	0.1357	No
501	PITG_07031	PITG_07031			8632	0.096	0.1347	No
502	PITG_04665	PITG_04665			8634	0.096	0.1348	No
503	PITG_18275	PITG_18275			8639	0.095	0.1347	No
504	PITG_14137	PITG_14137			8645	0.094	0.1346	No
505	PITG_19429	PITG_19429			8646	0.094	0.1347	No

506	PITG_22684	PITG_22684			8652	0.092	0.1346	No
507	PITG_06107	PITG_06107			8664	0.090	0.1343	No
508	PITG_17663	PITG_17663			8670	0.090	0.1342	No
509	PITG_13564	PITG_13564			8672	0.089	0.1342	No
510	PITG_20562	PITG_20562			8680	0.087	0.1340	No
511	PITG_06928	PITG_06928			8723	0.080	0.1325	No
512	PITG_19773	PITG_19773			8726	0.080	0.1325	No
513	PITG_01856	PITG_01856			8754	0.076	0.1315	No
514	PITG_11486	PITG_11486			8755	0.076	0.1316	No
515	PITG_21185	PITG_21185			8766	0.074	0.1313	No
516	PITG_00238	PITG_00238			8795	0.070	0.1303	No
517	PITG_18649	PITG_18649			8837	0.063	0.1288	No
518	PITG_03700	PITG_03700			8871	0.057	0.1276	No
519	PITG_02442	PITG_02442			8943	0.044	0.1249	No
520	PITG_01343	PITG_01343			8959	0.041	0.1244	No
521	PITG_09508	PITG_09508			8996	0.036	0.1231	No
522	PITG_07278	PITG_07278			9052	0.028	0.1210	No
523	PITG_09394	PITG_09394			9068	0.027	0.1204	No
524	PITG_07539	PITG_07539			9080	0.026	0.1200	No
525	PITG_11100	PITG_11100			9081	0.026	0.1201	No
526	PITG_03634	PITG_03634			9084	0.026	0.1200	No
527	PITG_11273	PITG_11273			9125	0.021	0.1185	No
528	PITG_14699	PITG_14699			9188	0.012	0.1162	No
529	PITG_13130	PITG_13130			9201	0.009	0.1157	No
530	PITG_08042	PITG_08042			9232	0.005	0.1146	No
531	PITG_09118	PITG_09118			9314	0.000	0.1115	No
532	PITG_20640	PITG_20640			9457	0.000	0.1061	No
533	PITG_12509	PITG_12509			9496	0.000	0.1047	No
534	PITG_18980	PITG_18980			9822	0.000	0.0923	No
535	PITG_07481	PITG_07481			9908	0.000	0.0891	No
536	PITG_21243	PITG_21243			9933	0.000	0.0882	No
537	PITG_12629	PITG_12629			9991	0.000	0.0860	No
538	PITG_18292	PITG_18292			10159	0.000	0.0797	No
539	PITG_19346	PITG_19346			10561	0.000	0.0645	No
540	PITG_22310	PITG_22310			10562	0.000	0.0645	No
541	PITG_23338	PITG_23338			11284	0.000	0.0371	No
542	PITG_07182	PITG_07182			11352	0.000	0.0345	No
543	PITG_21582	PITG_21582			11683	0.000	0.0220	No
544	PITG_21586	PITG_21586			11684	0.000	0.0220	No

545	PITG_20824	PITG_20824			11824	0.000	0.0167	No	
546	PITG_10610	PITG_10610			12049	0.000	0.0082	No	
547	PITG_20161	PITG_20161			12303	0.000	-0.0014	No	
548	PITG_15722	PITG_15722			12398	0.000	-0.0050	No	
549	PITG_22058	PITG_22058			12412	0.000	-0.0055	No	
550	PITG_10666	PITG_10666			12660	0.000	-0.0148	No	
551	PITG_10100	PITG_10100			12663	0.000	-0.0149	No	
552	PITG_01091	PITG_01091			13039	0.000	-0.0292	No	
553	PITG_05812	PITG_05812			13417	0.000	-0.0435	No	
554	PITG_01017	PITG_01017			13430	0.000	-0.0439	No	
555	PITG_20131	PITG_20131			13455	0.000	-0.0448	No	
556	PITG_09431	PITG_09431			13717	0.000	-0.0548	No	
557	PITG_18701	PITG_18701			13814	0.000	-0.0584	No	
558	PITG_08191	PITG_08191			13861	0.000	-0.0601	No	
559	PITG_07214	PITG_07214			13894	0.000	-0.0614	No	
560	PITG_01013	PITG_01013			13905	0.000	-0.0617	No	
561	PITG_01016	PITG_01016			13907	0.000	-0.0618	No	
562	PITG_17187	PITG_17187			14156	0.000	-0.0712	No	
563	PITG_06873	PITG_06873			14206	0.000	-0.0731	No	
564	PITG_19379	PITG_19379			14298	0.000	-0.0765	No	
565	PITG_19374	PITG_19374			14299	0.000	-0.0765	No	
566	Novel00393	Novel00393			14641	0.000	-0.0895	No	
567	PITG_22488	PITG_22488			14855	0.000	-0.0976	No	
568	PITG_05162	PITG_05162			15031	0.000	-0.1042	No	
569	PITG_21202	PITG_21202			15178	0.000	-0.1098	No	
570	PITG_14310	PITG_14310			15340	0.000	-0.1159	No	
571	PITG_14312	PITG_14312			15341	0.000	-0.1159	No	
572	PITG_14315	PITG_14315			15343	0.000	-0.1159	No	
573	PITG_04365	PITG_04365			15636	0.000	-0.1270	No	
574	PITG_16530	PITG_16530			15706	0.000	-0.1296	No	
575	PITG_21989	PITG_21989			15819	0.000	-0.1339	No	
576	PITG_20587	PITG_20587			15830	0.000	-0.1342	No	
577	PITG_02702	PITG_02702			15892	0.000	-0.1366	No	
578	PITG_01007	PITG_01007			16195	0.000	-0.1480	No	
579	PITG_06326	PITG_06326			16282	0.000	-0.1513	No	
580	PITG_16601	PITG_16601			16284	0.000	-0.1513	No	
581	PITG_14346	PITG_14346			16494	0.000	-0.1593	No	
582	PITG_14344	PITG_14344			16495	0.000	-0.1593	No	
583	PITG_10111	PITG_10111			16497	0.000	-0.1593	No	

584	PITG_20943	PITG_20943			16614	0.000	-0.1637	No
585	PITG_18225	PITG_18225			16859	0.000	-0.1730	No
586	PITG_18226	PITG_18226			16860	0.000	-0.1730	No
587	PITG_19256	PITG_19256			17102	0.000	-0.1821	No
588	PITG_05803	PITG_05803			17175	0.000	-0.1849	No
589	PITG_22629	PITG_22629			17194	0.000	-0.1856	No
590	PITG_21979	PITG_21979			17341	0.000	-0.1911	No
591	PITG_14322	PITG_14322			17578	0.000	-0.2001	No
592	PITG_20240	PITG_20240			17604	0.000	-0.2010	No
593	PITG_21148	PITG_21148			17848	0.000	-0.2102	No
594	PITG_15474	PITG_15474			17924	0.000	-0.2131	No
595	PITG_12459	PITG_12459			17988	0.000	-0.2155	No
596	Novel01184	Novel01184			18053	0.000	-0.2179	No
597	PITG_03806	PITG_03806			18089	0.000	-0.2192	No
598	PITG_03807	PITG_03807			18090	0.000	-0.2192	No
599	PITG_07548	PITG_07548			18291	0.000	-0.2268	No
600	PITG_05850	PITG_05850			18333	0.000	-0.2284	No
601	PITG_03730	PITG_03730			18437	0.000	-0.2323	No
602	PITG_03731	PITG_03731			18438	0.000	-0.2323	No
603	PITG_22374	PITG_22374			18442	0.000	-0.2324	No
604	PITG_19463	PITG_19463			18465	0.000	-0.2333	No
605	PITG_04498	PITG_04498			18503	0.000	-0.2347	No
606	PITG_18553	PITG_18553			18597	0.000	-0.2382	No
607	PITG_20405	PITG_20405			18630	0.000	-0.2394	No
608	PITG_04594	PITG_04594			18776	0.000	-0.2449	No
609	PITG_11927	PITG_11927			18817	0.000	-0.2464	No
610	PITG_21079	PITG_21079			18849	0.000	-0.2476	No
611	PITG_15977	PITG_15977			18871	0.000	-0.2484	No
612	PITG_07643	PITG_07643			19054	0.000	-0.2553	No
613	PITG_14325	PITG_14325			19076	0.000	-0.2561	No
614	PITG_21615	PITG_21615			19197	0.000	-0.2607	No
615	PITG_13638	PITG_13638			19265	0.000	-0.2632	No
616	PITG_05086	PITG_05086			19377	0.000	-0.2674	No
617	PITG_20103	PITG_20103			19412	-0.003	-0.2687	No
618	PITG_21395	PITG_21395			19416	-0.004	-0.2688	No
619	PITG_04474	PITG_04474			19423	-0.006	-0.2691	No
620	PITG_17926	PITG_17926			19440	-0.007	-0.2697	No
621	PITG_12293	PITG_12293			19449	-0.008	-0.2700	No
622	PITG_09010	PITG_09010			19459	-0.011	-0.2703	No

623	PITG_09706	PITG_09706			19489	-0.014	-0.2714	No
624	PITG_10193	PITG_10193			19492	-0.015	-0.2714	No
625	PITG_10003	PITG_10003			19511	-0.016	-0.2721	No
626	PITG_02762	PITG_02762			19550	-0.021	-0.2735	No
627	PITG_03150	PITG_03150			19553	-0.021	-0.2736	No
628	PITG_10760	PITG_10760			19601	-0.027	-0.2754	No
629	PITG_16214	PITG_16214			19641	-0.031	-0.2768	No
630	PITG_11304	PITG_11304			19656	-0.033	-0.2773	No
631	PITG_13172	PITG_13172			19672	-0.037	-0.2778	No
632	PITG_19459	PITG_19459			19677	-0.038	-0.2780	No
633	PITG_11793	PITG_11793			19692	-0.040	-0.2785	No
634	PITG_09092	PITG_09092			19694	-0.040	-0.2785	No
635	PITG_04254	PITG_04254			19700	-0.041	-0.2786	No
636	PITG_07851	PITG_07851			19709	-0.042	-0.2789	No
637	PITG_13042	PITG_13042			19712	-0.043	-0.2789	No
638	PITG_22801	PITG_22801			19727	-0.044	-0.2794	No
639	PITG_19148	PITG_19148			19741	-0.046	-0.2799	No
640	PITG_18303	PITG_18303			19751	-0.048	-0.2802	No
641	PITG_09791	PITG_09791			19763	-0.050	-0.2805	No
642	PITG_08876	PITG_08876			19771	-0.051	-0.2808	No
643	PITG_10778	PITG_10778			19776	-0.052	-0.2809	No
644	PITG_21673	PITG_21673			19781	-0.054	-0.2810	No
645	PITG_22427	PITG_22427			19791	-0.056	-0.2813	No
646	PITG_02401	PITG_02401			19798	-0.056	-0.2814	No
647	PITG_15982	PITG_15982			19802	-0.057	-0.2815	No
648	PITG_19294	PITG_19294			19848	-0.064	-0.2831	No
649	PITG_06015	PITG_06015			19886	-0.071	-0.2845	No
650	PITG_11900	PITG_11900			19901	-0.073	-0.2850	No
651	PITG_00331	PITG_00331			19921	-0.077	-0.2856	No
652	PITG_06223	PITG_06223			19927	-0.077	-0.2857	No
653	PITG_02077	PITG_02077			19961	-0.084	-0.2869	No
654	PITG_16604	PITG_16604			19976	-0.086	-0.2874	No
655	PITG_00296	PITG_00296			19977	-0.086	-0.2873	No
656	PITG_00208	PITG_00208			19978	-0.086	-0.2872	No
657	Novel01760	Novel01760			19995	-0.088	-0.2877	No
658	PITG_07670	PITG_07670			20005	-0.090	-0.2880	No
659	PITG_12041	PITG_12041			20014	-0.091	-0.2882	No
660	PITG_03480	PITG_03480			20045	-0.095	-0.2893	No
661	PITG_10008	PITG_10008			20083	-0.100	-0.2906	No

662	PITG_18129	PITG_18129			20089	-0.102	-0.2907	No
663	PITG_21349	PITG_21349			20105	-0.104	-0.2912	No
664	PITG_01389	PITG_01389			20161	-0.113	-0.2931	No
665	PITG_07165	PITG_07165			20178	-0.114	-0.2937	No
666	PITG_12540	PITG_12540			20191	-0.117	-0.2940	No
667	PITG_01203	PITG_01203			20227	-0.122	-0.2952	No
668	PITG_20156	PITG_20156			20241	-0.123	-0.2956	No
669	PITG_13641	PITG_13641			20247	-0.123	-0.2957	No
670	PITG_00254	PITG_00254			20282	-0.129	-0.2969	No
671	PITG_08599	PITG_08599			20297	-0.132	-0.2973	No
672	PITG_09666	PITG_09666			20334	-0.138	-0.2985	No
673	PITG_12514	PITG_12514			20340	-0.139	-0.2986	No
674	PITG_07055	PITG_07055			20354	-0.141	-0.2989	No
675	PITG_17586	PITG_17586			20356	-0.142	-0.2989	No
676	PITG_09846	PITG_09846			20357	-0.142	-0.2987	No
677	PITG_01255	PITG_01255			20360	-0.142	-0.2987	No
678	PITG_05587	PITG_05587			20363	-0.143	-0.2986	No
679	PITG_14557	PITG_14557			20366	-0.143	-0.2986	No
680	PITG_10645	PITG_10645			20370	-0.145	-0.2986	No
681	PITG_16203	PITG_16203			20372	-0.145	-0.2985	No
682	PITG_23114	PITG_23114			20375	-0.145	-0.2984	No
683	PITG_10513	PITG_10513			20400	-0.149	-0.2992	No
684	PITG_22989	PITG_22989			20406	-0.149	-0.2992	No
685	PITG_05483	PITG_05483			20415	-0.151	-0.2994	No
686	PITG_04506	PITG_04506			20429	-0.153	-0.2998	No
687	PITG_04747	PITG_04747			20459	-0.156	-0.3007	No
688	PITG_13669	PITG_13669			20463	-0.157	-0.3007	No
689	PITG_14992	PITG_14992			20468	-0.158	-0.3007	No
690	PITG_07549	PITG_07549			20475	-0.159	-0.3008	No
691	PITG_06195	PITG_06195			20493	-0.161	-0.3013	No
692	PITG_09393	PITG_09393			20582	-0.174	-0.3045	No
693	PITG_00633	PITG_00633			20587	-0.175	-0.3045	No
694	PITG_21223	PITG_21223			20593	-0.176	-0.3045	No
695	PITG_08806	PITG_08806			20614	-0.178	-0.3051	No
696	PITG_16741	PITG_16741			20622	-0.179	-0.3052	No
697	PITG_03681	PITG_03681			20625	-0.180	-0.3051	No
698	PITG_07149	PITG_07149			20626	-0.180	-0.3050	No
699	PITG_13991	PITG_13991			20651	-0.186	-0.3057	No
700	PITG_00757	PITG_00757			20661	-0.186	-0.3059	No

701	PITG_13913	PITG_13913			20702	-0.193	-0.3072	No
702	PITG_04729	PITG_04729			20710	-0.194	-0.3073	No
703	PITG_18276	PITG_18276			20741	-0.197	-0.3083	No
704	PITG_01260	PITG_01260			20816	-0.208	-0.3109	No
705	PITG_00077	PITG_00077			20886	-0.218	-0.3133	No
706	PITG_06022	PITG_06022			20893	-0.220	-0.3134	No
707	PITG_02493	PITG_02493			20900	-0.221	-0.3134	No
708	PITG_18296	PITG_18296			20914	-0.224	-0.3137	No
709	PITG_01580	PITG_01580			20920	-0.225	-0.3137	No
710	PITG_04677	PITG_04677			20921	-0.226	-0.3135	No
711	PITG_07995	PITG_07995			20928	-0.227	-0.3135	No
712	PITG_15774	PITG_15774			20933	-0.228	-0.3134	No
713	PITG_04457	PITG_04457			20934	-0.228	-0.3132	No
714	PITG_04774	PITG_04774			20937	-0.229	-0.3131	No
715	PITG_10077	PITG_10077			20981	-0.236	-0.3145	No
716	PITG_11236	PITG_11236			20985	-0.237	-0.3144	No
717	PITG_20584	PITG_20584			21004	-0.241	-0.3149	No
718	PITG_17703	PITG_17703			21014	-0.242	-0.3150	No
719	PITG_05730	PITG_05730			21018	-0.243	-0.3149	No
720	PITG_10317	PITG_10317			21019	-0.243	-0.3147	No
721	PITG_09101	PITG_09101			21047	-0.249	-0.3155	No
722	PITG_08900	PITG_08900			21059	-0.251	-0.3157	No
723	PITG_20772	PITG_20772			21060	-0.251	-0.3155	No
724	PITG_03220	PITG_03220			21071	-0.254	-0.3156	No
725	PITG_02561	PITG_02561			21072	-0.255	-0.3154	No
726	PITG_08210	PITG_08210			21095	-0.257	-0.3160	No
727	PITG_19849	PITG_19849			21100	-0.258	-0.3159	No
728	PITG_03901	PITG_03901			21126	-0.261	-0.3166	No
729	PITG_05112	PITG_05112			21178	-0.270	-0.3183	No
730	PITG_08802	PITG_08802			21211	-0.274	-0.3193	No
731	PITG_07535	PITG_07535			21219	-0.276	-0.3193	No
732	PITG_06518	PITG_06518			21226	-0.277	-0.3193	No
733	PITG_03415	PITG_03415			21230	-0.278	-0.3191	No
734	PITG_01193	PITG_01193			21260	-0.285	-0.3200	No
735	PITG_04611	PITG_04611			21271	-0.288	-0.3201	No
736	PITG_01142	PITG_01142			21273	-0.288	-0.3199	No
737	PITG_16360	PITG_16360			21275	-0.288	-0.3197	No
738	PITG_16057	PITG_16057			21307	-0.293	-0.3206	No
739	PITG_03276	PITG_03276			21320	-0.296	-0.3208	No

740	PITG_07153	PITG_07153			21332	-0.299	-0.3209	No
741	PITG_07354	PITG_07354			21368	-0.304	-0.3220	No
742	PITG_17664	PITG_17664			21382	-0.306	-0.3222	No
743	PITG_10821	PITG_10821			21386	-0.307	-0.3220	No
744	PITG_05853	PITG_05853			21389	-0.307	-0.3218	No
745	PITG_04344	PITG_04344			21391	-0.308	-0.3216	No
746	PITG_13724	PITG_13724			21399	-0.310	-0.3216	No
747	PITG_13421	PITG_13421			21402	-0.310	-0.3214	No
748	PITG_12077	PITG_12077			21406	-0.311	-0.3212	No
749	PITG_11734	PITG_11734			21411	-0.312	-0.3211	No
750	PITG_16856	PITG_16856			21416	-0.313	-0.3210	No
751	PITG_12489	PITG_12489			21431	-0.315	-0.3212	No
752	PITG_14970	PITG_14970			21432	-0.315	-0.3209	No
753	PITG_11910	PITG_11910			21468	-0.322	-0.3220	No
754	PITG_11733	PITG_11733			21492	-0.325	-0.3225	No
755	PITG_06118	PITG_06118			21499	-0.326	-0.3225	No
756	PITG_17791	PITG_17791			21534	-0.332	-0.3235	No
757	PITG_01002	PITG_01002			21556	-0.335	-0.3240	No
758	PITG_17133	PITG_17133			21566	-0.336	-0.3240	No
759	PITG_05487	PITG_05487			21571	-0.337	-0.3239	No
760	PITG_01447	PITG_01447			21582	-0.340	-0.3239	No
761	PITG_13347	PITG_13347			21592	-0.341	-0.3240	No
762	PITG_06817	PITG_06817			21611	-0.345	-0.3243	No
763	PITG_06979	PITG_06979			21659	-0.353	-0.3258	No
764	PITG_18277	PITG_18277			21660	-0.353	-0.3255	No
765	PITG_02474	PITG_02474			21661	-0.353	-0.3252	No
766	PITG_08368	PITG_08368			21662	-0.354	-0.3249	No
767	PITG_08014	PITG_08014			21696	-0.360	-0.3258	No
768	PITG_12151	PITG_12151			21701	-0.361	-0.3256	No
769	PITG_01235	PITG_01235			21702	-0.361	-0.3253	No
770	PITG_16807	PITG_16807			21711	-0.362	-0.3253	No
771	PITG_12160	PITG_12160			21750	-0.370	-0.3264	No
772	PITG_16328	PITG_16328			21756	-0.372	-0.3262	No
773	PITG_12181	PITG_12181			21763	-0.373	-0.3261	No
774	PITG_06749	PITG_06749			21769	-0.375	-0.3260	No
775	PITG_10519	PITG_10519			21779	-0.377	-0.3260	No
776	PITG_10092	PITG_10092			21790	-0.379	-0.3260	No
777	PITG_02114	PITG_02114			21801	-0.381	-0.3261	No
778	PITG_02707	PITG_02707			21802	-0.381	-0.3257	No

779	PITG_00407	PITG_00407			21809	-0.382	-0.3256	No
780	PITG_14850	PITG_14850			21815	-0.384	-0.3254	No
781	PITG_13148	PITG_13148			21820	-0.385	-0.3252	No
782	PITG_16757	PITG_16757			21821	-0.385	-0.3249	No
783	PITG_15603	PITG_15603			21844	-0.390	-0.3254	No
784	PITG_05245	PITG_05245			21857	-0.392	-0.3255	No
785	PITG_05318	PITG_05318			21858	-0.392	-0.3251	No
786	PITG_06701	PITG_06701			21872	-0.394	-0.3253	No
787	PITG_03643	PITG_03643			21876	-0.395	-0.3250	No
788	PITG_14918	PITG_14918			21886	-0.397	-0.3250	No
789	Novel01790	Novel01790			21888	-0.397	-0.3247	No
790	PITG_12099	PITG_12099			21909	-0.401	-0.3251	No
791	PITG_19364	PITG_19364			21911	-0.402	-0.3248	No
792	PITG_07201	PITG_07201			21935	-0.406	-0.3253	No
793	PITG_01087	PITG_01087			21950	-0.410	-0.3254	No
794	PITG_16055	PITG_16055			21951	-0.410	-0.3251	No
795	PITG_09596	PITG_09596			21965	-0.412	-0.3252	No
796	PITG_19235	PITG_19235			21967	-0.412	-0.3249	No
797	PITG_07797	PITG_07797			21968	-0.412	-0.3245	No
798	PITG_00395	PITG_00395			22013	-0.420	-0.3258	No
799	PITG_03799	PITG_03799			22019	-0.421	-0.3256	No
800	PITG_00279	PITG_00279			22020	-0.421	-0.3252	No
801	PITG_04610	PITG_04610			22066	-0.429	-0.3266	No
802	PITG_05405	PITG_05405			22072	-0.429	-0.3264	No
803	PITG_05733	PITG_05733			22091	-0.432	-0.3267	No
804	PITG_14835	PITG_14835			22092	-0.432	-0.3263	No
805	PITG_01777	PITG_01777			22104	-0.435	-0.3263	No
806	PITG_03860	PITG_03860			22108	-0.435	-0.3260	No
807	PITG_16137	PITG_16137			22114	-0.436	-0.3258	No
808	PITG_00172	PITG_00172			22118	-0.437	-0.3255	No
809	PITG_08669	PITG_08669			22119	-0.437	-0.3251	No
810	PITG_19041	PITG_19041			22120	-0.437	-0.3247	No
811	PITG_23274	PITG_23274			22122	-0.437	-0.3244	No
812	PITG_08452	PITG_08452			22132	-0.439	-0.3243	No
813	PITG_15569	PITG_15569			22135	-0.440	-0.3240	No
814	PITG_15890	PITG_15890			22149	-0.442	-0.3241	No
815	PITG_20188	PITG_20188			22157	-0.445	-0.3240	No
816	PITG_19488	PITG_19488			22158	-0.445	-0.3236	No
817	PITG_18420	PITG_18420			22180	-0.447	-0.3240	No

818	PITG_10929	PITG_10929			22185	-0.448	-0.3237	No
819	PITG_05920	PITG_05920			22186	-0.448	-0.3233	No
820	PITG_14228	PITG_14228			22217	-0.454	-0.3240	No
821	PITG_00477	PITG_00477			22253	-0.459	-0.3250	No
822	PITG_17495	PITG_17495			22322	-0.473	-0.3271	No
823	PITG_12122	PITG_12122			22333	-0.477	-0.3271	No
824	PITG_03364	PITG_03364			22349	-0.480	-0.3272	No
825	PITG_00464	PITG_00464			22388	-0.489	-0.3282	No
826	PITG_03617	PITG_03617			22395	-0.490	-0.3280	No
827	PITG_08425	PITG_08425			22454	-0.500	-0.3298	No
828	PITG_03056	PITG_03056			22459	-0.502	-0.3295	No
829	PITG_08197	PITG_08197			22480	-0.506	-0.3298	No
830	PITG_15294	PITG_15294			22488	-0.507	-0.3296	No
831	PITG_05007	PITG_05007			22498	-0.509	-0.3295	No
832	PITG_19669	PITG_19669			22503	-0.510	-0.3291	No
833	PITG_12839	PITG_12839			22518	-0.514	-0.3292	No
834	PITG_10877	PITG_10877			22533	-0.518	-0.3293	No
835	PITG_20084	PITG_20084			22551	-0.523	-0.3295	No
836	PITG_15776	PITG_15776			22592	-0.531	-0.3305	No
837	PITG_13680	PITG_13680			22596	-0.531	-0.3301	No
838	PITG_06505	PITG_06505			22598	-0.532	-0.3297	No
839	PITG_01290	PITG_01290			22600	-0.532	-0.3293	No
840	PITG_07830	PITG_07830			22601	-0.532	-0.3288	No
841	PITG_02394	PITG_02394			22610	-0.535	-0.3286	No
842	PITG_10932	PITG_10932			22626	-0.540	-0.3287	No
843	PITG_06174	PITG_06174			22631	-0.541	-0.3283	No
844	PITG_06191	PITG_06191			22653	-0.546	-0.3287	No
845	PITG_17599	PITG_17599			22655	-0.546	-0.3282	No
846	PITG_06222	PITG_06222			22748	-0.566	-0.3312	No
847	PITG_04207	PITG_04207			22767	-0.568	-0.3314	Yes
848	PITG_06776	PITG_06776			22768	-0.569	-0.3308	Yes
849	PITG_17251	PITG_17251			22780	-0.574	-0.3307	Yes
850	PITG_07242	PITG_07242			22787	-0.575	-0.3305	Yes
851	PITG_12692	PITG_12692			22807	-0.578	-0.3307	Yes
852	PITG_10979	PITG_10979			22822	-0.583	-0.3307	Yes
853	PITG_01296	PITG_01296			22829	-0.584	-0.3304	Yes
854	PITG_12482	PITG_12482			22831	-0.585	-0.3299	Yes
855	PITG_19535	PITG_19535			22839	-0.585	-0.3296	Yes
856	PITG_02925	PITG_02925			22842	-0.586	-0.3292	Yes

857	PITG_17410	PITG_17410			22846	-0.587	-0.3288	Yes
858	PITG_04589	PITG_04589			22853	-0.589	-0.3285	Yes
859	PITG_19999	PITG_19999			22878	-0.594	-0.3288	Yes
860	PITG_13832	PITG_13832			22885	-0.595	-0.3285	Yes
861	PITG_00257	PITG_00257			22922	-0.600	-0.3294	Yes
862	PITG_04918	PITG_04918			22927	-0.602	-0.3290	Yes
863	PITG_10447	PITG_10447			22931	-0.603	-0.3285	Yes
864	PITG_00443	PITG_00443			22936	-0.604	-0.3281	Yes
865	PITG_12993	PITG_12993			22944	-0.606	-0.3279	Yes
866	PITG_17578	PITG_17578			22973	-0.613	-0.3284	Yes
867	PITG_05354	PITG_05354			22976	-0.613	-0.3279	Yes
868	PITG_14497	PITG_14497			23012	-0.624	-0.3287	Yes
869	PITG_11142	PITG_11142			23021	-0.625	-0.3284	Yes
870	PITG_11603	PITG_11603			23046	-0.632	-0.3288	Yes
871	PITG_13671	PITG_13671			23051	-0.632	-0.3283	Yes
872	PITG_17575	PITG_17575			23061	-0.634	-0.3281	Yes
873	PITG_08756	PITG_08756			23063	-0.634	-0.3276	Yes
874	PITG_07022	PITG_07022			23069	-0.635	-0.3272	Yes
875	PITG_06175	PITG_06175			23070	-0.635	-0.3266	Yes
876	PITG_10998	PITG_10998			23114	-0.645	-0.3277	Yes
877	PITG_17502	PITG_17502			23120	-0.646	-0.3273	Yes
878	PITG_20769	PITG_20769			23130	-0.650	-0.3270	Yes
879	PITG_02921	PITG_02921			23131	-0.650	-0.3265	Yes
880	PITG_02191	PITG_02191			23149	-0.653	-0.3265	Yes
881	PITG_17356	PITG_17356			23155	-0.654	-0.3261	Yes
882	PITG_09640	PITG_09640			23185	-0.660	-0.3266	Yes
883	PITG_03498	PITG_03498			23191	-0.661	-0.3262	Yes
884	PITG_12961	PITG_12961			23203	-0.663	-0.3260	Yes
885	PITG_19158	PITG_19158			23247	-0.673	-0.3271	Yes
886	PITG_13315	PITG_13315			23265	-0.677	-0.3271	Yes
887	PITG_12745	PITG_12745			23280	-0.681	-0.3270	Yes
888	PITG_08471	PITG_08471			23281	-0.681	-0.3264	Yes
889	PITG_08888	PITG_08888			23309	-0.686	-0.3268	Yes
890	PITG_10450	PITG_10450			23331	-0.691	-0.3270	Yes
891	PITG_01804	PITG_01804			23342	-0.693	-0.3268	Yes
892	PITG_14609	PITG_14609			23351	-0.695	-0.3264	Yes
893	PITG_08155	PITG_08155			23354	-0.695	-0.3259	Yes
894	PITG_02580	PITG_02580			23358	-0.695	-0.3254	Yes
895	PITG_01862	PITG_01862			23364	-0.696	-0.3249	Yes

896	PITG_02294	PITG_02294			23416	-0.708	-0.3262	Yes
897	PITG_10953	PITG_10953			23460	-0.720	-0.3272	Yes
898	PITG_11111	PITG_11111			23461	-0.720	-0.3266	Yes
899	PITG_05521	PITG_05521			23481	-0.725	-0.3267	Yes
900	PITG_15090	PITG_15090			23482	-0.725	-0.3260	Yes
901	PITG_15850	PITG_15850			23491	-0.727	-0.3256	Yes
902	PITG_15307	PITG_15307			23514	-0.734	-0.3258	Yes
903	PITG_04843	PITG_04843			23523	-0.735	-0.3255	Yes
904	PITG_10049	PITG_10049			23526	-0.736	-0.3249	Yes
905	PITG_02136	PITG_02136			23534	-0.738	-0.3245	Yes
906	PITG_16008	PITG_16008			23545	-0.740	-0.3242	Yes
907	PITG_01314	PITG_01314			23548	-0.741	-0.3236	Yes
908	PITG_02124	PITG_02124			23587	-0.752	-0.3244	Yes
909	PITG_22662	PITG_22662			23589	-0.752	-0.3237	Yes
910	PITG_09698	PITG_09698			23607	-0.757	-0.3237	Yes
911	PITG_01036	PITG_01036			23631	-0.764	-0.3239	Yes
912	PITG_17748	PITG_17748			23641	-0.765	-0.3235	Yes
913	PITG_03077	PITG_03077			23643	-0.765	-0.3229	Yes
914	PITG_13735	PITG_13735			23644	-0.766	-0.3222	Yes
915	PITG_11752	PITG_11752			23691	-0.778	-0.3232	Yes
916	PITG_02565	PITG_02565			23697	-0.780	-0.3227	Yes
917	PITG_10147	PITG_10147			23705	-0.783	-0.3223	Yes
918	PITG_18259	PITG_18259			23713	-0.785	-0.3219	Yes
919	PITG_06927	PITG_06927			23726	-0.789	-0.3216	Yes
920	PITG_06771	PITG_06771			23729	-0.789	-0.3210	Yes
921	PITG_03754	PITG_03754			23730	-0.790	-0.3203	Yes
922	PITG_12588	PITG_12588			23738	-0.792	-0.3198	Yes
923	PITG_17574	PITG_17574			23751	-0.796	-0.3196	Yes
924	PITG_08369	PITG_08369			23772	-0.802	-0.3196	Yes
925	PITG_13512	PITG_13512			23778	-0.804	-0.3191	Yes
926	PITG_10887	PITG_10887			23788	-0.807	-0.3187	Yes
927	PITG_16671	PITG_16671			23803	-0.811	-0.3185	Yes
928	PITG_18067	PITG_18067			23816	-0.815	-0.3182	Yes
929	PITG_07274	PITG_07274			23833	-0.819	-0.3181	Yes
930	PITG_03813	PITG_03813			23834	-0.819	-0.3173	Yes
931	PITG_20189	PITG_20189			23835	-0.820	-0.3166	Yes
932	PITG_03420	PITG_03420			23870	-0.829	-0.3171	Yes
933	PITG_07967	PITG_07967			23906	-0.840	-0.3177	Yes
934	PITG_05374	PITG_05374			23918	-0.844	-0.3174	Yes

935	PITG_02182	PITG_02182			23932	-0.848	-0.3171	Yes
936	PITG_15723	PITG_15723			23962	-0.858	-0.3174	Yes
937	PITG_00525	PITG_00525			23993	-0.866	-0.3178	Yes
938	PITG_07968	PITG_07968			24006	-0.870	-0.3175	Yes
939	PITG_20747	PITG_20747			24009	-0.871	-0.3168	Yes
940	PITG_01922	PITG_01922			24019	-0.874	-0.3163	Yes
941	PITG_21313	PITG_21313			24020	-0.874	-0.3155	Yes
942	PITG_12916	PITG_12916			24029	-0.878	-0.3150	Yes
943	PITG_09550	PITG_09550			24034	-0.880	-0.3144	Yes
944	PITG_16213	PITG_16213			24078	-0.893	-0.3152	Yes
945	PITG_12699	PITG_12699			24133	-0.909	-0.3165	Yes
946	PITG_08001	PITG_08001			24134	-0.909	-0.3157	Yes
947	PITG_10974	PITG_10974			24137	-0.910	-0.3149	Yes
948	PITG_10080	PITG_10080			24151	-0.913	-0.3146	Yes
949	PITG_14001	PITG_14001			24176	-0.921	-0.3147	Yes
950	PITG_09547	PITG_09547			24182	-0.924	-0.3140	Yes
951	PITG_15817	PITG_15817			24196	-0.927	-0.3137	Yes
952	PITG_12864	PITG_12864			24223	-0.936	-0.3138	Yes
953	PITG_04703	PITG_04703			24269	-0.949	-0.3147	Yes
954	PITG_02867	PITG_02867			24296	-0.957	-0.3148	Yes
955	PITG_21071	PITG_21071			24300	-0.958	-0.3141	Yes
956	PITG_14972	PITG_14972			24302	-0.958	-0.3132	Yes
957	PITG_00005	PITG_00005			24303	-0.959	-0.3124	Yes
958	PITG_18298	PITG_18298			24307	-0.960	-0.3116	Yes
959	PITG_18271	PITG_18271			24322	-0.966	-0.3113	Yes
960	PITG_09870	PITG_09870			24327	-0.969	-0.3106	Yes
961	PITG_02694	PITG_02694			24328	-0.970	-0.3097	Yes
962	PITG_03856	PITG_03856			24341	-0.973	-0.3093	Yes
963	PITG_13458	PITG_13458			24352	-0.976	-0.3088	Yes
964	PITG_01445	PITG_01445			24362	-0.980	-0.3082	Yes
965	PITG_04487	PITG_04487			24365	-0.980	-0.3074	Yes
966	PITG_05649	PITG_05649			24366	-0.980	-0.3066	Yes
967	PITG_01943	PITG_01943			24378	-0.982	-0.3061	Yes
968	PITG_04838	PITG_04838			24399	-0.987	-0.3060	Yes
969	PITG_06379	PITG_06379			24403	-0.989	-0.3052	Yes
970	PITG_11923	PITG_11923			24405	-0.990	-0.3043	Yes
971	PITG_02082	PITG_02082			24424	-0.997	-0.3041	Yes
972	PITG_04678	PITG_04678			24425	-0.997	-0.3032	Yes
973	PITG_06639	PITG_06639			24452	-1.003	-0.3033	Yes

974	PITG_08984	PITG_08984			24460	-1.006	-0.3027	Yes
975	PITG_07028	PITG_07028			24510	-1.023	-0.3036	Yes
976	PITG_03274	PITG_03274			24545	-1.036	-0.3040	Yes
977	PITG_03221	PITG_03221			24557	-1.040	-0.3035	Yes
978	PITG_10334	PITG_10334			24566	-1.042	-0.3028	Yes
979	PITG_09635	PITG_09635			24568	-1.043	-0.3019	Yes
980	PITG_12050	PITG_12050			24576	-1.046	-0.3013	Yes
981	PITG_06274	PITG_06274			24587	-1.048	-0.3007	Yes
982	PITG_10139	PITG_10139			24603	-1.053	-0.3003	Yes
983	PITG_02700	PITG_02700			24612	-1.055	-0.2997	Yes
984	PITG_10777	PITG_10777			24618	-1.057	-0.2989	Yes
985	PITG_11909	PITG_11909			24630	-1.061	-0.2984	Yes
986	PITG_11766	PITG_11766			24642	-1.064	-0.2978	Yes
987	PITG_13488	PITG_13488			24643	-1.064	-0.2969	Yes
988	PITG_17945	PITG_17945			24644	-1.066	-0.2959	Yes
989	PITG_11615	PITG_11615			24652	-1.068	-0.2952	Yes
990	PITG_07841	PITG_07841			24668	-1.073	-0.2948	Yes
991	PITG_17252	PITG_17252			24669	-1.073	-0.2939	Yes
992	PITG_15015	PITG_15015			24677	-1.075	-0.2932	Yes
993	PITG_18278	PITG_18278			24697	-1.082	-0.2929	Yes
994	PITG_04698	PITG_04698			24698	-1.082	-0.2919	Yes
995	PITG_00688	PITG_00688			24703	-1.083	-0.2911	Yes
996	PITG_13586	PITG_13586			24704	-1.083	-0.2901	Yes
997	PITG_00754	PITG_00754			24709	-1.085	-0.2893	Yes
998	PITG_17501	PITG_17501			24726	-1.092	-0.2889	Yes
999	PITG_03322	PITG_03322			24740	-1.096	-0.2884	Yes
1000	PITG_14463	PITG_14463			24743	-1.097	-0.2875	Yes
1001	PITG_04034	PITG_04034			24744	-1.098	-0.2865	Yes
1002	PITG_08157	PITG_08157			24750	-1.102	-0.2857	Yes
1003	PITG_09576	PITG_09576			24756	-1.103	-0.2849	Yes
1004	PITG_06995	PITG_06995			24760	-1.104	-0.2841	Yes
1005	PITG_01833	PITG_01833			24767	-1.106	-0.2833	Yes
1006	PITG_13437	PITG_13437			24778	-1.109	-0.2827	Yes
1007	PITG_11253	PITG_11253			24792	-1.115	-0.2822	Yes
1008	PITG_13079	PITG_13079			24810	-1.121	-0.2818	Yes
1009	PITG_00997	PITG_00997			24825	-1.125	-0.2813	Yes
1010	PITG_01576	PITG_01576			24876	-1.142	-0.2822	Yes
1011	PITG_14380	PITG_14380			24886	-1.146	-0.2815	Yes
1012	PITG_01762	PITG_01762			24911	-1.153	-0.2814	Yes

1013	PITG_19213	PITG_19213			24913	-1.154	-0.2804	Yes
1014	PITG_05171	PITG_05171			24921	-1.158	-0.2796	Yes
1015	PITG_00566	PITG_00566			24953	-1.171	-0.2797	Yes
1016	PITG_04348	PITG_04348			24997	-1.189	-0.2803	Yes
1017	PITG_06016	PITG_06016			25004	-1.191	-0.2795	Yes
1018	PITG_22249	PITG_22249			25013	-1.195	-0.2787	Yes
1019	PITG_15069	PITG_15069			25019	-1.198	-0.2778	Yes
1020	PITG_11178	PITG_11178			25025	-1.201	-0.2769	Yes
1021	PITG_16366	PITG_16366			25055	-1.210	-0.2769	Yes
1022	PITG_00941	PITG_00941			25067	-1.214	-0.2763	Yes
1023	PITG_07888	PITG_07888			25072	-1.216	-0.2753	Yes
1024	PITG_09664	PITG_09664			25091	-1.223	-0.2749	Yes
1025	PITG_11569	PITG_11569			25112	-1.232	-0.2745	Yes
1026	PITG_18251	PITG_18251			25141	-1.242	-0.2745	Yes
1027	PITG_12697	PITG_12697			25164	-1.249	-0.2742	Yes
1028	PITG_13371	PITG_13371			25168	-1.249	-0.2732	Yes
1029	PITG_15407	PITG_15407			25203	-1.263	-0.2733	Yes
1030	PITG_08000	PITG_08000			25216	-1.268	-0.2727	Yes
1031	PITG_03698	PITG_03698			25237	-1.274	-0.2723	Yes
1032	PITG_19557	PITG_19557			25245	-1.278	-0.2714	Yes
1033	PITG_03999	PITG_03999			25257	-1.283	-0.2707	Yes
1034	PITG_14765	PITG_14765			25258	-1.283	-0.2695	Yes
1035	PITG_05174	PITG_05174			25267	-1.288	-0.2687	Yes
1036	PITG_17390	PITG_17390			25287	-1.297	-0.2682	Yes
1037	PITG_03294	PITG_03294			25303	-1.306	-0.2676	Yes
1038	PITG_08703	PITG_08703			25308	-1.308	-0.2666	Yes
1039	PITG_07173	PITG_07173			25342	-1.325	-0.2666	Yes
1040	PITG_09699	PITG_09699			25358	-1.334	-0.2660	Yes
1041	PITG_09553	PITG_09553			25359	-1.334	-0.2648	Yes
1042	PITG_13655	PITG_13655			25367	-1.336	-0.2639	Yes
1043	PITG_03672	PITG_03672			25370	-1.338	-0.2627	Yes
1044	PITG_06937	PITG_06937			25372	-1.339	-0.2616	Yes
1045	PITG_14913	PITG_14913			25405	-1.356	-0.2616	Yes
1046	PITG_13069	PITG_13069			25408	-1.358	-0.2604	Yes
1047	PITG_18052	PITG_18052			25424	-1.365	-0.2598	Yes
1048	PITG_02672	PITG_02672			25452	-1.378	-0.2596	Yes
1049	PITG_08959	PITG_08959			25458	-1.380	-0.2585	Yes
1050	PITG_03235	PITG_03235			25480	-1.389	-0.2581	Yes
1051	PITG_08206	PITG_08206			25481	-1.390	-0.2568	Yes

1052	PITG_14626	PITG_14626			25482	-1.390	-0.2556	Yes
1053	PITG_03661	PITG_03661			25487	-1.391	-0.2545	Yes
1054	PITG_04382	PITG_04382			25503	-1.398	-0.2538	Yes
1055	PITG_02397	PITG_02397			25510	-1.400	-0.2527	Yes
1056	PITG_09582	PITG_09582			25513	-1.402	-0.2515	Yes
1057	PITG_22959	PITG_22959			25525	-1.409	-0.2507	Yes
1058	PITG_14729	PITG_14729			25528	-1.410	-0.2495	Yes
1059	PITG_03416	PITG_03416			25532	-1.416	-0.2483	Yes
1060	PITG_13397	PITG_13397			25533	-1.417	-0.2471	Yes
1061	PITG_07141	PITG_07141			25545	-1.420	-0.2462	Yes
1062	PITG_09506	PITG_09506			25563	-1.431	-0.2456	Yes
1063	PITG_04418	PITG_04418			25564	-1.431	-0.2443	Yes
1064	PITG_08579	PITG_08579			25572	-1.438	-0.2433	Yes
1065	PITG_19399	PITG_19399			25621	-1.462	-0.2438	Yes
1066	PITG_14195	PITG_14195			25624	-1.463	-0.2425	Yes
1067	PITG_00643	PITG_00643			25627	-1.464	-0.2413	Yes
1068	PITG_05851	PITG_05851			25628	-1.465	-0.2400	Yes
1069	PITG_04992	PITG_04992			25670	-1.484	-0.2402	Yes
1070	PITG_18272	PITG_18272			25674	-1.486	-0.2390	Yes
1071	PITG_10146	PITG_10146			25685	-1.491	-0.2380	Yes
1072	PITG_09540	PITG_09540			25692	-1.493	-0.2369	Yes
1073	PITG_00523	PITG_00523			25696	-1.497	-0.2357	Yes
1074	PITG_06595	PITG_06595			25709	-1.500	-0.2348	Yes
1075	PITG_09234	PITG_09234			25712	-1.502	-0.2335	Yes
1076	PITG_10863	PITG_10863			25713	-1.502	-0.2321	Yes
1077	PITG_07405	PITG_07405			25728	-1.511	-0.2313	Yes
1078	PITG_12764	PITG_12764			25729	-1.511	-0.2300	Yes
1079	PITG_00073	PITG_00073			25737	-1.514	-0.2289	Yes
1080	PITG_20965	PITG_20965			25743	-1.516	-0.2277	Yes
1081	PITG_17500	PITG_17500			25747	-1.518	-0.2264	Yes
1082	PITG_06286	PITG_06286			25751	-1.520	-0.2252	Yes
1083	PITG_06237	PITG_06237			25757	-1.523	-0.2240	Yes
1084	PITG_08808	PITG_08808			25759	-1.524	-0.2227	Yes
1085	PITG_04922	PITG_04922			25773	-1.531	-0.2218	Yes
1086	PITG_17785	PITG_17785			25774	-1.532	-0.2204	Yes
1087	PITG_03353	PITG_03353			25780	-1.535	-0.2192	Yes
1088	PITG_12930	PITG_12930			25785	-1.538	-0.2180	Yes
1089	PITG_13860	PITG_13860			25786	-1.539	-0.2166	Yes
1090	PITG_07300	PITG_07300			25805	-1.550	-0.2159	Yes

1091	PITG_17512	PITG_17512			25821	-1.557	-0.2151	Yes
1092	PITG_20264	PITG_20264			25831	-1.561	-0.2140	Yes
1093	PITG_08968	PITG_08968			25847	-1.568	-0.2132	Yes
1094	PITG_07671	PITG_07671			25857	-1.574	-0.2121	Yes
1095	PITG_00302	PITG_00302			25872	-1.579	-0.2112	Yes
1096	PITG_04522	PITG_04522			25877	-1.580	-0.2099	Yes
1097	PITG_01769	PITG_01769			25892	-1.589	-0.2090	Yes
1098	PITG_16069	PITG_16069			25898	-1.591	-0.2078	Yes
1099	PITG_17579	PITG_17579			25904	-1.594	-0.2065	Yes
1100	PITG_16339	PITG_16339			25905	-1.596	-0.2051	Yes
1101	PITG_05523	PITG_05523			25916	-1.604	-0.2040	Yes
1102	PITG_06019	PITG_06019			25918	-1.604	-0.2026	Yes
1103	PITG_22112	PITG_22112			25925	-1.611	-0.2014	Yes
1104	PITG_20771	PITG_20771			25935	-1.619	-0.2003	Yes
1105	Novel00015	Novel00015			25949	-1.627	-0.1993	Yes
1106	PITG_14456	PITG_14456			25952	-1.628	-0.1980	Yes
1107	PITG_04683	PITG_04683			25968	-1.637	-0.1970	Yes
1108	PITG_04419	PITG_04419			25974	-1.640	-0.1958	Yes
1109	PITG_12947	PITG_12947			26003	-1.658	-0.1953	Yes
1110	PITG_03775	PITG_03775			26025	-1.671	-0.1946	Yes
1111	PITG_03239	PITG_03239			26044	-1.683	-0.1938	Yes
1112	PITG_16074	PITG_16074			26057	-1.695	-0.1927	Yes
1113	PITG_17572	PITG_17572			26059	-1.697	-0.1912	Yes
1114	PITG_02080	PITG_02080			26061	-1.698	-0.1898	Yes
1115	PITG_10062	PITG_10062			26063	-1.699	-0.1883	Yes
1116	PITG_02578	PITG_02578			26070	-1.705	-0.1870	Yes
1117	PITG_19531	PITG_19531			26082	-1.713	-0.1858	Yes
1118	PITG_21501	PITG_21501			26089	-1.718	-0.1845	Yes
1119	PITG_03460	PITG_03460			26094	-1.719	-0.1831	Yes
1120	PITG_03178	PITG_03178			26113	-1.732	-0.1823	Yes
1121	PITG_19428	PITG_19428			26114	-1.733	-0.1807	Yes
1122	PITG_01245	PITG_01245			26115	-1.733	-0.1791	Yes
1123	PITG_18053	PITG_18053			26117	-1.734	-0.1776	Yes
1124	PITG_15777	PITG_15777			26137	-1.749	-0.1768	Yes
1125	PITG_02721	PITG_02721			26145	-1.755	-0.1755	Yes
1126	PITG_08553	PITG_08553			26153	-1.762	-0.1741	Yes
1127	PITG_14639	PITG_14639			26157	-1.764	-0.1727	Yes
1128	PITG_17778	PITG_17778			26181	-1.779	-0.1719	Yes
1129	PITG_13831	PITG_13831			26183	-1.780	-0.1704	Yes

1130	PITG_18073	PITG_18073			26188	-1.784	-0.1689	Yes
1131	PITG_08129	PITG_08129			26202	-1.791	-0.1678	Yes
1132	PITG_20559	PITG_20559			26204	-1.791	-0.1662	Yes
1133	PITG_13513	PITG_13513			26216	-1.803	-0.1650	Yes
1134	PITG_07792	PITG_07792			26226	-1.805	-0.1637	Yes
1135	PITG_09619	PITG_09619			26227	-1.806	-0.1621	Yes
1136	PITG_00910	PITG_00910			26229	-1.807	-0.1605	Yes
1137	PITG_21854	PITG_21854			26230	-1.809	-0.1589	Yes
1138	PITG_13681	PITG_13681			26238	-1.816	-0.1575	Yes
1139	PITG_17573	PITG_17573			26245	-1.817	-0.1561	Yes
1140	PITG_04910	PITG_04910			26259	-1.827	-0.1550	Yes
1141	PITG_06821	PITG_06821			26267	-1.833	-0.1536	Yes
1142	PITG_09552	PITG_09552			26270	-1.835	-0.1520	Yes
1143	PITG_19676	PITG_19676			26289	-1.845	-0.1510	Yes
1144	PITG_02704	PITG_02704			26295	-1.847	-0.1496	Yes
1145	PITG_19157	PITG_19157			26301	-1.850	-0.1481	Yes
1146	PITG_06850	PITG_06850			26312	-1.856	-0.1468	Yes
1147	PITG_02392	PITG_02392			26315	-1.857	-0.1452	Yes
1148	PITG_08002	PITG_08002			26321	-1.864	-0.1437	Yes
1149	PITG_01694	PITG_01694			26354	-1.889	-0.1433	Yes
1150	PITG_12300	PITG_12300			26358	-1.891	-0.1417	Yes
1151	PITG_06636	PITG_06636			26370	-1.898	-0.1404	Yes
1152	PITG_09631	PITG_09631			26373	-1.899	-0.1387	Yes
1153	PITG_01042	PITG_01042			26385	-1.915	-0.1374	Yes
1154	PITG_00632	PITG_00632			26390	-1.921	-0.1359	Yes
1155	PITG_09726	PITG_09726			26397	-1.925	-0.1344	Yes
1156	PITG_00397	PITG_00397			26402	-1.929	-0.1328	Yes
1157	PITG_00132	PITG_00132			26411	-1.939	-0.1313	Yes
1158	PITG_10089	PITG_10089			26420	-1.946	-0.1299	Yes
1159	PITG_09555	PITG_09555			26429	-1.950	-0.1284	Yes
1160	PITG_23109	PITG_23109			26441	-1.970	-0.1271	Yes
1161	PITG_03598	PITG_03598			26463	-1.987	-0.1261	Yes
1162	PITG_15526	PITG_15526			26465	-1.988	-0.1243	Yes
1163	PITG_15611	PITG_15611			26470	-1.993	-0.1227	Yes
1164	PITG_13924	PITG_13924			26471	-1.993	-0.1209	Yes
1165	PITG_00074	PITG_00074			26475	-1.996	-0.1192	Yes
1166	PITG_13399	PITG_13399			26492	-2.007	-0.1180	Yes
1167	PITG_01849	PITG_01849			26505	-2.015	-0.1167	Yes
1168	PITG_09521	PITG_09521			26522	-2.027	-0.1155	Yes

1169	PITG_07991	PITG_07991			26527	-2.030	-0.1138	Yes
1170	PITG_00756	PITG_00756			26581	-2.077	-0.1139	Yes
1171	PITG_17300	PITG_17300			26597	-2.085	-0.1126	Yes
1172	PITG_05377	PITG_05377			26618	-2.106	-0.1115	Yes
1173	PITG_01271	PITG_01271			26631	-2.118	-0.1100	Yes
1174	PITG_17357	PITG_17357			26669	-2.154	-0.1095	Yes
1175	PITG_00952	PITG_00952			26682	-2.164	-0.1080	Yes
1176	PITG_13420	PITG_13420			26720	-2.197	-0.1075	Yes
1177	PITG_02039	PITG_02039			26754	-2.231	-0.1067	Yes
1178	PITG_02429	PITG_02429			26766	-2.248	-0.1051	Yes
1179	PITG_03768	PITG_03768			26780	-2.261	-0.1036	Yes
1180	PITG_17024	PITG_17024			26795	-2.275	-0.1020	Yes
1181	PITG_00761	PITG_00761			26796	-2.276	-0.1000	Yes
1182	PITG_14936	PITG_14936			26810	-2.306	-0.0984	Yes
1183	PITG_00571	PITG_00571			26814	-2.307	-0.0965	Yes
1184	PITG_17947	PITG_17947			26821	-2.313	-0.0946	Yes
1185	PITG_07725	PITG_07725			26832	-2.319	-0.0929	Yes
1186	PITG_14993	PITG_14993			26835	-2.324	-0.0909	Yes
1187	PITG_22529	PITG_22529			26856	-2.345	-0.0895	Yes
1188	PITG_06596	PITG_06596			26861	-2.351	-0.0876	Yes
1189	PITG_16736	PITG_16736			26862	-2.351	-0.0855	Yes
1190	PITG_18054	PITG_18054			26868	-2.362	-0.0835	Yes
1191	PITG_00661	PITG_00661			26878	-2.372	-0.0817	Yes
1192	PITG_15616	PITG_15616			26885	-2.377	-0.0798	Yes
1193	PITG_09851	PITG_09851			26916	-2.412	-0.0788	Yes
1194	PITG_00276	PITG_00276			26923	-2.419	-0.0768	Yes
1195	PITG_00999	PITG_00999			26925	-2.422	-0.0747	Yes
1196	PITG_02708	PITG_02708			26929	-2.427	-0.0726	Yes
1197	PITG_00646	PITG_00646			26941	-2.448	-0.0709	Yes
1198	PITG_02858	PITG_02858			26966	-2.472	-0.0695	Yes
1199	PITG_17607	PITG_17607			26969	-2.477	-0.0674	Yes
1200	PITG_06021	PITG_06021			26996	-2.511	-0.0661	Yes
1201	PITG_17289	PITG_17289			26999	-2.513	-0.0639	Yes
1202	PITG_18545	PITG_18545			27014	-2.541	-0.0622	Yes
1203	PITG_22020	PITG_22020			27018	-2.545	-0.0600	Yes
1204	PITG_03414	PITG_03414			27020	-2.547	-0.0578	Yes
1205	PITG_13732	PITG_13732			27029	-2.559	-0.0558	Yes
1206	PITG_14413	PITG_14413			27031	-2.568	-0.0535	Yes
1207	PITG_14969	PITG_14969			27033	-2.568	-0.0512	Yes

1208	PITG_17580	PITG_17580			27045	-2.593	-0.0493	Yes
1209	PITG_03493	PITG_03493			27048	-2.596	-0.0470	Yes
1210	PITG_17582	PITG_17582			27085	-2.652	-0.0460	Yes
1211	PITG_21299	PITG_21299			27100	-2.678	-0.0441	Yes
1212	PITG_18270	PITG_18270			27151	-2.759	-0.0436	Yes
1213	PITG_21661	PITG_21661			27161	-2.778	-0.0414	Yes
1214	PITG_21456	PITG_21456			27188	-2.833	-0.0398	Yes
1215	PITG_08714	PITG_08714			27196	-2.846	-0.0376	Yes
1216	PITG_00471	PITG_00471			27228	-2.898	-0.0361	Yes
1217	PITG_10110	PITG_10110			27232	-2.902	-0.0336	Yes
1218	PITG_13636	PITG_13636			27247	-2.935	-0.0315	Yes
1219	PITG_03773	PITG_03773			27268	-2.976	-0.0296	Yes
1220	PITG_21623	PITG_21623			27271	-2.978	-0.0270	Yes
1221	PITG_11116	PITG_11116			27364	-3.219	-0.0276	Yes
1222	PITG_01062	PITG_01062			27369	-3.233	-0.0248	Yes
1223	PITG_01824	PITG_01824			27371	-3.234	-0.0220	Yes
1224	PITG_17507	PITG_17507			27373	-3.240	-0.0191	Yes
1225	PITG_21127	PITG_21127			27388	-3.276	-0.0167	Yes
1226	PITG_14968	PITG_14968			27392	-3.283	-0.0138	Yes
1227	PITG_17571	PITG_17571			27429	-3.407	-0.0121	Yes
1228	PITG_17831	PITG_17831			27501	-3.680	-0.0115	Yes
1229	PITG_14352	PITG_14352			27505	-3.705	-0.0083	Yes
1230	PITG_19993	PITG_19993			27511	-3.727	-0.0052	Yes
1231	PITG_02621	PITG_02621			27522	-3.845	-0.0021	Yes
1232	PITG_00691	PITG_00691			27533	-3.913	0.0011	Yes

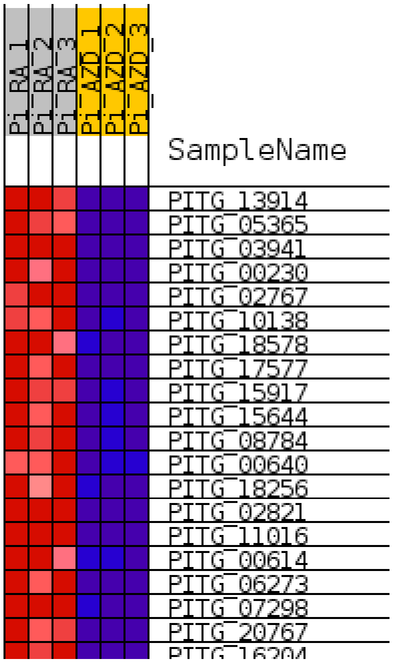

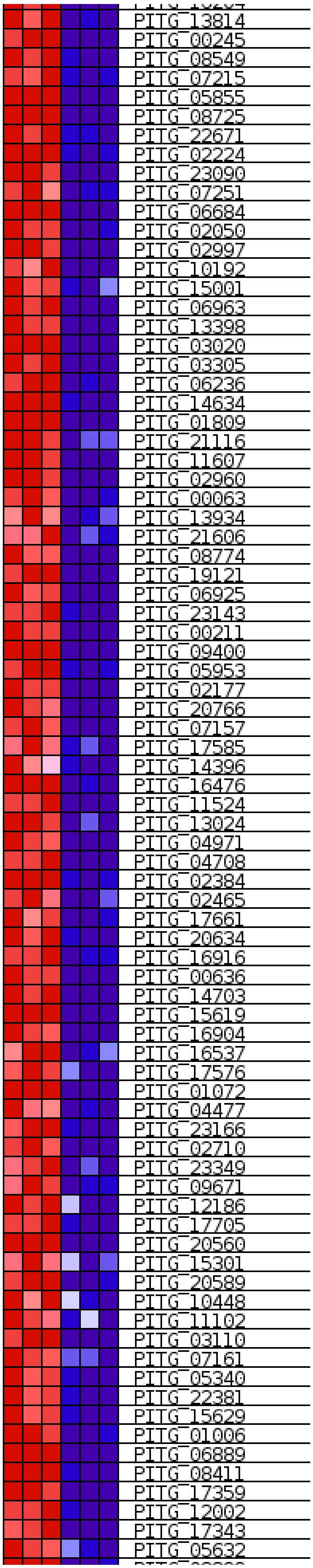
PITG_13814
PITG_00245
PITG_08549
PITG_07215
PITG_05855
PITG_08725
PITG_22671
PITG_02224
PITG_23090
PITG_07251
PITG_06684
PITG_02050
PITG_02997
PITG_10192
PITG_15001
PITG_06963
PITG_13398
PITG_03020
PITG_03305
PITG_06236
PITG_14634
PITG_01809
PITG_21116
PITG_11607
PITG_02960
PITG_00063
PITG_13934
PITG_21606
PITG_08774
PITG_19121
PITG_06925
PITG_23143
PITG_00211
PITG_09400
PITG_05953
PITG_02177
PITG_20766
PITG_07157
PITG_17585
PITG_14396
PITG_16476
PITG_11524
PITG_13024
PITG_04971
PITG_04708
PITG_02384
PITG_02465
PITG_17661
PITG_20634
PITG_16916
PITG_00636
PITG_14703
PITG_15619
PITG_16904
PITG_16537
PITG_17576
PITG_01072
PITG_04477
PITG_23166
PITG_02710
PITG_23349
PITG_09671
PITG_12186
PITG_17705
PITG_20560
PITG_15301
PITG_20589
PITG_10448
PITG_11102
PITG_03110
PITG_07161
PITG_05340
PITG_22381
PITG_15629
PITG_01006
PITG_06889
PITG_08411
PITG_17359
PITG_12002
PITG_17343
PITG_05632

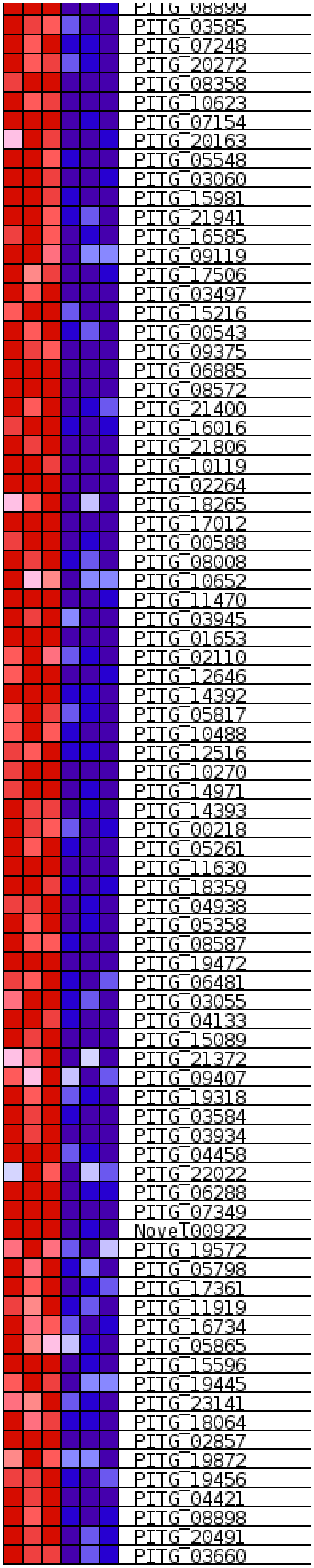
PITG_08899
PITG_03585
PITG_07248
PITG_20272
PITG_08358
PITG_10623
PITG_07154
PITG_20163
PITG_05548
PITG_03060
PITG_15981
PITG_21941
PITG_16585
PITG_09119
PITG_17506
PITG_03497
PITG_15216
PITG_00543
PITG_09375
PITG_06885
PITG_08572
PITG_21400
PITG_16016
PITG_21806
PITG_10119
PITG_02264
PITG_18265
PITG_17012
PITG_00588
PITG_08008
PITG_10652
PITG_11470
PITG_03945
PITG_01653
PITG_02110
PITG_12646
PITG_14392
PITG_05817
PITG_10488
PITG_12516
PITG_10270
PITG_14971
PITG_14393
PITG_00218
PITG_05261
PITG_11630
PITG_18359
PITG_04938
PITG_05358
PITG_08587
PITG_19472
PITG_06481
PITG_03055
PITG_04133
PITG_15089
PITG_21372
PITG_09407
PITG_19318
PITG_03584
PITG_03934
PITG_04458
PITG_22022
PITG_06288
PITG_07349
Novel00922
PITG_19572
PITG_05798
PITG_17361
PITG_11919
PITG_16734
PITG_05865
PITG_15596
PITG_19445
PITG_23141
PITG_18064
PITG_02857
PITG_19872
PITG_19456
PITG_04421
PITG_08898
PITG_20491
PITG_03660

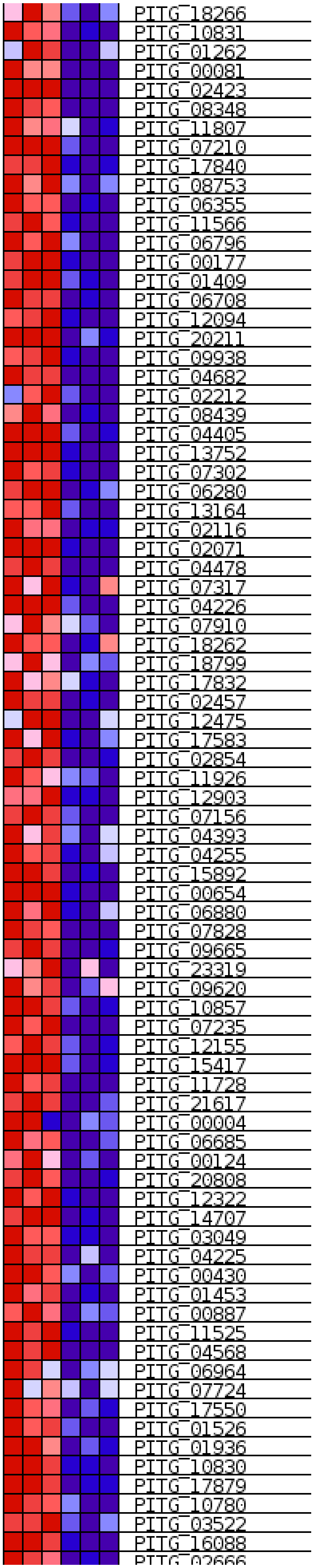
PITG_18266
PITG_10831
PITG_01262
PITG_00081
PITG_02423
PITG_08348
PITG_11807
PITG_07210
PITG_17840
PITG_08753
PITG_06355
PITG_11566
PITG_06796
PITG_00177
PITG_01409
PITG_06708
PITG_12094
PITG_20211
PITG_09938
PITG_04682
PITG_02212
PITG_08439
PITG_04405
PITG_13752
PITG_07302
PITG_06280
PITG_13164
PITG_02116
PITG_02071
PITG_04478
PITG_07317
PITG_04226
PITG_07910
PITG_18262
PITG_18799
PITG_17832
PITG_02457
PITG_12475
PITG_17583
PITG_02854
PITG_11926
PITG_12903
PITG_07156
PITG_04393
PITG_04255
PITG_15892
PITG_00654
PITG_06880
PITG_07828
PITG_09665
PITG_23319
PITG_09620
PITG_10857
PITG_07235
PITG_12155
PITG_15417
PITG_11728
PITG_21617
PITG_00004
PITG_06685
PITG_00124
PITG_20808
PITG_12322
PITG_14707
PITG_03049
PITG_04225
PITG_00430
PITG_01453
PITG_00887
PITG_11525
PITG_04568
PITG_06964
PITG_07724
PITG_17550
PITG_01526
PITG_01936
PITG_10830
PITG_17879
PITG_10780
PITG_03522
PITG_16088
PITG_02666

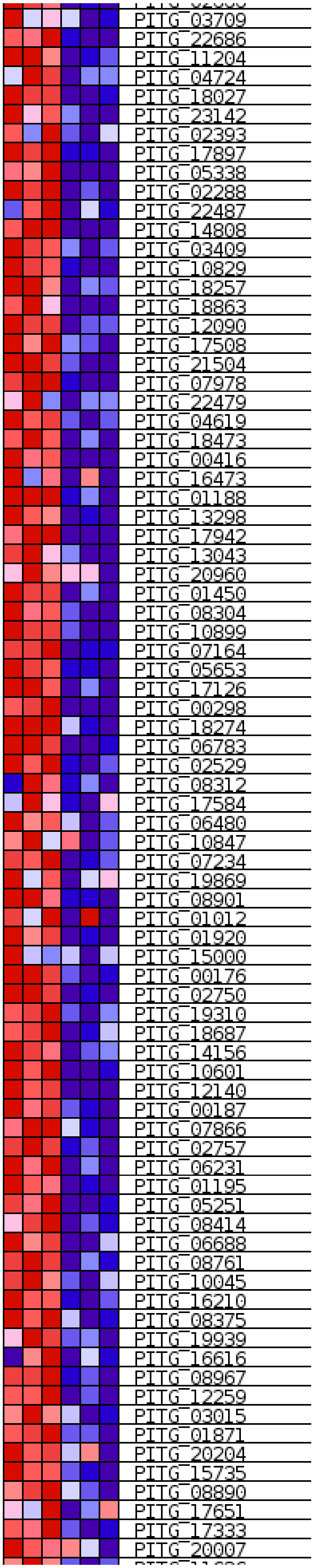
PITG_03709
PITG_22686
PITG_11204
PITG_04724
PITG_18027
PITG_23142
PITG_02393
PITG_17897
PITG_05338
PITG_02288
PITG_22487
PITG_14808
PITG_03409
PITG_10829
PITG_18257
PITG_18863
PITG_12090
PITG_17508
PITG_21504
PITG_07978
PITG_22479
PITG_04619
PITG_18473
PITG_00416
PITG_16473
PITG_01188
PITG_13298
PITG_17942
PITG_13043
PITG_20960
PITG_01450
PITG_08304
PITG_10899
PITG_07164
PITG_05653
PITG_17126
PITG_00298
PITG_18274
PITG_06783
PITG_02529
PITG_08312
PITG_17584
PITG_06480
PITG_10847
PITG_07234
PITG_19869
PITG_08901
PITG_01012
PITG_01920
PITG_15000
PITG_00176
PITG_02750
PITG_19310
PITG_18687
PITG_14156
PITG_10601
PITG_12140
PITG_00187
PITG_07866
PITG_02757
PITG_06231
PITG_01195
PITG_05251
PITG_08414
PITG_06688
PITG_08761
PITG_10045
PITG_16210
PITG_08375
PITG_19939
PITG_16616
PITG_08967
PITG_12259
PITG_03015
PITG_01871
PITG_20204
PITG_15735
PITG_08890
PITG_17651
PITG_17333
PITG_20007

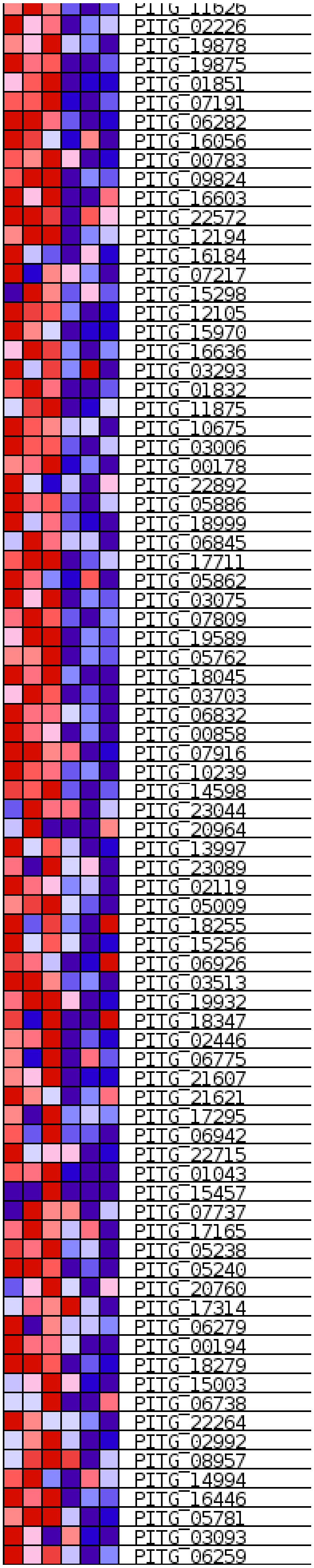
PITG_11626
PITG_02226
PITG_19878
PITG_19875
PITG_01851
PITG_07191
PITG_06282
PITG_16056
PITG_00783
PITG_09824
PITG_16603
PITG_22572
PITG_12194
PITG_16184
PITG_07217
PITG_15298
PITG_12105
PITG_15970
PITG_16636
PITG_03293
PITG_01832
PITG_11875
PITG_10675
PITG_03006
PITG_00178
PITG_22892
PITG_05886
PITG_18999
PITG_06845
PITG_17711
PITG_05862
PITG_03075
PITG_07809
PITG_19589
PITG_05762
PITG_18045
PITG_03703
PITG_06832
PITG_00858
PITG_07916
PITG_10239
PITG_14598
PITG_23044
PITG_20964
PITG_13997
PITG_23089
PITG_02119
PITG_05009
PITG_18255
PITG_15256
PITG_06926
PITG_03513
PITG_19932
PITG_18347
PITG_02446
PITG_06775
PITG_21607
PITG_21621
PITG_17295
PITG_06942
PITG_22715
PITG_01043
PITG_15457
PITG_07737
PITG_17165
PITG_05238
PITG_05240
PITG_20760
PITG_17314
PITG_06279
PITG_00194
PITG_18279
PITG_15003
PITG_06738
PITG_22264
PITG_02992
PITG_08957
PITG_14994
PITG_16446
PITG_05781
PITG_03093
PITG_06259

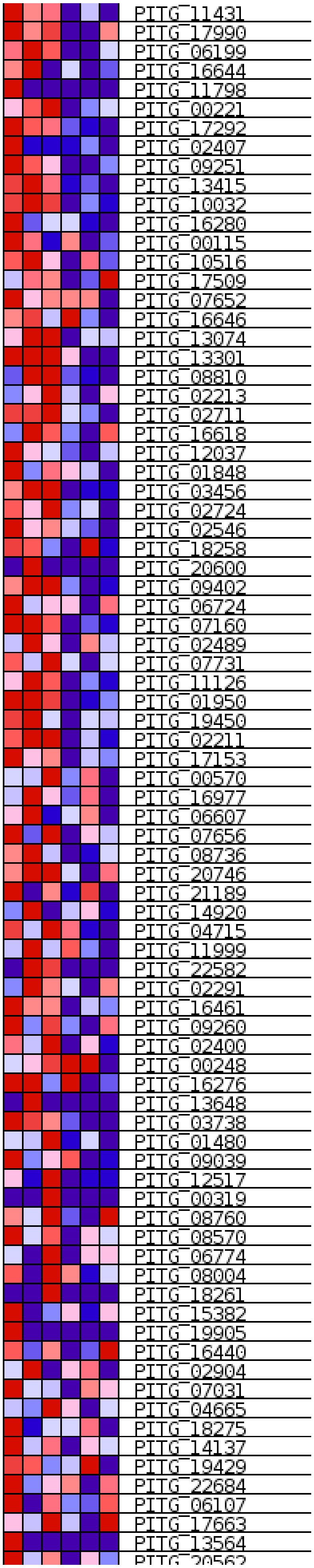
PITG_11431
PITG_17990
PITG_06199
PITG_16644
PITG_11798
PITG_00221
PITG_17292
PITG_02407
PITG_09251
PITG_13415
PITG_10032
PITG_16280
PITG_00115
PITG_10516
PITG_17509
PITG_07652
PITG_16646
PITG_13074
PITG_13301
PITG_08810
PITG_02213
PITG_02711
PITG_16618
PITG_12037
PITG_01848
PITG_03456
PITG_02724
PITG_02546
PITG_18258
PITG_20600
PITG_09402
PITG_06724
PITG_07160
PITG_02489
PITG_07731
PITG_11126
PITG_01950
PITG_19450
PITG_02211
PITG_17153
PITG_00570
PITG_16977
PITG_06607
PITG_07656
PITG_08736
PITG_20746
PITG_21189
PITG_14920
PITG_04715
PITG_11999
PITG_22582
PITG_02291
PITG_16461
PITG_09260
PITG_02400
PITG_00248
PITG_16276
PITG_13648
PITG_03738
PITG_01480
PITG_09039
PITG_12517
PITG_00319
PITG_08760
PITG_08570
PITG_06774
PITG_08004
PITG_18261
PITG_15382
PITG_19905
PITG_16440
PITG_02904
PITG_07031
PITG_04665
PITG_18275
PITG_14137
PITG_19429
PITG_22684
PITG_06107
PITG_17663
PITG_13564
PITG_20562

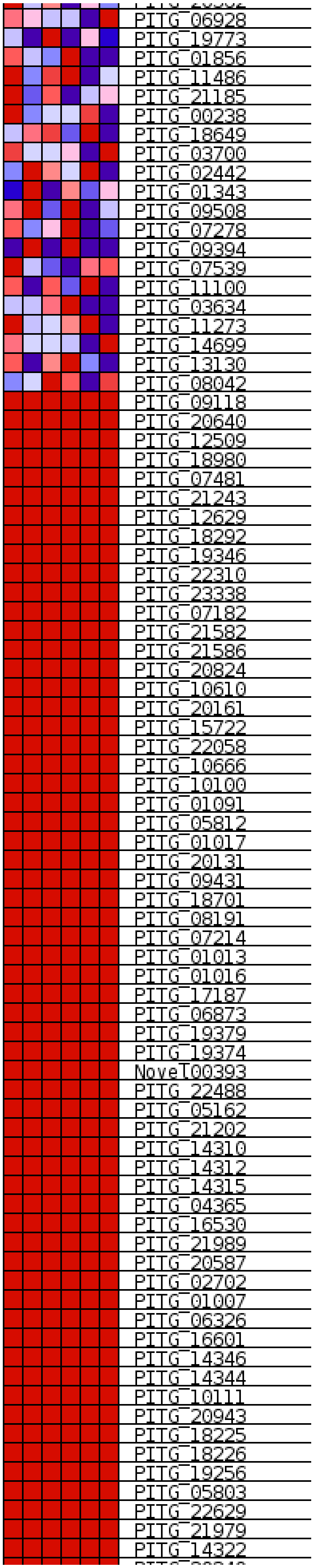
PITG_06928
PITG_19773
PITG_01856
PITG_11486
PITG_21185
PITG_00238
PITG_18649
PITG_03700
PITG_02442
PITG_01343
PITG_09508
PITG_07278
PITG_09394
PITG_07539
PITG_11100
PITG_03634
PITG_11273
PITG_14699
PITG_13130
PITG_08042
PITG_09118
PITG_20640
PITG_12509
PITG_18980
PITG_07481
PITG_21243
PITG_12629
PITG_18292
PITG_19346
PITG_22310
PITG_23338
PITG_07182
PITG_21582
PITG_21586
PITG_20824
PITG_10610
PITG_20161
PITG_15722
PITG_22058
PITG_10666
PITG_10100
PITG_01091
PITG_05812
PITG_01017
PITG_20131
PITG_09431
PITG_18701
PITG_08191
PITG_07214
PITG_01013
PITG_01016
PITG_17187
PITG_06873
PITG_19379
PITG_19374
Novel00393
PITG_22488
PITG_05162
PITG_21202
PITG_14310
PITG_14312
PITG_14315
PITG_04365
PITG_16530
PITG_21989
PITG_20587
PITG_02702
PITG_01007
PITG_06326
PITG_16601
PITG_14346
PITG_14344
PITG_10111
PITG_20943
PITG_18225
PITG_18226
PITG_19256
PITG_05803
PITG_22629
PITG_21979
PITG_14322

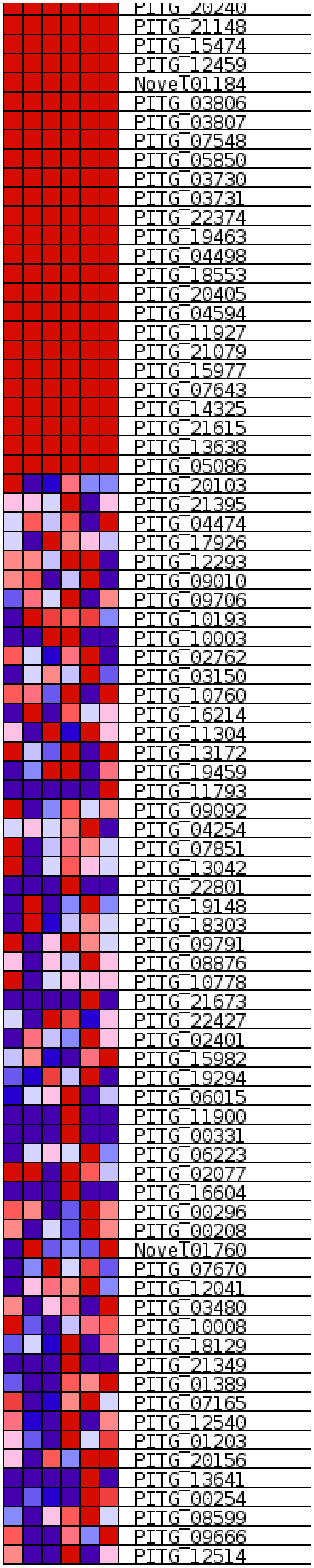
PITG_21148
PITG_15474
PITG_12459
Novel01184
PITG_03806
PITG_03807
PITG_07548
PITG_05850
PITG_03730
PITG_03731
PITG_22374
PITG_19463
PITG_04498
PITG_18553
PITG_20405
PITG_04594
PITG_11927
PITG_21079
PITG_15977
PITG_07643
PITG_14325
PITG_21615
PITG_13638
PITG_05086
PITG_20103
PITG_21395
PITG_04474
PITG_17926
PITG_12293
PITG_09010
PITG_09706
PITG_10193
PITG_10003
PITG_02762
PITG_03150
PITG_10760
PITG_16214
PITG_11304
PITG_13172
PITG_19459
PITG_11793
PITG_09092
PITG_04254
PITG_07851
PITG_13042
PITG_22801
PITG_19148
PITG_18303
PITG_09791
PITG_08876
PITG_10778
PITG_21673
PITG_22427
PITG_02401
PITG_15982
PITG_19294
PITG_06015
PITG_11900
PITG_00331
PITG_06223
PITG_02077
PITG_16604
PITG_00296
PITG_00208
Novel01760
PITG_07670
PITG_12041
PITG_03480
PITG_10008
PITG_18129
PITG_21349
PITG_01389
PITG_07165
PITG_12540
PITG_01203
PITG_20156
PITG_13641
PITG_00254
PITG_08599
PITG_09666
PITG_12514

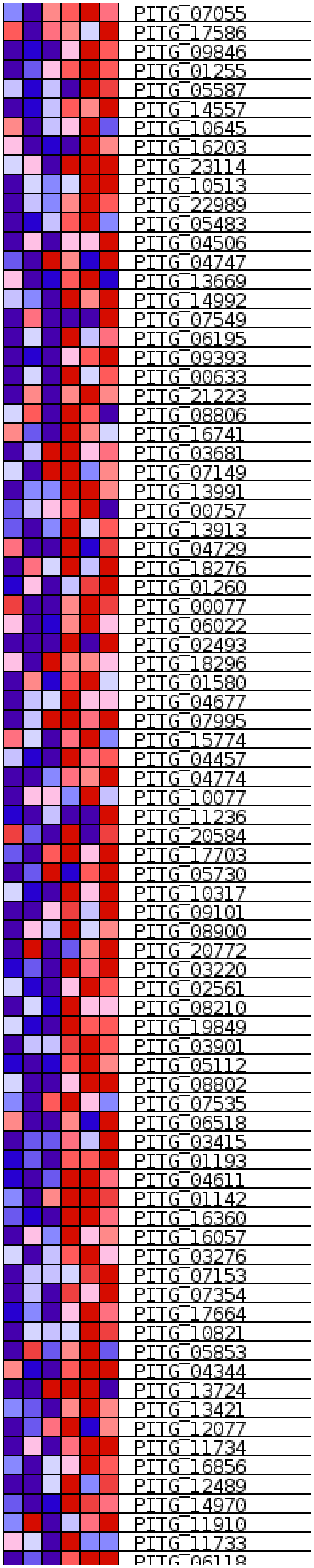
PITG_07055
PITG_17586
PITG_09846
PITG_01255
PITG_05587
PITG_14557
PITG_10645
PITG_16203
PITG_23114
PITG_10513
PITG_22989
PITG_05483
PITG_04506
PITG_04747
PITG_13669
PITG_14992
PITG_07549
PITG_06195
PITG_09393
PITG_00633
PITG_21223
PITG_08806
PITG_16741
PITG_03681
PITG_07149
PITG_13991
PITG_00757
PITG_13913
PITG_04729
PITG_18276
PITG_01260
PITG_00077
PITG_06022
PITG_02493
PITG_18296
PITG_01580
PITG_04677
PITG_07995
PITG_15774
PITG_04457
PITG_04774
PITG_10077
PITG_11236
PITG_20584
PITG_17703
PITG_05730
PITG_10317
PITG_09101
PITG_08900
PITG_20772
PITG_03220
PITG_02561
PITG_08210
PITG_19849
PITG_03901
PITG_05112
PITG_08802
PITG_07535
PITG_06518
PITG_03415
PITG_01193
PITG_04611
PITG_01142
PITG_16360
PITG_16057
PITG_03276
PITG_07153
PITG_07354
PITG_17664
PITG_10821
PITG_05853
PITG_04344
PITG_13724
PITG_13421
PITG_12077
PITG_11734
PITG_16856
PITG_12489
PITG_14970
PITG_11910
PITG_11733
PITG_06118

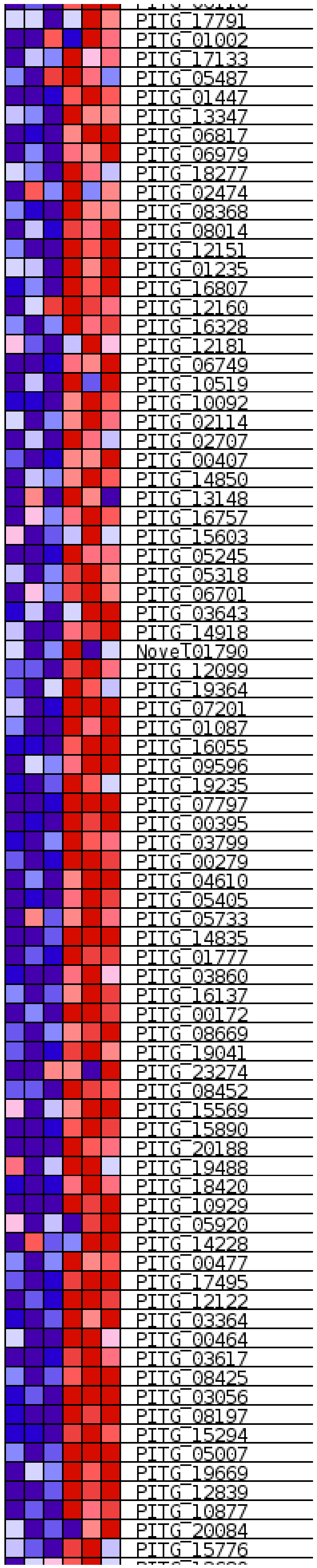
PITG_17791
PITG_01002
PITG_17133
PITG_05487
PITG_01447
PITG_13347
PITG_06817
PITG_06979
PITG_18277
PITG_02474
PITG_08368
PITG_08014
PITG_12151
PITG_01235
PITG_16807
PITG_12160
PITG_16328
PITG_12181
PITG_06749
PITG_10519
PITG_10092
PITG_02114
PITG_02707
PITG_00407
PITG_14850
PITG_13148
PITG_16757
PITG_15603
PITG_05245
PITG_05318
PITG_06701
PITG_03643
PITG_14918
Novel01790
PITG_12099
PITG_19364
PITG_07201
PITG_01087
PITG_16055
PITG_09596
PITG_19235
PITG_07797
PITG_00395
PITG_03799
PITG_00279
PITG_04610
PITG_05405
PITG_05733
PITG_14835
PITG_01777
PITG_03860
PITG_16137
PITG_00172
PITG_08669
PITG_19041
PITG_23274
PITG_08452
PITG_15569
PITG_15890
PITG_20188
PITG_19488
PITG_18420
PITG_10929
PITG_05920
PITG_14228
PITG_00477
PITG_17495
PITG_12122
PITG_03364
PITG_00464
PITG_03617
PITG_08425
PITG_03056
PITG_08197
PITG_15294
PITG_05007
PITG_19669
PITG_12839
PITG_10877
PITG_20084
PITG_15776

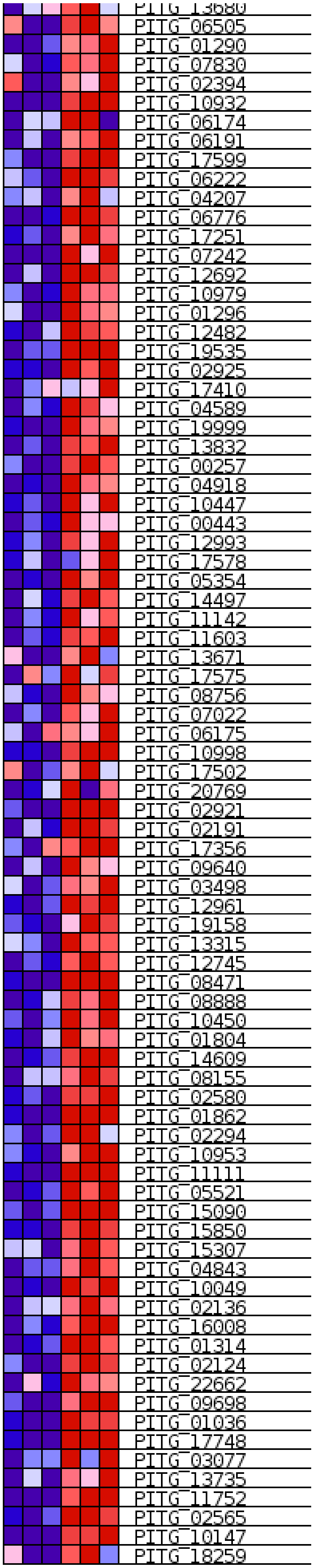
PITG_13680
PITG_06505
PITG_01290
PITG_07830
PITG_02394
PITG_10932
PITG_06174
PITG_06191
PITG_17599
PITG_06222
PITG_04207
PITG_06776
PITG_17251
PITG_07242
PITG_12692
PITG_10979
PITG_01296
PITG_12482
PITG_19535
PITG_02925
PITG_17410
PITG_04589
PITG_19999
PITG_13832
PITG_00257
PITG_04918
PITG_10447
PITG_00443
PITG_12993
PITG_17578
PITG_05354
PITG_14497
PITG_11142
PITG_11603
PITG_13671
PITG_17575
PITG_08756
PITG_07022
PITG_06175
PITG_10998
PITG_17502
PITG_20769
PITG_02921
PITG_02191
PITG_17356
PITG_09640
PITG_03498
PITG_12961
PITG_19158
PITG_13315
PITG_12745
PITG_08471
PITG_08888
PITG_10450
PITG_01804
PITG_14609
PITG_08155
PITG_02580
PITG_01862
PITG_02294
PITG_10953
PITG_11111
PITG_05521
PITG_15090
PITG_15850
PITG_15307
PITG_04843
PITG_10049
PITG_02136
PITG_16008
PITG_01314
PITG_02124
PITG_22662
PITG_09698
PITG_01036
PITG_17748
PITG_03077
PITG_13735
PITG_11752
PITG_02565
PITG_10147
PITG_18259

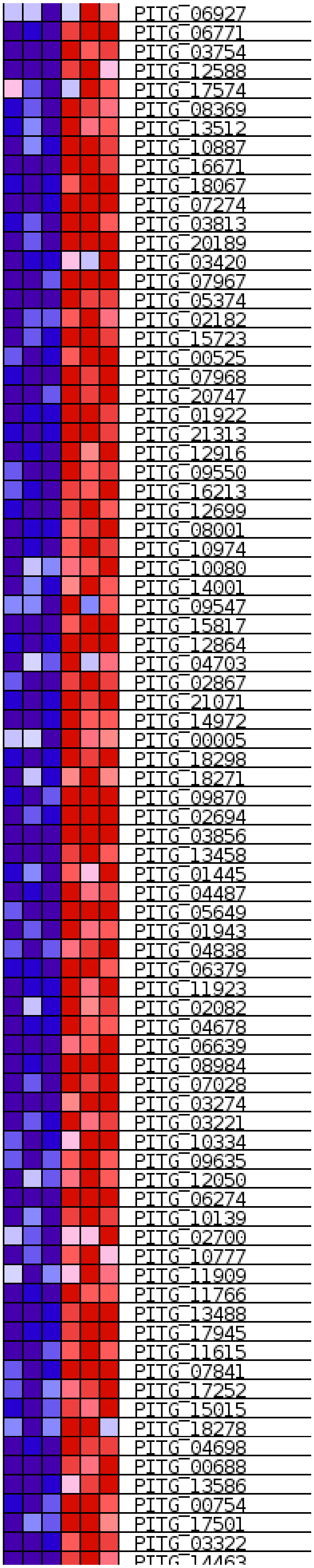
PITG_06927
PITG_06771
PITG_03754
PITG_12588
PITG_17574
PITG_08369
PITG_13512
PITG_10887
PITG_16671
PITG_18067
PITG_07274
PITG_03813
PITG_20189
PITG_03420
PITG_07967
PITG_05374
PITG_02182
PITG_15723
PITG_00525
PITG_07968
PITG_20747
PITG_01922
PITG_21313
PITG_12916
PITG_09550
PITG_16213
PITG_12699
PITG_08001
PITG_10974
PITG_10080
PITG_14001
PITG_09547
PITG_15817
PITG_12864
PITG_04703
PITG_02867
PITG_21071
PITG_14972
PITG_00005
PITG_18298
PITG_18271
PITG_09870
PITG_02694
PITG_03856
PITG_13458
PITG_01445
PITG_04487
PITG_05649
PITG_01943
PITG_04838
PITG_06379
PITG_11923
PITG_02082
PITG_04678
PITG_06639
PITG_08984
PITG_07028
PITG_03274
PITG_03221
PITG_10334
PITG_09635
PITG_12050
PITG_06274
PITG_10139
PITG_02700
PITG_10777
PITG_11909
PITG_11766
PITG_13488
PITG_17945
PITG_11615
PITG_07841
PITG_17252
PITG_15015
PITG_18278
PITG_04698
PITG_00688
PITG_13586
PITG_00754
PITG_17501
PITG_03322
PITG_14463

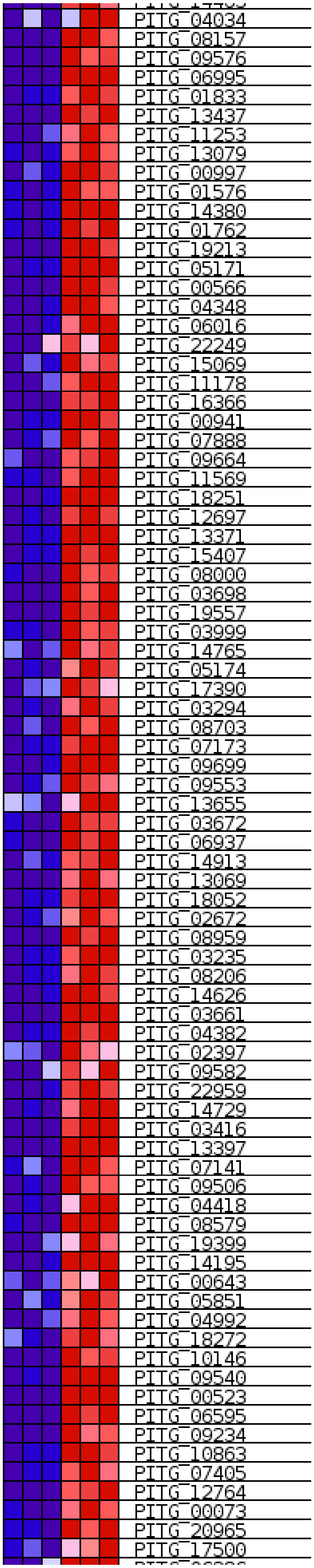
PITG_04034
PITG_08157
PITG_09576
PITG_06995
PITG_01833
PITG_13437
PITG_11253
PITG_13079
PITG_00997
PITG_01576
PITG_14380
PITG_01762
PITG_19213
PITG_05171
PITG_00566
PITG_04348
PITG_06016
PITG_22249
PITG_15069
PITG_11178
PITG_16366
PITG_00941
PITG_07888
PITG_09664
PITG_11569
PITG_18251
PITG_12697
PITG_13371
PITG_15407
PITG_08000
PITG_03698
PITG_19557
PITG_03999
PITG_14765
PITG_05174
PITG_17390
PITG_03294
PITG_08703
PITG_07173
PITG_09699
PITG_09553
PITG_13655
PITG_03672
PITG_06937
PITG_14913
PITG_13069
PITG_18052
PITG_02672
PITG_08959
PITG_03235
PITG_08206
PITG_14626
PITG_03661
PITG_04382
PITG_02397
PITG_09582
PITG_22959
PITG_14729
PITG_03416
PITG_13397
PITG_07141
PITG_09506
PITG_04418
PITG_08579
PITG_19399
PITG_14195
PITG_00643
PITG_05851
PITG_04992
PITG_18272
PITG_10146
PITG_09540
PITG_00523
PITG_06595
PITG_09234
PITG_10863
PITG_07405
PITG_12764
PITG_00073
PITG_20965
PITG_17500

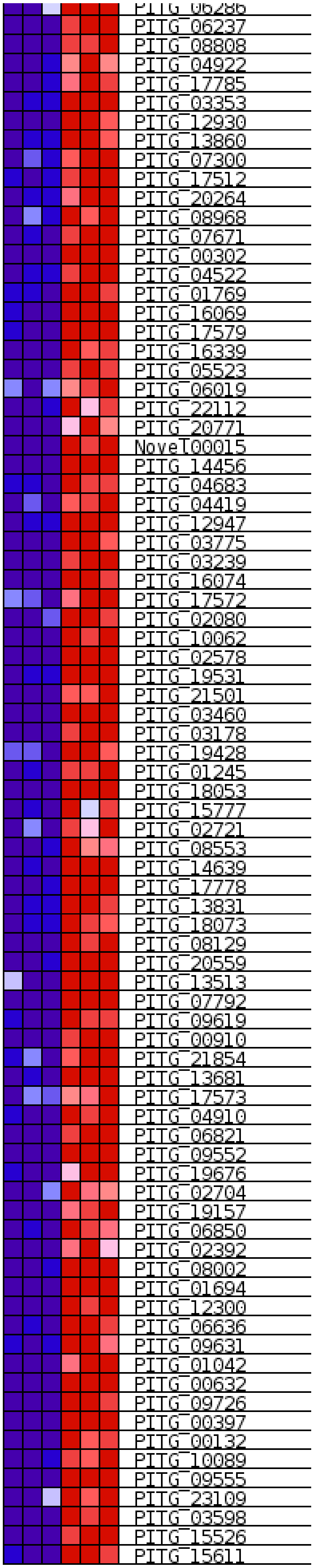
PITG_06286
PITG_06237
PITG_08808
PITG_04922
PITG_17785
PITG_03353
PITG_12930
PITG_13860
PITG_07300
PITG_17512
PITG_20264
PITG_08968
PITG_07671
PITG_00302
PITG_04522
PITG_01769
PITG_16069
PITG_17579
PITG_16339
PITG_05523
PITG_06019
PITG_22112
PITG_20771
Novel00015
PITG_14456
PITG_04683
PITG_04419
PITG_12947
PITG_03775
PITG_03239
PITG_16074
PITG_17572
PITG_02080
PITG_10062
PITG_02578
PITG_19531
PITG_21501
PITG_03460
PITG_03178
PITG_19428
PITG_01245
PITG_18053
PITG_15777
PITG_02721
PITG_08553
PITG_14639
PITG_17778
PITG_13831
PITG_18073
PITG_08129
PITG_20559
PITG_13513
PITG_07792
PITG_09619
PITG_00910
PITG_21854
PITG_13681
PITG_17573
PITG_04910
PITG_06821
PITG_09552
PITG_19676
PITG_02704
PITG_19157
PITG_06850
PITG_02392
PITG_08002
PITG_01694
PITG_12300
PITG_06636
PITG_09631
PITG_01042
PITG_00632
PITG_09726
PITG_00397
PITG_00132
PITG_10089
PITG_09555
PITG_23109
PITG_03598
PITG_15526
PITG_15611

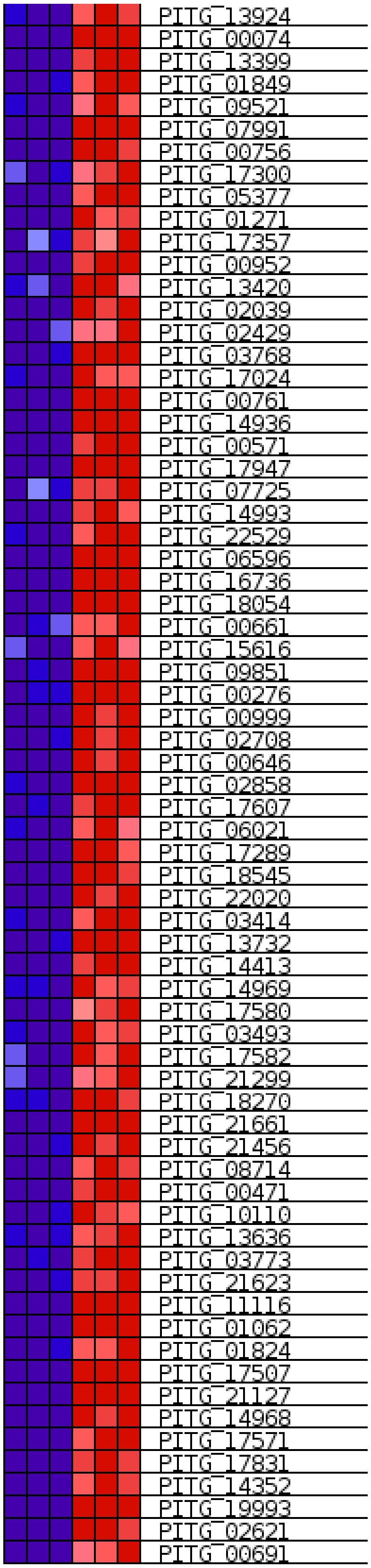

Fig 2: ORGANONITROGEN_COMPOUND_METABOLIC_PROCESS(GO:1901564)
Blue-Pink O' Gram in the Space of the Analyzed GeneSet

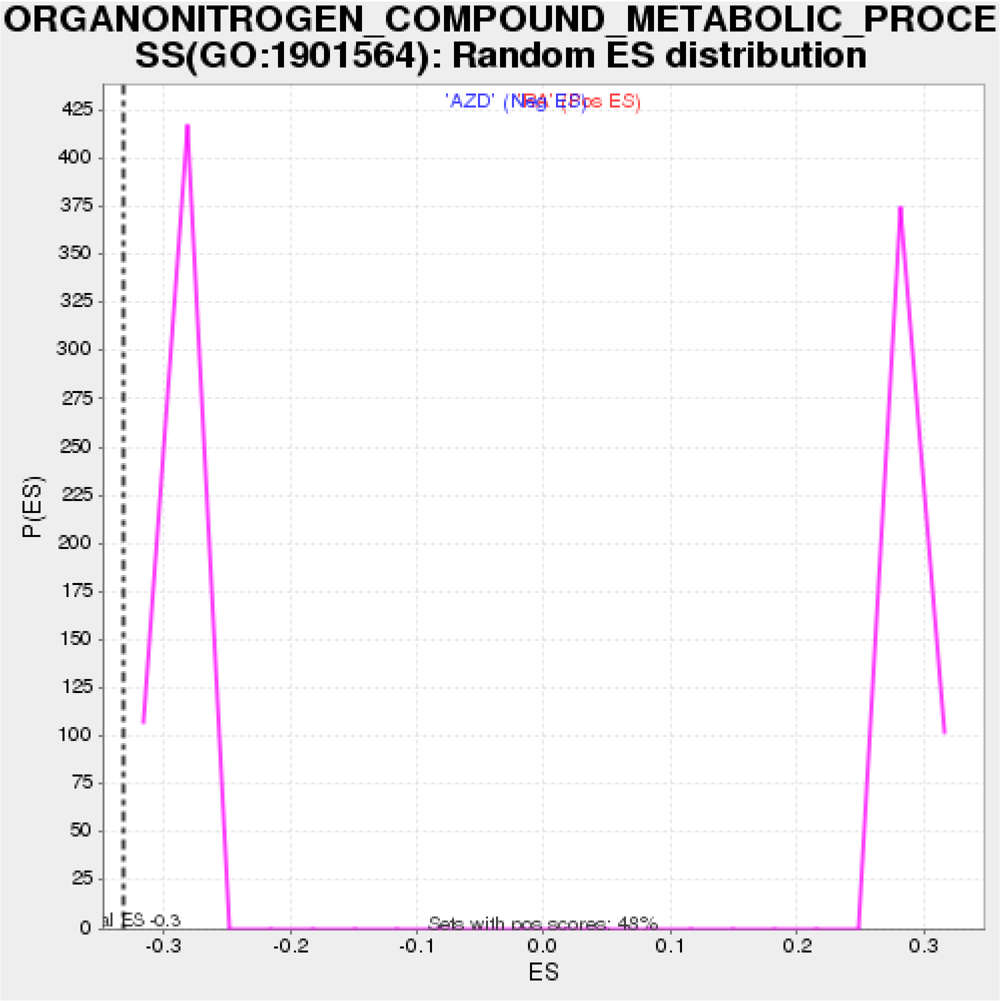

Fig 3: ORGANONITROGEN_COMPOUND_METABOLIC_PROCESS(GO:1901564): Random ES distribution

Gene set null distribution of ES for ORGANONITROGEN_COMPOUND_METABOLIC_PROCESS(GO:1901564)

4. peptide biosynthetic process

Table: GSEA Results Summary

Dataset	fpkm.sample
Phenotype	sample.cls
Upregulated in class	AZD
GeneSet	PEPTIDE_BIOSYNTHETIC_PROCESS(GO:0043043)
Enrichment Score (ES)	-0.7306118
Normalized Enrichment Score (NES)	-1.0785583
Nominal p-value	0.0
FDR q-value	0.10687017
FWER p-Value	0.056

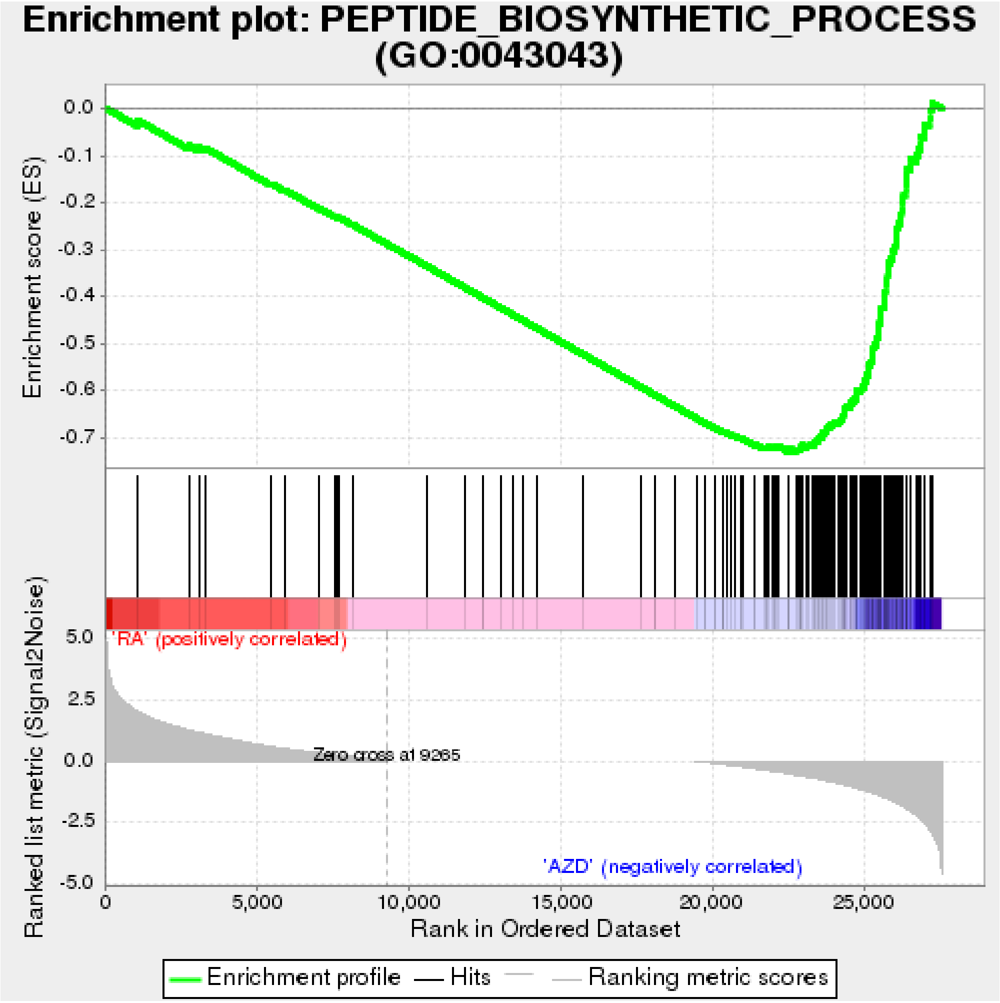

Fig 1: Enrichment plot: PEPTIDE_BIOSYNTHETIC_PROCESS(GO:0043043)
Profile of the Running ES Score & Positions of GeneSet Members on the Rank Ordered List

Table: GSEA details [plain text format]

	PROBE	DESCRIPTION	GENE	GENE_TITLE	RANK IN	RANK	RUNNING	CORE

		(from dataset)	SYMBOL		GENE LIST	METRIC SCORE	ES	ENRICHMENT
1	PITG_19121	PITG_19121			1014	2.048	-0.0242	No
2	PITG_11630	PITG_11630			2711	1.279	-0.0781	No
3	Novel00922	Novel00922			3038	1.181	-0.0826	No
4	PITG_03660	PITG_03660			3265	1.116	-0.0838	No
5	PITG_07234	PITG_07234			5449	0.602	-0.1597	No
6	PITG_17651	PITG_17651			5906	0.516	-0.1731	No
7	PITG_05009	PITG_05009			7048	0.319	-0.2128	No
8	PITG_02992	PITG_02992			7548	0.243	-0.2295	No
9	PITG_03093	PITG_03093			7622	0.232	-0.2307	No
10	PITG_10516	PITG_10516			7711	0.219	-0.2325	No
11	PITG_17153	PITG_17153			8119	0.169	-0.2463	No
12	PITG_22310	PITG_22310			10562	0.000	-0.3354	No
13	PITG_20824	PITG_20824			11824	0.000	-0.3814	No
14	PITG_15722	PITG_15722			12398	0.000	-0.4023	No
15	PITG_22058	PITG_22058			12412	0.000	-0.4028	No
16	PITG_01091	PITG_01091			13039	0.000	-0.4256	No
17	PITG_05812	PITG_05812			13417	0.000	-0.4394	No
18	PITG_09431	PITG_09431			13717	0.000	-0.4503	No
19	PITG_06873	PITG_06873			14206	0.000	-0.4681	No
20	PITG_16530	PITG_16530			15706	0.000	-0.5228	No
21	PITG_20240	PITG_20240			17604	0.000	-0.5920	No
22	PITG_03806	PITG_03806			18089	0.000	-0.6097	No
23	PITG_03807	PITG_03807			18090	0.000	-0.6097	No
24	PITG_04594	PITG_04594			18776	0.000	-0.6347	No
25	PITG_10193	PITG_10193			19492	-0.015	-0.6607	No
26	PITG_18303	PITG_18303			19751	-0.048	-0.6698	No
27	PITG_09791	PITG_09791			19763	-0.050	-0.6699	No
28	PITG_03480	PITG_03480			20045	-0.095	-0.6796	No
29	PITG_21349	PITG_21349			20105	-0.104	-0.6811	No
30	PITG_09846	PITG_09846			20357	-0.142	-0.6893	No
31	PITG_01255	PITG_01255			20360	-0.142	-0.6885	No
32	PITG_14557	PITG_14557			20366	-0.143	-0.6878	No
33	PITG_04747	PITG_04747			20459	-0.156	-0.6902	No
34	PITG_16741	PITG_16741			20622	-0.179	-0.6950	No
35	PITG_04729	PITG_04729			20710	-0.194	-0.6969	No
36	PITG_02493	PITG_02493			20900	-0.221	-0.7025	No
37	PITG_04774	PITG_04774			20937	-0.229	-0.7023	No
38	PITG_05730	PITG_05730			21018	-0.243	-0.7037	No

39	PITG_12077	PITG_12077			21406	-0.311	-0.7159	No
40	PITG_11734	PITG_11734			21411	-0.312	-0.7141	No
41	PITG_12151	PITG_12151			21701	-0.361	-0.7224	No
42	PITG_16328	PITG_16328			21756	-0.372	-0.7220	No
43	PITG_14850	PITG_14850			21815	-0.384	-0.7217	No
44	PITG_16757	PITG_16757			21821	-0.385	-0.7195	No
45	PITG_07797	PITG_07797			21968	-0.412	-0.7223	No
46	PITG_03799	PITG_03799			22019	-0.421	-0.7215	No
47	PITG_05405	PITG_05405			22072	-0.429	-0.7207	No
48	PITG_05733	PITG_05733			22091	-0.432	-0.7186	No
49	PITG_20188	PITG_20188			22157	-0.445	-0.7182	No
50	PITG_05007	PITG_05007			22498	-0.509	-0.7274	Yes
51	PITG_19669	PITG_19669			22503	-0.510	-0.7244	Yes
52	PITG_12839	PITG_12839			22518	-0.514	-0.7217	Yes
53	PITG_06222	PITG_06222			22748	-0.566	-0.7265	Yes
54	PITG_10979	PITG_10979			22822	-0.583	-0.7255	Yes
55	PITG_19999	PITG_19999			22878	-0.594	-0.7238	Yes
56	PITG_04918	PITG_04918			22927	-0.602	-0.7218	Yes
57	PITG_00443	PITG_00443			22936	-0.604	-0.7183	Yes
58	PITG_05354	PITG_05354			22976	-0.613	-0.7159	Yes
59	PITG_02921	PITG_02921			23131	-0.650	-0.7174	Yes
60	PITG_12961	PITG_12961			23203	-0.663	-0.7158	Yes
61	PITG_12745	PITG_12745			23280	-0.681	-0.7144	Yes
62	PITG_14609	PITG_14609			23351	-0.695	-0.7126	Yes
63	PITG_02580	PITG_02580			23358	-0.695	-0.7084	Yes
64	PITG_11111	PITG_11111			23461	-0.720	-0.7076	Yes
65	PITG_15090	PITG_15090			23482	-0.725	-0.7038	Yes
66	PITG_04843	PITG_04843			23523	-0.735	-0.7007	Yes
67	PITG_16008	PITG_16008			23545	-0.740	-0.6968	Yes
68	PITG_17748	PITG_17748			23641	-0.765	-0.6955	Yes
69	PITG_13735	PITG_13735			23644	-0.766	-0.6908	Yes
70	PITG_06771	PITG_06771			23729	-0.789	-0.6889	Yes
71	PITG_08369	PITG_08369			23772	-0.802	-0.6854	Yes
72	PITG_10887	PITG_10887			23788	-0.807	-0.6809	Yes
73	PITG_20189	PITG_20189			23835	-0.820	-0.6775	Yes
74	PITG_03420	PITG_03420			23870	-0.829	-0.6735	Yes
75	PITG_15723	PITG_15723			23962	-0.858	-0.6715	Yes
76	PITG_01922	PITG_01922			24019	-0.874	-0.6680	Yes
77	PITG_10974	PITG_10974			24137	-0.910	-0.6666	Yes

78	PITG_12864	PITG_12864			24223	-0.936	-0.6638	Yes
79	PITG_04703	PITG_04703			24269	-0.949	-0.6595	Yes
80	PITG_21071	PITG_21071			24300	-0.958	-0.6546	Yes
81	PITG_02694	PITG_02694			24328	-0.970	-0.6496	Yes
82	PITG_04487	PITG_04487			24365	-0.980	-0.6447	Yes
83	PITG_01943	PITG_01943			24378	-0.982	-0.6390	Yes
84	PITG_11923	PITG_11923			24405	-0.990	-0.6338	Yes
85	PITG_03274	PITG_03274			24545	-1.036	-0.6324	Yes
86	PITG_03221	PITG_03221			24557	-1.040	-0.6262	Yes
87	PITG_11766	PITG_11766			24642	-1.064	-0.6226	Yes
88	PITG_07841	PITG_07841			24668	-1.073	-0.6168	Yes
89	PITG_03322	PITG_03322			24740	-1.096	-0.6126	Yes
90	PITG_06995	PITG_06995			24760	-1.104	-0.6064	Yes
91	PITG_01833	PITG_01833			24767	-1.106	-0.5996	Yes
92	PITG_01762	PITG_01762			24911	-1.153	-0.5976	Yes
93	PITG_05171	PITG_05171			24921	-1.158	-0.5907	Yes
94	PITG_22249	PITG_22249			25013	-1.195	-0.5866	Yes
95	PITG_15069	PITG_15069			25019	-1.198	-0.5792	Yes
96	PITG_00941	PITG_00941			25067	-1.214	-0.5734	Yes
97	PITG_07888	PITG_07888			25072	-1.216	-0.5659	Yes
98	PITG_18251	PITG_18251			25141	-1.242	-0.5606	Yes
99	PITG_12697	PITG_12697			25164	-1.249	-0.5536	Yes
100	PITG_13371	PITG_13371			25168	-1.249	-0.5459	Yes
101	PITG_15407	PITG_15407			25203	-1.263	-0.5392	Yes
102	PITG_03999	PITG_03999			25257	-1.283	-0.5331	Yes
103	PITG_05174	PITG_05174			25267	-1.288	-0.5254	Yes
104	PITG_03294	PITG_03294			25303	-1.306	-0.5185	Yes
105	PITG_08703	PITG_08703			25308	-1.308	-0.5104	Yes
106	PITG_07173	PITG_07173			25342	-1.325	-0.5033	Yes
107	PITG_14913	PITG_14913			25405	-1.356	-0.4971	Yes
108	PITG_18052	PITG_18052			25424	-1.365	-0.4892	Yes
109	PITG_08959	PITG_08959			25458	-1.380	-0.4818	Yes
110	PITG_03235	PITG_03235			25480	-1.389	-0.4738	Yes
111	PITG_03661	PITG_03661			25487	-1.391	-0.4653	Yes
112	PITG_04382	PITG_04382			25503	-1.398	-0.4571	Yes
113	PITG_14729	PITG_14729			25528	-1.410	-0.4492	Yes
114	PITG_07141	PITG_07141			25545	-1.420	-0.4409	Yes
115	PITG_09506	PITG_09506			25563	-1.431	-0.4325	Yes
116	PITG_08579	PITG_08579			25572	-1.438	-0.4238	Yes

117	PITG_04992	PITG_04992			25670	-1.484	-0.4181	Yes
118	PITG_10146	PITG_10146			25685	-1.491	-0.4092	Yes
119	PITG_09540	PITG_09540			25692	-1.493	-0.4001	Yes
120	PITG_00523	PITG_00523			25696	-1.497	-0.3909	Yes
121	PITG_09234	PITG_09234			25712	-1.502	-0.3820	Yes
122	PITG_10863	PITG_10863			25713	-1.502	-0.3726	Yes
123	PITG_06237	PITG_06237			25757	-1.523	-0.3646	Yes
124	PITG_17785	PITG_17785			25774	-1.532	-0.3556	Yes
125	PITG_03353	PITG_03353			25780	-1.535	-0.3462	Yes
126	PITG_07300	PITG_07300			25805	-1.550	-0.3374	Yes
127	PITG_20264	PITG_20264			25831	-1.561	-0.3285	Yes
128	PITG_00302	PITG_00302			25872	-1.579	-0.3201	Yes
129	Novel00015	Novel00015			25949	-1.627	-0.3127	Yes
130	PITG_14456	PITG_14456			25952	-1.628	-0.3025	Yes
131	PITG_12947	PITG_12947			26003	-1.658	-0.2940	Yes
132	PITG_03239	PITG_03239			26044	-1.683	-0.2849	Yes
133	PITG_02578	PITG_02578			26070	-1.705	-0.2751	Yes
134	PITG_19531	PITG_19531			26082	-1.713	-0.2648	Yes
135	PITG_03460	PITG_03460			26094	-1.719	-0.2544	Yes
136	PITG_03178	PITG_03178			26113	-1.732	-0.2443	Yes
137	PITG_13831	PITG_13831			26183	-1.780	-0.2356	Yes
138	PITG_00910	PITG_00910			26229	-1.807	-0.2260	Yes
139	PITG_13681	PITG_13681			26238	-1.816	-0.2149	Yes
140	PITG_06821	PITG_06821			26267	-1.833	-0.2044	Yes
141	PITG_09552	PITG_09552			26270	-1.835	-0.1930	Yes
142	PITG_19157	PITG_19157			26301	-1.850	-0.1825	Yes
143	PITG_06636	PITG_06636			26370	-1.898	-0.1731	Yes
144	PITG_09631	PITG_09631			26373	-1.899	-0.1613	Yes
145	PITG_01042	PITG_01042			26385	-1.915	-0.1497	Yes
146	PITG_00397	PITG_00397			26402	-1.929	-0.1382	Yes
147	PITG_09555	PITG_09555			26429	-1.950	-0.1270	Yes
148	PITG_09521	PITG_09521			26522	-2.027	-0.1176	Yes
149	PITG_07991	PITG_07991			26527	-2.030	-0.1051	Yes
150	PITG_02039	PITG_02039			26754	-2.231	-0.0993	Yes
151	PITG_03768	PITG_03768			26780	-2.261	-0.0861	Yes
152	PITG_06596	PITG_06596			26861	-2.351	-0.0743	Yes
153	PITG_18054	PITG_18054			26868	-2.362	-0.0597	Yes
154	PITG_17607	PITG_17607			26969	-2.477	-0.0479	Yes
155	PITG_22020	PITG_22020			27018	-2.545	-0.0337	Yes

156	PITG_21661	PITG_21661			27161	-2.778	-0.0215	Yes
157	PITG_08714	PITG_08714			27196	-2.846	-0.0049	Yes
158	PITG_10110	PITG_10110			27232	-2.902	0.0120	Yes

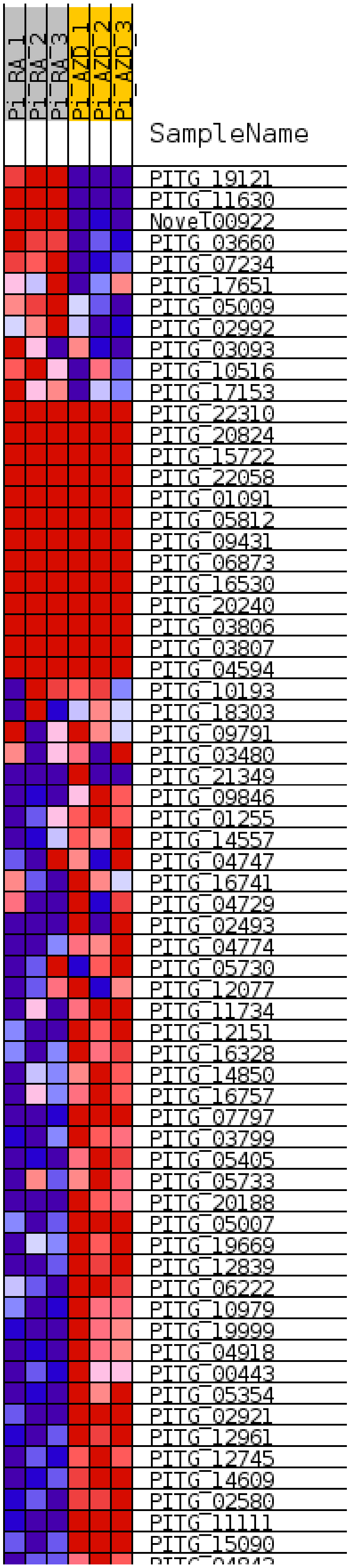

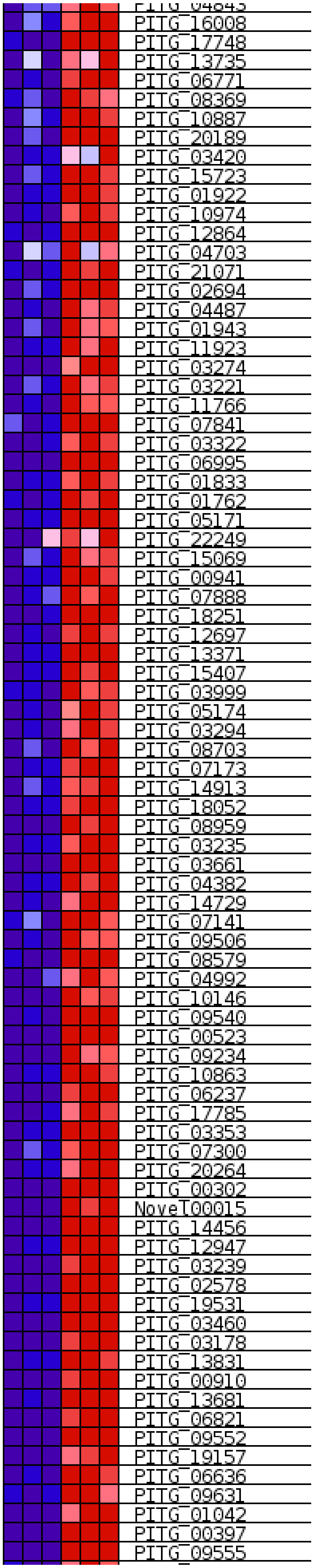
PITG_16008
PITG_17748
PITG_13735
PITG_06771
PITG_08369
PITG_10887
PITG_20189
PITG_03420
PITG_15723
PITG_01922
PITG_10974
PITG_12864
PITG_04703
PITG_21071
PITG_02694
PITG_04487
PITG_01943
PITG_11923
PITG_03274
PITG_03221
PITG_11766
PITG_07841
PITG_03322
PITG_06995
PITG_01833
PITG_01762
PITG_05171
PITG_22249
PITG_15069
PITG_00941
PITG_07888
PITG_18251
PITG_12697
PITG_13371
PITG_15407
PITG_03999
PITG_05174
PITG_03294
PITG_08703
PITG_07173
PITG_14913
PITG_18052
PITG_08959
PITG_03235
PITG_03661
PITG_04382
PITG_14729
PITG_07141
PITG_09506
PITG_08579
PITG_04992
PITG_10146
PITG_09540
PITG_00523
PITG_09234
PITG_10863
PITG_06237
PITG_17785
PITG_03353
PITG_07300
PITG_20264
PITG_00302
Novel00015
PITG_14456
PITG_12947
PITG_03239
PITG_02578
PITG_19531
PITG_03460
PITG_03178
PITG_13831
PITG_00910
PITG_13681
PITG_06821
PITG_09552
PITG_19157
PITG_06636
PITG_09631
PITG_01042
PITG_00397
PITG_09555

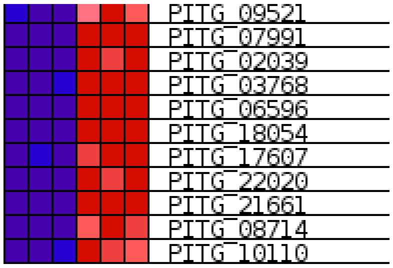

Fig 2: PEPTIDE_BIOSYNTHETIC_PROCESS(GO:0043043)
Blue-Pink O' Gram in the Space of the Analyzed GeneSet

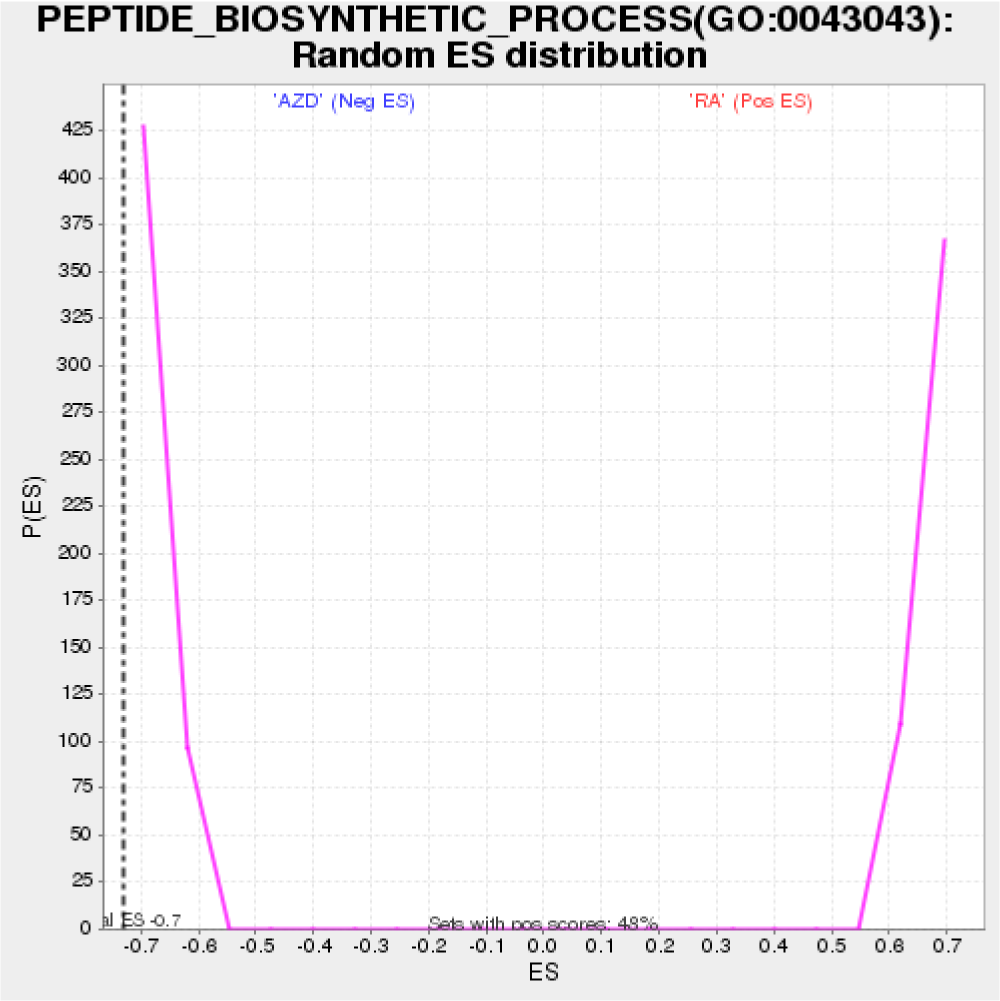

Fig 3: PEPTIDE_BIOSYNTHETIC_PROCESS(GO:0043043): Random ES distribution
Gene set null distribution of ES for PEPTIDE_BIOSYNTHETIC_PROCESS(GO:0043043)

5. peptide metabolic process

Table: GSEA Results Summary

Dataset	fpkm.sample
Phenotype	sample.cls
Upregulated in class	AZD
GeneSet	PEPTIDE_METABOLIC_PROCESS(GO:0006518)
Enrichment Score (ES)	-0.7296995
Normalized Enrichment Score (NES)	-1.0782264
Nominal p-value	0.0
FDR q-value	0.10687023
FWER p-Value	0.056

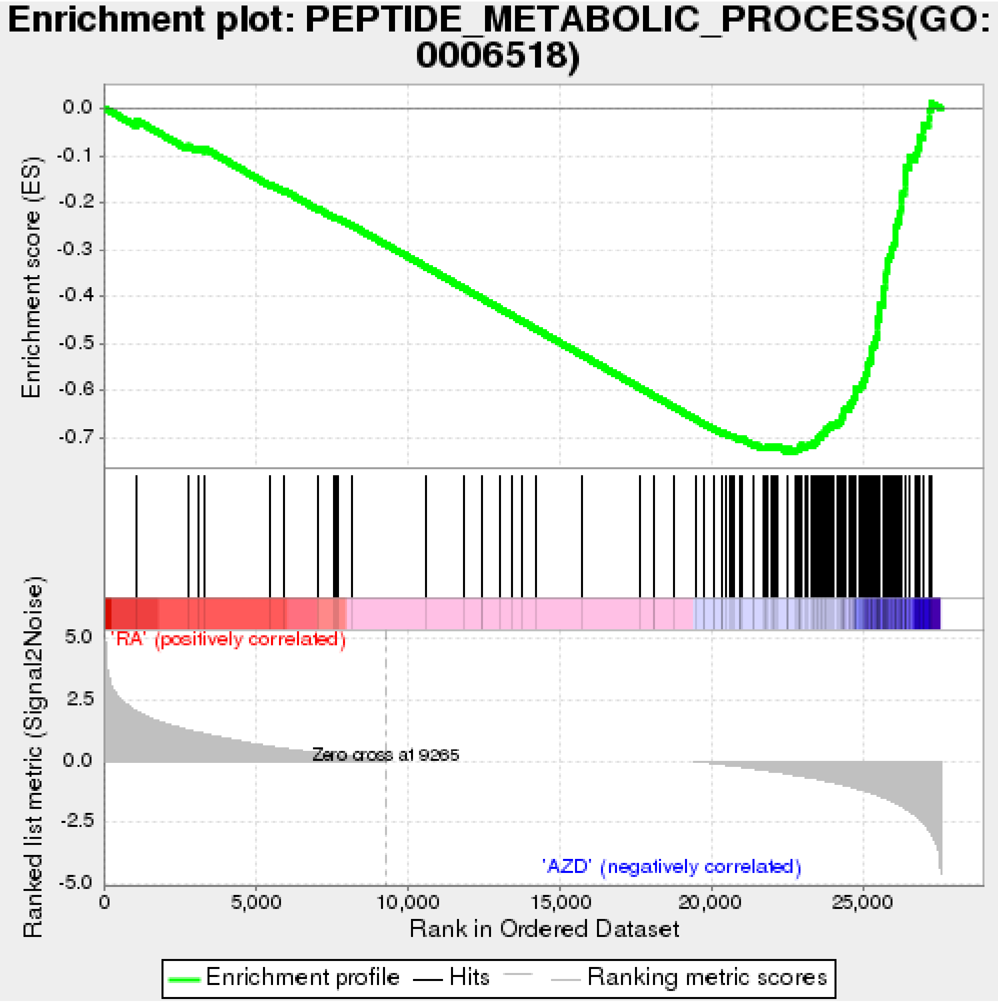

Fig 1: Enrichment plot: PEPTIDE_METABOLIC_PROCESS(GO:0006518)
Profile of the Running ES Score & Positions of GeneSet Members on the Rank Ordered List

Table: GSEA details [plain text format]

	PROBE	DESCRIPTION	GENE	GENE_TITLE	RANK IN	RANK	RUNNING	CORE

		(from dataset)	SYMBOL		GENE LIST	METRIC SCORE	ES	ENRICHMENT
1	PITG_19121	PITG_19121			1014	2.048	-0.0244	No
2	PITG_11630	PITG_11630			2711	1.279	-0.0784	No
3	Novel00922	Novel00922			3038	1.181	-0.0831	No
4	PITG_03660	PITG_03660			3265	1.116	-0.0845	No
5	PITG_07234	PITG_07234			5449	0.602	-0.1604	No
6	PITG_17651	PITG_17651			5906	0.516	-0.1739	No
7	PITG_05009	PITG_05009			7048	0.319	-0.2136	No
8	PITG_02992	PITG_02992			7548	0.243	-0.2303	No
9	PITG_03093	PITG_03093			7622	0.232	-0.2315	No
10	PITG_10516	PITG_10516			7711	0.219	-0.2334	No
11	PITG_17153	PITG_17153			8119	0.169	-0.2472	No
12	PITG_22310	PITG_22310			10562	0.000	-0.3363	No
13	PITG_20824	PITG_20824			11824	0.000	-0.3824	No
14	PITG_15722	PITG_15722			12398	0.000	-0.4033	No
15	PITG_22058	PITG_22058			12412	0.000	-0.4038	No
16	PITG_01091	PITG_01091			13039	0.000	-0.4266	No
17	PITG_05812	PITG_05812			13417	0.000	-0.4404	No
18	PITG_09431	PITG_09431			13717	0.000	-0.4513	No
19	PITG_06873	PITG_06873			14206	0.000	-0.4691	No
20	PITG_16530	PITG_16530			15706	0.000	-0.5238	No
21	PITG_20240	PITG_20240			17604	0.000	-0.5930	No
22	PITG_03806	PITG_03806			18089	0.000	-0.6107	No
23	PITG_03807	PITG_03807			18090	0.000	-0.6107	No
24	PITG_04594	PITG_04594			18776	0.000	-0.6357	No
25	PITG_10193	PITG_10193			19492	-0.015	-0.6617	No
26	PITG_18303	PITG_18303			19751	-0.048	-0.6708	No
27	PITG_09791	PITG_09791			19763	-0.050	-0.6709	No
28	PITG_03480	PITG_03480			20045	-0.095	-0.6806	No
29	PITG_21349	PITG_21349			20105	-0.104	-0.6821	No
30	PITG_09846	PITG_09846			20357	-0.142	-0.6904	No
31	PITG_01255	PITG_01255			20360	-0.142	-0.6896	No
32	PITG_14557	PITG_14557			20366	-0.143	-0.6889	No
33	PITG_04747	PITG_04747			20459	-0.156	-0.6913	No
34	PITG_16741	PITG_16741			20622	-0.179	-0.6961	No
35	PITG_00757	PITG_00757			20661	-0.186	-0.6963	No
36	PITG_04729	PITG_04729			20710	-0.194	-0.6969	No
37	PITG_02493	PITG_02493			20900	-0.221	-0.7024	No
38	PITG_01580	PITG_01580			20920	-0.225	-0.7017	No

39	PITG_04774	PITG_04774			20937	-0.229	-0.7009	No
40	PITG_05730	PITG_05730			21018	-0.243	-0.7023	No
41	PITG_12077	PITG_12077			21406	-0.311	-0.7146	No
42	PITG_11734	PITG_11734			21411	-0.312	-0.7128	No
43	PITG_12151	PITG_12151			21701	-0.361	-0.7211	No
44	PITG_16328	PITG_16328			21756	-0.372	-0.7208	No
45	PITG_14850	PITG_14850			21815	-0.384	-0.7206	No
46	PITG_16757	PITG_16757			21821	-0.385	-0.7184	No
47	PITG_07797	PITG_07797			21968	-0.412	-0.7212	No
48	PITG_03799	PITG_03799			22019	-0.421	-0.7204	No
49	PITG_05405	PITG_05405			22072	-0.429	-0.7197	No
50	PITG_05733	PITG_05733			22091	-0.432	-0.7177	No
51	PITG_20188	PITG_20188			22157	-0.445	-0.7173	No
52	PITG_05007	PITG_05007			22498	-0.509	-0.7266	Yes
53	PITG_19669	PITG_19669			22503	-0.510	-0.7236	Yes
54	PITG_12839	PITG_12839			22518	-0.514	-0.7209	Yes
55	PITG_06222	PITG_06222			22748	-0.566	-0.7258	Yes
56	PITG_10979	PITG_10979			22822	-0.583	-0.7249	Yes
57	PITG_19999	PITG_19999			22878	-0.594	-0.7232	Yes
58	PITG_04918	PITG_04918			22927	-0.602	-0.7213	Yes
59	PITG_00443	PITG_00443			22936	-0.604	-0.7179	Yes
60	PITG_05354	PITG_05354			22976	-0.613	-0.7155	Yes
61	PITG_02921	PITG_02921			23131	-0.650	-0.7171	Yes
62	PITG_12961	PITG_12961			23203	-0.663	-0.7157	Yes
63	PITG_12745	PITG_12745			23280	-0.681	-0.7142	Yes
64	PITG_14609	PITG_14609			23351	-0.695	-0.7125	Yes
65	PITG_02580	PITG_02580			23358	-0.695	-0.7085	Yes
66	PITG_11111	PITG_11111			23461	-0.720	-0.7078	Yes
67	PITG_15090	PITG_15090			23482	-0.725	-0.7040	Yes
68	PITG_04843	PITG_04843			23523	-0.735	-0.7010	Yes
69	PITG_16008	PITG_16008			23545	-0.740	-0.6972	Yes
70	PITG_17748	PITG_17748			23641	-0.765	-0.6959	Yes
71	PITG_13735	PITG_13735			23644	-0.766	-0.6913	Yes
72	PITG_06771	PITG_06771			23729	-0.789	-0.6895	Yes
73	PITG_08369	PITG_08369			23772	-0.802	-0.6861	Yes
74	PITG_10887	PITG_10887			23788	-0.807	-0.6817	Yes
75	PITG_20189	PITG_20189			23835	-0.820	-0.6783	Yes
76	PITG_03420	PITG_03420			23870	-0.829	-0.6745	Yes
77	PITG_15723	PITG_15723			23962	-0.858	-0.6725	Yes

78	PITG_01922	PITG_01922			24019	-0.874	-0.6692	Yes
79	PITG_10974	PITG_10974			24137	-0.910	-0.6679	Yes
80	PITG_12864	PITG_12864			24223	-0.936	-0.6652	Yes
81	PITG_04703	PITG_04703			24269	-0.949	-0.6610	Yes
82	PITG_21071	PITG_21071			24300	-0.958	-0.6562	Yes
83	PITG_02694	PITG_02694			24328	-0.970	-0.6513	Yes
84	PITG_04487	PITG_04487			24365	-0.980	-0.6465	Yes
85	PITG_01943	PITG_01943			24378	-0.982	-0.6409	Yes
86	PITG_11923	PITG_11923			24405	-0.990	-0.6358	Yes
87	PITG_03274	PITG_03274			24545	-1.036	-0.6345	Yes
88	PITG_03221	PITG_03221			24557	-1.040	-0.6285	Yes
89	PITG_11766	PITG_11766			24642	-1.064	-0.6250	Yes
90	PITG_07841	PITG_07841			24668	-1.073	-0.6193	Yes
91	PITG_00754	PITG_00754			24709	-1.085	-0.6141	Yes
92	PITG_03322	PITG_03322			24740	-1.096	-0.6085	Yes
93	PITG_06995	PITG_06995			24760	-1.104	-0.6024	Yes
94	PITG_01833	PITG_01833			24767	-1.106	-0.5958	Yes
95	PITG_01762	PITG_01762			24911	-1.153	-0.5939	Yes
96	PITG_05171	PITG_05171			24921	-1.158	-0.5871	Yes
97	PITG_22249	PITG_22249			25013	-1.195	-0.5831	Yes
98	PITG_15069	PITG_15069			25019	-1.198	-0.5759	Yes
99	PITG_00941	PITG_00941			25067	-1.214	-0.5702	Yes
100	PITG_07888	PITG_07888			25072	-1.216	-0.5628	Yes
101	PITG_18251	PITG_18251			25141	-1.242	-0.5577	Yes
102	PITG_12697	PITG_12697			25164	-1.249	-0.5508	Yes
103	PITG_13371	PITG_13371			25168	-1.249	-0.5432	Yes
104	PITG_15407	PITG_15407			25203	-1.263	-0.5367	Yes
105	PITG_03999	PITG_03999			25257	-1.283	-0.5308	Yes
106	PITG_05174	PITG_05174			25267	-1.288	-0.5232	Yes
107	PITG_03294	PITG_03294			25303	-1.306	-0.5164	Yes
108	PITG_08703	PITG_08703			25308	-1.308	-0.5085	Yes
109	PITG_07173	PITG_07173			25342	-1.325	-0.5016	Yes
110	PITG_14913	PITG_14913			25405	-1.356	-0.4955	Yes
111	PITG_18052	PITG_18052			25424	-1.365	-0.4877	Yes
112	PITG_08959	PITG_08959			25458	-1.380	-0.4805	Yes
113	PITG_03235	PITG_03235			25480	-1.389	-0.4727	Yes
114	PITG_08206	PITG_08206			25481	-1.390	-0.4641	Yes
115	PITG_03661	PITG_03661			25487	-1.391	-0.4558	Yes
116	PITG_04382	PITG_04382			25503	-1.398	-0.4477	Yes

117	PITG_14729	PITG_14729			25528	-1.410	-0.4399	Yes
118	PITG_07141	PITG_07141			25545	-1.420	-0.4318	Yes
119	PITG_09506	PITG_09506			25563	-1.431	-0.4236	Yes
120	PITG_08579	PITG_08579			25572	-1.438	-0.4150	Yes
121	PITG_04992	PITG_04992			25670	-1.484	-0.4094	Yes
122	PITG_10146	PITG_10146			25685	-1.491	-0.4008	Yes
123	PITG_09540	PITG_09540			25692	-1.493	-0.3918	Yes
124	PITG_00523	PITG_00523			25696	-1.497	-0.3827	Yes
125	PITG_09234	PITG_09234			25712	-1.502	-0.3740	Yes
126	PITG_10863	PITG_10863			25713	-1.502	-0.3648	Yes
127	PITG_06237	PITG_06237			25757	-1.523	-0.3570	Yes
128	PITG_17785	PITG_17785			25774	-1.532	-0.3482	Yes
129	PITG_03353	PITG_03353			25780	-1.535	-0.3389	Yes
130	PITG_07300	PITG_07300			25805	-1.550	-0.3302	Yes
131	PITG_20264	PITG_20264			25831	-1.561	-0.3216	Yes
132	PITG_00302	PITG_00302			25872	-1.579	-0.3133	Yes
133	Novel00015	Novel00015			25949	-1.627	-0.3061	Yes
134	PITG_14456	PITG_14456			25952	-1.628	-0.2961	Yes
135	PITG_12947	PITG_12947			26003	-1.658	-0.2878	Yes
136	PITG_03239	PITG_03239			26044	-1.683	-0.2789	Yes
137	PITG_02578	PITG_02578			26070	-1.705	-0.2693	Yes
138	PITG_19531	PITG_19531			26082	-1.713	-0.2592	Yes
139	PITG_03460	PITG_03460			26094	-1.719	-0.2490	Yes
140	PITG_03178	PITG_03178			26113	-1.732	-0.2390	Yes
141	PITG_13831	PITG_13831			26183	-1.780	-0.2306	Yes
142	PITG_00910	PITG_00910			26229	-1.807	-0.2211	Yes
143	PITG_13681	PITG_13681			26238	-1.816	-0.2102	Yes
144	PITG_06821	PITG_06821			26267	-1.833	-0.2000	Yes
145	PITG_09552	PITG_09552			26270	-1.835	-0.1887	Yes
146	PITG_19157	PITG_19157			26301	-1.850	-0.1785	Yes
147	PITG_06636	PITG_06636			26370	-1.898	-0.1693	Yes
148	PITG_09631	PITG_09631			26373	-1.899	-0.1577	Yes
149	PITG_01042	PITG_01042			26385	-1.915	-0.1463	Yes
150	PITG_00397	PITG_00397			26402	-1.929	-0.1350	Yes
151	PITG_09555	PITG_09555			26429	-1.950	-0.1240	Yes
152	PITG_09521	PITG_09521			26522	-2.027	-0.1149	Yes
153	PITG_07991	PITG_07991			26527	-2.030	-0.1025	Yes
154	PITG_02039	PITG_02039			26754	-2.231	-0.0971	Yes
155	PITG_03768	PITG_03768			26780	-2.261	-0.0841	Yes

156	PITG_06596	PITG_06596			26861	-2.351	-0.0725	Yes
157	PITG_18054	PITG_18054			26868	-2.362	-0.0582	Yes
158	PITG_17607	PITG_17607			26969	-2.477	-0.0466	Yes
159	PITG_22020	PITG_22020			27018	-2.545	-0.0327	Yes
160	PITG_21661	PITG_21661			27161	-2.778	-0.0208	Yes
161	PITG_08714	PITG_08714			27196	-2.846	-0.0046	Yes
162	PITG_10110	PITG_10110			27232	-2.902	0.0120	Yes

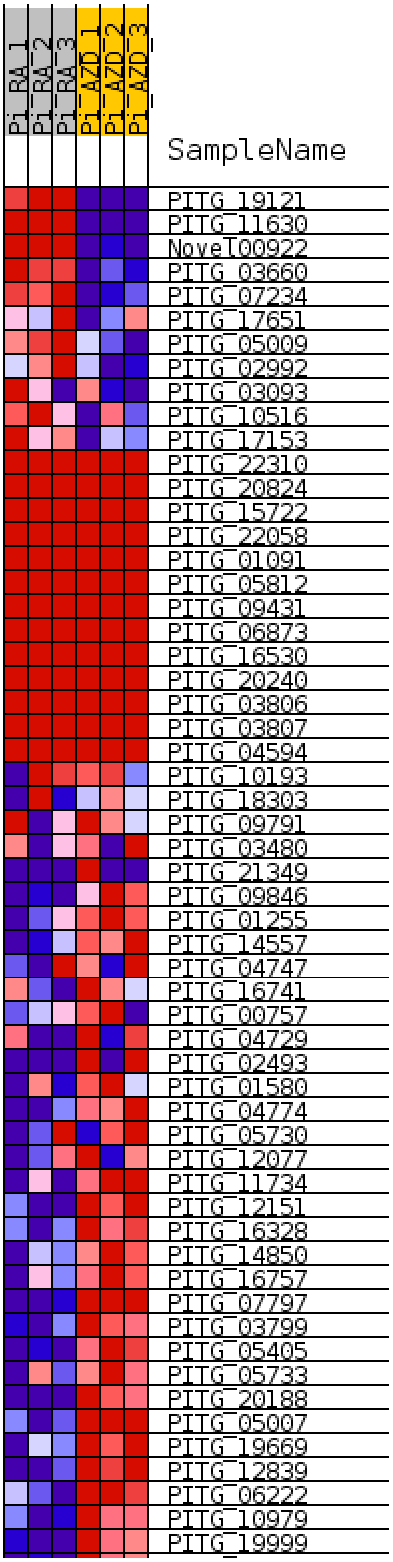

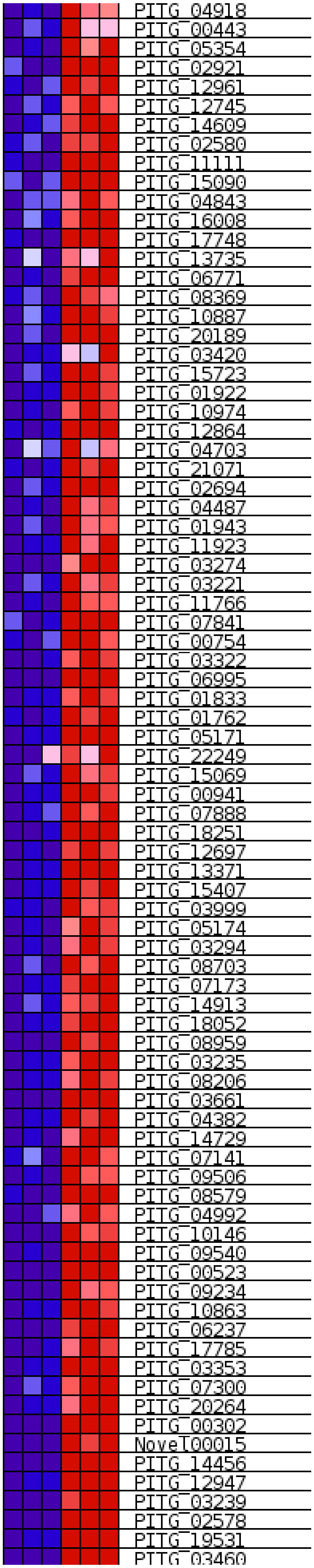
PITG_04918
PITG_00443
PITG_05354
PITG_02921
PITG_12961
PITG_12745
PITG_14609
PITG_02580
PITG_11111
PITG_15090
PITG_04843
PITG_16008
PITG_17748
PITG_13735
PITG_06771
PITG_08369
PITG_10887
PITG_20189
PITG_03420
PITG_15723
PITG_01922
PITG_10974
PITG_12864
PITG_04703
PITG_21071
PITG_02694
PITG_04487
PITG_01943
PITG_11923
PITG_03274
PITG_03221
PITG_11766
PITG_07841
PITG_00754
PITG_03322
PITG_06995
PITG_01833
PITG_01762
PITG_05171
PITG_22249
PITG_15069
PITG_00941
PITG_07888
PITG_18251
PITG_12697
PITG_13371
PITG_15407
PITG_03999
PITG_05174
PITG_03294
PITG_08703
PITG_07173
PITG_14913
PITG_18052
PITG_08959
PITG_03235
PITG_08206
PITG_03661
PITG_04382
PITG_14729
PITG_07141
PITG_09506
PITG_08579
PITG_04992
PITG_10146
PITG_09540
PITG_00523
PITG_09234
PITG_10863
PITG_06237
PITG_17785
PITG_03353
PITG_07300
PITG_20264
PITG_00302
Novel00015
PITG_14456
PITG_12947
PITG_03239
PITG_02578
PITG_19531
PITG_03460

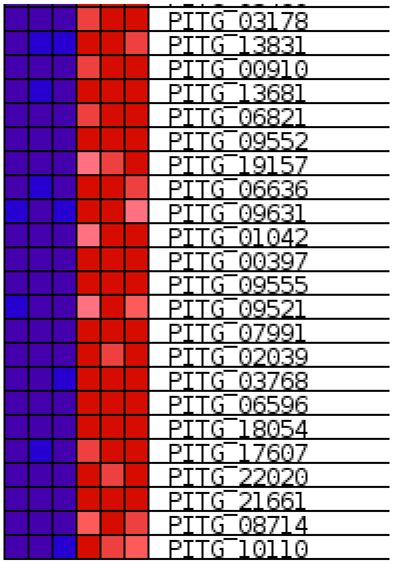

Fig 2: PEPTIDE_METABOLIC_PROCESS(GO:0006518)
Blue-Pink O' Gram in the Space of the Analyzed GeneSet

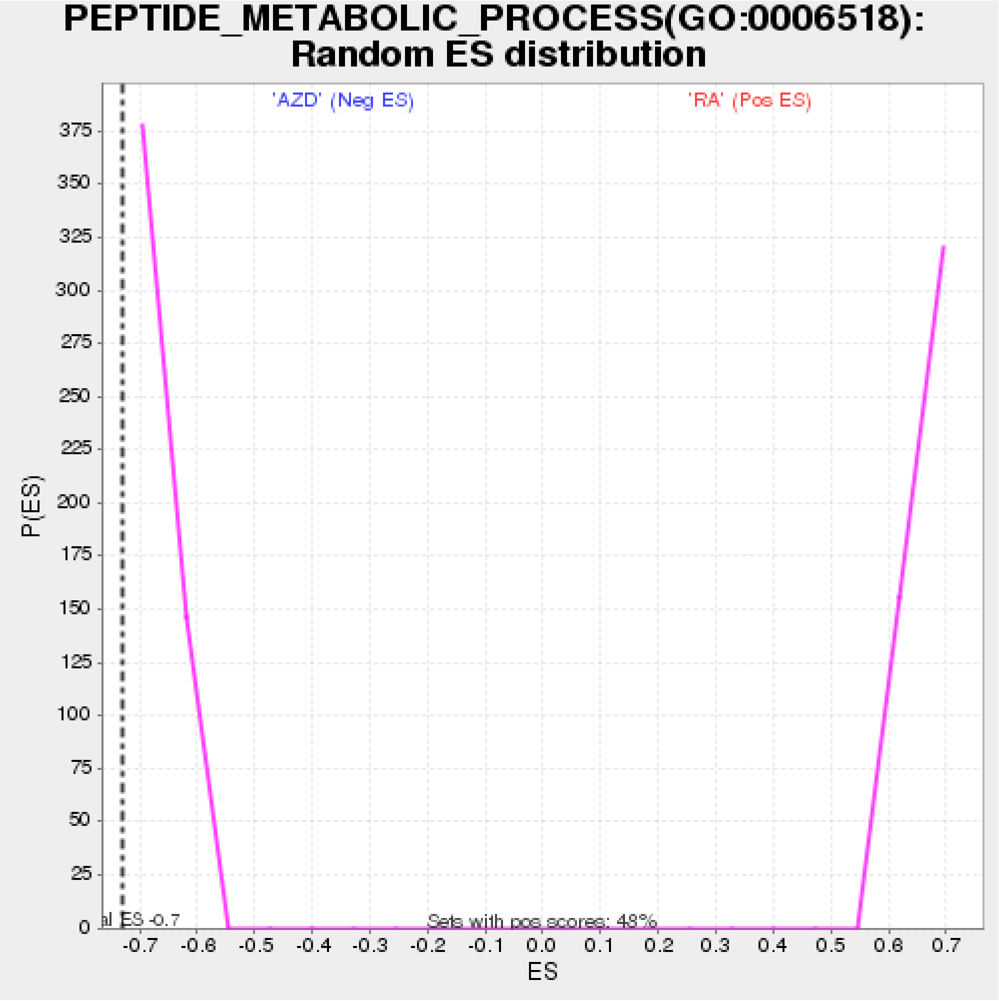

Fig 3: PEPTIDE_METABOLIC_PROCESS(GO:0006518): Random ES distribution
Gene set null distribution of ES for PEPTIDE_METABOLIC_PROCESS(GO:0006518)

6. ribonucleoprotein complex

Table: GSEA Results Summary

Dataset	fpkm.sample
Phenotype	sample.cls
Upregulated in class	AZD
GeneSet	RIBONUCLEOPROTEIN_COMPLEX(GO:1990904)
Enrichment Score (ES)	-0.7274527
Normalized Enrichment Score (NES)	-1.0561713
Nominal p-value	0.0
FDR q-value	0.13096356
FWER p-Value	0.157

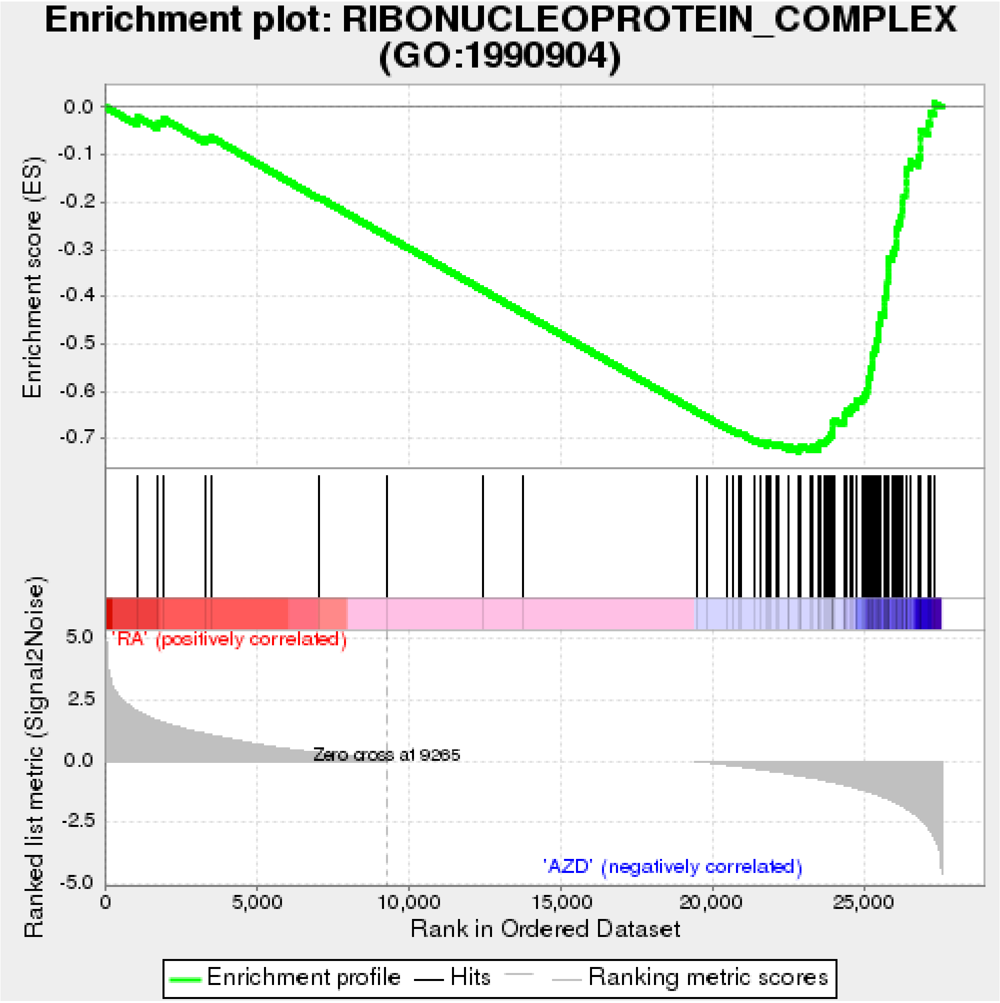

Fig 1: Enrichment plot: RIBONUCLEOPROTEIN_COMPLEX(GO:1990904)
Profile of the Running ES Score & Positions of GeneSet Members on the Rank Ordered List

Table: GSEA details [plain text format]

	PROBE	DESCRIPTION	GENE	GENE_TITLE	RANK IN	RANK	RUNNING	CORE

		(from dataset)	SYMBOL		GENE LIST	METRIC SCORE	ES	ENRICHMENT
1	PITG_19121	PITG_19121			1014	2.048	-0.0196	No
2	PITG_05680	PITG_05680			1713	1.654	-0.0311	No
3	PITG_03040	PITG_03040			1878	1.583	-0.0237	No
4	PITG_03660	PITG_03660			3265	1.116	-0.0648	No
5	PITG_01925	PITG_01925			3455	1.062	-0.0627	No
6	PITG_05009	PITG_05009			7048	0.319	-0.1908	No
7	PITG_10482	PITG_10482			9256	0.002	-0.2712	No
8	PITG_15722	PITG_15722			12398	0.000	-0.3856	No
9	PITG_09431	PITG_09431			13717	0.000	-0.4335	No
10	PITG_10193	PITG_10193			19492	-0.015	-0.6437	No
11	PITG_12932	PITG_12932			19783	-0.055	-0.6538	No
12	PITG_03818	PITG_03818			20478	-0.160	-0.6777	No
13	PITG_04395	PITG_04395			20690	-0.190	-0.6838	No
14	PITG_10725	PITG_10725			20836	-0.211	-0.6873	No
15	PITG_17166	PITG_17166			20957	-0.232	-0.6897	No
16	PITG_11734	PITG_11734			21411	-0.312	-0.7036	No
17	PITG_17733	PITG_17733			21594	-0.341	-0.7073	No
18	PITG_14850	PITG_14850			21815	-0.384	-0.7121	No
19	PITG_16757	PITG_16757			21821	-0.385	-0.7090	No
20	PITG_01246	PITG_01246			21937	-0.406	-0.7098	No
21	PITG_17727	PITG_17727			22139	-0.441	-0.7134	No
22	PITG_20188	PITG_20188			22157	-0.445	-0.7102	No
23	PITG_19669	PITG_19669			22503	-0.510	-0.7185	No
24	PITG_12839	PITG_12839			22518	-0.514	-0.7147	No
25	PITG_14854	PITG_14854			22870	-0.592	-0.7225	Yes
26	PITG_19999	PITG_19999			22878	-0.594	-0.7177	Yes
27	PITG_00443	PITG_00443			22936	-0.604	-0.7147	Yes
28	PITG_14730	PITG_14730			23216	-0.668	-0.7192	Yes
29	PITG_12745	PITG_12745			23280	-0.681	-0.7158	Yes
30	PITG_15090	PITG_15090			23482	-0.725	-0.7170	Yes
31	PITG_04843	PITG_04843			23523	-0.735	-0.7122	Yes
32	PITG_16008	PITG_16008			23545	-0.740	-0.7067	Yes
33	PITG_06771	PITG_06771			23729	-0.789	-0.7067	Yes
34	PITG_10887	PITG_10887			23788	-0.807	-0.7021	Yes
35	PITG_20189	PITG_20189			23835	-0.820	-0.6968	Yes
36	PITG_03420	PITG_03420			23870	-0.829	-0.6911	Yes
37	Novel01297	Novel01297			23960	-0.857	-0.6871	Yes
38	PITG_15723	PITG_15723			23962	-0.858	-0.6799	Yes

39	PITG_19007	PITG_19007			23986	-0.865	-0.6734	Yes
40	PITG_02996	PITG_02996			23987	-0.865	-0.6661	Yes
41	PITG_01922	PITG_01922			24019	-0.874	-0.6599	Yes
42	PITG_02694	PITG_02694			24328	-0.970	-0.6629	Yes
43	PITG_04487	PITG_04487			24365	-0.980	-0.6560	Yes
44	PITG_01943	PITG_01943			24378	-0.982	-0.6481	Yes
45	PITG_11923	PITG_11923			24405	-0.990	-0.6407	Yes
46	PITG_03221	PITG_03221			24557	-1.040	-0.6374	Yes
47	PITG_11766	PITG_11766			24642	-1.064	-0.6315	Yes
48	PITG_06995	PITG_06995			24760	-1.104	-0.6265	Yes
49	PITG_01833	PITG_01833			24767	-1.106	-0.6173	Yes
50	PITG_05171	PITG_05171			24921	-1.158	-0.6131	Yes
51	PITG_15069	PITG_15069			25019	-1.198	-0.6066	Yes
52	PITG_00941	PITG_00941			25067	-1.214	-0.5980	Yes
53	PITG_01171	PITG_01171			25118	-1.234	-0.5895	Yes
54	PITG_12697	PITG_12697			25164	-1.249	-0.5806	Yes
55	PITG_13371	PITG_13371			25168	-1.249	-0.5701	Yes
56	PITG_15407	PITG_15407			25203	-1.263	-0.5607	Yes
57	PITG_12305	PITG_12305			25229	-1.272	-0.5509	Yes
58	PITG_05174	PITG_05174			25267	-1.288	-0.5414	Yes
59	PITG_03294	PITG_03294			25303	-1.306	-0.5316	Yes
60	PITG_08703	PITG_08703			25308	-1.308	-0.5208	Yes
61	PITG_07173	PITG_07173			25342	-1.325	-0.5108	Yes
62	PITG_14913	PITG_14913			25405	-1.356	-0.5016	Yes
63	PITG_18052	PITG_18052			25424	-1.365	-0.4907	Yes
64	PITG_08959	PITG_08959			25458	-1.380	-0.4803	Yes
65	PITG_03235	PITG_03235			25480	-1.389	-0.4693	Yes
66	PITG_04382	PITG_04382			25503	-1.398	-0.4583	Yes
67	PITG_14729	PITG_14729			25528	-1.410	-0.4473	Yes
68	PITG_09506	PITG_09506			25563	-1.431	-0.4365	Yes
69	PITG_10146	PITG_10146			25685	-1.491	-0.4283	Yes
70	PITG_09540	PITG_09540			25692	-1.493	-0.4159	Yes
71	PITG_00523	PITG_00523			25696	-1.497	-0.4034	Yes
72	PITG_10863	PITG_10863			25713	-1.502	-0.3913	Yes
73	PITG_06237	PITG_06237			25757	-1.523	-0.3801	Yes
74	PITG_17785	PITG_17785			25774	-1.532	-0.3677	Yes
75	PITG_03353	PITG_03353			25780	-1.535	-0.3550	Yes
76	PITG_07300	PITG_07300			25805	-1.550	-0.3428	Yes
77	PITG_12292	PITG_12292			25812	-1.552	-0.3299	Yes

78	PITG_20264	PITG_20264			25831	-1.561	-0.3174	Yes
79	Novel00015	Novel00015			25949	-1.627	-0.3079	Yes
80	PITG_12947	PITG_12947			26003	-1.658	-0.2959	Yes
81	PITG_03239	PITG_03239			26044	-1.683	-0.2831	Yes
82	PITG_02578	PITG_02578			26070	-1.705	-0.2696	Yes
83	PITG_19531	PITG_19531			26082	-1.713	-0.2556	Yes
84	PITG_03178	PITG_03178			26113	-1.732	-0.2421	Yes
85	PITG_01019	PITG_01019			26205	-1.792	-0.2303	Yes
86	PITG_13681	PITG_13681			26238	-1.816	-0.2161	Yes
87	PITG_09552	PITG_09552			26270	-1.835	-0.2018	Yes
88	PITG_19157	PITG_19157			26301	-1.850	-0.1873	Yes
89	PITG_06636	PITG_06636			26370	-1.898	-0.1737	Yes
90	PITG_09631	PITG_09631			26373	-1.899	-0.1578	Yes
91	PITG_01042	PITG_01042			26385	-1.915	-0.1420	Yes
92	PITG_09555	PITG_09555			26429	-1.950	-0.1271	Yes
93	PITG_09521	PITG_09521			26522	-2.027	-0.1134	Yes
94	PITG_03768	PITG_03768			26780	-2.261	-0.1037	Yes
95	PITG_06596	PITG_06596			26861	-2.351	-0.0868	Yes
96	PITG_18054	PITG_18054			26868	-2.362	-0.0671	Yes
97	PITG_12931	PITG_12931			26872	-2.368	-0.0472	Yes
98	PITG_02593	PITG_02593			27105	-2.686	-0.0330	Yes
99	PITG_17365	PITG_17365			27192	-2.839	-0.0122	Yes
100	PITG_02592	PITG_02592			27299	-3.031	0.0095	Yes

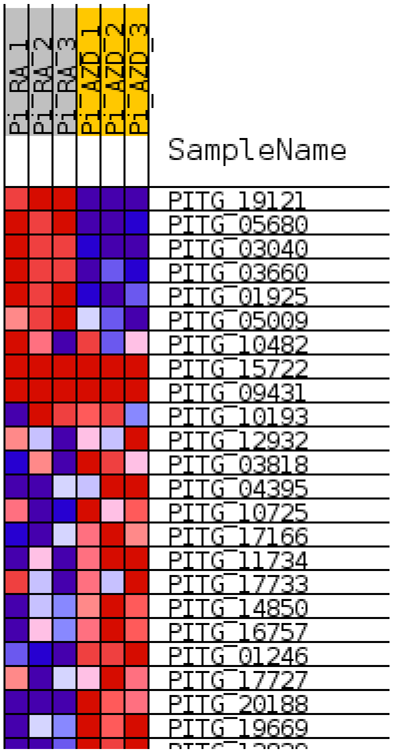

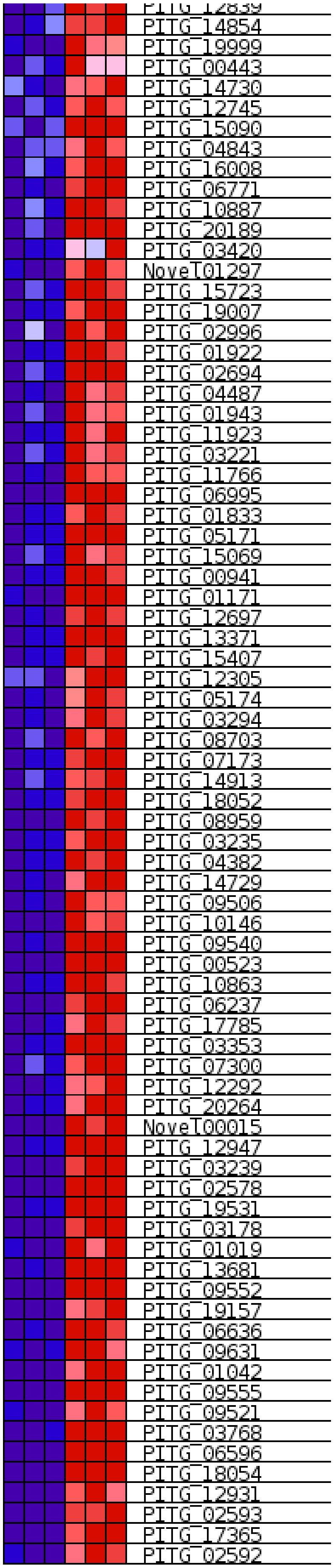

Fig 2: RIBONUCLEOPROTEIN_COMPLEX(GO:1990904)
Blue-Pink O' Gram in the Space of the Analyzed GeneSet

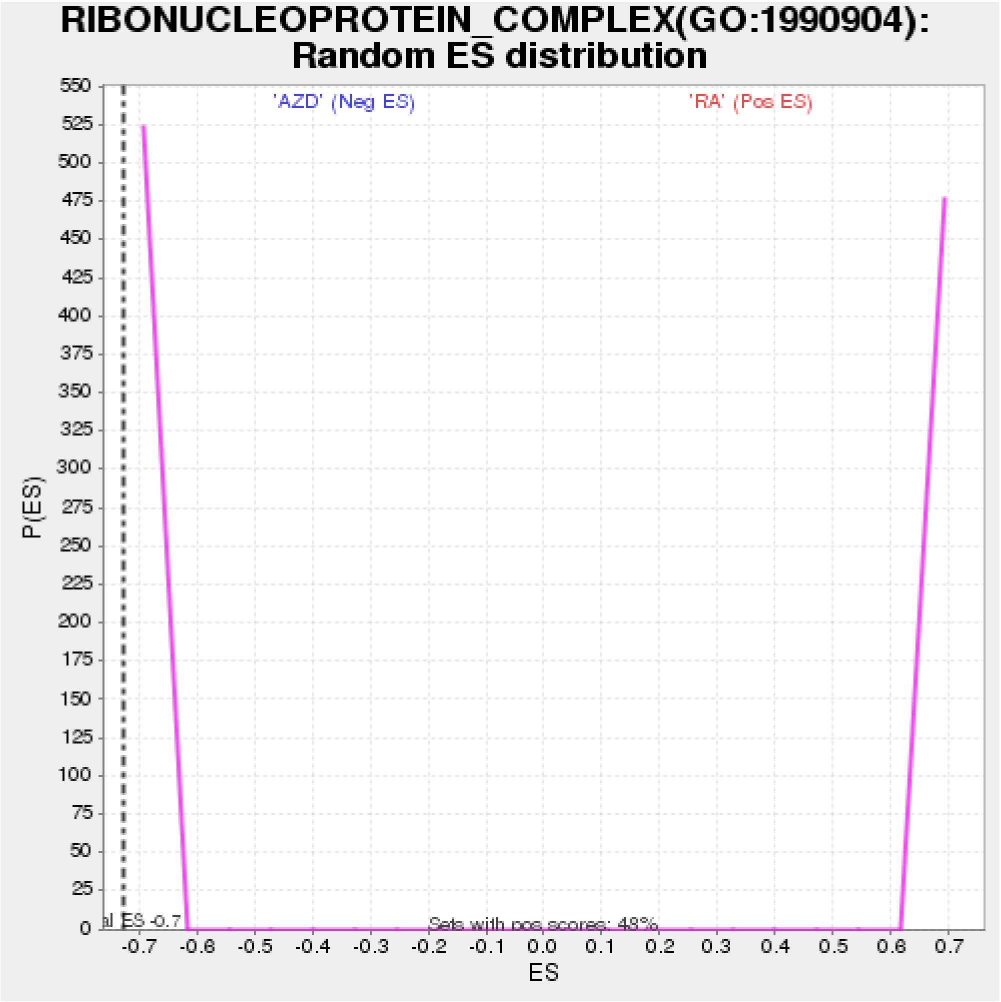

Fig 3: RIBONUCLEOPROTEIN_COMPLEX(GO:1990904): Random ES distribution
Gene set null distribution of ES for RIBONUCLEOPROTEIN_COMPLEX(GO:1990904)

7. Ribosome

Table: GSEA Results Summary

Dataset	fpkm.sample
Phenotype	sample.cls
Upregulated in class	AZD
GeneSet	RIBOSOME(GO:0005840)
Enrichment Score (ES)	-0.76810294
Normalized Enrichment Score (NES)	-1.0443331
Nominal p-value	0.0
FDR q-value	0.15763332
FWER p-Value	0.264

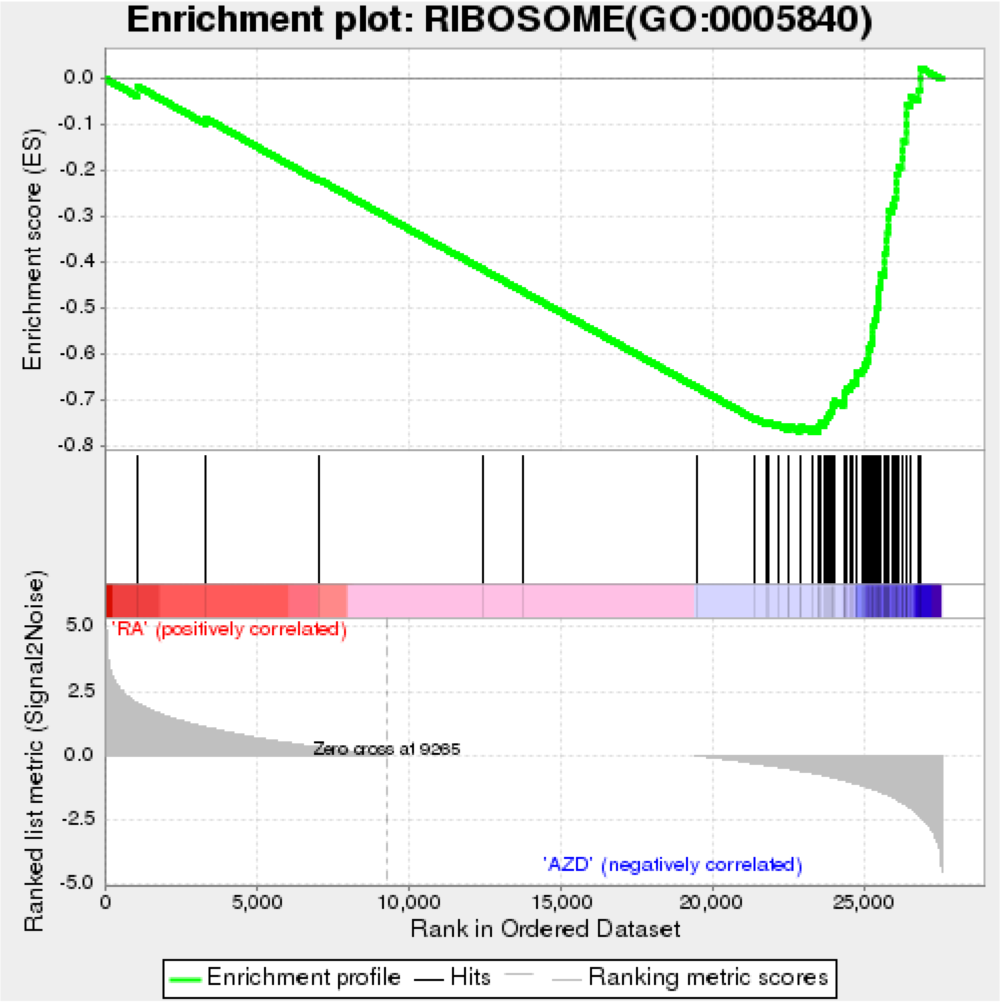

Fig 1: Enrichment plot: RIBOSOME(GO:0005840)
Profile of the Running ES Score & Positions of GeneSet Members on the Rank Ordered List

Table: GSEA details [plain text format]

	PROBE	DESCRIPTION	GENE	GENE_TITLE	RANK IN	RANK	RUNNING	CORE

		(from dataset)	SYMBOL		GENE LIST	METRIC SCORE	ES	ENRICHMENT
1	PITG_19121	PITG_19121			1014	2.048	-0.0147	No
2	PITG_03660	PITG_03660			3265	1.116	-0.0845	No
3	PITG_05009	PITG_05009			7048	0.319	-0.2187	No
4	PITG_15722	PITG_15722			12398	0.000	-0.4133	No
5	PITG_09431	PITG_09431			13717	0.000	-0.4612	No
6	PITG_10193	PITG_10193			19492	-0.015	-0.6712	No
7	PITG_11734	PITG_11734			21411	-0.312	-0.7376	No
8	PITG_14850	PITG_14850			21815	-0.384	-0.7481	No
9	PITG_16757	PITG_16757			21821	-0.385	-0.7441	No
10	PITG_20188	PITG_20188			22157	-0.445	-0.7515	No
11	PITG_19669	PITG_19669			22503	-0.510	-0.7585	No
12	PITG_12839	PITG_12839			22518	-0.514	-0.7534	No
13	PITG_19999	PITG_19999			22878	-0.594	-0.7601	No
14	PITG_00443	PITG_00443			22936	-0.604	-0.7556	No
15	PITG_12745	PITG_12745			23280	-0.681	-0.7607	Yes
16	PITG_15090	PITG_15090			23482	-0.725	-0.7602	Yes
17	PITG_04843	PITG_04843			23523	-0.735	-0.7537	Yes
18	PITG_16008	PITG_16008			23545	-0.740	-0.7465	Yes
19	PITG_06771	PITG_06771			23729	-0.789	-0.7446	Yes
20	PITG_10887	PITG_10887			23788	-0.807	-0.7380	Yes
21	PITG_20189	PITG_20189			23835	-0.820	-0.7308	Yes
22	PITG_03420	PITG_03420			23870	-0.829	-0.7231	Yes
23	PITG_15723	PITG_15723			23962	-0.858	-0.7171	Yes
24	PITG_19007	PITG_19007			23986	-0.865	-0.7086	Yes
25	PITG_01922	PITG_01922			24019	-0.874	-0.7003	Yes
26	PITG_02694	PITG_02694			24328	-0.970	-0.7010	Yes
27	PITG_04487	PITG_04487			24365	-0.980	-0.6917	Yes
28	PITG_01943	PITG_01943			24378	-0.982	-0.6815	Yes
29	PITG_11923	PITG_11923			24405	-0.990	-0.6718	Yes
30	PITG_03221	PITG_03221			24557	-1.040	-0.6660	Yes
31	PITG_11766	PITG_11766			24642	-1.064	-0.6576	Yes
32	PITG_06995	PITG_06995			24760	-1.104	-0.6499	Yes
33	PITG_01833	PITG_01833			24767	-1.106	-0.6381	Yes
34	PITG_05171	PITG_05171			24921	-1.158	-0.6312	Yes
35	PITG_15069	PITG_15069			25019	-1.198	-0.6217	Yes
36	PITG_00941	PITG_00941			25067	-1.214	-0.6103	Yes
37	PITG_12697	PITG_12697			25164	-1.249	-0.6003	Yes
38	PITG_13371	PITG_13371			25168	-1.249	-0.5869	Yes

39	PITG_15407	PITG_15407			25203	-1.263	-0.5745	Yes
40	PITG_05174	PITG_05174			25267	-1.288	-0.5628	Yes
41	PITG_03294	PITG_03294			25303	-1.306	-0.5500	Yes
42	PITG_08703	PITG_08703			25308	-1.308	-0.5360	Yes
43	PITG_07173	PITG_07173			25342	-1.325	-0.5229	Yes
44	PITG_14913	PITG_14913			25405	-1.356	-0.5104	Yes
45	PITG_18052	PITG_18052			25424	-1.365	-0.4963	Yes
46	PITG_08959	PITG_08959			25458	-1.380	-0.4826	Yes
47	PITG_03235	PITG_03235			25480	-1.389	-0.4683	Yes
48	PITG_04382	PITG_04382			25503	-1.398	-0.4540	Yes
49	PITG_14729	PITG_14729			25528	-1.410	-0.4396	Yes
50	PITG_09506	PITG_09506			25563	-1.431	-0.4254	Yes
51	PITG_10146	PITG_10146			25685	-1.491	-0.4137	Yes
52	PITG_09540	PITG_09540			25692	-1.493	-0.3977	Yes
53	PITG_00523	PITG_00523			25696	-1.497	-0.3817	Yes
54	PITG_10863	PITG_10863			25713	-1.502	-0.3660	Yes
55	PITG_06237	PITG_06237			25757	-1.523	-0.3511	Yes
56	PITG_17785	PITG_17785			25774	-1.532	-0.3351	Yes
57	PITG_03353	PITG_03353			25780	-1.535	-0.3187	Yes
58	PITG_07300	PITG_07300			25805	-1.550	-0.3028	Yes
59	PITG_20264	PITG_20264			25831	-1.561	-0.2868	Yes
60	Novel00015	Novel00015			25949	-1.627	-0.2735	Yes
61	PITG_12947	PITG_12947			26003	-1.658	-0.2575	Yes
62	PITG_03239	PITG_03239			26044	-1.683	-0.2407	Yes
63	PITG_02578	PITG_02578			26070	-1.705	-0.2232	Yes
64	PITG_19531	PITG_19531			26082	-1.713	-0.2051	Yes
65	PITG_03178	PITG_03178			26113	-1.732	-0.1874	Yes
66	PITG_13681	PITG_13681			26238	-1.816	-0.1723	Yes
67	PITG_09552	PITG_09552			26270	-1.835	-0.1536	Yes
68	PITG_19157	PITG_19157			26301	-1.850	-0.1347	Yes
69	PITG_06636	PITG_06636			26370	-1.898	-0.1166	Yes
70	PITG_09631	PITG_09631			26373	-1.899	-0.0961	Yes
71	PITG_01042	PITG_01042			26385	-1.915	-0.0758	Yes
72	PITG_09555	PITG_09555			26429	-1.950	-0.0563	Yes
73	PITG_09521	PITG_09521			26522	-2.027	-0.0377	Yes
74	PITG_03768	PITG_03768			26780	-2.261	-0.0226	Yes
75	PITG_06596	PITG_06596			26861	-2.351	-0.0001	Yes
76	PITG_18054	PITG_18054			26868	-2.362	0.0252	Yes

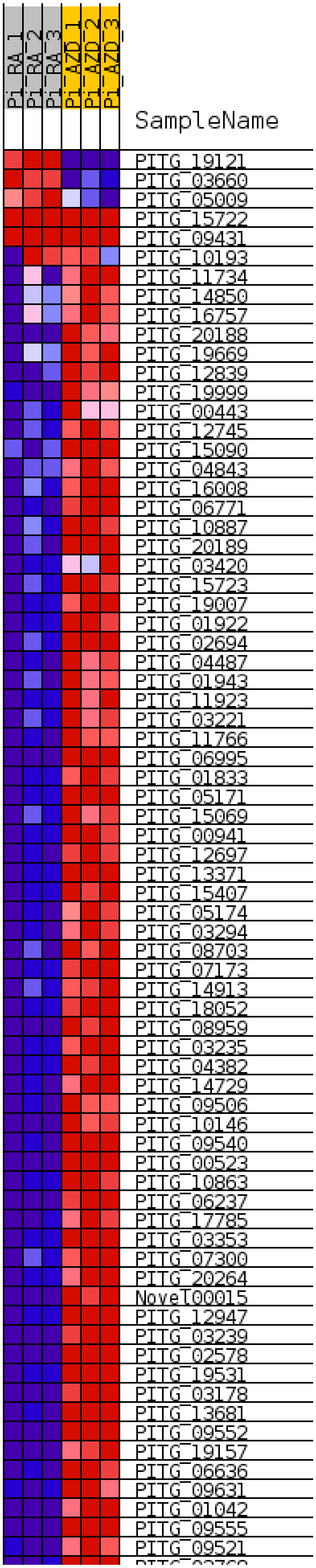
Pi_RA_1
Pi_RA_2
Pi_RA_3
Pi_AZD_1
Pi_AZD_2
Pi_AZD_3
SampleName
PITG_19121
PITG_03660
PITG_05009
PITG_15722
PITG_09431
PITG_10193
PITG_11734
PITG_14850
PITG_16757
PITG_20188
PITG_19669
PITG_12839
PITG_19999
PITG_00443
PITG_12745
PITG_15090
PITG_04843
PITG_16008
PITG_06771
PITG_10887
PITG_20189
PITG_03420
PITG_15723
PITG_19007
PITG_01922
PITG_02694
PITG_04487
PITG_01943
PITG_11923
PITG_03221
PITG_11766
PITG_06995
PITG_01833
PITG_05171
PITG_15069
PITG_00941
PITG_12697
PITG_13371
PITG_15407
PITG_05174
PITG_03294
PITG_08703
PITG_07173
PITG_14913
PITG_18052
PITG_08959
PITG_03235
PITG_04382
PITG_14729
PITG_09506
PITG_10146
PITG_09540
PITG_00523
PITG_10863
PITG_06237
PITG_17785
PITG_03353
PITG_07300
PITG_20264
Novel00015
PITG_12947
PITG_03239
PITG_02578
PITG_19531
PITG_03178
PITG_13681
PITG_09552
PITG_19157
PITG_06636
PITG_09631
PITG_01042
PITG_09555
PITG_09521

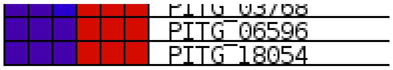

Fig 2: RIBOSOME(GO:0005840)
lue-Pink O' Gram in the Space of the Analyzed GeneSet

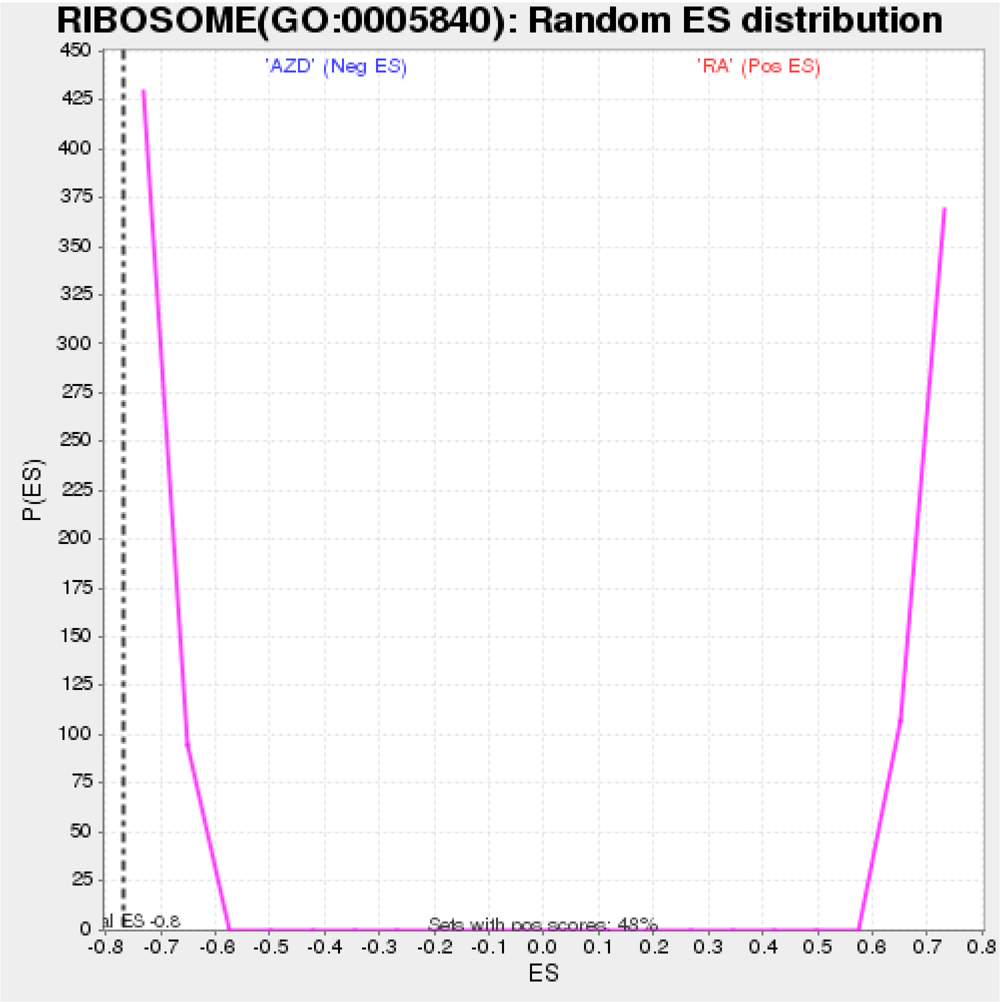

Fig 3: RIBOSOME(GO:0005840): Random ES distribution
Gene set null distribution of ES for RIBOSOME(GO:0005840)

8. RNA binding

Table: GSEA Results Summary

Dataset	fpkm.sample
Phenotype	sample.cls
Upregulated in class	AZD
GeneSet	RNA_BINDING(GO:0003723)
Enrichment Score (ES)	-0.5640048
Normalized Enrichment Score (NES)	-1.2147759
Nominal p-value	0.0
FDR q-value	0.10687017
FWER p-Value	0.0

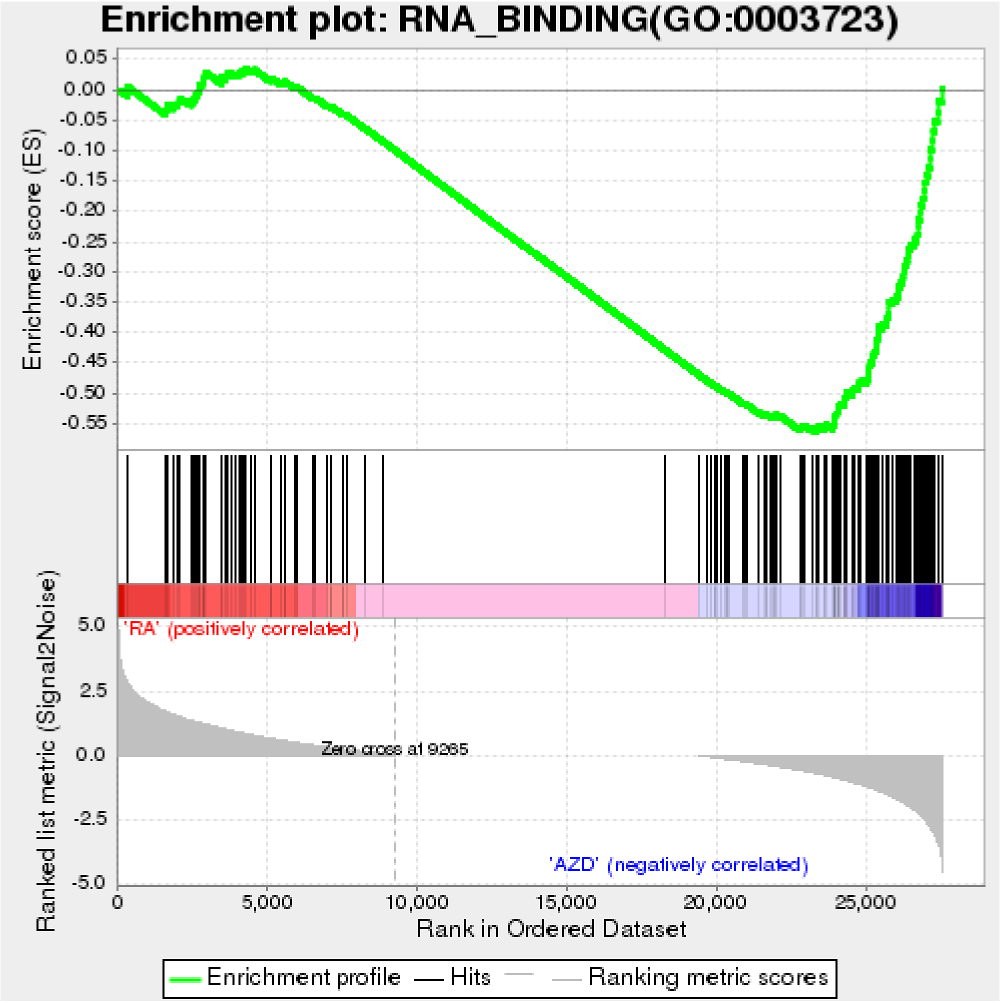

Fig 1: Enrichment plot: RNA_BINDING(GO:0003723)
Profile of the Running ES Score & Positions of GeneSet Members on the Rank Ordered List

Table: GSEA details [plain text format]

	PROBE	DESCRIPTION	GENE	GENE_TITLE	RANK IN	RANK	RUNNING	CORE

		(from dataset)	SYMBOL		GENE LIST	METRIC SCORE	ES	ENRICHMENT
1	PITG_08597	PITG_08597			316	2.812	0.0044	No
2	PITG_17224	PITG_17224			1604	1.694	-0.0329	No
3	PITG_06886	PITG_06886			1632	1.684	-0.0243	No
4	PITG_04376	PITG_04376			1867	1.587	-0.0239	No
5	PITG_16523	PITG_16523			2005	1.527	-0.0202	No
6	PITG_00483	PITG_00483			2069	1.501	-0.0140	No
7	PITG_11433	PITG_11433			2409	1.376	-0.0185	No
8	PITG_16519	PITG_16519			2534	1.334	-0.0155	No
9	PITG_23106	PITG_23106			2575	1.321	-0.0095	No
10	PITG_07100	PITG_07100			2655	1.295	-0.0050	No
11	PITG_17407	PITG_17407			2691	1.285	0.0010	No
12	PITG_11630	PITG_11630			2711	1.279	0.0076	No
13	PITG_16517	PITG_16517			2809	1.247	0.0111	No
14	PITG_16378	PITG_16378			2828	1.243	0.0175	No
15	PITG_10574	PITG_10574			2873	1.230	0.0229	No
16	PITG_16524	PITG_16524			2933	1.211	0.0276	No
17	PITG_12486	PITG_12486			3454	1.062	0.0147	No
18	PITG_01925	PITG_01925			3455	1.062	0.0207	No
19	PITG_17222	PITG_17222			3596	1.023	0.0214	No
20	PITG_12355	PITG_12355			3616	1.019	0.0265	No
21	PITG_18633	PITG_18633			3754	0.984	0.0271	No
22	PITG_08211	PITG_08211			3929	0.937	0.0260	No
23	PITG_17964	PITG_17964			4016	0.917	0.0281	No
24	PITG_10857	PITG_10857			4110	0.893	0.0298	No
25	PITG_03118	PITG_03118			4210	0.871	0.0311	No
26	PITG_16516	PITG_16516			4217	0.870	0.0358	No
27	PITG_06546	PITG_06546			4453	0.813	0.0319	No
28	PITG_09292	PITG_09292			4546	0.790	0.0330	No
29	PITG_13146	PITG_13146			5092	0.673	0.0169	No
30	PITG_10167	PITG_10167			5137	0.665	0.0191	No
31	PITG_07234	PITG_07234			5449	0.602	0.0112	No
32	PITG_16369	PITG_16369			5554	0.583	0.0107	No
33	PITG_12487	PITG_12487			5572	0.578	0.0133	No
34	PITG_17651	PITG_17651			5906	0.516	0.0041	No
35	PITG_16522	PITG_16522			5987	0.503	0.0041	No
36	PITG_17225	PITG_17225			6524	0.409	-0.0132	No
37	PITG_20903	PITG_20903			6592	0.397	-0.0134	No
38	PITG_16012	PITG_16012			6997	0.327	-0.0262	No

39	PITG_16521	PITG_16521			7139	0.306	-0.0297	No
40	PITG_11369	PITG_11369			7140	0.306	-0.0279	No
41	PITG_17930	PITG_17930			7545	0.243	-0.0413	No
42	PITG_02992	PITG_02992			7548	0.243	-0.0400	No
43	PITG_03093	PITG_03093			7622	0.232	-0.0413	No
44	PITG_06610	PITG_06610			8256	0.148	-0.0636	No
45	PITG_05536	PITG_05536			8865	0.058	-0.0854	No
46	PITG_17228	PITG_17228			18290	0.000	-0.4293	No
47	PITG_12797	PITG_12797			19400	-0.001	-0.4697	No
48	PITG_13806	PITG_13806			19695	-0.040	-0.4802	No
49	PITG_13500	PITG_13500			19785	-0.055	-0.4832	No
50	PITG_08684	PITG_08684			19922	-0.077	-0.4877	No
51	PITG_10481	PITG_10481			19923	-0.077	-0.4873	No
52	PITG_03480	PITG_03480			20045	-0.095	-0.4911	No
53	PITG_08831	PITG_08831			20140	-0.109	-0.4939	No
54	PITG_04567	PITG_04567			20295	-0.132	-0.4988	No
55	PITG_01255	PITG_01255			20360	-0.142	-0.5003	No
56	PITG_14557	PITG_14557			20366	-0.143	-0.4997	No
57	PITG_10969	PITG_10969			20448	-0.155	-0.5018	No
58	PITG_02493	PITG_02493			20900	-0.221	-0.5170	No
59	PITG_01846	PITG_01846			20964	-0.233	-0.5180	No
60	PITG_04538	PITG_04538			21042	-0.247	-0.5194	No
61	PITG_03778	PITG_03778			21427	-0.314	-0.5316	No
62	PITG_17733	PITG_17733			21594	-0.341	-0.5357	No
63	PITG_14278	PITG_14278			21692	-0.359	-0.5372	No
64	PITG_16525	PITG_16525			21695	-0.360	-0.5352	No
65	PITG_01045	PITG_01045			21818	-0.384	-0.5375	No
66	PITG_09243	PITG_09243			21892	-0.398	-0.5379	No
67	PITG_01246	PITG_01246			21937	-0.406	-0.5372	No
68	PITG_07797	PITG_07797			21968	-0.412	-0.5360	No
69	PITG_03799	PITG_03799			22019	-0.421	-0.5354	No
70	PITG_17727	PITG_17727			22139	-0.441	-0.5373	No
71	PITG_10979	PITG_10979			22822	-0.583	-0.5588	No
72	PITG_10201	PITG_10201			22838	-0.585	-0.5561	No
73	PITG_14854	PITG_14854			22870	-0.592	-0.5538	No
74	PITG_05366	PITG_05366			22984	-0.616	-0.5545	No
75	PITG_15393	PITG_15393			23201	-0.663	-0.5586	No
76	PITG_14609	PITG_14609			23351	-0.695	-0.5601	Yes
77	PITG_02580	PITG_02580			23358	-0.695	-0.5563	Yes

78	PITG_00967	PITG_00967			23427	-0.711	-0.5548	Yes
79	PITG_01036	PITG_01036			23631	-0.764	-0.5579	Yes
80	PITG_13735	PITG_13735			23644	-0.766	-0.5539	Yes
81	PITG_18184	PITG_18184			23702	-0.782	-0.5516	Yes
82	PITG_18998	PITG_18998			23917	-0.844	-0.5546	Yes
83	PITG_09563	PITG_09563			23937	-0.851	-0.5505	Yes
84	Novel01297	Novel01297			23960	-0.857	-0.5464	Yes
85	PITG_09987	PITG_09987			23978	-0.863	-0.5421	Yes
86	PITG_02996	PITG_02996			23987	-0.865	-0.5375	Yes
87	PITG_00462	PITG_00462			23998	-0.868	-0.5329	Yes
88	PITG_04079	PITG_04079			24062	-0.889	-0.5302	Yes
89	PITG_21196	PITG_21196			24068	-0.891	-0.5253	Yes
90	PITG_04666	PITG_04666			24084	-0.895	-0.5208	Yes
91	PITG_10974	PITG_10974			24137	-0.910	-0.5175	Yes
92	PITG_04703	PITG_04703			24269	-0.949	-0.5169	Yes
93	PITG_01795	PITG_01795			24305	-0.960	-0.5128	Yes
94	PITG_17779	PITG_17779			24311	-0.962	-0.5075	Yes
95	PITG_04487	PITG_04487			24365	-0.980	-0.5038	Yes
96	PITG_05649	PITG_05649			24366	-0.980	-0.4983	Yes
97	PITG_03274	PITG_03274			24545	-1.036	-0.4989	Yes
98	PITG_03221	PITG_03221			24557	-1.040	-0.4934	Yes
99	PITG_15217	PITG_15217			24636	-1.062	-0.4902	Yes
100	PITG_07885	PITG_07885			24738	-1.095	-0.4877	Yes
101	PITG_21447	PITG_21447			24791	-1.115	-0.4833	Yes
102	PITG_06981	PITG_06981			24862	-1.138	-0.4793	Yes
103	PITG_19551	PITG_19551			25020	-1.198	-0.4783	Yes
104	PITG_07888	PITG_07888			25072	-1.216	-0.4732	Yes
105	PITG_17240	PITG_17240			25080	-1.218	-0.4666	Yes
106	PITG_01171	PITG_01171			25118	-1.234	-0.4609	Yes
107	PITG_16548	PITG_16548			25128	-1.238	-0.4542	Yes
108	PITG_18251	PITG_18251			25141	-1.242	-0.4476	Yes
109	PITG_08724	PITG_08724			25231	-1.272	-0.4436	Yes
110	PITG_03999	PITG_03999			25257	-1.283	-0.4373	Yes
111	PITG_20377	PITG_20377			25283	-1.295	-0.4308	Yes
112	PITG_22434	PITG_22434			25346	-1.327	-0.4256	Yes
113	PITG_11653	PITG_11653			25357	-1.334	-0.4183	Yes
114	PITG_07890	PITG_07890			25386	-1.344	-0.4117	Yes
115	PITG_05603	PITG_05603			25403	-1.355	-0.4046	Yes
116	PITG_03201	PITG_03201			25430	-1.367	-0.3978	Yes

117	PITG_08959	PITG_08959			25458	-1.380	-0.3910	Yes
118	PITG_09506	PITG_09506			25563	-1.431	-0.3867	Yes
119	PITG_10146	PITG_10146			25685	-1.491	-0.3826	Yes
120	PITG_15623	PITG_15623			25726	-1.510	-0.3755	Yes
121	PITG_06262	PITG_06262			25777	-1.533	-0.3686	Yes
122	PITG_01248	PITG_01248			25790	-1.543	-0.3603	Yes
123	PITG_15740	PITG_15740			25791	-1.543	-0.3515	Yes
124	PITG_00302	PITG_00302			25872	-1.579	-0.3455	Yes
125	PITG_14747	PITG_14747			26012	-1.664	-0.3411	Yes
126	PITG_19531	PITG_19531			26082	-1.713	-0.3339	Yes
127	PITG_03178	PITG_03178			26113	-1.732	-0.3252	Yes
128	PITG_02450	PITG_02450			26193	-1.786	-0.3179	Yes
129	PITG_00910	PITG_00910			26229	-1.807	-0.3089	Yes
130	PITG_06821	PITG_06821			26267	-1.833	-0.2999	Yes
131	PITG_09728	PITG_09728			26297	-1.848	-0.2905	Yes
132	PITG_10166	PITG_10166			26343	-1.875	-0.2815	Yes
133	PITG_00397	PITG_00397			26402	-1.929	-0.2726	Yes
134	PITG_18688	PITG_18688			26419	-1.945	-0.2622	Yes
135	PITG_01215	PITG_01215			26496	-2.008	-0.2535	Yes
136	PITG_01035	PITG_01035			26606	-2.096	-0.2456	Yes
137	PITG_00558	PITG_00558			26732	-2.206	-0.2377	Yes
138	PITG_01680	PITG_01680			26749	-2.230	-0.2256	Yes
139	PITG_02039	PITG_02039			26754	-2.231	-0.2131	Yes
140	PITG_04428	PITG_04428			26849	-2.337	-0.2032	Yes
141	PITG_03764	PITG_03764			26855	-2.344	-0.1901	Yes
142	PITG_01495	PITG_01495			26914	-2.411	-0.1785	Yes
143	PITG_17607	PITG_17607			26969	-2.477	-0.1665	Yes
144	PITG_20401	PITG_20401			26992	-2.503	-0.1531	Yes
145	PITG_22020	PITG_22020			27018	-2.545	-0.1395	Yes
146	PITG_05826	PITG_05826			27124	-2.709	-0.1280	Yes
147	PITG_21661	PITG_21661			27161	-2.778	-0.1135	Yes
148	PITG_06827	PITG_06827			27165	-2.789	-0.0978	Yes
149	PITG_12483	PITG_12483			27235	-2.906	-0.0838	Yes
150	PITG_16015	PITG_16015			27267	-2.974	-0.0681	Yes
151	PITG_07651	PITG_07651			27309	-3.056	-0.0522	Yes
152	PITG_07308	PITG_07308			27405	-3.341	-0.0367	Yes
153	PITG_05512	PITG_05512			27421	-3.384	-0.0181	Yes
154	PITG_03308	PITG_03308			27545	-4.077	0.0006	Yes

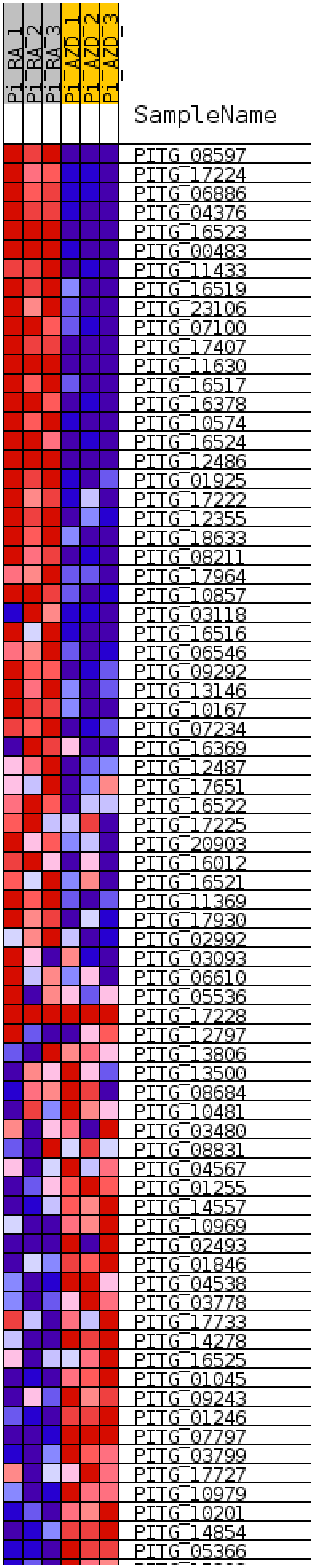
Pi_RA_1
Pi_RA_2
Pi_RA_3
Pi_AZD_1
Pi_AZD_2
Pi_AZD_3
SampleName
PITG_08597
PITG_17224
PITG_06886
PITG_04376
PITG_16523
PITG_00483
PITG_11433
PITG_16519
PITG_23106
PITG_07100
PITG_17407
PITG_11630
PITG_16517
PITG_16378
PITG_10574
PITG_16524
PITG_12486
PITG_01925
PITG_17222
PITG_12355
PITG_18633
PITG_08211
PITG_17964
PITG_10857
PITG_03118
PITG_16516
PITG_06546
PITG_09292
PITG_13146
PITG_10167
PITG_07234
PITG_16369
PITG_12487
PITG_17651
PITG_16522
PITG_17225
PITG_20903
PITG_16012
PITG_16521
PITG_11369
PITG_17930
PITG_02992
PITG_03093
PITG_06610
PITG_05536
PITG_17228
PITG_12797
PITG_13806
PITG_13500
PITG_08684
PITG_10481
PITG_03480
PITG_08831
PITG_04567
PITG_01255
PITG_14557
PITG_10969
PITG_02493
PITG_01846
PITG_04538
PITG_03778
PITG_17733
PITG_14278
PITG_16525
PITG_01045
PITG_09243
PITG_01246
PITG_07797
PITG_03799
PITG_17727
PITG_10979
PITG_10201
PITG_14854
PITG_05366

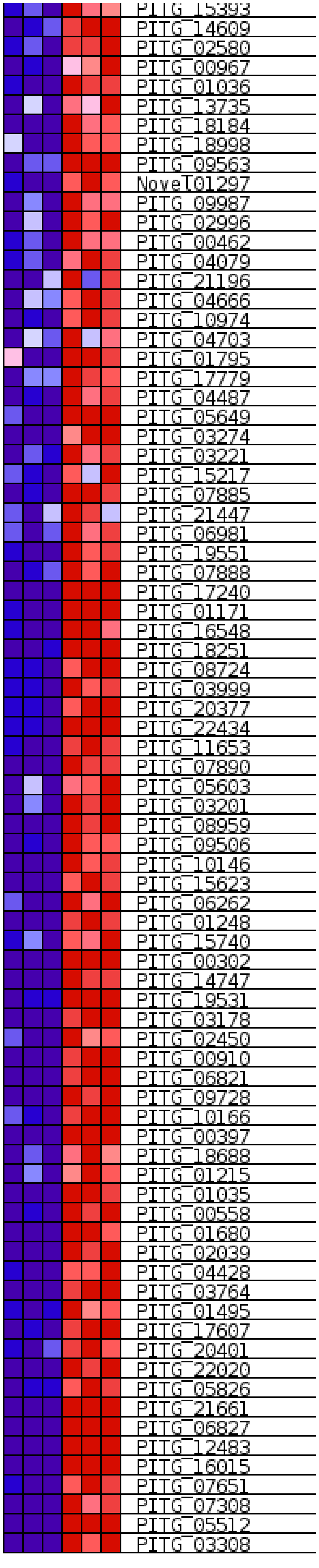
PITG_15393
PITG_14609
PITG_02580
PITG_00967
PITG_01036
PITG_13735
PITG_18184
PITG_18998
PITG_09563
Novel_01297
PITG_09987
PITG_02996
PITG_00462
PITG_04079
PITG_21196
PITG_04666
PITG_10974
PITG_04703
PITG_01795
PITG_17779
PITG_04487
PITG_05649
PITG_03274
PITG_03221
PITG_15217
PITG_07885
PITG_21447
PITG_06981
PITG_19551
PITG_07888
PITG_17240
PITG_01171
PITG_16548
PITG_18251
PITG_08724
PITG_03999
PITG_20377
PITG_22434
PITG_11653
PITG_07890
PITG_05603
PITG_03201
PITG_08959
PITG_09506
PITG_10146
PITG_15623
PITG_06262
PITG_01248
PITG_15740
PITG_00302
PITG_14747
PITG_19531
PITG_03178
PITG_02450
PITG_00910
PITG_06821
PITG_09728
PITG_10166
PITG_00397
PITG_18688
PITG_01215
PITG_01035
PITG_00558
PITG_01680
PITG_02039
PITG_04428
PITG_03764
PITG_01495
PITG_17607
PITG_20401
PITG_22020
PITG_05826
PITG_21661
PITG_06827
PITG_12483
PITG_16015
PITG_07651
PITG_07308
PITG_05512
PITG_03308

Fig 2: RNA_BINDING(GO:0003723)
Blue-Pink O' Gram in the Space of the Analyzed GeneSet

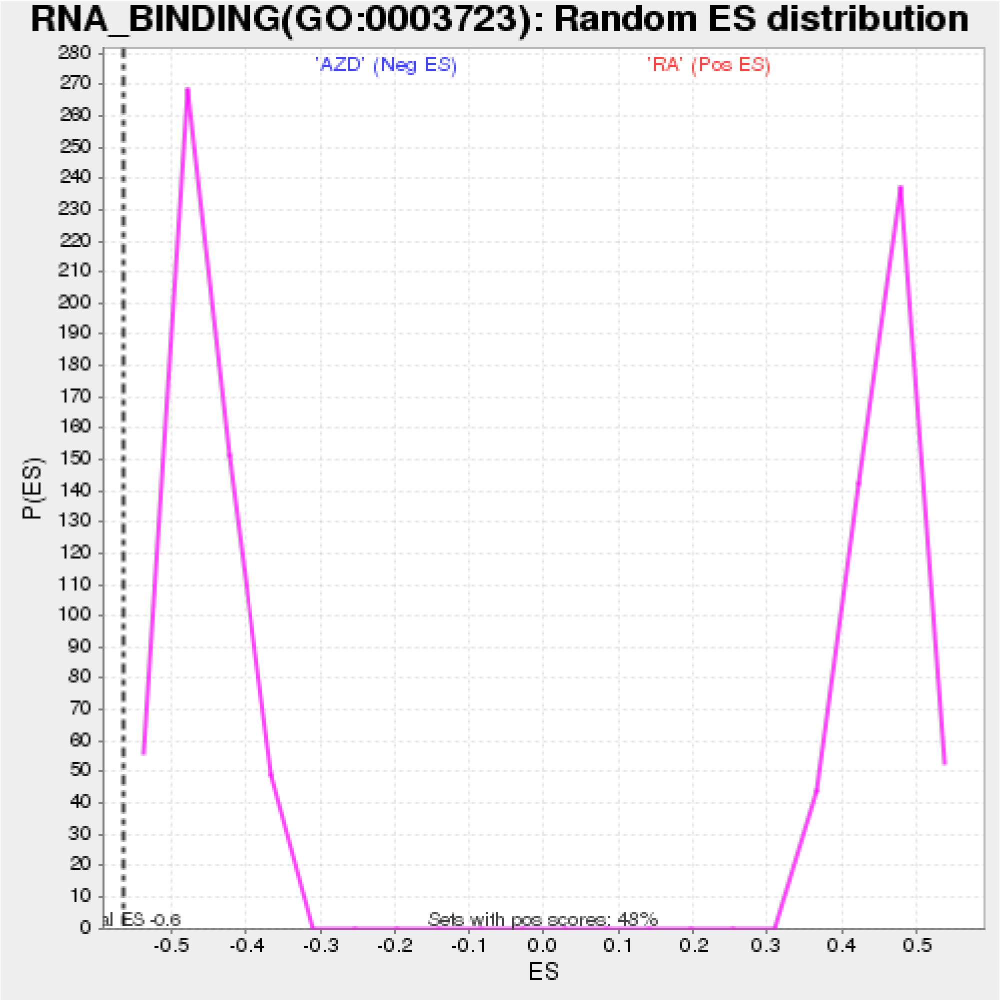

Fig 3: RNA_BINDING(GO:0003723): Random ES distribution
Gene set null distribution of ES for RNA_BINDING(GO:0003723)

9. structural constituent of ribosome

Table: GSEA Results Summary

Dataset	fpkm.sample
Phenotype	sample.cls
Upregulated in class	AZD
GeneSet	STRUCTURAL_CONSTITUENT_OF_RIBOSOME(GO:0003735)
Enrichment Score (ES)	-0.76056206
Normalized Enrichment Score (NES)	-1.0504744
Nominal p-value	0.09351145
FDR q-value	0.13867675
FWER p-Value	0.206

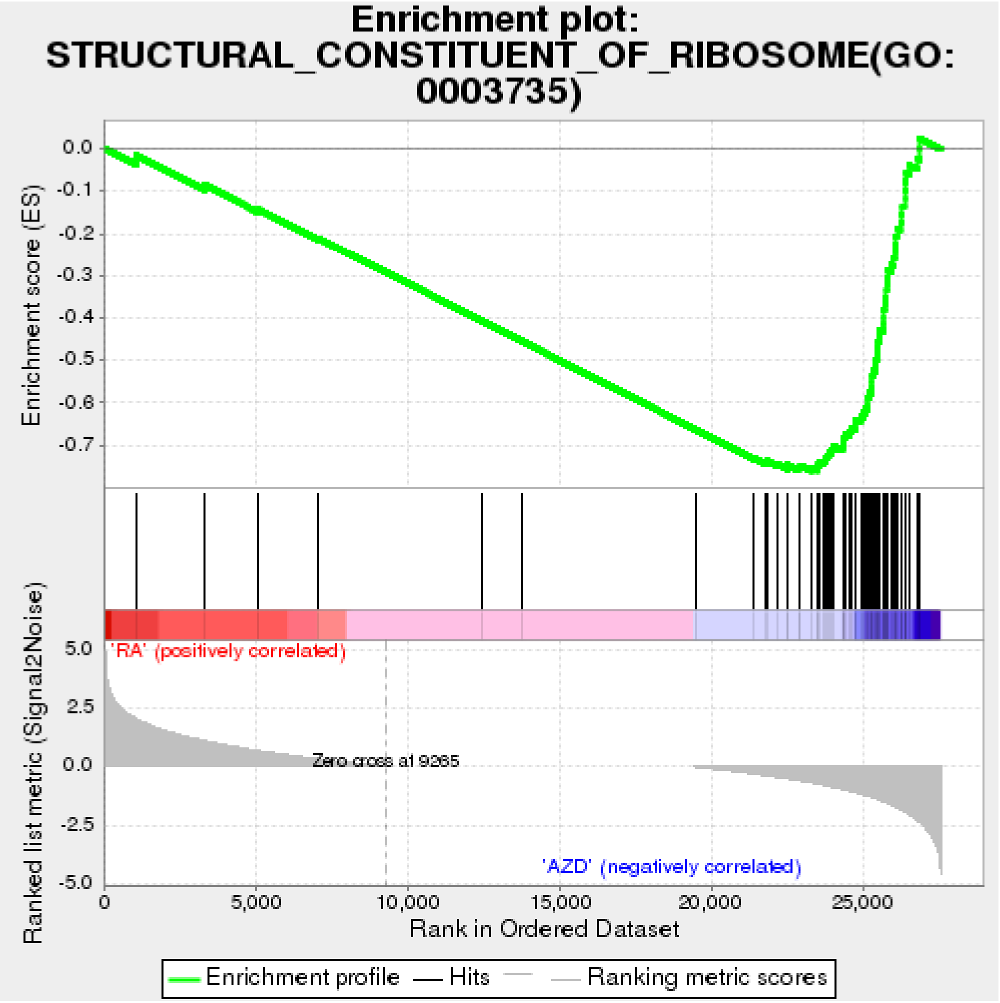

Fig 1: Enrichment plot: STRUCTURAL_CONSTITUENT_OF_RIBOSOME(GO:0003735)
Profile of the Running ES Score & Positions of GeneSet Members on the Rank Ordered List

Table: GSEA details [plain text format]

	PROBE	DESCRIPTION	GENE	GENE_TITLE	RANK IN	RANK	RUNNING	CORE

		(from dataset)	SYMBOL		GENE LIST	METRIC SCORE	ES	ENRICHMENT
1	PITG_19121	PITG_19121			1014	2.048	-0.0147	No
2	PITG_03660	PITG_03660			3265	1.116	-0.0845	No
3	PITG_04337	PITG_04337			5068	0.678	-0.1427	No
4	PITG_05009	PITG_05009			7048	0.319	-0.2112	No
5	PITG_15722	PITG_15722			12398	0.000	-0.4058	No
6	PITG_09431	PITG_09431			13717	0.000	-0.4538	No
7	PITG_10193	PITG_10193			19492	-0.015	-0.6637	No
8	PITG_11734	PITG_11734			21411	-0.312	-0.7301	No
9	PITG_14850	PITG_14850			21815	-0.384	-0.7406	No
10	PITG_16757	PITG_16757			21821	-0.385	-0.7366	No
11	PITG_20188	PITG_20188			22157	-0.445	-0.7440	No
12	PITG_19669	PITG_19669			22503	-0.510	-0.7510	No
13	PITG_12839	PITG_12839			22518	-0.514	-0.7459	No
14	PITG_19999	PITG_19999			22878	-0.594	-0.7526	No
15	PITG_00443	PITG_00443			22936	-0.604	-0.7481	No
16	PITG_12745	PITG_12745			23280	-0.681	-0.7532	Yes
17	PITG_15090	PITG_15090			23482	-0.725	-0.7526	Yes
18	PITG_04843	PITG_04843			23523	-0.735	-0.7461	Yes
19	PITG_16008	PITG_16008			23545	-0.740	-0.7389	Yes
20	PITG_06771	PITG_06771			23729	-0.789	-0.7370	Yes
21	PITG_10887	PITG_10887			23788	-0.807	-0.7303	Yes
22	PITG_20189	PITG_20189			23835	-0.820	-0.7231	Yes
23	PITG_03420	PITG_03420			23870	-0.829	-0.7154	Yes
24	PITG_15723	PITG_15723			23962	-0.858	-0.7094	Yes
25	PITG_01922	PITG_01922			24019	-0.874	-0.7020	Yes
26	PITG_02694	PITG_02694			24328	-0.970	-0.7027	Yes
27	PITG_04487	PITG_04487			24365	-0.980	-0.6933	Yes
28	PITG_01943	PITG_01943			24378	-0.982	-0.6831	Yes
29	PITG_11923	PITG_11923			24405	-0.990	-0.6734	Yes
30	PITG_03221	PITG_03221			24557	-1.040	-0.6676	Yes
31	PITG_11766	PITG_11766			24642	-1.064	-0.6591	Yes
32	PITG_06995	PITG_06995			24760	-1.104	-0.6514	Yes
33	PITG_01833	PITG_01833			24767	-1.106	-0.6396	Yes
34	PITG_05171	PITG_05171			24921	-1.158	-0.6326	Yes
35	PITG_15069	PITG_15069			25019	-1.198	-0.6232	Yes
36	PITG_00941	PITG_00941			25067	-1.214	-0.6117	Yes
37	PITG_12697	PITG_12697			25164	-1.249	-0.6017	Yes
38	PITG_13371	PITG_13371			25168	-1.249	-0.5883	Yes

39	PITG_15407	PITG_15407			25203	-1.263	-0.5758	Yes
40	PITG_05174	PITG_05174			25267	-1.288	-0.5641	Yes
41	PITG_03294	PITG_03294			25303	-1.306	-0.5513	Yes
42	PITG_08703	PITG_08703			25308	-1.308	-0.5372	Yes
43	PITG_07173	PITG_07173			25342	-1.325	-0.5241	Yes
44	PITG_14913	PITG_14913			25405	-1.356	-0.5116	Yes
45	PITG_18052	PITG_18052			25424	-1.365	-0.4975	Yes
46	PITG_08959	PITG_08959			25458	-1.380	-0.4837	Yes
47	PITG_03235	PITG_03235			25480	-1.389	-0.4694	Yes
48	PITG_04382	PITG_04382			25503	-1.398	-0.4551	Yes
49	PITG_14729	PITG_14729			25528	-1.410	-0.4407	Yes
50	PITG_09506	PITG_09506			25563	-1.431	-0.4264	Yes
51	PITG_10146	PITG_10146			25685	-1.491	-0.4147	Yes
52	PITG_09540	PITG_09540			25692	-1.493	-0.3987	Yes
53	PITG_00523	PITG_00523			25696	-1.497	-0.3826	Yes
54	PITG_10863	PITG_10863			25713	-1.502	-0.3669	Yes
55	PITG_06237	PITG_06237			25757	-1.523	-0.3519	Yes
56	PITG_17785	PITG_17785			25774	-1.532	-0.3359	Yes
57	PITG_03353	PITG_03353			25780	-1.535	-0.3195	Yes
58	PITG_07300	PITG_07300			25805	-1.550	-0.3035	Yes
59	PITG_20264	PITG_20264			25831	-1.561	-0.2875	Yes
60	Novel00015	Novel00015			25949	-1.627	-0.2742	Yes
61	PITG_12947	PITG_12947			26003	-1.658	-0.2581	Yes
62	PITG_03239	PITG_03239			26044	-1.683	-0.2413	Yes
63	PITG_02578	PITG_02578			26070	-1.705	-0.2238	Yes
64	PITG_19531	PITG_19531			26082	-1.713	-0.2056	Yes
65	PITG_03178	PITG_03178			26113	-1.732	-0.1879	Yes
66	PITG_13681	PITG_13681			26238	-1.816	-0.1728	Yes
67	PITG_09552	PITG_09552			26270	-1.835	-0.1540	Yes
68	PITG_19157	PITG_19157			26301	-1.850	-0.1350	Yes
69	PITG_06636	PITG_06636			26370	-1.898	-0.1169	Yes
70	PITG_09631	PITG_09631			26373	-1.899	-0.0964	Yes
71	PITG_01042	PITG_01042			26385	-1.915	-0.0761	Yes
72	PITG_09555	PITG_09555			26429	-1.950	-0.0565	Yes
73	PITG_09521	PITG_09521			26522	-2.027	-0.0379	Yes
74	PITG_03768	PITG_03768			26780	-2.261	-0.0227	Yes
75	PITG_06596	PITG_06596			26861	-2.351	-0.0002	Yes
76	PITG_18054	PITG_18054			26868	-2.362	0.0252	Yes

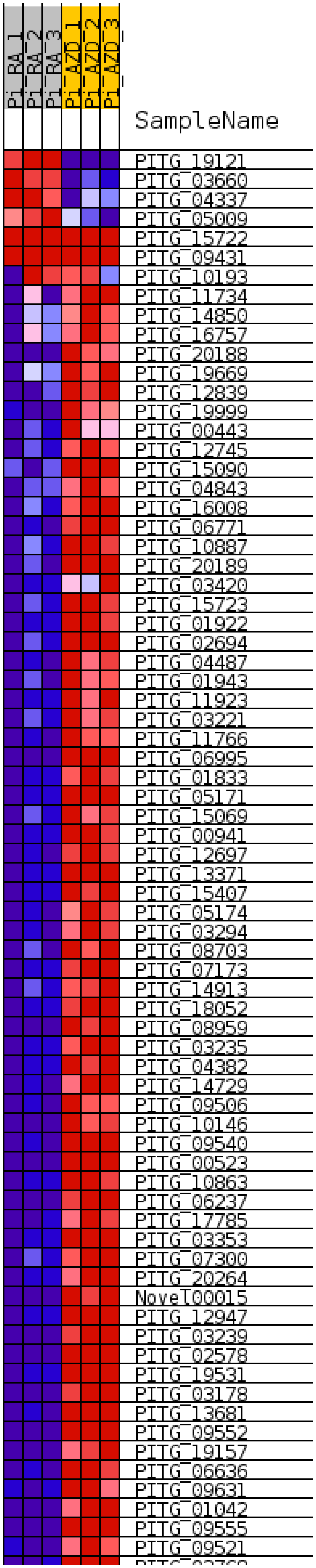
Pi_RA_1
Pi_RA_2
Pi_RA_3
Pi_AZD_1
Pi_AZD_2
Pi_AZD_3
SampleName
PITG_19121
PITG_03660
PITG_04337
PITG_05009
PITG_15722
PITG_09431
PITG_10193
PITG_11734
PITG_14850
PITG_16757
PITG_20188
PITG_19669
PITG_12839
PITG_19999
PITG_00443
PITG_12745
PITG_15090
PITG_04843
PITG_16008
PITG_06771
PITG_10887
PITG_20189
PITG_03420
PITG_15723
PITG_01922
PITG_02694
PITG_04487
PITG_01943
PITG_11923
PITG_03221
PITG_11766
PITG_06995
PITG_01833
PITG_05171
PITG_15069
PITG_00941
PITG_12697
PITG_13371
PITG_15407
PITG_05174
PITG_03294
PITG_08703
PITG_07173
PITG_14913
PITG_18052
PITG_08959
PITG_03235
PITG_04382
PITG_14729
PITG_09506
PITG_10146
PITG_09540
PITG_00523
PITG_10863
PITG_06237
PITG_17785
PITG_03353
PITG_07300
PITG_20264
Novel00015
PITG_12947
PITG_03239
PITG_02578
PITG_19531
PITG_03178
PITG_13681
PITG_09552
PITG_19157
PITG_06636
PITG_09631
PITG_01042
PITG_09555
PITG_09521

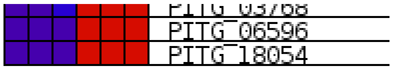

Fig 2: STRUCTURAL_CONSTITUENT_OF_RIBOSOME(GO:0003735)
Blue-Pink O' Gram in the Space of the Analyzed GeneSet

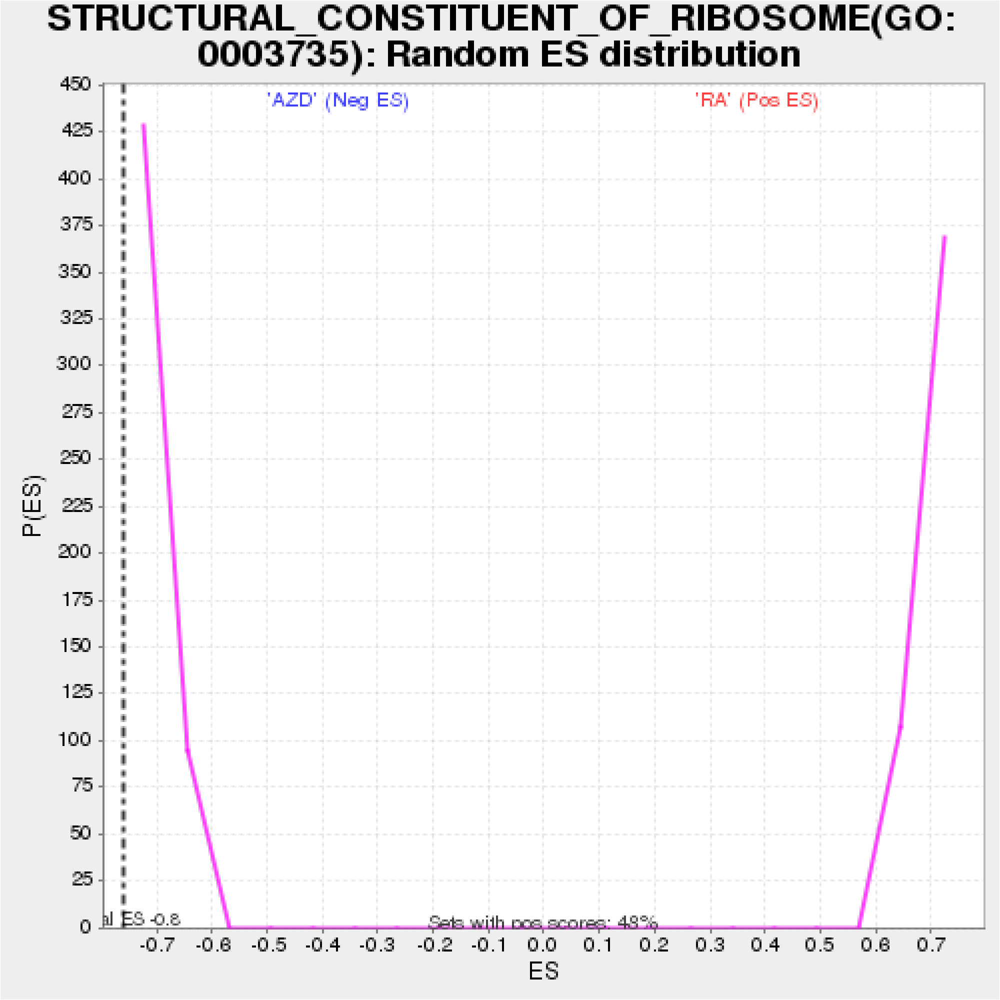

Fig 3: STRUCTURAL_CONSTITUENT_OF_RIBOSOME(GO:0003735): Random ES distribution
Gene set null distribution of ES for STRUCTURAL_CONSTITUENT_OF_RIBOSOME(GO:0003735)

10. Translation

Table: GSEA Results Summary

Dataset	fpkm.sample
Phenotype	sample.cls
Upregulated in class	AZD
GeneSet	TRANSLATION(GO:0006412)
Enrichment Score (ES)	-0.74030447
Normalized Enrichment Score (NES)	-1.0779121
Nominal p-value	0.0
FDR q-value	0.10687026
FWER p-Value	0.056

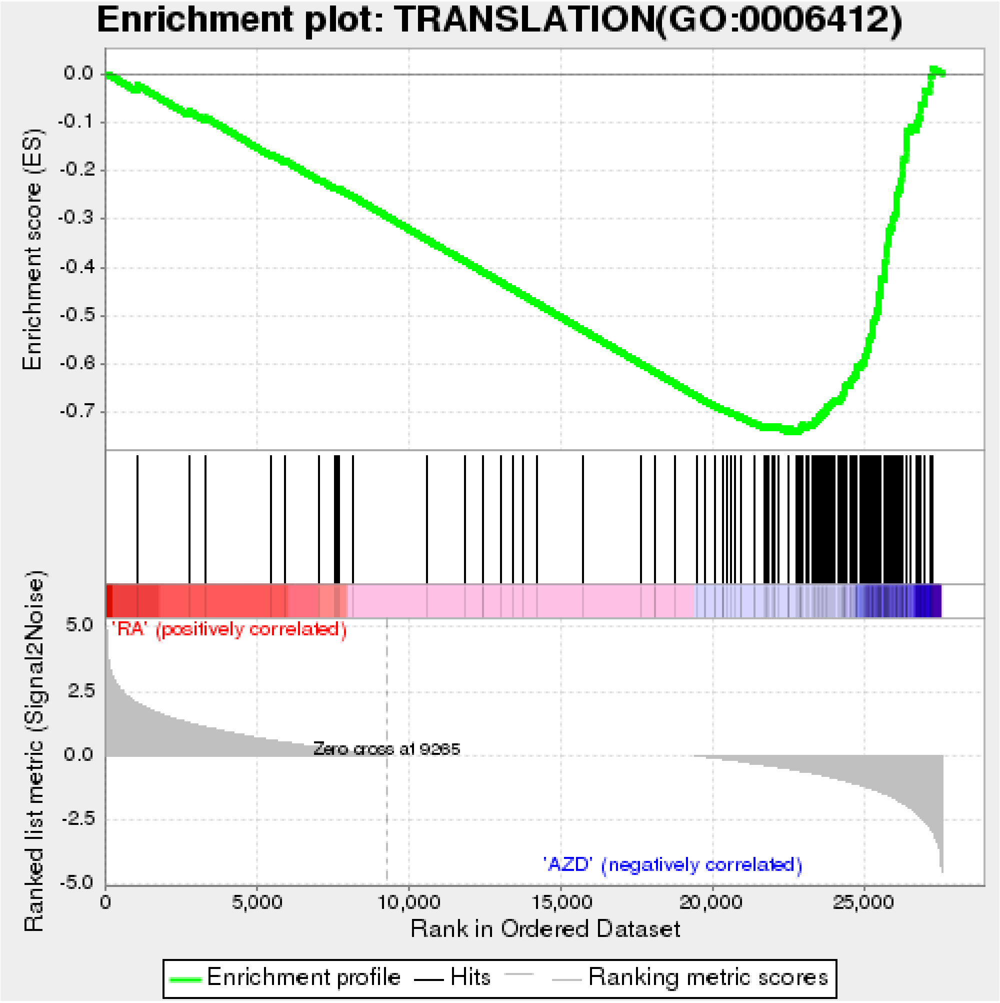

Fig 1: Enrichment plot: TRANSLATION(GO:0006412)
Profile of the Running ES Score & Positions of GeneSet Members on the Rank Ordered List

Table: GSEA details [plain text format]

	PROBE	DESCRIPTION	GENE	GENE_TITLE	RANK IN	RANK	RUNNING	CORE

		(from dataset)	SYMBOL		GENE LIST	METRIC SCORE	ES	ENRICHMENT
1	PITG_19121	PITG_19121			1014	2.048	-0.0239	No
2	PITG_11630	PITG_11630			2711	1.279	-0.0775	No
3	PITG_03660	PITG_03660			3265	1.116	-0.0905	No
4	PITG_07234	PITG_07234			5449	0.602	-0.1663	No
5	PITG_17651	PITG_17651			5906	0.516	-0.1797	No
6	PITG_05009	PITG_05009			7048	0.319	-0.2192	No
7	PITG_02992	PITG_02992			7548	0.243	-0.2359	No
8	PITG_03093	PITG_03093			7622	0.232	-0.2371	No
9	PITG_10516	PITG_10516			7711	0.219	-0.2389	No
10	PITG_17153	PITG_17153			8119	0.169	-0.2526	No
11	PITG_22310	PITG_22310			10562	0.000	-0.3417	No
12	PITG_20824	PITG_20824			11824	0.000	-0.3877	No
13	PITG_15722	PITG_15722			12398	0.000	-0.4086	No
14	PITG_22058	PITG_22058			12412	0.000	-0.4091	No
15	PITG_01091	PITG_01091			13039	0.000	-0.4320	No
16	PITG_05812	PITG_05812			13417	0.000	-0.4457	No
17	PITG_09431	PITG_09431			13717	0.000	-0.4566	No
18	PITG_06873	PITG_06873			14206	0.000	-0.4744	No
19	PITG_16530	PITG_16530			15706	0.000	-0.5291	No
20	PITG_20240	PITG_20240			17604	0.000	-0.5983	No
21	PITG_03806	PITG_03806			18089	0.000	-0.6160	No
22	PITG_03807	PITG_03807			18090	0.000	-0.6160	No
23	PITG_04594	PITG_04594			18776	0.000	-0.6410	No
24	PITG_10193	PITG_10193			19492	-0.015	-0.6670	No
25	PITG_18303	PITG_18303			19751	-0.048	-0.6761	No
26	PITG_09791	PITG_09791			19763	-0.050	-0.6762	No
27	PITG_03480	PITG_03480			20045	-0.095	-0.6858	No
28	PITG_21349	PITG_21349			20105	-0.104	-0.6873	No
29	PITG_09846	PITG_09846			20357	-0.142	-0.6955	No
30	PITG_01255	PITG_01255			20360	-0.142	-0.6947	No
31	PITG_14557	PITG_14557			20366	-0.143	-0.6940	No
32	PITG_04747	PITG_04747			20459	-0.156	-0.6963	No
33	PITG_16741	PITG_16741			20622	-0.179	-0.7011	No
34	PITG_04729	PITG_04729			20710	-0.194	-0.7030	No
35	PITG_02493	PITG_02493			20900	-0.221	-0.7085	No
36	PITG_04774	PITG_04774			20937	-0.229	-0.7083	No
37	PITG_12077	PITG_12077			21406	-0.311	-0.7234	No
38	PITG_11734	PITG_11734			21411	-0.312	-0.7216	No

39	PITG_12151	PITG_12151			21701	-0.361	-0.7298	No
40	PITG_16328	PITG_16328			21756	-0.372	-0.7294	No
41	PITG_14850	PITG_14850			21815	-0.384	-0.7290	No
42	PITG_16757	PITG_16757			21821	-0.385	-0.7267	No
43	PITG_07797	PITG_07797			21968	-0.412	-0.7294	No
44	PITG_03799	PITG_03799			22019	-0.421	-0.7285	No
45	PITG_05405	PITG_05405			22072	-0.429	-0.7277	No
46	PITG_20188	PITG_20188			22157	-0.445	-0.7279	No
47	PITG_05007	PITG_05007			22498	-0.509	-0.7370	Yes
48	PITG_19669	PITG_19669			22503	-0.510	-0.7339	Yes
49	PITG_12839	PITG_12839			22518	-0.514	-0.7311	Yes
50	PITG_06222	PITG_06222			22748	-0.566	-0.7358	Yes
51	PITG_10979	PITG_10979			22822	-0.583	-0.7348	Yes
52	PITG_19999	PITG_19999			22878	-0.594	-0.7330	Yes
53	PITG_04918	PITG_04918			22927	-0.602	-0.7309	Yes
54	PITG_00443	PITG_00443			22936	-0.604	-0.7273	Yes
55	PITG_05354	PITG_05354			22976	-0.613	-0.7248	Yes
56	PITG_02921	PITG_02921			23131	-0.650	-0.7262	Yes
57	PITG_12961	PITG_12961			23203	-0.663	-0.7245	Yes
58	PITG_12745	PITG_12745			23280	-0.681	-0.7229	Yes
59	PITG_14609	PITG_14609			23351	-0.695	-0.7210	Yes
60	PITG_02580	PITG_02580			23358	-0.695	-0.7168	Yes
61	PITG_11111	PITG_11111			23461	-0.720	-0.7159	Yes
62	PITG_15090	PITG_15090			23482	-0.725	-0.7120	Yes
63	PITG_04843	PITG_04843			23523	-0.735	-0.7087	Yes
64	PITG_16008	PITG_16008			23545	-0.740	-0.7047	Yes
65	PITG_17748	PITG_17748			23641	-0.765	-0.7033	Yes
66	PITG_13735	PITG_13735			23644	-0.766	-0.6984	Yes
67	PITG_06771	PITG_06771			23729	-0.789	-0.6964	Yes
68	PITG_08369	PITG_08369			23772	-0.802	-0.6928	Yes
69	PITG_10887	PITG_10887			23788	-0.807	-0.6882	Yes
70	PITG_20189	PITG_20189			23835	-0.820	-0.6846	Yes
71	PITG_03420	PITG_03420			23870	-0.829	-0.6805	Yes
72	PITG_15723	PITG_15723			23962	-0.858	-0.6784	Yes
73	PITG_01922	PITG_01922			24019	-0.874	-0.6748	Yes
74	PITG_10974	PITG_10974			24137	-0.910	-0.6732	Yes
75	PITG_12864	PITG_12864			24223	-0.936	-0.6703	Yes
76	PITG_04703	PITG_04703			24269	-0.949	-0.6659	Yes
77	PITG_21071	PITG_21071			24300	-0.958	-0.6608	Yes

78	PITG_02694	PITG_02694			24328	-0.970	-0.6556	Yes
79	PITG_04487	PITG_04487			24365	-0.980	-0.6506	Yes
80	PITG_01943	PITG_01943			24378	-0.982	-0.6447	Yes
81	PITG_11923	PITG_11923			24405	-0.990	-0.6393	Yes
82	PITG_03274	PITG_03274			24545	-1.036	-0.6378	Yes
83	PITG_03221	PITG_03221			24557	-1.040	-0.6315	Yes
84	PITG_11766	PITG_11766			24642	-1.064	-0.6277	Yes
85	PITG_07841	PITG_07841			24668	-1.073	-0.6217	Yes
86	PITG_03322	PITG_03322			24740	-1.096	-0.6173	Yes
87	PITG_06995	PITG_06995			24760	-1.104	-0.6109	Yes
88	PITG_01833	PITG_01833			24767	-1.106	-0.6040	Yes
89	PITG_01762	PITG_01762			24911	-1.153	-0.6019	Yes
90	PITG_05171	PITG_05171			24921	-1.158	-0.5947	Yes
91	PITG_22249	PITG_22249			25013	-1.195	-0.5904	Yes
92	PITG_15069	PITG_15069			25019	-1.198	-0.5829	Yes
93	PITG_00941	PITG_00941			25067	-1.214	-0.5768	Yes
94	PITG_07888	PITG_07888			25072	-1.216	-0.5692	Yes
95	PITG_18251	PITG_18251			25141	-1.242	-0.5637	Yes
96	PITG_12697	PITG_12697			25164	-1.249	-0.5565	Yes
97	PITG_13371	PITG_13371			25168	-1.249	-0.5486	Yes
98	PITG_15407	PITG_15407			25203	-1.263	-0.5417	Yes
99	PITG_03999	PITG_03999			25257	-1.283	-0.5354	Yes
100	PITG_05174	PITG_05174			25267	-1.288	-0.5274	Yes
101	PITG_03294	PITG_03294			25303	-1.306	-0.5203	Yes
102	PITG_08703	PITG_08703			25308	-1.308	-0.5121	Yes
103	PITG_07173	PITG_07173			25342	-1.325	-0.5048	Yes
104	PITG_14913	PITG_14913			25405	-1.356	-0.4984	Yes
105	PITG_18052	PITG_18052			25424	-1.365	-0.4902	Yes
106	PITG_08959	PITG_08959			25458	-1.380	-0.4826	Yes
107	PITG_03235	PITG_03235			25480	-1.389	-0.4744	Yes
108	PITG_03661	PITG_03661			25487	-1.391	-0.4657	Yes
109	PITG_04382	PITG_04382			25503	-1.398	-0.4573	Yes
110	PITG_14729	PITG_14729			25528	-1.410	-0.4491	Yes
111	PITG_07141	PITG_07141			25545	-1.420	-0.4406	Yes
112	PITG_09506	PITG_09506			25563	-1.431	-0.4320	Yes
113	PITG_08579	PITG_08579			25572	-1.438	-0.4231	Yes
114	PITG_04992	PITG_04992			25670	-1.484	-0.4171	Yes
115	PITG_10146	PITG_10146			25685	-1.491	-0.4081	Yes
116	PITG_09540	PITG_09540			25692	-1.493	-0.3987	Yes

117	PITG_00523	PITG_00523			25696	-1.497	-0.3892	Yes
118	PITG_09234	PITG_09234			25712	-1.502	-0.3801	Yes
119	PITG_10863	PITG_10863			25713	-1.502	-0.3705	Yes
120	PITG_06237	PITG_06237			25757	-1.523	-0.3623	Yes
121	PITG_17785	PITG_17785			25774	-1.532	-0.3530	Yes
122	PITG_03353	PITG_03353			25780	-1.535	-0.3434	Yes
123	PITG_07300	PITG_07300			25805	-1.550	-0.3343	Yes
124	PITG_20264	PITG_20264			25831	-1.561	-0.3252	Yes
125	PITG_00302	PITG_00302			25872	-1.579	-0.3165	Yes
126	Novel00015	Novel00015			25949	-1.627	-0.3088	Yes
127	PITG_14456	PITG_14456			25952	-1.628	-0.2985	Yes
128	PITG_12947	PITG_12947			26003	-1.658	-0.2897	Yes
129	PITG_03239	PITG_03239			26044	-1.683	-0.2803	Yes
130	PITG_02578	PITG_02578			26070	-1.705	-0.2703	Yes
131	PITG_19531	PITG_19531			26082	-1.713	-0.2597	Yes
132	PITG_03460	PITG_03460			26094	-1.719	-0.2491	Yes
133	PITG_03178	PITG_03178			26113	-1.732	-0.2386	Yes
134	PITG_13831	PITG_13831			26183	-1.780	-0.2297	Yes
135	PITG_00910	PITG_00910			26229	-1.807	-0.2197	Yes
136	PITG_13681	PITG_13681			26238	-1.816	-0.2084	Yes
137	PITG_06821	PITG_06821			26267	-1.833	-0.1976	Yes
138	PITG_09552	PITG_09552			26270	-1.835	-0.1859	Yes
139	PITG_19157	PITG_19157			26301	-1.850	-0.1751	Yes
140	PITG_06636	PITG_06636			26370	-1.898	-0.1655	Yes
141	PITG_09631	PITG_09631			26373	-1.899	-0.1533	Yes
142	PITG_01042	PITG_01042			26385	-1.915	-0.1414	Yes
143	PITG_00397	PITG_00397			26402	-1.929	-0.1296	Yes
144	PITG_09555	PITG_09555			26429	-1.950	-0.1181	Yes
145	PITG_09521	PITG_09521			26522	-2.027	-0.1084	Yes
146	PITG_02039	PITG_02039			26754	-2.231	-0.1025	Yes
147	PITG_03768	PITG_03768			26780	-2.261	-0.0890	Yes
148	PITG_06596	PITG_06596			26861	-2.351	-0.0768	Yes
149	PITG_18054	PITG_18054			26868	-2.362	-0.0618	Yes
150	PITG_17607	PITG_17607			26969	-2.477	-0.0496	Yes
151	PITG_22020	PITG_22020			27018	-2.545	-0.0350	Yes
152	PITG_21661	PITG_21661			27161	-2.778	-0.0224	Yes
153	PITG_08714	PITG_08714			27196	-2.846	-0.0053	Yes
154	PITG_10110	PITG_10110			27232	-2.902	0.0120	Yes

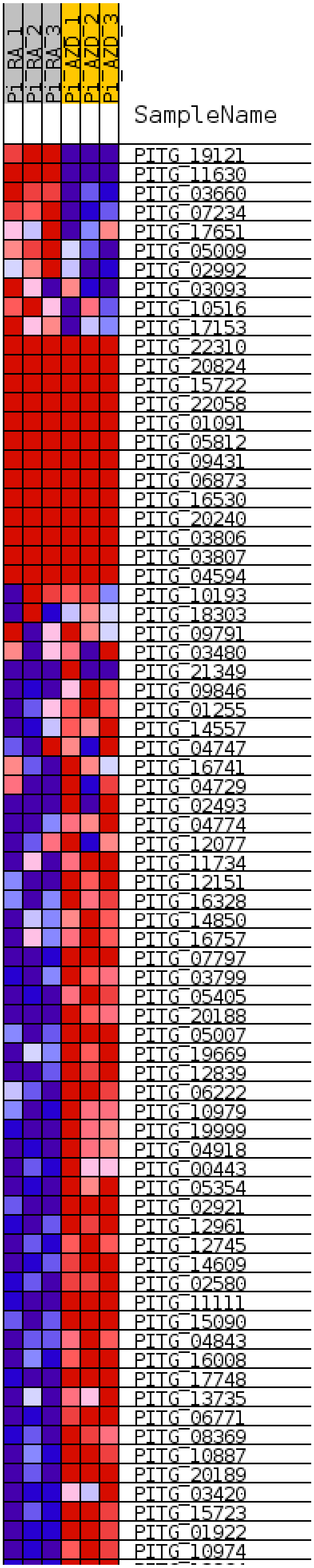
Pi_RA_1
Pi_RA_2
Pi_RA_3
Pi_AZD_1
Pi_AZD_2
Pi_AZD_3
SampleName
PITG_19121
PITG_11630
PITG_03660
PITG_07234
PITG_17651
PITG_05009
PITG_02992
PITG_03093
PITG_10516
PITG_17153
PITG_22310
PITG_20824
PITG_15722
PITG_22058
PITG_01091
PITG_05812
PITG_09431
PITG_06873
PITG_16530
PITG_20240
PITG_03806
PITG_03807
PITG_04594
PITG_10193
PITG_18303
PITG_09791
PITG_03480
PITG_21349
PITG_09846
PITG_01255
PITG_14557
PITG_04747
PITG_16741
PITG_04729
PITG_02493
PITG_04774
PITG_12077
PITG_11734
PITG_12151
PITG_16328
PITG_14850
PITG_16757
PITG_07797
PITG_03799
PITG_05405
PITG_20188
PITG_05007
PITG_19669
PITG_12839
PITG_06222
PITG_10979
PITG_19999
PITG_04918
PITG_00443
PITG_05354
PITG_02921
PITG_12961
PITG_12745
PITG_14609
PITG_02580
PITG_11111
PITG_15090
PITG_04843
PITG_16008
PITG_17748
PITG_13735
PITG_06771
PITG_08369
PITG_10887
PITG_20189
PITG_03420
PITG_15723
PITG_01922
PITG_10974

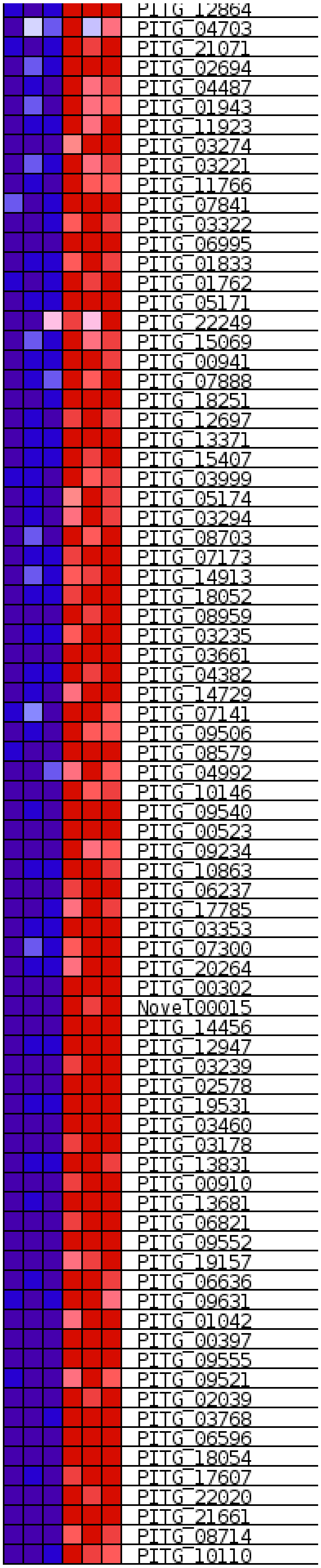
PITG_12864
PITG_04703
PITG_21071
PITG_02694
PITG_04487
PITG_01943
PITG_11923
PITG_03274
PITG_03221
PITG_11766
PITG_07841
PITG_03322
PITG_06995
PITG_01833
PITG_01762
PITG_05171
PITG_22249
PITG_15069
PITG_00941
PITG_07888
PITG_18251
PITG_12697
PITG_13371
PITG_15407
PITG_03999
PITG_05174
PITG_03294
PITG_08703
PITG_07173
PITG_14913
PITG_18052
PITG_08959
PITG_03235
PITG_03661
PITG_04382
PITG_14729
PITG_07141
PITG_09506
PITG_08579
PITG_04992
PITG_10146
PITG_09540
PITG_00523
PITG_09234
PITG_10863
PITG_06237
PITG_17785
PITG_03353
PITG_07300
PITG_20264
PITG_00302
Novel00015
PITG_14456
PITG_12947
PITG_03239
PITG_02578
PITG_19531
PITG_03460
PITG_03178
PITG_13831
PITG_00910
PITG_13681
PITG_06821
PITG_09552
PITG_19157
PITG_06636
PITG_09631
PITG_01042
PITG_00397
PITG_09555
PITG_09521
PITG_02039
PITG_03768
PITG_06596
PITG_18054
PITG_17607
PITG_22020
PITG_21661
PITG_08714
PITG_10110

Fig 2: TRANSLATION(GO:0006412)
Blue-Pink O' Gram in the Space of the Analyzed GeneSet

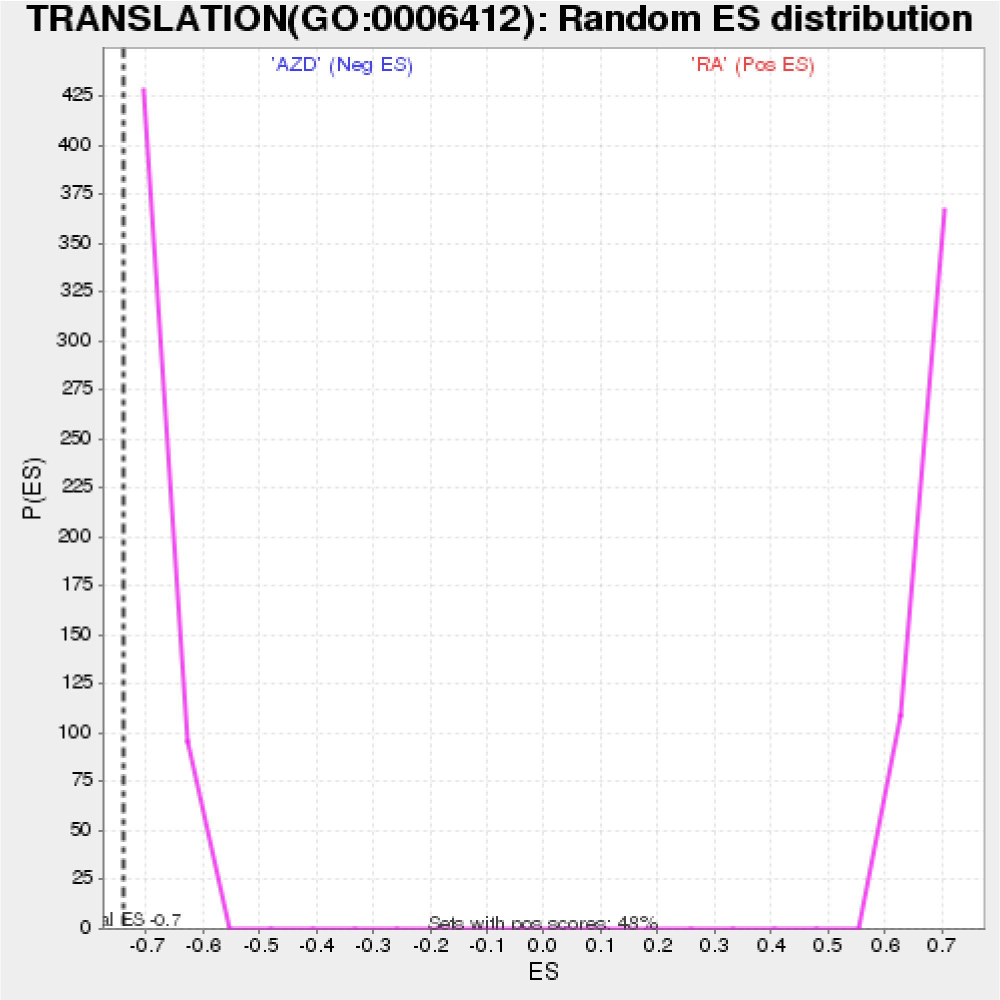

Fig 3: TRANSLATION(GO:0006412): Random ES distribution
Gene set null distribution of ES for TRANSLATION(GO:0006412)
