# Supplementary material for: TOR Inhibitors Synergistically Suppress the Growth and Development of Phytophthora infestans, a Highly Destructive Pathogenic Oomycete
Source: Front Microbiol. 2021 Apr 16;12:596874. doi: 10.3389/fmicb.2021.596874 (PMC8086431; doi:10.3389/fmicb.2021.596874)
Supplement: Supplementary file 6 [file Data_Sheet_3.PDF]

1. Biosynthesis of amino acids

Table: GSEA Results Summary

Dataset	fpkm.sample
Phenotype	sample.cls
Upregulated in class	RAP
GeneSet	BIOSYNTHESIS_OF_AMINO_ACIDS(PIF01230)
Enrichment Score (ES)	-0.41223896
Normalized Enrichment Score (NES)	-1.1468942
Nominal p-value	0.0
FDR q-value	0.13333331
FWER p-Value	0.154

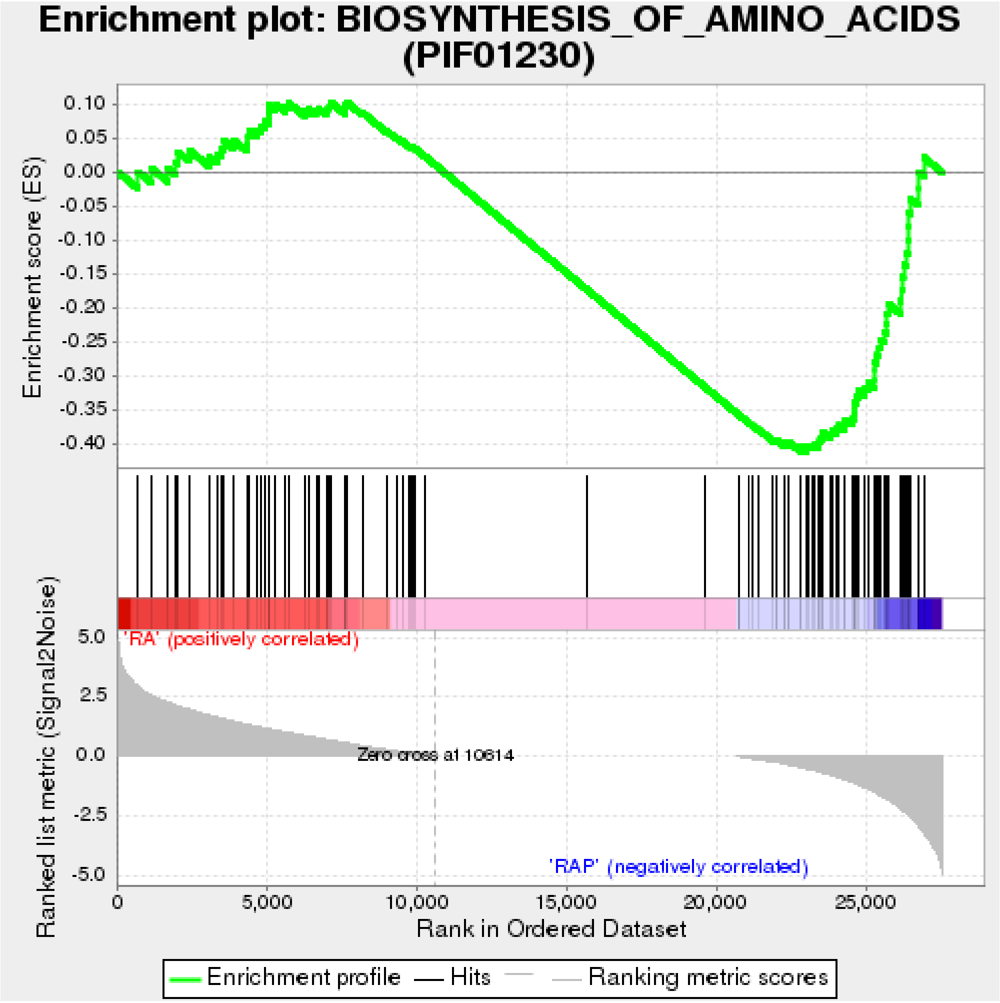

Fig 1: Enrichment plot: BIOSYNTHESIS_OF_AMINO_ACIDS(PIF01230)
Profile of the Running ES Score & Positions of GeneSet Members on the Rank Ordered List

Table: GSEA details [plain text format]

	PROBE	DESCRIPTION	GENE	GENE_TITLE	RANK IN	RANK	RUNNING	CORE

		(from dataset)	SYMBOL		GENE LIST	METRIC SCORE	ES	ENRICHMENT
1	PITG_03593	PITG_03593			657	2.938	0.0010	No
2	PITG_02757	PITG_02757			1125	2.546	0.0056	No
3	PITG_14634	PITG_14634			1656	2.276	0.0056	No
4	PITG_09400	PITG_09400			1906	2.172	0.0150	No
5	PITG_00029	PITG_00029			1980	2.138	0.0305	No
6	PITG_12513	PITG_12513			2366	1.977	0.0332	No
7	PITG_10595	PITG_10595			3059	1.721	0.0226	No
8	PITG_05861	PITG_05861			3303	1.645	0.0277	No
9	PITG_08444	PITG_08444			3442	1.601	0.0363	No
10	PITG_13116	PITG_13116			3529	1.571	0.0465	No
11	PITG_03900	PITG_03900			3830	1.479	0.0481	No
12	PITG_07380	PITG_07380			4294	1.356	0.0428	No
13	PITG_01188	PITG_01188			4341	1.345	0.0525	No
14	PITG_09402	PITG_09402			4368	1.339	0.0629	No
15	PITG_22103	PITG_22103			4659	1.269	0.0631	No
16	PITG_02852	PITG_02852			4805	1.230	0.0683	No
17	PITG_03078	PITG_03078			4894	1.207	0.0753	No
18	PITG_14179	PITG_14179			5021	1.177	0.0807	No
19	PITG_16048	PITG_16048			5028	1.174	0.0905	No
20	PITG_05374	PITG_05374			5055	1.167	0.0994	No
21	PITG_04466	PITG_04466			5276	1.108	0.1008	No
22	PITG_09393	PITG_09393			5588	1.031	0.0982	No
23	PITG_20970	PITG_20970			5705	1.002	0.1025	No
24	PITG_12725	PITG_12725			6228	0.889	0.0910	No
25	PITG_17032	PITG_17032			6359	0.855	0.0935	No
26	PITG_07400	PITG_07400			6621	0.796	0.0908	No
27	PITG_07056	PITG_07056			6723	0.771	0.0937	No
28	PITG_13139	PITG_13139			6956	0.717	0.0913	No
29	PITG_15449	PITG_15449			7014	0.704	0.0952	No
30	PITG_16057	PITG_16057			7082	0.692	0.0986	No
31	PITG_23158	PITG_23158			7135	0.681	0.1025	No
32	PITG_19493	PITG_19493			7580	0.583	0.0913	No
33	PITG_02198	PITG_02198			7607	0.577	0.0952	No
34	PITG_00166	PITG_00166			7613	0.575	0.0999	No
35	PITG_12462	PITG_12462			7678	0.561	0.1024	No
36	PITG_08022	PITG_08022			8158	0.453	0.0888	No
37	PITG_02049	PITG_02049			8985	0.289	0.0611	No
38	PITG_13262	PITG_13262			9320	0.224	0.0509	No

39	PITG_10301	PITG_10301			9501	0.196	0.0460	No
40	PITG_22764	PITG_22764			9755	0.151	0.0380	No
41	PITG_01752	PITG_01752			9804	0.142	0.0375	No
42	PITG_09394	PITG_09394			9840	0.133	0.0373	No
43	PITG_19961	PITG_19961			9941	0.118	0.0347	No
44	PITG_06265	PITG_06265			10247	0.068	0.0242	No
45	PITG_16047	PITG_16047			15683	0.000	-0.1738	No
46	PITG_05858	PITG_05858			19621	0.000	-0.3172	No
47	PITG_03098	PITG_03098			20723	-0.010	-0.3572	No
48	PITG_04851	PITG_04851			20726	-0.010	-0.3572	No
49	PITG_03101	PITG_03101			20746	-0.015	-0.3577	No
50	PITG_02785	PITG_02785			21080	-0.089	-0.3691	No
51	PITG_03700	PITG_03700			21245	-0.122	-0.3741	No
52	PITG_04665	PITG_04665			21414	-0.151	-0.3789	No
53	PITG_01564	PITG_01564			21920	-0.243	-0.3952	No
54	PITG_03598	PITG_03598			21994	-0.254	-0.3957	No
55	PITG_03599	PITG_03599			22009	-0.258	-0.3941	No
56	PITG_22069	PITG_22069			22261	-0.318	-0.4005	No
57	PITG_01195	PITG_01195			22270	-0.321	-0.3981	No
58	PITG_00028	PITG_00028			22431	-0.365	-0.4008	No
59	PITG_05245	PITG_05245			22442	-0.368	-0.3980	No
60	PITG_03698	PITG_03698			22833	-0.469	-0.4083	Yes
61	PITG_18048	PITG_18048			22848	-0.473	-0.4048	Yes
62	PITG_02256	PITG_02256			23052	-0.536	-0.4076	Yes
63	PITG_14180	PITG_14180			23060	-0.537	-0.4033	Yes
64	PITG_05318	PITG_05318			23201	-0.581	-0.4035	Yes
65	PITG_00132	PITG_00132			23312	-0.612	-0.4023	Yes
66	PITG_02925	PITG_02925			23409	-0.645	-0.4003	Yes
67	PITG_17786	PITG_17786			23457	-0.657	-0.3964	Yes
68	PITG_01768	PITG_01768			23503	-0.671	-0.3924	Yes
69	PITG_01939	PITG_01939			23547	-0.690	-0.3881	Yes
70	PITG_02735	PITG_02735			23566	-0.701	-0.3828	Yes
71	PITG_14697	PITG_14697			23859	-0.806	-0.3866	Yes
72	PITG_17516	PITG_17516			23892	-0.818	-0.3808	Yes
73	PITG_17925	PITG_17925			24028	-0.867	-0.3784	Yes
74	PITG_21397	PITG_21397			24090	-0.897	-0.3730	Yes
75	PITG_01804	PITG_01804			24285	-0.982	-0.3717	Yes
76	PITG_02740	PITG_02740			24320	-0.996	-0.3645	Yes
77	PITG_09698	PITG_09698			24544	-1.090	-0.3634	Yes

78	PITG_07283	PITG_07283			24600	-1.115	-0.3559	Yes
79	PITG_01711	PITG_01711			24640	-1.133	-0.3477	Yes
80	PITG_04698	PITG_04698			24645	-1.135	-0.3382	Yes
81	PITG_05636	PITG_05636			24720	-1.175	-0.3309	Yes
82	PITG_03620	PITG_03620			24767	-1.200	-0.3224	Yes
83	PITG_06448	PITG_06448			24953	-1.309	-0.3181	Yes
84	PITG_22685	PITG_22685			25077	-1.387	-0.3108	Yes
85	PITG_09582	PITG_09582			25294	-1.519	-0.3057	Yes
86	PITG_13402	PITG_13402			25297	-1.521	-0.2929	Yes
87	PITG_02210	PITG_02210			25299	-1.522	-0.2800	Yes
88	PITG_12053	PITG_12053			25384	-1.569	-0.2698	Yes
89	PITG_06267	PITG_06267			25457	-1.624	-0.2586	Yes
90	PITG_13749	PITG_13749			25518	-1.666	-0.2467	Yes
91	PITG_06427	PITG_06427			25643	-1.748	-0.2364	Yes
92	PITG_20687	PITG_20687			25706	-1.801	-0.2233	Yes
93	PITG_20759	PITG_20759			25719	-1.808	-0.2084	Yes
94	PITG_01245	PITG_01245			25744	-1.834	-0.1937	Yes
95	PITG_06518	PITG_06518			26151	-2.183	-0.1900	Yes
96	PITG_01769	PITG_01769			26199	-2.231	-0.1728	Yes
97	PITG_07405	PITG_07405			26228	-2.258	-0.1546	Yes
98	PITG_13399	PITG_13399			26268	-2.305	-0.1365	Yes
99	PITG_12727	PITG_12727			26360	-2.403	-0.1194	Yes
100	PITG_09817	PITG_09817			26422	-2.482	-0.1006	Yes
101	PITG_03992	PITG_03992			26434	-2.495	-0.0798	Yes
102	PITG_14195	PITG_14195			26456	-2.517	-0.0592	Yes
103	PITG_19096	PITG_19096			26515	-2.575	-0.0395	Yes
104	PITG_14696	PITG_14696			26770	-2.944	-0.0237	Yes
105	PITG_05551	PITG_05551			26774	-2.956	0.0012	Yes
106	PITG_12161	PITG_12161			26947	-3.227	0.0224	Yes

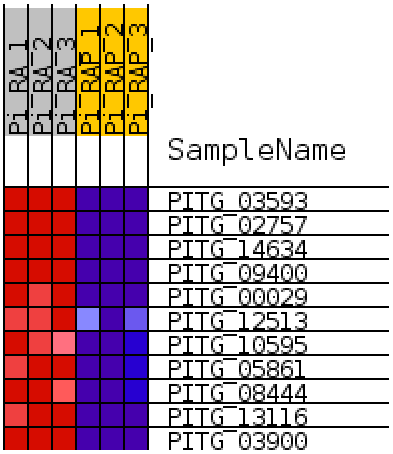

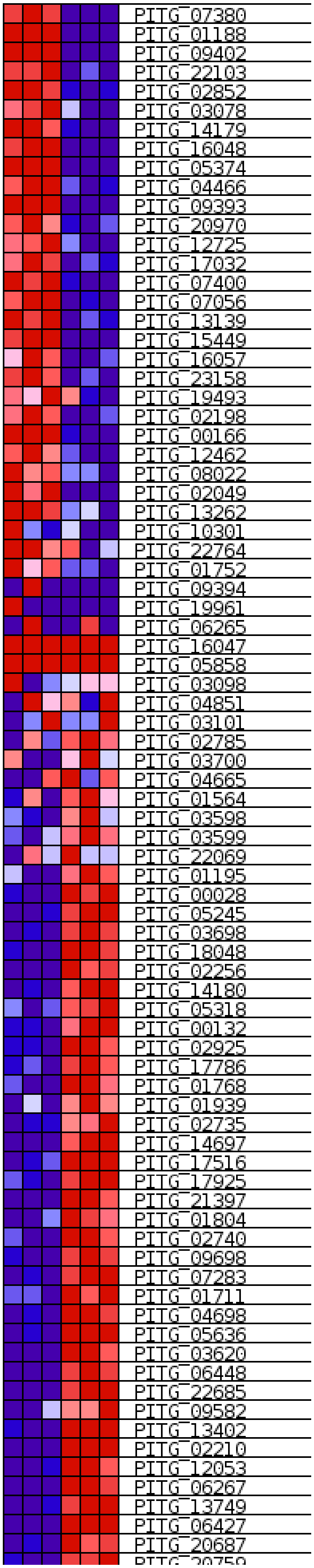
PITG_07380
PITG_01188
PITG_09402
PITG_22103
PITG_02852
PITG_03078
PITG_14179
PITG_16048
PITG_05374
PITG_04466
PITG_09393
PITG_20970
PITG_12725
PITG_17032
PITG_07400
PITG_07056
PITG_13139
PITG_15449
PITG_16057
PITG_23158
PITG_19493
PITG_02198
PITG_00166
PITG_12462
PITG_08022
PITG_02049
PITG_13262
PITG_10301
PITG_22764
PITG_01752
PITG_09394
PITG_19961
PITG_06265
PITG_16047
PITG_05858
PITG_03098
PITG_04851
PITG_03101
PITG_02785
PITG_03700
PITG_04665
PITG_01564
PITG_03598
PITG_03599
PITG_22069
PITG_01195
PITG_00028
PITG_05245
PITG_03698
PITG_18048
PITG_02256
PITG_14180
PITG_05318
PITG_00132
PITG_02925
PITG_17786
PITG_01768
PITG_01939
PITG_02735
PITG_14697
PITG_17516
PITG_17925
PITG_21397
PITG_01804
PITG_02740
PITG_09698
PITG_07283
PITG_01711
PITG_04698
PITG_05636
PITG_03620
PITG_06448
PITG_22685
PITG_09582
PITG_13402
PITG_02210
PITG_12053
PITG_06267
PITG_13749
PITG_06427
PITG_20687

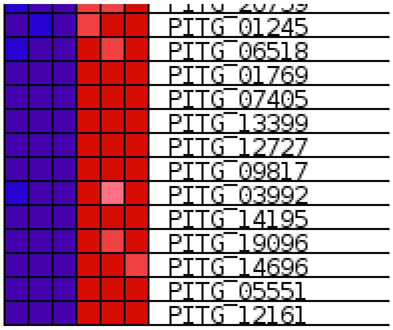

Fig 2: BIOSYNTHESIS_OF_AMINO_ACIDS(PIF01230)
Blue-Pink O' Gram in the Space of the Analyzed GeneSet

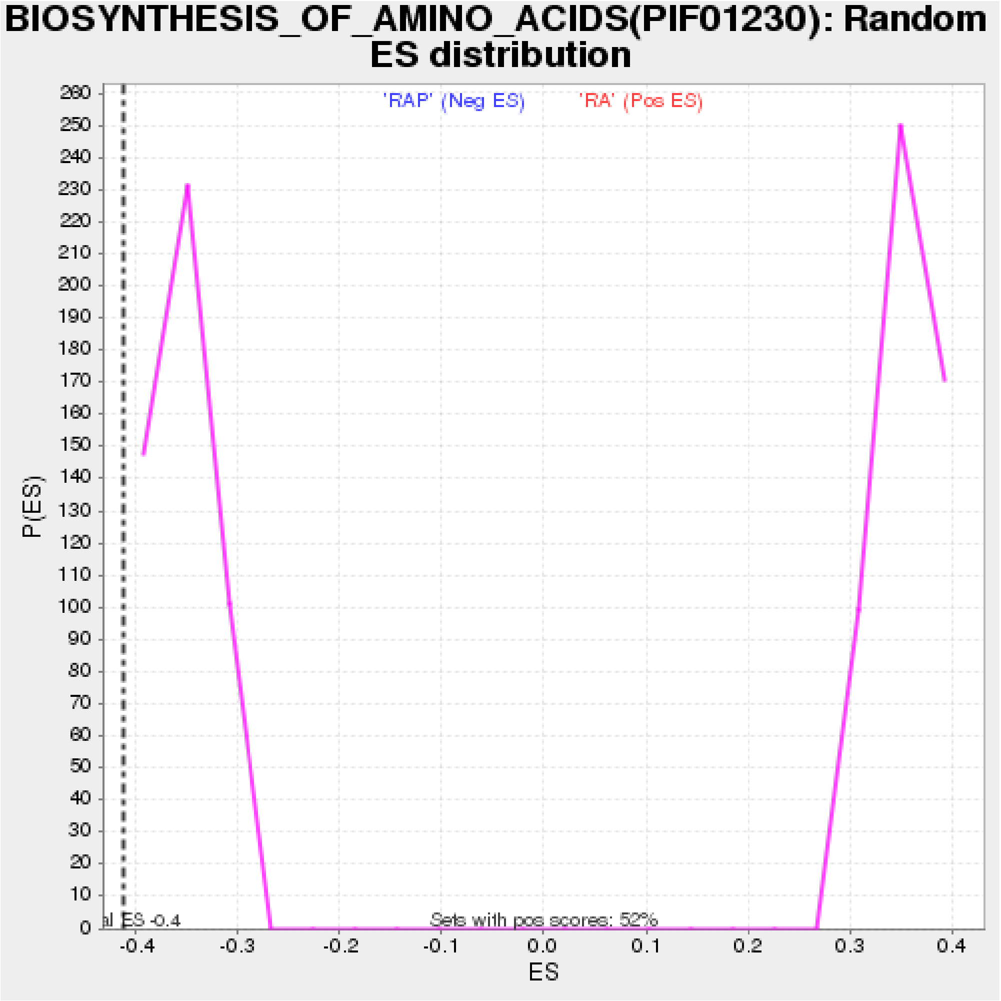

Fig 3: BIOSYNTHESIS_OF_AMINO_ACIDS(PIF01230): Random ES distribution
Gene set null distribution of ES for BIOSYNTHESIS_OF_AMINO_ACIDS(PIF01230)

2. Citrate cycle (TCA cycle)

Table: GSEA Results Summary

Dataset	fpkm.sample
Phenotype	sample.cls
Upregulated in class	RAP
GeneSet	CITRATE_CYCLE_(TCA_CYCLE)(PIF00020)
Enrichment Score (ES)	-0.62979466
Normalized Enrichment Score (NES)	-1.1235752
Nominal p-value	0.0
FDR q-value	0.14192699
FWER p-Value	0.202

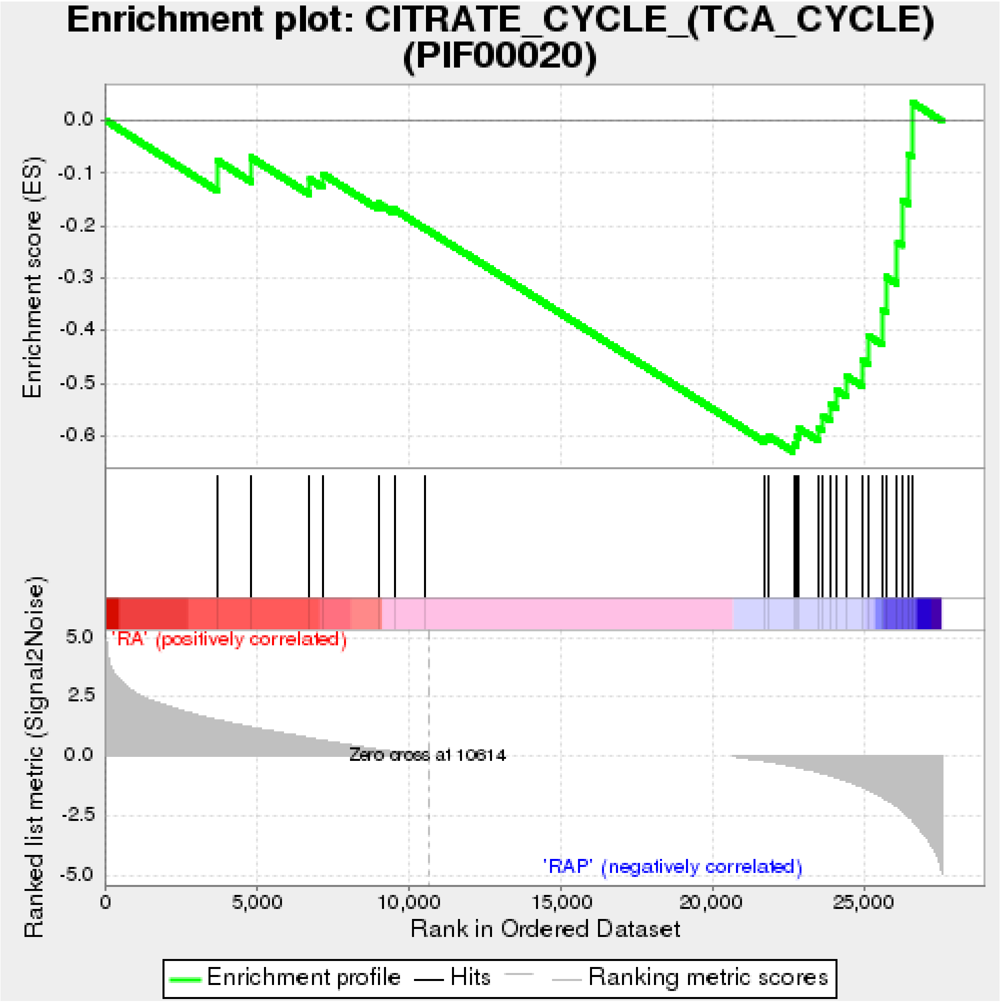

Fig 1: Enrichment plot: CITRATE_CYCLE_(TCA_CYCLE)(PIF00020)
Profile of the Running ES Score & Positions of GeneSet Members on the Rank Ordered List

Table: GSEA details [plain text format]

	PROBE	DESCRIPTION	GENE	GENE_TITLE	RANK IN	RANK	RUNNING	CORE

		(from dataset)	SYMBOL		GENE LIST	METRIC SCORE	ES	ENRICHMENT
1	PITG_15476	PITG_15476			3646	1.531	-0.0749	No
2	PITG_19191	PITG_19191			4773	1.239	-0.0693	No
3	PITG_07056	PITG_07056			6723	0.771	-0.1111	No
4	PITG_23158	PITG_23158			7135	0.681	-0.1005	No
5	PITG_02049	PITG_02049			8985	0.289	-0.1568	No
6	PITG_08880	PITG_08880			9496	0.196	-0.1680	No
7	PITG_12905	PITG_12905			10544	0.016	-0.2054	No
8	PITG_03277	PITG_03277			21718	-0.202	-0.6036	No
9	PITG_18720	PITG_18720			21847	-0.227	-0.5997	No
10	PITG_10951	PITG_10951			22677	-0.425	-0.6138	Yes
11	PITG_16966	PITG_16966			22783	-0.456	-0.6005	Yes
12	PITG_18048	PITG_18048			22848	-0.473	-0.5851	Yes
13	PITG_22743	PITG_22743			23489	-0.665	-0.5834	Yes
14	PITG_15705	PITG_15705			23661	-0.738	-0.5619	Yes
15	PITG_11452	PITG_11452			23908	-0.824	-0.5399	Yes
16	PITG_19161	PITG_19161			24088	-0.896	-0.5128	Yes
17	PITG_06108	PITG_06108			24411	-1.035	-0.4856	Yes
18	PITG_06448	PITG_06448			24953	-1.309	-0.4561	Yes
19	PITG_11929	PITG_11929			25134	-1.425	-0.4092	Yes
20	PITG_13614	PITG_13614			25628	-1.736	-0.3619	Yes
21	PITG_06604	PITG_06604			25726	-1.810	-0.2975	Yes
22	PITG_18354	PITG_18354			26104	-2.132	-0.2311	Yes
23	PITG_15359	PITG_15359			26267	-2.302	-0.1506	Yes
24	PITG_18935	PITG_18935			26483	-2.545	-0.0629	Yes
25	PITG_02026	PITG_02026			26621	-2.717	0.0341	Yes

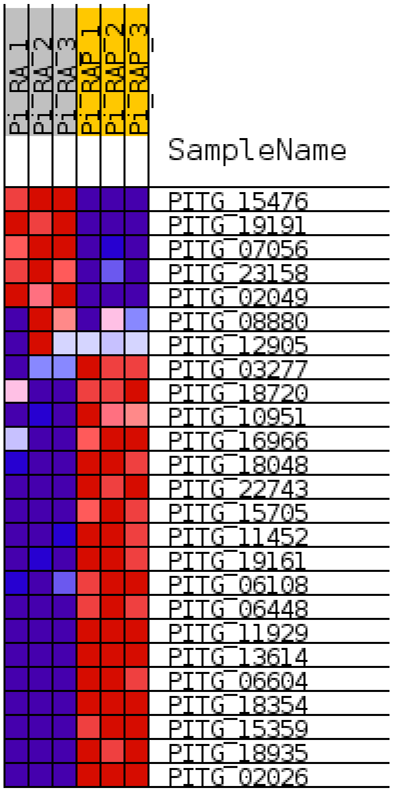

Fig 2: CITRATE_CYCLE_(TCA_CYCLE)(PIF00020)
Blue-Pink O' Gram in the Space of the Analyzed GeneSet

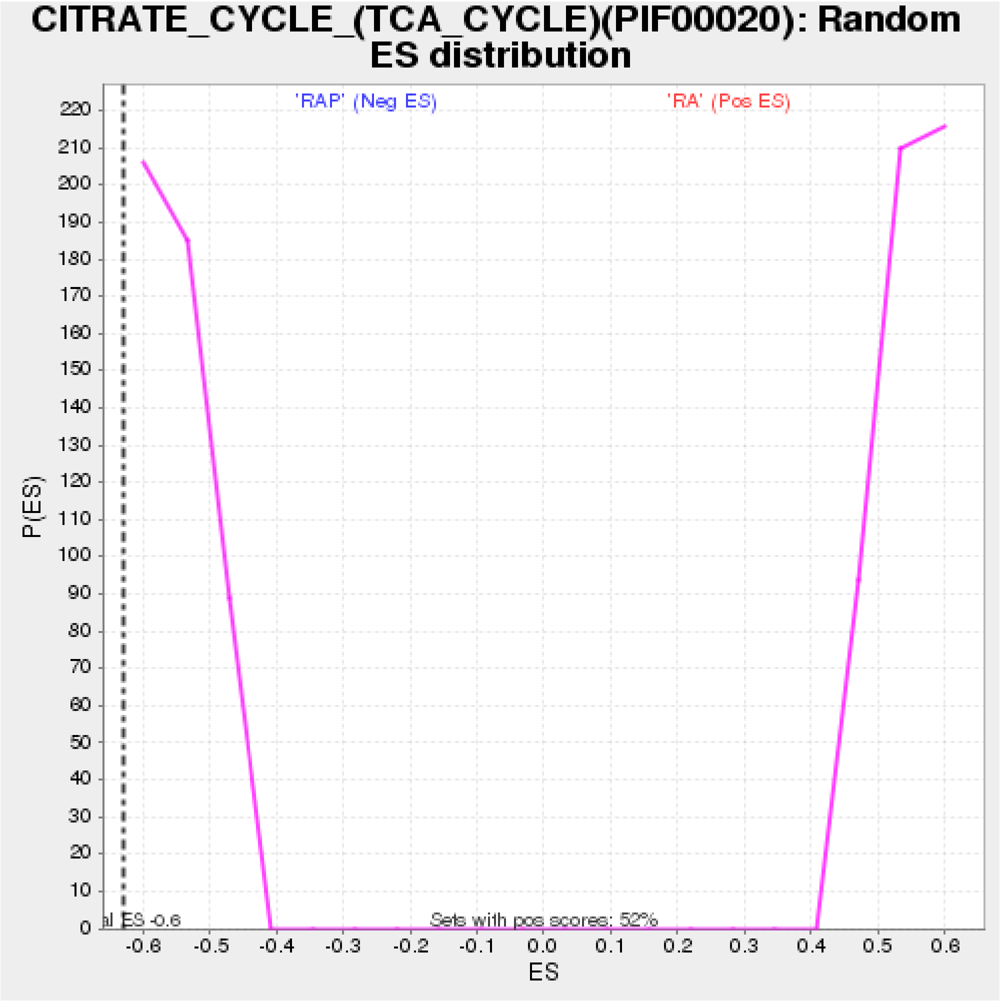

Fig 3: CITRATE_CYCLE_(TCA_CYCLE)(PIF00020): Random ES distribution
Gene set null distribution of ES for CITRATE_CYCLE_(TCA_CYCLE)(PIF00020)

3. DNA replication

Table: GSEA Results Summary

Dataset	fpkm.sample
Phenotype	sample.cls
Upregulated in class	RAP
GeneSet	DNA_REPLICATION(PIF03030)
Enrichment Score (ES)	-0.6251232
Normalized Enrichment Score (NES)	-1.2559272
Nominal p-value	0.0
FDR q-value	0.10208329
FWER p-Value	0.049

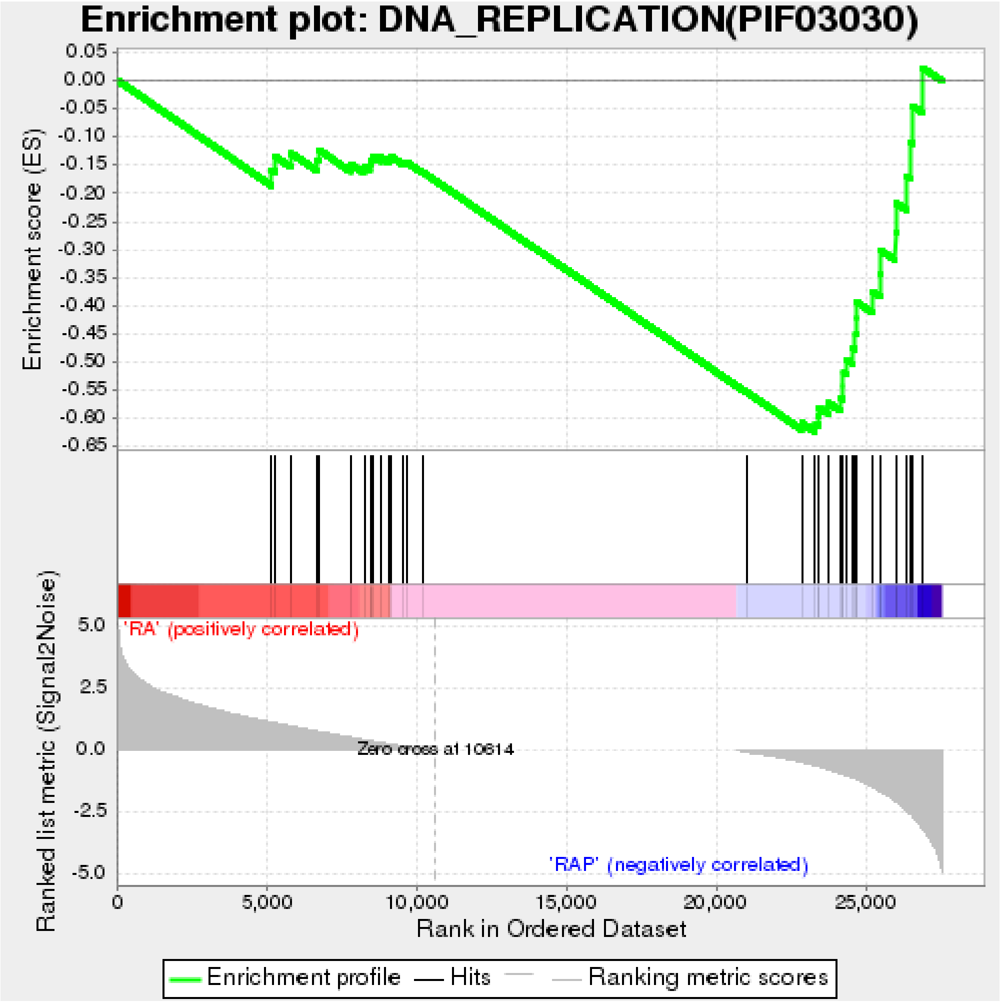

Fig 1: Enrichment plot: DNA_REPLICATION(PIF03030)
Profile of the Running ES Score & Positions of GeneSet Members on the Rank Ordered List

Table: GSEA details ***[plain text format]***

	PROBE	DESCRIPTION	GENE	GENE_TITLE	RANK IN	RANK	RUNNING	CORE

		(from dataset)	SYMBOL		GENE LIST	METRIC SCORE	ES	ENRICHMENT
1	PITG_00557	PITG_00557			5131	1.143	-0.1575	No
2	PITG_10249	PITG_10249			5263	1.110	-0.1342	No
3	PITG_12746	PITG_12746			5798	0.983	-0.1288	No
4	PITG_08606	PITG_08606			6670	0.785	-0.1406	No
5	PITG_10245	PITG_10245			6701	0.777	-0.1220	No
6	PITG_07644	PITG_07644			7775	0.541	-0.1473	No
7	PITG_18696	PITG_18696			8223	0.443	-0.1524	No
8	PITG_13336	PITG_13336			8423	0.396	-0.1496	No
9	PITG_05302	PITG_05302			8434	0.394	-0.1400	No
10	PITG_04709	PITG_04709			8519	0.373	-0.1336	No
11	PITG_10254	PITG_10254			8786	0.322	-0.1352	No
12	PITG_19314	PITG_19314			9075	0.270	-0.1388	No
13	PITG_05369	PITG_05369			9149	0.256	-0.1350	No
14	PITG_04614	PITG_04614			9545	0.188	-0.1446	No
15	PITG_20527	PITG_20527			9678	0.164	-0.1452	No
16	PITG_15505	PITG_15505			10207	0.073	-0.1626	No
17	PITG_18237	PITG_18237			21001	-0.073	-0.5529	No
18	PITG_03389	PITG_03389			22862	-0.477	-0.6084	No
19	PITG_15868	PITG_15868			23323	-0.614	-0.6096	Yes
20	PITG_11812	PITG_11812			23414	-0.646	-0.5965	Yes
21	PITG_12152	PITG_12152			23450	-0.656	-0.5812	Yes
22	PITG_07175	PITG_07175			23767	-0.771	-0.5732	Yes
23	PITG_13792	PITG_13792			24163	-0.928	-0.5641	Yes
24	PITG_05585	PITG_05585			24259	-0.973	-0.5430	Yes
25	PITG_03056	PITG_03056			24260	-0.973	-0.5184	Yes
26	PITG_04710	PITG_04710			24371	-1.017	-0.4966	Yes
27	PITG_08326	PITG_08326			24551	-1.093	-0.4755	Yes
28	PITG_16691	PITG_16691			24661	-1.146	-0.4505	Yes
29	PITG_16698	PITG_16698			24679	-1.157	-0.4219	Yes
30	PITG_07546	PITG_07546			24682	-1.158	-0.3927	Yes
31	PITG_04527	PITG_04527			25212	-1.468	-0.3748	Yes
32	PITG_11912	PITG_11912			25492	-1.647	-0.3433	Yes
33	PITG_12346	PITG_12346			25500	-1.652	-0.3018	Yes
34	PITG_02054	PITG_02054			25999	-2.035	-0.2684	Yes
35	PITG_15053	PITG_15053			26027	-2.059	-0.2173	Yes
36	PITG_06111	PITG_06111			26389	-2.434	-0.1689	Yes
37	PITG_03624	PITG_03624			26480	-2.543	-0.1079	Yes
38	PITG_07399	PITG_07399			26578	-2.652	-0.0444	Yes

39	PITG_14397	PITG_14397			26913	-3.167	0.0235	Yes

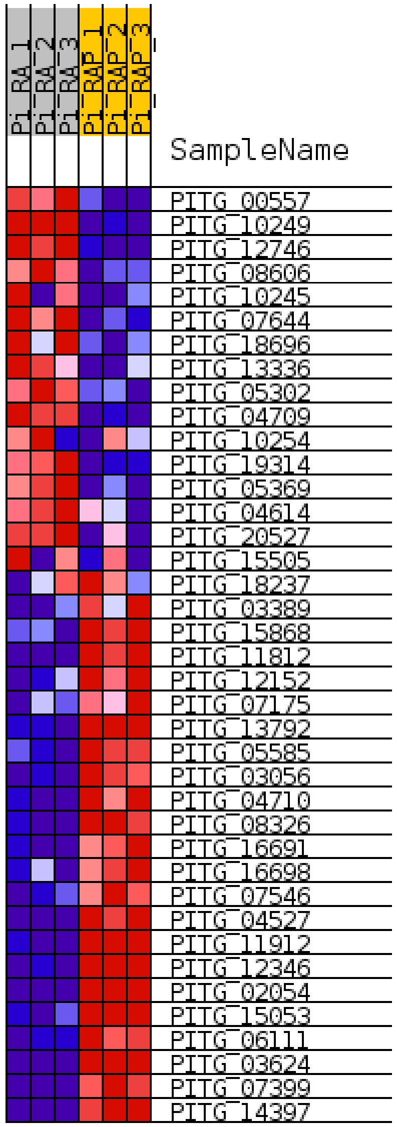

Fig 2: DNA_REPLICATION(PIF03030)
Blue-Pink O' Gram in the Space of the Analyzed GeneSet

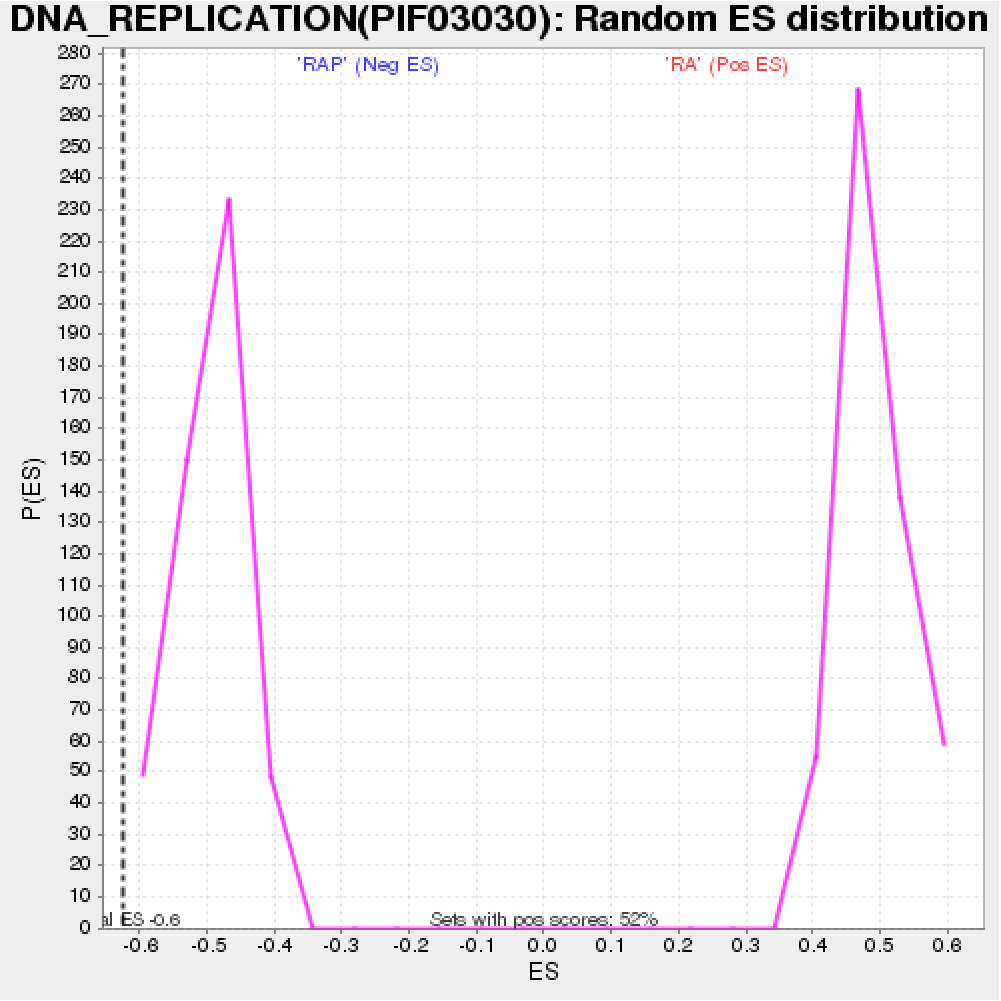

Fig 3: DNA_REPLICATION(PIF03030): Random ES distribution
Gene set null distribution of ES for DNA_REPLICATION(PIF03030)

4. Oxidative phosphorylation

Table: GSEA Results Summary

Dataset	fpkm.sample
Phenotype	sample.cls
Upregulated in class	RAP
GeneSet	OXIDATIVE_PHOSPHORYLATION(PIF00190)
Enrichment Score (ES)	-0.5647333
Normalized Enrichment Score (NES)	-1.1714244
Nominal p-value	0.0
FDR q-value	0.102083296
FWER p-Value	0.049

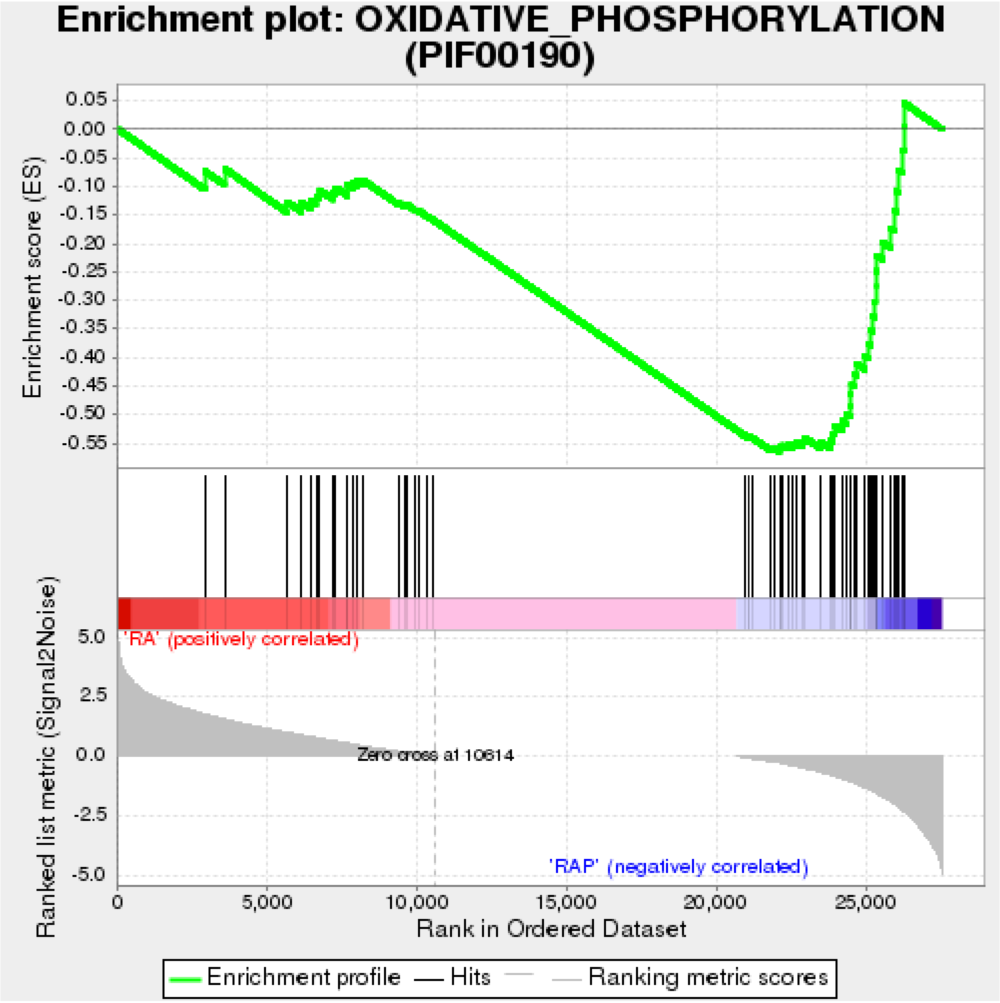

Fig 1: Enrichment plot: OXIDATIVE_PHOSPHORYLATION(PIF00190)
Profile of the Running ES Score & Positions of GeneSet Members on the Rank Ordered List

Table: GSEA details [plain text format]

	PROBE	DESCRIPTION	GENE	GENE_TITLE	RANK IN	RANK	RUNNING	CORE

		(from dataset)	SYMBOL		GENE LIST	METRIC SCORE	ES	ENRICHMENT
1	PITG_09016	PITG_09016			2886	1.775	-0.0729	No
2	PITG_16016	PITG_16016			3545	1.565	-0.0685	No
3	Novel00931	Novel00931			5642	1.015	-0.1264	No
4	PITG_17747	PITG_17747			6129	0.911	-0.1276	No
5	PITG_13301	PITG_13301			6465	0.830	-0.1248	No
6	PITG_06615	PITG_06615			6619	0.796	-0.1160	No
7	PITG_08094	PITG_08094			6745	0.765	-0.1067	No
8	PITG_00764	PITG_00764			7185	0.672	-0.1106	No
9	PITG_06814	PITG_06814			7271	0.652	-0.1019	No
10	PITG_09550	PITG_09550			7673	0.562	-0.1063	No
11	PITG_06385	PITG_06385			7676	0.561	-0.0963	No
12	PITG_09032	PITG_09032			7870	0.518	-0.0939	No
13	PITG_12289	PITG_12289			7999	0.488	-0.0897	No
14	PITG_16670	PITG_16670			8207	0.446	-0.0892	No
15	PITG_09796	PITG_09796			9405	0.210	-0.1290	No
16	PITG_01698	PITG_01698			9578	0.182	-0.1319	No
17	PITG_12141	PITG_12141			9653	0.170	-0.1315	No
18	PITG_19772	PITG_19772			9955	0.115	-0.1404	No
19	PITG_13912	PITG_13912			10076	0.099	-0.1430	No
20	PITG_04452	PITG_04452			10353	0.050	-0.1521	No
21	PITG_12905	PITG_12905			10544	0.016	-0.1587	No
22	PITG_10862	PITG_10862			20924	-0.057	-0.5351	No
23	PITG_09472	PITG_09472			21065	-0.085	-0.5387	No
24	PITG_07860	PITG_07860			21091	-0.091	-0.5379	No
25	PITG_13728	PITG_13728			21209	-0.114	-0.5401	No
26	PITG_00688	PITG_00688			21852	-0.227	-0.5594	No
27	PITG_03344	PITG_03344			21954	-0.248	-0.5586	No
28	PITG_00693	PITG_00693			22125	-0.281	-0.5596	Yes
29	PITG_09031	PITG_09031			22128	-0.282	-0.5546	Yes
30	PITG_12427	PITG_12427			22247	-0.313	-0.5533	Yes
31	PITG_09015	PITG_09015			22408	-0.359	-0.5526	Yes
32	PITG_12520	PITG_12520			22558	-0.395	-0.5509	Yes
33	PITG_10951	PITG_10951			22677	-0.425	-0.5475	Yes
34	PITG_12309	PITG_12309			22918	-0.491	-0.5474	Yes
35	PITG_14362	PITG_14362			22976	-0.510	-0.5402	Yes
36	PITG_05840	PITG_05840			23498	-0.670	-0.5471	Yes
37	PITG_09436	PITG_09436			23835	-0.797	-0.5449	Yes
38	PITG_11452	PITG_11452			23908	-0.824	-0.5326	Yes

39	PITG_03033	PITG_03033			23973	-0.851	-0.5196	Yes
40	PITG_02045	PITG_02045			24208	-0.953	-0.5109	Yes
41	PITG_09438	PITG_09438			24364	-1.013	-0.4982	Yes
42	PITG_00997	PITG_00997			24484	-1.064	-0.4833	Yes
43	PITG_15553	PITG_15553			24493	-1.068	-0.4643	Yes
44	PITG_12049	PITG_12049			24527	-1.084	-0.4459	Yes
45	PITG_03632	PITG_03632			24602	-1.118	-0.4284	Yes
46	PITG_13683	PITG_13683			24705	-1.169	-0.4110	Yes
47	PITG_11036	PITG_11036			24973	-1.318	-0.3969	Yes
48	PITG_14612	PITG_14612			25098	-1.399	-0.3762	Yes
49	PITG_06595	PITG_06595			25183	-1.453	-0.3530	Yes
50	PITG_23229	PITG_23229			25245	-1.484	-0.3284	Yes
51	PITG_18776	PITG_18776			25283	-1.512	-0.3024	Yes
52	PITG_09445	PITG_09445			25332	-1.541	-0.2763	Yes
53	PITG_08565	PITG_08565			25336	-1.546	-0.2485	Yes
54	PITG_07792	PITG_07792			25364	-1.561	-0.2213	Yes
55	PITG_15526	PITG_15526			25559	-1.690	-0.1978	Yes
56	PITG_12264	PITG_12264			25836	-1.900	-0.1735	Yes
57	PITG_19880	PITG_19880			25946	-1.986	-0.1416	Yes
58	PITG_14936	PITG_14936			26019	-2.053	-0.1071	Yes
59	PITG_18354	PITG_18354			26104	-2.132	-0.0717	Yes
60	PITG_13682	PITG_13682			26239	-2.270	-0.0355	Yes
61	PITG_17921	PITG_17921			26277	-2.310	0.0048	Yes
62	PITG_09547	PITG_09547			26285	-2.315	0.0464	Yes

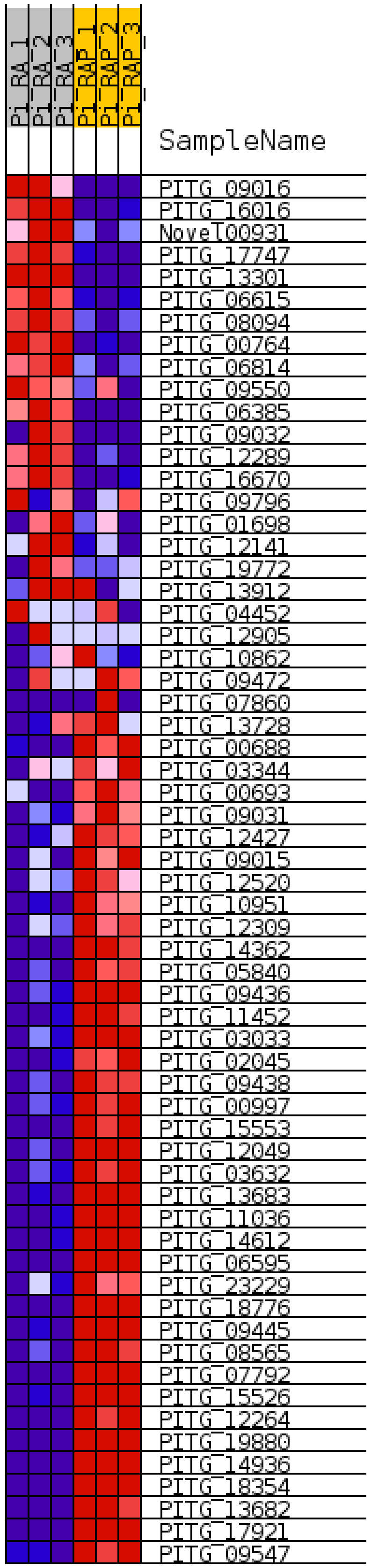

Fig 2: OXIDATIVE_PHOSPHORYLATION(PIF00190)
Blue-Pink O' Gram in the Space of the Analyzed GeneSet

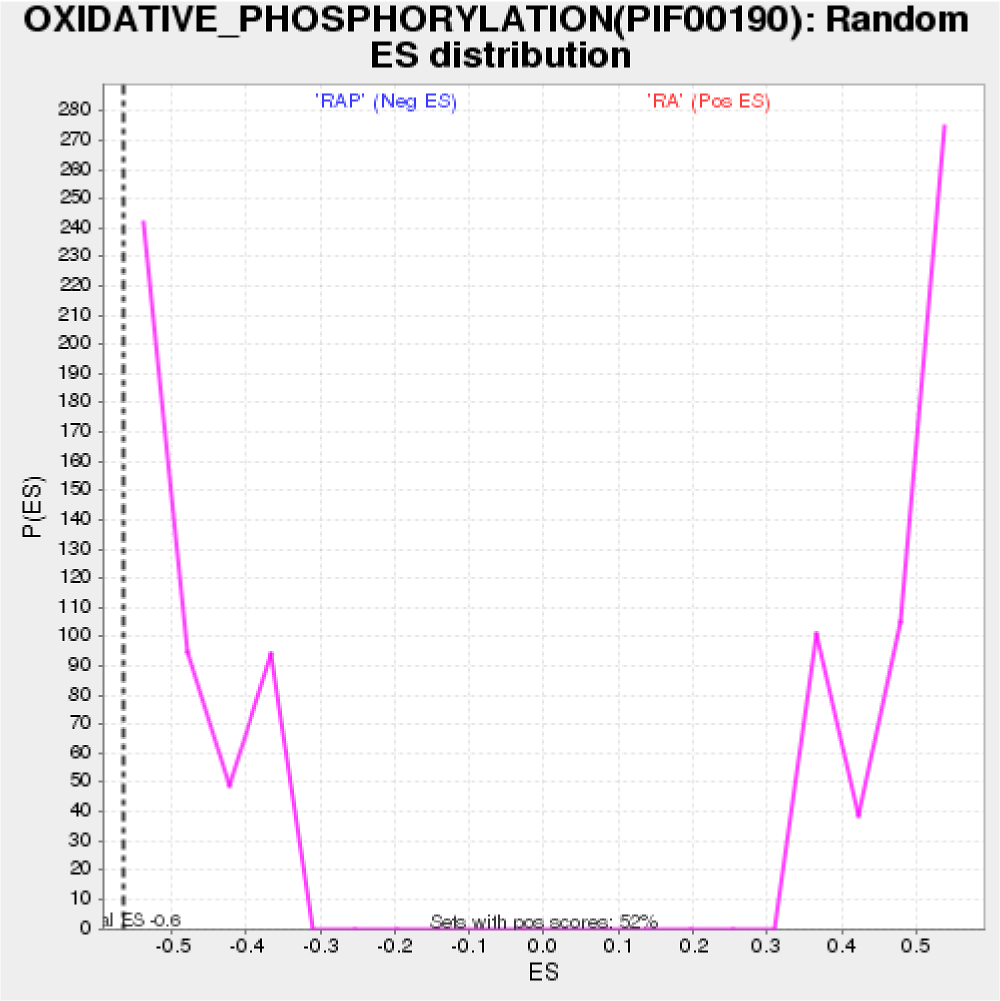

Fig 3: OXIDATIVE_PHOSPHORYLATION(PIF00190): Random ES distribution
Gene set null distribution of ES for OXIDATIVE_PHOSPHORYLATION(PIF00190)

5. Pentose phosphate pathway

Table: GSEA Results Summary

Dataset	fpkm.sample
Phenotype	sample.cls
Upregulated in class	RAP
GeneSet	PENTOSE_PHOSPHATE_PATHWAY(PIF00030)
Enrichment Score (ES)	-0.6685241
Normalized Enrichment Score (NES)	-1.2184933
Nominal p-value	0.0
FDR q-value	0.10208338
FWER p-Value	0.049

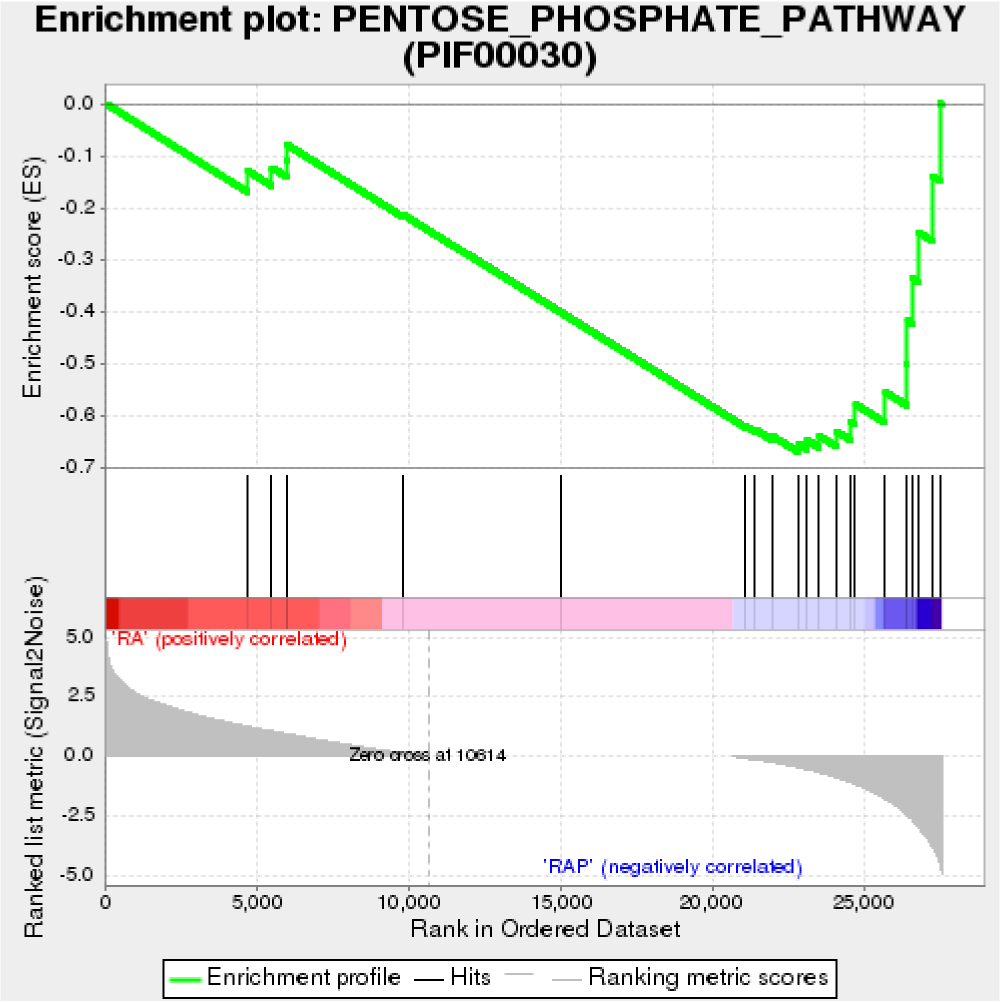

Fig 1: Enrichment plot: PENTOSE_PHOSPHATE_PATHWAY(PIF00030)
Profile of the Running ES Score & Positions of GeneSet Members on the Rank Ordered List

Table: GSEA details [plain text format]

	PROBE	DESCRIPTION	GENE	GENE_TITLE	RANK IN	RANK	RUNNING	CORE

		(from dataset)	SYMBOL		GENE LIST	METRIC SCORE	ES	ENRICHMENT
1	PITG_02038	PITG_02038			4671	1.266	-0.1280	No
2	PITG_00146	PITG_00146			5469	1.062	-0.1221	No
3	PITG_10032	PITG_10032			5953	0.951	-0.1084	No
4	PITG_08598	PITG_08598			5987	0.945	-0.0786	No
5	PITG_01752	PITG_01752			9804	0.142	-0.2125	No
6	PITG_03920	PITG_03920			15005	0.000	-0.4013	No
7	PITG_02785	PITG_02785			21080	-0.089	-0.6190	No
8	PITG_04665	PITG_04665			21414	-0.151	-0.6261	No
9	PITG_03598	PITG_03598			21994	-0.254	-0.6388	No
10	PITG_01862	PITG_01862			22814	-0.464	-0.6533	Yes
11	PITG_18414	PITG_18414			23131	-0.558	-0.6465	Yes
12	PITG_03919	PITG_03919			23530	-0.684	-0.6385	Yes
13	PITG_21397	PITG_21397			24090	-0.897	-0.6293	Yes
14	PITG_19174	PITG_19174			24562	-1.097	-0.6104	Yes
15	PITG_05636	PITG_05636			24720	-1.175	-0.5775	Yes
16	PITG_20687	PITG_20687			25706	-1.801	-0.5542	Yes
17	PITG_09817	PITG_09817			26422	-2.482	-0.4987	Yes
18	PITG_03992	PITG_03992			26434	-2.495	-0.4172	Yes
19	PITG_02129	PITG_02129			26586	-2.657	-0.3354	Yes
20	PITG_05551	PITG_05551			26774	-2.956	-0.2451	Yes
21	PITG_04624	PITG_04624			27253	-3.799	-0.1378	Yes
22	PITG_21468	PITG_21468			27507	-4.536	0.0020	Yes

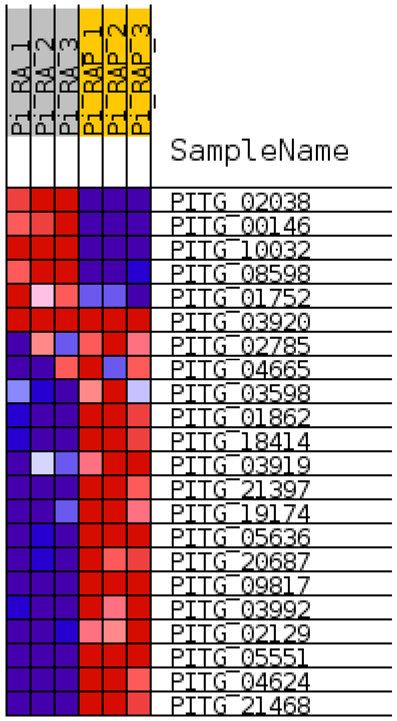

Fig 2: PENTOSE_PHOSPHATE_PATHWAY(PIF00030)
Blue-Pink O' Gram in the Space of the Analyzed GeneSet

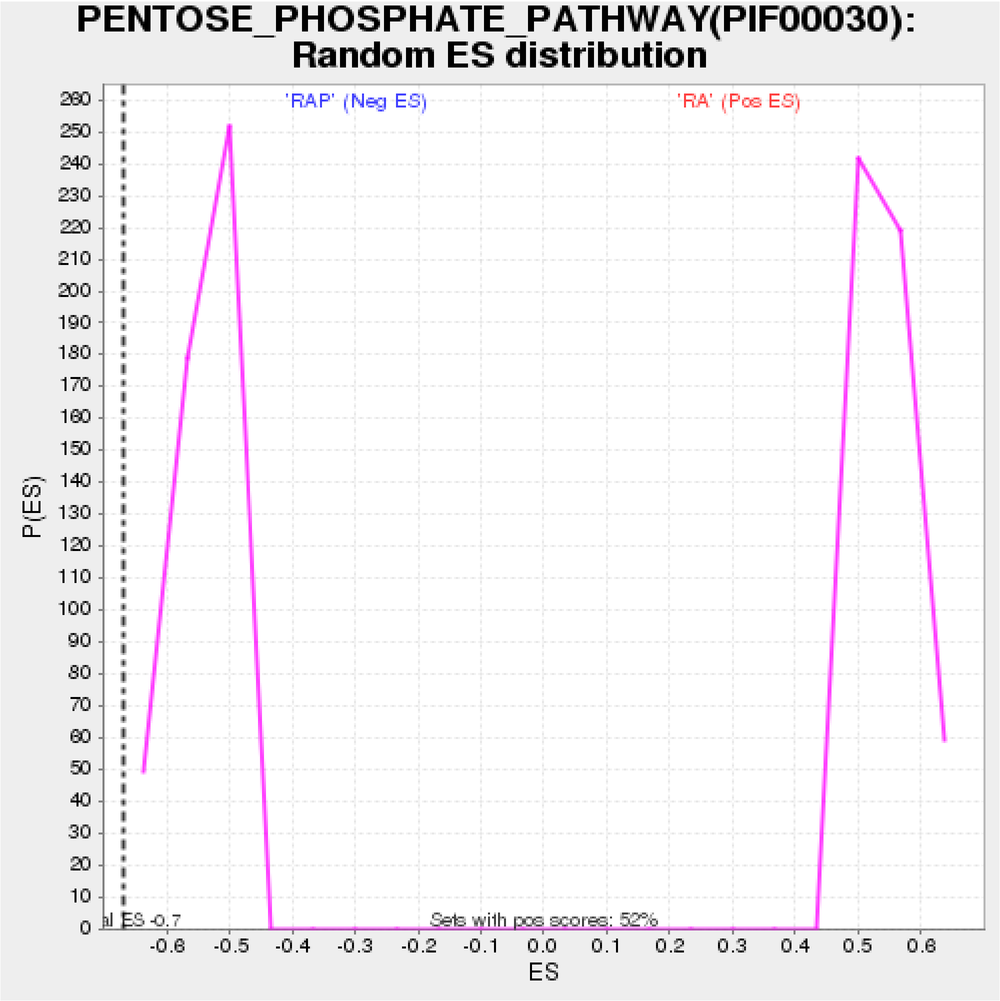

Fig 3: PENTOSE_PHOSPHATE_PATHWAY(PIF00030): Random ES distribution
Gene set null distribution of ES for PENTOSE_PHOSPHATE_PATHWAY(PIF00030)

6. Protein processing in endoplasmic reticulum

Table: GSEA Results Summary

Dataset	fpkm.sample
Phenotype	sample.cls
Upregulated in class	RAP
GeneSet	PROTEIN_PROCESSING_IN_ENDOPLASMIC_RETICULUM(PIF04141)
Enrichment Score (ES)	-0.4980084
Normalized Enrichment Score (NES)	-1.1678841
Nominal p-value	0.0
FDR q-value	0.10208329
FWER p-Value	0.049

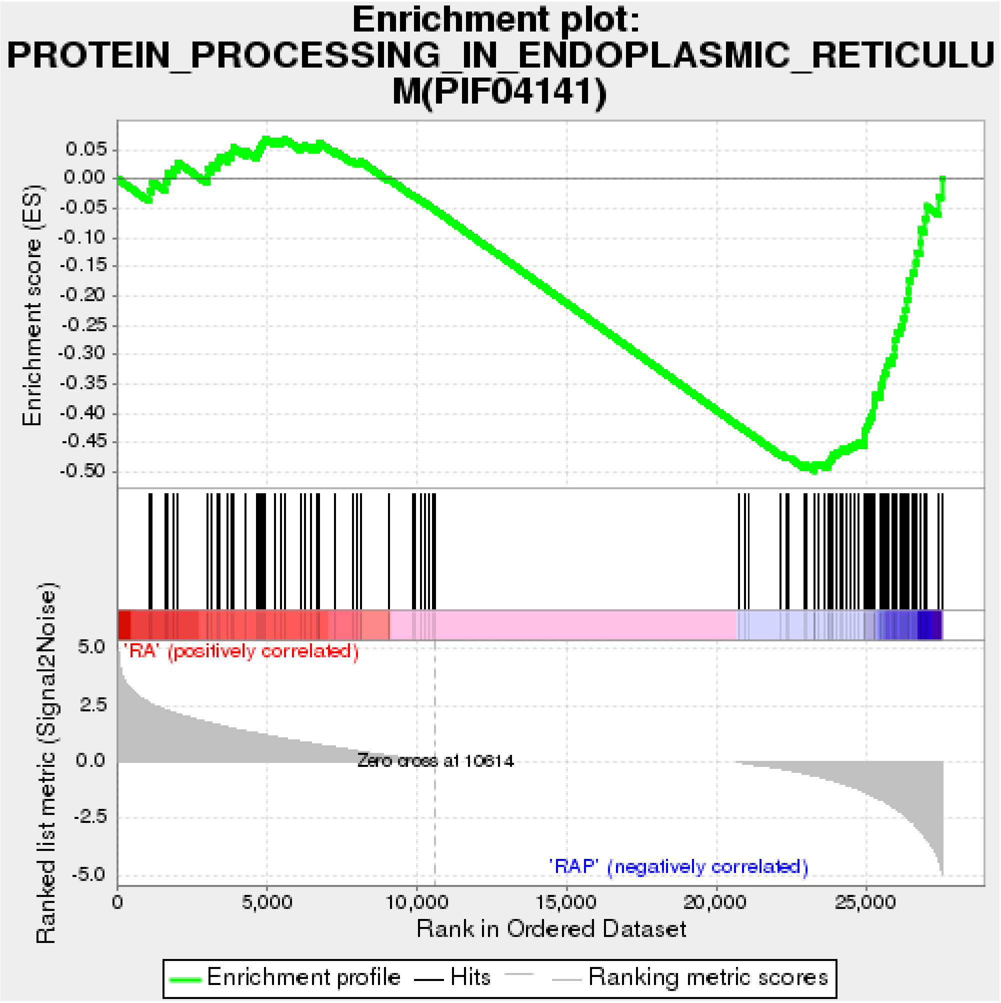

Fig 1: Enrichment plot: PROTEIN_PROCESSING_IN_ENDOPLASMIC_RETICULUM(PIF04141) Profile of the Running ES Score & Positions of GeneSet Members on the Rank Ordered List

Table: GSEA details [plain text format]

	PROBE	DESCRIPTION (from dataset)	GENE SYMBOL	GENE_TITLE	RANK IN GENE LIST	RANK METRIC SCORE	RUNNING ES	CORE ENRICHMENT
1	PITG_11249	PITG_11249			1009	2.632	-0.0179	No
2	PITG_14396	PITG_14396			1130	2.544	-0.0040	No
3	PITG_11247	PITG_11247			1568	2.314	-0.0034	No
4	PITG_15216	PITG_15216			1613	2.295	0.0114	No
5	PITG_21580	PITG_21580			1838	2.201	0.0190	No
6	PITG_06946	PITG_06946			1974	2.139	0.0294	No
7	PITG_01181	PITG_01181			2966	1.749	0.0059	No
8	PITG_11728	PITG_11728			2986	1.744	0.0177	No
9	PITG_12120	PITG_12120			3138	1.698	0.0243	No
10	PITG_17879	PITG_17879			3293	1.648	0.0305	No
11	PITG_16065	PITG_16065			3381	1.620	0.0390	No
12	PITG_07083	PITG_07083			3634	1.536	0.0408	No
13	PITG_15761	PITG_15761			3801	1.488	0.0454	No
14	PITG_08469	PITG_08469			3816	1.485	0.0555	No
15	PITG_05781	PITG_05781			4239	1.372	0.0500	No
16	PITG_01382	PITG_01382			4632	1.276	0.0448	No
17	PITG_13434	PITG_13434			4701	1.260	0.0514	No
18	PITG_10149	PITG_10149			4761	1.243	0.0581	No
19	PITG_17383	PITG_17383			4868	1.214	0.0629	No
20	PITG_10857	PITG_10857			4907	1.203	0.0702	No
21	PITG_11252	PITG_11252			5251	1.112	0.0657	No
22	PITG_06885	PITG_06885			5449	1.066	0.0661	No
23	PITG_06659	PITG_06659			5567	1.034	0.0693	No
24	PITG_18037	PITG_18037			6091	0.919	0.0568	No
25	PITG_09453	PITG_09453			6218	0.892	0.0586	No
26	PITG_11886	PITG_11886			6468	0.829	0.0555	No
27	PITG_09377	PITG_09377			6639	0.794	0.0549	No
28	PITG_02211	PITG_02211			6700	0.777	0.0583	No
29	PITG_13748	PITG_13748			6711	0.774	0.0635	No
30	PITG_14939	PITG_14939			7264	0.653	0.0481	No
31	PITG_01524	PITG_01524			7821	0.530	0.0316	No
32	PITG_15998	PITG_15998			8012	0.486	0.0282	No
33	PITG_02584	PITG_02584			8095	0.467	0.0285	No
34	PITG_16137	PITG_16137			8118	0.462	0.0310	No
35	PITG_19537	PITG_19537			9071	0.272	-0.0017	No
36	PITG_00505	PITG_00505			9078	0.270	0.0000	No
37	PITG_06505	PITG_06505			9858	0.131	-0.0274	No

38	PITG_16528	PITG_16528			9893	0.126	-0.0277	No
39	PITG_19871	PITG_19871			10104	0.093	-0.0347	No
40	PITG_10020	PITG_10020			10289	0.062	-0.0410	No
41	PITG_13234	PITG_13234			10376	0.046	-0.0438	No
42	PITG_00089	PITG_00089			10553	0.015	-0.0501	No
43	PITG_17814	PITG_17814			10595	0.006	-0.0515	No
44	PITG_22715	PITG_22715			20744	-0.014	-0.4210	No
45	PITG_07201	PITG_07201			20983	-0.069	-0.4292	No
46	PITG_13370	PITG_13370			21053	-0.083	-0.4311	No
47	PITG_06992	PITG_06992			22163	-0.293	-0.4694	No
48	PITG_01260	PITG_01260			22362	-0.344	-0.4741	No
49	PITG_02867	PITG_02867			22441	-0.367	-0.4743	No
50	PITG_12881	PITG_12881			22980	-0.512	-0.4903	No
51	PITG_20714	PITG_20714			23009	-0.521	-0.4876	No
52	PITG_11712	PITG_11712			23297	-0.605	-0.4937	Yes
53	PITG_03068	PITG_03068			23311	-0.612	-0.4898	Yes
54	PITG_04610	PITG_04610			23321	-0.613	-0.4857	Yes
55	PITG_09693	PITG_09693			23451	-0.656	-0.4857	Yes
56	PITG_02597	PITG_02597			23607	-0.717	-0.4862	Yes
57	PITG_03694	PITG_03694			23745	-0.765	-0.4857	Yes
58	Novel00522	Novel00522			23775	-0.774	-0.4812	Yes
59	PITG_01058	PITG_01058			23827	-0.794	-0.4774	Yes
60	PITG_05498	PITG_05498			23882	-0.815	-0.4736	Yes
61	PITG_07061	PITG_07061			23916	-0.826	-0.4688	Yes
62	PITG_21378	PITG_21378			24003	-0.860	-0.4658	Yes
63	PITG_19557	PITG_19557			24128	-0.914	-0.4638	Yes
64	PITG_03390	PITG_03390			24204	-0.951	-0.4597	Yes
65	PITG_18107	PITG_18107			24385	-1.025	-0.4589	Yes
66	PITG_06657	PITG_06657			24465	-1.058	-0.4542	Yes
67	PITG_10972	PITG_10972			24601	-1.115	-0.4512	Yes
68	PITG_11703	PITG_11703			24755	-1.194	-0.4482	Yes
69	PITG_16366	PITG_16366			24945	-1.308	-0.4457	Yes
70	PITG_05672	PITG_05672			24951	-1.308	-0.4365	Yes
71	PITG_03468	PITG_03468			24981	-1.321	-0.4281	Yes
72	PITG_13151	PITG_13151			25011	-1.341	-0.4196	Yes
73	PITG_11704	PITG_11704			25093	-1.396	-0.4125	Yes
74	PITG_03594	PITG_03594			25177	-1.448	-0.4052	Yes
75	PITG_18053	PITG_18053			25243	-1.483	-0.3969	Yes
76	PITG_01855	PITG_01855			25264	-1.500	-0.3869	Yes

77	PITG_12694	PITG_12694			25300	-1.523	-0.3773	Yes
78	PITG_12260	PITG_12260			25325	-1.537	-0.3671	Yes
79	PITG_17592	PITG_17592			25491	-1.646	-0.3614	Yes
80	PITG_07885	PITG_07885			25526	-1.673	-0.3506	Yes
81	PITG_09965	PITG_09965			25590	-1.707	-0.3407	Yes
82	PITG_06795	PITG_06795			25599	-1.716	-0.3287	Yes
83	PITG_08327	PITG_08327			25708	-1.802	-0.3197	Yes
84	PITG_11244	PITG_11244			25734	-1.825	-0.3076	Yes
85	PITG_03388	PITG_03388			25928	-1.978	-0.3004	Yes
86	PITG_07843	PITG_07843			25951	-1.992	-0.2870	Yes
87	PITG_00632	PITG_00632			25974	-2.018	-0.2733	Yes
88	PITG_17683	PITG_17683			26041	-2.071	-0.2609	Yes
89	PITG_05501	PITG_05501			26156	-2.189	-0.2494	Yes
90	PITG_02266	PITG_02266			26258	-2.295	-0.2366	Yes
91	PITG_15358	PITG_15358			26325	-2.357	-0.2222	Yes
92	PITG_08328	PITG_08328			26375	-2.420	-0.2066	Yes
93	PITG_18934	PITG_18934			26421	-2.479	-0.1905	Yes
94	PITG_15771	PITG_15771			26455	-2.517	-0.1737	Yes
95	PITG_02467	PITG_02467			26534	-2.594	-0.1579	Yes
96	PITG_00527	PITG_00527			26666	-2.775	-0.1428	Yes
97	PITG_18089	PITG_18089			26695	-2.815	-0.1237	Yes
98	PITG_22023	PITG_22023			26818	-3.016	-0.1065	Yes
99	PITG_01858	PITG_01858			26836	-3.047	-0.0854	Yes
100	PITG_17637	PITG_17637			26946	-3.226	-0.0662	Yes
101	PITG_09451	PITG_09451			27010	-3.332	-0.0447	Yes
102	PITG_03546	PITG_03546			27449	-4.309	-0.0298	Yes
103	PITG_11913	PITG_11913			27537	-4.720	0.0009	Yes

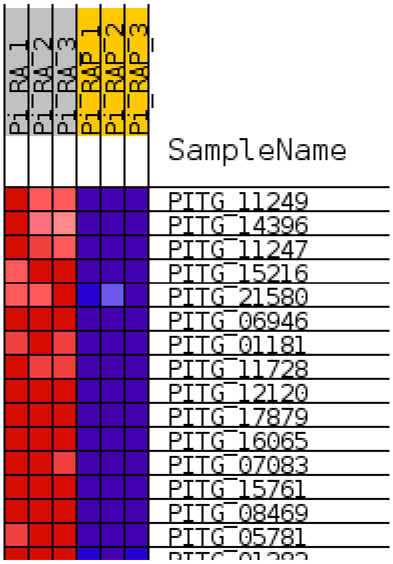

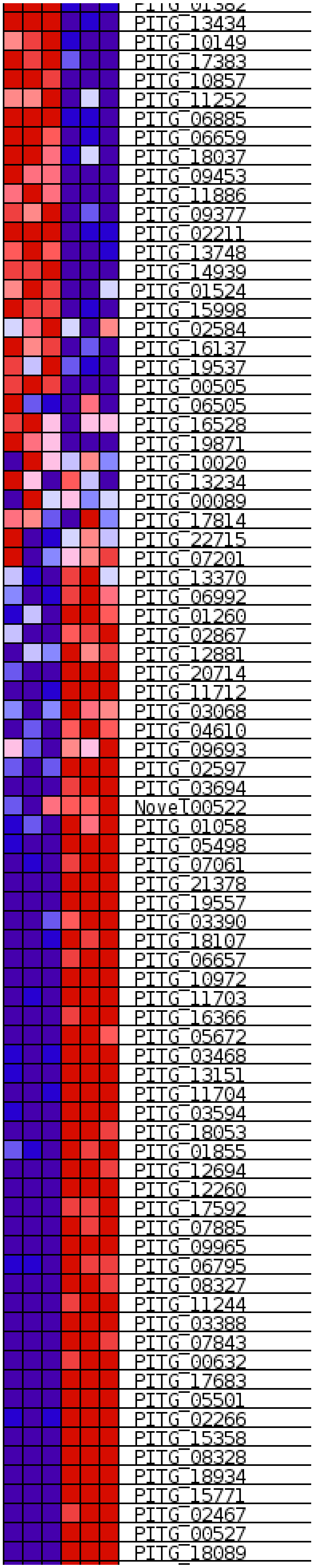
PITG_13434
PITG_10149
PITG_17383
PITG_10857
PITG_11252
PITG_06885
PITG_06659
PITG_18037
PITG_09453
PITG_11886
PITG_09377
PITG_02211
PITG_13748
PITG_14939
PITG_01524
PITG_15998
PITG_02584
PITG_16137
PITG_19537
PITG_00505
PITG_06505
PITG_16528
PITG_19871
PITG_10020
PITG_13234
PITG_00089
PITG_17814
PITG_22715
PITG_07201
PITG_13370
PITG_06992
PITG_01260
PITG_02867
PITG_12881
PITG_20714
PITG_11712
PITG_03068
PITG_04610
PITG_09693
PITG_02597
PITG_03694
Novel00522
PITG_01058
PITG_05498
PITG_07061
PITG_21378
PITG_19557
PITG_03390
PITG_18107
PITG_06657
PITG_10972
PITG_11703
PITG_16366
PITG_05672
PITG_03468
PITG_13151
PITG_11704
PITG_03594
PITG_18053
PITG_01855
PITG_12694
PITG_12260
PITG_17592
PITG_07885
PITG_09965
PITG_06795
PITG_08327
PITG_11244
PITG_03388
PITG_07843
PITG_00632
PITG_17683
PITG_05501
PITG_02266
PITG_15358
PITG_08328
PITG_18934
PITG_15771
PITG_02467
PITG_00527
PITG_18089

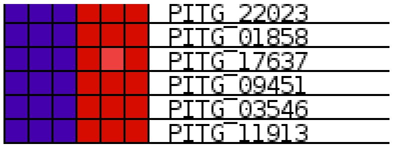

Fig 2: PROTEIN_PROCESSING_IN_ENDOPLASMIC_RETICULUM(PIF04141) Blue-Pink O' Gram in the Space of the Analyzed GeneSet

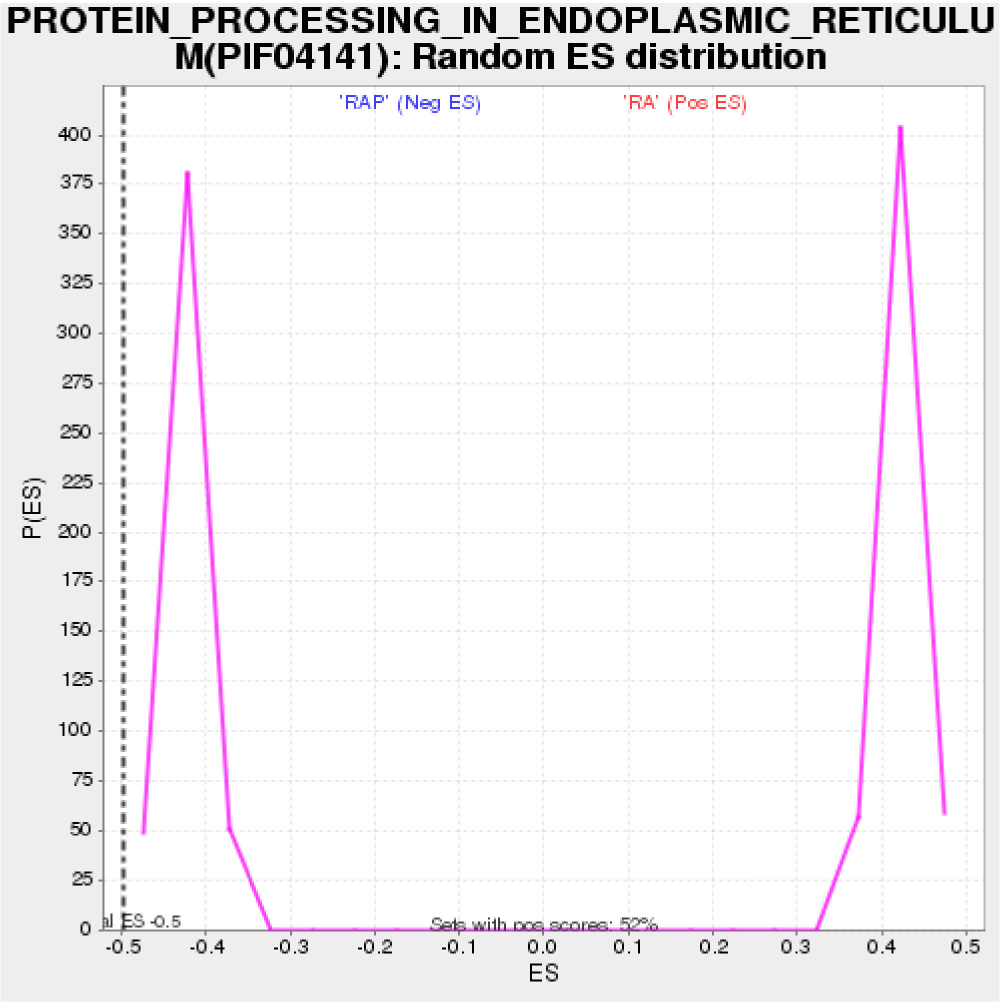

Fig 3: PROTEIN_PROCESSING_IN_ENDOPLASMIC_RETICULUM(PIF04141): Random ES distribution
Gene set null distribution of ES for
PROTEIN_PROCESSING_IN_ENDOPLASMIC_RETICULUM(PIF04141)

7. Ribosome

Table: GSEA Results Summary

Dataset	fpkm.sample
Phenotype	sample.cls
Upregulated in class	RAP
GeneSet	RIBOSOME(PIF03010)
Enrichment Score (ES)	-0.8566887
Normalized Enrichment Score (NES)	-1.0569768
Nominal p-value	0.0
FDR q-value	0.2048615
FWER p-Value	0.346

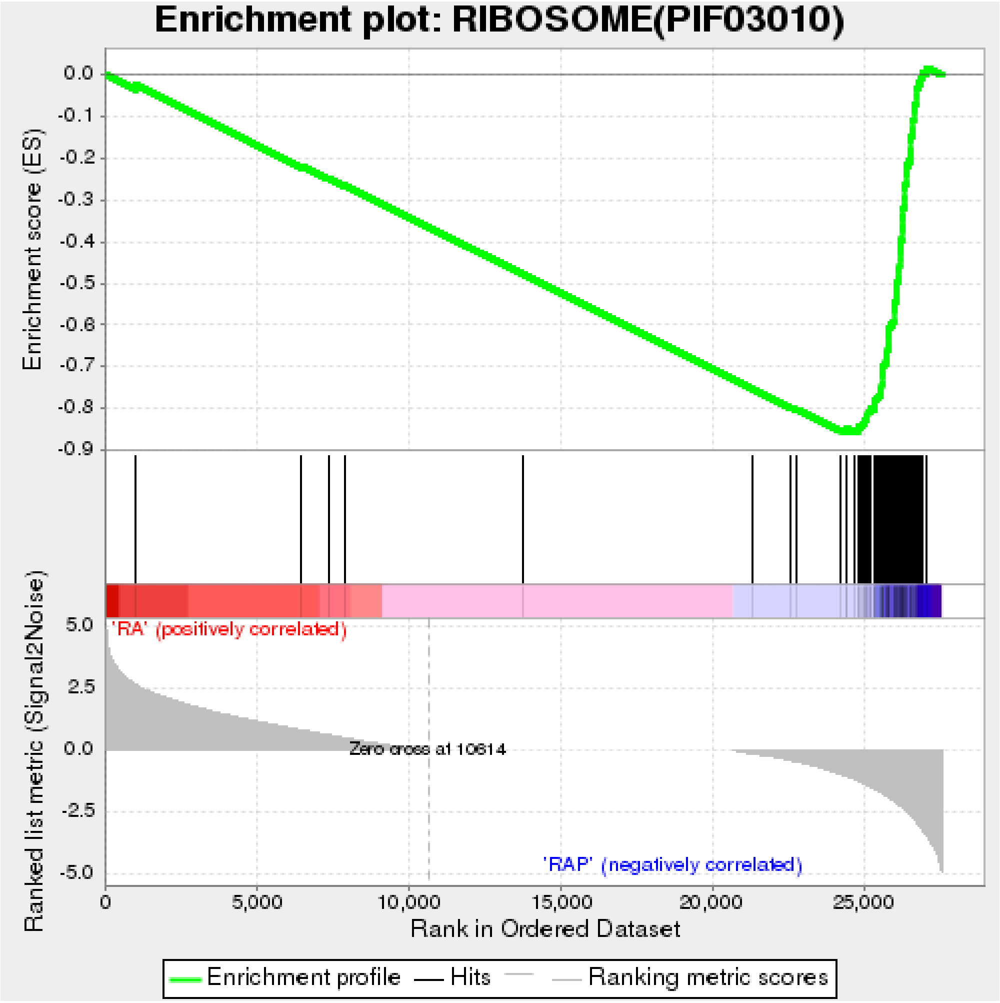

Fig 1: Enrichment plot: RIBOSOME(PIF03010)
Profile of the Running ES Score & Positions of GeneSet Members on the Rank Ordered List

Table: GSEA details [plain text format]

	PROBE	DESCRIPTION	GENE	GENE_TITLE	RANK IN	RANK	RUNNING	CORE

		(from dataset)	SYMBOL		GENE LIST	METRIC SCORE	ES	ENRICHMENT
1	PITG_19121	PITG_19121			962	2.666	-0.0221	No
2	PITG_03660	PITG_03660			6456	0.832	-0.2181	No
3	PITG_09430	PITG_09430			7325	0.637	-0.2466	No
4	PITG_04337	PITG_04337			7866	0.519	-0.2637	No
5	PITG_15722	PITG_15722			13740	0.000	-0.4776	No
6	PITG_05009	PITG_05009			21324	-0.134	-0.7532	No
7	PITG_10193	PITG_10193			22606	-0.404	-0.7979	No
8	PITG_11734	PITG_11734			22780	-0.455	-0.8019	No
9	PITG_19669	PITG_19669			24217	-0.958	-0.8496	No
10	PITG_16757	PITG_16757			24413	-1.036	-0.8517	Yes
11	PITG_09442	PITG_09442			24425	-1.041	-0.8470	Yes
12	PITG_13500	PITG_13500			24681	-1.158	-0.8506	Yes
13	PITG_19999	PITG_19999			24792	-1.214	-0.8488	Yes
14	PITG_19007	PITG_19007			24804	-1.221	-0.8432	Yes
15	PITG_03916	PITG_03916			24891	-1.273	-0.8402	Yes
16	PITG_20189	PITG_20189			24966	-1.317	-0.8365	Yes
17	PITG_01922	PITG_01922			25002	-1.336	-0.8312	Yes
18	PITG_12839	PITG_12839			25031	-1.352	-0.8257	Yes
19	PITG_14850	PITG_14850			25066	-1.380	-0.8202	Yes
20	PITG_16008	PITG_16008			25114	-1.410	-0.8151	Yes
21	PITG_06771	PITG_06771			25118	-1.413	-0.8083	Yes
22	PITG_00443	PITG_00443			25193	-1.456	-0.8039	Yes
23	PITG_04843	PITG_04843			25217	-1.471	-0.7976	Yes
24	PITG_05171	PITG_05171			25340	-1.548	-0.7945	Yes
25	PITG_12745	PITG_12745			25347	-1.553	-0.7872	Yes
26	PITG_09563	PITG_09563			25371	-1.565	-0.7804	Yes
27	PITG_00266	PITG_00266			25399	-1.581	-0.7737	Yes
28	PITG_20188	PITG_20188			25502	-1.654	-0.7694	Yes
29	PITG_12697	PITG_12697			25529	-1.673	-0.7622	Yes
30	PITG_21503	PITG_21503			25544	-1.681	-0.7545	Yes
31	PITG_08834	PITG_08834			25576	-1.699	-0.7474	Yes
32	PITG_15697	PITG_15697			25580	-1.701	-0.7393	Yes
33	PITG_10887	PITG_10887			25597	-1.713	-0.7315	Yes
34	PITG_07269	PITG_07269			25598	-1.716	-0.7232	Yes
35	PITG_13676	PITG_13676			25606	-1.722	-0.7150	Yes
36	PITG_07300	PITG_07300			25611	-1.727	-0.7068	Yes
37	PITG_03221	PITG_03221			25613	-1.729	-0.6984	Yes
38	PITG_15090	PITG_15090			25650	-1.757	-0.6912	Yes

39	PITG_08703	PITG_08703			25716	-1.807	-0.6848	Yes
40	PITG_17093	PITG_17093			25724	-1.809	-0.6762	Yes
41	PITG_16198	PITG_16198			25760	-1.842	-0.6686	Yes
42	PITG_02694	PITG_02694			25772	-1.849	-0.6600	Yes
43	PITG_15069	PITG_15069			25785	-1.859	-0.6514	Yes
44	PITG_01943	PITG_01943			25791	-1.864	-0.6425	Yes
45	PITG_15407	PITG_15407			25795	-1.868	-0.6335	Yes
46	PITG_10263	PITG_10263			25797	-1.869	-0.6245	Yes
47	PITG_11766	PITG_11766			25814	-1.881	-0.6159	Yes
48	PITG_01833	PITG_01833			25831	-1.897	-0.6073	Yes
49	PITG_09627	PITG_09627			25860	-1.924	-0.5989	Yes
50	PITG_20264	PITG_20264			25950	-1.992	-0.5925	Yes
51	PITG_07173	PITG_07173			25980	-2.022	-0.5837	Yes
52	PITG_19157	PITG_19157			25981	-2.022	-0.5739	Yes
53	PITG_10863	PITG_10863			25982	-2.022	-0.5640	Yes
54	PITG_09540	PITG_09540			25997	-2.035	-0.5547	Yes
55	PITG_03353	PITG_03353			26026	-2.059	-0.5457	Yes
56	PITG_09506	PITG_09506			26047	-2.074	-0.5363	Yes
57	PITG_00941	PITG_00941			26052	-2.078	-0.5264	Yes
58	PITG_14913	PITG_14913			26073	-2.104	-0.5168	Yes
59	PITG_13312	PITG_13312			26090	-2.122	-0.5071	Yes
60	PITG_04487	PITG_04487			26098	-2.129	-0.4970	Yes
61	PITG_17261	PITG_17261			26116	-2.142	-0.4872	Yes
62	PITG_03235	PITG_03235			26130	-2.165	-0.4772	Yes
63	PITG_22135	PITG_22135			26132	-2.168	-0.4667	Yes
64	PITG_06995	PITG_06995			26169	-2.197	-0.4573	Yes
65	PITG_08959	PITG_08959			26174	-2.203	-0.4467	Yes
66	PITG_05174	PITG_05174			26178	-2.205	-0.4361	Yes
67	PITG_03239	PITG_03239			26180	-2.206	-0.4254	Yes
68	PITG_11099	PITG_11099			26200	-2.234	-0.4153	Yes
69	PITG_03294	PITG_03294			26205	-2.241	-0.4045	Yes
70	PITG_14608	PITG_14608			26218	-2.248	-0.3940	Yes
71	PITG_20798	PITG_20798			26248	-2.286	-0.3840	Yes
72	PITG_09555	PITG_09555			26249	-2.286	-0.3728	Yes
73	PITG_00179	PITG_00179			26253	-2.292	-0.3618	Yes
74	PITG_03477	PITG_03477			26259	-2.296	-0.3508	Yes
75	PITG_10202	PITG_10202			26263	-2.298	-0.3398	Yes
76	PITG_12947	PITG_12947			26294	-2.326	-0.3295	Yes
77	PITG_18052	PITG_18052			26295	-2.327	-0.3182	Yes

78	PITG_15638	PITG_15638			26332	-2.371	-0.3080	Yes
79	PITG_13371	PITG_13371			26336	-2.376	-0.2966	Yes
80	PITG_03486	PITG_03486			26347	-2.389	-0.2853	Yes
81	PITG_06636	PITG_06636			26348	-2.389	-0.2737	Yes
82	PITG_01217	PITG_01217			26362	-2.408	-0.2625	Yes
83	PITG_03762	PITG_03762			26390	-2.434	-0.2516	Yes
84	PITG_03178	PITG_03178			26392	-2.437	-0.2398	Yes
85	PITG_00523	PITG_00523			26405	-2.460	-0.2283	Yes
86	PITG_20795	PITG_20795			26409	-2.461	-0.2164	Yes
87	PITG_13681	PITG_13681			26460	-2.519	-0.2060	Yes
88	PITG_02053	PITG_02053			26508	-2.564	-0.1953	Yes
89	PITG_10146	PITG_10146			26517	-2.576	-0.1830	Yes
90	PITG_00631	PITG_00631			26545	-2.608	-0.1713	Yes
91	PITG_02578	PITG_02578			26546	-2.608	-0.1586	Yes
92	PITG_06237	PITG_06237			26557	-2.626	-0.1462	Yes
93	PITG_03420	PITG_03420			26604	-2.687	-0.1349	Yes
94	PITG_01042	PITG_01042			26613	-2.704	-0.1220	Yes
95	PITG_20116	PITG_20116			26615	-2.706	-0.1089	Yes
96	PITG_08548	PITG_08548			26637	-2.738	-0.0963	Yes
97	PITG_06799	PITG_06799			26639	-2.740	-0.0830	Yes
98	PITG_09345	PITG_09345			26676	-2.790	-0.0708	Yes
99	PITG_09552	PITG_09552			26705	-2.829	-0.0581	Yes
100	PITG_09525	PITG_09525			26708	-2.829	-0.0444	Yes
101	PITG_14729	PITG_14729			26712	-2.839	-0.0307	Yes
102	PITG_01041	PITG_01041			26784	-2.972	-0.0188	Yes
103	PITG_03768	PITG_03768			26845	-3.062	-0.0061	Yes
104	PITG_18054	PITG_18054			26907	-3.165	0.0071	Yes
105	PITG_22323	PITG_22323			27081	-3.441	0.0175	Yes

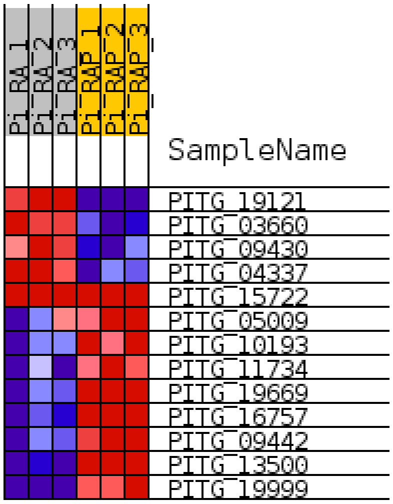

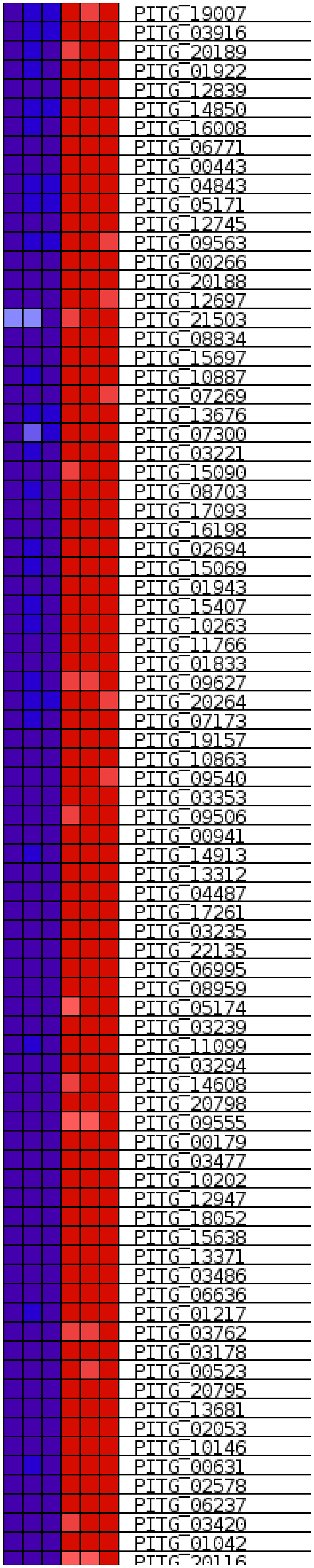

PITG_19007
PITG_03916
PITG_20189
PITG_01922
PITG_12839
PITG_14850
PITG_16008
PITG_06771
PITG_00443
PITG_04843
PITG_05171
PITG_12745
PITG_09563
PITG_00266
PITG_20188
PITG_12697
PITG_21503
PITG_08834
PITG_15697
PITG_10887
PITG_07269
PITG_13676
PITG_07300
PITG_03221
PITG_15090
PITG_08703
PITG_17093
PITG_16198
PITG_02694
PITG_15069
PITG_01943
PITG_15407
PITG_10263
PITG_11766
PITG_01833
PITG_09627
PITG_20264
PITG_07173
PITG_19157
PITG_10863
PITG_09540
PITG_03353
PITG_09506
PITG_00941
PITG_14913
PITG_13312
PITG_04487
PITG_17261
PITG_03235
PITG_22135
PITG_06995
PITG_08959
PITG_05174
PITG_03239
PITG_11099
PITG_03294
PITG_14608
PITG_20798
PITG_09555
PITG_00179
PITG_03477
PITG_10202
PITG_12947
PITG_18052
PITG_15638
PITG_13371
PITG_03486
PITG_06636
PITG_01217
PITG_03762
PITG_03178
PITG_00523
PITG_20795
PITG_13681
PITG_02053
PITG_10146
PITG_00631
PITG_02578
PITG_06237
PITG_03420
PITG_01042
PITG_20116

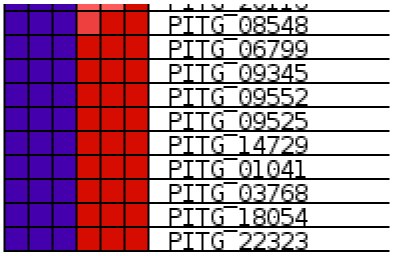

Fig 2: RIBOSOME(PIF03010)
lue-Pink O' Gram in the Space of the Analyzed GeneSet

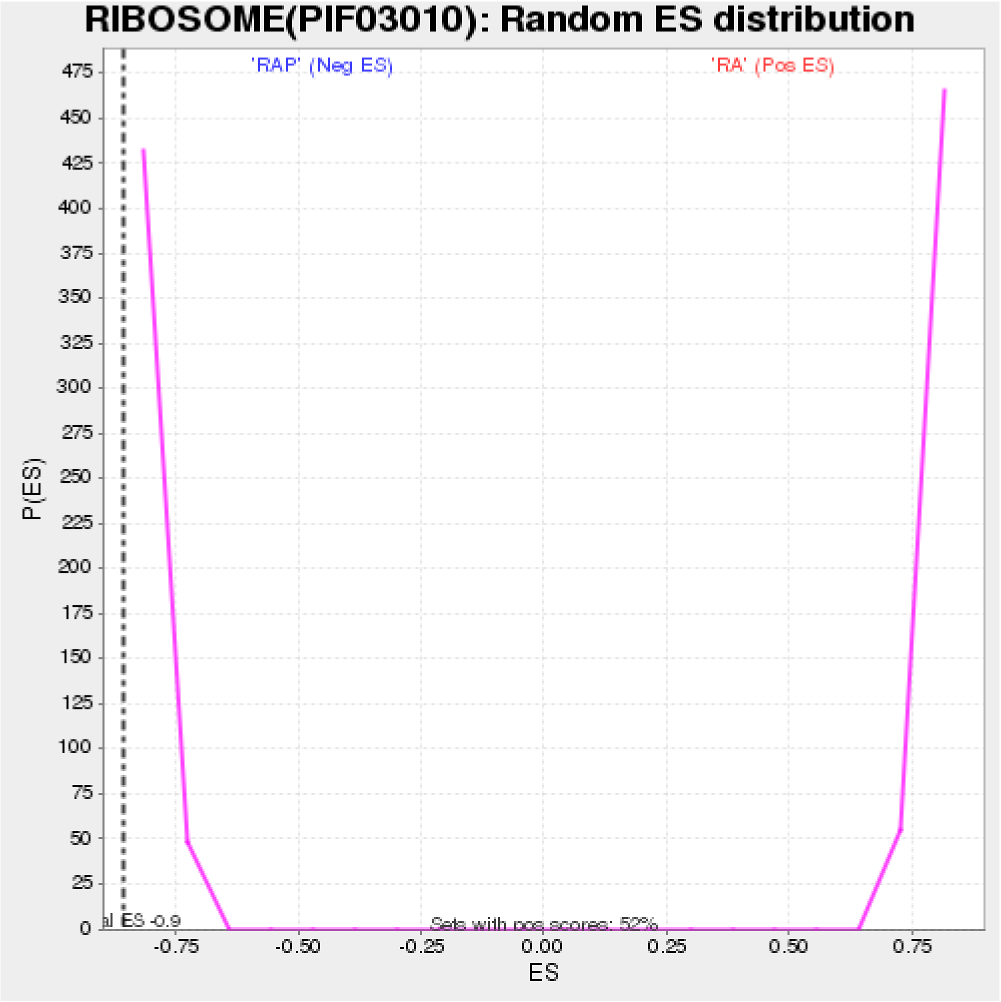

Fig 3: RIBOSOME(PIF03010): Random ES distribution
Gene set null distribution of ES for RIBOSOME(PIF03010)

8. Ribosome biogenesis in eukaryotes

Table: GSEA Results Summary

Dataset	fpkm.sample
Phenotype	sample.cls
Upregulated in class	RAP
GeneSet	RIBOSOME_BIOGENESIS_IN_EUKARYOTES(PIF03008)
Enrichment Score (ES)	-0.85391337
Normalized Enrichment Score (NES)	-1.2601833
Nominal p-value	0.0
FDR q-value	0.10208338
FWER p-Value	0.049

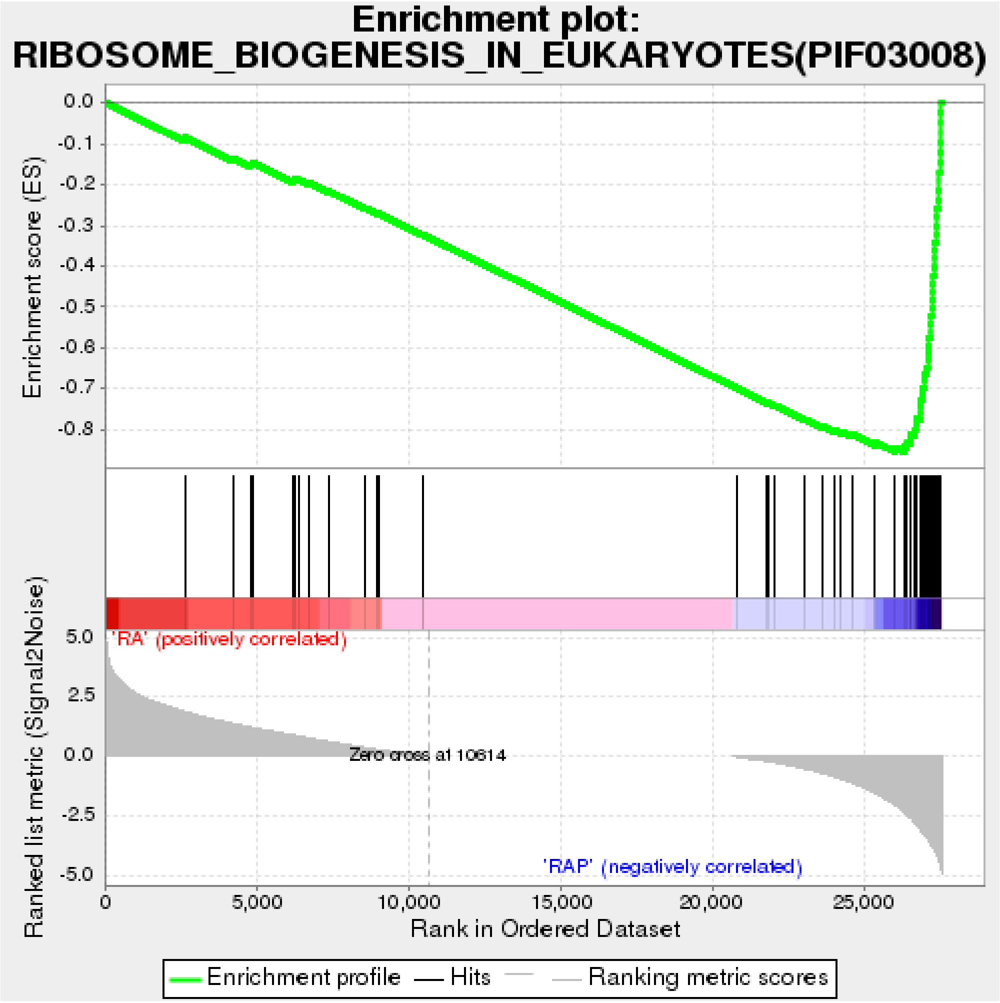

Fig 1: Enrichment plot: RIBOSOME_BIOGENESIS_IN_EUKARYOTES(PIF03008)
Profile of the Running ES Score & Positions of GeneSet Members on the Rank Ordered List

Table: GSEA details [plain text format]

	PROBE	DESCRIPTION	GENE	GENE_TITLE	RANK IN	RANK	RUNNING	CORE

		(from dataset)	SYMBOL		GENE LIST	METRIC SCORE	ES	ENRICHMENT
1	PITG_12932	PITG_12932			2577	1.892	-0.0838	No
2	PITG_00401	PITG_00401			4193	1.381	-0.1352	No
3	PITG_15975	PITG_15975			4750	1.246	-0.1489	No
4	PITG_22541	PITG_22541			4822	1.225	-0.1450	No
5	PITG_06679	PITG_06679			6155	0.907	-0.1887	No
6	PITG_05240	PITG_05240			6207	0.894	-0.1858	No
7	PITG_04584	PITG_04584			6363	0.854	-0.1870	No
8	PITG_08447	PITG_08447			6712	0.773	-0.1955	No
9	PITG_16556	PITG_16556			7335	0.634	-0.2148	No
10	PITG_06700	PITG_06700			8545	0.368	-0.2569	No
11	PITG_00194	PITG_00194			8957	0.294	-0.2703	No
12	PITG_00787	PITG_00787			8966	0.292	-0.2690	No
13	PITG_21600	PITG_21600			10426	0.033	-0.3219	No
14	PITG_02745	PITG_02745			10439	0.030	-0.3222	No
15	PITG_17674	PITG_17674			20814	-0.028	-0.6994	No
16	PITG_05058	PITG_05058			21819	-0.220	-0.7348	No
17	PITG_10163	PITG_10163			21826	-0.222	-0.7338	No
18	PITG_17205	PITG_17205			22054	-0.266	-0.7407	No
19	PITG_12107	PITG_12107			23062	-0.538	-0.7745	No
20	PITG_03802	PITG_03802			23630	-0.726	-0.7913	No
21	PITG_12465	PITG_12465			24025	-0.867	-0.8010	No
22	PITG_17724	PITG_17724			24244	-0.966	-0.8039	No
23	PITG_07174	PITG_07174			24615	-1.125	-0.8114	No
24	PITG_18421	PITG_18421			25379	-1.567	-0.8309	No
25	PITG_18718	PITG_18718			26011	-2.046	-0.8431	Yes
26	PITG_03818	PITG_03818			26310	-2.341	-0.8416	Yes
27	PITG_11635	PITG_11635			26431	-2.494	-0.8328	Yes
28	PITG_03672	PITG_03672			26510	-2.566	-0.8221	Yes
29	PITG_01725	PITG_01725			26536	-2.595	-0.8093	Yes
30	PITG_01076	PITG_01076			26691	-2.810	-0.8001	Yes
31	PITG_00032	PITG_00032			26702	-2.824	-0.7856	Yes
32	PITG_17404	PITG_17404			26734	-2.874	-0.7716	Yes
33	PITG_00035	PITG_00035			26847	-3.066	-0.7595	Yes
34	PITG_12871	PITG_12871			26851	-3.070	-0.7434	Yes
35	PITG_12305	PITG_12305			26887	-3.143	-0.7282	Yes
36	PITG_16813	PITG_16813			26919	-3.180	-0.7125	Yes
37	PITG_00748	PITG_00748			26940	-3.214	-0.6963	Yes
38	PITG_19671	PITG_19671			26975	-3.278	-0.6803	Yes

39	PITG_08708	PITG_08708			27004	-3.323	-0.6638	Yes
40	PITG_01019	PITG_01019			27065	-3.415	-0.6480	Yes
41	PITG_06262	PITG_06262			27099	-3.481	-0.6308	Yes
42	PITG_00036	PITG_00036			27107	-3.501	-0.6126	Yes
43	PITG_04887	PITG_04887			27117	-3.520	-0.5944	Yes
44	PITG_05630	PITG_05630			27143	-3.571	-0.5765	Yes
45	PITG_05445	PITG_05445			27198	-3.675	-0.5591	Yes
46	PITG_12931	PITG_12931			27200	-3.684	-0.5397	Yes
47	PITG_19178	PITG_19178			27218	-3.719	-0.5207	Yes
48	PITG_18717	PITG_18717			27230	-3.750	-0.5014	Yes
49	PITG_16792	PITG_16792			27244	-3.781	-0.4819	Yes
50	PITG_13300	PITG_13300			27271	-3.829	-0.4627	Yes
51	PITG_10819	PITG_10819			27276	-3.851	-0.4425	Yes
52	PITG_09374	PITG_09374			27287	-3.872	-0.4225	Yes
53	PITG_09222	PITG_09222			27296	-3.891	-0.4023	Yes
54	PITG_00052	PITG_00052			27309	-3.915	-0.3821	Yes
55	PITG_02536	PITG_02536			27313	-3.922	-0.3615	Yes
56	PITG_15652	PITG_15652			27336	-3.964	-0.3414	Yes
57	PITG_19608	PITG_19608			27376	-4.051	-0.3215	Yes
58	PITG_13380	PITG_13380			27381	-4.062	-0.3002	Yes
59	PITG_00142	PITG_00142			27390	-4.085	-0.2790	Yes
60	PITG_02593	PITG_02593			27396	-4.103	-0.2576	Yes
61	PITG_08338	PITG_08338			27459	-4.341	-0.2369	Yes
62	PITG_03955	PITG_03955			27474	-4.396	-0.2143	Yes
63	PITG_06667	PITG_06667			27485	-4.443	-0.1912	Yes
64	PITG_05534	PITG_05534			27486	-4.444	-0.1678	Yes
65	PITG_00597	PITG_00597			27494	-4.489	-0.1444	Yes
66	PITG_02887	PITG_02887			27502	-4.519	-0.1209	Yes
67	PITG_07308	PITG_07308			27517	-4.578	-0.0972	Yes
68	PITG_09432	PITG_09432			27524	-4.636	-0.0730	Yes
69	PITG_20593	PITG_20593			27525	-4.639	-0.0486	Yes
70	PITG_11198	PITG_11198			27529	-4.674	-0.0241	Yes
71	PITG_06263	PITG_06263			27552	-4.778	0.0003	Yes

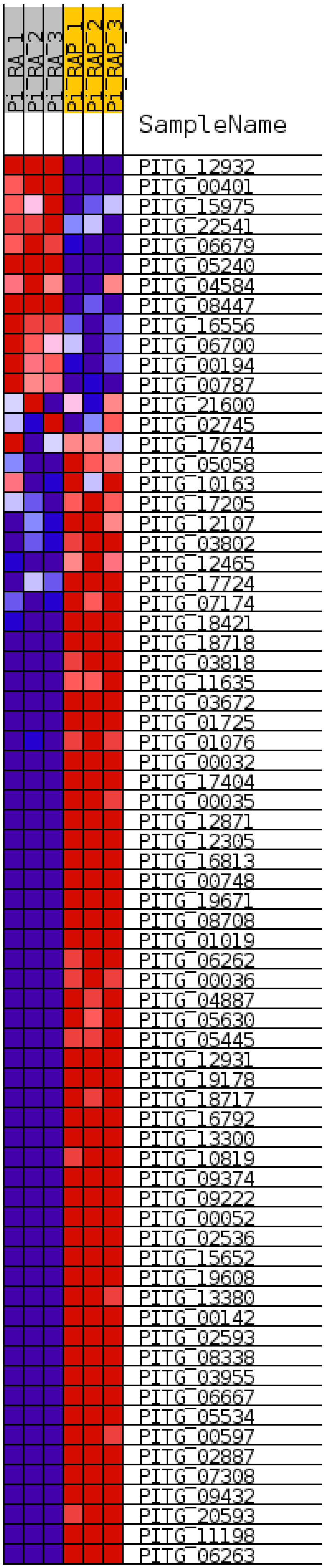
Pi_RA_1
Pi_RA_2
Pi_RA_3
Pi_RAP_1
Pi_RAP_2
Pi_RAP_3
SampleName
PITG_12932
PITG_00401
PITG_15975
PITG_22541
PITG_06679
PITG_05240
PITG_04584
PITG_08447
PITG_16556
PITG_06700
PITG_00194
PITG_00787
PITG_21600
PITG_02745
PITG_17674
PITG_05058
PITG_10163
PITG_17205
PITG_12107
PITG_03802
PITG_12465
PITG_17724
PITG_07174
PITG_18421
PITG_18718
PITG_03818
PITG_11635
PITG_03672
PITG_01725
PITG_01076
PITG_00032
PITG_17404
PITG_00035
PITG_12871
PITG_12305
PITG_16813
PITG_00748
PITG_19671
PITG_08708
PITG_01019
PITG_06262
PITG_00036
PITG_04887
PITG_05630
PITG_05445
PITG_12931
PITG_19178
PITG_18717
PITG_16792
PITG_13300
PITG_10819
PITG_09374
PITG_09222
PITG_00052
PITG_02536
PITG_15652
PITG_19608
PITG_13380
PITG_00142
PITG_02593
PITG_08338
PITG_03955
PITG_06667
PITG_05534
PITG_00597
PITG_02887
PITG_07308
PITG_09432
PITG_20593
PITG_11198
PITG_06263

Fig 2: RIBOSOME_BIOGENESIS_IN_EUKARYOTES(PIF03008)
Blue-Pink O' Gram in the Space of the Analyzed GeneSet

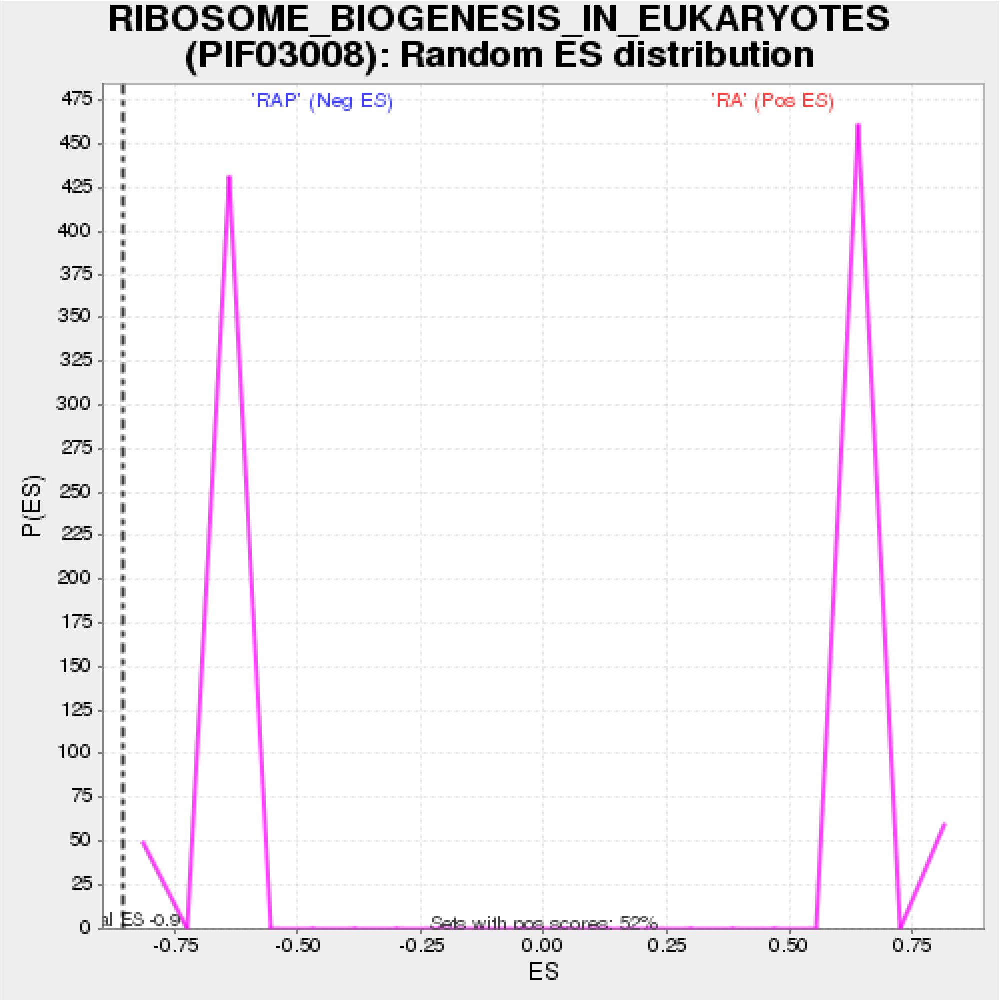

Fig 3: RIBOSOME_BIOGENESIS_IN_EUKARYOTES(PIF03008): Random ES distribution
Gene set null distribution of ES for RIBOSOME_BIOGENESIS_IN_EUKARYOTES(PIF03008)

9. RNA polymerase

Table: GSEA Results Summary

Dataset	fpkm.sample
Phenotype	sample.cls
Upregulated in class	RAP
GeneSet	RNA_POLYMERASE(PIF03020)
Enrichment Score (ES)	-0.7920969
Normalized Enrichment Score (NES)	-1.2601836
Nominal p-value	0.0
FDR q-value	0.10208338
FWER p-Value	0.0

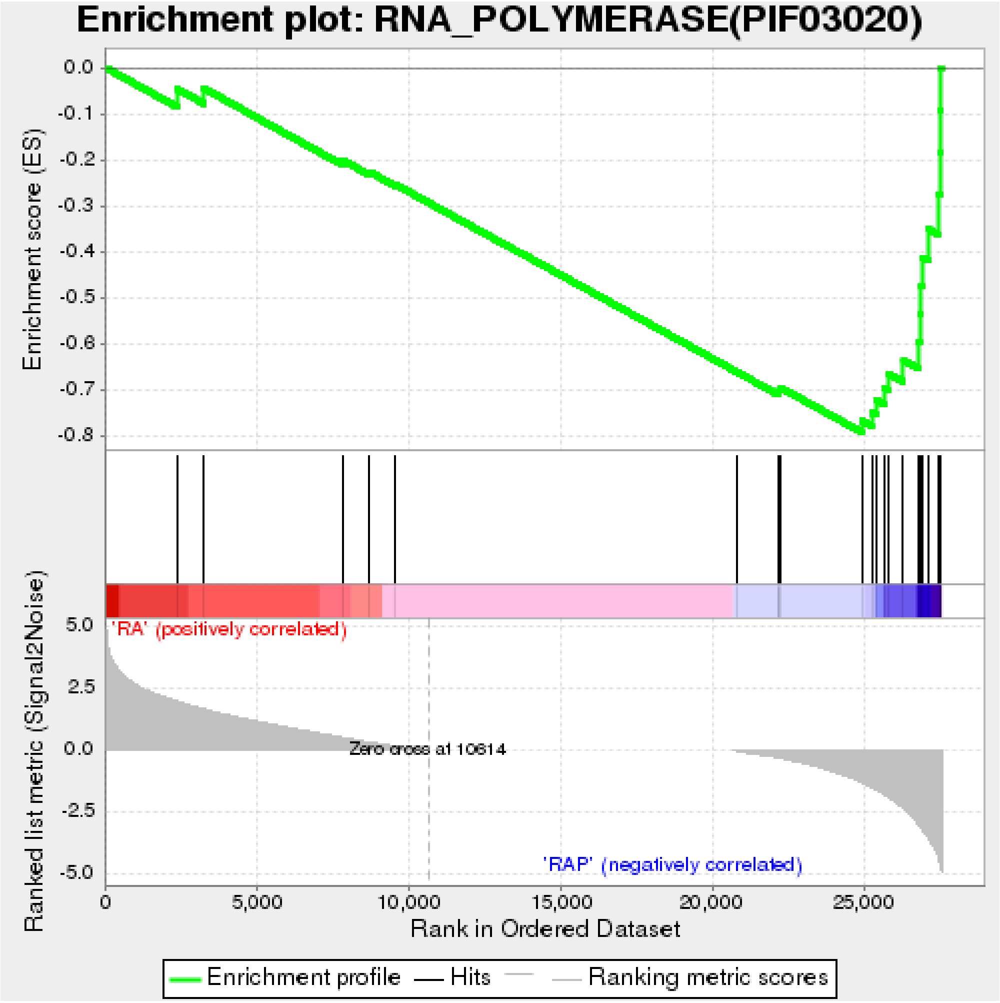

Fig 1: Enrichment plot: RNA_POLYMERASE(PIF03020)
Profile of the Running ES Score & Positions of GeneSet Members on the Rank Ordered List

Table: GSEA details [plain text format]

	PROBE	DESCRIPTION	GENE	GENE_TITLE	RANK IN	RANK	RUNNING	CORE

		(from dataset)	SYMBOL		GENE LIST	METRIC SCORE	ES	ENRICHMENT
1	Novel00232	Novel00232			2338	1.988	-0.0454	No
2	PITG_03793	PITG_03793			3220	1.671	-0.0442	No
3	PITG_21077	PITG_21077			7829	0.528	-0.2010	No
4	PITG_14885	PITG_14885			8683	0.340	-0.2252	No
5	PITG_05854	PITG_05854			9555	0.186	-0.2531	No
6	PITG_03795	PITG_03795			20816	-0.029	-0.6615	No
7	PITG_18727	PITG_18727			22194	-0.300	-0.7055	No
8	PITG_04645	PITG_04645			22213	-0.304	-0.7001	No
9	PITG_13404	PITG_13404			22268	-0.320	-0.6957	No
10	PITG_18113	PITG_18113			24924	-1.291	-0.7664	Yes
11	PITG_10445	PITG_10445			25254	-1.493	-0.7487	Yes
12	PITG_09425	PITG_09425			25390	-1.577	-0.7223	Yes
13	PITG_08383	PITG_08383			25672	-1.775	-0.6972	Yes
14	PITG_11365	PITG_11365			25807	-1.877	-0.6647	Yes
15	PITG_00051	PITG_00051			26301	-2.331	-0.6363	Yes
16	PITG_16116	PITG_16116			26810	-3.006	-0.5950	Yes
17	PITG_12877	PITG_12877			26837	-3.047	-0.5354	Yes
18	PITG_06706	PITG_06706			26889	-3.144	-0.4747	Yes
19	PITG_16526	PITG_16526			26926	-3.197	-0.4125	Yes
20	PITG_09712	PITG_09712			27123	-3.533	-0.3494	Yes
21	PITG_14613	PITG_14613			27480	-4.421	-0.2744	Yes
22	PITG_16658	PITG_16658			27516	-4.574	-0.1848	Yes
23	PITG_16659	PITG_16659			27527	-4.647	-0.0927	Yes
24	PITG_18777	PITG_18777			27539	-4.726	0.0008	Yes

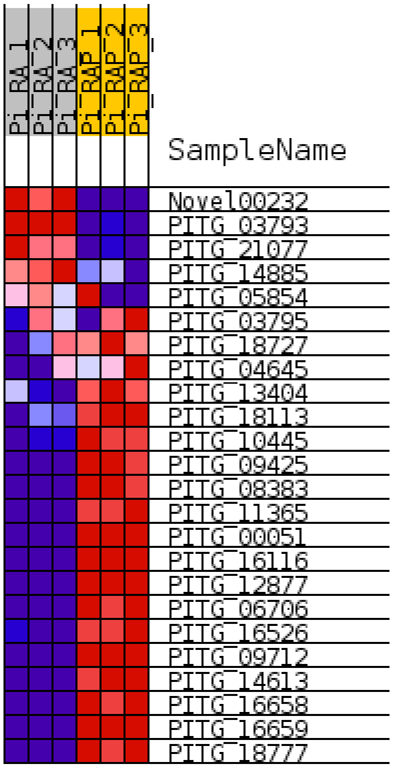

Fig 2: RNA_POLYMERASE(PIF03020)
Blue-Pink O' Gram in the Space of the Analyzed GeneSet

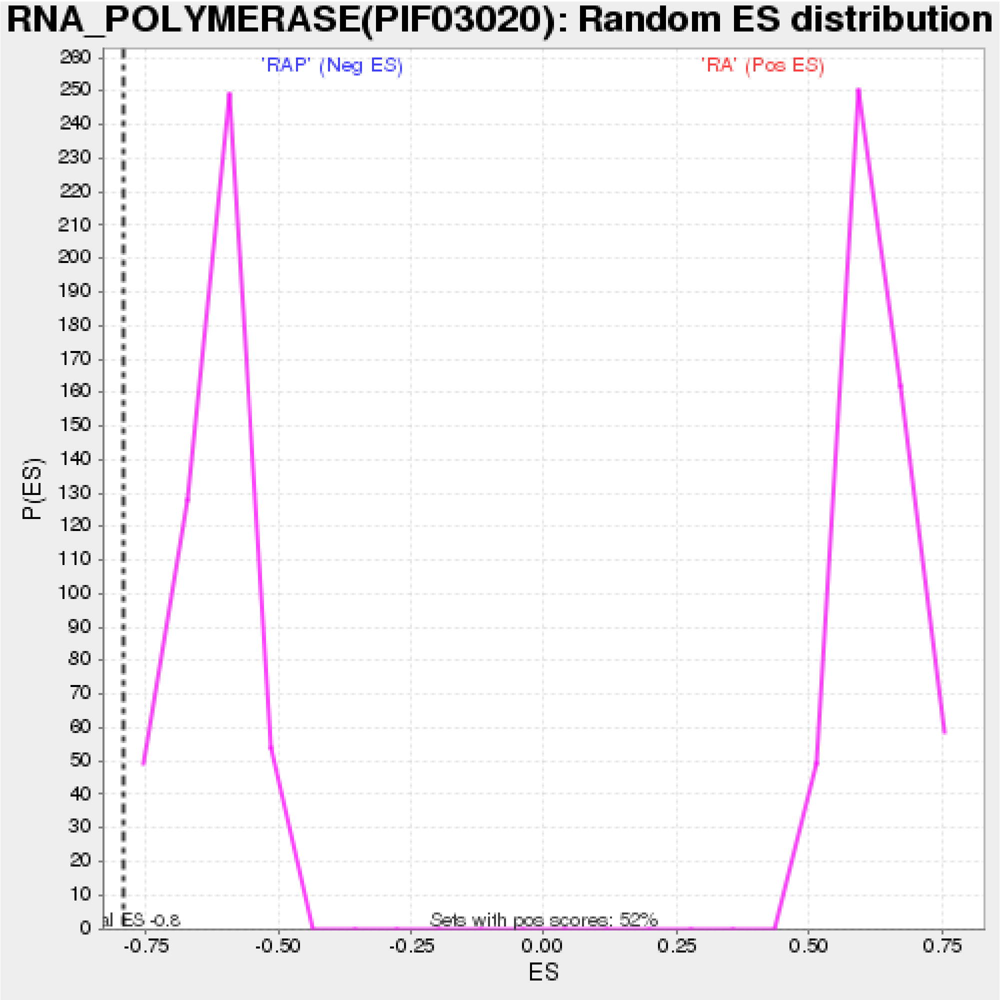

Fig 3: RNA_POLYMERASE(PIF03020): Random ES distribution
Gene set null distribution of ES for RNA_POLYMERASE(PIF03020)
