# Supplementary material for: TOR Inhibitors Synergistically Suppress the Growth and Development of Phytophthora infestans, a Highly Destructive Pathogenic Oomycete
Source: Front Microbiol. 2021 Apr 16;12:596874. doi: 10.3389/fmicb.2021.596874 (PMC8086431; doi:10.3389/fmicb.2021.596874)
Supplement: Supplementary Data Sheets 3, 4 — The detailed GSEA analyses of KEGG pathways (RAP + AZD vs. RAP, RAP + AZD vs. AZD). 1. Biosynthesis of amino acids; 2. Citrate cycle (TCA cycle); 3. DNA replication; 4. Oxidative phosphorylation; 5. Pentose phosphate pathway; 6. Protein processing in endoplasmic reticulum; 7. Ribosome; 8. Ribosome biogenesis in eukaryotes; 9. RNA polymerase. [file Data_Sheet_4.PDF]

1. Biosynthesis of amino acids

Table: GSEA Results Summary

Dataset	fpkm.sample
Phenotype	sample.cls
Upregulated in class	AZD
GeneSet	BIOSYNTHESIS_OF_AMINO_ACIDS(PIF01230)
Enrichment Score (ES)	-0.5094561
Normalized Enrichment Score (NES)	-1.1463071
Nominal p-value	0.11016949
FDR q-value	0.14067788
FWER p-Value	0.136

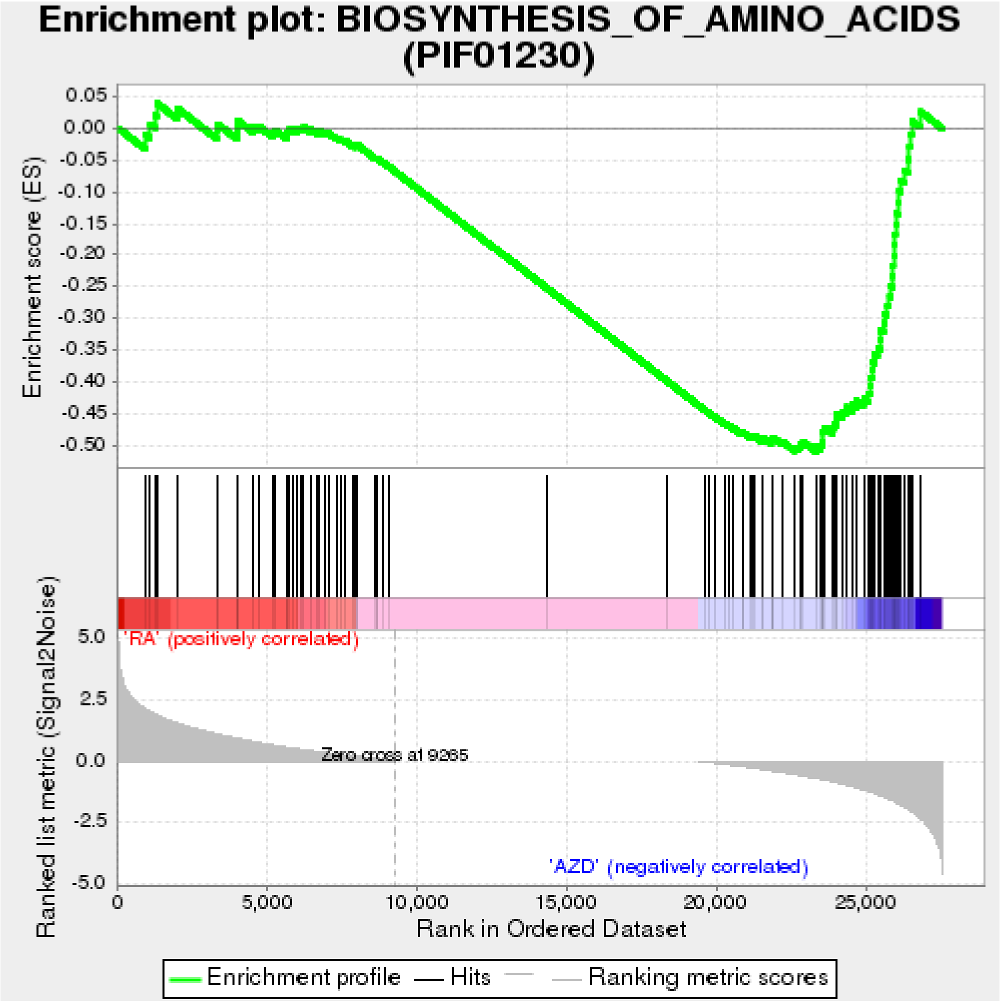

Fig 1: Enrichment plot: BIOSYNTHESIS_OF_AMINO_ACIDS(PIF01230)
Profile of the Running ES Score & Positions of GeneSet Members on the Rank Ordered List

Table: GSEA details [plain text format]

	PROBE	DESCRIPTION	GENE	GENE_TITLE	RANK IN	RANK	RUNNING	CORE

		(from dataset)	SYMBOL		GENE LIST	METRIC SCORE	ES	ENRICHMENT
1	PITG_14634	PITG_14634			891	2.148	-0.0092	No
2	PITG_09400	PITG_09400			1065	2.019	0.0064	No
3	PITG_12513	PITG_12513			1215	1.924	0.0218	No
4	PITG_07283	PITG_07283			1278	1.882	0.0399	No
5	PITG_00029	PITG_00029			1950	1.547	0.0322	No
6	PITG_02049	PITG_02049			3284	1.111	-0.0043	No
7	PITG_02852	PITG_02852			3297	1.108	0.0073	No
8	PITG_15449	PITG_15449			3956	0.931	-0.0066	No
9	PITG_08444	PITG_08444			3979	0.925	0.0026	No
10	PITG_22764	PITG_22764			3996	0.921	0.0120	No
11	PITG_04466	PITG_04466			4515	0.798	0.0018	No
12	PITG_05861	PITG_05861			4695	0.758	0.0035	No
13	PITG_01188	PITG_01188			5156	0.661	-0.0061	No
14	PITG_07380	PITG_07380			5259	0.637	-0.0030	No
15	PITG_12725	PITG_12725			5631	0.567	-0.0103	No
16	PITG_02757	PITG_02757			5656	0.562	-0.0051	No
17	PITG_01195	PITG_01195			5728	0.549	-0.0018	No
18	PITG_14179	PITG_14179			5849	0.526	-0.0004	No
19	PITG_13262	PITG_13262			5997	0.501	-0.0004	No
20	PITG_00028	PITG_00028			6109	0.479	0.0008	No
21	PITG_03900	PITG_03900			6212	0.462	0.0021	No
22	PITG_23158	PITG_23158			6439	0.425	-0.0016	No
23	PITG_07056	PITG_07056			6640	0.390	-0.0046	No
24	PITG_17516	PITG_17516			6710	0.378	-0.0030	No
25	PITG_22103	PITG_22103			6927	0.339	-0.0072	No
26	PITG_12462	PITG_12462			7022	0.323	-0.0072	No
27	PITG_17032	PITG_17032			7310	0.279	-0.0146	No
28	PITG_10595	PITG_10595			7468	0.254	-0.0176	No
29	PITG_13139	PITG_13139			7597	0.236	-0.0197	No
30	PITG_10301	PITG_10301			7849	0.199	-0.0266	No
31	PITG_02198	PITG_02198			7951	0.187	-0.0283	No
32	PITG_09402	PITG_09402			7984	0.183	-0.0275	No
33	PITG_03098	PITG_03098			7985	0.183	-0.0255	No
34	PITG_02785	PITG_02785			8592	0.101	-0.0465	No
35	PITG_04665	PITG_04665			8634	0.096	-0.0469	No
36	PITG_06265	PITG_06265			8650	0.093	-0.0465	No
37	PITG_03700	PITG_03700			8871	0.057	-0.0539	No
38	PITG_09394	PITG_09394			9068	0.027	-0.0607	No

39	PITG_16047	PITG_16047			14349	0.000	-0.2530	No
40	PITG_05858	PITG_05858			18330	0.000	-0.3980	No
41	PITG_19493	PITG_19493			19636	-0.031	-0.4452	No
42	PITG_02210	PITG_02210			19755	-0.049	-0.4489	No
43	PITG_03593	PITG_03593			19979	-0.086	-0.4561	No
44	PITG_08022	PITG_08022			20275	-0.127	-0.4655	No
45	PITG_16048	PITG_16048			20414	-0.150	-0.4689	No
46	PITG_09393	PITG_09393			20582	-0.174	-0.4731	No
47	PITG_03599	PITG_03599			20862	-0.215	-0.4809	No
48	PITG_03101	PITG_03101			20872	-0.216	-0.4789	No
49	PITG_00166	PITG_00166			21147	-0.265	-0.4860	No
50	PITG_06518	PITG_06518			21226	-0.277	-0.4859	No
51	PITG_16057	PITG_16057			21307	-0.293	-0.4856	No
52	PITG_01752	PITG_01752			21535	-0.332	-0.4903	No
53	PITG_18048	PITG_18048			21538	-0.332	-0.4867	No
54	PITG_05245	PITG_05245			21857	-0.392	-0.4941	No
55	PITG_05318	PITG_05318			21858	-0.392	-0.4898	No
56	PITG_22069	PITG_22069			21898	-0.399	-0.4869	No
57	PITG_14180	PITG_14180			22213	-0.452	-0.4935	No
58	PITG_02735	PITG_02735			22634	-0.542	-0.5029	Yes
59	PITG_19961	PITG_19961			22791	-0.576	-0.5023	Yes
60	PITG_02925	PITG_02925			22842	-0.586	-0.4978	Yes
61	PITG_01711	PITG_01711			22919	-0.600	-0.4941	Yes
62	PITG_01804	PITG_01804			23342	-0.693	-0.5020	Yes
63	PITG_13402	PITG_13402			23489	-0.727	-0.4994	Yes
64	PITG_01564	PITG_01564			23544	-0.740	-0.4934	Yes
65	PITG_04851	PITG_04851			23573	-0.748	-0.4863	Yes
66	PITG_14697	PITG_14697			23574	-0.748	-0.4782	Yes
67	PITG_09698	PITG_09698			23607	-0.757	-0.4711	Yes
68	PITG_05374	PITG_05374			23918	-0.844	-0.4733	Yes
69	PITG_21397	PITG_21397			23938	-0.851	-0.4648	Yes
70	PITG_02740	PITG_02740			24032	-0.880	-0.4586	Yes
71	PITG_17925	PITG_17925			24047	-0.884	-0.4496	Yes
72	PITG_06448	PITG_06448			24253	-0.946	-0.4468	Yes
73	PITG_17786	PITG_17786			24336	-0.971	-0.4393	Yes
74	PITG_13116	PITG_13116			24537	-1.033	-0.4353	Yes
75	PITG_04698	PITG_04698			24698	-1.082	-0.4295	Yes
76	PITG_09817	PITG_09817			24933	-1.163	-0.4254	Yes
77	PITG_03620	PITG_03620			25088	-1.222	-0.4178	Yes

78	PITG_06427	PITG_06427			25146	-1.244	-0.4064	Yes
79	PITG_20970	PITG_20970			25173	-1.251	-0.3938	Yes
80	PITG_20687	PITG_20687			25210	-1.266	-0.3814	Yes
81	PITG_03698	PITG_03698			25237	-1.274	-0.3685	Yes
82	PITG_01768	PITG_01768			25291	-1.299	-0.3564	Yes
83	PITG_13749	PITG_13749			25401	-1.354	-0.3457	Yes
84	PITG_02256	PITG_02256			25486	-1.391	-0.3337	Yes
85	PITG_09582	PITG_09582			25513	-1.402	-0.3194	Yes
86	PITG_07400	PITG_07400			25604	-1.452	-0.3070	Yes
87	PITG_14195	PITG_14195			25624	-1.463	-0.2918	Yes
88	PITG_07405	PITG_07405			25728	-1.511	-0.2792	Yes
89	PITG_19096	PITG_19096			25797	-1.546	-0.2650	Yes
90	PITG_05636	PITG_05636			25845	-1.568	-0.2497	Yes
91	PITG_01769	PITG_01769			25892	-1.589	-0.2342	Yes
92	PITG_12161	PITG_12161			25912	-1.599	-0.2175	Yes
93	PITG_22685	PITG_22685			25933	-1.616	-0.2007	Yes
94	PITG_12053	PITG_12053			25941	-1.621	-0.1834	Yes
95	PITG_06267	PITG_06267			25950	-1.627	-0.1661	Yes
96	PITG_14696	PITG_14696			26002	-1.656	-0.1500	Yes
97	PITG_12727	PITG_12727			26036	-1.675	-0.1331	Yes
98	PITG_01245	PITG_01245			26115	-1.733	-0.1172	Yes
99	PITG_20759	PITG_20759			26127	-1.742	-0.0987	Yes
100	PITG_05551	PITG_05551			26168	-1.770	-0.0810	Yes
101	PITG_03078	PITG_03078			26299	-1.849	-0.0657	Yes
102	PITG_00132	PITG_00132			26411	-1.939	-0.0488	Yes
103	PITG_03598	PITG_03598			26463	-1.987	-0.0291	Yes
104	PITG_13399	PITG_13399			26492	-2.007	-0.0084	Yes
105	PITG_03992	PITG_03992			26542	-2.040	0.0119	Yes
106	PITG_01939	PITG_01939			26833	-2.322	0.0265	Yes

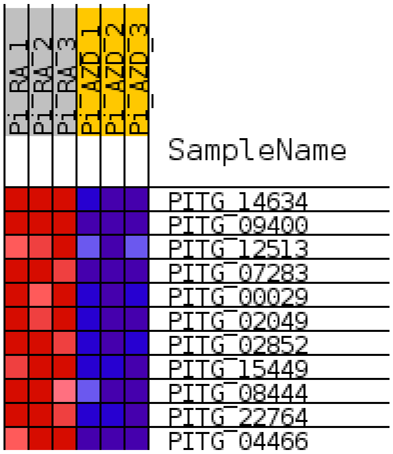

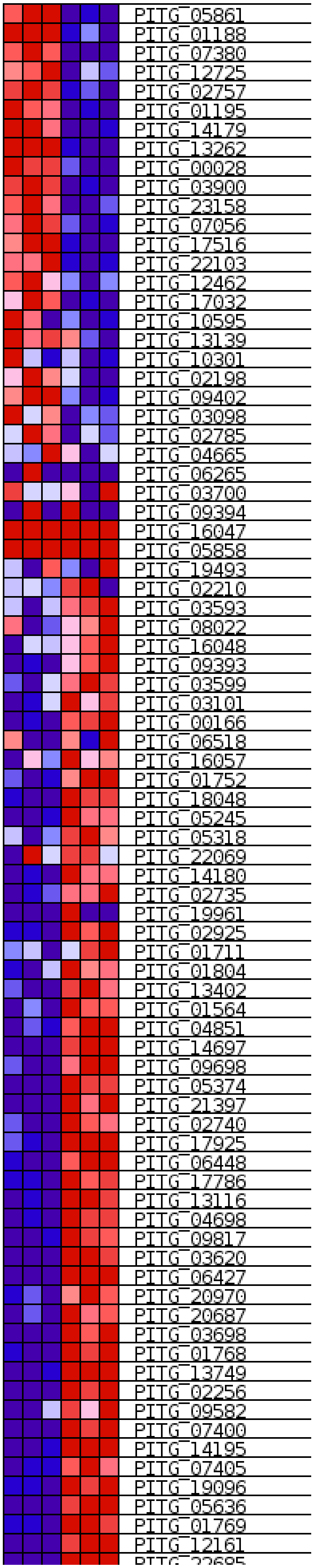
PITG_05861
PITG_01188
PITG_07380
PITG_12725
PITG_02757
PITG_01195
PITG_14179
PITG_13262
PITG_00028
PITG_03900
PITG_23158
PITG_07056
PITG_17516
PITG_22103
PITG_12462
PITG_17032
PITG_10595
PITG_13139
PITG_10301
PITG_02198
PITG_09402
PITG_03098
PITG_02785
PITG_04665
PITG_06265
PITG_03700
PITG_09394
PITG_16047
PITG_05858
PITG_19493
PITG_02210
PITG_03593
PITG_08022
PITG_16048
PITG_09393
PITG_03599
PITG_03101
PITG_00166
PITG_06518
PITG_16057
PITG_01752
PITG_18048
PITG_05245
PITG_05318
PITG_22069
PITG_14180
PITG_02735
PITG_19961
PITG_02925
PITG_01711
PITG_01804
PITG_13402
PITG_01564
PITG_04851
PITG_14697
PITG_09698
PITG_05374
PITG_21397
PITG_02740
PITG_17925
PITG_06448
PITG_17786
PITG_13116
PITG_04698
PITG_09817
PITG_03620
PITG_06427
PITG_20970
PITG_20687
PITG_03698
PITG_01768
PITG_13749
PITG_02256
PITG_09582
PITG_07400
PITG_14195
PITG_07405
PITG_19096
PITG_05636
PITG_01769
PITG_12161

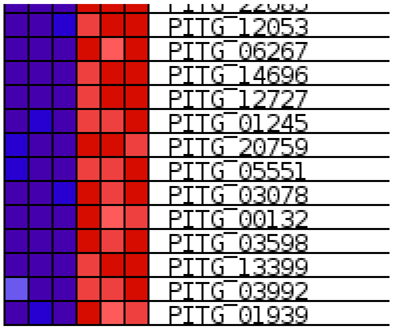

Fig 2: BIOSYNTHESIS_OF_AMINO_ACIDS(PIF01230)
Blue-Pink O' Gram in the Space of the Analyzed GeneSet

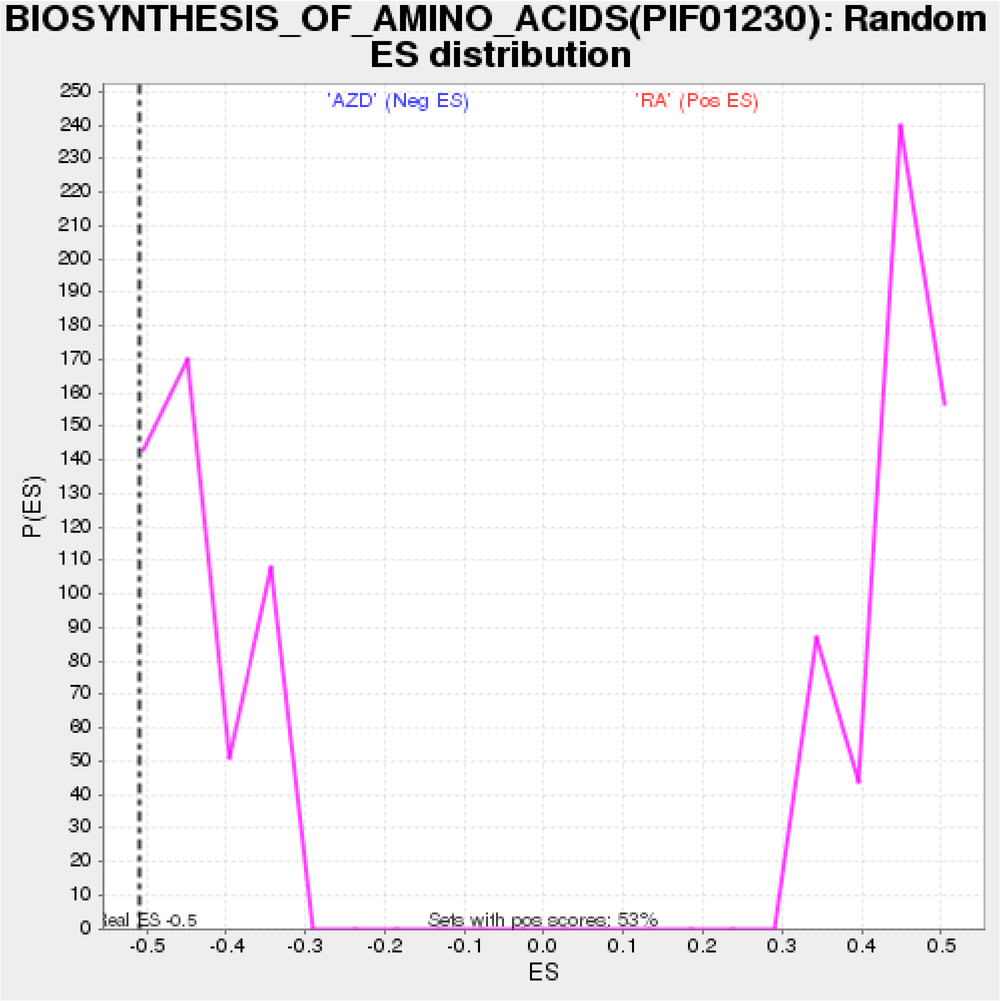

Fig 3: BIOSYNTHESIS_OF_AMINO_ACIDS(PIF01230): Random ES distribution
Gene set null distribution of ES for BIOSYNTHESIS_OF_AMINO_ACIDS(PIF01230)

2. Citrate cycle (TCA cycle)

Table: GSEA Results Summary

Dataset	fpkm.sample
Phenotype	sample.cls
Upregulated in class	AZD
GeneSet	CITRATE_CYCLE_(TCA_CYCLE)(PIF00020)
Enrichment Score (ES)	-0.7002187
Normalized Enrichment Score (NES)	-1.0963011
Nominal p-value	0.0
FDR q-value	0.1634003
FWER p-Value	0.179

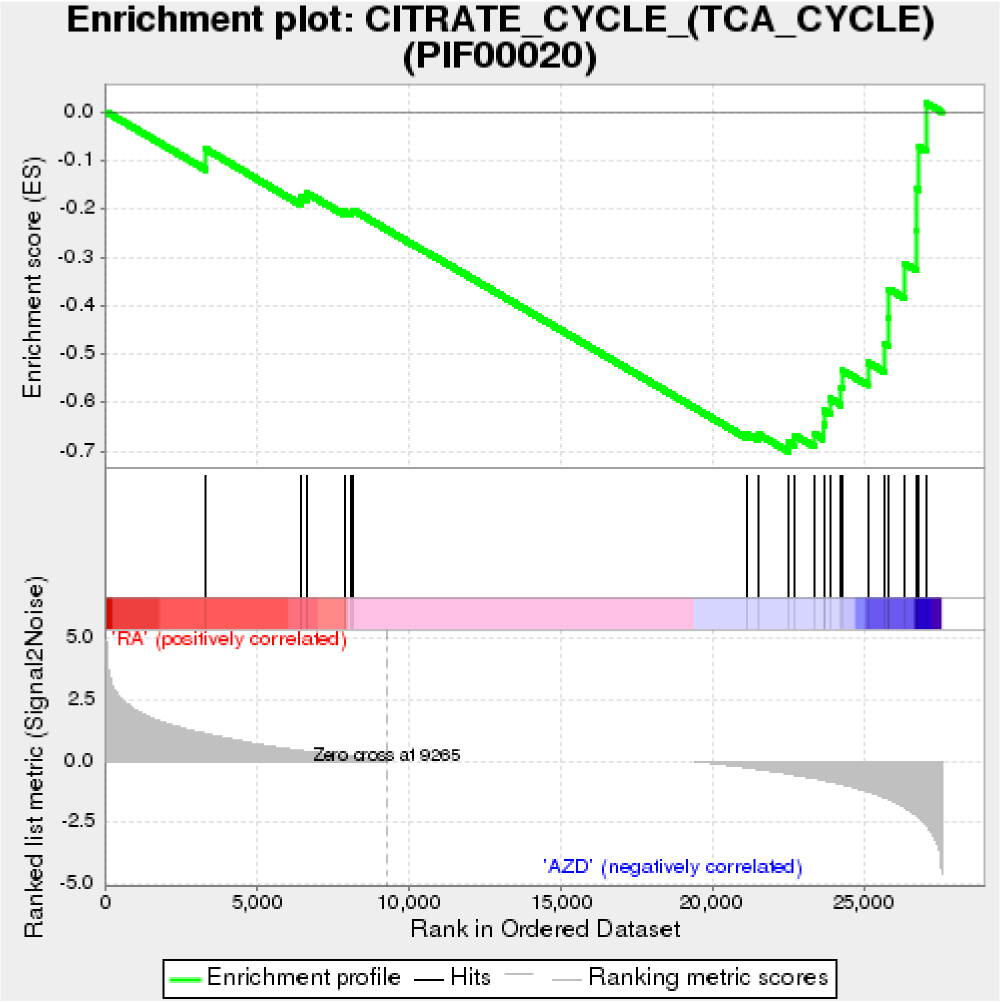

Fig 1: Enrichment plot: CITRATE_CYCLE_(TCA_CYCLE)(PIF00020)
Profile of the Running ES Score & Positions of GeneSet Members on the Rank Ordered List

Table: GSEA details [plain text format]

	PROBE	DESCRIPTION	GENE	GENE_TITLE	RANK IN	RANK	RUNNING	CORE

		(from dataset)	SYMBOL		GENE LIST	METRIC SCORE	ES	ENRICHMENT
1	PITG_02049	PITG_02049			3284	1.111	-0.0768	No
2	PITG_23158	PITG_23158			6439	0.425	-0.1751	No
3	PITG_07056	PITG_07056			6640	0.390	-0.1675	No
4	PITG_08880	PITG_08880			7853	0.199	-0.2039	No
5	PITG_19191	PITG_19191			8108	0.170	-0.2066	No
6	PITG_15476	PITG_15476			8136	0.167	-0.2012	No
7	PITG_16966	PITG_16966			21136	-0.263	-0.6632	No
8	PITG_18048	PITG_18048			21538	-0.332	-0.6651	No
9	PITG_12905	PITG_12905			22506	-0.511	-0.6807	Yes
10	PITG_22743	PITG_22743			22733	-0.563	-0.6674	Yes
11	PITG_10951	PITG_10951			23348	-0.694	-0.6632	Yes
12	PITG_06108	PITG_06108			23666	-0.772	-0.6452	Yes
13	PITG_03277	PITG_03277			23686	-0.778	-0.6162	Yes
14	PITG_11452	PITG_11452			23883	-0.832	-0.5915	Yes
15	PITG_06448	PITG_06448			24253	-0.946	-0.5687	Yes
16	PITG_19161	PITG_19161			24315	-0.963	-0.5342	Yes
17	PITG_15705	PITG_15705			25120	-1.234	-0.5162	Yes
18	PITG_18720	PITG_18720			25690	-1.492	-0.4799	Yes
19	PITG_11929	PITG_11929			25800	-1.548	-0.4247	Yes
20	PITG_13614	PITG_13614			25825	-1.559	-0.3660	Yes
21	PITG_15359	PITG_15359			26322	-1.864	-0.3128	Yes
22	PITG_06604	PITG_06604			26738	-2.215	-0.2433	Yes
23	PITG_18354	PITG_18354			26745	-2.222	-0.1586	Yes
24	PITG_18935	PITG_18935			26825	-2.316	-0.0730	Yes
25	PITG_02026	PITG_02026			27058	-2.609	0.0183	Yes

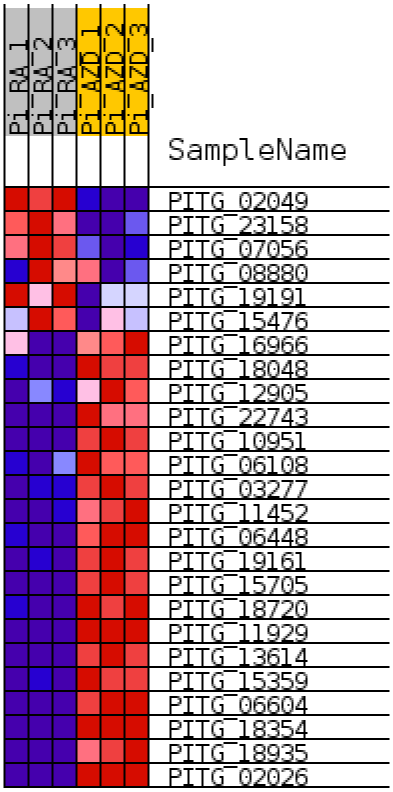

Fig 2: CITRATE_CYCLE_(TCA_CYCLE)(PIF00020)
Blue-Pink O' Gram in the Space of the Analyzed GeneSet

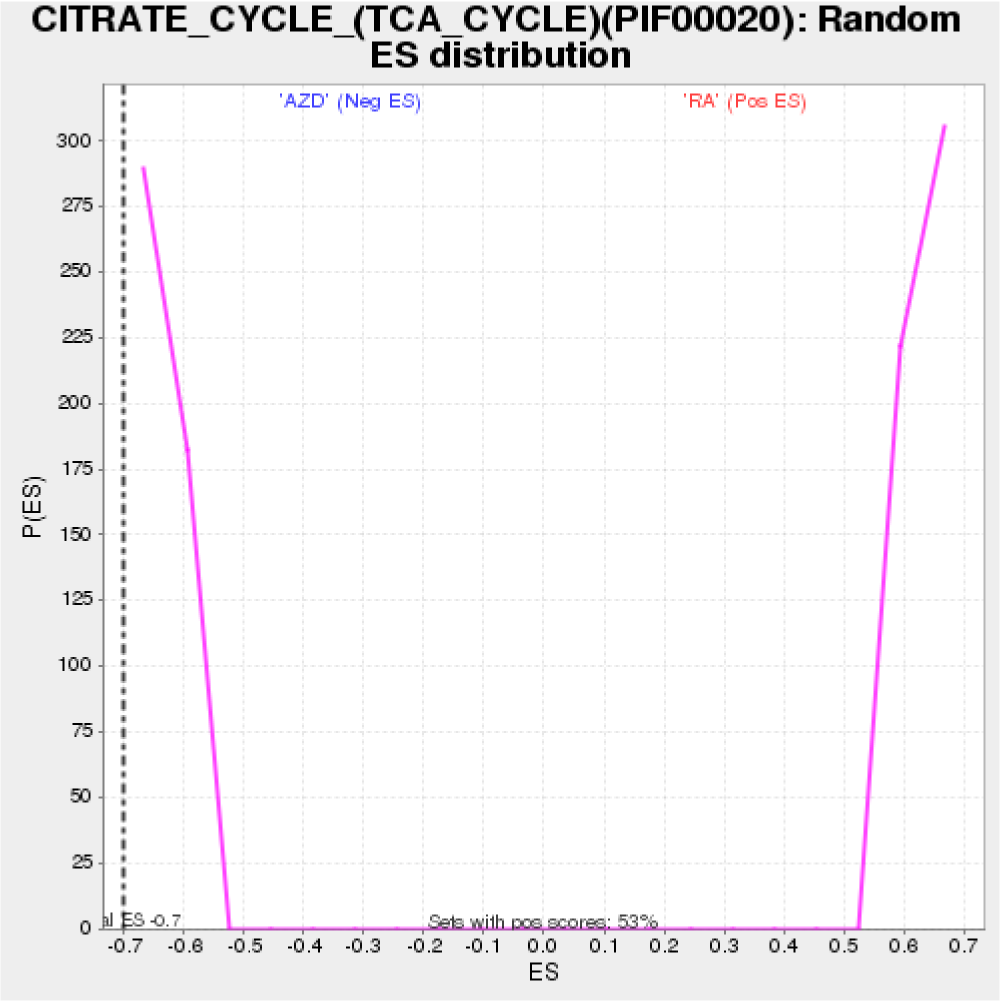

Fig 3: CITRATE_CYCLE_(TCA_CYCLE)(PIF00020): Random ES distribution
Gene set null distribution of ES for CITRATE_CYCLE_(TCA_CYCLE)(PIF00020)

3. DNA replication

Table: GSEA Results Summary

Dataset	fpkm.sample
Phenotype	sample.cls
Upregulated in class	AZD
GeneSet	DNA_REPLICATION(PIF03030)
Enrichment Score (ES)	-0.6260042
Normalized Enrichment Score (NES)	-1.1730305
Nominal p-value	0.0
FDR q-value	0.13135591
FWER p-Value	0.101

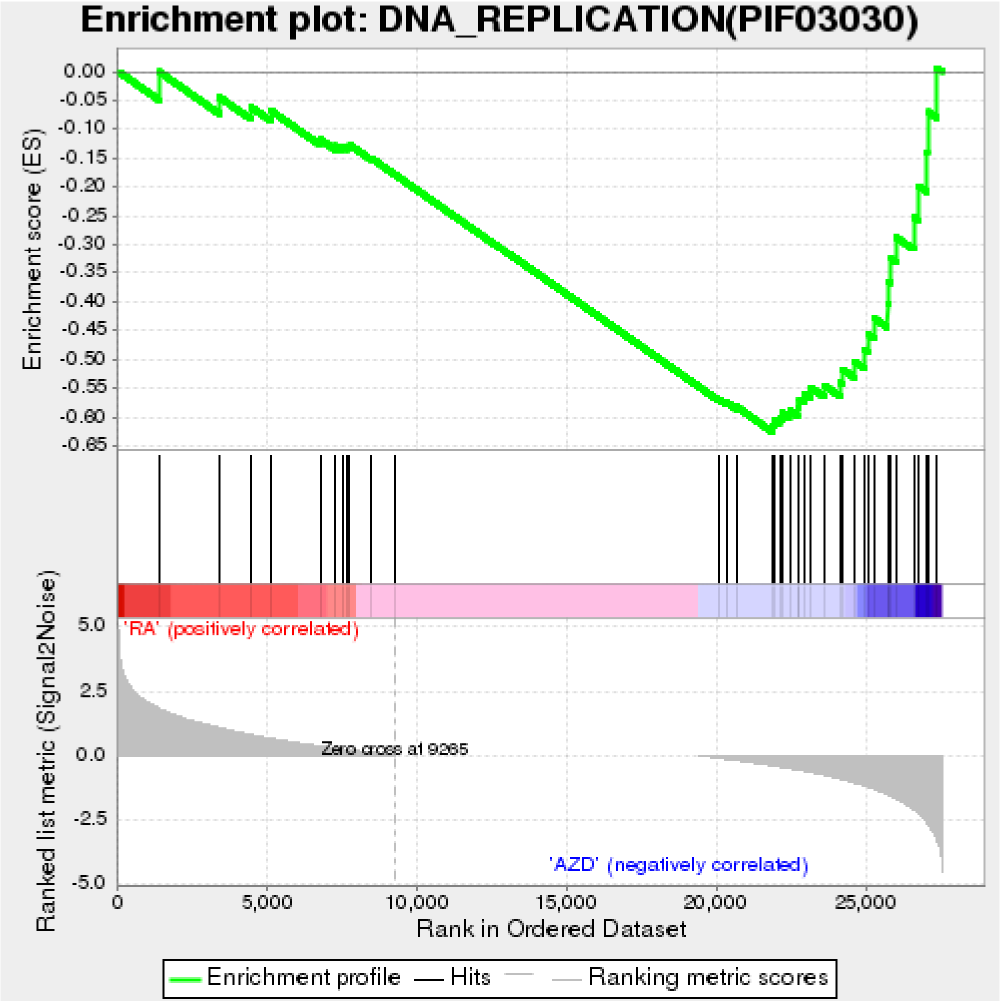

Fig 1: Enrichment plot: DNA_REPLICATION(PIF03030)
Profile of the Running ES Score & Positions of GeneSet Members on the Rank Ordered List

Table: GSEA details [plain text format]

	PROBE	DESCRIPTION	GENE	GENE_TITLE	RANK IN	RANK	RUNNING	CORE

		(from dataset)	SYMBOL		GENE LIST	METRIC SCORE	ES	ENRICHMENT
1	PITG_12746	PITG_12746			1348	1.838	0.0002	No
2	PITG_10249	PITG_10249			3375	1.083	-0.0444	No
3	PITG_13336	PITG_13336			4441	0.815	-0.0613	No
4	PITG_00557	PITG_00557			5093	0.672	-0.0670	No
5	PITG_04614	PITG_04614			6758	0.371	-0.1175	No
6	PITG_18696	PITG_18696			7262	0.287	-0.1281	No
7	PITG_05369	PITG_05369			7487	0.251	-0.1295	No
8	PITG_07644	PITG_07644			7648	0.229	-0.1292	No
9	PITG_10245	PITG_10245			7752	0.213	-0.1273	No
10	PITG_10254	PITG_10254			8452	0.118	-0.1495	No
11	PITG_19314	PITG_19314			9238	0.005	-0.1779	No
12	PITG_18237	PITG_18237			20079	-0.100	-0.5691	No
13	PITG_04709	PITG_04709			20351	-0.140	-0.5752	No
14	PITG_08606	PITG_08606			20666	-0.187	-0.5816	No
15	PITG_07175	PITG_07175			21890	-0.397	-0.6154	Yes
16	PITG_12152	PITG_12152			21942	-0.408	-0.6063	Yes
17	PITG_15505	PITG_15505			22125	-0.438	-0.6012	Yes
18	PITG_05585	PITG_05585			22222	-0.454	-0.5925	Yes
19	PITG_03056	PITG_03056			22459	-0.502	-0.5877	Yes
20	PITG_15868	PITG_15868			22730	-0.563	-0.5824	Yes
21	PITG_20527	PITG_20527			22785	-0.575	-0.5690	Yes
22	PITG_03389	PITG_03389			22961	-0.610	-0.5591	Yes
23	PITG_04710	PITG_04710			23179	-0.659	-0.5493	Yes
24	PITG_11812	PITG_11812			23615	-0.758	-0.5448	Yes
25	PITG_16698	PITG_16698			24166	-0.919	-0.5403	Yes
26	PITG_05302	PITG_05302			24230	-0.938	-0.5175	Yes
27	PITG_13792	PITG_13792			24607	-1.053	-0.5029	Yes
28	PITG_16691	PITG_16691			24971	-1.179	-0.4846	Yes
29	PITG_08326	PITG_08326			25071	-1.216	-0.4556	Yes
30	PITG_07546	PITG_07546			25312	-1.311	-0.4293	Yes
31	PITG_02054	PITG_02054			25732	-1.513	-0.4040	Yes
32	PITG_11912	PITG_11912			25761	-1.526	-0.3642	Yes
33	PITG_04527	PITG_04527			25858	-1.574	-0.3256	Yes
34	PITG_12346	PITG_12346			26010	-1.662	-0.2866	Yes
35	PITG_15053	PITG_15053			26608	-2.097	-0.2522	Yes
36	PITG_06111	PITG_06111			26757	-2.234	-0.1978	Yes
37	PITG_03624	PITG_03624			27055	-2.604	-0.1389	Yes
38	PITG_07399	PITG_07399			27071	-2.627	-0.0692	Yes

39	PITG_14397	PITG_14397			27378	-3.250	0.0066	Yes

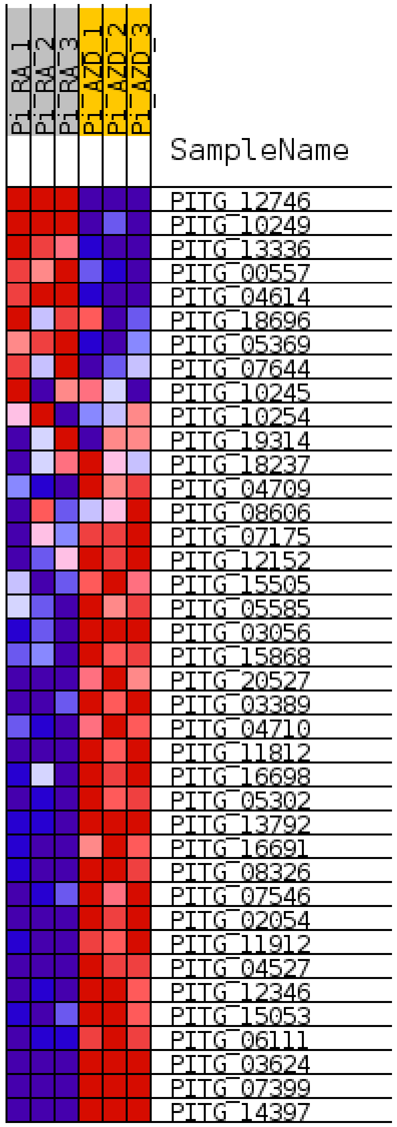

Fig 2: DNA_REPLICATION(PIF03030)
Blue-Pink O' Gram in the Space of the Analyzed GeneSet

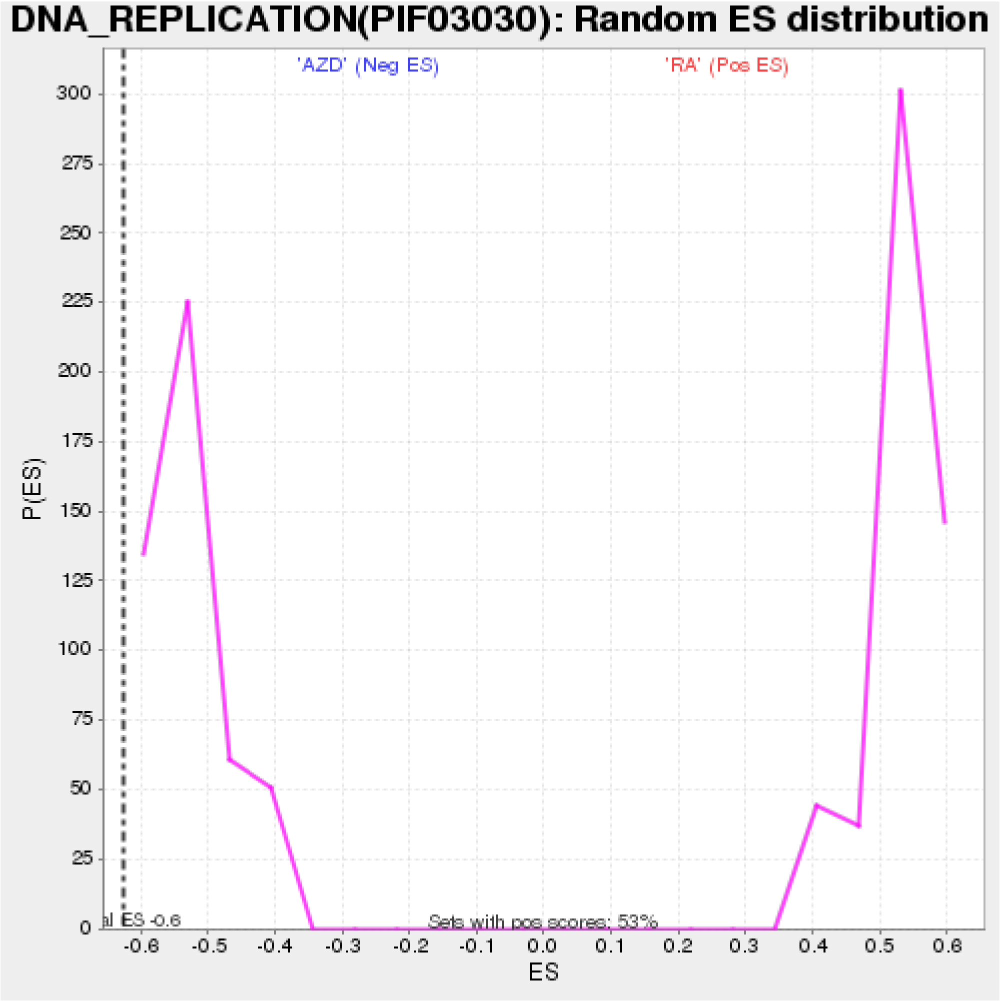

Fig 3: DNA_REPLICATION(PIF03030): Random ES distribution
Gene set null distribution of ES for DNA_REPLICATION(PIF03030)

4. Oxidative phosphorylation

Table: GSEA Results Summary

Dataset	fpkm.sample
Phenotype	sample.cls
Upregulated in class	AZD
GeneSet	OXIDATIVE_PHOSPHORYLATION(PIF00190)
Enrichment Score (ES)	-0.6449316
Normalized Enrichment Score (NES)	-1.0968301
Nominal p-value	0.11016949
FDR q-value	0.17191273
FWER p-Value	0.179

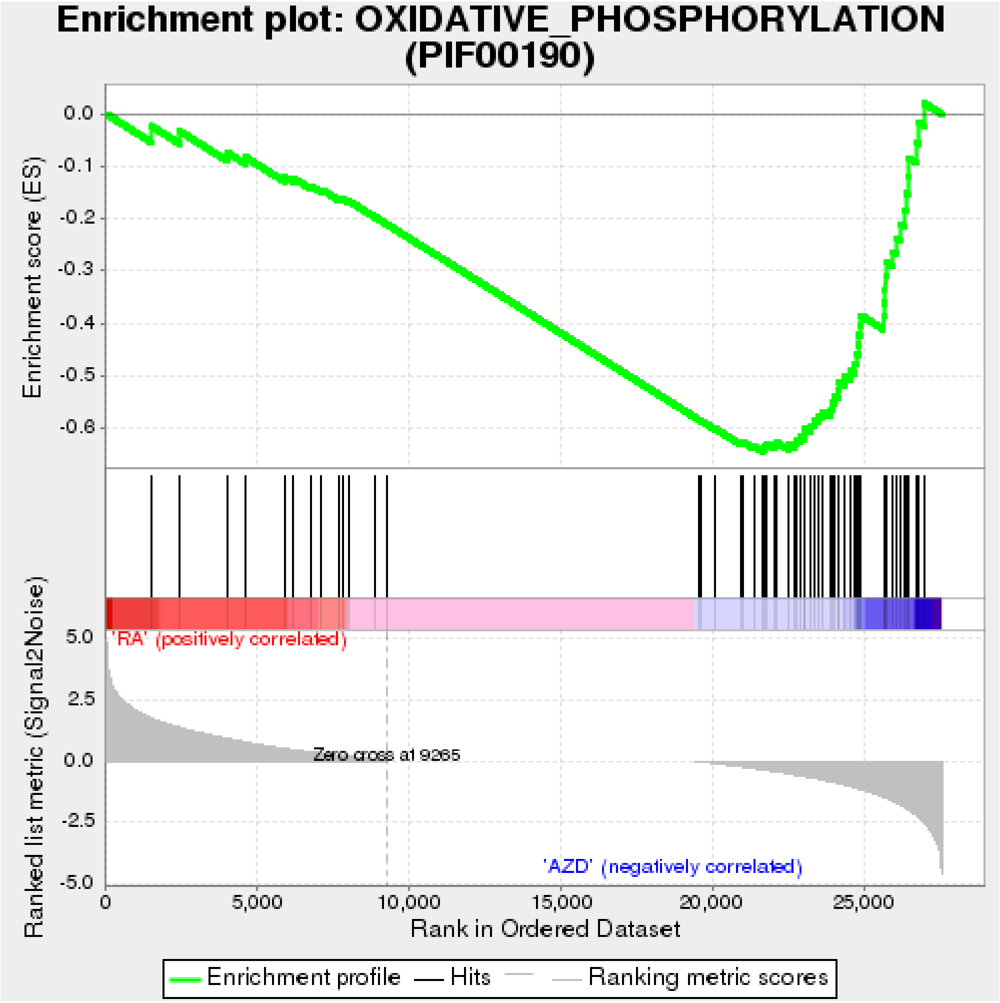

Fig 1: Enrichment plot: OXIDATIVE_PHOSPHORYLATION(PIF00190)
Profile of the Running ES Score & Positions of GeneSet Members on the Rank Ordered List

Table: GSEA details [plain text format]

	PROBE	DESCRIPTION	GENE	GENE_TITLE	RANK IN	RANK	RUNNING	CORE

		(from dataset)	SYMBOL		GENE LIST	METRIC SCORE	ES	ENRICHMENT
1	PITG_09016	PITG_09016			1456	1.775	-0.0218	No
2	PITG_16016	PITG_16016			2415	1.374	-0.0325	No
3	PITG_06814	PITG_06814			3997	0.921	-0.0738	No
4	Novel00931	Novel00931			4608	0.777	-0.0823	No
5	PITG_09032	PITG_09032			5887	0.518	-0.1197	No
6	PITG_00693	PITG_00693			6182	0.468	-0.1221	No
7	PITG_08094	PITG_08094			6773	0.368	-0.1371	No
8	PITG_17747	PITG_17747			7112	0.309	-0.1440	No
9	PITG_16670	PITG_16670			7658	0.228	-0.1598	No
10	PITG_13301	PITG_13301			7795	0.205	-0.1611	No
11	PITG_04452	PITG_04452			7983	0.183	-0.1647	No
12	PITG_06385	PITG_06385			8893	0.053	-0.1968	No
13	PITG_12289	PITG_12289			9254	0.002	-0.2099	No
14	PITG_01698	PITG_01698			19530	-0.018	-0.5832	No
15	PITG_06615	PITG_06615			19640	-0.031	-0.5866	No
16	PITG_07860	PITG_07860			20088	-0.101	-0.6011	No
17	PITG_19772	PITG_19772			20917	-0.225	-0.6272	No
18	PITG_00764	PITG_00764			20979	-0.236	-0.6253	No
19	PITG_09472	PITG_09472			21360	-0.303	-0.6338	No
20	PITG_09796	PITG_09796			21667	-0.354	-0.6387	Yes
21	PITG_17921	PITG_17921			21737	-0.367	-0.6348	Yes
22	PITG_12309	PITG_12309			21780	-0.377	-0.6297	Yes
23	PITG_13728	PITG_13728			22057	-0.426	-0.6322	Yes
24	PITG_09031	PITG_09031			22113	-0.436	-0.6266	Yes
25	PITG_12905	PITG_12905			22506	-0.511	-0.6318	Yes
26	PITG_12520	PITG_12520			22708	-0.560	-0.6293	Yes
27	PITG_09436	PITG_09436			22766	-0.568	-0.6214	Yes
28	PITG_09015	PITG_09015			22916	-0.599	-0.6163	Yes
29	PITG_15553	PITG_15553			23050	-0.632	-0.6100	Yes
30	PITG_03344	PITG_03344			23062	-0.634	-0.5993	Yes
31	PITG_02045	PITG_02045			23263	-0.676	-0.5947	Yes
32	PITG_10951	PITG_10951			23348	-0.694	-0.5855	Yes
33	PITG_14362	PITG_14362			23477	-0.724	-0.5775	Yes
34	PITG_12141	PITG_12141			23603	-0.756	-0.5687	Yes
35	PITG_11452	PITG_11452			23883	-0.832	-0.5643	Yes
36	PITG_05840	PITG_05840			23958	-0.856	-0.5519	Yes
37	PITG_09550	PITG_09550			24034	-0.880	-0.5392	Yes
38	PITG_03632	PITG_03632			24145	-0.913	-0.5271	Yes

39	PITG_09547	PITG_09547			24182	-0.924	-0.5122	Yes
40	PITG_10862	PITG_10862			24348	-0.975	-0.5011	Yes
41	PITG_03033	PITG_03033			24574	-1.045	-0.4909	Yes
42	PITG_00688	PITG_00688			24703	-1.083	-0.4765	Yes
43	PITG_09445	PITG_09445			24733	-1.094	-0.4583	Yes
44	PITG_13683	PITG_13683			24801	-1.118	-0.4411	Yes
45	PITG_00997	PITG_00997			24825	-1.125	-0.4222	Yes
46	PITG_12264	PITG_12264			24859	-1.137	-0.4034	Yes
47	PITG_09438	PITG_09438			24904	-1.151	-0.3848	Yes
48	PITG_13912	PITG_13912			25644	-1.471	-0.3858	Yes
49	PITG_11036	PITG_11036			25653	-1.477	-0.3601	Yes
50	PITG_12427	PITG_12427			25703	-1.499	-0.3356	Yes
51	PITG_06595	PITG_06595			25709	-1.500	-0.3094	Yes
52	PITG_18776	PITG_18776			25758	-1.524	-0.2844	Yes
53	PITG_12049	PITG_12049			25954	-1.630	-0.2628	Yes
54	PITG_14612	PITG_14612			26049	-1.692	-0.2365	Yes
55	PITG_07792	PITG_07792			26226	-1.805	-0.2112	Yes
56	PITG_08565	PITG_08565			26338	-1.873	-0.1823	Yes
57	PITG_19880	PITG_19880			26383	-1.913	-0.1503	Yes
58	PITG_13682	PITG_13682			26458	-1.983	-0.1181	Yes
59	PITG_15526	PITG_15526			26465	-1.988	-0.0834	Yes
60	PITG_18354	PITG_18354			26745	-2.222	-0.0545	Yes
61	PITG_14936	PITG_14936			26810	-2.306	-0.0163	Yes
62	PITG_23229	PITG_23229			26975	-2.481	0.0213	Yes

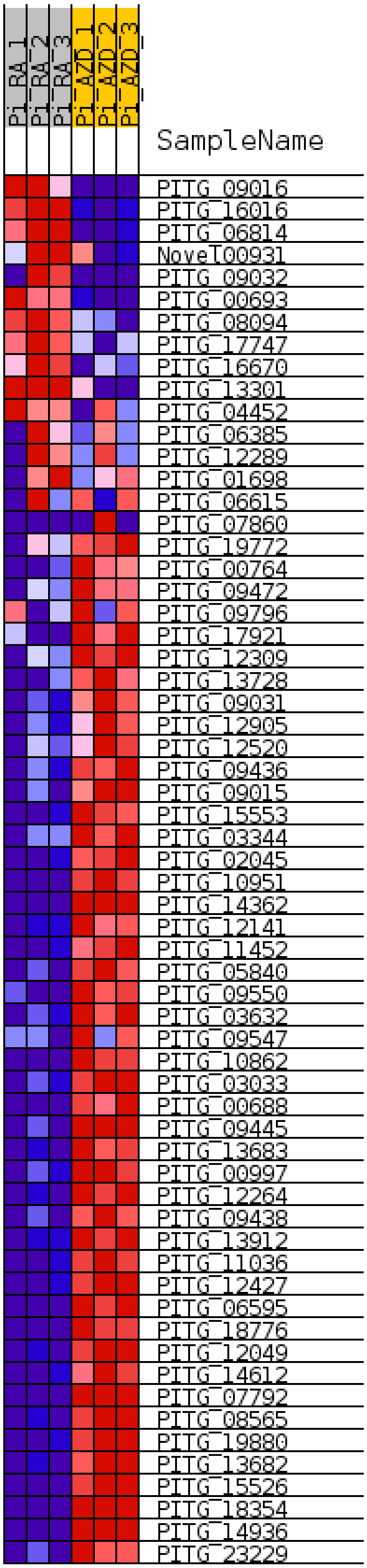

Fig 2: OXIDATIVE_PHOSPHORYLATION(PIF00190)
Blue-Pink O' Gram in the Space of the Analyzed GeneSet

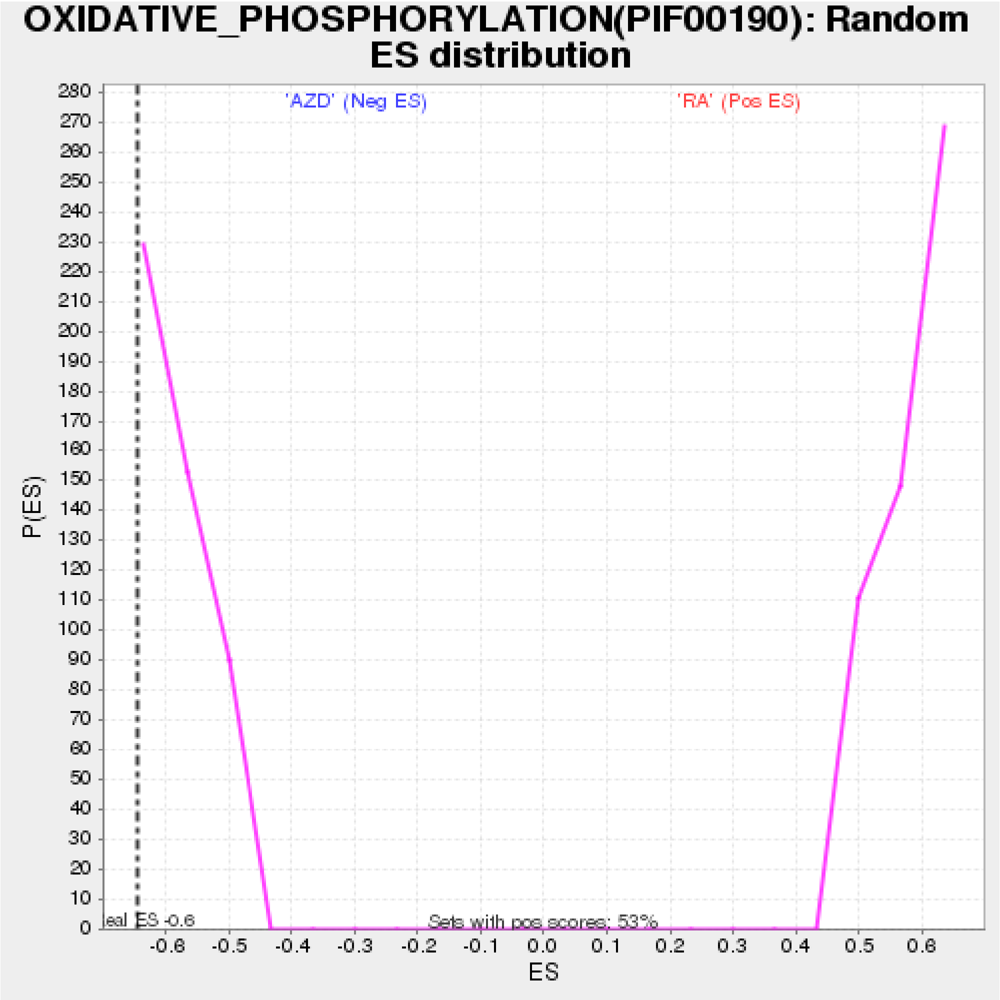

Fig 3: OXIDATIVE_PHOSPHORYLATION(PIF00190): Random ES distribution
Gene set null distribution of ES for OXIDATIVE_PHOSPHORYLATION(PIF00190)

5. Pentose phosphate pathway

Table: GSEA Results Summary

Dataset	fpkm.sample
Phenotype	sample.cls
Upregulated in class	AZD
GeneSet	PENTOSE_PHOSPHATE_PATHWAY(PIF00030)
Enrichment Score (ES)	-0.717205
Normalized Enrichment Score (NES)	-1.1303189
Nominal p-value	0.0
FDR q-value	0.16807897
FWER p-Value	0.179

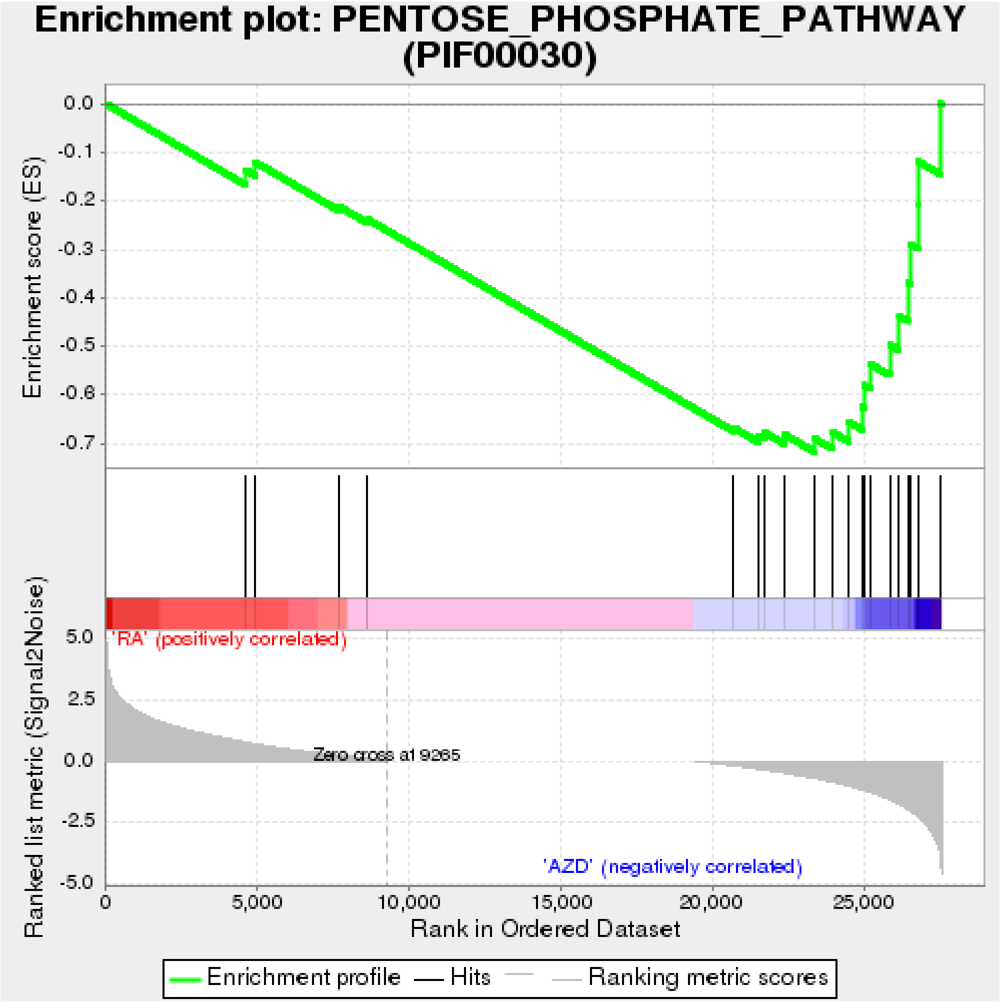

Fig 1: Enrichment plot: PENTOSE_PHOSPHATE_PATHWAY(PIF00030)
Profile of the Running ES Score & Positions of GeneSet Members on the Rank Ordered List

Table: GSEA details [plain text format]

	PROBE	DESCRIPTION	GENE	GENE_TITLE	RANK IN	RANK	RUNNING	CORE

		(from dataset)	SYMBOL		GENE LIST	METRIC SCORE	ES	ENRICHMENT
1	PITG_08598	PITG_08598			4584	0.780	-0.1352	No
2	PITG_02038	PITG_02038			4935	0.702	-0.1198	No
3	PITG_10032	PITG_10032			7703	0.220	-0.2115	No
4	PITG_02785	PITG_02785			8592	0.101	-0.2397	No
5	PITG_04665	PITG_04665			8634	0.096	-0.2373	No
6	PITG_03920	PITG_03920			20687	-0.190	-0.6674	No
7	PITG_01752	PITG_01752			21535	-0.332	-0.6848	No
8	PITG_03919	PITG_03919			21707	-0.362	-0.6766	No
9	PITG_00146	PITG_00146			22381	-0.486	-0.6815	No
10	PITG_01862	PITG_01862			23364	-0.696	-0.6893	Yes
11	PITG_21397	PITG_21397			23938	-0.851	-0.6761	Yes
12	PITG_19174	PITG_19174			24501	-1.018	-0.6558	Yes
13	PITG_09817	PITG_09817			24933	-1.163	-0.6249	Yes
14	PITG_18414	PITG_18414			25014	-1.196	-0.5799	Yes
15	PITG_20687	PITG_20687			25210	-1.266	-0.5363	Yes
16	PITG_05636	PITG_05636			25845	-1.568	-0.4966	Yes
17	PITG_05551	PITG_05551			26168	-1.770	-0.4375	Yes
18	PITG_03598	PITG_03598			26463	-1.987	-0.3687	Yes
19	PITG_03992	PITG_03992			26542	-2.040	-0.2899	Yes
20	PITG_02129	PITG_02129			26775	-2.258	-0.2079	Yes
21	PITG_04624	PITG_04624			26778	-2.260	-0.1176	Yes
22	PITG_21468	PITG_21468			27495	-3.648	0.0024	Yes

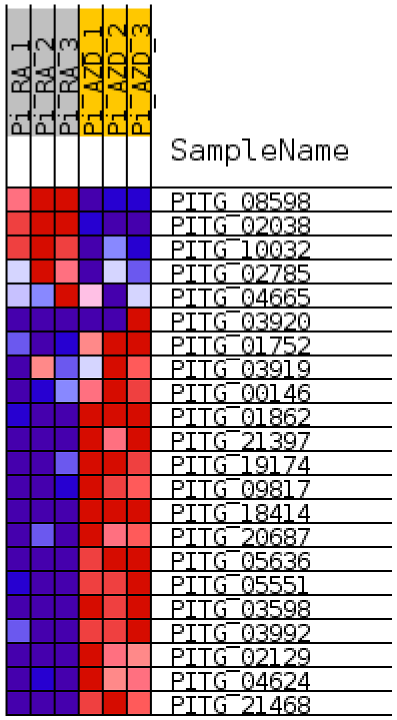

Fig 2: PENTOSE_PHOSPHATE_PATHWAY(PIF00030)
Blue-Pink O' Gram in the Space of the Analyzed GeneSet

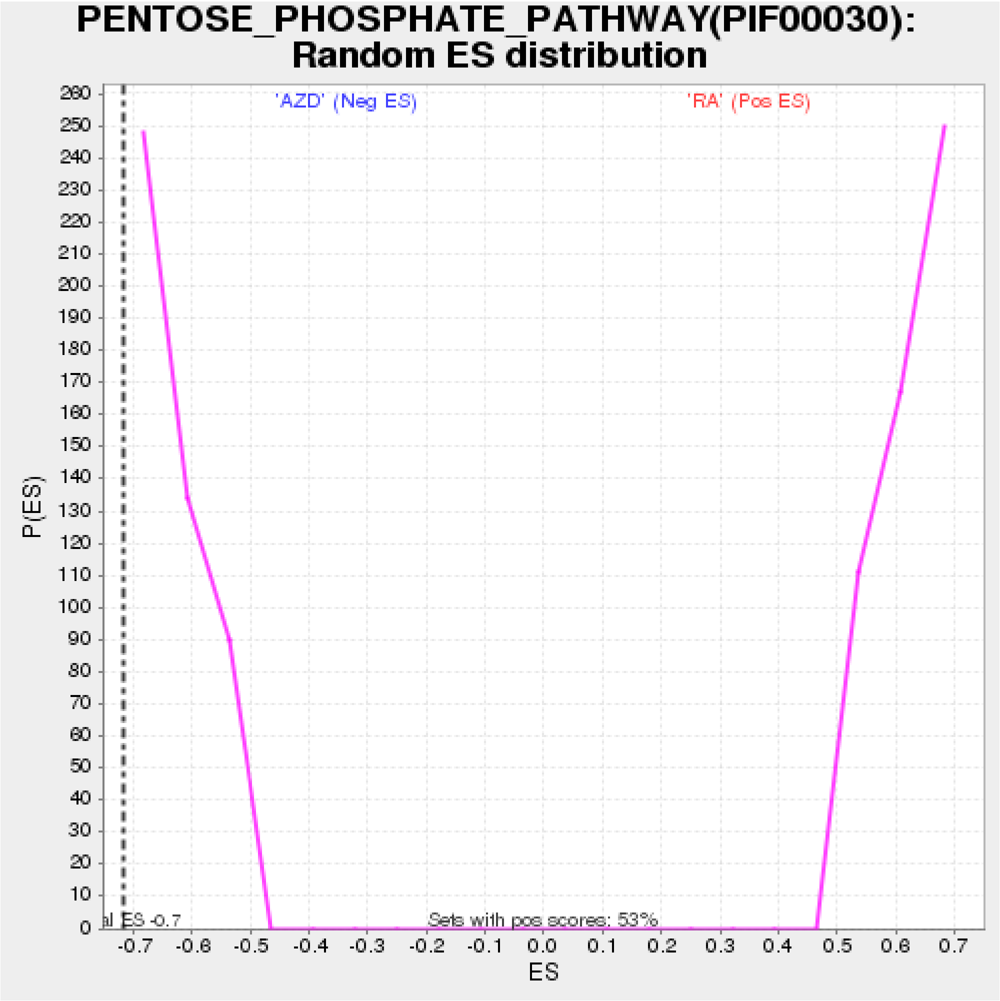

Fig 3: PENTOSE_PHOSPHATE_PATHWAY(PIF00030): Random ES distribution
Gene set null distribution of ES for PENTOSE_PHOSPHATE_PATHWAY(PIF00030)

6. Protein processing in endoplasmic reticulum

Table: GSEA Results Summary

Dataset	fpkm.sample
Phenotype	sample.cls
Upregulated in class	AZD
GeneSet	PROTEIN_PROCESSING_IN_ENDOPLASMIC_RETICULUM(PIF04141)
Enrichment Score (ES)	-0.6474953
Normalized Enrichment Score (NES)	-1.1959991
Nominal p-value	0.0
FDR q-value	0.1038136
FWER p-Value	0.049

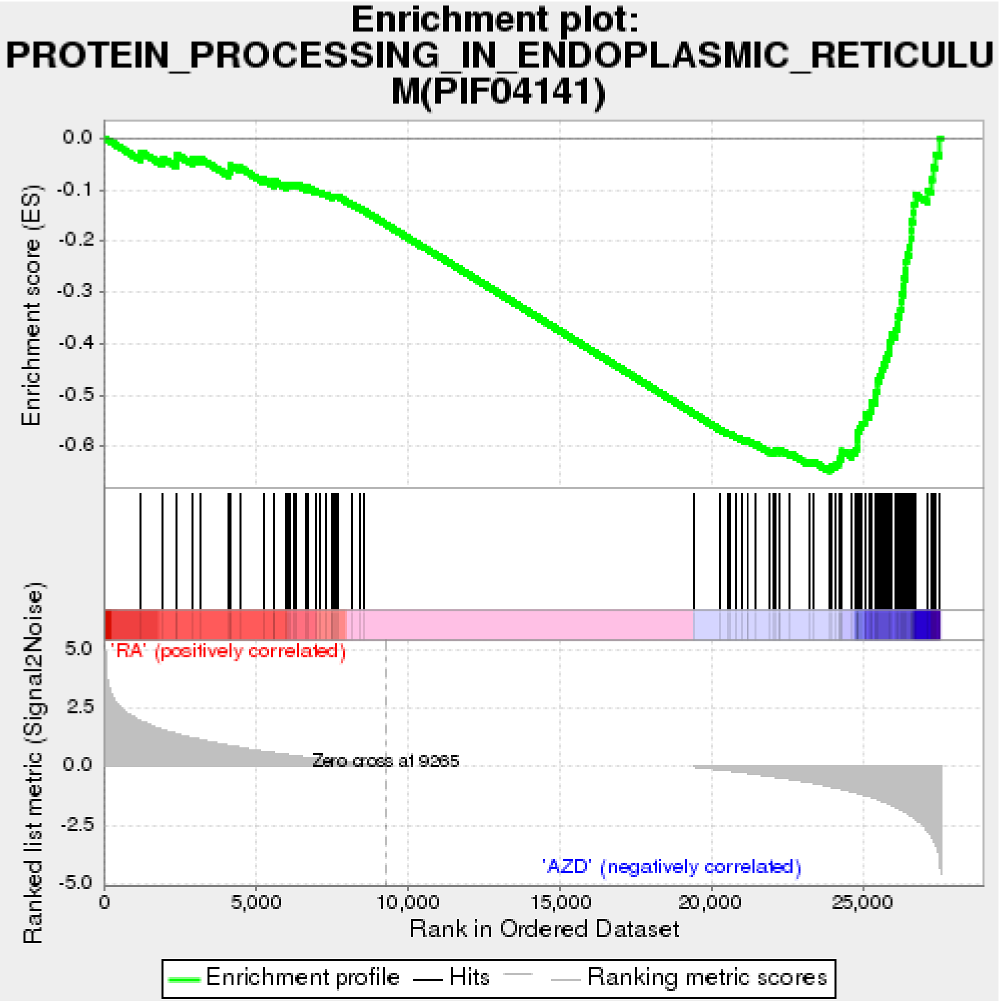

Fig 1: Enrichment plot: PROTEIN_PROCESSING_IN_ENDOPLASMIC_RETICULUM(PIF04141) Profile of the Running ES Score & Positions of GeneSet Members on the Rank Ordered List

Table: GSEA details [plain text format]

	PROBE	DESCRIPTION (from dataset)	GENE SYMBOL	GENE_TITLE	RANK IN GENE LIST	RANK METRIC SCORE	RUNNING ES	CORE ENRICHMENT
1	PITG_14396	PITG_14396			1180	1.945	-0.0262	No
2	PITG_21580	PITG_21580			1906	1.566	-0.0391	No
3	PITG_15216	PITG_15216			2324	1.403	-0.0422	No
4	PITG_06885	PITG_06885			2367	1.388	-0.0318	No
5	PITG_16065	PITG_16065			2884	1.228	-0.0400	No
6	PITG_06946	PITG_06946			3123	1.152	-0.0388	No
7	PITG_11252	PITG_11252			4069	0.903	-0.0654	No
8	PITG_10857	PITG_10857			4110	0.893	-0.0592	No
9	PITG_11728	PITG_11728			4139	0.885	-0.0526	No
10	PITG_17879	PITG_17879			4440	0.815	-0.0565	No
11	PITG_12120	PITG_12120			5226	0.646	-0.0795	No
12	PITG_06659	PITG_06659			5578	0.577	-0.0873	No
13	PITG_01181	PITG_01181			5588	0.575	-0.0827	No
14	PITG_08469	PITG_08469			5970	0.505	-0.0922	No
15	PITG_07083	PITG_07083			6016	0.497	-0.0896	No
16	PITG_17814	PITG_17814			6103	0.480	-0.0886	No
17	PITG_17383	PITG_17383			6246	0.457	-0.0898	No
18	PITG_13434	PITG_13434			6316	0.447	-0.0885	No
19	PITG_16528	PITG_16528			6654	0.388	-0.0974	No
20	PITG_01382	PITG_01382			6675	0.385	-0.0948	No
21	PITG_19537	PITG_19537			6977	0.330	-0.1029	No
22	PITG_15998	PITG_15998			7079	0.313	-0.1039	No
23	PITG_22715	PITG_22715			7265	0.287	-0.1082	No
24	PITG_13748	PITG_13748			7460	0.256	-0.1131	No
25	PITG_11886	PITG_11886			7489	0.251	-0.1119	No
26	PITG_18037	PITG_18037			7533	0.245	-0.1114	No
27	PITG_05781	PITG_05781			7620	0.232	-0.1125	No
28	PITG_09377	PITG_09377			7676	0.225	-0.1126	No
29	PITG_02211	PITG_02211			8116	0.169	-0.1271	No
30	PITG_14939	PITG_14939			8393	0.130	-0.1360	No
31	PITG_13234	PITG_13234			8512	0.112	-0.1394	No
32	PITG_01524	PITG_01524			19417	-0.004	-0.5364	No
33	PITG_00089	PITG_00089			20259	-0.125	-0.5660	No
34	PITG_15761	PITG_15761			20547	-0.168	-0.5750	No
35	PITG_10020	PITG_10020			20590	-0.175	-0.5750	No
36	PITG_01260	PITG_01260			20816	-0.208	-0.5814	No
37	PITG_09693	PITG_09693			21007	-0.241	-0.5863	No

38	PITG_13370	PITG_13370			21179	-0.270	-0.5902	No
39	PITG_02597	PITG_02597			21184	-0.271	-0.5880	No
40	PITG_10149	PITG_10149			21439	-0.316	-0.5945	No
41	PITG_07201	PITG_07201			21935	-0.406	-0.6090	No
42	PITG_04610	PITG_04610			22066	-0.429	-0.6101	No
43	PITG_16137	PITG_16137			22114	-0.436	-0.6080	No
44	PITG_06992	PITG_06992			22240	-0.457	-0.6086	No
45	PITG_00505	PITG_00505			22271	-0.462	-0.6058	No
46	PITG_06505	PITG_06505			22598	-0.532	-0.6131	No
47	PITG_05672	PITG_05672			23214	-0.667	-0.6297	No
48	PITG_19871	PITG_19871			23336	-0.691	-0.6282	No
49	PITG_11712	PITG_11712			23868	-0.828	-0.6404	Yes
50	PITG_12881	PITG_12881			23981	-0.864	-0.6370	Yes
51	PITG_02584	PITG_02584			24123	-0.906	-0.6343	Yes
52	PITG_01058	PITG_01058			24195	-0.926	-0.6289	Yes
53	PITG_20714	PITG_20714			24254	-0.946	-0.6229	Yes
54	PITG_02867	PITG_02867			24296	-0.957	-0.6162	Yes
55	PITG_07061	PITG_07061			24313	-0.962	-0.6084	Yes
56	PITG_11703	PITG_11703			24648	-1.067	-0.6114	Yes
57	PITG_07885	PITG_07885			24738	-1.095	-0.6052	Yes
58	PITG_03694	PITG_03694			24793	-1.116	-0.5976	Yes
59	PITG_03468	PITG_03468			24797	-1.117	-0.5881	Yes
60	Novel00522	Novel00522			24826	-1.126	-0.5794	Yes
61	PITG_18107	PITG_18107			24833	-1.129	-0.5699	Yes
62	PITG_06795	PITG_06795			24898	-1.149	-0.5623	Yes
63	PITG_05501	PITG_05501			24970	-1.179	-0.5548	Yes
64	PITG_16366	PITG_16366			25055	-1.210	-0.5474	Yes
65	PITG_03390	PITG_03390			25057	-1.210	-0.5370	Yes
66	PITG_19557	PITG_19557			25245	-1.278	-0.5328	Yes
67	PITG_21378	PITG_21378			25275	-1.291	-0.5228	Yes
68	PITG_06657	PITG_06657			25284	-1.296	-0.5119	Yes
69	PITG_05498	PITG_05498			25419	-1.365	-0.5050	Yes
70	PITG_17592	PITG_17592			25433	-1.369	-0.4937	Yes
71	PITG_12260	PITG_12260			25467	-1.383	-0.4830	Yes
72	PITG_03068	PITG_03068			25469	-1.385	-0.4711	Yes
73	PITG_11704	PITG_11704			25523	-1.408	-0.4609	Yes
74	PITG_01855	PITG_01855			25614	-1.455	-0.4516	Yes
75	PITG_07843	PITG_07843			25651	-1.475	-0.4402	Yes
76	PITG_13151	PITG_13151			25715	-1.503	-0.4296	Yes

77	PITG_10972	PITG_10972			25794	-1.545	-0.4191	Yes
78	PITG_01858	PITG_01858			25865	-1.577	-0.4081	Yes
79	PITG_02266	PITG_02266			25882	-1.584	-0.3950	Yes
80	PITG_12694	PITG_12694			25914	-1.602	-0.3824	Yes
81	PITG_03594	PITG_03594			26074	-1.708	-0.3735	Yes
82	PITG_18053	PITG_18053			26117	-1.734	-0.3600	Yes
83	PITG_08328	PITG_08328			26159	-1.765	-0.3463	Yes
84	PITG_17683	PITG_17683			26233	-1.811	-0.3334	Yes
85	PITG_15358	PITG_15358			26268	-1.834	-0.3188	Yes
86	PITG_08327	PITG_08327			26287	-1.844	-0.3036	Yes
87	PITG_11244	PITG_11244			26344	-1.877	-0.2895	Yes
88	PITG_09453	PITG_09453			26362	-1.894	-0.2738	Yes
89	PITG_00632	PITG_00632			26390	-1.921	-0.2582	Yes
90	PITG_03388	PITG_03388			26421	-1.947	-0.2426	Yes
91	PITG_02467	PITG_02467			26440	-1.968	-0.2263	Yes
92	PITG_18934	PITG_18934			26517	-2.024	-0.2116	Yes
93	PITG_09965	PITG_09965			26566	-2.058	-0.1956	Yes
94	PITG_22023	PITG_22023			26579	-2.075	-0.1782	Yes
95	PITG_15771	PITG_15771			26590	-2.082	-0.1606	Yes
96	PITG_18089	PITG_18089			26645	-2.134	-0.1442	Yes
97	PITG_17637	PITG_17637			26676	-2.159	-0.1267	Yes
98	PITG_00527	PITG_00527			26750	-2.230	-0.1101	Yes
99	PITG_09451	PITG_09451			27131	-2.724	-0.1005	Yes
100	PITG_11247	PITG_11247			27265	-2.968	-0.0798	Yes
101	PITG_11249	PITG_11249			27293	-3.011	-0.0548	Yes
102	PITG_03546	PITG_03546			27410	-3.357	-0.0301	Yes
103	PITG_11913	PITG_11913			27550	-4.134	0.0004	Yes

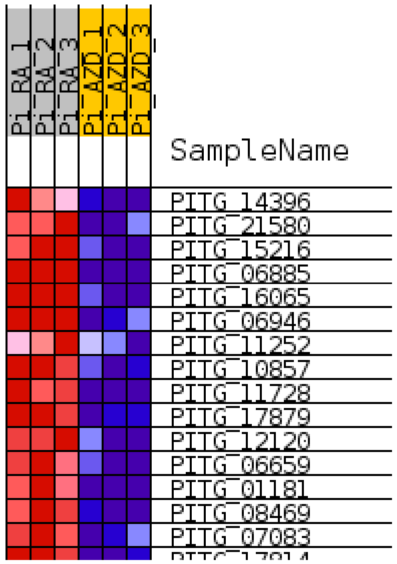

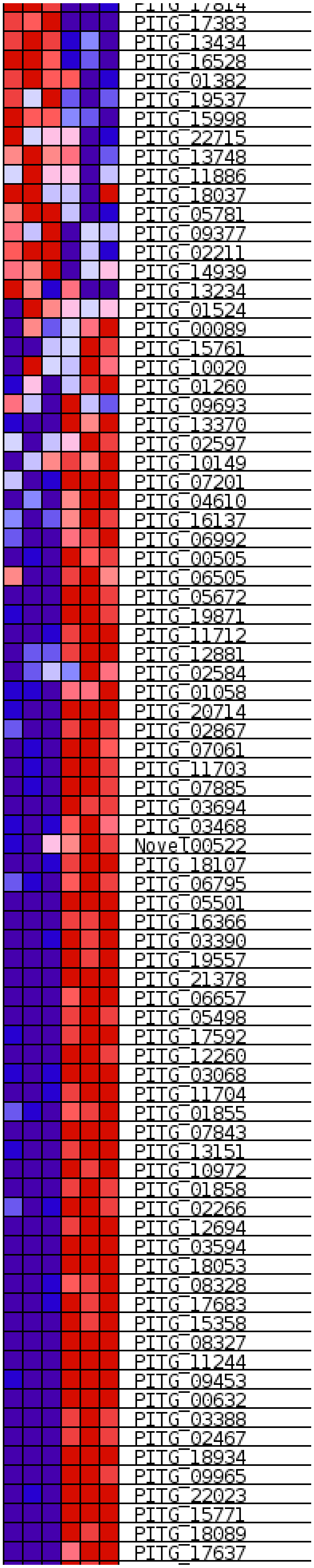
PITG_17383
PITG_13434
PITG_16528
PITG_01382
PITG_19537
PITG_15998
PITG_22715
PITG_13748
PITG_11886
PITG_18037
PITG_05781
PITG_09377
PITG_02211
PITG_14939
PITG_13234
PITG_01524
PITG_00089
PITG_15761
PITG_10020
PITG_01260
PITG_09693
PITG_13370
PITG_02597
PITG_10149
PITG_07201
PITG_04610
PITG_16137
PITG_06992
PITG_00505
PITG_06505
PITG_05672
PITG_19871
PITG_11712
PITG_12881
PITG_02584
PITG_01058
PITG_20714
PITG_02867
PITG_07061
PITG_11703
PITG_07885
PITG_03694
PITG_03468
Novel00522
PITG_18107
PITG_06795
PITG_05501
PITG_16366
PITG_03390
PITG_19557
PITG_21378
PITG_06657
PITG_05498
PITG_17592
PITG_12260
PITG_03068
PITG_11704
PITG_01855
PITG_07843
PITG_13151
PITG_10972
PITG_01858
PITG_02266
PITG_12694
PITG_03594
PITG_18053
PITG_08328
PITG_17683
PITG_15358
PITG_08327
PITG_11244
PITG_09453
PITG_00632
PITG_03388
PITG_02467
PITG_18934
PITG_09965
PITG_22023
PITG_15771
PITG_18089
PITG_17637

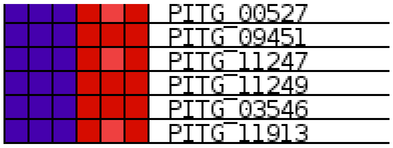

Fig 2: PROTEIN_PROCESSING_IN_ENDOPLASMIC_RETICULUM(PIF04141)
Blue-Pink O' Gram in the Space of the Analyzed GeneSet

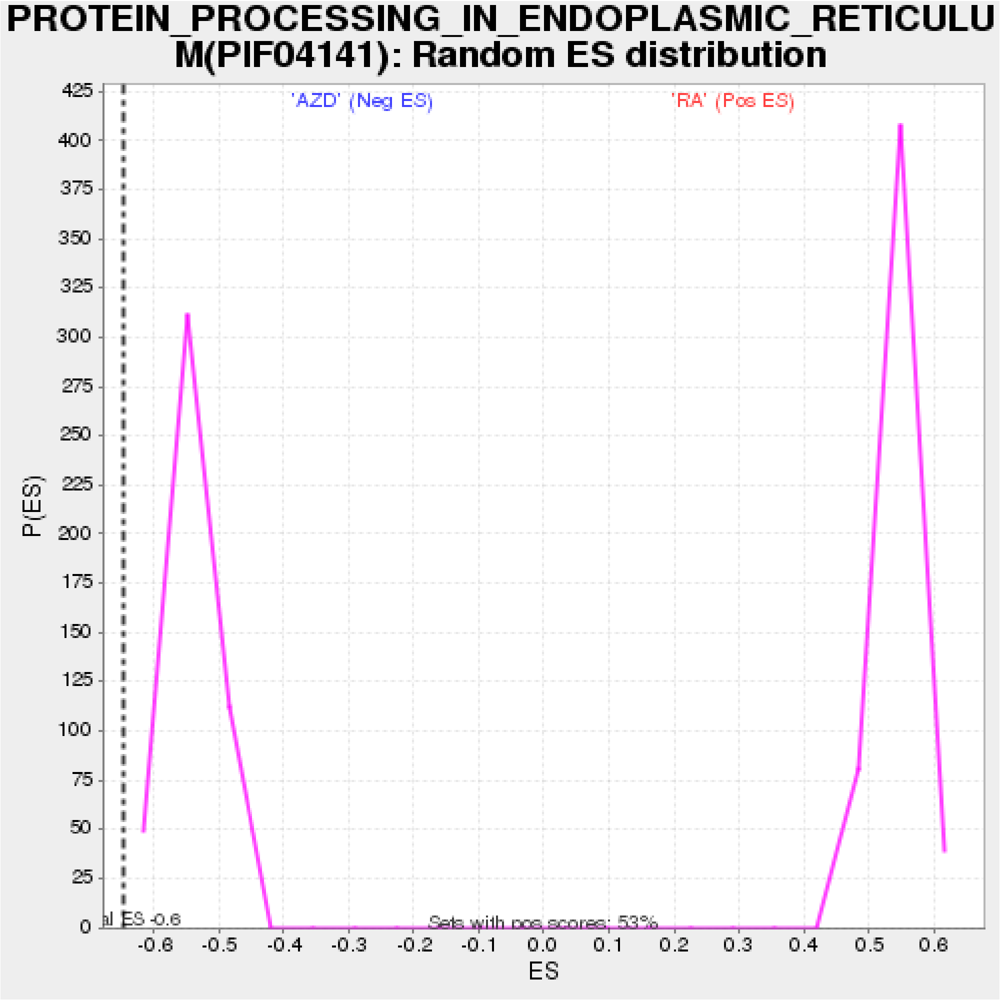

Fig 3: PROTEIN_PROCESSING_IN_ENDOPLASMIC_RETICULUM(PIF04141): Random ES distribution
Gene set null distribution of ES for
PROTEIN_PROCESSING_IN_ENDOPLASMIC_RETICULUM(PIF04141)

7. Ribosome

Table: GSEA Results Summary

Dataset	fpkm.sample
Phenotype	sample.cls
Upregulated in class	AZD
GeneSet	RIBOSOME(PIF03010)
Enrichment Score (ES)	-0.7706513
Normalized Enrichment Score (NES)	-1.0528373
Nominal p-value	0.108050846
FDR q-value	0.23352191
FWER p-Value	0.376

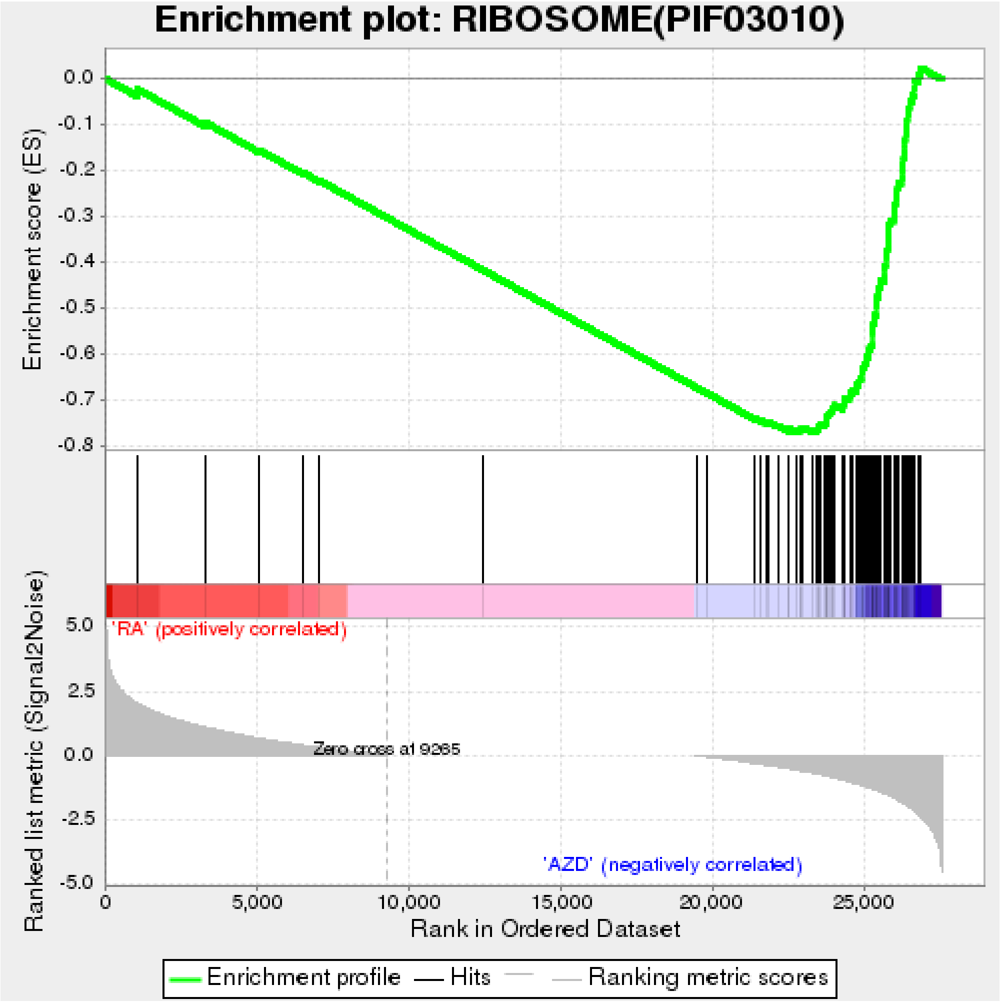

Fig 1: Enrichment plot: RIBOSOME(PIF03010)
Profile of the Running ES Score & Positions of GeneSet Members on the Rank Ordered List

Table: GSEA details [plain text format]

	PROBE	DESCRIPTION	GENE	GENE_TITLE	RANK IN	RANK	RUNNING	CORE

		(from dataset)	SYMBOL		GENE LIST	METRIC SCORE	ES	ENRICHMENT
1	PITG_19121	PITG_19121			1014	2.048	-0.0211	No
2	PITG_03660	PITG_03660			3265	1.116	-0.0944	No
3	PITG_04337	PITG_04337			5068	0.678	-0.1548	No
4	PITG_09430	PITG_09430			6515	0.410	-0.2043	No
5	PITG_05009	PITG_05009			7048	0.319	-0.2212	No
6	PITG_15722	PITG_15722			12398	0.000	-0.4160	No
7	PITG_10193	PITG_10193			19492	-0.015	-0.6742	No
8	PITG_13500	PITG_13500			19785	-0.055	-0.6844	No
9	PITG_11734	PITG_11734			21411	-0.312	-0.7412	No
10	PITG_09442	PITG_09442			21613	-0.345	-0.7459	No
11	PITG_14850	PITG_14850			21815	-0.384	-0.7502	No
12	PITG_16757	PITG_16757			21821	-0.385	-0.7474	No
13	PITG_20188	PITG_20188			22157	-0.445	-0.7562	No
14	PITG_19669	PITG_19669			22503	-0.510	-0.7648	No
15	PITG_12839	PITG_12839			22518	-0.514	-0.7613	No
16	PITG_16198	PITG_16198			22775	-0.573	-0.7662	Yes
17	PITG_19999	PITG_19999			22878	-0.594	-0.7653	Yes
18	PITG_00443	PITG_00443			22936	-0.604	-0.7627	Yes
19	PITG_15697	PITG_15697			22991	-0.618	-0.7599	Yes
20	PITG_12745	PITG_12745			23280	-0.681	-0.7652	Yes
21	PITG_03916	PITG_03916			23421	-0.710	-0.7648	Yes
22	PITG_15090	PITG_15090			23482	-0.725	-0.7613	Yes
23	PITG_04843	PITG_04843			23523	-0.735	-0.7571	Yes
24	PITG_16008	PITG_16008			23545	-0.740	-0.7521	Yes
25	PITG_06771	PITG_06771			23729	-0.789	-0.7527	Yes
26	PITG_00266	PITG_00266			23767	-0.800	-0.7479	Yes
27	PITG_07269	PITG_07269			23787	-0.807	-0.7423	Yes
28	PITG_10887	PITG_10887			23788	-0.807	-0.7361	Yes
29	PITG_20189	PITG_20189			23835	-0.820	-0.7314	Yes
30	PITG_03420	PITG_03420			23870	-0.829	-0.7262	Yes
31	PITG_09563	PITG_09563			23937	-0.851	-0.7221	Yes
32	PITG_19007	PITG_19007			23986	-0.865	-0.7171	Yes
33	PITG_01922	PITG_01922			24019	-0.874	-0.7115	Yes
34	PITG_21503	PITG_21503			24287	-0.953	-0.7139	Yes
35	PITG_02694	PITG_02694			24328	-0.970	-0.7078	Yes
36	PITG_04487	PITG_04487			24365	-0.980	-0.7016	Yes
37	PITG_01943	PITG_01943			24378	-0.982	-0.6944	Yes
38	PITG_22323	PITG_22323			24524	-1.029	-0.6917	Yes

39	PITG_03221	PITG_03221			24557	-1.040	-0.6848	Yes
40	PITG_11766	PITG_11766			24642	-1.064	-0.6797	Yes
41	PITG_06995	PITG_06995			24760	-1.104	-0.6754	Yes
42	PITG_01833	PITG_01833			24767	-1.106	-0.6671	Yes
43	PITG_13676	PITG_13676			24803	-1.119	-0.6597	Yes
44	PITG_08834	PITG_08834			24852	-1.136	-0.6526	Yes
45	PITG_17261	PITG_17261			24920	-1.156	-0.6461	Yes
46	PITG_05171	PITG_05171			24921	-1.158	-0.6372	Yes
47	PITG_00179	PITG_00179			24952	-1.171	-0.6292	Yes
48	PITG_15069	PITG_15069			25019	-1.198	-0.6223	Yes
49	PITG_00941	PITG_00941			25067	-1.214	-0.6147	Yes
50	PITG_03477	PITG_03477			25104	-1.229	-0.6065	Yes
51	PITG_12697	PITG_12697			25164	-1.249	-0.5990	Yes
52	PITG_13371	PITG_13371			25168	-1.249	-0.5894	Yes
53	PITG_15407	PITG_15407			25203	-1.263	-0.5809	Yes
54	PITG_14608	PITG_14608			25265	-1.288	-0.5731	Yes
55	PITG_05174	PITG_05174			25267	-1.288	-0.5632	Yes
56	PITG_09627	PITG_09627			25269	-1.289	-0.5533	Yes
57	PITG_03294	PITG_03294			25303	-1.306	-0.5444	Yes
58	PITG_08703	PITG_08703			25308	-1.308	-0.5344	Yes
59	PITG_17093	PITG_17093			25335	-1.321	-0.5251	Yes
60	PITG_07173	PITG_07173			25342	-1.325	-0.5151	Yes
61	PITG_13312	PITG_13312			25404	-1.355	-0.5068	Yes
62	PITG_14913	PITG_14913			25405	-1.356	-0.4963	Yes
63	PITG_22135	PITG_22135			25416	-1.361	-0.4862	Yes
64	PITG_18052	PITG_18052			25424	-1.365	-0.4759	Yes
65	PITG_08959	PITG_08959			25458	-1.380	-0.4664	Yes
66	PITG_03235	PITG_03235			25480	-1.389	-0.4564	Yes
67	PITG_14729	PITG_14729			25528	-1.410	-0.4472	Yes
68	PITG_09506	PITG_09506			25563	-1.431	-0.4374	Yes
69	PITG_10146	PITG_10146			25685	-1.491	-0.4303	Yes
70	PITG_09540	PITG_09540			25692	-1.493	-0.4189	Yes
71	PITG_00523	PITG_00523			25696	-1.497	-0.4075	Yes
72	PITG_10863	PITG_10863			25713	-1.502	-0.3964	Yes
73	PITG_11099	PITG_11099			25716	-1.504	-0.3849	Yes
74	PITG_06237	PITG_06237			25757	-1.523	-0.3746	Yes
75	PITG_03353	PITG_03353			25780	-1.535	-0.3635	Yes
76	PITG_20798	PITG_20798			25789	-1.543	-0.3519	Yes
77	PITG_07300	PITG_07300			25805	-1.550	-0.3404	Yes

78	PITG_10263	PITG_10263			25818	-1.555	-0.3288	Yes
79	PITG_20264	PITG_20264			25831	-1.561	-0.3172	Yes
80	PITG_08548	PITG_08548			25856	-1.574	-0.3059	Yes
81	PITG_03486	PITG_03486			25977	-1.641	-0.2976	Yes
82	PITG_02053	PITG_02053			25987	-1.650	-0.2851	Yes
83	PITG_12947	PITG_12947			26003	-1.658	-0.2728	Yes
84	PITG_03239	PITG_03239			26044	-1.683	-0.2613	Yes
85	PITG_02578	PITG_02578			26070	-1.705	-0.2490	Yes
86	PITG_09525	PITG_09525			26102	-1.724	-0.2368	Yes
87	PITG_03178	PITG_03178			26113	-1.732	-0.2238	Yes
88	PITG_13681	PITG_13681			26238	-1.816	-0.2142	Yes
89	PITG_15638	PITG_15638			26261	-1.830	-0.2009	Yes
90	PITG_09552	PITG_09552			26270	-1.835	-0.1870	Yes
91	PITG_19157	PITG_19157			26301	-1.850	-0.1738	Yes
92	PITG_01217	PITG_01217			26311	-1.855	-0.1597	Yes
93	PITG_09345	PITG_09345			26320	-1.862	-0.1456	Yes
94	PITG_03762	PITG_03762			26325	-1.866	-0.1313	Yes
95	PITG_06636	PITG_06636			26370	-1.898	-0.1183	Yes
96	PITG_01042	PITG_01042			26385	-1.915	-0.1040	Yes
97	PITG_09555	PITG_09555			26429	-1.950	-0.0904	Yes
98	PITG_10202	PITG_10202			26436	-1.959	-0.0755	Yes
99	PITG_20795	PITG_20795			26482	-2.001	-0.0617	Yes
100	PITG_01041	PITG_01041			26502	-2.013	-0.0468	Yes
101	PITG_00631	PITG_00631			26594	-2.084	-0.0340	Yes
102	PITG_20116	PITG_20116			26650	-2.137	-0.0195	Yes
103	PITG_06799	PITG_06799			26687	-2.169	-0.0040	Yes
104	PITG_03768	PITG_03768			26780	-2.261	0.0101	Yes
105	PITG_18054	PITG_18054			26868	-2.362	0.0252	Yes

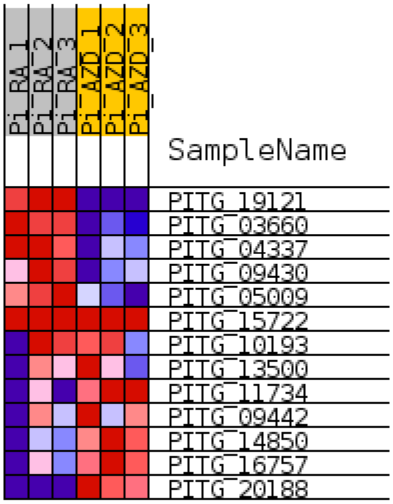

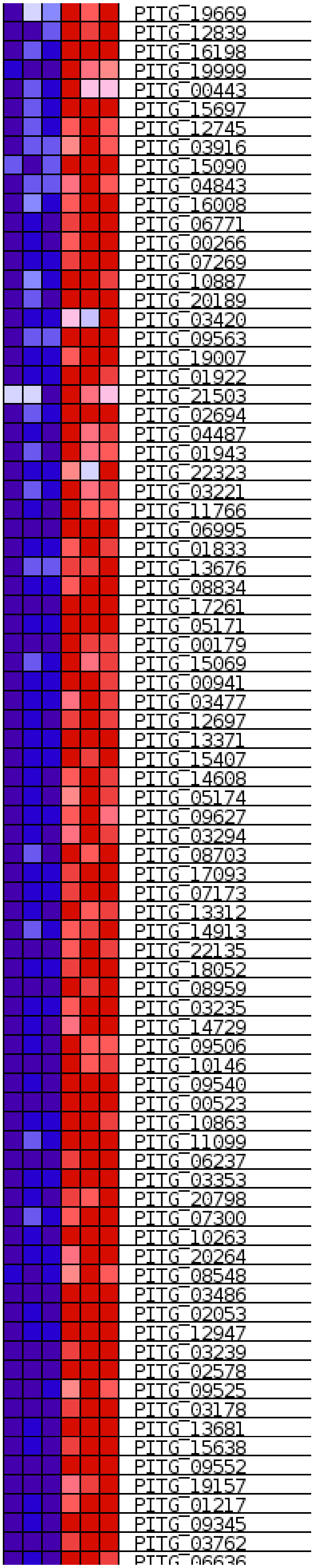
PITG_19669
PITG_12839
PITG_16198
PITG_19999
PITG_00443
PITG_15697
PITG_12745
PITG_03916
PITG_15090
PITG_04843
PITG_16008
PITG_06771
PITG_00266
PITG_07269
PITG_10887
PITG_20189
PITG_03420
PITG_09563
PITG_19007
PITG_01922
PITG_21503
PITG_02694
PITG_04487
PITG_01943
PITG_22323
PITG_03221
PITG_11766
PITG_06995
PITG_01833
PITG_13676
PITG_08834
PITG_17261
PITG_05171
PITG_00179
PITG_15069
PITG_00941
PITG_03477
PITG_12697
PITG_13371
PITG_15407
PITG_14608
PITG_05174
PITG_09627
PITG_03294
PITG_08703
PITG_17093
PITG_07173
PITG_13312
PITG_14913
PITG_22135
PITG_18052
PITG_08959
PITG_03235
PITG_14729
PITG_09506
PITG_10146
PITG_09540
PITG_00523
PITG_10863
PITG_11099
PITG_06237
PITG_03353
PITG_20798
PITG_07300
PITG_10263
PITG_20264
PITG_08548
PITG_03486
PITG_02053
PITG_12947
PITG_03239
PITG_02578
PITG_09525
PITG_03178
PITG_13681
PITG_15638
PITG_09552
PITG_19157
PITG_01217
PITG_09345
PITG_03762
PITG_06636

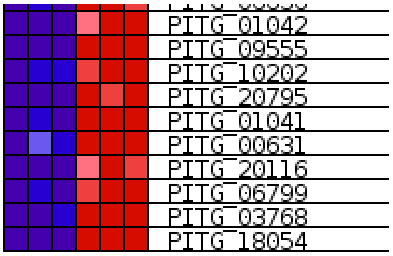

Fig 2: RIBOSOME(PIF03010)
lue-Pink O' Gram in the Space of the Analyzed GeneSet

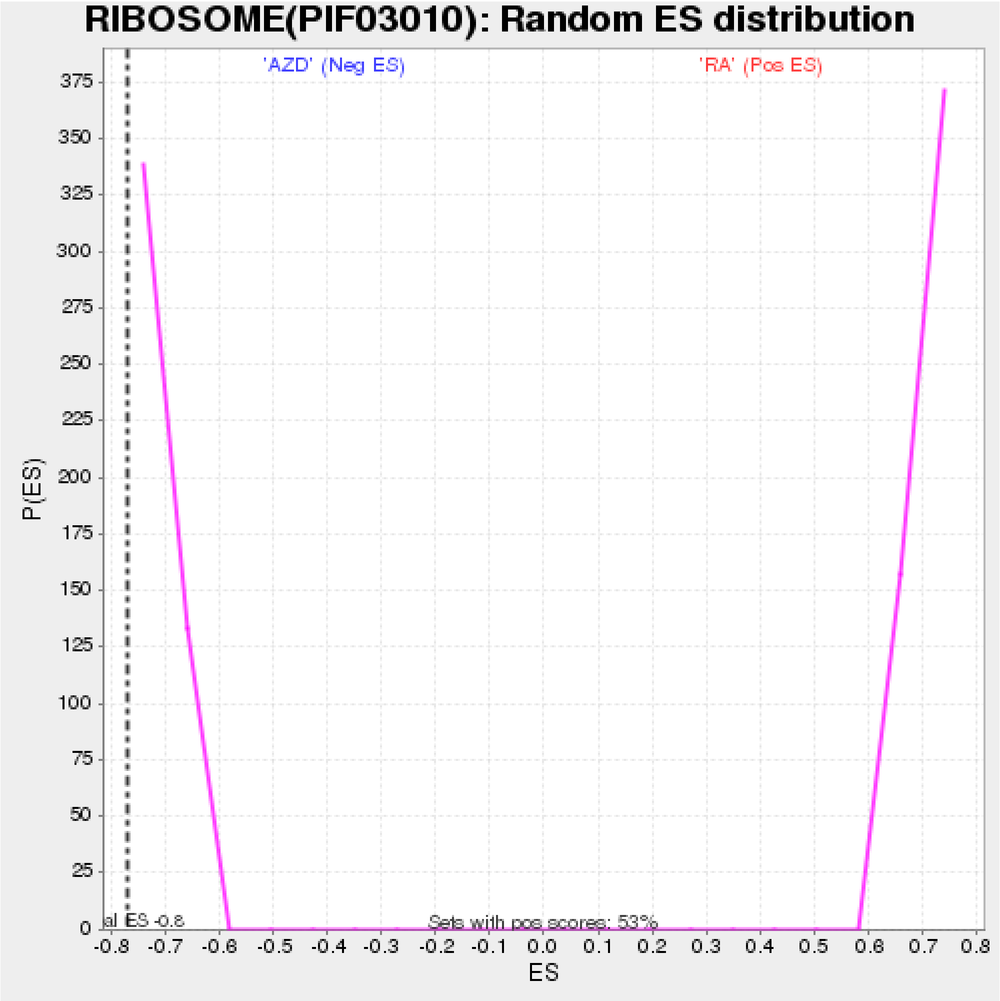

Fig 3: RIBOSOME(PIF03010): Random ES distribution
Gene set null distribution of ES for RIBOSOME(PIF03010)

8. Ribosome biogenesis in eukaryotes

Table: GSEA Results Summary

Dataset	fpkm.sample
Phenotype	sample.cls
Upregulated in class	AZD
GeneSet	RIBOSOME_BIOGENESIS_IN_EUKARYOTES(PIF03008)
Enrichment Score (ES)	-0.8229289
Normalized Enrichment Score (NES)	-1.2382945
Nominal p-value	0.0
FDR q-value	0.1038136
FWER p-Value	0.0

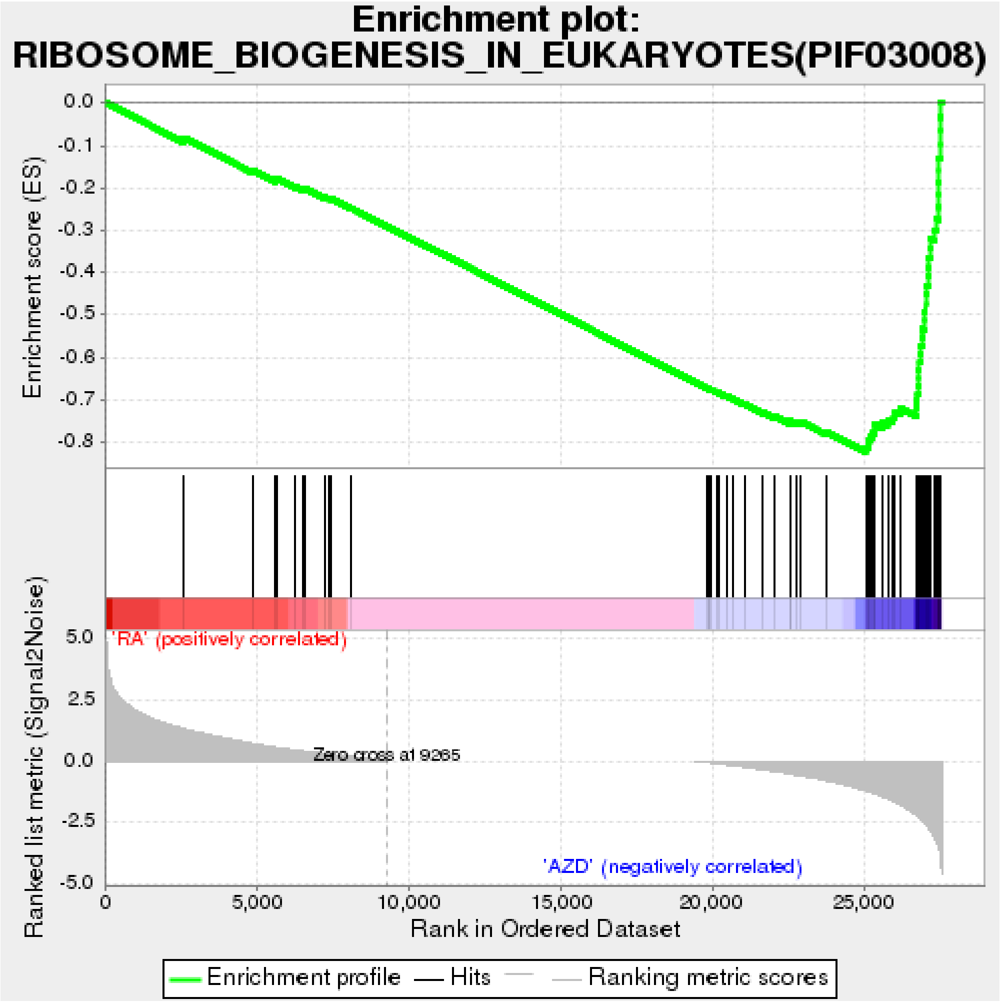

Fig 1: Enrichment plot: RIBOSOME_BIOGENESIS_IN_EUKARYOTES(PIF03008)
Profile of the Running ES Score & Positions of GeneSet Members on the Rank Ordered List

Table: GSEA details [plain text format]

	PROBE	DESCRIPTION	GENE	GENE_TITLE	RANK IN	RANK	RUNNING	CORE

		(from dataset)	SYMBOL		GENE LIST	METRIC SCORE	ES	ENRICHMENT
1	PITG_04584	PITG_04584			2556	1.327	-0.0818	No
2	PITG_00401	PITG_00401			4838	0.723	-0.1587	No
3	PITG_06700	PITG_06700			5567	0.579	-0.1803	No
4	PITG_17205	PITG_17205			5621	0.570	-0.1774	No
5	PITG_16556	PITG_16556			6234	0.458	-0.1959	No
6	PITG_05058	PITG_05058			6473	0.418	-0.2010	No
7	PITG_06679	PITG_06679			6557	0.404	-0.2006	No
8	PITG_08447	PITG_08447			7207	0.298	-0.2217	No
9	PITG_05240	PITG_05240			7362	0.272	-0.2250	No
10	PITG_00194	PITG_00194			7427	0.260	-0.2252	No
11	PITG_02745	PITG_02745			8107	0.170	-0.2484	No
12	PITG_12932	PITG_12932			19783	-0.055	-0.6727	No
13	PITG_12465	PITG_12465			19843	-0.063	-0.6743	No
14	PITG_03802	PITG_03802			19849	-0.064	-0.6739	No
15	PITG_10163	PITG_10163			19967	-0.085	-0.6775	No
16	PITG_21600	PITG_21600			20118	-0.106	-0.6820	No
17	PITG_00787	PITG_00787			20201	-0.119	-0.6840	No
18	PITG_03818	PITG_03818			20478	-0.160	-0.6927	No
19	PITG_12107	PITG_12107			20646	-0.184	-0.6972	No
20	PITG_07174	PITG_07174			21069	-0.253	-0.7105	No
21	PITG_22541	PITG_22541			21685	-0.357	-0.7298	No
22	PITG_18421	PITG_18421			22080	-0.431	-0.7405	No
23	PITG_17724	PITG_17724			22554	-0.523	-0.7533	No
24	PITG_18718	PITG_18718			22589	-0.530	-0.7501	No
25	PITG_17674	PITG_17674			22802	-0.578	-0.7530	No
26	PITG_17404	PITG_17404			22932	-0.603	-0.7526	No
27	PITG_00035	PITG_00035			23771	-0.802	-0.7763	No
28	PITG_11635	PITG_11635			25053	-1.209	-0.8128	Yes
29	PITG_01725	PITG_01725			25143	-1.242	-0.8055	Yes
30	PITG_15975	PITG_15975			25170	-1.250	-0.7960	Yes
31	PITG_12305	PITG_12305			25229	-1.272	-0.7874	Yes
32	PITG_00032	PITG_00032			25301	-1.306	-0.7790	Yes
33	PITG_00748	PITG_00748			25366	-1.336	-0.7701	Yes
34	PITG_03672	PITG_03672			25370	-1.338	-0.7589	Yes
35	PITG_08708	PITG_08708			25601	-1.449	-0.7551	Yes
36	PITG_06262	PITG_06262			25777	-1.533	-0.7486	Yes
37	PITG_19671	PITG_19671			25970	-1.638	-0.7418	Yes
38	PITG_12871	PITG_12871			26033	-1.673	-0.7299	Yes

39	PITG_01019	PITG_01019			26205	-1.792	-0.7211	Yes
40	PITG_00036	PITG_00036			26717	-2.196	-0.7212	Yes
41	PITG_05445	PITG_05445			26729	-2.205	-0.7031	Yes
42	PITG_16813	PITG_16813			26762	-2.241	-0.6854	Yes
43	PITG_04887	PITG_04887			26801	-2.288	-0.6675	Yes
44	PITG_02536	PITG_02536			26817	-2.308	-0.6486	Yes
45	PITG_01076	PITG_01076			26820	-2.312	-0.6292	Yes
46	PITG_05630	PITG_05630			26829	-2.317	-0.6100	Yes
47	PITG_12931	PITG_12931			26872	-2.368	-0.5917	Yes
48	PITG_13300	PITG_13300			26884	-2.376	-0.5721	Yes
49	PITG_16792	PITG_16792			26907	-2.395	-0.5527	Yes
50	PITG_19178	PITG_19178			26926	-2.423	-0.5330	Yes
51	PITG_18717	PITG_18717			26974	-2.481	-0.5138	Yes
52	PITG_10819	PITG_10819			26977	-2.484	-0.4930	Yes
53	PITG_13380	PITG_13380			27028	-2.559	-0.4733	Yes
54	PITG_00052	PITG_00052			27030	-2.560	-0.4518	Yes
55	PITG_00142	PITG_00142			27051	-2.597	-0.4307	Yes
56	PITG_02593	PITG_02593			27105	-2.686	-0.4100	Yes
57	PITG_09374	PITG_09374			27116	-2.701	-0.3877	Yes
58	PITG_09222	PITG_09222			27134	-2.729	-0.3653	Yes
59	PITG_19608	PITG_19608			27170	-2.799	-0.3430	Yes
60	PITG_15652	PITG_15652			27191	-2.838	-0.3199	Yes
61	PITG_08338	PITG_08338			27312	-3.069	-0.2984	Yes
62	PITG_07308	PITG_07308			27405	-3.341	-0.2737	Yes
63	PITG_06667	PITG_06667			27438	-3.428	-0.2460	Yes
64	PITG_02887	PITG_02887			27445	-3.461	-0.2171	Yes
65	PITG_03955	PITG_03955			27450	-3.473	-0.1880	Yes
66	PITG_00597	PITG_00597			27479	-3.555	-0.1592	Yes
67	PITG_05534	PITG_05534			27481	-3.557	-0.1293	Yes
68	PITG_09432	PITG_09432			27523	-3.848	-0.0984	Yes
69	PITG_11198	PITG_11198			27526	-3.854	-0.0660	Yes
70	PITG_20593	PITG_20593			27530	-3.884	-0.0335	Yes
71	PITG_06263	PITG_06263			27548	-4.108	0.0005	Yes

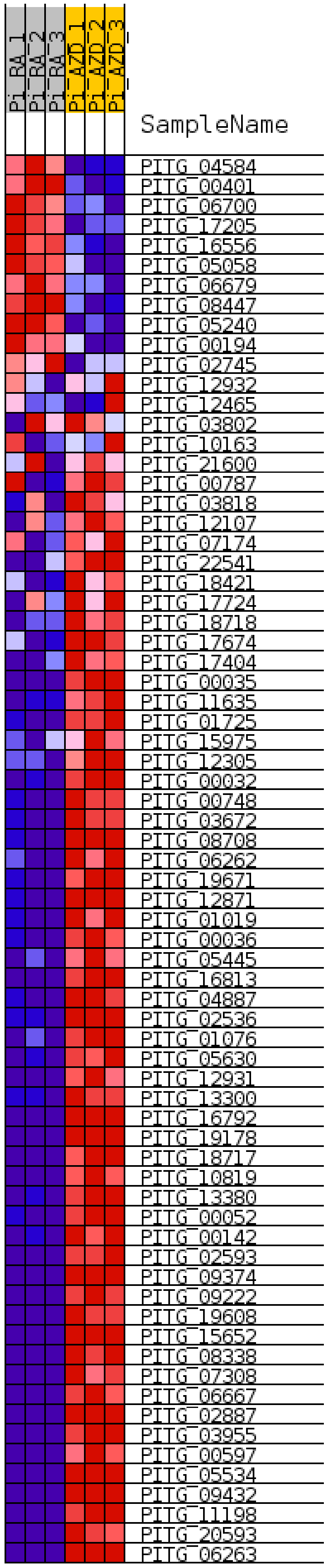
Pi_RA_1
Pi_RA_2
Pi_RA_3
Pi_AZD_1
Pi_AZD_2
Pi_AZD_3
SampleName
PITG_04584
PITG_00401
PITG_06700
PITG_17205
PITG_16556
PITG_05058
PITG_06679
PITG_08447
PITG_05240
PITG_00194
PITG_02745
PITG_12932
PITG_12465
PITG_03802
PITG_10163
PITG_21600
PITG_00787
PITG_03818
PITG_12107
PITG_07174
PITG_22541
PITG_18421
PITG_17724
PITG_18718
PITG_17674
PITG_17404
PITG_00035
PITG_11635
PITG_01725
PITG_15975
PITG_12305
PITG_00032
PITG_00748
PITG_03672
PITG_08708
PITG_06262
PITG_19671
PITG_12871
PITG_01019
PITG_00036
PITG_05445
PITG_16813
PITG_04887
PITG_02536
PITG_01076
PITG_05630
PITG_12931
PITG_13300
PITG_16792
PITG_19178
PITG_18717
PITG_10819
PITG_13380
PITG_00052
PITG_00142
PITG_02593
PITG_09374
PITG_09222
PITG_19608
PITG_15652
PITG_08338
PITG_07308
PITG_06667
PITG_02887
PITG_03955
PITG_00597
PITG_05534
PITG_09432
PITG_11198
PITG_20593
PITG_06263

Fig 2: RIBOSOME_BIOGENESIS_IN_EUKARYOTES(PIF03008)
Blue-Pink O' Gram in the Space of the Analyzed GeneSet

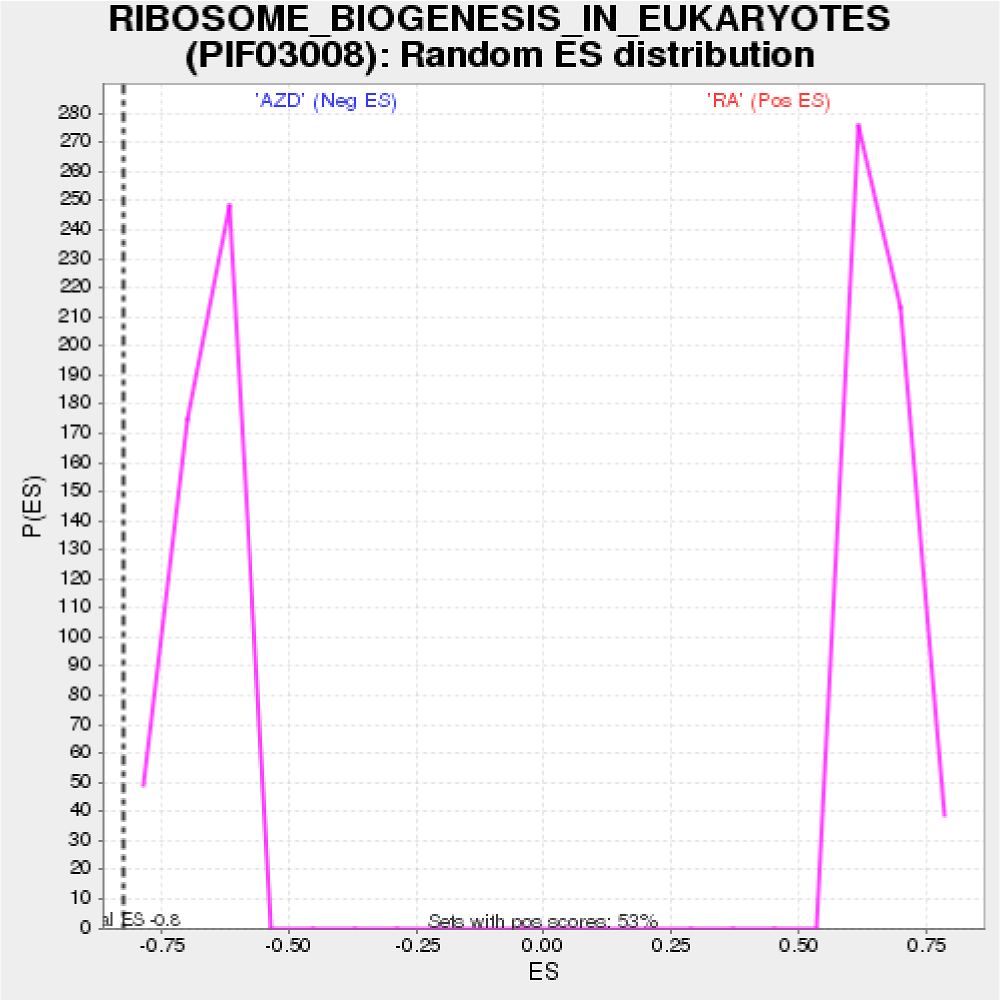

Fig 3: RIBOSOME_BIOGENESIS_IN_EUKARYOTES(PIF03008): Random ES distribution
Gene set null distribution of ES for RIBOSOME_BIOGENESIS_IN_EUKARYOTES(PIF03008)

9. RNA polymerase

Table: GSEA Results Summary

Dataset	fpkm.sample
Phenotype	sample.cls
Upregulated in class	AZD
GeneSet	RNA_POLYMERASE(PIF03020)
Enrichment Score (ES)	-0.6453421
Normalized Enrichment Score (NES)	-1.1896459
Nominal p-value	0.0
FDR q-value	0.14053668
FWER p-Value	0.101

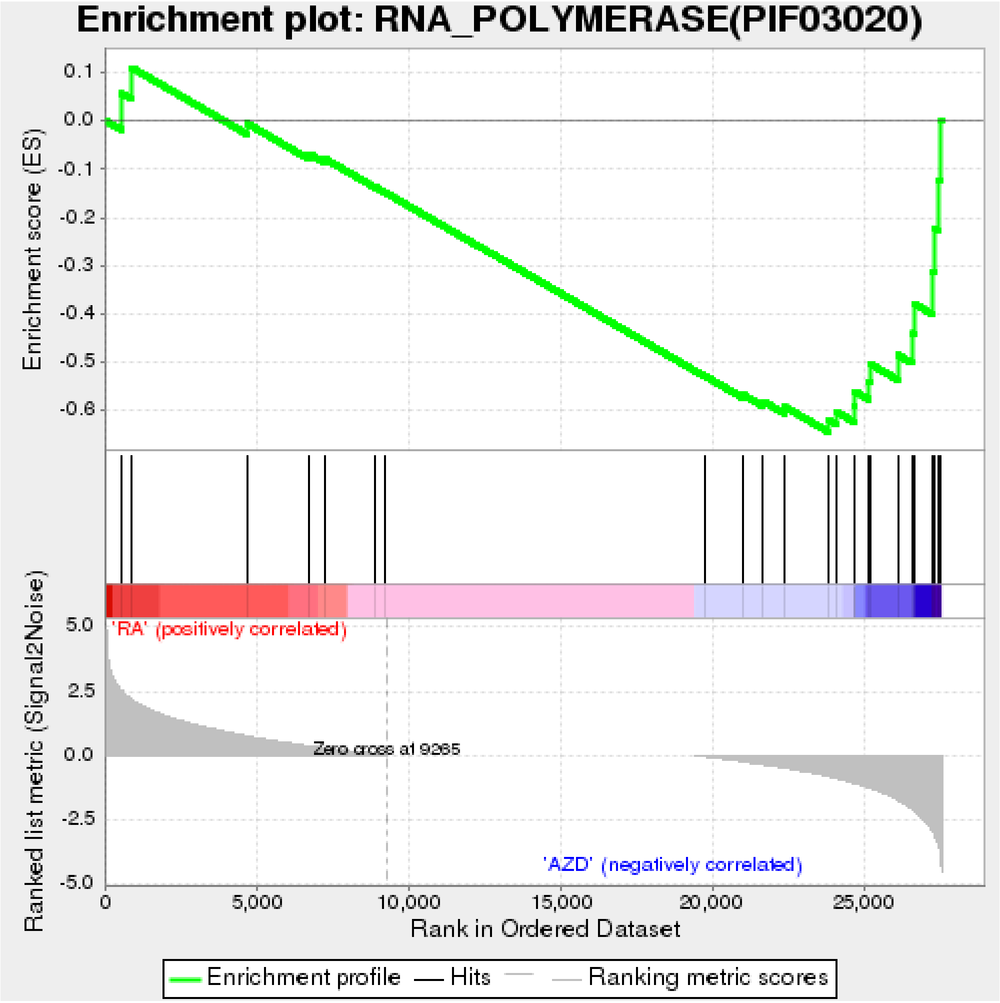

Fig 1: Enrichment plot: RNA_POLYMERASE(PIF03020)
Profile of the Running ES Score & Positions of GeneSet Members on the Rank Ordered List

Table: GSEA details [plain text format]

	PROBE	DESCRIPTION	GENE	GENE_TITLE	RANK IN	RANK	RUNNING	CORE

		(from dataset)	SYMBOL		GENE LIST	METRIC SCORE	ES	ENRICHMENT
1	Novel00232	Novel00232			503	2.543	0.0577	No
2	PITG_03793	PITG_03793			838	2.190	0.1109	No
3	PITG_05854	PITG_05854			4630	0.772	-0.0037	No
4	PITG_09425	PITG_09425			6697	0.381	-0.0673	No
5	PITG_04645	PITG_04645			7239	0.291	-0.0783	No
6	PITG_14885	PITG_14885			8888	0.055	-0.1365	No
7	PITG_18727	PITG_18727			9177	0.014	-0.1465	No
8	PITG_21077	PITG_21077			19730	-0.044	-0.5284	No
9	PITG_03795	PITG_03795			21012	-0.242	-0.5677	No
10	PITG_10445	PITG_10445			21681	-0.357	-0.5813	No
11	PITG_08383	PITG_08383			22377	-0.486	-0.5920	No
12	PITG_13404	PITG_13404			23846	-0.822	-0.6208	Yes
13	PITG_00051	PITG_00051			24089	-0.896	-0.6028	Yes
14	PITG_11365	PITG_11365			24667	-1.072	-0.5918	Yes
15	PITG_16526	PITG_16526			24688	-1.079	-0.5603	Yes
16	PITG_06706	PITG_06706			25169	-1.249	-0.5404	Yes
17	PITG_12877	PITG_12877			25225	-1.271	-0.5045	Yes
18	PITG_18113	PITG_18113			26111	-1.731	-0.4849	Yes
19	PITG_16116	PITG_16116			26573	-2.067	-0.4400	Yes
20	PITG_09712	PITG_09712			26654	-2.140	-0.3790	Yes
21	PITG_16658	PITG_16658			27263	-2.960	-0.3127	Yes
22	PITG_14613	PITG_14613			27333	-3.119	-0.2221	Yes
23	PITG_16659	PITG_16659			27477	-3.551	-0.1212	Yes
24	PITG_18777	PITG_18777			27551	-4.160	0.0004	Yes

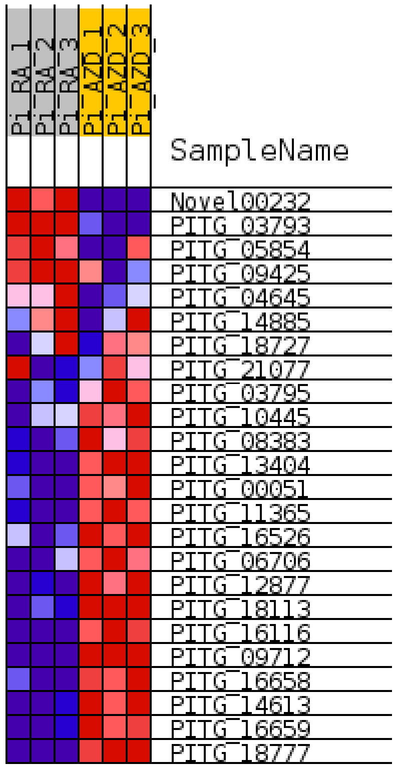

Fig 2: RNA_POLYMERASE(PIF03020)
Blue-Pink O' Gram in the Space of the Analyzed GeneSet

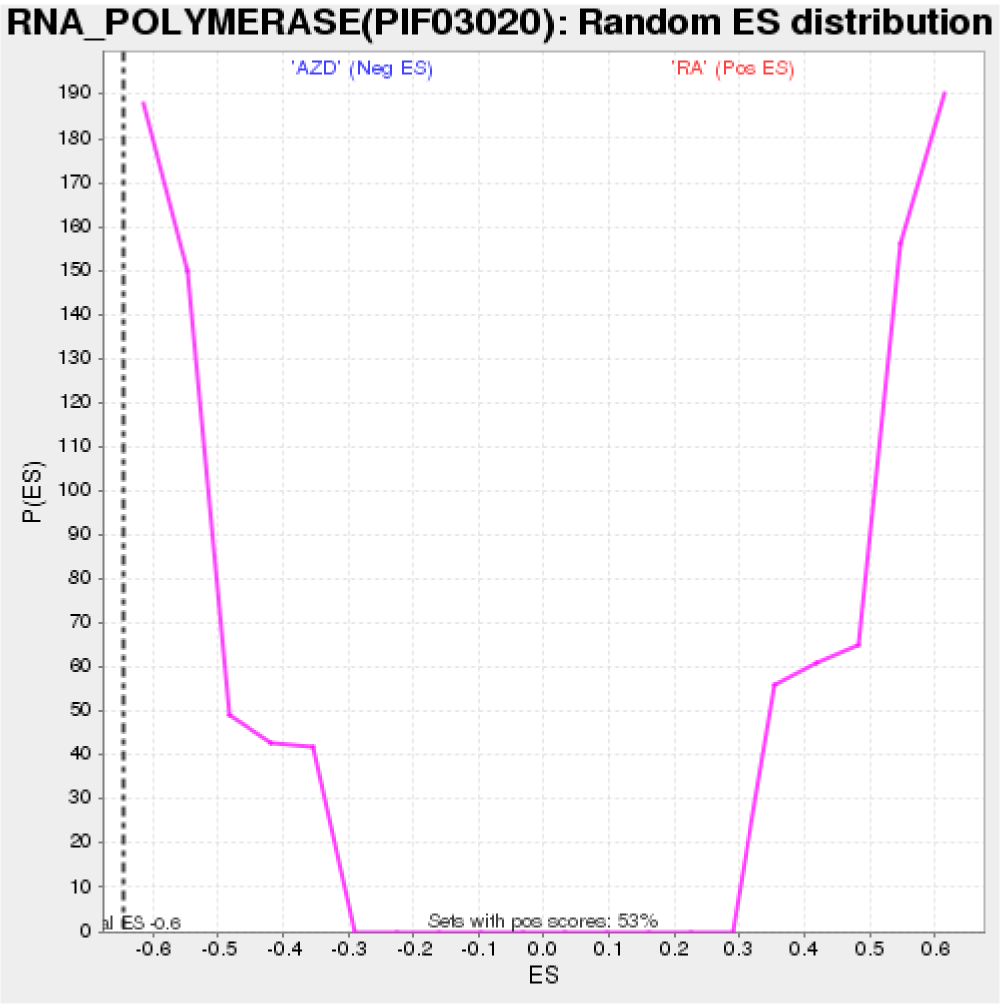

Fig 3: RNA_POLYMERASE(PIF03020): Random ES distribution
Gene set null distribution of ES for RNA_POLYMERASE(PIF03020)
