# Supplementary material for: TOR Inhibitors Synergistically Suppress the Growth and Development of Phytophthora infestans, a Highly Destructive Pathogenic Oomycete
Source: Front Microbiol. 2021 Apr 16;12:596874. doi: 10.3389/fmicb.2021.596874 (PMC8086431; doi:10.3389/fmicb.2021.596874)
Supplement: Supplementary Table 1 — (A) Primer sequence of qPCR. (B) Combination of RAP and AZD. (C) Combination of RAP and KU. (D) Combination of RAP and Torin1. [file Table_1.DOCX]

| P. infestans | | | | |
| --- | --- | --- | --- | --- |
| number | Gene_id | Interpro Description | primer(F) | primer(R) |
| 1 | PITG_12483 | Eukaryotic translation initiation factor 3, putative | TTCGCCAGTGAAGACGCCC | GCTGCTTGCCCTTTTTGAG |
| 2 | PITG_07885 | Eukaryotic translation initiation factor 2 subunit alpha | CCGTTTTTACGAGCAGAGA | CGGACAACAGAATCATACC |
| 3 | PITG_22657 | Elongation factor 1-alpha | AACTATGGGCAAGGAAAAG | AATAACGACCAGCGAGATG |
| 4 | PITG_08714 | Lysyl-tRNA synthetase | GTGGTGACATTGTGGGCGT | TTGAGGTAAAGTTCGGGGG |
| 5 | PITG_03768 | 50S ribosomal protein L4 | CGACCTGGAGTTGAAGATG | GCACAACACGCTGTAGGAT |
| 6 | PITG_17009 | Selenocysteine-specific elongation factor | TCCAGAACAAAGCCCCCAT | TACCAGGCACAGCAAAGCA |
| 7 | PITG_18420 | Protein kinase, putative | ATGGGCAATAAGGGATCCAAGGGCG | CGCCCTTGGATCCCTTATTGCCCAT |

**Table S1A** Primer sequence

**Table S1B** Combination of RAP and AZD

| **RAP**  **AZD** | 0 | 0.05 µM | 0.1 µM | 0.5 µM | 1 µM | 3 µM |
| --- | --- | --- | --- | --- | --- | --- |
| 0 | 0 | R 0.5 µL | R 1 µL | R 5 µL | R 10 µL | R 30 µL |
| 0.01 µM | A 0.01 µL | R 0.5  A 0.01 | R 1  A 0.01 | R 5  A 0.01 | R 10  A 0.01 | R 30  A 0.01 |
| 0.05 µM | A 0.5 µL | R 0.5  A 0.5 | R 1  A 0.5 | R 5  A 0.5 | R 10  A 0.5 | R 30  A 0.5 |
| 0.1 µM | A 1 µL | R 0.5  A 1 | R 1  A 1 | R 5  A 1 | R 10  A 1 | R 30  A 1 |
| 0.2 µM | A 2 µL | R 0.5  A 2 | R 1  A 2 | R 5  A 2 | R 10  A 2 | R 30  A 2 |

**Table S1C** Combination of RAP and KU

| **RAP**  **KU** | 0 | 0.05 µM | 0.1 µM | 0.5 µM | 1 µM | 3 µM |
| --- | --- | --- | --- | --- | --- | --- |
| 0 | 0 | R 0.5 µL | R 1 µL | R 5 µL | R 10 µL | R 30 µL |
| 0.5 µM | K 5 µL | R 0.5  K 5 | R 1  K 5 | R 5  K 5 | R 10  K 5 | R 30  K 5 |
| 1 µM | K 10 µL | R 0.5  K 10 | R 1  K 10 | R 5  K 10 | R 10  K 10 | R 30  K 10 |
| 2 µM | K 20 µL | R 0.5  K 20 | R 1  K 20 | R 5  K 20 | R 10  K 20 | R 30  K 20 |
| 5 µM | K 50 µL | R 0.5  K 50 | R 1  K 50 | R 5  K 50 | R 10  K 50 | R 30  K 50 |

**Table S1D** Combination of RAP and Torin1

| **RAP**  **Torin1** | 0 | 0.05 µM | 0.1 µM | 0.5 µM | 1 µM | 3 µM |
| --- | --- | --- | --- | --- | --- | --- |
| 0 | 0 | R 0.5 µL | R 1 µL | R 5 µL | R 10 µL | R 30 µL |
| 0.5 µM | T 5 µL | R 0.5  T 5 | R 1  T 5 | R 5  T 5 | R 10  T 5 | R 30  T 5 |
| 2 µM | T 20 µL | R 0.5  T 20 | R 1  T 20 | R 5  T 20 | R 10  T 20 | R 30  T 20 |
| 5 µM | T 50 µL | R 0.5  T 50 | R 1  T 50 | R 5  T 50 | R 10  T 50 | R 30  T 50 |
| 10 µM | T 100 µL | R 0.5  T 100 | R 1  T 100 | R 5  T 100 | R 10  T 100 | R 30  T 100 |
