# Supplementary material for: TOR Inhibitors Synergistically Suppress the Growth and Development of Phytophthora infestans, a Highly Destructive Pathogenic Oomycete
Source: Front Microbiol. 2021 Apr 16;12:596874. doi: 10.3389/fmicb.2021.596874 (PMC8086431; doi:10.3389/fmicb.2021.596874)
Supplement: Supplementary Table 4 — IC50 of RAP, Torin1 and RAP + Torin1 for P. infestans T30-4 and 002. [file Table_4.DOCX]

Table S4 The IC50 of RAP, Torin1, RAP + Torin1 for *P. infestans* T30-4 and 002.

|  | RAP（IC50） | Torin1（IC50） | RAP+Torin1（IC50） |
| --- | --- | --- | --- |
| T30-4 | 5 μm | 2 μm | 0.5 μm+0.3 μm |
| 002 | 0.5 μm | 1 μm | 0.05 μm+0.05 μm |
